# Supplementary material for: Catalytic asymmetric synthesis of planar-chiral dianthranilides via (Dynamic) kinetic resolution
Source: Nat Commun. 2024 May 29;15:4580. doi: 10.1038/s41467-024-48947-1 (PMC11136957; doi:10.1038/s41467-024-48947-1)
Supplement: Supplementary file 1 — Supplementary Information [file 41467_2024_48947_MOESM1_ESM.pdf]

Supplementary Information

Catalytic Asymmetric Synthesis of Planar-Chiral Dianthranilides via (Dynamic) Kinetic Resolution

Chun-Yan Guan,[1] Shuai Zou,[1] Can Luo,[1] Zhen-Yu Li,[1] Mingjie Huang,[1] Lihua Huang,[1,2]* Xiao Xiao,[3] Donghui Wei,[1] Min-Can Wang,[1] and Guang-Jian Mei[1,2]*

[1] Henan Key Laboratory of Chemical Biology and Organic Chemistry, College of Chemistry, Zhengzhou University, Zhengzhou 450001, China. Email: hlh606@zzu.edu.cn (L. Huang); meigj@zzu.edu.cn (G.-J. Mei).

[2] Pingyuan Laboratory (Zhengzhou University), Zhengzhou 450001, China.

[3] Collaborative Innovation Center of Yangtze River Delta Region Green Pharmaceuticals, Zhejiang University of Technology, Hangzhou 310014, China.

Table of Contents

1. General methods S3

2. Representative procedures S4

3. General procedures S7

4. Investigation on the rotational barriers S13

6. Characterization of racemic compounds (±)-**1** S23

7. Characterization of products S25

8. Crystallographic data collection for compound **3m** S51

9. NMR spectra S52

10. HPLC spectra S138

11. General procedure for the in vitro antitumor activity study S210

12. DFT Computational studies S213

13. Supplementary references S217

Supplementary Methods

1. General methods

Unless otherwise specified, all reactions were conducted under an inert atmosphere and anhydrous conditions. All the solvents were purified according to the standard procedures. All chemicals which are commercially available were employed without further purification. Thin-layer chromatography (TLC) was performed on silica gel plates (60F-254) using UV-light (254 nm). Flash chromatography was conducted on silica gel (200–300 mesh). ^{1}H and ^{13}C NMR spectra were recorded at ambient temperature in $CDCl_3$ or d_6-DMSO on a 400 MHz NMR spectrometer. Chemical shifts were reported in parts per million (ppm). The data are reported as follows: for ^{1}H NMR, chemical shift in ppm from tetramethylsilane with the solvent as internal standard ($CDCl_3$ δ 7.26 ppm), multiplicity (s = singlet, d = doublet, t = triplet, q = quartet, m = multiplet or overlap of non-equivalent resonances), integration; for ^{13}C NMR, chemical shift in ppm from tetramethylsilane with the solvent as internal indicator ($CDCl_3$ δ 77.1 ppm), multiplicity with respect to protons. All high-resolution mass spectra were obtained on a Q-TOF Micro LC/MS System ESI spectrometer to be given in m/z. Enantiomeric excesses values were determined with HPLC (chiral column; mobile phase *n*-hexane/*i*-PrOH). MBH carbonic esters **2** were synthesized according to modified literature-reported procedures[2].

2. Representative procedures

2.1 Optimization of the reaction conditions of DKR

Supplementary Table 1. Effect of solvents and catalysts

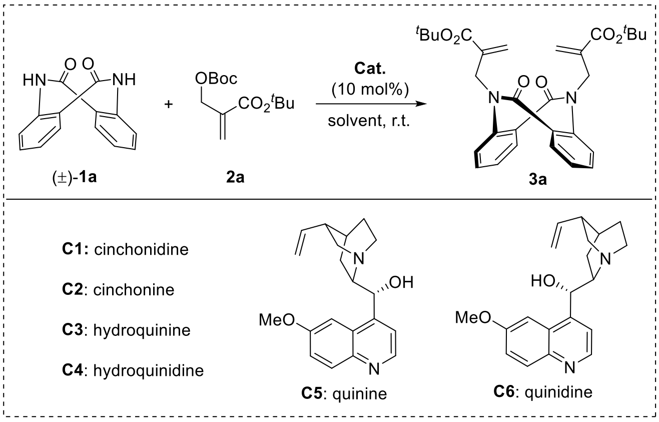

Entry	Cat.	solvent	yield (%)[b]	*ee* (%)[c]
1	**C1**	CH_2Cl_2	45	18
2	**C1**	toluene	50	10
3	**C1**	THF	70	40
4	**C1**	CH_3CN	89	83
5	**C2**	CH_3CN	63	-75
6	**C3**	CH_3CN	87	83
7	**C4**	CH_3CN	80	-90
8	**C5**	CH_3CN	95	98
9	**C6**	CH_3CN	90	-97

[a]Unless indicated otherwise, reaction conditions: **1a** (0.05 mmol), **2a** (0.12 mmol), and **Cat.** (10 mol%) in a specified solvent (1 mL) at room temperature (r.t.) for 6 h. [b]Isolated yield. [c]Determined by chiral HPLC.

2.2 Optimization of the reaction conditions of KR

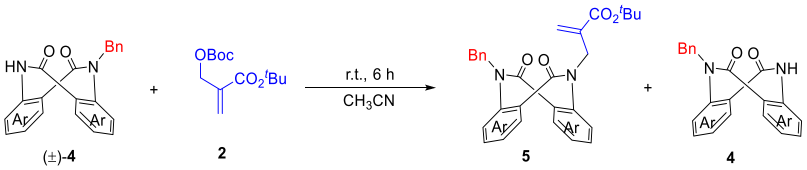

Supplementary Table 2. Effect of catalysts

Entry	Cat.	Yield (%)		*ee* (%)	
		**4**	**5**	**4**	**5**
1	**C1**	57	42	30	73

2	**C2**	62	37	-27	-63
3	**C3**	52	46	53	82
4	**C4**	55	41	-50	-87
5	**C5**	50	45	23	87
6	**C6**	58	42	-65	-87

Reaction conditions: **4** (0.1 mmol), **2** (0.06 mmol), and **Cat.** (10 mol%) in CH_3CN (1 mL) at room temperature (r.t.) for 6 h, isolated yield, *ee* was determined by chiral HPLC.

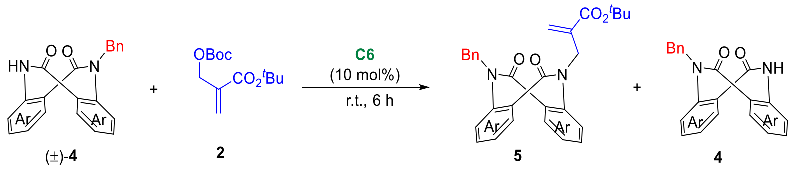

Supplementary Table 3. Effect of solvents

Entry	Solvent	Yield (%)		*ee* (%)	
		**4**	**5**	**4**	**5**
1	DCM	60	37	-15	-55
2	Toluene	61	36	-20	-51
3	CH_3CN	53	46	-65	-88
4	1,4-Dioxane	62	35	-23	-87
5	MeOH	59	37	-35	-50

Reaction conditions: **4** (0.1 mmol), **2** (0.06 mmol), and **C6** (10 mol%) in sol. (1 mL) at room temperature (r.t.) for 6 h, isolated yield, *ee* was determined by chiral HPLC.

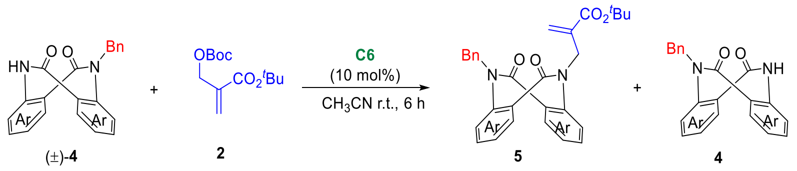

Supplementary Table 4. Effect of additive

Entry	Additive	Yield (%)		*ee* (%)	
		**4**	**5**	**4**	**5**
1	3Å MS	55	35	-47	-85
2	4Å MS	62	30	-27	-85
3	5Å MS	65	30	-32	-85
4	Na_2CO_3	61	32	-56	-77
5	K_2CO_3	57	40	-70	-75
6	Cs_2CO_3	61	35	-45	-56
7	CH_3COONa	63	33	-67	-85
8	H_2O	66	30	-58	-77

Reaction conditions: **4** (0.1 mmol), **2** (0.06 mmol), additive (0.01 mmol) and **C6** (10 mol%) in CH_3CN (1 mL) at room temperature (r.t.) for 6 h, isolated yield, *ee* was determined by chiral HPLC.

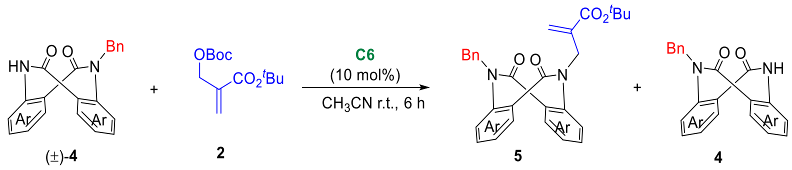

Supplementary Table 5. Effect of equivalent

Entry	Equiv	Yield (%)		*ee* (%)	
		**4**	**5**	**4**	**5**
1	1:0.5	40	43	-65	-87
2	1:0.6	50	44	-91	-91
3	1:0.7	45	49	-92	-87
4	1:0.8	49	50	-91	-80

Reaction conditions: **4** (0.1 mmol), **2**, and **C6** (10 mol%) in CH_3CN (1 mL) at room temperature (r.t.) for 6 h, isolated yield, *ee* was determined by chiral HPLC.

3. General procedures

3.1 The Synthesis of racemic compounds (±)-1

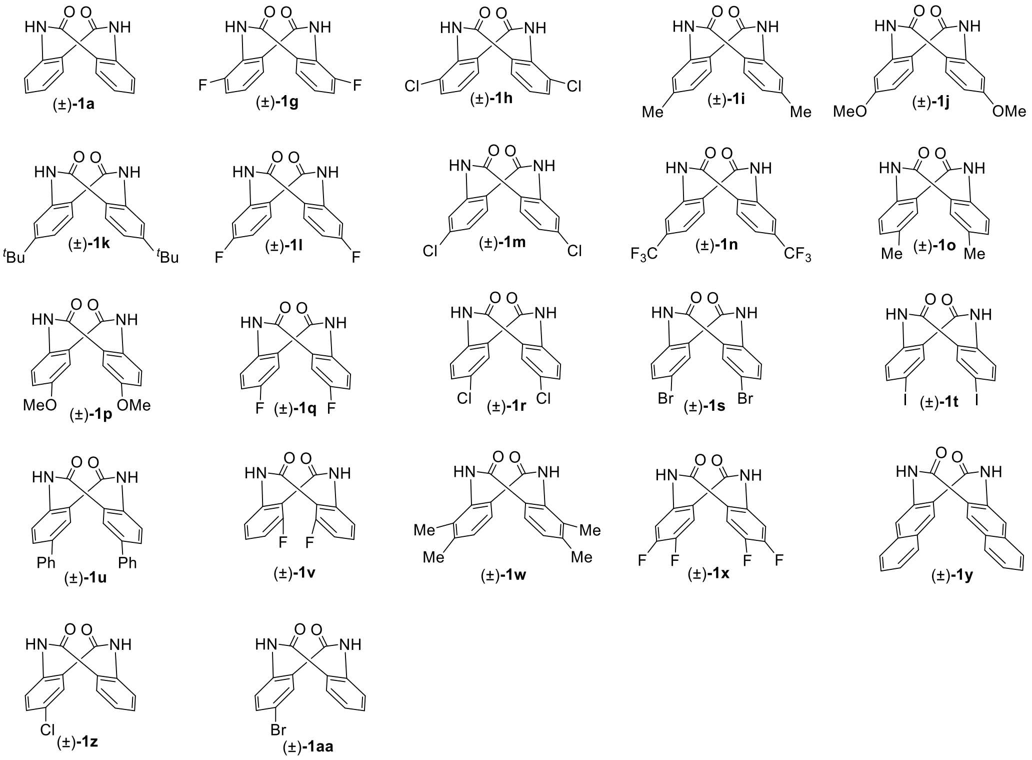

Supplementary Figure 1. The scope racemic compounds (±)-1

Method A:

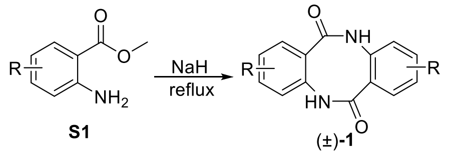

Substrate **1a – 1y** was synthesized with **Method A**

General procedure of **method A:**

Synthesis of (±)**-1**: o-Aminobenzoate (1.0 equiv) was added dropwise to the suspension of NaH (3.0 equiv, 60% in oil) in anhydrous THF at room temperature. The resulting mixture was gradually heated to reflux and stirred for 2 days. The mixture was cooled to room temperature and then poured slowly into 1 M HCl and ice. After the ice had melted, the precipitated product was collected by filtration, washed several times with cold water, and vacuum-dried at 50 °C to yield the crude product **1**, which was then purified by column chromatography or used directly.

Method B:

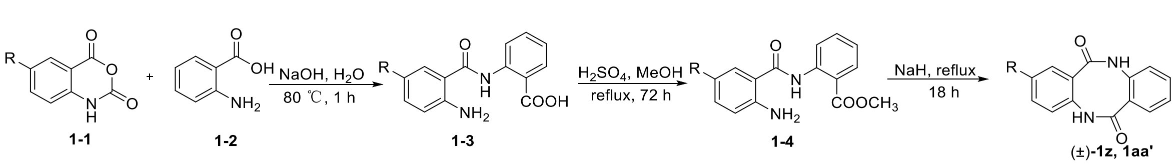

Substrate (±)-**1z, 1aa** was synthesized with **Method B**

General procedure of **method B:**

Synthesis of **1-3**: A suspension of **1-1** (1 equiv), **1-2** (1 equiv) and sodium hydroxide (1 equiv) in water (10 mL/mmol) were heated at 80 °C for 1 h until the evolution of carbon dioxide had ceased and a clear solution had formed. After cooling the reaction mixture, the obtained solution was diluted with water, and the crude product was precipitated by addition of glacial acetic acid, filtered and dried under vacuum. The crude product was extracted with ethyl acetate, the organic phase was washed with brine and dried over anhydrous magnesium sulfate. Evaporation of the solvent left a glass-like residue which was used in next step without further purification.

Synthesis of **1-4**: **1-3** was dissolved in methanol then, concentrated sulfuric acid was added (0.5 mL/mmol). The resulted solution was refluxed for 72 h. The excess of methanol was evaporated and the resulting residue was added to water. The pH was adjusted to 8 by addition of NaOH and the crude products were extracted with ethyl acetate. The combined organic layers were washed with 1 N sodium hydroxide, water and brine, and then dried over anhydrous magnesium sulfate. Evaporation of solvent yielded a dark residue of crude methyl esters **1-4**, which was used in next step without further purification.

Synthesis of **1z-1aa**: **1-4** was dissolved in the anhydrous THF (20 mL/mmol) then 60% sodium hydride in mineral oil (2 equiv) was added and the resulted solution was refluxed for 18 h. The excess of THF was evaporated, the obtained residue was poured into 1 N HCl and the crude product was extracted with ethyl acetate. The combined organic layers were washed with 1 N HCl, ice water and brine, then dried over anhydrous magnesium sulfate. Crude products were purified by column chromatography using petroleum ether/ethyl acetate = 1:1.

3.2 The synthesis of compounds 3

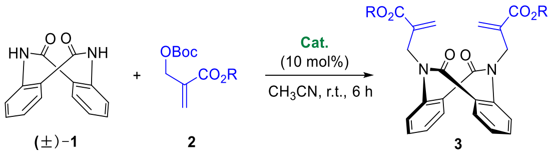

Octa-lactam **1** (0.10 mmol) and the **C5** (10 mol%) were dissolved in CH_3CN and MBH carbonic esters **2** (0.24 mmol) was added. The reaction mixture was stirred for 6 h at room temperature. The solvent was removed in vacuo and the crude product was separated by flash column chromatography on silica gel (petroleum ether/ethyl acetate = 4:1) to afford **3**.

3.3 The synthesis of compounds 4a-4m

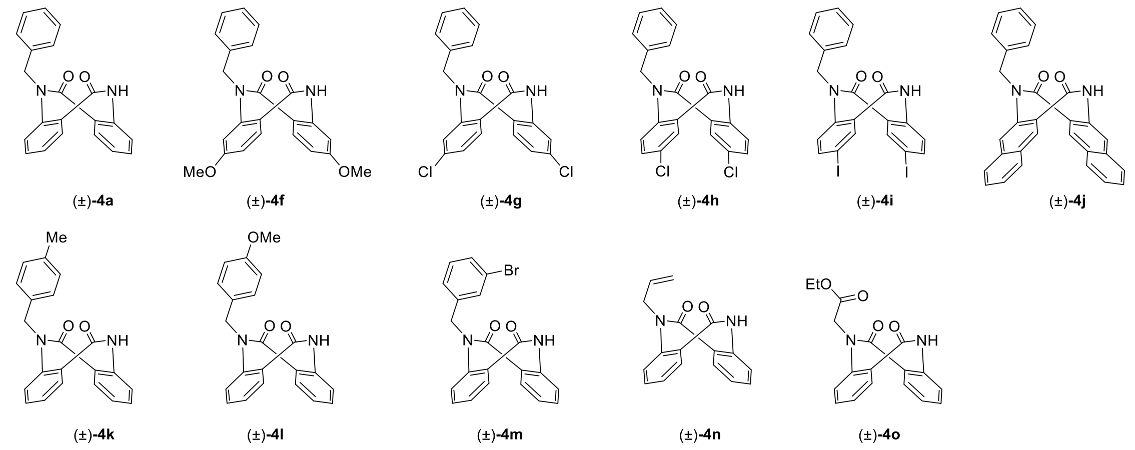

Supplementary Figure 2. The scope of compounds (±)-4

Method C:

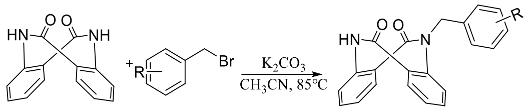

(±)-1a 4-1 (±)-4

Substrate **4a – 4m** was synthesized with **Method C**

General procedure of **method C:**

Synthesis of (±)**-4**: Octa-lactam **1** (1.0 equiv) and the K_2CO_3 (2.0 equiv) were dissolved in CH_3CN and benzyl bromide **4-1** (1.0 equiv) was added. The reaction mixture was stirred for 3 h at 85°C. After completion of the reaction as indicated by TLC analysis, the reaction mixture was quenched with saturated NH_4Cl solution (25 mL), and extracted with EA (3×30 mL). The combined organic layers were washed with brine (30 mL), dried over Na_2SO_4, filtered and concentrated to give a residue, which was purified by column chromatography isolation (petroleum ether/ethyl acetate = 2:1–1:1) to afford product **4**.

Method D:

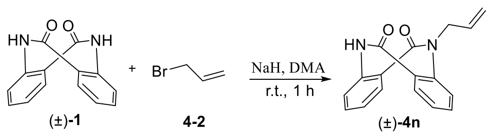

(±)-1 4-2 (±)-4n

Substrate **4n** was synthesized with **Method D**

Synthesis of (±)**-4n**: Octa-lactam **1** (1.0 equiv) and the allyl bromide **4-2** (2.0 equiv) were dissolved in DMA and NaH (1.0 equiv) was added slowly. The reaction mixture was stirred for 1 h at room temperature. After completion of the reaction as indicated by TLC analysis, the reaction mixture was quenched with saturated NH_4Cl solution (25 mL), and extracted with EA (3×30 mL). The combined organic layers were washed with brine (3×30 mL), dried over Na_2SO_4, filtered and concentrated to give a residue, which was purified by column chromatography isolation (petroleum ether/ethyl acetate = 3:1 – 1:1) to afford product **4o**.

Method E:

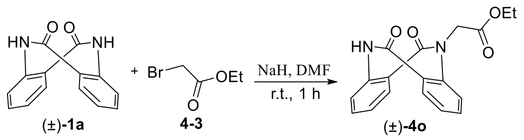

Substrate **4o** was synthesized with **Method E**

Synthesis of (±)-**4o**: Octa-lactam **1** (1.0 equiv) and the ethyl bromoacetate **4-3** (2.0 equiv) were dissolved in DMF and NaH (1.0 equiv) was added slowly. The reaction mixture was stirred for 1 h at room temperature. After completion of the reaction as indicated by TLC analysis, the reaction mixture was quenched with saturated NH_4Cl solution (25 mL), and extracted with EA (3×30 mL). The combined organic layers were washed with brine (3×30 mL), dried over Na_2SO_4, filtered and concentrated to give a residue, which was purified by column chromatography isolation (petroleum ether/ethyl acetate = 2:1) to afford product **4o**.

3.4 The synthesis of compounds 5

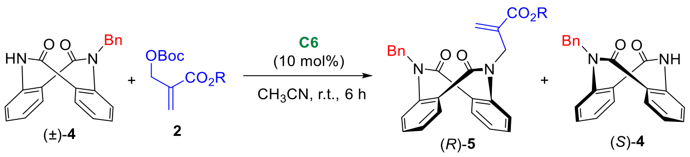

Benzyl protected octa-lactam **4** (0.10 mmol) and the catalyst **C6** (10 mol%) were dissolved in CH_3CN and MBH carbonic esters **2** (0.06 mmol) was added. The reaction mixture was stirred for 6 h at room temperature. The solvent was removed in vacuo and the crude product was separated by flash column chromatography on silica gel (petroleum ether/ethyl acetate = 3:1 – 1:1) to afford recovered (*S*)-**4** and (*R*)-**5**.

3.5 The synthesis of compounds 11

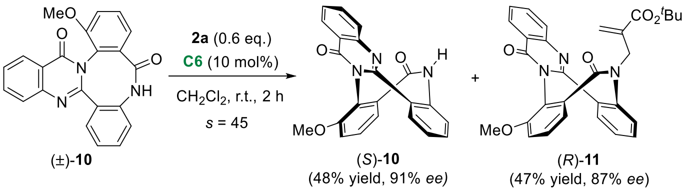

(±)-**10** (0.10 mmol) (synthesized in 5 steps according to Tan's synthetic route, see text ref. 53) and the catalyst **C6** (10 mol%) was dissolved in DCM and MBH carbonic ester **2** (0.06 mmol) was added. The reaction mixture was stirred for 2 h at room temperature. The solvent was removed in vacuo and the crude product was separated by flash column chromatography on silica gel (petroleum ether/ethyl acetate = 1:1) to afford recovered (*S*)-**10** and (*R*)-**11**. The racemic **11** was synthesized according to the above method using DMAP instead of **C6**.

3.6 The synthesis of compounds (*R*)-10

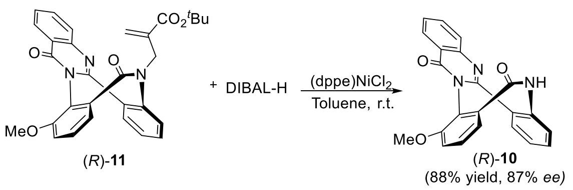

To a 10 mL Schlenk tube, (*R*)**-11** (0.10 mmol) and the (dppe)$NiCl_2$ (4 mol%) were dissolved in toluene under N_2 and the DIBAL-H (1.5 equiv) was added slowly, stirred for 24 h at room temperature. The solvent was removed in vacuo and the crude product was separated by flash column chromatography on silica gel (petroleum ether/ethyl acetate = 1:1) to afford product (*R*)-**10** in 88% yield with 87% *ee*.

3.7 The synthesis of compounds 12

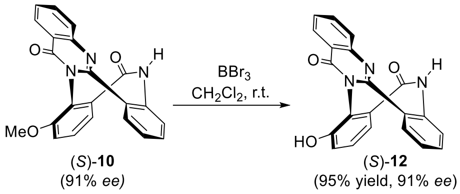

To a 10 mL Schlenk tube, (*S*)-**10** (25 mg, 0.68 mmol) and 4.0 mL anhydrous DCM were added under N_2, BBr_3(47 µL, 4.05 mmol) was added slowly and the mixture was stirred for 24 h at room temperature. The solution was slowly added to 10 mL saturated $NaHCO_3$ solution and the resulting mixture was acidified with 1M HCl. The aqueous phase was extracted with EA(3 × 5 mL), the combined organic layer was dried over Na_2SO_4 and concentrated under reduced pressure. The residual white solid was purified through flash chromatography on silica gel to yield corresponding natural product (+)-**Eupolyphagin** in 95% yield with 91% *ee* as a white solid. The (**-**)-**Eupolyphagin** was synthesized according to the above method using (*R*)-**10.** The racemic **Eupolyphagin** was synthesized according to the above method using racemic **10**.

3.8 Procedure for the gram-scale reaction

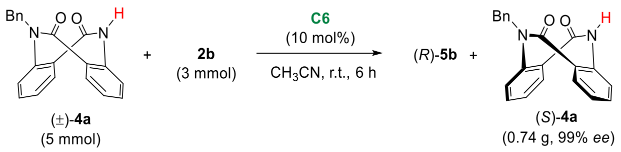

To a stirring anhydrous CH_3CN solution (20 mL) of benzyl protected octa-lactam **4a** (5 mmol) and **2b** (3 mmol) were added **C6** (10 mol%) at room temperature. The reaction mixture was stirred until completion of reaction (6 hours, as monitored by TLC). Water was added and the mixture was extracted with AcOEt (3 × 20 mL). The combined organic layer was washed with brine, separated, dried over Na_2SO_4 and filtered. The solvent was then removed under reduced pressure and the residue was purified by column chromatography isolation (petroleum ether/ethyl acetate = 3:1–1:1) to afford product (*R*)-**5b** (1.10 g) in 50% yield with 92% *ee* and recovered (*S*)-**4a** (0.74 g) in 45% yield with 99% *ee*.

3.9 The synthesis of compounds (*S*)-1a'

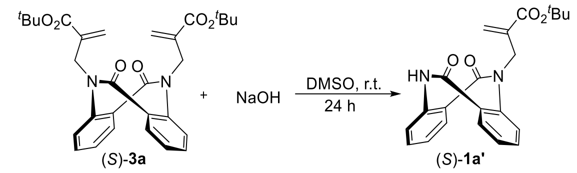

To a 10 mL Schlenk tube, (*S*)-**3a** (50.8 mg, 0.1 mmol) and 4.0 mL DMSO were added under N_2, NaOH (8 mg, 0.2 mmol) was added and the mixture was stirred for 24 h at room temperature. The residual white solid was purified through flash chromatography on silica gel (petroleum ether/ethyl acetate = 1:1) to yield corresponding (*S*)-**1a'** in 80% yield with 98% *ee* as a white solid.

3.10 The synthesis of compounds (*R*)-4a

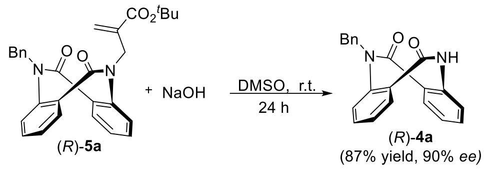

To a 10 mL Schlenk tube, (*R*)-**5a** (0.10 mmol) and 4.0 mL DMSO were added under N_2, NaOH (8 mg, 0.2 mmol) was added and the mixture was stirred for 24 h at room temperature. The residual white solid was purified through flash chromatography on silica gel (petroleum ether/ethyl acetate = 2:1) to yield corresponding (*R*)-**4a** in 85% yield with 90% *ee* as a white solid.

3.11 The synthesis of compounds 6

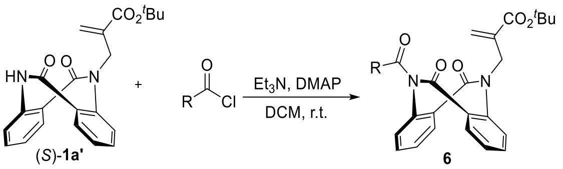

(*S*)**-1a'** (99% *ee*) (0.10 mmol), the Et_3N (2.0 equiv) and DMAP (0.1 equiv) were dissolved in DCM and the acyl chloride (1.5 equiv) was added slowly. stirred for 30 mins at room temperature. The solvent was removed in vacuo and the crude product was separated by flash column chromatography on silica gel (petroleum ether/ethyl acetate = 3:1) to afford product **6** in 98% yield with 99% *ee*.

3.12 The synthesis of compounds 8

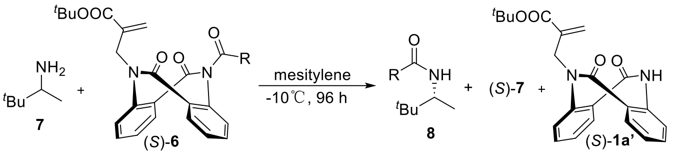

Racemic-**7** (0.10 mmol) was dissolved in mesitylene (6 mL) in the -25°C, and the (*S*)-**6** (0.5 equiv in 6 mL mesitylene) was added slowly within 60 mins. After completion (monitored by TLC), the solvent was removed under reduced pressure and the crude product was directly purified by flash chromatography on silica gel

employing mixtures of petroleum and ethyl acetate (petroleum ether/ethyl acetate = 3:1) as eluents to afford the desired product **8 in** 47% yield with 81% *ee* and the (*S*)-**1a**' in 48% yield and 99% *ee*.

4. Investigation on the rotational barriers

4.1 Investigation on the rotational barrier of 1a

Compound (*S*)-**1a** (0.1 mmol) was dissolved in acetonitrile (5 mL) in a sealed vial, and the mixture was stirred at 30 °C. At given interval of time, small samples were removed and subjected into the HPLC to measure the enantiomeric excess.

The slope of the first-order kinetic line gives the racemization constant ($k_{racemization}$ = 2×$k_{enantiomerisation}$). Eyring equation gives the enantiomerisation barrier from enantiomerisation constant ($k_{enantiomerisation}$), R = 8.31451 J·K^{-1}·mol^{-1}, h = 6.62608×10^{-34} J·s and k_B = 1.38066×10^{-23} J·K^{-1}.

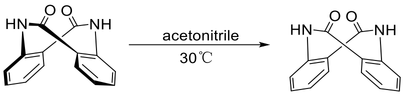

Supplementary Table 6. Rotational barrier of **1a**

Time	Major **1a**	minor **1a**	M+m	M-m	ln[(M+m)/(M-m)]
0	91.62	8.38	100	83.24	0.183442
300	89.807	10.193	100	79.614	0.22798
600	86.282	13.718	100	72.564	0.320701
900	84.357	15.643	100	68.714	0.375217
1200	82.153	17.847	100	64.306	0.441517
1500	80.393	19.607	100	60.786	0.497811
1800	77.809	22.191	100	55.618	0.586663
2100	75.814	24.186	100	51.628	0.661106
2400	74.802	25.198	100	49.604	0.701099
2700	72.171	27.829	100	44.342	0.813238
3000	70.845	29.155	100	41.69	0.874909
3300	67.708	32.292	100	35.416	1.038006

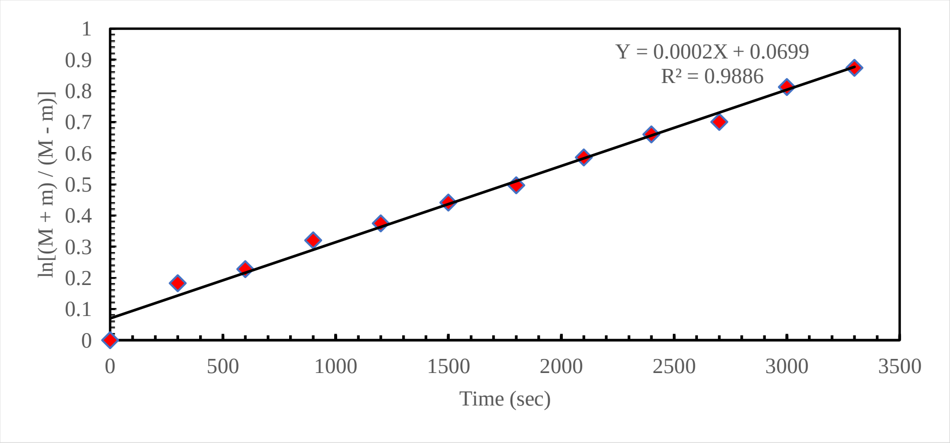

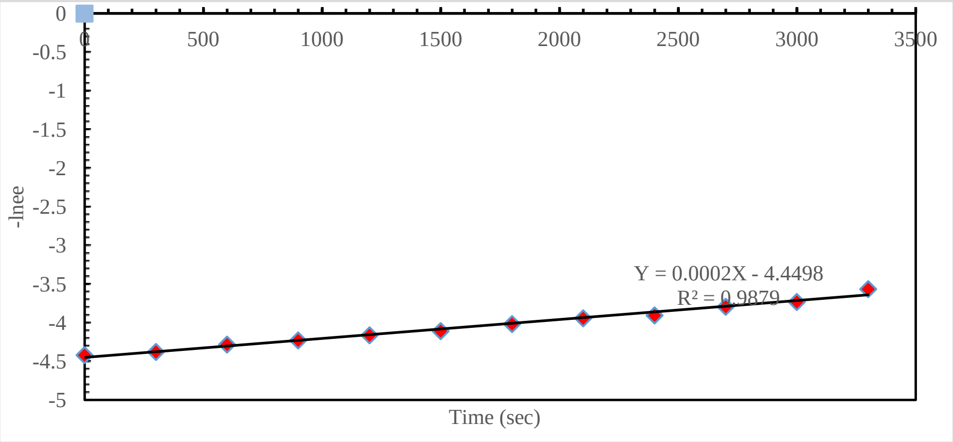

Supplementary equation (1): $-\ln(ee) = 2k_{ent}t + C$, $k_{ent} = 1*10^{-4}\ s^{-1}$

Supplementary equation (2): $t_{1/2}{}^{30}{}_{rac} = ln(2)/2\ k_{ent} = 3465.7\ s = 0.96\ h$

$t_{1/2}{}^{25}{}_{rac} = 13409.1\ s = 3.72\ h$

Supplementary equation (3): $\Delta G = -RT\ln(k_{ent} * h / k_B * T) = 97.5\ kJ/mol = 23.3\ kcal/mol$

4.2 Investigation on the rotational barrier of 3a

Compound (*S*)-**3a** (0.1 mmol) was dissolved in dimethylbenzene (5 mL) in a sealed vial, and the mixture was stirred at 125 °C. At given interval of time, small samples was removed and subjected into the HPLC to measure the enantiomeric excess.

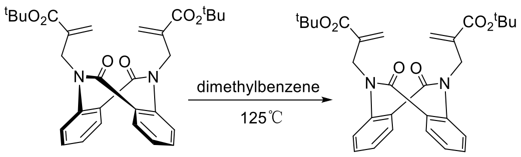

Supplementary Table 7. Rotational barrier of **3a**

Time	Major **3a**	minor **3a**	M+m	M-m	ln[(M+m)/(M-m)]
0	97.56	2.44	100	95.12	0.050031
3600	95.48	4.52	100	90.96	0.09475
7200	92.51	7.49	100	85.02	0.162284
10800	88.74	11.26	100	77.48	0.25515

14400	87.96	12.04	100	75.92	0.27549
18000	85.44	14.56	100	70.88	0.344182
21600	83.87	16.13	100	67.74	0.389493

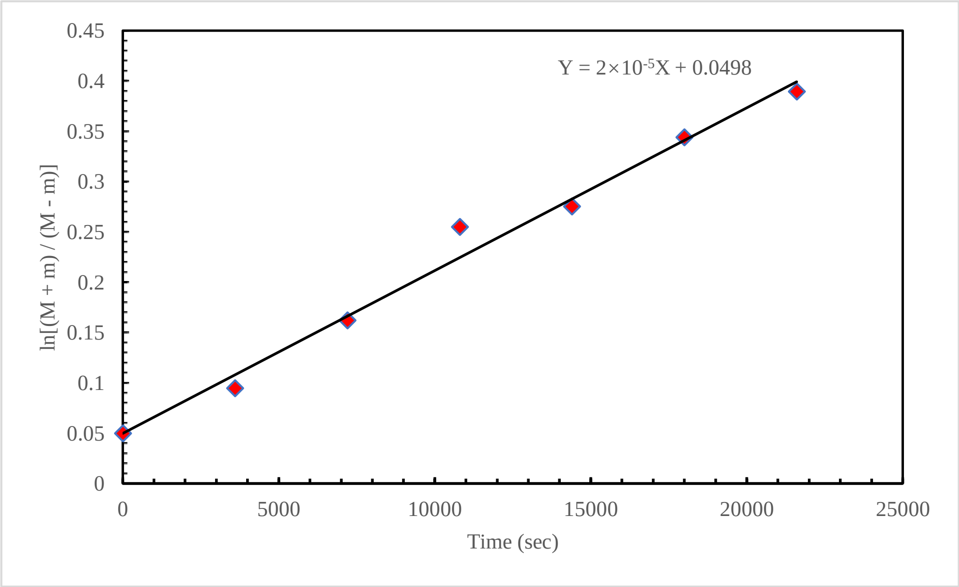

$k_{racemization}(125\ °C) = 1\times10^{-5}\ s^{-1}$

$k_{enantiomerization}(125\ °C) = 1.21\times10^{-18}\ s^{-1}$

$\Delta G^{\ddagger} = 32.6$ kcal/mol

4.3 Investigation on the rotational barrier of 3l

Compound (*S*)-**3l** (0.1 mmol) was dissolved in dimethylbenzene (5 mL) in a sealed vial, and the mixture was stirred at 130 °C. At given interval of time, small samples were removed and subjected into the HPLC to measure the enantiomeric excess.

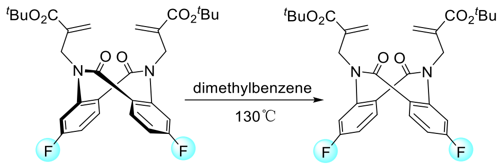

Supplementary Table 8. Rotational barrier of **3l**

Time	Major **3l**	minor **3l**	M+m	M-m	ln[(M+m)/(M-m)]
0	90.66	9.34	100	81.32	0.206778
900	89.74	10.26	100	79.48	0.229665
1800	88.01	11.99	100	76.02	0.274174
3600	85.83	14.17	100	71.66	0.333237
5400	83.79	16.21	100	67.58	0.391858
7200	80.66	19.34	100	61.32	0.489064
9000	78.67	21.33	100	57.34	0.556172

12600	72.76	27.24	100	45.52	0.787018
16200	68.52	31.48	100	37.04	0.993172
19800	63.3	36.7	100	26.6	1.324259

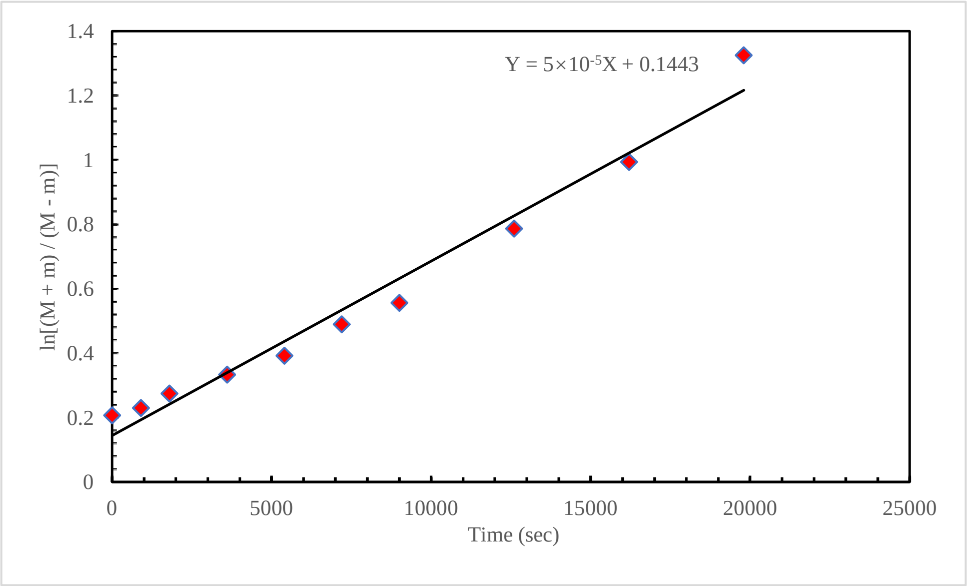

$k_{racemization}(130\ °C) = 2.5\times10^{-5}\ s^{-1}$

$k_{enantiomerization}(130\ °C) = 2.98\times10^{-18}\ s^{-1}$

$\Delta G^{\ddagger} = 32.3$ kcal/mol

4.4 Investigation on the rotational barrier of 3j

Compound (*R*)-**3j** (0.1 mmol) was dissolved in mesitylene (5 mL) in a sealed vial, and the mixture was stirred at 155 °C. At given interval of time, small samples were removed and subjected into the HPLC to measure the enantiomeric excess.

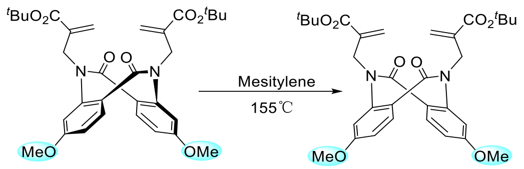

Supplementary Table 9. Rotational barrier of **3j**

Time	Major **3j**	minor **3j**	M+m	M-m	ln[(M+m)/(M-m)]
0	90.13	9.87	100	80.26	0.219899
1800	88.07	11.93	100	76.14	0.272596
3600	86.16	13.84	100	72.32	0.324069
5400	84.34	15.66	100	68.68	0.375712
7200	83.89	16.11	100	67.78	0.388903
9000	80.47	19.53	100	60.94	0.49528
10080	78.47	21.53	100	56.94	0.563172
12600	72.76	27.24	100	45.52	0.787018

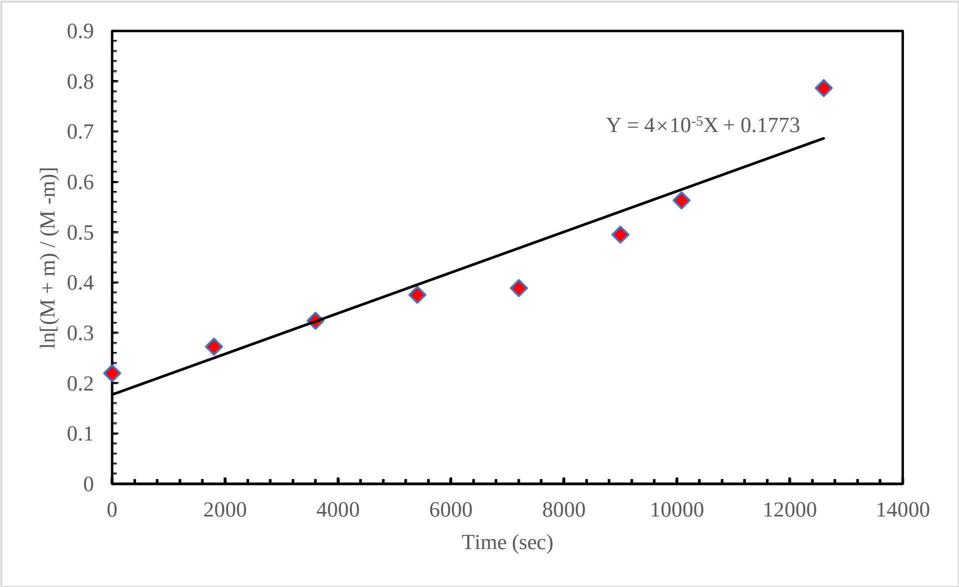

$k_{racemization}$(155 °C) = 2.0×10^{-5} s^{-1}

$k_{enantiomerization}$(155 °C) = 2.24×10^{-18} s^{-1}

ΔG‡ = 34.6 kcal/mol

4.5 Investigation on the rotational barrier of 4a

Compound (*S*)-**4a** (0.1 mmol) was dissolved in toluene (5 mL) in a sealed vial, and the mixture was stirred at 70 °C. At given interval of time, small samples were removed and subjected into the HPLC to measure the enantiomeric excess.

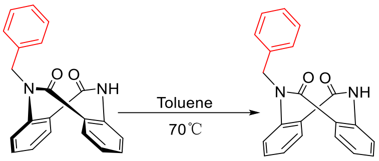

Supplementary Table 10. Rotational barrier of **4a**

Time	Major **4a**	minor **4a**	M+m	M-m	ln[(M+m)/(M-m)]
0	94.7	5.3	100	89.4	0.11205
1800	93.12	6.88	100	86.24	0.148036
3600	90.78	9.22	100	81.56	0.203831
5400	89.04	10.96	100	78.08	0.247436
7200	87.8	12.2	100	75.6	0.279714
9000	86.72	13.28	100	73.44	0.308701
10800	85.22	14.78	100	70.44	0.350409
12600	84.12	15.88	100	68.24	0.382139
14400	82.26	17.74	100	64.52	0.438195
16200	80.64	19.36	100	61.28	0.489717
18000	79.02	20.98	100	58.04	0.544038
21600	74.84	25.16	100	49.68	0.699568

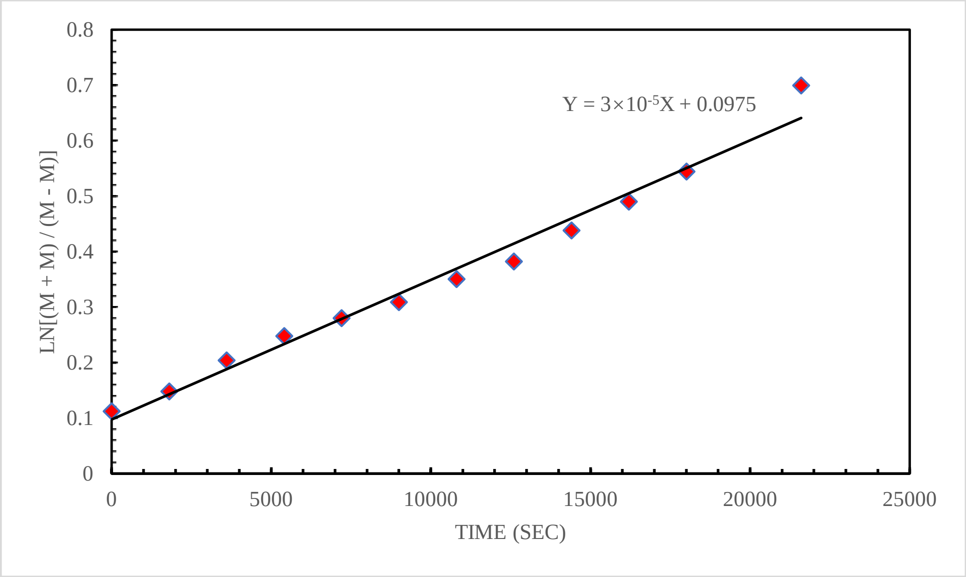

$k_{racemization}$(70 °C) = 1.5×10^{-5} s^{-1}

$k_{enantiomerization}$(70 °C) = 1.98×10^{-18} s^{-1}

ΔG‡ = 27.8 kcal/mol

4.6 Investigation on the rotational barrier of 1a′

Compound (*S*)- **1a′** (0.1 mmol) was dissolved in acetonitrile (5 mL) in a sealed vial, and the mixture was stirred at 80 °C. At given interval of time, small samples were removed and subjected into the HPLC to measure the enantiomeric excess.

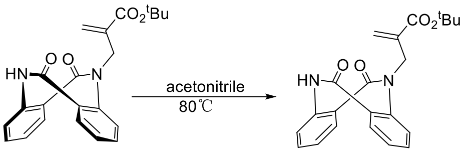

Supplementary Table 11. Rotational barrier of **1a′**

Time	Major **3a**	minor **3a**	M+m	M-m	ln[(M+m)/(M-m)]
0	97.619	2.381	100	95.238	0.048791
1800	96.523	3.477	100	93.046	0.072076
3600	94.499	5.501	100	88.998	0.116556
5400	91.335	8.665	100	82.670	0.190313
7200	87.641	12.359	100	75.282	0.283929
9000	83.742	16.258	100	67.484	0.393279
10800	80.034	19.966	100	60.068	0.509692
12600	77.247	22.753	100	54.494	0.607079
14400	75.121	24.879	100	50.242	0.688318
16200	74.576	25.424	100	49.152	0.710252

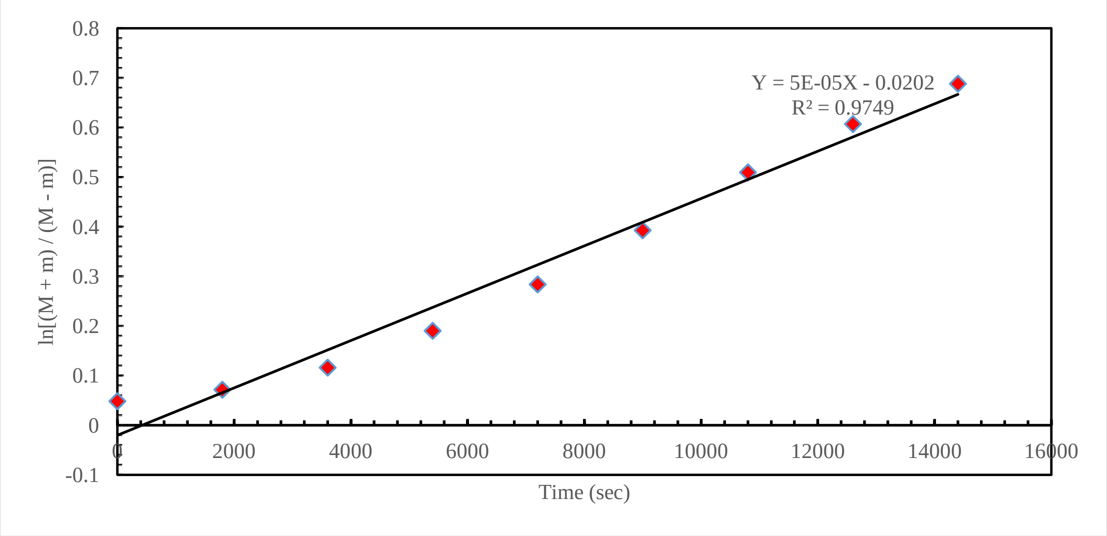

$k_{racemization}(80\ °C) = 2.5\times10^{-5}\ s^{-1}$

$k_{enantiomerization}(80\ °C) = 3.396\times10^{-18}\ s^{-1}$

$\Delta G^{\ddagger} = 28.2$ kcal/mol

5. Investigation of mechanism

5.1 Dynamic kinetic resolution process detection

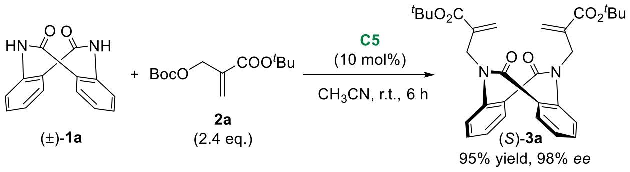

Octa-lactam (±)-**1a** (0.10 mmol) and the **C5** (10 mol%) was dissolved in CH_3CN and MBH carbonic ester **2a** (0.24 mmol) was added at room temperature. At given interval of time, small samples were removed and subjected into the HPLC to measure the enantiomeric excess of **1a** and **3a**.

Supplementary Table 12. DKR process

Time (min)	*ee* of **1a**	*ee* of **3a**
60	0	98.3
90	0	98.3
120	0	98.1
150	0	98.2
180	0	98.0
210	0	98.4
240	0	98.2
270	0	98.5

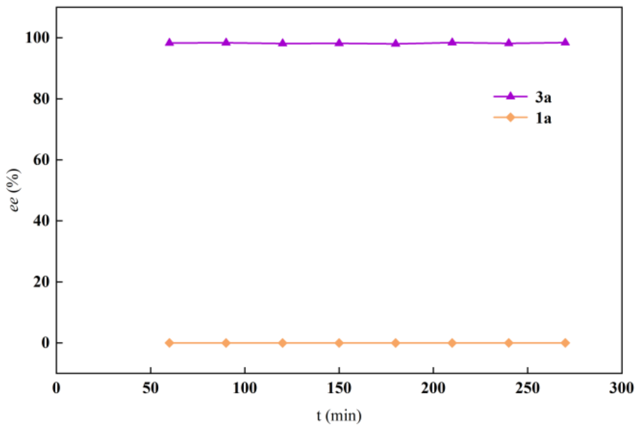

5.2 Kinetic resolution process detection

(*S*)- **1a′** (0.10 mmol)(74% *ee*) and the **C6** (10 mol%) was dissolved in CH_3CN and MBH carbonic esters **2a** (0.12 mmol) was added at room temperature. At given interval of time, small samples were removed and subjected into the HPLC to measure the enantiomeric excess of **1a′** and **3a**.

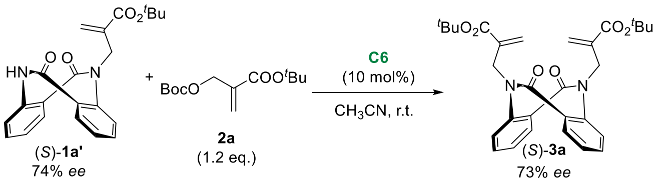

Supplementary Table 13. KR process

Time (min)	*ee* of **1a′**	*ee* of **3a**
0	73.8	
20	82.2	-44.1
25	85.6	-42.2
30	89.1	-26.6
40	94.1	-6.2
55	98.6	16.6
65	100	28.2
75	100	38.1
85	100	45.9
95	100	53.0
110	100	61.7
130	100	65.9
150	100	70.0
180	100	73.0

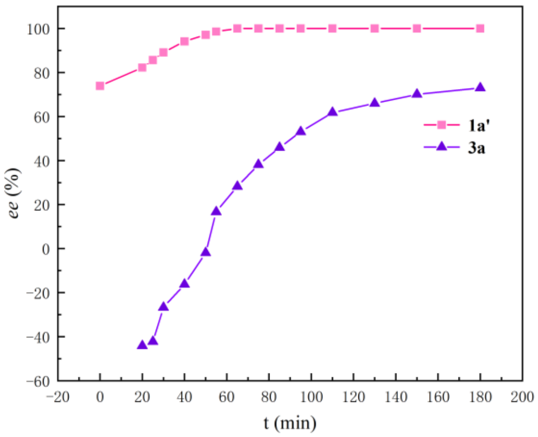

(±)-**1a′** (0.10 mmol) and the **C6** (10 mol%) was dissolved in CH3CN and MBH carbonic esters **2a** (0.12 mmol) was added at room temperature. At given interval of time, small samples were removed and subjected into the HPLC to measure the enantiomeric excess of **1a′** and **3a**.

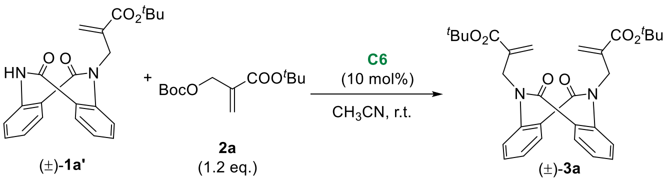

Supplementary Table 14. KR process detection

Time (min)	*ee* of **1a′**	*ee* of **3a**
20	11.1	93.4
40	14.3	89.6
70	19.1	87.6
100	23.5	81.8
130	35.9	74.2
190	98.5	58.2
210	99.9	42.39
270	100	19.7
300	100	13.4
330	100	7.77

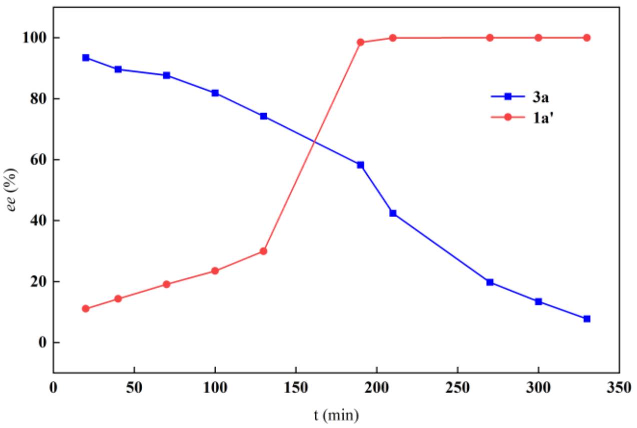

5.3 Dynamic resolution exploration

5.3.1 *ee* maintenance experiment

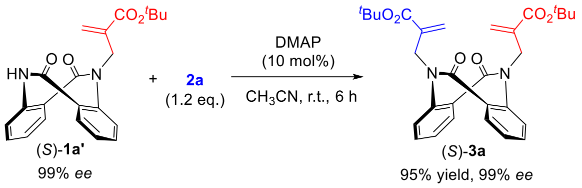

(*S*)-**1a′** (0.10 mmol) (99% *ee*) and the DMAP (10 mol%) was dissolved in CH_3CN and MBH carbonic esters **2a** (0.12 mmol) was added at room temperature. The reaction mixture was stirred until completion of reaction (as monitored by TLC). The solvent was then removed under reduced pressure and the residue was purified by flash column chromatography isolation (petroleum ether/ethyl acetate = 2:1) to afford product (*S*)-**3a** in 95% yield with 99% *ee*.

5.3.2 Kinetic resolution

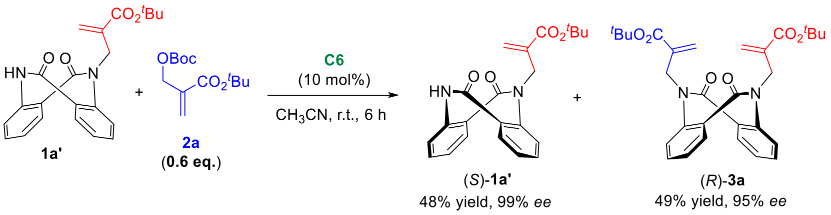

(±)-**1a′** (0.10 mmol) and the **C6** (10 mol%) was dissolved in CH_3CN and MBH carbonic esters **2a** (0.06 mmol) was added at room temperature. The reaction mixture was stirred for 6 h at room temperature. The solvent was removed in vacuo and the crude product was separated by flash column chromatography on silica gel (petroleum ether/ethyl acetate = 2:1) to afford (*R*)-**3a** (a white solid) in 49% yield with 95% *ee* and recovered (*S*)-**1a′** (a white solid) in 48% yield with 99% *ee*.

6. Characterization of racemic compounds (±)-1

The characterization of **1a, 1k-1o, 1q-1t, 1w-1y** can be obtained from reference[1] [3] [4], **1z-1aa′** can be obtained from reference[4].

(±)-4,10-difluorodibenzo[*b,f*][1,5]diazocine-6,12(5*H*,11*H*)-dione: **1g**

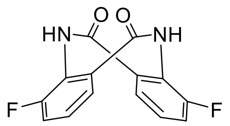

A white solid; 252 mg; isolated yield = 92%. M.p.: 234.3 – 235.6 °C; ^{1}H NMR (400 MHz, DMSO) δ 10.27 (s, 2H), 7.43 – 7.27 (m, 4H), 7.24 – 7.14 (m, 2H); ^{13}C NMR (100 MHz, DMSO) δ 168.2, 156.2 (*J* = 250 Hz), 136.1, 130.3, 123.9, 123.3, 118.0 (*J* = 20 Hz); ^{19}F NMR (376 MHz,) δ -240.03. HRMS (ESI) m/z calcd for $C_{14}H_{8}F_{2}N_{2}O_{2}$ [M+Na]$^{+}$= 297.0447, found = 297.0457.

(±)-4,10-dichlorodibenzo[*b,f*][1,5]diazocine-6,12(5*H*,11*H*)-dione: **1h**

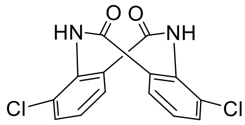

A white solid; 290 mg; isolated yield = 95%. M.p.: 215.5 – 216.9 °C; ^{1}H NMR (400 MHz, DMSO) δ 10.32 (s, 2H), 7.60 – 7.44 (m, 2H), 7.36 – 7.19 (m, 4H); ^{13}C NMR (100 MHz, DMSO) δ 168.2 136.4, 132.3, 131.5, 131.0, 130.1, 126.4. HRMS (ESI) m/z calcd for $C_{14}H_{8}Cl_{2}N_{2}O_{2}$ [M+Na]$^{+}$= 328.9856, found = 328.9854.

(±)-3,9-dimethyldibenzo[*b,f*][1,5]diazocine-6,12(5*H*,11*H*)-dione: **1i**

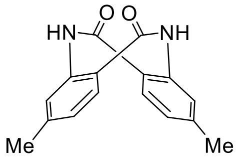

A white solid; 261 mg; isolated yield = 98%. M.p.: >300 °C; ^{1}H NMR (400 MHz, DMSO) δ 10.08 (s, 2H), 7.19 (d, *J* = 7.8 Hz, 2H), 7.05 (d, *J* = 7.8 Hz, 2H), 6.86 (s, 2H), 2.23 (s, 6H); ^{13}C NMR (100 MHz, DMSO) δ 169.9, 140.9, 135.2, 131.2, 128.6, 128.3, 126.3, 21.0. HRMS (ESI) m/z calcd for $C_{16}H_{14}N_{2}O_{2}$[M+Na]$^{+}$= 289.0948, found = 289.0959.

(±)-3,9-dimethoxydibenzo[b,f][1,5]diazocine-6,12(5H,11H)-dione: **1j**

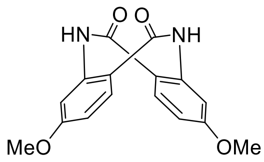

A pale yellow solid; 268 mg; isolated yield = 90%. M.p.: >300 °C; ^{1}H NMR (400 MHz, DMSO) δ 9.98 (s, 2H), 7.26 (d, *J* = 8.6 Hz, 2H), 6.90 – 6.76 (m, 2H), 6.58 (d, *J* = 1.6 Hz, 2H), 3.72 (s, 6H); ^{13}C NMR (100 MHz, DMSO) δ 169.7, 161.0, 137.0, 130.3, 126.3,

113.4, 111.0, 55.9. HRMS (ESI) m/z calcd for $C_{16}H_{14}N_2O_4$ $[M+Na]^+$= 321.0846, found = 321.0856.

(±)-3,9-difluorodibenzo[b,f][1,5]diazocine-6,12(5H,11H)-dione: **1l**

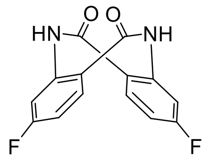

A white solid; 250 mg; isolated yield = 90%. M.p.: 262.0 – 263.6 °C; A white solid; isolated yield = 93%. ^{1}H NMR (400 MHz, DMSO) δ 10.33 (s, 2H), 7.46 – 7.30 (m, 2H), 7.23 – 7.10 (m, 2H), 7.03 – 6.83 (m, 2H); ^{13}C NMR (100 MHz, DMSO) δ 168.8, 162.9 (J = 250 Hz), 137.2, 137.1, 131.1, 131.0, 130.3, 115.2 (J = 20 Hz), 113.2, 113.0; ^{19}F NMR (376 MHz, DMSO) δ -109.63. HRMS (ESI) m/z calcd for $C_{14}H_8F_2N_2O_2$ $[M+H]^+$= 275.0627, found = 275.0639.

(±)-2,8-dimethoxydibenzo[b,f][1,5]diazocine-6,12(5H,11H)-dione: **1p**

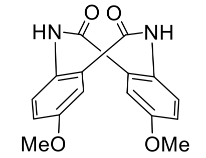

A greyish green solid; 286 mg; isolated yield = 96%. M.p.: 262.0 – 263.6 °C;^{1}H NMR (400 MHz, DMSO) δ 9.95 (s, 2H), 6.99 (d, J = 8.7 Hz, 2H), 6.93 – 6.87 (m, 2H), 6.79 (d, J = 2.8 Hz, 2H), 3.70 (s, 6H); ^{13}C NMR (100 MHz, DMSO) δ 169.6, 158.3, 135.3, 127.9, 127.9, 116.6, 112.7, 55.9. HRMS (ESI) m/z calcd for $C_{16}H_{14}N_2O_4$ $[M+Na]^+$= 321.0846, found = 321.0857.

(±)-2,8-diphenyldibenzo[b,f][1,5]diazocine-6,12(5H,11H)-dione: **1u**

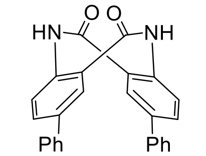

A white solid; 347 mg; isolated yield = 89%. M.p.: 263.1 – 264.9 °C;^{1}H NMR (400 MHz, DMSO) δ 10.34 (s, 2H), 7.71 – 7.60 (m, 8H), 7.47 – 7.40 (m, 4H), 7.38 – 7.32 (m, 2H), 7.23 – 7.16 (m, 2H); ^{13}C NMR (100 MHz, DMSO) δ 169.6, 139.4, 138.7, 134.5, 129.5, 129.2, 128.4, 127.0, 126.9, 126.6. HRMS (ESI) m/z calcd for $C_{26}H_{18}N_2O_2$ $[M+Na]^+$= 413.1261, found = 413.1273.

(±)-1,7-difluorodibenzo[b,f][1,5]diazocine-6,12(5H,11H)-dione: **1v**

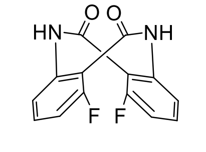

A white solid; 233 mg; isolated yield = 85%. M.p.: 279.7 – 281.9 °C; ^{1}H NMR (400 MHz, DMSO) δ 10.77 (s, 2H), 7.52 – 7.32 (m, 2H), 7.14 (t, *J* = 8.8 Hz, 2H), 7.02 (d, *J* = 8.0 Hz, 2H); ^{13}C NMR (100 MHz, DMSO) δ 164.39, 158.5 (*J* = 240 Hz), 136.8, 136.7, 132.1, 132.0, 122.1 (*J* = 20 Hz), 121.9, 121.8, 115.1 (*J* = 10 Hz); ^{19}F NMR (376 MHz, DMSO) δ -114.99. HRMS (ESI) m/z calcd for $C_{14}H_{8}F_{2}N_{2}O_{2}$ $[M+Na]^{+}$= 297.0447, found = 297.0457.

7. Characterization of products

(*S*)-Di-*tert*-butyl 2,2'-((6,12-dioxodibenzo[*b*,*f*][1,5]diazocine-5,11(6*H*,12*H*)-diyl)bis(methylene))diacrylate: **3a**

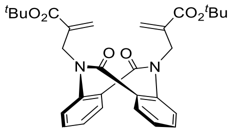

A colorless oil; 49.2 mg; isolated yield = 95%. $[A]_{D}^{20}$ = -16.06 (*c* 0.33, CH_2Cl_2); HPLC (IA column, *i*-propanol/*n*-hexane = 30/70, flow rate 1.0 mL/min, λ = 254 nm), t_1 = 7.53 min (minor), t_2 = 12.54 min (major), *ee* = 98%; ^{1}H NMR (400 MHz, DMSO) δ 7.36 – 7.30 (m, 4H), 7.28 – 7.13 (m, 4H), 6.12 (s, 2H), 5.74 (s, 2H), 4.98 (d, *J* = 15.8 Hz, 2H), 4.37 (d, *J* = 15.8 Hz, 2H), 1.37 (s, 18H); ^{13}C NMR (100 MHz, DMSO) δ 167.8, 164.7, 139.1, 136.5, 135.2, 131.1, 128.5, 127.9, 127.8, 125.9, 81.1, 48.9, 28.0. HRMS (ESI) m/z calcd for $C_{30}H_{34}N_{2}O_{6}$ $[M+Na]^{+}$= 541.2310, found = 541.2313.

(*S*)-Dimethyl 2,2'-((6,12-dioxodibenzo[*b*,*f*][1,5]diazocine-5,11(6*H*,12*H*)-diyl)bis(methylene))diacrylate: **3b**

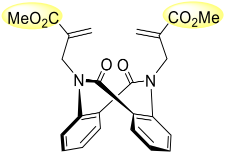

A white solid; 39.1 mg; isolated yield = 90%. M.p.: 106.3 – 106.7 °C; $[\alpha]_{D}^{20}$ = -78.80 (*c* 0.25, CH_2Cl_2); HPLC (IB column, *i*-propanol/*n*-hexane = 30/70, flow rate 1.0 mL/min, λ = 254 nm), t_1 = 10.52 min (major), t_2 = 13.51 min (minor), *ee* = 97%; ^{1}H NMR (400 MHz, $CDCl_3$) δ 7.31 – 7.24 (m, 4H), 7.22 – 7.15 (m, 2H), 7.09 (d, *J* = 7.9 Hz, 2H), 6.37 (s, 2H), 5.88 (s, 2H), 5.13 (d, *J* = 15.3 Hz, 2H), 4.44 (d, *J* = 15.3 Hz, 2H), 3.69 (s, 6H); ^{13}C NMR (100 MHz, $CDCl_3$) δ 168.1, 166.0, 139.1, 135.2, 134.6, 130.6, 129.1, 128.3, 127.8, 125.3, 52.1, 49.4. HRMS (ESI) m/z calcd for $C_{24}H_{22}N_{2}O_{6}$ $[M+Na]^{+}$= 457.1371, found = 457.1380.

(*S*)-Diethyl 2,2'-((6,12-dioxodibenzo[*b*,*f*][1,5]diazocine-5,11(6*H*,12*H*)-diyl)bis(methylene))diacrylate: **3c**

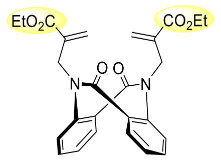

A colorless oil; 43.0 mg; isolated yield = 93%. $[A]_D^{20}$ = -11.75 (*c* 0.40, CH_2Cl_2); HPLC (IA column, *i*-propanol/*n*-hexane = 30/70, flow rate 1.0 mL/min, λ = 254 nm), t_1 = 15.03 min (minor), t_2 = 17.72 min (major), *ee* = 96%; ^1H NMR (400 MHz, $CDCl_3$) δ 7.31 – 7.24 (m, 4H), 7.22 – 7.16 (m, 2H), 7.11 (d, *J* = 8.0 Hz, 2H), 6.39 (s, 2H), 5.88 (s, 2H), 5.07 (d, *J* = 15.4 Hz, 2H), 4.50 (d, *J* = 15.4 Hz, 2H), 4.23 – 4.09 (m, 4H), 1.19 (t, *J* = 7.1 Hz, 6H); ^{13}C NMR (100 MHz, $CDCl_3$) δ 168.1, 165.6, 139.2, 135.1, 134.8, 130.6, 128.6, 128.2, 127.7, 125.2, 61.0, 49.4, 14.0. HRMS (ESI) m/z calcd for $C_{26}H_{26}N_2O_6$ $[M+H]^+$= 463.1864, found = 463.1860.

(*S*)-Dibutyl 2,2'-((6,12-dioxodibenzo[*b*,*f*][1,5]diazocine-5,11(6*H*,12*H*)-diyl)bis(methylene))diacrylate: **3d**

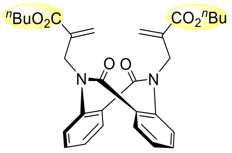

A colorless oil; 47.1 mg; isolated yield = 91%. $[A]_D^{20}$ = +64.80 (*c* 0.75, CH_2Cl_2); HPLC (IC column, *i*-propanol/*n*-hexane = 30/70, flow rate 1.0 mL/min, λ = 254 nm), t_1 = 18.89 min (major), t_2 = 25.88 min (minor), *ee* = 92%; ^1H NMR (400 MHz, $CDCl_3$) δ 7.32 – 7.23 (m, 4H), 7.23 – 7.15 (m, 2H), 7.11 (d, *J* = 7.9 Hz, 2H), 6.39 (s, 2H), 5.89 (s, 2H), 5.07 (d, *J* = 15.5 Hz, 2H), 4.51 (d, *J* = 15.5 Hz, 2H), 4.21 – 4.03 (m, 4H), 1.61 – 1.49 (m, 4H), 1.38 – 1.28 (m, 4H), 0.90 (t, *J* = 7.4 Hz, 6H); ^{13}C NMR (100 MHz, $CDCl_3$) δ 168.1, 165.6, 139.2, 135.1, 134.8, 130.6, 128.5, 128.2, 127.7, 125.2, 64.8, 49.4, 30.4, 19.1, 13.7. HRMS (ESI) m/z calcd for $C_{30}H_{34}N_2O_6$ $[M+Na]^+$= 541.2310, found = 541.2324.

(*S*)-Diisobutyl 2,2'-((6,12-dioxodibenzo[*b*,*f*][1,5]diazocine-5,11(6*H*,12*H*)-diyl)bis(methylene))diacrylate: **3e**

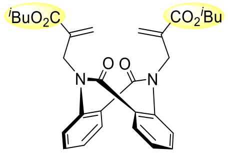

A colorless oil; 46.6 mg; isolated yield = 90%. $[A]_D^{20}$ = -152.84 (*c* 0.60, CH_2Cl_2); HPLC (IF column, *i*-propanol/*n*-hexane = 30/70, flow rate 1.0 mL/min, λ = 254 nm), t_1 = 23.73 min (major), t_2 = 56.42 min (minor), *ee* = 92%; ^1H NMR (400 MHz, $CDCl_3$) δ 7.32 – 7.23 (m, 4H), 7.22 – 7.15 (m, 2H), 7.11 (d, *J* = 7.9 Hz, 2H), 6.41 (s, 2H), 5.89 (s, 2H), 5.06 (d, *J* = 15.6 Hz, 2H), 4.53 (d, *J* = 15.6 Hz, 2H), 4.01 – 3.78 (m, 4H), 1.94 – 1.84 (m, 2H), 0.90 (dd, *J* = 6.7, 2.9 Hz, 12H); ^{13}C NMR (100 MHz, $CDCl_3$) δ 168.2, 165.6, 139.2, 135.1, 134.8, 130.7, 128.3, 127.8, 125.2, 71.1, 49.5, 27.6, 19.2, 19.1. HRMS (ESI) m/z calcd for $C_{30}H_{34}N_2O_6$ $[M+Na]^+$= 541.2310, found = 541.2308.

(*S*)-Dibenzyl 2,2'-((6,12-dioxodibenzo[*b*,*f*][1,5]diazocine-5,11(6*H*,12*H*)-diyl)bis(methylene))diacrylate: **3f**

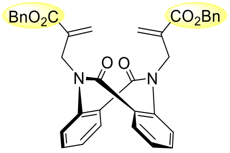

A colorless oil; 55.1 mg; isolated yield = 94%. $[A]_D^{20}$ = +62.24 (*c* 0.85, CH_2Cl_2); HPLC (IF column, *i*-propanol/*n*-hexane = 30/70, flow rate 1.0 mL/min, λ = 254 nm), t_1 = 36.92 min (major), t_2 = 46.98 min (minor), *ee* = 91%; ^{1}H NMR (400 MHz, $CDCl_3$) δ 7.34 – 7.23 (m, 12H), 7.23 – 7.11 (m, 4H), 7.06 (d, *J* = 7.9 Hz, 2H), 6.41 (s, 2H), 5.91 (s, 2H), 5.21 – 5.06 (m, 6H), 4.48 (d, *J* = 15.4 Hz, 2H); ^{13}C NMR (100 MHz, $CDCl_3$) δ 168.1, 165.4, 139.2, 135.8, 135.1, 134.6, 130.7, 129.4, 128.5, 128.3, 128.2, 128.1, 127.7, 125.3, 66.8, 49.5. HRMS (ESI) m/z calcd for $C_{36}H_{30}N_2O_6$ $[M+Na]^+$= 609.1997, found = 609.2006.

(*R*)-Di-*tert*-butyl 2,2'-((4,10-difluoro-6,12-dioxodibenzo[*b*,*f*][1,5]diazocine-5,11(6*H*,12*H*)-diyl)bis(methylene))diacrylate: **3g**

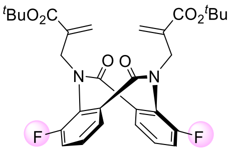

A white solid; 49.9 mg; isolated yield = 90%; M.p.: 105.5 – 106.7 °C; $[\alpha]_D^{20}$ = -57.67 (*c* 0.30, CH_2Cl_2); HPLC (IF column, *i*-propanol/*n*-hexane = 30/70, flow rate 1.0 mL/min, λ = 254 nm), t_1 = 8.14 min (minor), t_2 = 9.48 min (major), *ee* = 92%; ^{1}H NMR (400 MHz, $CDCl_3$) δ 7.24 – 7.13 (m, 2H), 7.10 (d, *J* = 7.5 Hz, 2H), 7.06 – 6.92 (m, 2H), 6.29 (s, 2H), 5.88 (s, 2H), 5.14 (d, *J* = 14.9 Hz, 2H), 4.23 (d, *J* = 14.9 Hz, 2H), 1.38 (s, 18H); ^{13}C NMR (100 MHz, $CDCl_3$) δ 166.6, 164.7, 156.9 (*J* = 251 Hz), 137.1, 135.8, 130.3, 130.2(*J* = 8 Hz), 129.7, 126.9, 126.7, 122.9, 122.8, 117.7 (*J* = 20 Hz), 81.1, 47.7, 47.6, 27.89. ^{19}F NMR (376 MHz, $CDCl_3$) δ -118.19. HRMS (ESI) m/z calcd for $C_{30}H_{32}F_2N_2O_6$ $[M+Na]^+$= 577.2121, found = 577.2125.

(*S*)-Di-*tert*-butyl 2,2'-((4,10-dichloro-6,12-dioxodibenzo[*b*,*f*][1,5]diazocine-5,11(6*H*,12*H*)-diyl)bis(methylene))diacrylate: **3h**

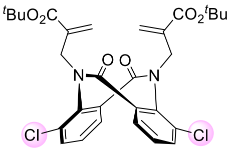

A white solid; 53.9 mg; isolated yield = 92%. M.p.: 124.0 – 126.3 °C; $[\alpha]_D^{20}$ = +16.17 (*c* 0.17, CH_2Cl_2); HPLC (IC column, *i*-propanol/*n*-hexane = 30/70, flow rate 1.0 mL/min, λ = 254 nm), t_1 = 5.64 min (minor), t_2 = 6.09 min (major), *ee* = 92%; ^{1}H NMR (400 MHz, $CDCl_3$) δ 7.33 – 7.27 (m, 2H), 7.26 – 7.21 (m, 2H), 7.19 – 7.12 (m, 2H), 6.32 (d, *J* = 0.8 Hz, 2H), 5.98 (s, 2H), 5.15 (d, *J* = 14.5 Hz, 2H), 4.18 (d, *J* = 14.5 Hz, 2H), 1.34 (s, 18H); ^{13}C NMR (100 MHz, $CDCl_3$) δ 166.6, 164.8, 137.6, 136.5, 135.6, 131.9, 131.5, 131.0, 129.8, 125.2, 80.9, 47.0, 27.8. HRMS (ESI) m/z calcd for

$C_{30}H_{32}Cl_2N_2O_6$ $[M+Na]^+$= 609.1530, found = 609.1531.

(*R*)-Di-*tert*-butyl 2,2'-((3,9-dimethyl-6,12-dioxodibenzo[*b*,*f*][1,5]diazocine-5,11(6*H*,12*H*)-diyl)bis(methylene))diacrylate: **3i**

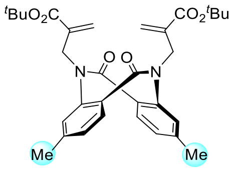

A colorless oil; 54.1 mg; isolated yield = 99%. $[A]_D^{20}$ = -7.71 (*c* 0.12, CH_2Cl_2); HPLC (IF column, *i*-propanol/*n*-hexane = 30/70, flow rate 1.0 mL/min, λ = 254 nm), t_1 = 11.49 min (major), t_2 = 13.43 min (minor), *ee* = 99%; ^{1}H NMR (400 MHz, $CDCl_3$) δ 7.17 (d, *J* = 7.8 Hz, 2H), 6.99 (d, *J* = 7.8 Hz, 2H), 6.89 (s, 2H), 6.31 (s, 2H), 5.78 (s, 2H), 4.91 (d, *J* = 16.1 Hz, 2H), 4.53 (d, *J* = 16.1 Hz, 2H), 2.23 (s, 6H), 1.43 (s, 18H); ^{13}C NMR (100 MHz, $CDCl_3$) δ 168.5, 164.9, 141.0, 139.3, 136.1, 132.3, 129.0, 127.6, 126.8, 125.5, 81.2, 49.5, 28.0, 21.3. HRMS (ESI) m/z calcd for $C_{32}H_{38}N_2O_6$ $[M+Na]^+$= 569.2623, found = 569.2631.

(*R*)-Di-*tert*-butyl 2,2'-((3,9-dimethoxy-6,12-dioxodibenzo[*b*,*f*][1,5]diazocine-5,11(6*H*,12*H*)-diyl)bis(methylene))diacrylate: **3j**

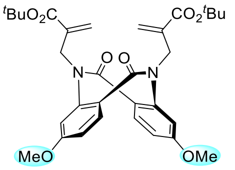

A colorless oil; 55.5 mg; isolated yield = 96%. $[A]_D^{20}$ = -11.99 (*c* 0.17, CH_2Cl_2); HPLC (IF column, *i*-propanol/*n*-hexane = 30/70, flow rate 1.0 mL/min, λ = 254 nm), t_1 = 17.44 min (minor), t_2 = 20.31 min (major), *ee* = >99%; ^{1}H NMR (400 MHz, $CDCl_3$) δ 7.22 (d, *J* = 8.6 Hz, 2H), 6.79 – 6.65 (m, 2H), 6.58 (d, *J* = 2.4 Hz, 2H), 6.29 (s, 2H), 5.75 (s, 2H), 4.92 (d, *J* = 15.9 Hz, 2H), 4.49 (d, *J* = 15.9 Hz, 2H), 3.70 (s, 6H), 1.42 (s, 18H); ^{13}C NMR (100 MHz, $CDCl_3$) δ 168.2, 164.9, 161.0, 140.8, 136.2, 129.1, 127.6, 127.0, 113.6, 110.9, 81.2, 55.4, 49.4, 28.0. HRMS (ESI) m/z calcd for $C_{32}H_{38}N_2O_8$ $[M+Na]^+$= 601.2521, found = 601.2529.

(*S*)-Di-tert-butyl 2,2'-((3,9-di-tert-butyl-6,12-dioxodibenzo[*b*,*f*][1,5]diazocine-5,11(6*H*,12*H*)-diyl)bis(methylene))diacrylate: **3k**

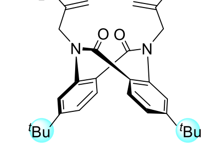

A white solid; 58.5 mg; isolated yield = 93%. $[A]_D^{20}$ = -47.50 (*c* 0.20, CH_2Cl_2); HPLC (IK column, *i*-propanol/*n*-hexane = 30/70, flow rate 1.0 mL/min, λ = 254 nm), t_1 = 3.63 min (minor), t_2 = 4.67 min (major), *ee* = 90%; ^{1}H NMR (400 MHz, $CDCl_3$) δ 7.21 (s,

4H), 7.06 (s, 2H), 6.31 (s, 2H), 5.76 (s, 2H), 4.93 (d, J = 15.9 Hz, 2H), 4.60 (d, J = 15.9 Hz, 2H), 1.44 (s, 18H), 1.20 (s, 18H), ^{13}C NMR (100 MHz, $CDCl_3$) δ 168.6, 164.9, 154.1, 138.9, 136.3, 132.2, 127.5, 127.2, 125.4, 121.0, 8.13, 49.4, 34.8, 30.96, 28.02. HRMS (ESI) m/z calcd for $C_{38}H_{50}N_2O_6$ $[M+Na]^+$= 653.3567, found = 653.3569.

(*S*)-Di-*tert*-butyl 2,2'-((3,9-difluoro-6,12-dioxodibenzo[*b*,*f*][1,5]diazocine-5,11(6*H*,12*H*)-diyl)bis(methylene))diacrylate: **3l**

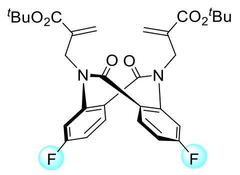

A colorless oil; 52.6 mg; isolated yield = 95%. $[A]_D^{20}$ = -189.21 (*c* 0.25, CH_2Cl_2); HPLC (IF column, *i*-propanol/*n*-hexane = 30/70, flow rate 1.0 mL/min, λ = 254 nm), t_1 = 7.72 min (minor), t_2 = 10.70 min (major), *ee* = 96%; ^{1}H NMR (400 MHz, $CDCl_3$) δ 7.32 – 7.25 (m, 2H), 6.99 – 6.77 (m, 4H), 6.30 (s, 2H), 5.76 (s, 2H), 4.94 (d, J = 15.7 Hz, 2H), 4.46 (d, J = 15.7 Hz, 2H), 1.42 (s, 18H); ^{13}C NMR (100 MHz, $CDCl_3$) δ 167.0, 164.7, 163.1 (J = 251 Hz), 140.8, 140.7, 135.8, 131.2, 130.0 (J = 8 Hz), 127.8, 116.0 (J = 22 Hz), 112.9, 112.7, 81.4, 49.2, 28.0; ^{19}F NMR (376 MHz, $CDCl_3$) δ -107.72. HRMS (ESI) m/z calcd for $C_{30}H_{32}F_2N_2O_6$ $[M+Na]^+$= 577.2121, found = 577.2123.

(*S*)-Di-*tert*-butyl 2,2'-((3,9-dichloro-6,12-dioxodibenzo[*b*,*f*][1,5]diazocine-5,11(6*H*,12*H*)-diyl)bis(methylene))diacrylate: **3m**

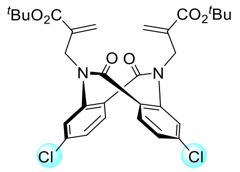

A white solid; 53.9 mg; isolated yield = 92%. M.p.: 110.0 – 112.6 °C; $[\alpha]_D^{20}$ = -104.98 (*c* 0.58, CH_2Cl_2); HPLC (IF column, *i*-propanol/*n*-hexane = 30/70, flow rate 1.0 mL/min, λ = 254 nm), t_1 = 8.77 min (minor), t_2 = 10.86 min (major), *ee* = 91%; ^{1}H NMR (400 MHz, $CDCl_3$) δ 7.25 – 7.15 (m, 4H), 7.15 – 7.06 (m, 2H), 6.30 (s, 2H), 5.77 (s, 2H), 4.94 (d, J = 15.6 Hz, 2H), 4.45 (d, J = 15.6 Hz, 2H), 1.42 (s, 18H); ^{13}C NMR (100 MHz, $CDCl_3$) δ 166.9, 164.6, 140.0, 136.3, 135.7, 133.3, 129.1, 128.9, 128.1, 125.7, 81.4, 49.2, 28.0. HRMS (ESI) m/z calcd for $C_{30}H_{32}Cl_2N_2O_6$ $[M+Na]^+$= 609.1530, found = 609.1533.

(*R*)-Di-*tert*-butyl 2,2'-((6,12-dioxo-3,9-bis(trifluoromethyl)dibenzo[*b*,*f*][1,5]diazocine-5,11(6*H*,12*H*)-diyl)bis(methylene))diacrylate: **3n**

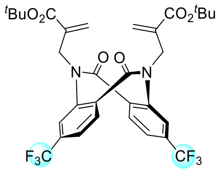

A yellow solid; 42.5 mg; isolated yield = 65%. M.p.: 197.1 – 198.6 °C; $[\alpha]_D^{20}$ = +56.75 (*c* 0.15, CH_2Cl_2); HPLC (IC column, *i*-propanol/*n*-hexane = 30/70, flow rate 1.0 mL/min, λ = 254 nm), t_1 = 3.83 min (major), t_2 = 4.38 min (minor), *ee* = 88%; ^{1}H NMR (400 MHz, $CDCl_3$) δ 7.49 (s, 2H), 7.47 – 7.34 (m, 4H), 6.33 (s, 2H), 5.83 (s, 2H), 5.03 (d, *J* = 15.3 Hz, 2H), 4.52 (d, *J* = 15.3 Hz, 2H), 1.42 (s, 18H); ^{13}C NMR (100 MHz, $CDCl_3$) δ 164.5, 139.2, 138.1, 135.6, 133.2, 132.8, 128.8, 125.6, 125.5, 122.8 (*J* = 271 Hz), 122.7, 49.2, 27.9; ^{19}F NMR (376 MHz, $CDCl_3$) δ -63.00. HRMS (ESI) m/z calcd for $C_{32}H_{32}F_6N_2O_6$ $[M+Na]^+$= 677.2057, found = 677.2061.

(*S*)-Di-*tert*-butyl 2,2'-((2,8-dimethyl-6,12-dioxodibenzo[*b*,*f*][1,5]diazocine-5,11(6*H*,12*H*)-diyl)bis(methylene))diacrylate: **3o**

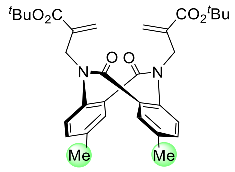

A white solid; 51.9 mg; isolated yield = 95%. M.p.: 89.5 – 91.2 °C; $[\alpha]_D^{20}$ = +75.63 (*c* 0.08, CH_2Cl_2); HPLC (IC column, *i*-propanol/*n*-hexane = 30/70, flow rate 1.0 mL/min, λ = 254 nm), t_1 = 12.02 min (minor), t_2 = 15.76 min (major), *ee* = 93%; ^{1}H NMR (400 MHz, $CDCl_3$) δ 7.18 – 6.92 (m, 6H), 6.32 (s, 2H), 5.76 (s, 2H), 4.88 (d, *J* = 16.1 Hz, 2H), 4.55 (d, *J* = 16.1 Hz, 2H), 2.24 (s, 6H), 1.44 (s, 18H); ^{13}C NMR (100 MHz, $CDCl_3$) δ 168.5, 164.9, 138.3, 136.7, 136.2, 134.9, 131.3, 128.1, 126.8, 125.0, 81.2, 49.4, 28.0, 20.9. HRMS (ESI) m/z calcd for $C_{32}H_{38}N_2O_6$ $[M+Na]^+$= 569.2623, found = 569.2624.

(*R*)-Di-*tert*-butyl 2,2'-((2,8-dimethoxy-6,12-dioxodibenzo[*b*,*f*][1,5]diazocine-5,11(6*H*,12*H*)-diyl)bis(methylene))diacrylate: **3p**

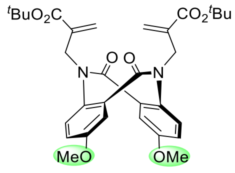

A colorless oil; 53.8 mg; isolated yield = 93%. $[A]_D^{20}$ = -75.00 (*c* 0.10, CH_2Cl_2); HPLC (IC column, *i*-propanol/*n*-hexane = 30/70, flow rate 1.0 mL/min, λ = 254 nm), t_1 = 16.81 min (major), t_2 = 23.85 min (minor), *ee* = >99%; ^{1}H NMR (400 MHz, $CDCl_3$) δ 7.03 – 6.95 (m, 2H), 6.81 – 6.68 (m, 4H), 6.31 (s, 2H), 5.75 (s, 2H), 4.86 (d, *J* = 15.8 Hz, 2H), 4.52 (d, *J* = 15.8 Hz, 2H), 3.72 (s, 6H), 1.44 (s, 18H); ^{13}C NMR (100 MHz, $CDCl_3$) δ 168.2, 164.9, 158.9, 136.2, 136.1, 131.8, 127.2, 126.8, 116.6, 111.7, 81.2, 55.5, 49.4, 28.0. HRMS (ESI) m/z calcd for $C_{32}H_{38}N_2O_8$ $[M+Na]^+$= 601.2521, found = 601.2524.

(*R*)-Di-*tert*-butyl 2,2'-((2,8-difluoro-6,12-dioxodibenzo[*b*,*f*][1,5]diazocine-5,11(6*H*,12*H*)-diyl)bis(methylene))diacrylate: **3q**

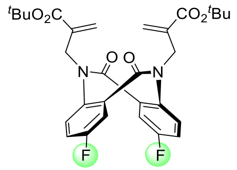

A white solid; 53.7 mg; isolated yield = 97%. M.p.: 123.8 – 124.9 °C; $[\alpha]_D^{20}$ = -98.00 (*c* 0.05, CH_2Cl_2); HPLC (IC column, *i*-propanol/*n*-hexane = 30/70, flow rate 1.0 mL/min, λ = 254 nm), t_1 = 6.70 min (major), t_2 = 7.74 min (minor), *ee* = 91%; ^{1}H NMR (400 MHz, $CDCl_3$) δ 7.13 – 7.07 (m, 2H), 7.02 – 6.92 (m, 4H), 6.31 (s, 2H), 5.76 (s, 2H), 4.89 (d, *J* = 15.5 Hz, 2H), 4.50 (d, *J* = 15.5 Hz, 2H), 1.43 (s, 18H); ^{13}C NMR (100 MHz, $CDCl_3$) δ 166.6, 164.7, 162.7, 161.5 (*J* = 250 Hz), 136.8, 135.9, 134.9, 134.8, 128.0, 127.7 (*J* = 8 Hz), 117.8 (*J* = 22 Hz), 114.8, 114.5, 81.4, 49.3, 28.0. ^{19}F NMR (376 MHz, $CDCl_3$) δ -111.23. HRMS (ESI) m/z calcd for $C_{30}H_{32}F_2N_2O_6$ $[M+Na]^+$= 577.2121, found = 577.2130.

<u>(*R*)-Di-*tert*-butyl 2,2'-((2,8-dichloro-6,12-dioxodibenzo[*b*,*f*][1,5]diazocine-5,11(6*H*,12*H*)-diyl)bis(methylene))diacrylate: **3r**</u>

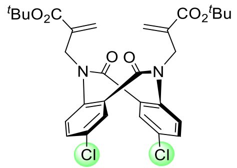

A white solid; 56.2 mg; isolated yield = 96%. M.p.: 109.5 – 110.9 °C; $[\alpha]_D^{20}$ = -12.90 (*c* 0.17, CH_2Cl_2); HPLC (IC column, *i*-propanol/*n*-hexane = 30/70, flow rate 1.0 mL/min, λ = 254 nm), t_1 = 6.54 min (major), t_2 = 8.55 min (minor), *ee* = 94%; ^{1}H NMR (400 MHz, $CDCl_3$) δ 7.27 (s, 2H), 7.26 – 7.23 (m, 2H), 7.13 – 7.03 (m, 2H), 6.31 (s, 2H), 5.76 (s, 2H), 4.92 (d, *J* = 15.6 Hz, 2H), 4.49 (d, *J* = 15.6 Hz, 2H), 1.44 (s, 18H); ^{13}C NMR (100 MHz, $CDCl_3$) δ 166.4, 164.7, 137.2, 136.3, 135.8, 134.3, 130.9, 128.0, 127.8, 127.0, 81.4, 49.2, 28.0. HRMS (ESI) m/z calcd for $C_{30}H_{32}Cl_2N_2O_6$ $[M+Na]^+$= 609.1530, found = 609.1539.

<u>(*R*)-Di-*tert*-butyl 2,2'-((2,8-dibromo-6,12-dioxodibenzo[*b*,*f*][1,5]diazocine-5,11(6*H*,12*H*)-diyl)bis(methylene))diacrylate: **3s**</u>

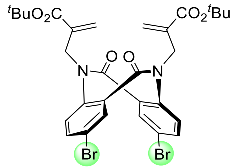

A pale yellow solid; 60.7 mg; isolated yield = 90%. M.p.: 75.9 – 77.7 °C; $[\alpha]_D^{20}$ = +6.00 (*c* 0.08, CH_2Cl_2); HPLC (IC column, *i*-propanol/*n*-hexane = 30/70, flow rate 1.0 mL/min, λ = 254 nm), t_1 = 6.32 min (major), t_2 = 9.55 min (minor), *ee* = 91%; ^{1}H NMR (400 MHz, $CDCl_3$) δ 7.53 – 7.29 (m, 4H), 7.09 – 6.95 (m, 2H), 6.31 (s, 2H), 5.76 (s, 2H), 4.92 (d, *J* = 15.6 Hz, 2H), 4.48 (d, *J* = 15.6 Hz, 2H), 1.44 (s, 18H); ^{13}C NMR (100 MHz, $CDCl_3$) δ 166.2, 164.7, 137.7, 136.5, 135.8, 133.9, 130.7, 128.0, 127.2, 122.2,

81.5, 49.2, 28.0. HRMS (ESI) m/z calcd for $C_{30}H_{32}Br_2N_2O_6$ $[M+Na]^+$= 699.0499, found = 699.0525.

<u>(*S*)-Di-*tert*-butyl 2,2'-((2,8-diiodo-6,12-dioxodibenzo[*b,f*][1,5]diazocine-5,11(6*H*,12*H*)-diyl)bis(methylene))diacrylate: **3t**</u>

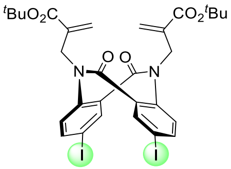

A pale yellow solid; 72.4 mg; isolated yield = 94%. M.p.: 91.2 – 92.9 °C; $[\alpha]_D^{20}$ = +19.25 (*c* 0.20, CH_2Cl_2); HPLC (IF column, *i*-propanol/*n*-hexane = 30/70, flow rate 1.0 mL/min, λ = 254 nm), t_1 = 7.31 min (major), t_2 = 27.64 min (minor), *ee* = 97%; ^{1}H NMR (400 MHz, $CDCl_3$) δ 7.66 – 7.55 (m, 4H), 6.88 (d, *J* = 8.3 Hz, 2H), 6.31 (s, 2H), 5.75 (s, 2H), 4.92 (d, *J* = 15.6 Hz, 2H), 4.47 (d, *J* = 15.7 Hz, 2H), 1.44 (s, 18H); ^{13}C NMR (100 MHz, $CDCl_3$) δ 166.0, 164.7, 139.9, 138.4, 136.6, 136.5, 135.7, 127.9, 127.2, 93.4, 81.4, 49.2, 28.0. HRMS (ESI) m/z calcd for $C_{30}H_{32}I_2N_2O_6$ $[M+Na]^+$= 793.0242, found = 793.0241.

<u>(*S*)-Di-*tert*-butyl 2,2'-((6,12-dioxo-2,8-diphenyldibenzo[*b,f*][1,5]diazocine-5,11(6*H*,12*H*)-diyl)bis(methylene))diacrylate: **3u**</u>

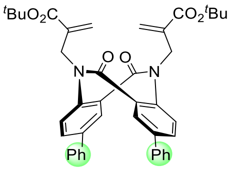

A white solid; 62.3 mg; isolated yield = 93%. M.p.: 100.0 – 101.3 °C; $[\alpha]_D^{20}$ = +16.00 (*c* 0.05, CH_2Cl_2); HPLC (IF column, *i*-propanol/*n*-hexane = 30/70, flow rate 1.0 mL/min, λ = 254 nm), t_1 = 21.86 min (minor), t_2 = 41.49 min (major), *ee* = 95%; ^{1}H NMR (400 MHz, $CDCl_3$) δ 7.53 (d, *J* = 1.9 Hz, 2H), 7.51 – 7.43 (m, 6H), 7.43 – 7.30 (m, 6H), 7.19 (d, *J* = 8.4 Hz, 2H), 6.37 (s, 2H), 5.85 (s, 2H), 4.99 (d, *J* = 15.9 Hz, 2H), 4.63 (d, *J* = 15.9 Hz, 2H), 1.45 (s, 18H); ^{13}C NMR (100 MHz, $CDCl_3$) δ 168.2, 164.9, 141.2, 138.9, 138.2, 136.1, 135.5, 129.2, 128.9, 128.1, 127.2, 126.9, 126.2, 125.8, 100.0, 81.3, 49.5, 28.0. HRMS (ESI) m/z calcd for $C_{42}H_{42}N_2O_6$ $[M+Na]^+$= 693.2936, found = 693.2949.

<u>(*R*)-Di-*tert*-butyl 2,2'-((1,7-difluoro-6,12-dioxodibenzo[*b,f*][1,5]diazocine-5,11(6*H*,12*H*)-diyl)bis(methylene))diacrylate: **3v**</u>

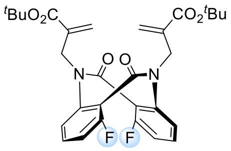

A colorless oil; 49.9 mg; isolated yield = 90%. $[A]_D^{20}$ = -29.00 (*c* 0.10, CH_2Cl_2); HPLC

(IC column, *i*-propanol/*n*-hexane = 30/70, flow rate 1.0 mL/min, λ = 254 nm), t_1 = 4.76 min (major), t_2 = 5.14 min (minor), *ee* = 92%; ^{1}H NMR (400 MHz, $CDCl_3$) δ 7.27 (s, 1H), 7.24 – 7.21 (m, 1H), 7.03 (d, *J* = 8.1 Hz, 2H), 6.95 (t, *J* = 8.4 Hz, 2H), 6.38 (s, 2H), 5.85 (s, 2H), 4.89 – 4.76 (m, 2H), 4.71 – 4.59 (m, 2H), 1.46 (s, 18H); ^{13}C NMR (100 MHz, $CDCl_3$) δ 164.7, 162.8, 158.3 (*J* = 250 Hz), 140.7, 140.7, 135.9, 131.3 (*J* = 9 Hz), 127.1, 123.3, 123.1, 120.6, 120.6, 115.8 (*J* = 21 Hz), 81.5, 49.4, 28.0. ^{19}F NMR (376 MHz, $CDCl_3$) δ -113.64. HRMS (ESI) m/z calcd for $C_{30}H_{32}F_2N_2O_6$ $[M+Na]^+$= 577.2121, found = 577.2122.

(*R*)-Di-*tert*-butyl 2,2'-((3,4,9,10-tetramethyl-6,12-dioxodibenzo[*b*,*f*][1,5]diazocine-5,11(6*H*,12*H*)-diyl)bis(methylene))diacrylate: **3w**

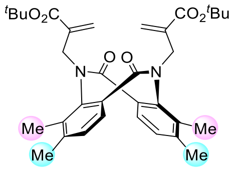

A colorless oil; 51.7 mg; isolated yield = 90%. $[A]_D^{20}$ = +56.37 (*c* 0.20, CH_2Cl_2); HPLC (IC column, *i*-propanol/*n*-hexane = 30/70, flow rate 1.0 mL/min, λ = 254 nm), t_1 = 10.75 min (major), t_2 = 14.89 min (minor), *ee* = 99%; ^{1}H NMR (400 MHz, $CDCl_3$) δ 7.03 – 6.89 (m, 4H), 6.27 (s, 2H), 5.91 (s, 2H), 5.09 (d, *J* = 14.4 Hz, 2H), 3.93 (d, *J* = 14.4 Hz, 2H), 2.11 (s, 6H), 2.00 (s, 6H), 1.33 (s, 18H); ^{13}C NMR (100 MHz, $CDCl_3$) δ 168.8, 165.0, 139.8, 138.1, 135.8, 133.9, 133.3, 130.1, 129.6, 123.4, 80.7, 47.7, 27.8, 20.4, 14.7. HRMS (ESI) m/z calcd for $C_{34}H_{42}N_2O_6$ $[M+Na]^+$= 597.2936, found = 597.2946.

(*R*)-Di-*tert*-butyl 2,2'-((2,3,8,9-tetrafluoro-6,12-dioxodibenzo[*b*,*f*][1,5]diazocine-5,11(6*H*,12*H*)-diyl)bis(methylene))diacrylate: **3x**

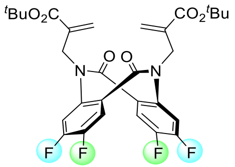

A colorless oil; 56.0 mg; isolated yield = 95%. $[A]_D^{20}$ = +125.84(*c* 0.17, CH_2Cl_2); HPLC (IC column, *i*-propanol/*n*-hexane = 30/70, flow rate 1.0 mL/min, λ = 254 nm), t_1 = 5.07 min (major), t_2 = 6.44 min (minor), *ee* = 91%; ^{1}H NMR (400 MHz, $CDCl_3$) δ 7.12 (t, *J* = 8.8 Hz, 2H), 7.04 – 6.94 (m, 2H), 6.30 (s, 2H), 5.76 (s, 2H), 4.92 (d, *J* = 15.3 Hz, 2H), 4.40 (d, *J* = 15.3 Hz, 2H), 1.44 (s, 18H); ^{13}C NMR (100 MHz, $CDCl_3$) δ 165.6, 164.6, 150.7 (*J* = 255 Hz), 150.1 (*J* = 254 Hz), 149.8 (*J* = 252 Hz), 149.7 (*J* = 252 Hz),135.6, 135.2 (*J* = 7 Hz), 135.1 (*J* = 7 Hz), 131.4 (*J* = 7 Hz), 128.6, 116.7 (*J* = 20 Hz), 115.3 (*J* = 19 Hz), 81.6, 49.2, 28.0; ^{19}F NMR (376 MHz, $CDCl_3$) δ -130.75, -130.81, -134.48, -134.54. HRMS (ESI) m/z calcd for $C_{30}H_{30}F_4N_2O_6$ $[M+Na]^+$= 613.1933, found = 613.1933.

(*S*)-Di-*tert*-butyl 2,2'-((7,15-dioxodinaphtho[2,3-*b*:2',3'-*f*][1,5]diazocine-6,14(7*H*,15*H*)-diyl)bis(methylene))diacrylate: **3y**

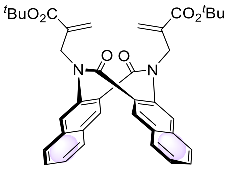

A white solid; 56.2 mg; isolated yield = 91%. M.p.: 174.2 – 175.5 °C; $[\alpha]_D^{20}$ = -87.94 (*c* 0.20, CH_2Cl_2); HPLC (IB column, *i*-propanol/*n*-hexane = 30/70, flow rate 1.0 mL/min, λ = 254 nm), t_1 = 4.84 min (minor), t_2 = 6.35 min (major), *ee* = 91%; ^{1}H NMR (400 MHz, $CDCl_3$) δ 7.82 (s, 2H), 7.76 – 7.62 (m, 4H), 7.58 (s, 2H), 7.45 – 7.36 (m, 4H), 6.39 (s, 2H), 5.91 (s, 2H), 4.97 (d, *J* = 15.8 Hz, 2H), 4.77 (d, *J* = 15.8 Hz, 2H), 1.40 (s, 18H); ^{13}C NMR (100 MHz, $CDCl_3$) δ 168.5, 165.0, 136.5, 136.4, 134.1, 133.5, 132.1, 128.0, 127.9, 127.7, 127.6, 127.4, 127.2, 124.7, 81.3, 50.3, 28.0. HRMS (ESI) m/z calcd for $C_{38}H_{38}N_2O_6$ $[M+Na]^+$= 641.2623, found = 641.2631.

(*R*)-Di-*tert*-butyl 2,2'-((2-chloro-6,12-dioxodibenzo[*b,f*][1,5]diazocine-5,11(6*H*,12*H*)-diyl)bis(methylene))diacrylate: **3z**

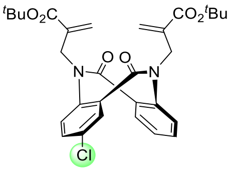

A white solid; 49.1 mg; isolated yield = 89%. M.p.: 122.2 – 123.7 °C; $[\alpha]_D^{20}$ = +112.76(*c* 0.56, CH_2Cl_2); HPLC (ID column, *i*-propanol/*n*-hexane = 30/70, flow rate 1.0 mL/min, λ = 254 nm), t_1 = 12.77 min (major), t_2 = 16.04 min (minor), *ee* = 96%; ^{1}H NMR (400 MHz, $CDCl_3$) δ 7.34 – 7.27 (m, 2H), 7.26 – 7.17 (m, 3H), 7.14 (d, *J* = 8.0 Hz, 1H), 7.06 (d, *J* = 8.6 Hz, 1H), 6.32 (s, 2H), 5.78 (d, *J* = 5.3 Hz, 2H), 4.92 (dd, *J* = 15.7, 5.2 Hz, 2H), 4.54 (dd, *J* = 15.7, 3.3 Hz, 2H), 1.44 (d, *J* = 4.3 Hz, 18H); ^{13}C NMR (100 MHz, $CDCl_3$) δ 167.9, 166.6, 164.8, 164.8, 138.8, 137.6, 136.6, 136.0, 134.9, 134.0, 130.9, 130.7, 128.6, 127.8, 127.7, 127.6, 127.5, 126.8, 125.5, 81.4, 81.3, 49.4, 49.21, 28.0. HRMS (ESI) m/z calcd for $C_{30}H_{33}ClN_2O_6$ $[M+Na]^+$= 575.1920, found = 575.1934.

(*R*)-Di-*tert*-butyl 2,2'-((2-bromo-6,12-dioxodibenzo[*b,f*][1,5]diazocine-5,11(6*H*,12*H*)-diyl)bis(methylene))diacrylate: **3aa**

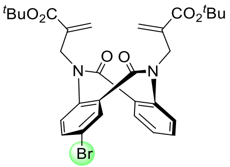

A white solid; 54.8 mg; isolated yield = 92%. M.p.: 126.0 – 128.8 °C; $[\alpha]_D^{20}$ = -34.10 (*c* 0.20, CH_2Cl_2); HPLC (IF column, *i*-propanol/*n*-hexane = 30/70, flow rate 1.0 mL/min, λ = 254 nm), t_1 = 8.89 min (major), t_2 = 9.52 min (minor), *ee* = 91%; ^{1}H NMR (400 MHz, $CDCl_3$) δ 7.41 (d, *J* = 2.3 Hz, 1H), 7.38 – 7.33 (m, 1H), 7.33 – 7.27 (m, 2H), 7.25 – 7.20 (m, 1H), 7.14 (d, *J* = 8.0 Hz, 1H), 7.00 (d, *J* = 8.6 Hz, 1H), 6.32 (s, 2H),

5.77 (d, J = 4.3 Hz, 2H), 4.92 (dd, J = 15.7, 3.4 Hz, 2H), 4.56 – 4.48 (m, 2H), 1.44 (d, J = 4.4 Hz, 18H); ^{13}C NMR (100 MHz, $CDCl_3$) δ 167.8, 166.5, 164.8, 164.8, 138.8, 138.1, 136.8, 136.0, 135.9, 134.9, 133.6, 130.9, 130.6, 128.6, 127.8, 127.6, 127.5, 127.0, 125.5, 121.8, 81.4, 81.3, 49.4, 49.2, 28.0. HRMS (ESI) m/z calcd for $C_{30}H_{33}BrN_2O_6$ $[M+Na]^+$= 619.1415, found = 619.1422.

(*S*)-5-benzyldibenzo[*b*,*f*][1,5]diazocine-6,12(5*H*,11*H*)-dione: **4a**

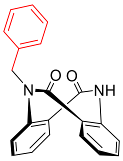

A white solid; 15.4 mg; isolated yield = 47%. M.p.: 137.3.2 – 138.9 °C; $[\alpha]_D^{20}$ = +19.07 (*c* 0.12, CH_2Cl_2); HPLC (IC column, *i*-propanol/*n*-hexane = 30/70, flow rate 1.0 mL/min, λ = 254 nm), t_1 = 16.95 min (minor), t_2 = 18.32 min (major), *ee* = 91%; ^{1}H NMR (400 MHz, $CDCl_3$) δ 8.50 (d, J = 11.9 Hz, 1H), 7.51 – 7.41 (m, 1H), 7.37 – 7.31 (m, 1H), 7.29 – 7.16 (m, 9H), 7.03 – 6.94 (m, 2H), 5.43 – 5.15 (m, 1H), 4.98 – 4.74 (m, 1H); ^{13}C NMR (100 MHz, $CDCl_3$) δ 170.3, 170.3, 168.2, 168.1, 139.19, 136.4, 134.7, 134.1, 133.3, 131.2, 130.6, 128.9, 128.7, 128.3, 127.9, 127.9, 126.6, 126.6, 125.5, 53.5. HRMS (ESI) m/z calcd for $C_{21}H_{16}N_2O_2$ $[M+H]^+$= 329.1285, found = 329.1292.

(*R*)-*Tert*-butyl 2-((11-benzyl-6,12-dioxo-11,12-dihydrodibenzo[*b*,*f*][1,5]diazocin-5(6*H*)-yl)methyl)acrylate: **5a**

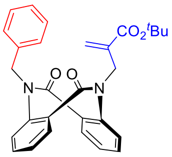

A colorless oil; 21.5 mg; isolated yield = 46%. $[A]_D^{20}$ = -79.05 (*c* 0.20, CH_2Cl_2); HPLC (IC column, *i*-propanol/*n*-hexane = 30/70, flow rate 1.0 mL/min, λ = 254 nm), t_1 = 12.34 min (minor), t_2 = 16.35 min (major), *ee* = 91%; ^{1}H NMR (400 MHz, $CDCl_3$) δ 7.37 – 7.13 (m, 11H), 7.12 – 7.07 (m, 1H), 6.90 – 6.80 (m, 1H), 6.22 (s, 1H), 5.58 (s, 1H), 5.11 – 4.92 (m, 3H), 4.45 (d, J = 15.9 Hz, 1H), 1.43 (s, 9H); ^{13}C NMR (100 MHz, $CDCl_3$) δ 168.1, 167.93 164.9, 139.3, 139.0, 136.5, 136.0, 135.2, 135.2, 130.6, 130.4, 129.1, 128.6, 128.4, 128.2, 127.9, 127.8, 127.7, 127.0, 126.0, 125.5, 81.2, 53.0, 49.4, 28.0. HRMS (ESI) m/z calcd for $C_{29}H_{28}N_2O_4$ $[M+Na]^+$= 491.1942, found = 491.1940.

(*R*)-Ethyl 2-((11-benzyl-6,12-dioxo-11,12-dihydrodibenzo[*b*,*f*][1,5]diazocin-5(6*H*)-yl)methyl)acrylate: **5b**

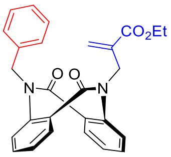

A colorless oil; 20.7 mg; isolated yield = 47%. $[A]_D^{20}$ = -101.45 (*c* 0.91, CH_2Cl_2); HPLC (IC column, *i*-propanol/*n*-hexane = 30/70, flow rate 1.0 mL/min, λ = 254 nm), t_1 = 16.75 min (minor), t_2 = 27.03 min (major), *ee* = 92%; ^{1}H NMR (400 MHz, $CDCl_3$)

δ 7.37 – 7.28 (m, 5H), 7.27 – 7.20 (m, 3H), 7.18 – 7.07 (m, 4H), 6.89 – 6.81 (m, 1H), 6.28 (s, 1H), 5.66 (s, 1H), 5.13 (d, *J* = 15.4 Hz, 1H), 5.09 – 4.97 (m, 2H), 4.37 (d, *J* = 15.4 Hz, 1H), 4.21 – 4.10 (m, 2H), 1.19 (t, *J* = 7.1 Hz, 3H); ^{13}C NMR (100 MHz, $CDCl_3$) δ 168.0, 167.9, 165.6, 139.2, 139.0, 136.5, 135.2, 135.1, 134.7, 130.6, 130.4, 129.1, 128.6, 128.4, 128.3, 128.2, 127.8, 127.7, 126.0, 125.4, 61.0, 53.0, 49.5, 14.0. HRMS (ESI) m/z calcd for $C_{27}H_{24}N_2O_4$ $[M+H]^+$= 441.1809, found = 441.1818.

(*R*)-Butyl 2-((11-benzyl-6,12-dioxo-11,12-dihydrodibenzo[*b*,*f*][1,5]diazocin-5(6*H*)-yl)methyl)acrylate: **5c**

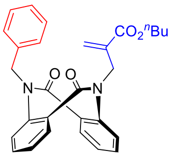

A colorless oil; 22.0 mg; isolated yield = 47%. $[A]_D^{20}$ = -74.45 (*c* 0.15, CH_2Cl_2); HPLC (IC column, *i*-propanol/*n*-hexane = 30/70, flow rate 1.0 mL/min, λ = 254 nm), t_1 = 17.46 min (minor), t_2 = 28.59 min (major), *ee* = 95%; ^{1}H NMR (400 MHz, $CDCl_3$) δ 7.35 – 7.27 (m, 6H), 7.26 – 7.22 (m, 2H), 7.21 – 7.14 (m, 3H), 7.11 – 7.05 (m, 1H), 6.88 – 6.80 (m, 1H), 6.29 (s, 1H), 5.66 (d, *J* = 0.6 Hz, 1H), 5.09 (d, *J* = 15.5 Hz, 1H), 5.03 (s, 2H), 4.40 (d, *J* = 15.2 Hz, 1H), 4.18 – 4.05 (m, 2H), 1.62 – 1.51 (m, 2H), 1.39 – 1.29 (m, 2H), 0.91 (t, *J* = 7.4 Hz, 3H); ^{13}C NMR (100 MHz, $CDCl_3$) δ 168.0, 167.9, 165.7, 139.3, 139.1, 136.5, 135.3, 135.1, 134.7, 130.6, 130.4, 129.1, 128.6, 128.4, 128.2, 128.1, 127.8, 127.8, 127.7, 126.0, 125.3, 64.9, 53.0, 49.5, 30.5, 19.2, 13.7. HRMS (ESI) m/z calcd for $C_{29}H_{28}N_2O_4$ $[M+H]^+$= 469.2122, found = 469.2127.

(*R*)-Isobutyl 2-((11-benzyl-6,12-dioxo-11,12-dihydrodibenzo[*b*,*f*][1,5]diazocin-5(6*H*)-yl)methyl)acrylate: **5d**

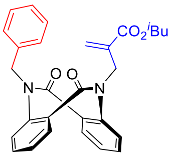

A colorless oil; 19.7 mg; isolated yield = 42%. $[A]_D^{20}$ = -365.35 (*c* 0.15, CH_2Cl_2); HPLC (IC column, *i*-propanol/*n*-hexane = 30/70, flow rate 1.0 mL/min, λ = 254 nm), t_1 = 13.41 min (major), t_2 = 14.51 min (minor), *ee* = 90%; ^{1}H NMR (400 MHz, $CDCl_3$) δ 7.35 – 7.21 (m, 8H), 7.16 – 7.07 (m, 4H), 6.90 – 6.82 (m, 1H), 6.29 (s, 1H), 5.67 (s, 1H), 5.12 (d, *J* = 15.5 Hz, 1H), 5.03 (q, *J* = 14.3 Hz, 2H), 4.41 (d, *J* = 15.5 Hz, 1H), 3.90 (qd, *J* = 10.6, 6.7 Hz, 2H), 1.94 – 1.81 (m, 1H), 0.88 (dd, *J* = 6.7, 3.2 Hz, 6H); ^{13}C NMR (100 MHz, $CDCl_3$) δ 168.0, 167.8, 165.5, 139.2, 139.0, 136.5, 135.2, 135.1, 134.7, 130.6, 130.4, 129.1, 128.5, 128.3, 128.2, 128.1, 127.8, 127.7, 126.0, 125.3, 71.0, 52.9, 49.5, 27.6, 19.2, 19.1. HRMS (ESI) m/z calcd for $C_{29}H_{28}N_2O_4$ $[M+H]^+$= 469.2122, found = 469.2122.

(*R*)-Benzyl 2-((11-benzyl-6,12-dioxo-11,12-dihydrodibenzo[*b*,*f*][1,5]diazocin-5(6*H*)-yl)methyl)acrylate: **5e**

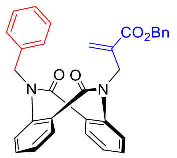

A colorless oil; 20.6 mg; isolated yield = 41%. $[A]_D^{20}$ = -42.60 (*c* 0.20, CH_2Cl_2); HPLC (IC column, *i*-propanol/*n*-hexane = 30/70, flow rate 1.0 mL/min, λ = 254 nm), t_1 = 20.05 min (major), t_2 = 27.14 min (minor), *ee* = 91%; ^1H NMR (400 MHz, $CDCl_3$) δ 7.37 – 7.11 (m, 16H), 7.10 – 7.01 (m, 1H), 6.89 – 6.78 (m, 1H), 6.32 (s, 1H), 5.69 (s, 1H), 5.24 – 5.08 (m, 3H), 5.01 (d, *J* = 1.1 Hz, 2H), 4.38 (d, *J* = 15.4 Hz, 1H); ^{13}C NMR (100 MHz, $CDCl_3$) δ 168.0, 167.9, 165.4, 139.2, 139.1, 136.5, 135.9, 135.3, 135.1, 134.5, 130.6, 130.5, 129.1, 129.0, 128.6, 128.5, 128.4, 128.3, 128.2, 128.1, 127.8, 127.7, 126.0, 125.3, 66.8, 53.0, 49.5. HRMS (ESI) m/z calcd for $C_{32}H_{26}N_2O_4$ $[M+H]^+$= 503.1966, found = 503.1970.

(*S*)-5-benzyl-3,9-dimethoxydibenzo[*b*,*f*][1,5]diazocine-6,12(5*H*,11*H*)-dione: **4f**

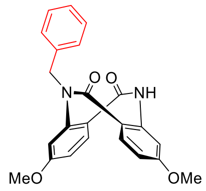

A white solid; 17.8 mg; isolated yield = 46%. M.p.: 97.5 – 99.9 °C; $[\alpha]_D^{20}$ = -10.94 (*c* 0.32, CH_2Cl_2); HPLC (IB column, *i*-propanol/*n*-hexane = 30/70, flow rate 1.0 mL/min, λ = 254 nm), t_1 = 8.18 min (minor), t_2 = 8.84 min (major), *ee* = 90%; ^1H NMR (400 MHz, $CDCl_3$) δ 8.04 (s, 1H), 7.40 (d, *J* = 8.6 Hz, 1H), 7.31 – 7.21 (m, 6H), 6.81 – 6.69 (m, 2H), 6.55 – 6.34 (m, 2H), 5.15 (d, *J* = 14.3 Hz, 1H), 4.92 (d, *J* = 14.3 Hz, 1H), 3.72 (s, 3H), 3.62 (s, 3H); ^{13}C NMR (100 MHz, $CDCl_3$) δ 170.2, 168.2, 161. 5, 161.1, 140.9, 136.8, 135.8, 129.9, 129.8, 129.0, 128.0, 127.8, 127.1, 125.4, 114.3, 113.7, 112.0, 110.6, 55.5, 55.4. HRMS (ESI) m/z calcd for $C_{23}H_{20}N_2O_4$ $[M+H]^+$= 389.1496, found = 389.1497.

(*R*)-*Tert*-butyl 2-((11-benzyl-3,9-dimethoxy-6,12-dioxo-11,12-dihydrodibenzo[*b*,*f*][1,5]diazocin-5(6*H*)-yl)methyl)acrylate: **5f**

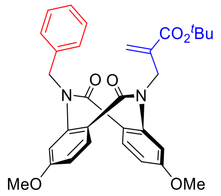

A white solid; 25.9 mg; isolated yield = 49%. M.p.: 158.1 – 158.9 °C; $[\alpha]_D^{20}$ = +36.00 (*c* 0.40, CH_2Cl_2); HPLC (IB column, *i*-propanol/*n*-hexane = 30/70, flow rate 1.0 mL/min, λ = 254 nm), t_1 = 6.07 min (major), t_2 = 6.47 min (minor), *ee* = 90%; ^1H NMR (400 MHz, $CDCl_3$) δ 7.35 – 7.26 (m, 6H), 7.22 (d, *J* = 8.6 Hz, 1H), 6.78 – 6.65 (m, 2H), 6.58 (d, *J* = 2.4 Hz, 1H), 6.27 (d, *J* = 2.4 Hz, 1H), 6.19 (s, 1H), 5.55 (s, 1H), 5.07 (d, *J* = 14.2 Hz, 1H), 5.03 – 4.87 (m, 2H), 4.38 (d, *J* = 15.9 Hz, 1H), 3.71 (s, 3H), 3.57 (s, 3H), 1.44 (s, 9H); ^{13}C NMR (100 MHz, $CDCl_3$) δ 168.1, 168.0, 164.9, 161.0, 160.7,

141.0, 140.7, 136.9, 136.0, 129.2, 129.1, 128.6, 127.8, 127.7, 127.5, 126.8, 114.1, 113.6, 111.4, 111.1, 81.1, 55.4, 55.3, 53.1, 49.5, 28.0. HRMS (ESI) m/z calcd for $C_{31}H_{32}N_2O_6$ $[M+Na]^+$= 551.2153, found = 551.2159.

(*S*)-5-benzyl-3,9-dichlorodibenzo[b,f][1,5]diazocine-6,12(5H,11H)-dione: **4g**

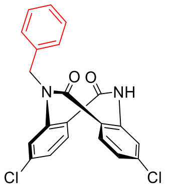

A white solid; 19.0 mg; isolated yield = 48%. M.p.: 265.8 – 267.2 °C; $[\alpha]_D^{20}$ = +49.80 (*c* 0.50, CH_2Cl_2); HPLC (IC column, *i*-propanol/*n*-hexane = 30/70, flow rate 1.0 mL/min, λ = 254 nm), t_1 = 7.01 min (major), t_2 = 8.51 min (minor), *ee* = 94%; ^{1}H NMR (400 MHz, $CDCl_3$) δ 8.38 (s, 1H), 7.40 (d, *J* = 8.3 Hz, 1H), 7.31 – 7.18 (m, 8H), 7.09 – 6.82 (m, 2H), 5.33 (t, *J* = 14.7 Hz, 1H), 4.77 (t, *J* = 14.2 Hz, 1H); ^{13}C NMR (100 MHz, $CDCl_3$) δ 169.0, 166.9, 140.0, 137.1, 136.4, 135.6, 135.0, 132.8, 131.4, 129.7, 129.6, 129.3, 128.9, 128.8, 128.6, 128.2, 127.0, 125.7, 53.6. HRMS (ESI) m/z calcd for $C_{21}H_{14}Cl_2N_2O_2$ $[M+H]^+$= 397.0506, found = 397.0504.

(*R*)-*Tert*-butyl 2-((11-benzyl-3,9-dichloro-6,12-dioxo-11,12-dihydrodibenzo[*b*,*f*][1,5]diazocin-5(6*H*)-yl)methyl)acrylate: **5g**

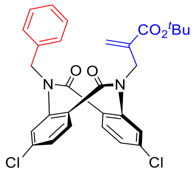

A white solid; 21.4 mg; isolated yield = 40%. M.p.: 213.6 – 214.7 °C; $[\alpha]_D^{20}$ = -56.00 (*c* 0.20, CH_2Cl_2); HPLC (IC column, *i*-propanol/*n*-hexane = 30/70, flow rate 1.0 mL/min, λ = 254 nm), t_1 = 7.70 min (minor), t_2 = 8.65 min (major), *ee* = 83%; ^{1}H NMR (400 MHz, $CDCl_3$) δ 7.35 – 7.25 (m, 6H), 7.25 – 7.19 (m, 3H), 7.10 (d, *J* = 1.8 Hz, 1H), 6.88 (d, *J* = 1.6 Hz, 1H), 6.21 (s, 1H), 5.55 (s, 1H), 5.11 (d, *J* = 14.3 Hz, 1H), 5.01 – 4.81 (m, 2H), 4.35 (d, *J* = 15.4 Hz, 1H), 1.44 (s, 9H); ^{13}C NMR (100 MHz, $CDCl_3$) δ 166.8, 166.7, 164.6, 140.2, 139.9, 136.3, 136.2, 135.7, 135.6, 133.4, 129.1, 129.0, 128.9, 128.7, 128.2, 127.9, 126.3, 125.8, 81.4, 53.1, 49.4, 28.0. HRMS (ESI) m/z calcd for $C_{29}H_{26}Cl_2N_2O_4$ $[M+Na]^+$= 559.1162, found = 559.1171.

(*S*)-5-benzyl-2,8-dichlorodibenzo[*b*,*f*][1,5]diazocine-6,12(5*H*,11*H*)-dione: **4h**

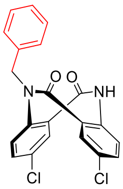

A white solid; 19.0 mg; isolated yield = 48%. M.p.: 137.2 – 138.6 °C; $[\alpha]_D^{20}$ = +2.83 (*c* 0.60, CH_2Cl_2); HPLC (IC column, *i*-propanol/*n*-hexane = 30/70, flow rate 1.0

mL/min, λ = 254 nm), t_1 = 4.98 min (minor), t_2 = 5.83 min (major), *ee* = >99%; ^{1}H NMR (400 MHz, $CDCl_3$) δ 7.98 (s, 1H), 7.42 (d, *J* = 2.4 Hz, 1H), 7.38 – 7.19 (m, 8H), 7.09 – 6.86 (m, 2H), 5.22 (d, *J* = 14.3 Hz, 1H), 4.85 (d, *J* = 14.3 Hz, 1H); ^{13}C NMR (100 MHz, $CDCl_3$) δ 168.2, 166.4, 137.2, 135.9, 135.8, 134.0, 134.4, 134.1, 132.1, 131.6, 130.9, 128.9, 128.8, 128.39, 128.4, 128.2, 127.0, 53.5. HRMS (ESI) m/z calcd for $C_{21}H_{14}Cl_2N_2O_2$ $[M+H]^+$= 397.0506, found = 397.0503.

(*R*)-*Tert*-butyl 2-((11-benzyl-2,8-dichloro-6,12-dioxo-11,12-dihydrodibenzo[*b*,*f*][1,5]diazocin-5(6*H*)-yl)methyl)acrylate: **5h**

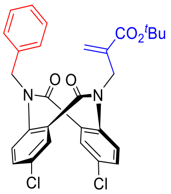

A white solid; 24.7 mg; isolated yield = 46%. M.p.: 193.5-195.9 °C; $[\alpha]_D^{20}$ = -47.34 (*c* 0.15, CH_2Cl_2); HPLC (IC column, *i*-propanol/*n*-hexane = 30/70, flow rate 1.0 mL/min, λ = 254 nm), t_1 = 7.44 min (major), t_2 = 8.25 min (minor), *ee* = 87%; ^{1}H NMR (400 MHz, $CDCl_3$) δ 7.35 – 7.24 (m, 8H), 7.15 (d, *J* = 8.6 Hz, 1H), 7.06 (d, *J* = 8.6 Hz, 1H), 6.82 – 6.74 (m, 1H), 6.23 (s, 1H), 5.57 (s, 1H), 5.05 – 4.93 (m, 3H), 4.37 (d, *J* = 15.6 Hz, 1H), 1.44 (s, 9H); ^{13}C NMR (100 MHz, $CDCl_3$) δ 166.4, 166.2, 164.7, 137.4, 137.1, 136.3, 136.3, 136.0, 135.7, 134.4, 134.3, 130.9, 130.7, 129.1, 129.0, 128.8, 128.2, 127.9, 127.8, 127.6, 127.1, 81.4, 53.0, 49.3, 28.0. HRMS (ESI) m/z calcd for $C_{29}H_{26}Cl_2N_2O_4$ $[M+Na]^+$= 559.1162, found = 559.1160.

(*S*)-5-benzyl-4,10-dichlorodibenzo[*b*,*f*][1,5]diazocine-6,12(5*H*,11*H*)-dione: **4i**

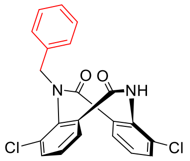

A white solid; 27.2 mg; isolated yield = 48%. M.p.: 202.1 – 203.9 °C; $[\alpha]_D^{20}$ = -27.50 (*c* 0.20, CH_2Cl_2); HPLC (IC column, *i*-propanol/*n*-hexane = 30/70, flow rate 1.0 mL/min, λ = 254 nm), t_1 = 6.60 min (major), t_2 = 8.90min (minor), *ee* = 99%; ^{1}H NMR (400 MHz, $CDCl_3$) δ 7.50 – 7.27 (m, 4H), 7.28 – 7.21 (m, 6H), 7.20 – 7.11 (m, 1H), 5.84 (d, *J* = 14.2 Hz, 1H), 4.26 (d, *J* = 14.2 Hz, 1H); ^{13}C NMR (100 MHz, $CDCl_3$) δ 167.8, 166.8, 136.4, 135.9, 135.3, 135.1, 132.5, 132.4, 131.4, 131.0, 130.5, 130.3, 129.4, 129.2, 128.6, 128.2, 126.6, 126.0, 51.6. HRMS (ESI) m/z calcd for $C_{21}H_{14}Cl_2N_2O_2$ $[M+Na]^+$= 419.0325, found = 419.0315.

(*R*)-*Tert*-butyl2-((11-benzyl-4,10-dichloro-6,12-dioxo-11,12-dihydrodibenzo[*b*,*f*][1,5] diazocin-5(6*H*)-yl)methyl)acrylate: **5i**

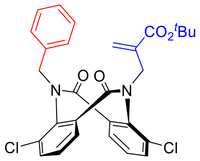

A white solid; 33.0 mg; isolated yield = 46%. M.p.: 113.0 – 114.4 °C; $[\alpha]_D^{20}$ = -59.30 (*c* 0.10, CH_2Cl_2); HPLC (IC column, *i*-propanol/*n*-hexane = 30/70, flow rate 1.0 mL/min, λ = 254 nm), t_1 = 6.33 min (minor), t_2 = 8.17 min (major), *ee* = 83%;^{1}H NMR (400 MHz, $CDCl_3$) δ 7.31 – 7.24 (m, 8H), 7.21 – 7.10 (m, 3H), 6.05 (s, 1H), 5.53 (d, *J* = 14.0 Hz, 1H), 5.38 (s, 1H), 5.10 (d, *J* = 14.8 Hz, 1H), 4.38 (d, *J* = 14.0 Hz, 1H), 4.05 (d, *J* = 14.8 Hz, 1H), 1.37 (s, 9H); ^{13}C NMR (10 MHz, $CDCl_3$) δ 166.5, 166.4, 164.7, 137.7, 137.6, 135.4, 134.8, 132.0, 131.7, 131.6, 130.3, 130.2, 129.9, 129.8, 128.35, 128.1, 125.5, 125.34, 80.9, 51.8, 46.8, 27.9. HRMS (ESI) m/z calcd for $C_{29}H_{26}Cl_2N_2O_4$ $[M+Na]^+$= 536.1270, found = 536.1273.

(*S*)-6-benzyldinaphtho[2,3-*b*:2',3'-*f*][1,5]diazocine-7,15(6*H*,14*H*)-dione: **4j**

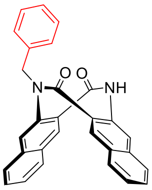

A colorless oil; 20.1 mg; isolated yield = 47%. $[A]_D^{20}$ = -135.58 (*c* 0.17, CH_2Cl_2); HPLC (IC column, *i*-propanol/*n*-hexane = 30/70, flow rate 1.0 mL/min, λ = 254 nm), t_1 = 22.24 min (major), t_2 = 27.08 min (minor), *ee* = >99%; ^{1}H NMR (400 MHz, $CDCl_3$) δ 8.38 (s, 1H), 7.97 (s, 1H), 7.89 (s, 1H), 7.80 – 7.68 (m, 2H), 7.66 – 7.60 (m, 1H), 7.59 – 7.53 (m, 1H), 7.48 (s, 1H), 7.46 – 7.37 (m, 4H), 7.37 – 7.24 (m, 6H), 5.19 (s, 2H); ^{13}C NMR (100 MHz, $CDCl_3$) δ 170.5, 168.6, 136.7, 136.0, 133.8, 133.8, 133.6, 132.1, 132.0, 131.9, 130.9, 129.2, 128. 9, 128.7, 128.4, 128.3, 128.1, 127.9, 127.9, 127.7, 127.7, 127.5, 127.4, 127.0, 126.3, 124.5, 54.2. HRMS (ESI) m/z calcd for $C_{29}H_{20}N_2O_2$ $[M+H]^+$= 429.1598, found = 429.1607.

(*R*)-*Tert*-butyl 2-((14-benzyl-7,15-dioxo-14,15-dihydrodinaphtho[2,3-*b*:2',3'-*f*][1,5]diazocin-6(7*H*)-yl)methyl)acrylate: **5j**

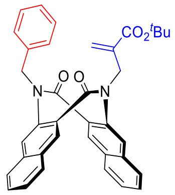

A colorless oil; 26.1 mg; isolated yield = 46%. $[A]_D^{20}$ = -13.92 (*c* 0.13, CH_2Cl_2); HPLC (IF column, *i*-propanol/*n*-hexane = 30/70, flow rate 1.0 mL/min, λ = 254 nm), t_1 = 24.47 min (minor), t_2 = 33.98 min (major), *ee* = 90%; ^{1}H NMR (400 MHz, $CDCl_3$) δ 7.85 (t, *J* = 10.2 Hz, 2H), 7.74 – 7.45 (m, 6H), 7.40 – 7.25 (m, 9H), 6.32 (s, 1H), 5.75 (d, *J* = 0.9 Hz, 1H), 5.34 (d, *J* = 14.2 Hz, 1H), 5.05 – 4.91 (m, 2H), 4.66 (d, *J* = 15.7 Hz, 1H), 1.41 (s, 9H); ^{13}C NMR (100 MHz, $CDCl_3$) δ 168.5, 168.3, 165.0, 136.9, 136.7, 136.3, 136.2, 134.1, 134.0, 133.6, 133.4, 132.1, 129.4, 129.2, 128.8, 128.7, 128.1, 128.0, 127.9, 127.7, 127.6, 127.5, 127.3, 127.2, 125.6, 124.9, 84.0, 81.2, 53.8, 50.5, 28.0. HRMS (ESI) m/z calcd for $C_{37}H_{32}N_2O_4$ $[M+Na]^+$= 591.2255, found = 591.2267.

(*S*)-5-(4-methylbenzyl)dibenzo[*b*,*f*][1,5]diazocine-6,12(5*H*,11*H*)-dione: **4k**

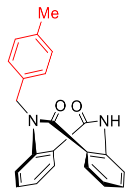

A white solid; 16.8 mg; isolated yield = 49%. M.p.: 166.7 – 167.3 °C; $[\alpha]_D^{20}$ = +27.00 (*c* 0.10, CH_2Cl_2); HPLC (IK column, *i*-propanol/*n*-hexane = 30/70, flow rate 1.0 mL/min, λ = 254 nm), t_1 = 10.48 min (minor), t_2 = 12.05 min (major), *ee* = 98%; ^{1}H NMR (400 MHz, $CDCl_3$) δ 9.12 (s, 1H), 7.44 (dd, *J* = 7.0, 2.0 Hz, 1H), 7.35 – 7.30 (m, 1H), 7.27 – 7.15 (m, 6H), 7.06 (d, *J* = 7.9 Hz, 2H), 7.00 (d, *J* = 8.4 Hz, 2H), 5.28 (d, *J* = 14.3 Hz, 1H), 4.83 (d, *J* = 14.3 Hz, 1H), 2.16 (s, 3H); ^{13}C NMR (100 MHz, $CDCl_3$) δ 170.7, 168.1, 139.2, 137.6, 134.7, 134.3, 133.4, 133.3, 131.1, 130.5, 129.3, 128.8, 128.6, 128.3, 128.2, 127.8, 126.6, 125.4, 53.2, 21.0. HRMS (ESI) m/z calcd for $C_{22}H_{18}N_2O_2$ $[M+H]^+$= 343.1442, found = 343.1449.

(*R*)-*Tert*-butyl 2-((11-(4-methylbenzyl)-6,12-dioxo-11,12-dihydrodibenzo[*b*,*f*][1,5]diazocin-5(6*H*)-yl)methyl)acrylate: **5k**

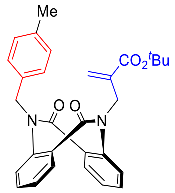

A white solid; 20.2 mg; isolated yield = 42%. M.p.: 111.3 – 113.6 °C; $[\alpha]_D^{20}$ = +103.97 (*c* 0.17, CH_2Cl_2); HPLC (IK column, *i*-propanol/*n*-hexane = 30/70, flow rate 1.0 mL/min, λ = 254 nm), t_1 = 15.15 min (minor), t_2 = 24.73 min (major), *ee* = 90%; ^{1}H NMR (400 MHz, $CDCl_3$) δ 7.32 (dd, *J* = 7.4, 1.7 Hz, 1H), 7.29 – 7.23 (m, 2H), 7.23 – 7.18 (m, 3H), 7.17 – 7.08 (m, 5H), 6.89 – 6.78 (m, 1H), 6.23 (s, 1H), 5.60 (s, 1H), 5.07 (d, *J* = 14.3 Hz, 1H), 5.00 – 4.91 (m, 2H), 4.46 (d, *J* = 15.9 Hz, 1H), 2.32 (s, 3H), 1.44 (s, 9H); ^{13}C NMR (100 MHz, $CDCl_3$) δ 168.1, 168.0, 164.9, 139.3, 139.1, 137.5, 136.0, 135.3, 135.1, 133.5, 130.6, 130.3, 129.3, 129.1, 128.3, 128.2, 127.7, 127.7, 126.9, 126.0, 125.4, 81.2, 52.7, 49.4, 28.0, 21.2. HRMS (ESI) m/z calcd for $C_{30}H_{30}N_2O_4$ $[M+Na]^+$= 505.2098, found = 505.2091.

(*S*)-5-(4-methoxybenzyl)dibenzo[*b*,*f*][1,5]diazocine-6,12(5*H*,11*H*)-dione: **4l**

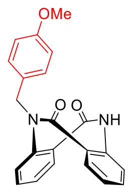

A white solid; 15.8 mg; isolated yield = 47%. M.p.: 198.5 – 199.9 °C; $[\alpha]_D^{20}$ = +35.24 (*c* 0.21, CH_2Cl_2); HPLC (IF column, *i*-propanol/*n*-hexane = 30/70, flow rate 1.0 mL/min, λ = 254 nm), t_1 = 23.12 min (major), t_2 = 29.72 min (minor), *ee* = >99%; ^{1}H NMR (400 MHz, $CDCl_3$) δ 8.37 (s, 1H), 7.42 (dd, *J* = 7.2, 1.9 Hz, 1H), 7.38 – 7.31 (m, 1H), 7.28 – 7.19 (m, 6H), 7.04 – 6.98 (m, 1H), 6.96 – 6.90 (m, 1H), 6.81 (d, *J* = 8.6 Hz,

2H), 5.11 (d, J = 14.3 Hz, 1H), 4.91 (d, J = 14.3 Hz, 1H), 3.73 (s, 3H); ^{13}C NMR (100 MHz, $CDCl_3$) δ 170.2, 168.1, 159.2, 139.2, 134.8, 134.0, 133.3, 131.1, 130.5, 130.3, 128.6, 128.6, 128.3, 128.3, 127.9, 126.7, 125.5, 114.0, 55.2, 52.8. HRMS (ESI) m/z calcd for $C_{22}H_{18}N_2O_3$ $[M+H]^+$= 359.1391, found = 359.1398.

(*R*)-*Tert*-butyl 2-((11-(4-methoxybenzyl)-6,12-dioxo-11,12-dihydrodibenzo[*b*,*f*][1,5]diazocin-5(6*H*)-yl)methyl)acrylate: **5l**

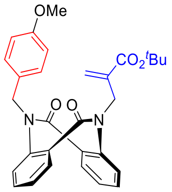

A white solid; 22.4 mg; isolated yield = 45%. M.p.: 107.1 – 108.6 °C; $[\alpha]_D^{20}$ = +96.37 (c 0.11, CH_2Cl_2); HPLC (IK column, *i*-propanol/*n*-hexane = 30/70, flow rate 1.0 mL/min, λ = 254 nm), t_1 = 11.45 min (major), t_2 = 13.44 min (minor), *ee* = 85%; ^{1}H NMR (400 MHz, $CDCl_3$) δ 7.30 (d, J = 1.7 Hz, 1H), 7.28 – 7.21 (m, 4H), 7.21 – 7.11 (m, 3H), 7.11 – 7.04 (m, 1H), 6.97 – 6.66 (m, 3H), 6.23 (s, 1H), 5.59 (s, 1H), 5.12 – 4.78 (m, 3H), 4.45 (d, J = 15.9 Hz, 1H), 3.79 (s, 3H), 1.44 (s, 9H); ^{13}C NMR (100 MHz, $CDCl_3$) δ 168.1, 164.9, 159.2, 139.3, 139.0, 136.0, 135.3, 135.2, 130.6, 130.5, 130.3, 128.7, 128.3, 128.2, 127.7, 127.7, 126.9, 126.1, 125.5, 113.9, 81.2, 55.2, 52.4, 49.4, 28.0. HRMS (ESI) m/z calcd for $C_{30}H_{30}N_2O_5$ $[M+Na]^+$= 521.2047, found = 521.2051.

(*S*)-5-(3-bromobenzyl)dibenzo[*b*,*f*][1,5]diazocine-6,12(5*H*,11*H*)-dione: **4m**

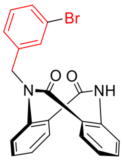

A white solid; 19.1 mg; isolated yield = 47%. M.p.: 106.8 – 107.9 °C; $[\alpha]_D^{20}$ = +28.00 (c 0.20, CH_2Cl_2); HPLC (IF column, *i*-propanol/*n*-hexane = 30/70, flow rate 1.0 mL/min, λ = 254 nm), t_1 = 13.72 min (minor), t_2 = 14.59 min (major), *ee* = 96%; ^{1}H NMR (400 MHz, $CDCl_3$) δ 8.16 (s, 1H), 7.50 – 7.43 (m, 2H), 7.41 – 7.34 (m, 2H), 7.32 – 7.20 (m, 5H), 7.18 – 7.13 (m, 1H), 7.05 – 6.96 (m, 2H), 5.24 (d, J = 14.5 Hz, 1H), 4.84 (d, J = 14.5 Hz, 1H); ^{13}C NMR (100 MHz, $CDCl_3$) δ 169.9, 168.2, 139.0, 138.6, 134.5, 133.9, 133.2, 131.8, 131.4, 131.0, 130.7, 130.2, 128.8, 128.5, 128.4, 128.1, 127.3, 126.4, 125.5, 122.7, 52.9. HRMS (ESI) m/z calcd for $C_{21}H_{15}BrN_2O_2$ $[M+H]^+$= 407.0390, found = 407.0389.

(*R*)-*Tert*-butyl 2-((11-(3-bromobenzyl)-6,12-dioxo-11,12-dihydrodibenzo[*b*,*f*][1,5]diazocin-5(6*H*)-yl)methyl)acrylate: **5m**

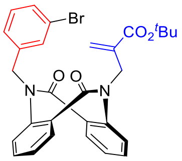

A white solid; 24.6 mg; isolated yield = 45%. M.p.: 97.4 – 98.8 °C; $[\alpha]_D^{20}$ = +122.55 (*c* 0.10, CH_2Cl_2); HPLC (IK column, *i*-propanol/*n*-hexane = 30/70, flow rate 1.0 mL/min, λ = 254 nm), t_1 = 7.82 min (minor), t_2 = 10.19 min (major), *ee* = 90%; ^1H NMR (400 MHz, $CDCl_3$) δ 7.50 (s, 1H), 7.45 – 7.32 (m, 2H), 7.31 – 7.23 (m, 3H), 7.21 – 7.18 (m, 4H), 7.12 – 7.09 (m, 1H), 6.90 – 6.83 (m, 1H), 6.25 (s, 1H), 5.61 (d, *J* = 0.9 Hz, 1H), 5.04 (d, *J* = 14.4 Hz, 1H), 4.95 (dd, *J* = 15.1, 3.7 Hz, 2H), 4.47 (d, *J* = 15.6 Hz, 1H), 1.42 (d, *J* = 8.3 Hz, 9H); ^{13}C NMR (100 MHz, $CDCl_3$) δ 168.2, 167.8, 164.8, 138.7, 136.1, 135.2, 134.9, 132.0, 131.0, 130.7, 130.5, 130.2, 128.6, 128.3, 127.9, 127.7, 127.6, 127.2, 125.8, 125.5, 122.6, 81.2, 52.4, 49.4, 28.0. HRMS (ESI) m/z calcd for $C_{29}H_{27}BrN_2O_4$ $[M+Na]^+$= 569.1047, found = 569.1058.

(*S*)-5-allyldibenzo[*b*,*f*][1,5]diazocine-6,12(5*H*,11*H*)-dione: **4n**

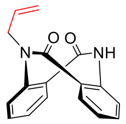

A colorless oil; 12.3 mg; isolated yield = 48%. $[A]_D^{20}$ = +98,00 (*c* 0.20, CH_2Cl_2); HPLC (IF column, *i*-propanol/*n*-hexane = 30/70, flow rate 1.0 mL/min, λ = 254 nm), t_1 = 9.11 min (minor), t_2 = 12.33 min (major), *ee* = 95%; ^1H NMR (400 MHz, DMSO) δ 10.26 (s, 1H), 7.46 – 7.25 (m, 6H), 7.23 – 7.18 (m, 1H), 7.03 (d, *J* = 7.8 Hz, 1H), 5.81 – 5.65 (m, 1H), 5.25 – 5.14 (m, 1H), 5.10 (d, *J* = 10.2 Hz, 1H), 4.84 (dd, *J* = 15.3, 5.4 Hz, 1H), 4.13 (dd, *J* = 15.3, 6.7 Hz, 1H); ^{13}C NMR (100 MHz, $CDCl_3$) δ 174.3, 172.3, 143.8, 140.0, 139.7, 139.4, 137.9, 136.1, 135.5, 133.5, 133.0, 132.7, 132.3, 131.4, 130.6, 123.5, 56.3. HRMS (ESI) m/z calcd for $C_{17}H_{14}N_2O_2$ $[M+H]^+$= 279.1129, found = 279.1136.

(*R*)-*Tert*-butyl 2-((11-allyl-6,12-dioxo-11,12-dihydrodibenzo[*b*,*f*][1,5]diazocin-5(6*H*)-yl)methyl)acrylate: **5n**

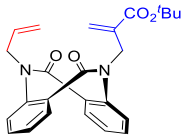

A colorless oil; 19.2 mg; isolated yield = 46%. $[A]_D^{20}$ = +136.90 (*c* 0.20, CH_2Cl_2); HPLC (IK column, *i*-propanol/*n*-hexane = 30/70, flow rate 1.0 mL/min, λ = 254 nm), t_1 = 7.67 min (minor), t_2 = 8.33 min (major), *ee* = 90%; ^1H NMR (400 MHz, $CDCl_3$) δ 7.36 – 7.23 (m, 4H), 7.22 – 7.14 (m, 2H), 7.13 – 6.99 (m, 2H), 6.31 (s, 1H), 5.98 – 5.82 (m, 1H), 5.74 (s, 1H), 5.19 (s, 1H), 5.18 – 5.11 (m, 1H), 5.05 (d, *J* = 15.9 Hz, 1H), 4.80 (dd, *J* = 14.7, 6.4 Hz, 1H), 4.47 (d, *J* = 15.9 Hz, 1H), 4.22 (dd, *J* = 14.7, 6.7 Hz, 1H), 1.45 (s, 9H); ^{13}C NMR (100 MHz, $CDCl_3$) δ 168.1, 167.6, 164.8, 139.2, 138.8, 136.1, 135.4, 135.3, 132.3, 130.6, 130.5, 128.4, 128.3, 127.7, 127.5, 126.8, 125.8, 125.5, 119.3, 81.2, 51.7, 49.5, 28.0. HRMS (ESI) m/z calcd for $C_{25}H_{26}N_2O_4$ $[M+Na]^+$= 441.1785, found = 441.1794.

(*S*)-Ethyl 2-(6,12-dioxo-11,12-dihydrodibenzo[*b*,*f*][1,5]diazocin-5(6*H*)-yl)acetate: **4o**

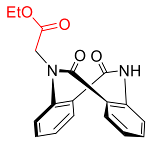

A white solid; 14.9 mg; isolated yield = 46%. M.p.: 194.1 – 195.5 °C; $[\alpha]_D^{20}$ = +9.00 (*c* 0.20, CH_2Cl_2); HPLC (IF column, *i*-propanol/*n*-hexane = 30/70, flow rate 1.0 mL/min, λ = 254 nm), t_1 = 20.42 min (major), t_2 = 26.02 min (minor), *ee* = 90%; ^1H NMR (400 MHz, $CDCl_3$) δ 7.79 (s, 1H), 7.50 – 7.40 (m, 2H), 7.40 – 7.32 (m, 2H), 7.30 – 7.22 (m, 3H), 7.04 (d, *J* = 7.4 Hz, 1H), 4.60 (d, *J* = 17.1 Hz, 1H), 4.44 (d, *J* = 17.1 Hz, 1H), 4.34 – 4.21 (m, 2H), 1.32 (t, *J* = 7.1 Hz, 3H); ^{13}C NMR (100 MHz, $CDCl_3$) δ 169.9, 168.6, 168.3, 139.7, 134.1, 133.8, 132.9, 131.7, 130.8, 128.9, 128.6, 128.2, 125.8, 125.5, 61.7, 51.9, 14.2. HRMS (ESI) m/z calcd for $C_{18}H_{16}N_2O_4$ $[M+H]^+$= 325.1183, found = 325.1191.

(*R*)-*Tert*-butyl 2-((11-(2-ethoxy-2-oxoethyl)-6,12-dioxo-11,12-dihydrodibenzo[*b,f*][1,5]diazocin-5(6*H*)-yl)methyl)acrylate: **5o**

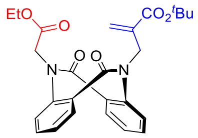

A white solid; 20.5 mg; isolated yield = 44%. M.p.: 98.4 – 99.9 °C; $[\alpha]_D^{20}$ = +36.25 (*c* 0.20, CH_2Cl_2); HPLC (IK column, *i*-propanol/*n*-hexane = 30/70, flow rate 1.0 mL/min, λ = 254 nm), t_1 = 12.11 min (minor), t_2 = 14.87 min (major), *ee* = 86%; ^1H NMR (400 MHz, $CDCl_3$) δ 7.34 – 7.18 (m, 7H), 7.14 (d, *J* = 7.9 Hz, 1H), 6.31 (s, 1H), 5.76 (s, 1H), 5.02 (d, *J* = 16.0 Hz, 1H), 4.68 (d, *J* = 17.1 Hz, 1H), 4.53 (d, *J* = 16.0 Hz, 1H), 4.37 – 4.22 (m, 3H), 1.44 (s, 9H), 1.32 (t, *J* = 7.1 Hz, 3H); ^{13}C NMR (100 MHz, $CDCl_3$) δ 168.7, 168.4, 168.0, 164.9, 139.8, 139.2, 136.0, 134.8, 134.4, 130.9, 128.6, 128.4, 128.0, 127.8, 126.9, 125.5, 125.2, 81.2, 61.7, 51.4, 49.5, 28.0, 14.2. HRMS (ESI) m/z calcd for $C_{26}H_{28}N_2O_6$ $[M+Na]^+$= 487.1840, found = 487.1840.

(*S*)-5-phenyldibenzo[*b,f*][1,5]diazocine-6,12(5*H*,11*H*)-dione: **4p**

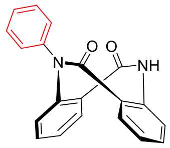

A white solid; 13.5 mg; isolated yield = 42%. M.p.: 193.2 – 194.5 °C; $[\alpha]_D^{20}$ = +36.18(*c* 0.20, CH_2Cl_2); HPLC (AD column, *i*-propanol/*n*-hexane = 30/70, flow rate 1.0 mL/min, λ = 254 nm), t_1 = 20.24 min (minor), t_2 = 22.02 min (major), *ee* = 97%; ^1H NMR (400 MHz, DMSO) δ 10.48 (s, 1H), 7.47 – 7.43 (m, 3H), 7.40 – 7.25 (m, 8H), 7.21 – 7.14 (m, 1H), 7.10 (d, *J* = 7.7 Hz, 1H); ^{13}C NMR (100 MHz, DMSO) δ 169.7, 167.4, 142.0, 139.6, 135.3, 135.2, 134.8, 131.5, 130.9, 129.7, 129.2, 128.1, 127.9, 127.8, 127.6, 127.4, 126.0. HRMS (ESI) m/z calcd for $C_{20}H_{14}N_2O_2$ $[M+H]^+$= 315.1129, found = 315.1132.

(*R*)-*Tert*-butyl 2-((6,12-dioxo-11-phenyl-11,12-dihydrodibenzo[*b,f*][1,5]diazocin-

5(6*H*)-yl)methyl)acrylate: **5p**

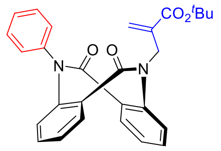

A white solid; 22.2 mg; isolated yield = 49%. M.p.: 175.0 – 176.5 °C; $[\alpha]_D^{20}$ = -47.24 (*c* 1.18, CH_2Cl_2); HPLC (IB column, *i*-propanol/*n*-hexane = 1/99, flow rate 1.0 mL/min, λ = 254 nm), t_1 = 7.00 min (major), t_2 = 7.71 min (minor), *ee* = 90%; ^{1}H NMR (400 MHz, $CDCl_3$) δ 7.46 – 7.34 (m, 6H), 7.33 – 7.26 (m, 2H), 7.26 – 7.23 (m, 1H), 7.22 – 7.14 (m, 3H), 7.00 – 6.92 (m, 1H), 6.25 (s, 1H), 5.73 (s, 1H), 5.05 (d, *J* = 16.0 Hz, 1H), 4.57 (d, *J* = 16.0 Hz, 1H), 1.38 (s, 9H); ^{13}C NMR (100 MHz, $CDCl_3$) δ 168.4, 167.3, 164.8, 141.2, 139.7, 139.2, 135.8, 135.6, 135.5, 130.8, 130.7, 129.3, 128.5, 128.4, 127.7, 127.4, 127.3, 127.2, 127.0, 126.8, 125.6, 81.2, 49.6, 27.9. HRMS (ESI) m/z calcd for $C_{28}H_{26}N_2O_4$ $[M+Na]^+$= 477.1785, found = 477.1788.

(±)-5-benzoyldibenzo[*b*,*f*][1,5]diazocine-6,12(5*H*,11*H*)-dione: **4q**

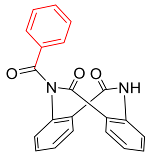

A white solid; 32.5 mg; isolated yield = 95%. M.p.: 117.6 – 118.5 °C; ^{1}H NMR (400 MHz, DMSO) δ 10.67 (s, 1H), 7.82 (d, *J* = 6.4 Hz, 2H), 7.68 – 7.40 (m, 9H), 7.33 – 7.16 (m, 2H); ^{13}C NMR (100 MHz, DMSO) δ 171.4, 169.7, 169.2, 135.8, 134.8, 134.8, 133.8, 133.3, 133.0, 132.7, 131.2, 130.6, 130.4, 130.4, 129.2, 128.8, 128.1, 127.9, 127.3. HRMS (ESI) m/z calcd for $C_{21}H_{14}N_2O_3$ $[M+H]^+$= 343.1078, found = 343.1081.

(*R*)-*tert*-butyl 2-((11-benzoyl-6,12-dioxo-11,12-dihydrodibenzo[*b*,*f*][1,5]diazocin-5(6*H*)-yl)methyl)acrylate: **5q**

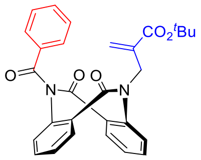

A white solid; 42.4 mg; isolated yield = 88%. M.p.: 143.2 – 144.1 °C;$[\alpha]_D^{20}$ = -36.15 (*c* 0.12, CH_2Cl_2); HPLC (IH column, *i*-propanol/*n*-hexane = 30/70, flow rate 1.0 mL/min, λ = 254 nm), t_1 = 6.04 min (minor), t_2 = 6.56 min (major), *ee* = 87%; ^{1}H NMR (400 MHz, $CDCl_3$) δ 7.82 – 7.75 (m, 2H), 7.57 – 7.48 (m, 1H), 7.45 (t, *J* = 7.4 Hz, 2H), 7.38 – 7.26 (m, 6H), 7.24 – 7.16 (m, 2H), 6.49 (s, 1H), 6.02 (d, *J* = 0.9 Hz, 1H), 4.99 (d, *J* = 15.1 Hz, 1H), 4.78 (d, *J* = 15.0 Hz, 1H), 1.34 (s, 9H); ^{13}C NMR (100 MHz, $CDCl_3$) δ 171.2, 169.1, 168.2, 164.8, 139.6, 136.7, 135.1, 134.7, 134.5, 134.0, 132.6, 131.9, 130.3, 129.4, 129.0, 129.0, 128.9, 128.5, 128.4, 127.4, 125.9, 81.6, 50.1, 27.8. HRMS (ESI) m/z calcd for $C_{29}H_{26}N_2O_5$ $[M+Na]^+$= 505.1734, found = 505.1741.

(±)-*Tert*-butyl 6,12-dioxo-11,12-dihydrodibenzo[*b*,*f*][1,5]diazocine-5(6*H*)-carboxylate: **4r**

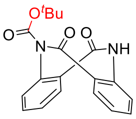

A white solid; 32.4 mg; isolated yield = 96%. M.p.: 188.1 – 188.9 °C; ^{1}H NMR (400 MHz, $CDCl_3$) δ 8.02 (s, 1H), 7.54 – 7.46 (m, 1H), 7.46 – 7.41 (m, 1H), 7.36 – 7.26 (m, 3H), 7.25 – 7.19 (m, 2H), 7.01 (d, *J* = 7.8 Hz, 1H), 1.39 (s, 9H); ^{13}C NMR (100 MHz, $CDCl_3$) δ 169.7, 167.6, 150.1, 136.1, 134.1, 134.0, 132.6, 132.0, 131.1, 129.8, 129.7, 128.8, 128.2, 127.9, 126.12, 84.21, 27.7. HRMS (ESI) m/z calcd for $C_{19}H_{18}N_2O_4$ $[M+Na]^+$= 361.1159, found = 361.1168.

(*R*)-*tert*-butyl 11-(2-(tert-butoxycarbonyl)allyl)-6,12-dioxo-11,12-dihydrodibenzo[*b,f*][1,5]diazocine-5(6*H*)-carboxylate: **5r**

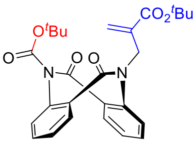

A white solid; 43.0 mg; isolated yield = 90%. M.p.: 191.1 – 192.5 °C; $[\alpha]_D^{20}$ = -75.65 (*c* 0.30, CH_2Cl_2); HPLC (IC column, *i*-propanol/*n*-hexane = 30/70, flow rate 1.0 mL/min, λ = 254 nm), t_1 = 7.81 min (minor), t_2 = 8.45 min (major), *ee* = 93%; ^{1}H NMR (400 MHz, $CDCl_3$) δ 7.37 – 7.30 (m, 2H), 7.30 – 7.23 (m, 3H), 7.20 – 7.15 (m, 1H), 7.14 – 7.07 (m, 2H), 6.30 (s, 1H), 5.88 (s, 1H), 5.14 (d, *J* = 15.8 Hz, 1H), 4.38 (d, *J* = 15.8 Hz, 1H), 1.41 (s, 9H), 1.39 (s, 9H); ^{13}C NMR (100 MHz, $CDCl_3$) δ 167.7, 167.5, 164.9, 150.1, 139.3, 136.1, 135.9, 134.6, 131.9, 130.3, 129.4, 128.9, 128.5, 128.4, 127.3, 127.2, 126.4, 84.3, 81.2, 49.2, 27.9, 27.74. HRMS (ESI) m/z calcd for $C_{27}H_{30}N_2O_6$$[M+Na]^+$= 501.1997, found = 501.2001.

(*S*)-*Tert*-butyl 2-((6,12-dioxo-11,12-dihydrodibenzo[*b,f*][1,5]diazocin-5(6*H*)-yl)methyl)acrylate: **1a′**

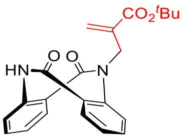

A white solid; 18.1 mg; isolated yield = 48%. M.p.: 134.7 – 135.4 °C; $[\alpha]_D^{20}$ = -8.20 (*c* 0.09, CH_2Cl_2); HPLC (ID column, *i*-propanol/*n*-hexane = 30/70, flow rate 1.0 mL/min, λ = 254 nm), t_1 = 13.10 min (major), t_2 = 17.47 min (minor), *ee* = 99%; ^{1}H NMR (400 MHz, $CDCl_3$) δ 8.40 (s, 1H), 7.43 – 7.32 (m, 3H), 7.28 – 7.19 (m, 4H), 7.04 (d, *J* = 7.8 Hz, 1H), 6.31 (s, 1H), 5.80 (s, 1H), 5.15 (d, *J* = 15.9 Hz, 1H), 4.42 (d, *J* = 15.9 Hz, 1H), 1.45 (s, 9H); ^{13}C NMR (100 MHz, $CDCl_3$) δ 170.5, 168.1, 164.8, 139.6, 136.0, 134.6, 133.9, 133.2, 131.5, 130.7, 128.6, 128.4, 128.4, 128.0, 127.0, 126.1, 125.4, 81.3, 50.2, 28.0. HRMS (ESI) m/z calcd for $C_{22}H_{22}N_2O_4$ $[M+Na]^+$= 401.1472, found = 401.1470.

(*S*)-5-benzoyl-11-benzyldibenzo[*b,f*][1,5]diazocine-6,12(5*H*,11*H*)-dione: **6a**

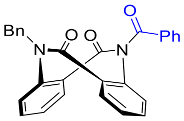

A white solid; 41.9 mg; isolated yield = 97%. M.p.: 162.1 – 162.9 °C; $[\alpha]_D^{20}$ = -9.00 (*c* 0.10, CH_2Cl_2); HPLC (IB column, *i*-propanol/*n*-hexane = 30/70, flow rate 1.0 mL/min, λ = 254 nm), t_1 = 8.14 min (major), t_2 = 11.88 min (minor), *ee* = 98%; ^{1}H NMR (400 MHz, $CDCl_3$) δ 7.74 – 7.60 (m, 2H), 7.54 – 7.46 (m, 1H), 7.43 – 7.26 (m, 12H), 7.22 – 7.16 (m, 2H), 6.86 – 6.77 (m, 1H), 5.48 (d, *J* = 14.2 Hz, 1H), 4.94 (d, *J* = 14.2 Hz, 1H); ^{13}C NMR (100 MHz, $CDCl_3$) δ 171.2, 169.1, 168.0, 139.1, 136.7, 135.0, 134.7, 134.6, 134.0, 132.4, 131.8, 130.3, 129.5, 129.3, 129.0, 129.0, 128.9, 128.8, 128.7, 128.5, 128.2, 127.5, 126.8, 53.1. HRMS (ESI) m/z calcd for $C_{28}H_{20}N_2O_3$ $[M+H]^+$= 433.1547, found = 433.1559.

<u>(*S*)-5-benzyl-11-(furan-2-carbonyl)dibenzo[*b*,*f*][1,5]diazocine-6,12(5*H*,11*H*)-dione:</u> **<u>6b</u>**

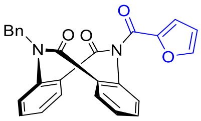

A white solid; 40.5 mg; isolated yield = 96%. M.p.: 176.5 – 177.9 °C; $[\alpha]_D^{20}$ = -35.5 (*c* 0.20, CH_2Cl_2); HPLC (IB column, *i*-propanol/*n*-hexane = 30/70, flow rate 1.0 mL/min, λ = 254 nm), t_1 = 10.97 min (major), t_2 = 23.02 min (minor), *ee* = 97%; ^{1}H NMR (400 MHz, $CDCl_3$) δ 7.50 (d, *J* = 0.9 Hz, 1H), 7.45 – 7.26 (m, 10H), 7.25 – 7.18 (m, 2H), 7.09 – 7.00 (m, 1H), 6.86 – 6.76 (m, 1H), 6.45 (dd, *J* = 3.6, 1.7 Hz, 1H), 5.40 (d, *J* = 14.3 Hz, 1H), 4.89 (d, *J* = 14.3 Hz, 1H); ^{13}C NMR (100 MHz, $CDCl_3$) δ 168.7, 167.9, 159.7, 147.3, 146.9, 139.2, 136.7, 135.0, 134.6, 133.5, 131.8, 130.5, 129.6, 129.3, 129.1, 128.8, 128.7, 128.0, 127.6, 127.1, 120.6, 112.8, 53.2. HRMS (ESI) m/z calcd for $C_{26}H_{18}N_2O_4$ $[M+H]^+$= 423.1340, found = 423.1342.

<u>(*S*)-5-(2-naphthoyl)-11-benzyldibenzo[b,f][1,5]diazocine-6,12(5H,11H)-dione:</u> **<u>6c</u>**

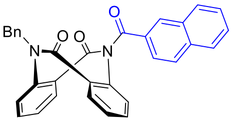

A white solid; 44.3 mg; isolated yield = 92%. M.p.: 182.3 – 183.7 °C; $[\alpha]_D^{20}$= -26.00 (*c* 0.17, CH_2Cl_2); HPLC (IB column, *i*-propanol/*n*-hexane = 30/70, flow rate 1.0 mL/min, λ = 254 nm), t_1 = 9.33 min (major), t_2 = 10.42 min (minor), *ee* = >99%;^{1}H NMR (400 MHz, $CDCl_3$) δ 8.39 (s, 1H), 7.92 (d, *J* = 8.0 Hz, 1H), 7.88 – 7.81 (m, 2H), 7.80 – 7.70 (m, 1H), 7.60 – 7.49 (m, 2H), 7.46 – 7.40 (m, 3H), 7.38 – 7.33 (m, 4H), 7.29 – 7.26 (m, 2H), 7.22 – 7.11 (m, 3H), 6.82 – 6.74 (m, 1H), 5.68 (d, *J* = 14.2 Hz, 1H), 4.90 (d, *J* = 14.2 Hz, 1H); ^{13}C NMR (100 MHz, $CDCl_3$) δ 171.3, 169.2, 168.2, 138.9, 136.8, 135.3, 135.2, 134.8, 134.0, 132.5, 131.7, 131.6, 130.7, 130.4, 129.5, 129.4, 129.2, 129.0, 128.9, 128.9, 128.8, 128.7, 128.6, 128.4, 128.4, 128.2, 127.8, 127.6, 126.8, 126.8, 124.8, 53.1. HRMS (ESI) m/z calcd for $C_{32}H_{22}N_2O_3$$[M+H]^+$= 483.1704, found = 483.1710.

(*R*)-*N*-(3,3-dimethylbutan-2-yl)benzamide: **8a**

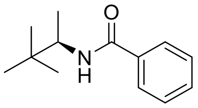

A colorless oil; 10.0 mg; isolated yield = 48%. $[A]_D^{20}$= -89.5 (*c* 0.20, CH_2Cl_2); HPLC (IB column, *i*-propanol/*n*-hexane = 30/70, flow rate 1.0 mL/min, λ = 254 nm), t_1 = 3.69 min (major), t_2 = 4.29 min (minor), *ee* = 81%; ^{1}H NMR (400 MHz, $CDCl_3$) δ 7.90 – 7.67 (m, 2H), 7.61 – 7.34 (m, 3H), 5.99 (d, *J* = 7.6 Hz, 1H), 4.22 – 3.97 (m, 1H), 1.16 (d, *J* = 6.8 Hz, 3H), 0.97 (s, 9H); ^{13}C NMR (100 MHz, $CDCl_3$) δ 166.9, 135.3, 131.3, 128.6, 126.8, 53.1, 34.5, 26.3, 16.2. HRMS (ESI) m/z calcd for $C_{13}H_{19}NO[M+H]^+$= 206.1540, found = 206.1543.

(*R*)-*N*-(3,3-dimethylbutan-2-yl)furan-2-carboxamide: **8b**

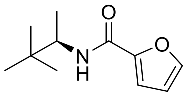

A white solid; 9.0 mg; isolated yield = 46%. M.p.: 75.0 – 78.5 °C; $[\alpha]_D^{20}$= + 4.5 (*c* 0.20, CH_2Cl_2); HPLC (IB column, *i*-propanol/*n*-hexane = 30/70, flow rate 1.0 mL/min, λ = 254 nm), t_1 = 3.91 min (major), t_2 = 4.45 min (minor), *ee* = 38%; ^{1}H NMR (400 MHz, $CDCl_3$) δ 7.43 (dd, *J* = 1.6, 0.7 Hz, 1H), 7.10 (dd, *J* = 3.5, 0.7 Hz, 1H), 6.50 (dd, *J* = 3.5, 1.8 Hz, 1H), 6.21 (d, *J* = 7.2 Hz, 1H), 4.12 – 3.97 (m, 1H), 1.15 (d, *J* = 6.8 Hz, 3H), 0.96 (s, 9H); ^{13}C NMR (100 MHz, $CDCl_3$) δ 157.8, 148.3, 143.6, 114.0, 112.2, 52.4, 34.4, 26.2, 16.2. HRMS (ESI) m/z calcd for $C_{11}H_{17}NO_2[M+H]^+$= 196.1333, found = 196.1334.

(*R*)-*N*-(3,3-dimethylbutan-2-yl)-2-naphthamide: **8c**

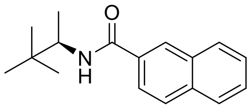

A colorless oil; 11.7 mg; isolated yield = 46%. $[A]_D^{20}$= -36.65 (*c* 0.2, CH_2Cl_2); HPLC (IB column, *i*-propanol/*n*-hexane = 30/70, flow rate 1.0 mL/min, λ = 254 nm), t_1 = 4.48 min (major), t_2 = 5.00 min (minor), *ee* = 50%; ^{1}H NMR (400 MHz, $CDCl_3$) δ 8.25 (s, 1H), 7.98 – 7.77 (m, 4H), 7.63 – 7.48 (m, 2H), 6.07 (d, *J* = 9.3 Hz, 1H), 4.24 – 4.11 (m, 1H), 1.21 (d, *J* = 6.8 Hz, 3H), 1.02 (s, 9H); ^{13}C NMR (100 MHz, $CDCl_3$) δ 167.0, 128.9, 128.5, 127.8, 127.6, 127.1, 126.8, 123.6, 53.3, 34.6, 26.3, 16.3. HRMS (ESI) m/z calcd for $C_{17}H_{21}NO$ $[M+H]^+$= 256.1696, found = 256.1694.

(*S*)-15-methoxy-11*H*-dibenzo[3,4:7,8][1,5]diazocino[2,1-*b*]quinazoline-11,17(10*H*)-dione: **10**

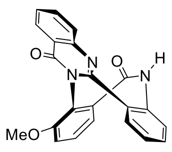

A white solid; 17.7 mg; isolated yield = 48%. HPLC (AD column, *i*-propanol/*n*-hexane = 30/70, flow rate 1.0 mL/min, λ = 254 nm), t_1 = 10.522 min (minor), t_2 = 13.388 min (major), *ee* = 91%; ^{1}H NMR (400 MHz, DMSO) δ 10.22 (s, 1H), 8.21 (d, *J* = 7.9 Hz, 1H), 7.96 (t, *J* = 7.6 Hz, 1H), 7.79 (d, *J* = 8.1 Hz, 1H), 7.66 (t, *J* = 7.5 Hz, 1H), 7.52 (d, *J* = 7.5 Hz, 1H), 7.44 (t, *J* = 7.9 Hz, 2H), 7.33 (t, *J* = 7.5 Hz, 1H), 7.15 (t, *J* = 7.5 Hz, 2H), 6.99 (d, *J* = 7.5 Hz, 1H), 3.68 (s, 3H); ^{13}C NMR (100 MHz, DMSO) δ 168.8, 160.3, 153.9, 147.6, 137.0, 135.9, 135.0, 134.0, 132.0, 131.7, 129.2, 128.4, 128.1, 128.0, 127.1, 126.8, 123.1, 120.5, 118.9, 114.2, 56.9. ((***R*-10**): HPLC (AD column, *i*-propanol/*n*-hexane = 30/70, flow rate 1.0 mL/min, λ = 254 nm), t_1 = 7.767 min (major), t_2 = 9.542min (minor), *ee* = -87%)

(*R*)-*tert*-butyl 2-((15-methoxy-11,17-dioxo-11*H*-dibenzo[3,4:7,8][1,5]diazocino[2,1-*b*]quinazolin-10(17*H*)-yl)methyl)acrylate: **11**

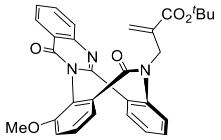

A white solid; 23.9 mg; isolated yield = 47%. HPLC (IG column, *i*-propanol/*n*-hexane = 30/70, flow rate 1.0 mL/min, λ = 254 nm), t_1 = 29.639 min (major), t_2 = 38.923 min (minor), *ee* = 88%; ^{1}H NMR (400 MHz, DMSO) δ 8.25 (d, *J* = 7.9 Hz, 1H), 7.99 (t, *J* = 7.6 Hz, 1H), 7.81 (d, *J* = 8.1 Hz, 1H), 7.70 (t, *J* = 7.6 Hz, 1H), 7.64 – 7.25 (m, 6H), 7.17 (d, *J* = 8.4 Hz, 1H), 7.03 (d, *J* = 7.6 Hz, 1H), 5.76 (s, 1H), 5.27 (s, 1H), 5.15 (d, *J* = 15.1 Hz, 1H), 4.17 (d, *J* = 15.2 Hz, 1H), 3.69 (s, 3H), 1.22 (s, 9H); ^{13}C NMR (100 MHz, DMSO) δ 166.8, 164.4, 160.3, 153.7, 153.3, 147.5, 140.8, 137.0, 135.8, 135.6, 134.4, 132.1, 131.8, 128.9, 128.3, 127.8, 127.44, 127.0, 123.2, 120.7, 118.8, 114.2, 80.9, 56.8, 48.7, 27.8.

(*S*)-15-hydroxy-11*H*-dibenzo[3,4:7,8][1,5]diazocino[2,1-*b*]quinazoline-11,17(10*H*)-dione: **12**

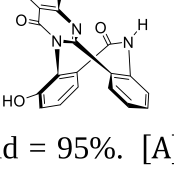

A white solid; 16.2 mg; isolated yield = 95%. $[A]_D^{20}$= +65.0 (*c* 0.1, CH_3OH); HPLC (AD column, *i*-propanol/*n*-hexane = 30/70, flow rate 1.0 mL/min, λ = 254 nm), t_1 = 6.308 min (major), t_2 = 9.752 min (minor), *ee* = 91%; ^{1}H NMR (400 MHz, CD_3OD) δ 8.31 (d, *J* = 8.0 Hz, 1H), 7.94 (t, *J* = 7.7 Hz, 1H), 7.81 (d, *J* = 8.1 Hz, 1H), 7.65 (dd, *J* = 15.8, 8.2 Hz, 2H), 7.48 (t, *J* = 7.2 Hz, 1H), 7.40 (t, *J* = 7.4 Hz, 1H), 7.30 (t, *J* = 7.9 Hz, 1H), 7.19 (d, *J* = 7.9 Hz, 1H), 6.94 (d, *J* = 4.9 Hz, 1H), 6.92 (d, *J* = 4.4 Hz, 1H);

^{13}C NMR (100 MHz, DMSO) δ 169.2, 160.3, 154.2, 152.5, 147.7, 137.0, 135.7, 135.1, 134.2, 131.6, 131.4, 129.1, 128.2, 128.0, 127.9, 127.0, 126.8, 122.0, 120.8, 118.1, 117.4. ((***R*-12**): $[\alpha]_D^{20}$= -32.0 (*c* 0.09, CH_3OH); HPLC (AD column, *i*-propanol/*n*-hexane = 30/70, flow rate 1.0 mL/min, λ = 254 nm), t_1 = 4.926 min (minor), t_2 = 6.841min (major), *ee* = -87%)

8. Crystallographic data collection for compound 3m

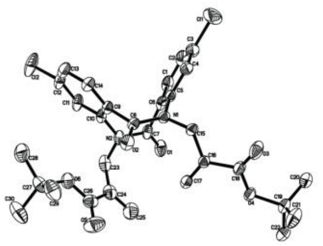

Supplementary Table 15. Crystal data and structure refinement for 20230343_auto.

Identification code	20230343_auto
Empirical formula	$C_{30}H_{32}Cl_2N_2O_6$
Formula weight	587.47
Temperature/K	293(2)
Crystal system	orthorhombic
Space group	$P2_12_12_1$
a/Å	9.06675(8)
b/Å	18.47429(15)
c/Å	18.5088(2)
α/°	90
β/°	90
γ/°	90
Volume/Å^3	3100.25(5)
Z	4
ρ_{calc}g/cm^3	1.259
μ/mm^{-1}	2.241
F(000)	1232.0
Crystal size/mm^3	0.15 × 0.11 × 0.1
Radiation	CuKα (λ = 1.54184)
2Θ range for data collection/°	6.76 to 140.702
Index ranges	$-11 \le h \le 11$, $-22 \le k \le 14$, $-22 \le l \le 22$
Reflections collected	11273
Independent reflections	5803 [R_{int} = 0.0240, R_{sigma} = 0.0352]
Data/restraints/parameters	5803/8/392
Goodness-of-fit on F^2	1.029
Final R indexes [I>=2σ (I)]	R_1 = 0.0465, wR_2 = 0.1218
Final R indexes [all data]	R_1 = 0.0524, wR_2 = 0.1290
Largest diff. peak/hole / e Å^{-3}	0.26/-0.29
Flack parameter	0.000(9)

9. NMR spectras

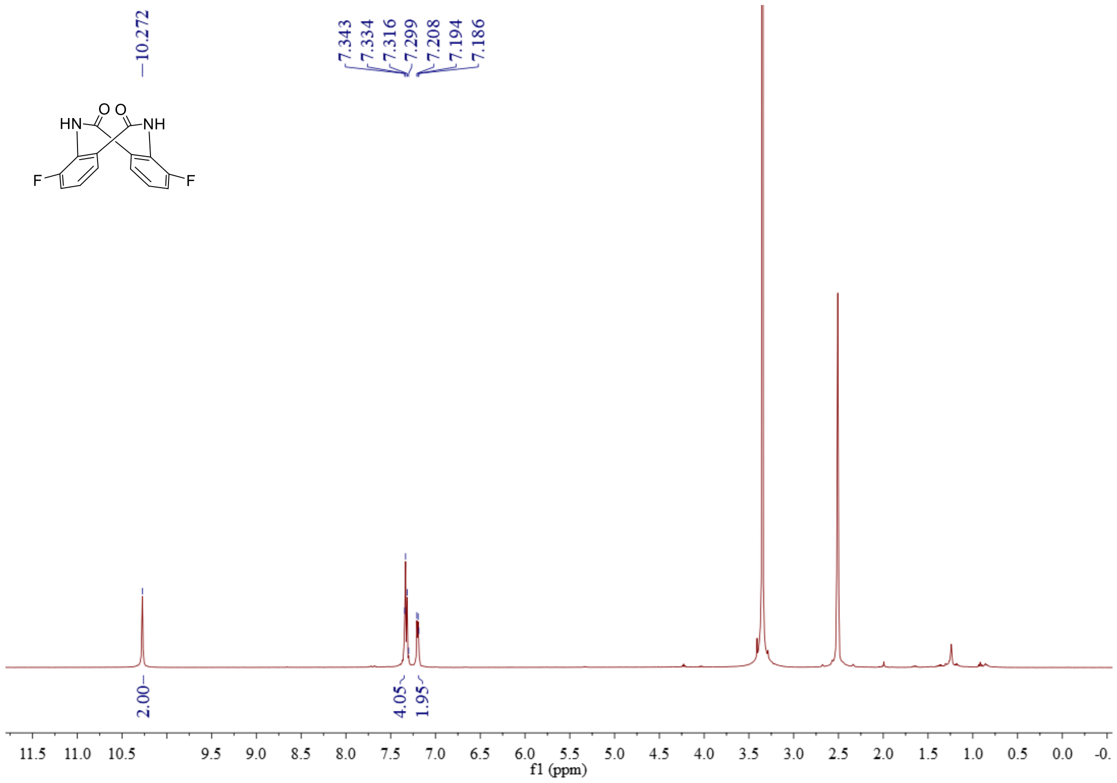

Supplementary Figure 3. ^{1}H NMR spectrum of compound **1g** ($(CD_3)_2SO$, 400 MHz)

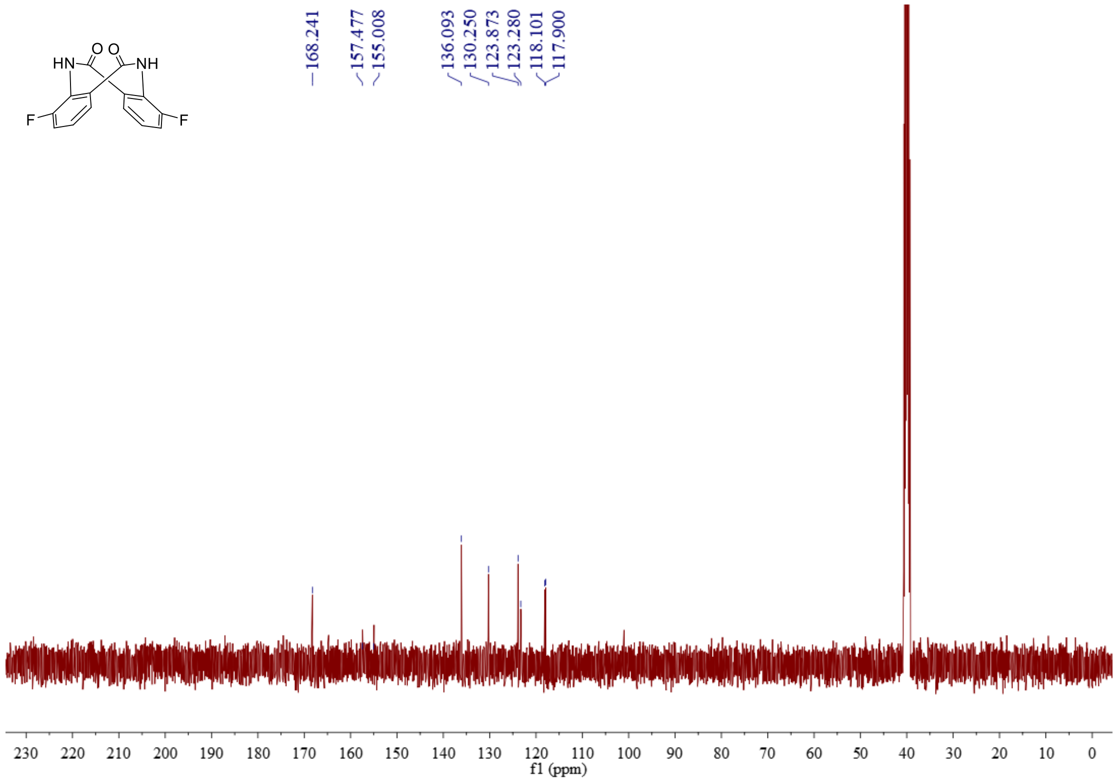

Supplementary Figure 4. ^{13}C NMR spectrum of compound **1g** ($(CD_3)_2SO$, 100 MHz)

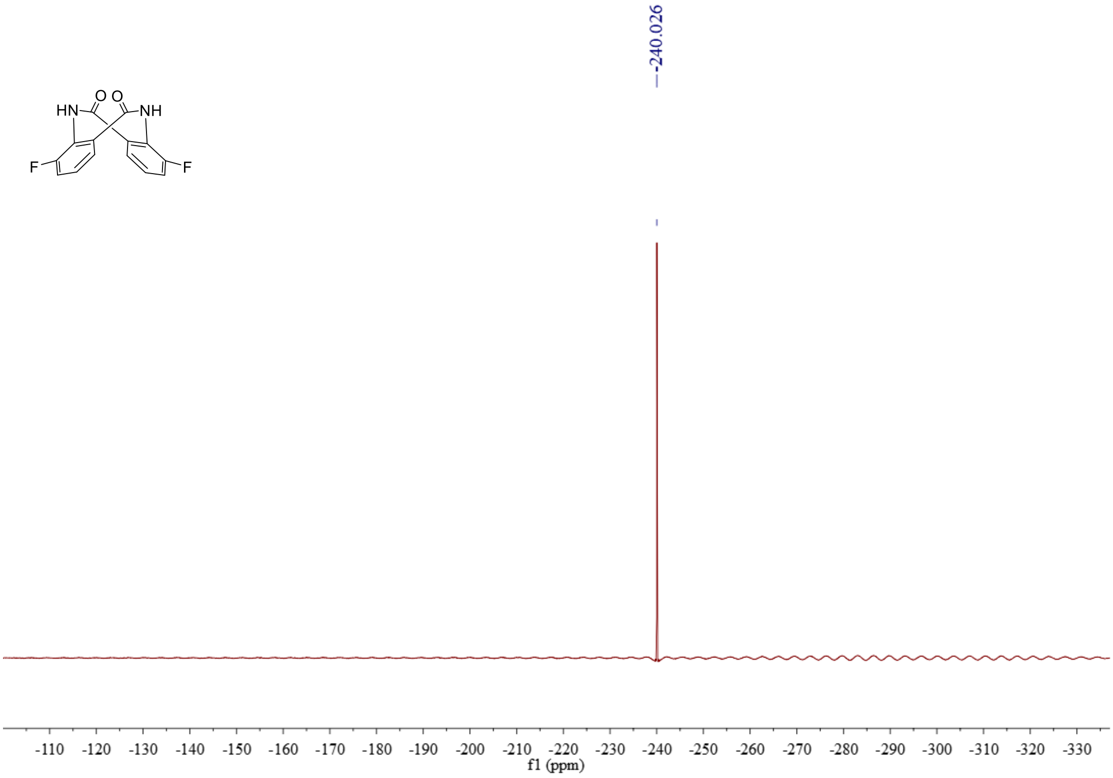

Supplementary Figure 5. ^{19}F NMR spectrum of compound **1g** ($(CD_3)_2SO$, 376 MHz)

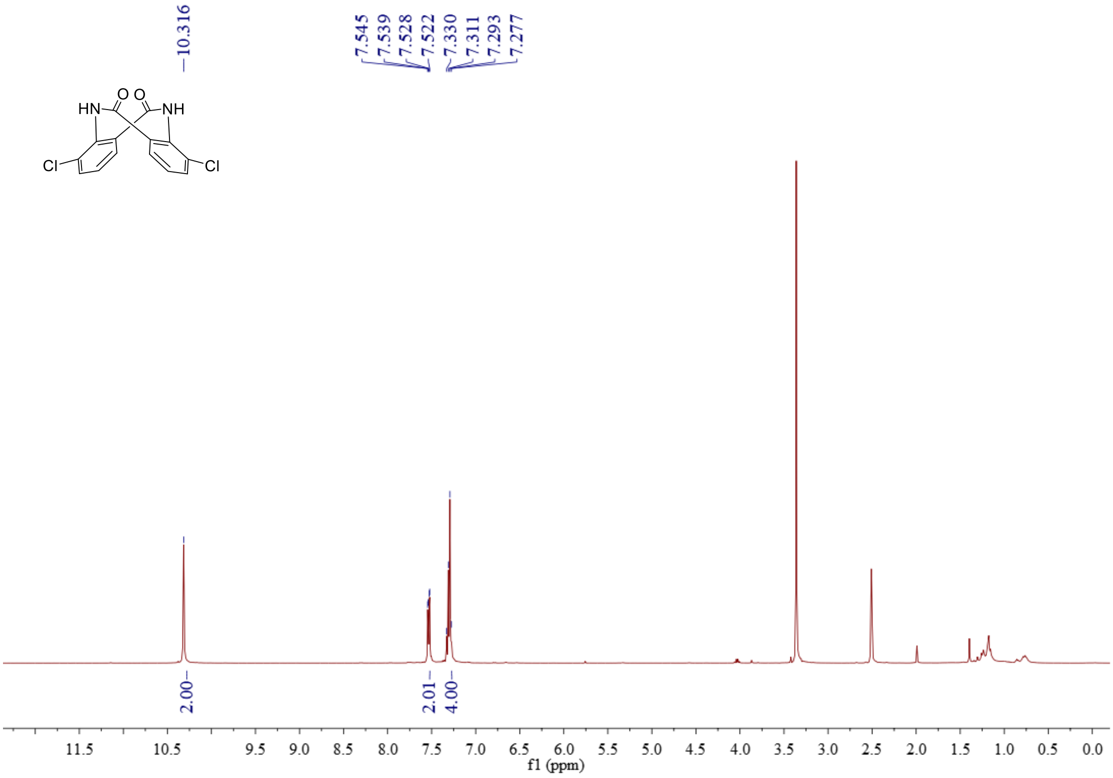

Supplementary Figure 6. ^{1}H NMR spectrum of compound **1h** ($(CD_3)_2SO$, 400 MHz)

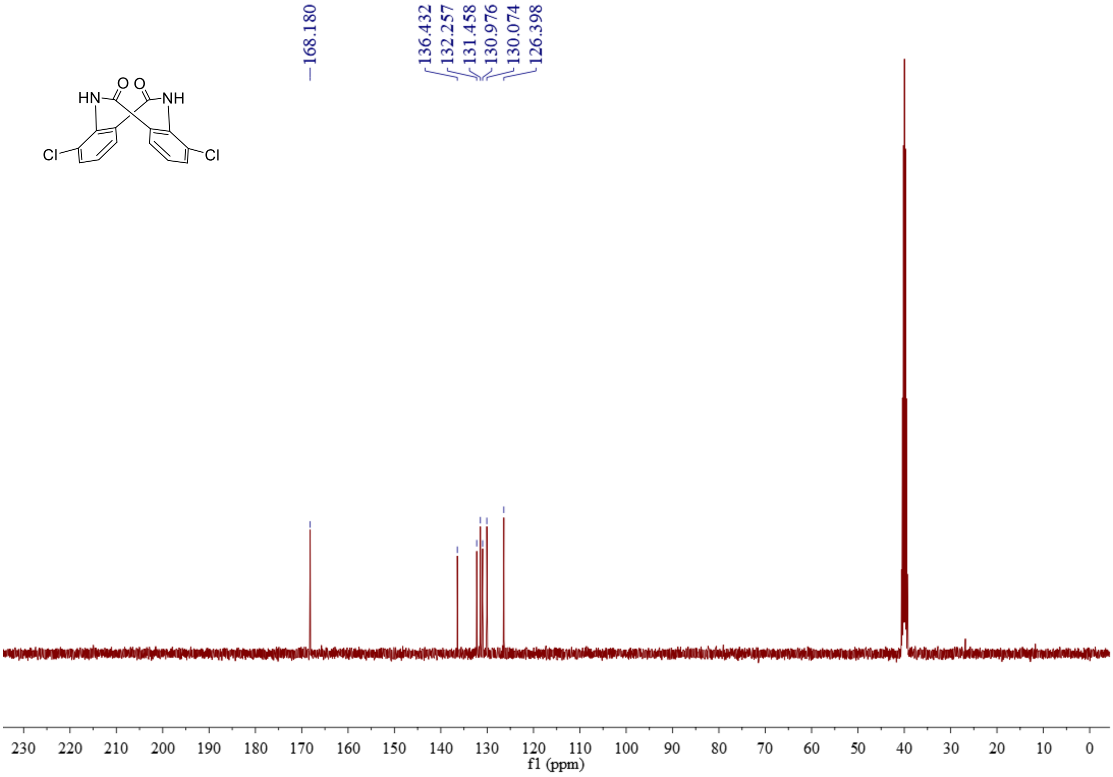

Supplementary Figure 7. ^{13}C NMR spectrum of compound **1h** ($(CD_3)_2SO$, 100 MHz)

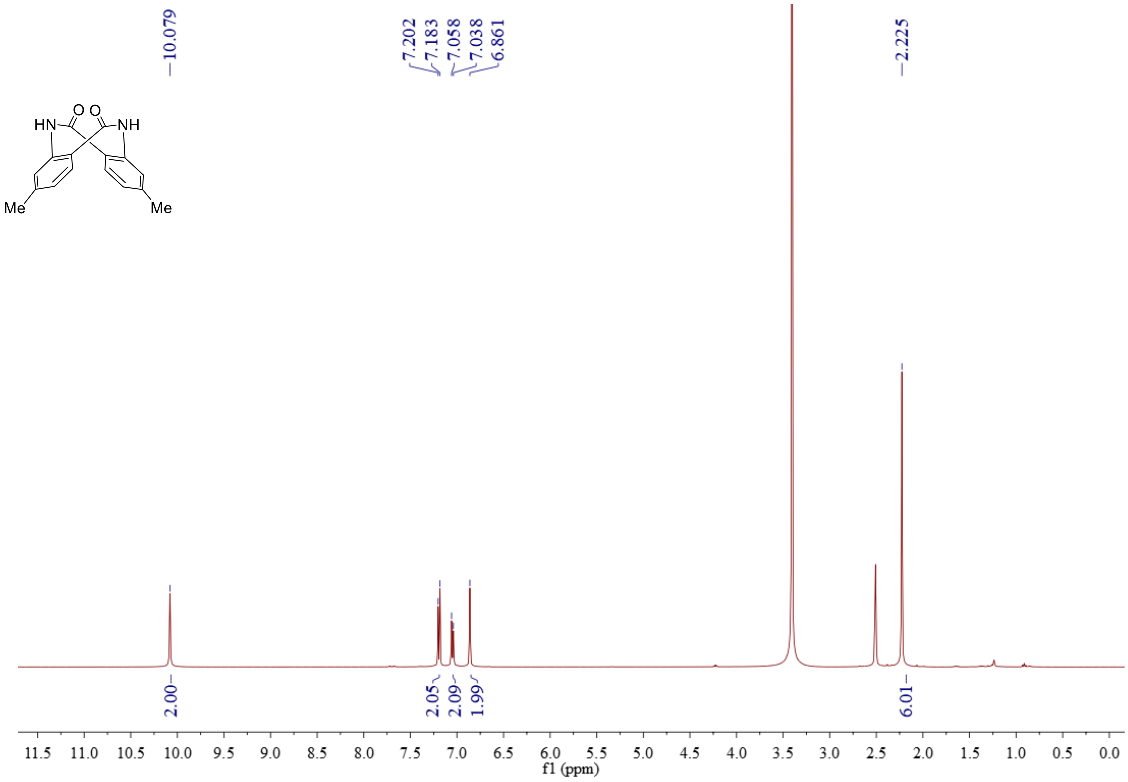

Supplementary Figure 8. ^{1}H NMR spectrum of compound **1i** ($(CD_3)_2SO$, 400 MHz)

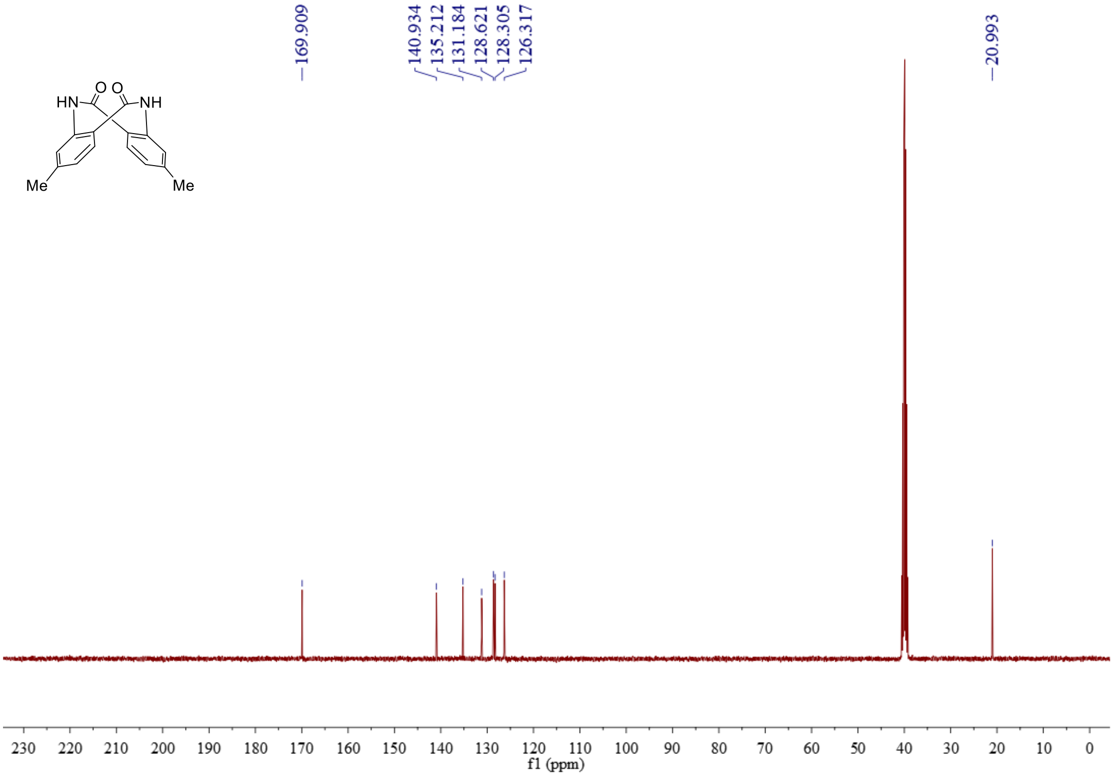

Supplementary Figure 9. ^{13}C NMR spectrum of compound **1i** ($(CD_3)_2SO$, 100 MHz)

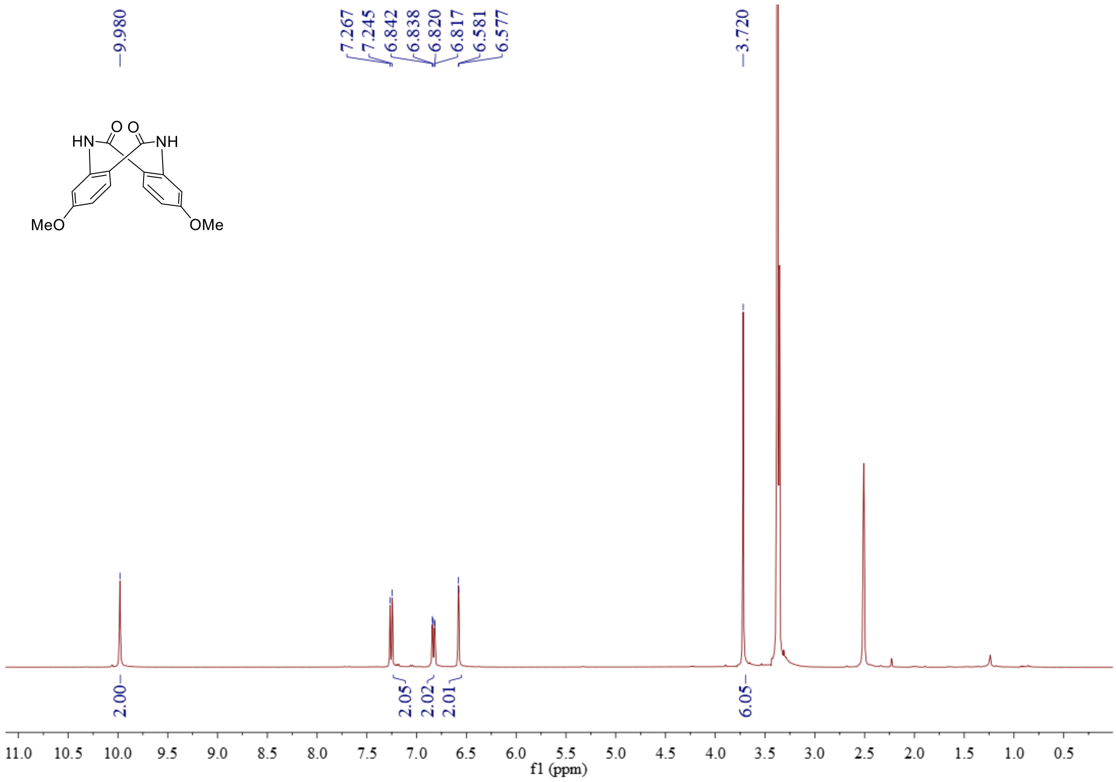

Supplementary Figure 10. ^{1}H NMR spectrum of compound **1j** ($(CD_3)_2SO$, 400 MHz)

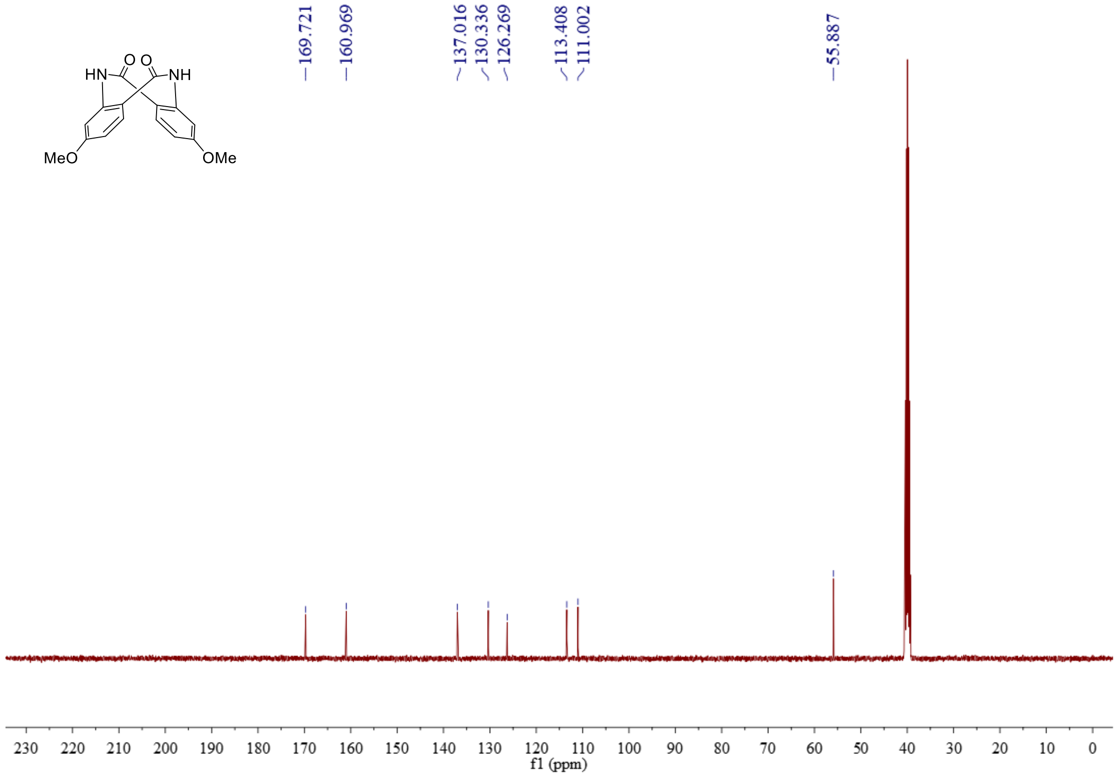

Supplementary Figure 11. ^{13}C NMR spectrum of compound **1j** ($(CD_3)_2SO$, 100 MHz)

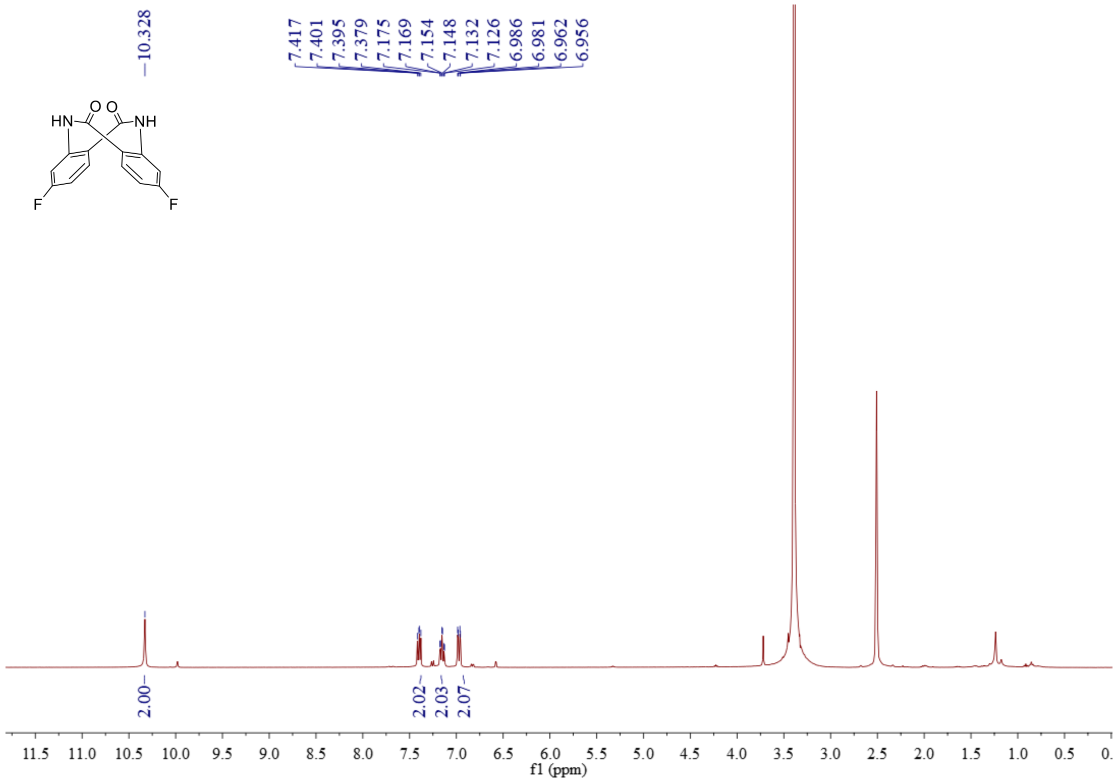

Supplementary Figure 12. ^{1}H NMR spectrum of compound **1l** ($(CD_3)_2SO$, 400 MHz)

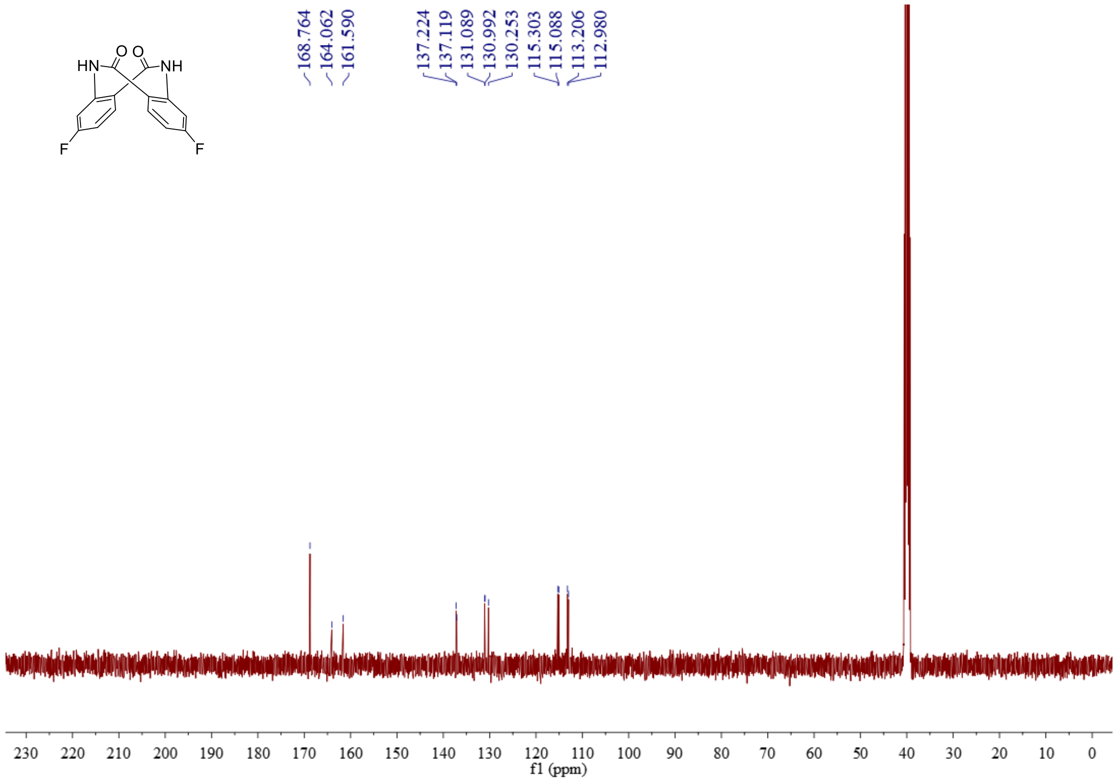

Supplementary Figure 13. ^{13}C NMR spectrum of compound **1l** ($(CD_3)_2SO$, 100 MHz)

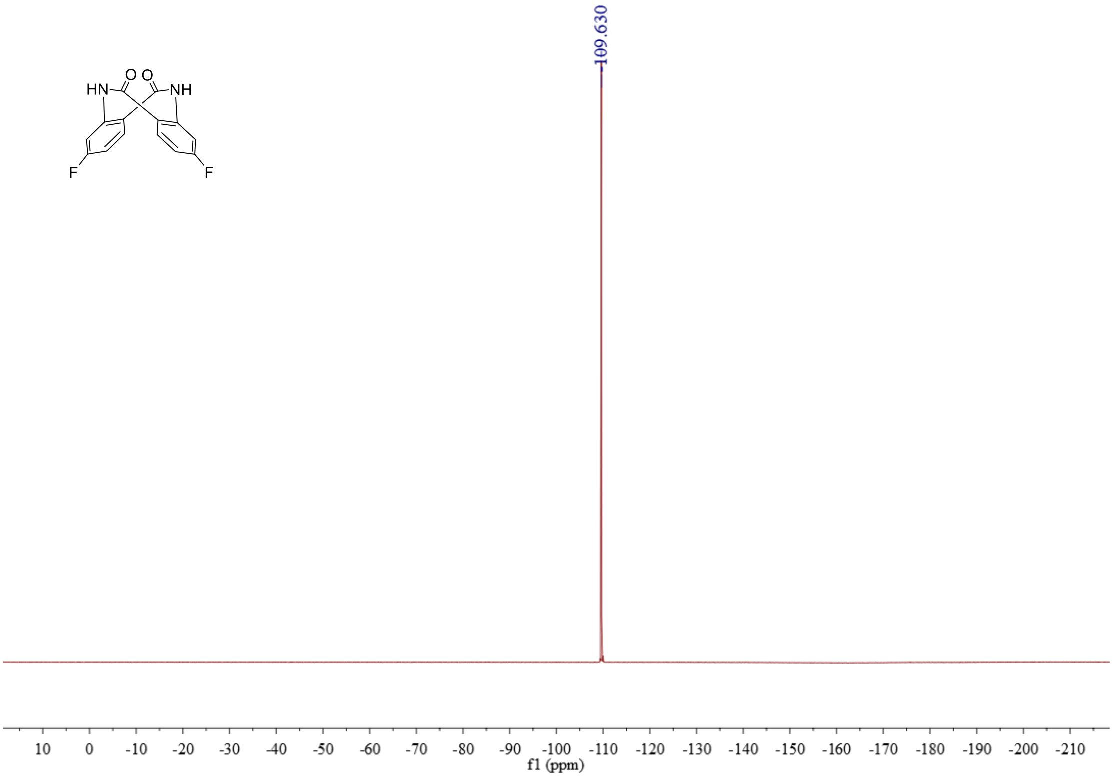

Supplementary Figure 14. ^{19}F NMR spectrum of compound **1l** ($(CD_3)_2SO$, 376 MHz)

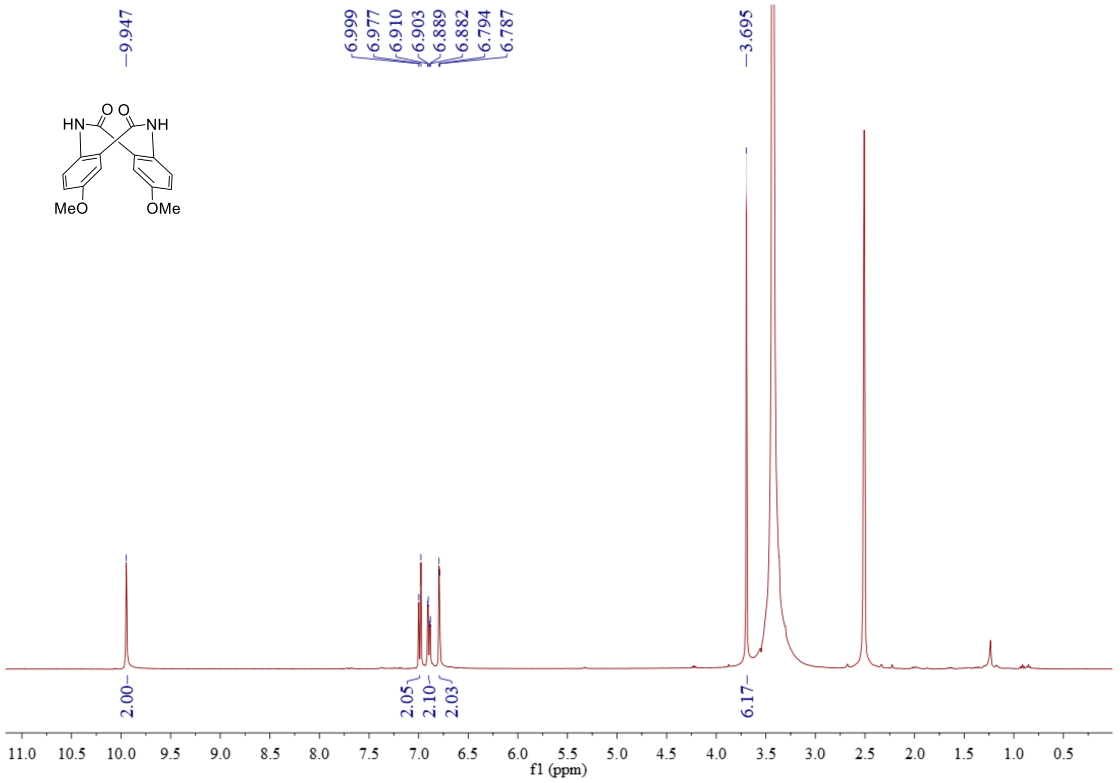

Supplementary Figure 15. ^{1}H NMR spectrum of compound **1p** ($(CD_3)_2SO$, 400 MHz)

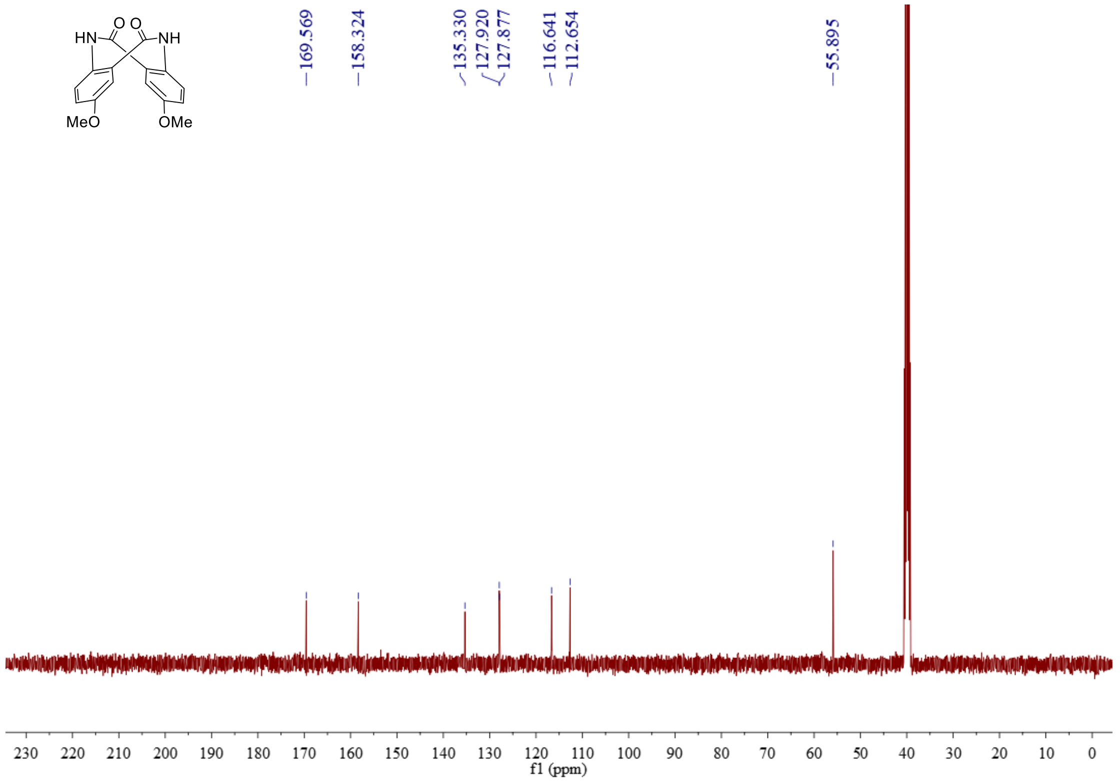

Supplementary Figure 16. ^{13}C NMR spectrum of compound **1p** ($(CD_3)_2SO$, 100 MHz)

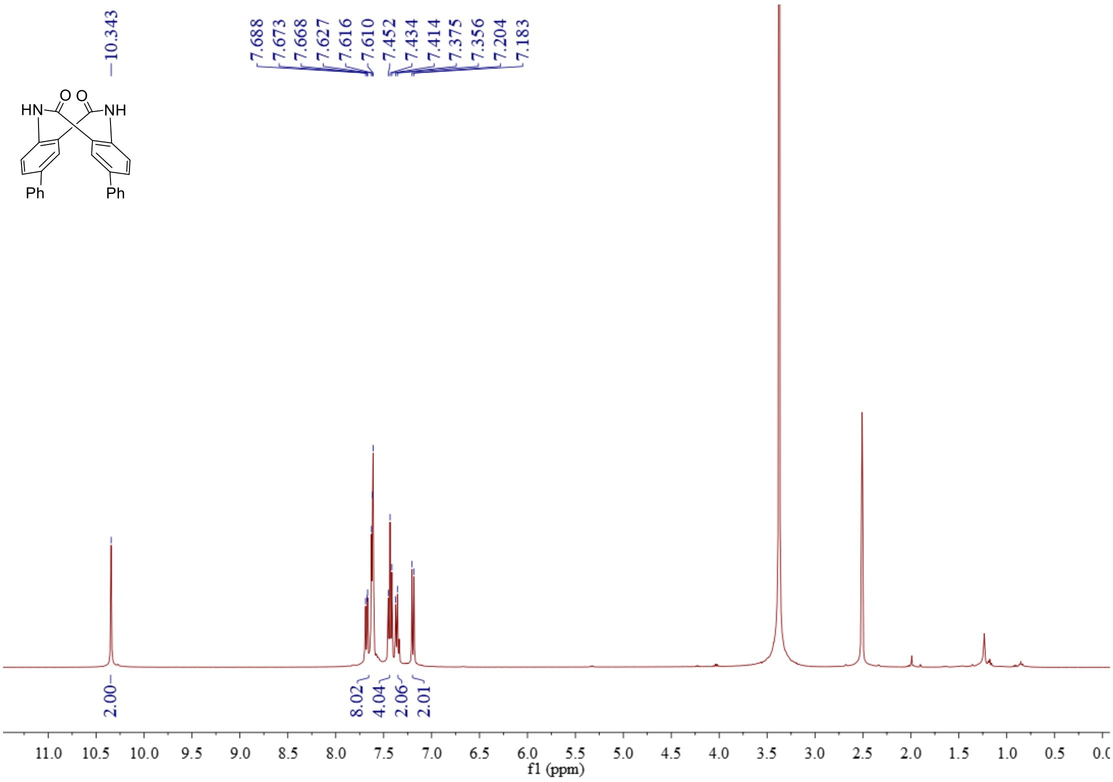

Supplementary Figure 17. ^{1}H NMR spectrum of compound **1u** ($(CD_3)_2SO$, 400 MHz)

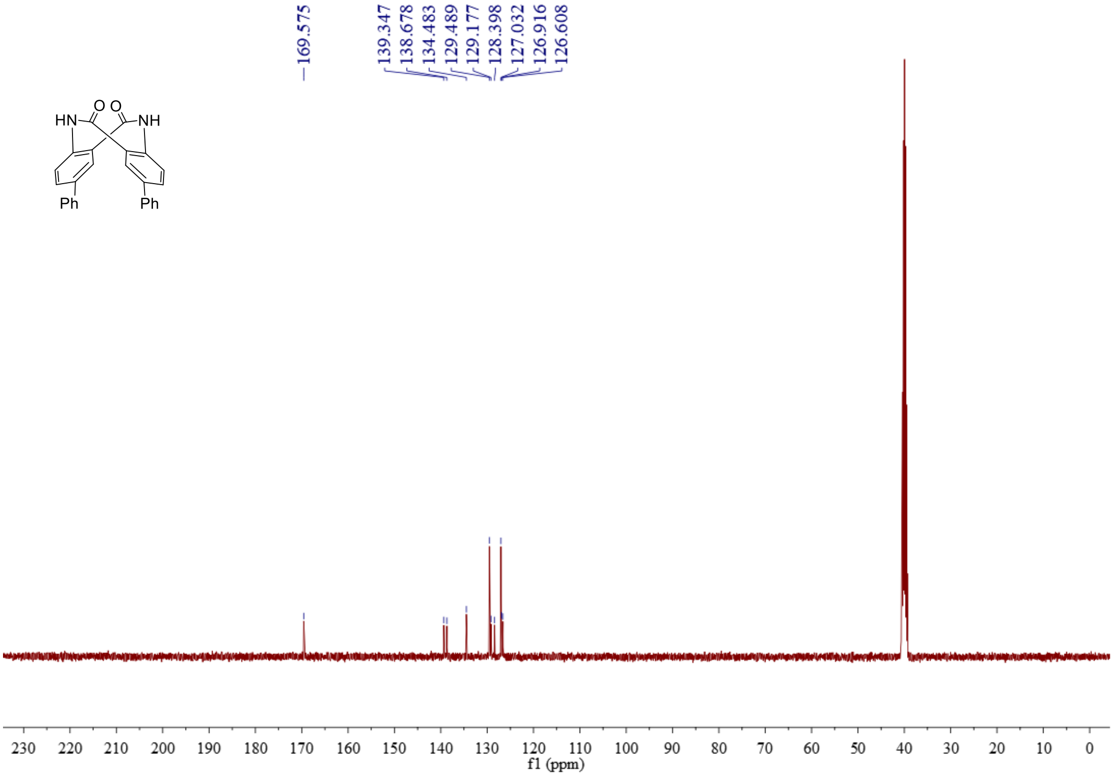

Supplementary Figure 18. ^{13}C NMR spectrum of compound **1u** ($(CD_3)_2SO$, 100 MHz)

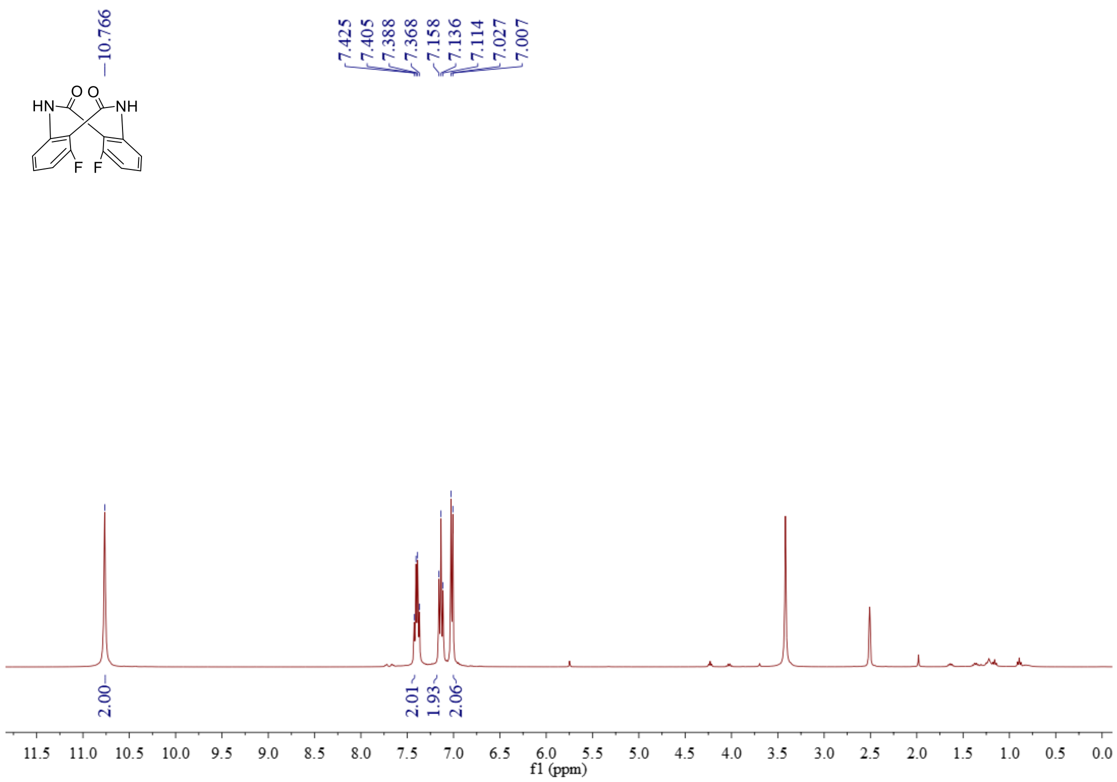

Supplementary Figure 19. ^{1}H NMR spectrum of compound **1v** ($(CD_3)_2SO$, 400 MHz)

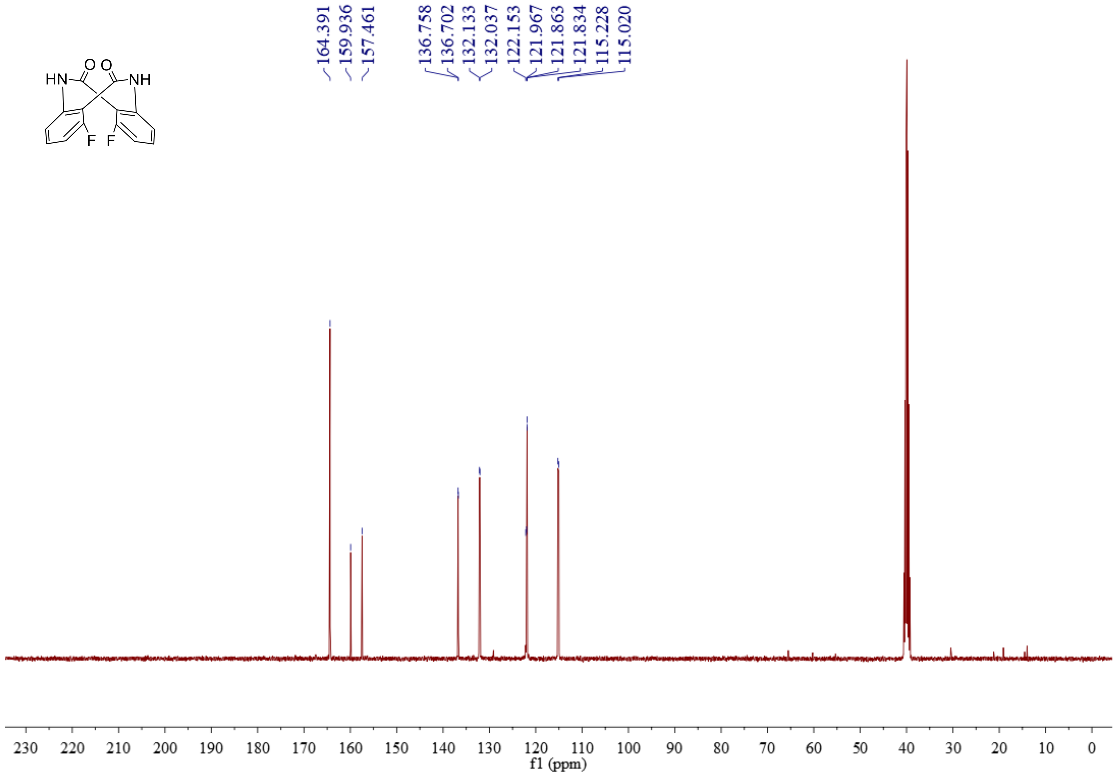

Supplementary Figure 20. ^{13}C NMR spectrum of compound **1v** ($(CD_3)_2SO$, 100 MHz)

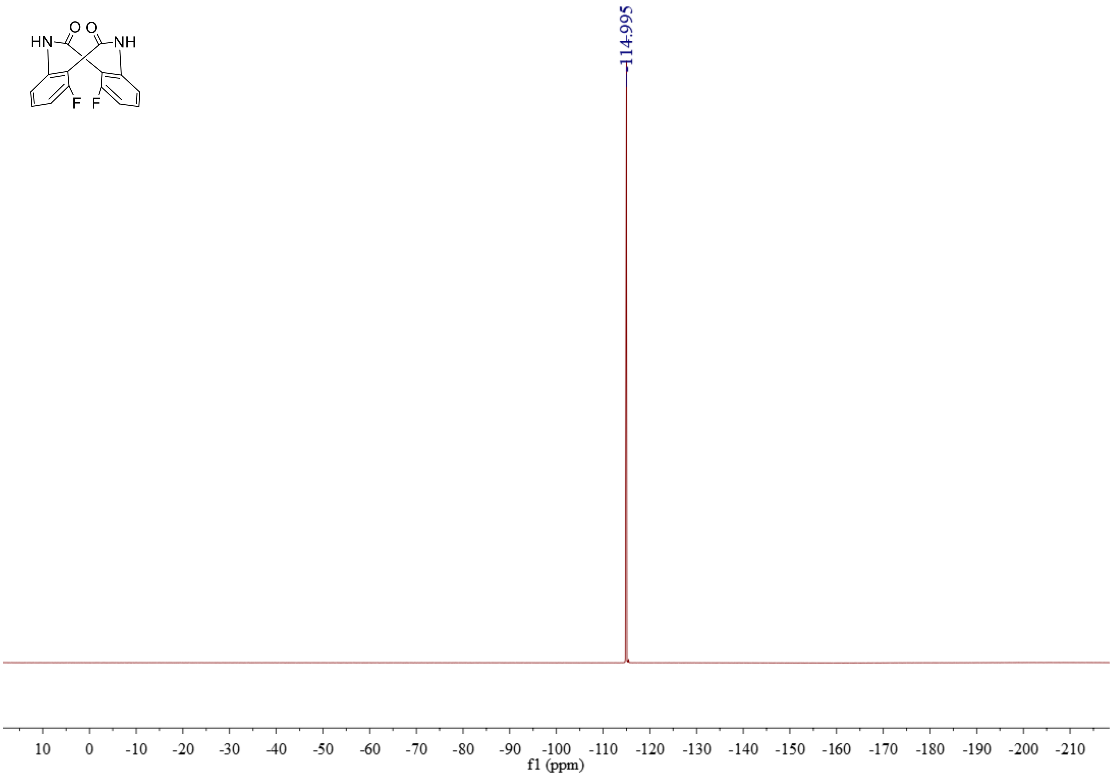

Supplementary Figure 21. ^{19}F NMR spectrum of compound **1v** (($(CD_3)_2SO$, 376 MHz)

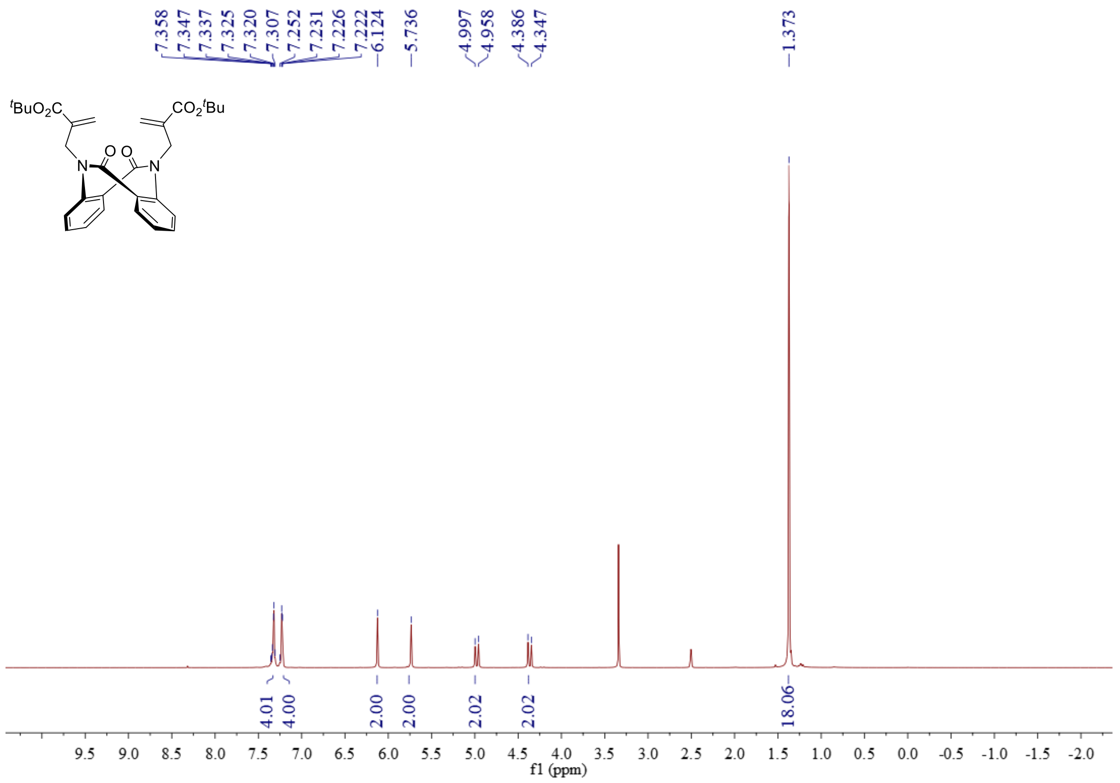

Supplementary Figure 22. ^{1}H NMR spectrum of compound **3a** ($(CD_3)_2SO$, 400 MHz)

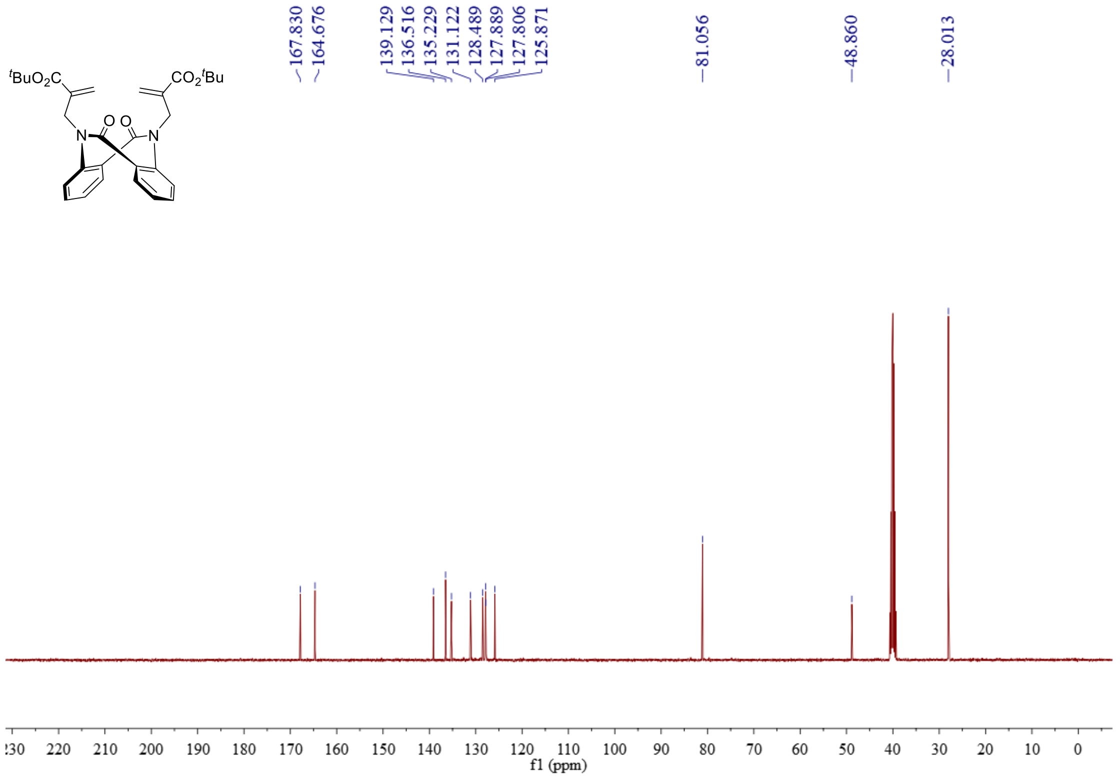

Supplementary Figure 23. ^{13}C NMR spectrum of compound **3a** ($(CD_3)_2SO$, 100 MHz)

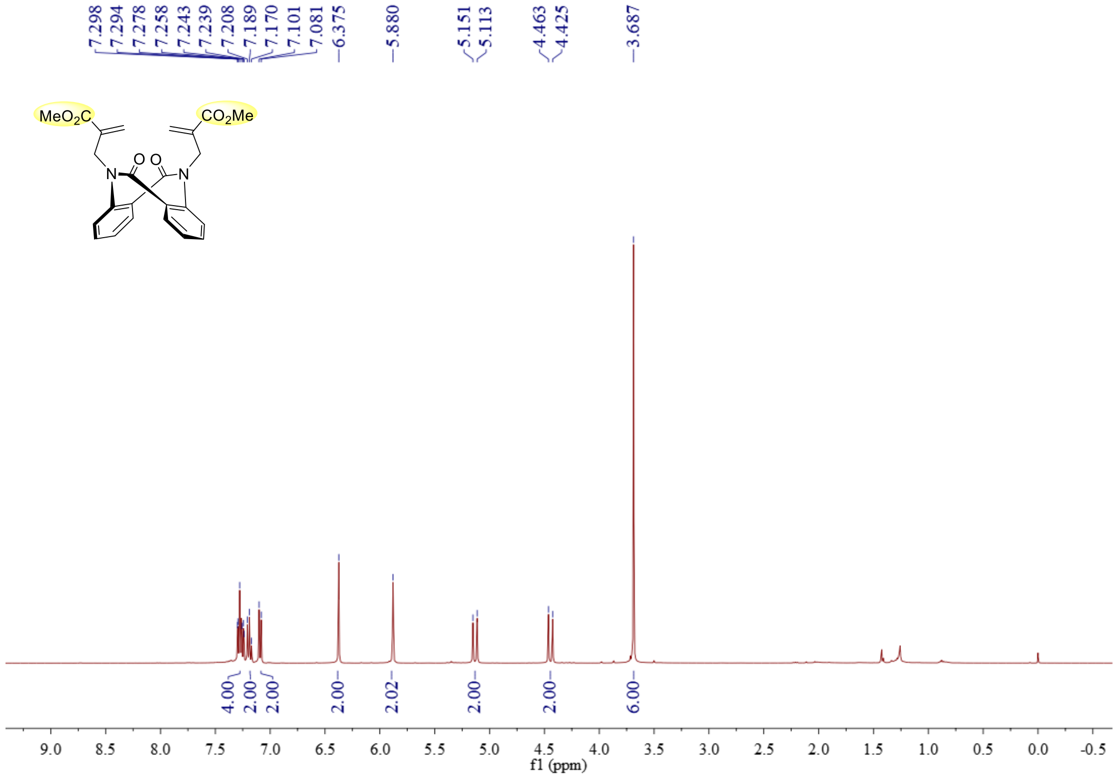

Supplementary Figure 24. ^{1}H NMR spectrum of compound **3b** ($CDCl_3$, 400 MHz)

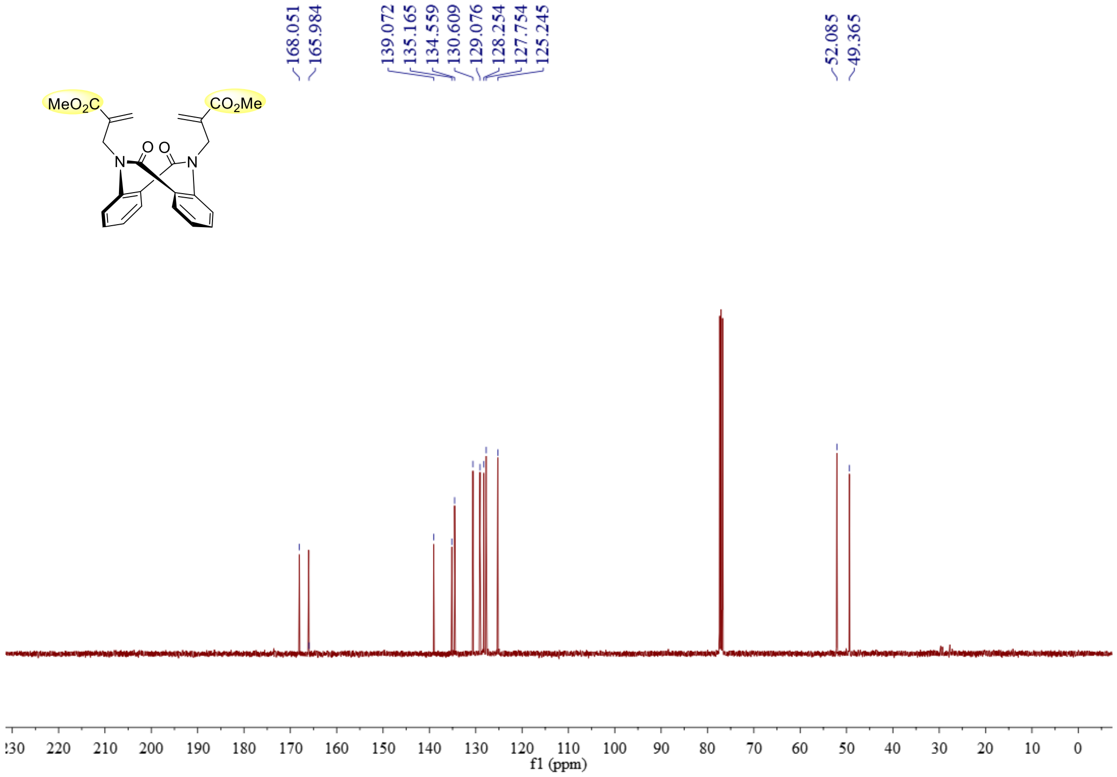

Supplementary Figure 25. ^{13}C NMR spectrum of compound **3b** ($CDCl_3$, 100 MHz)

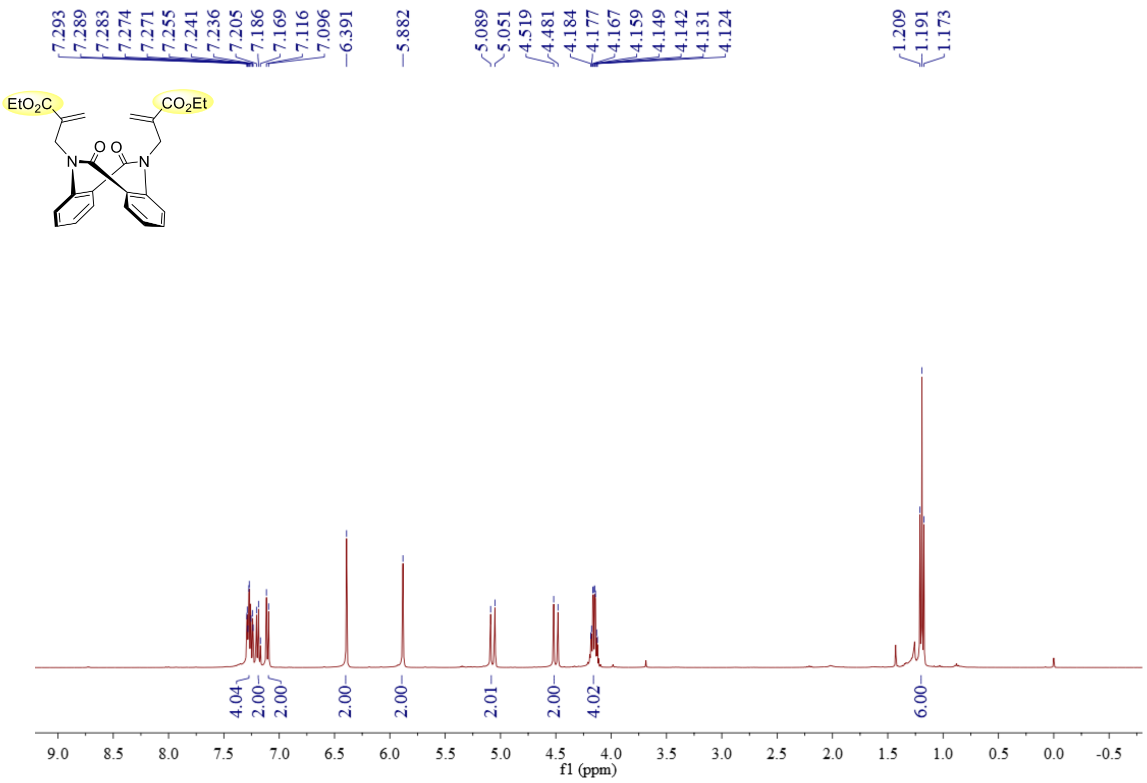

Supplementary Figure 26. ^{1}H NMR spectrum of compound **3c** ($CDCl_3$, 400 MHz)

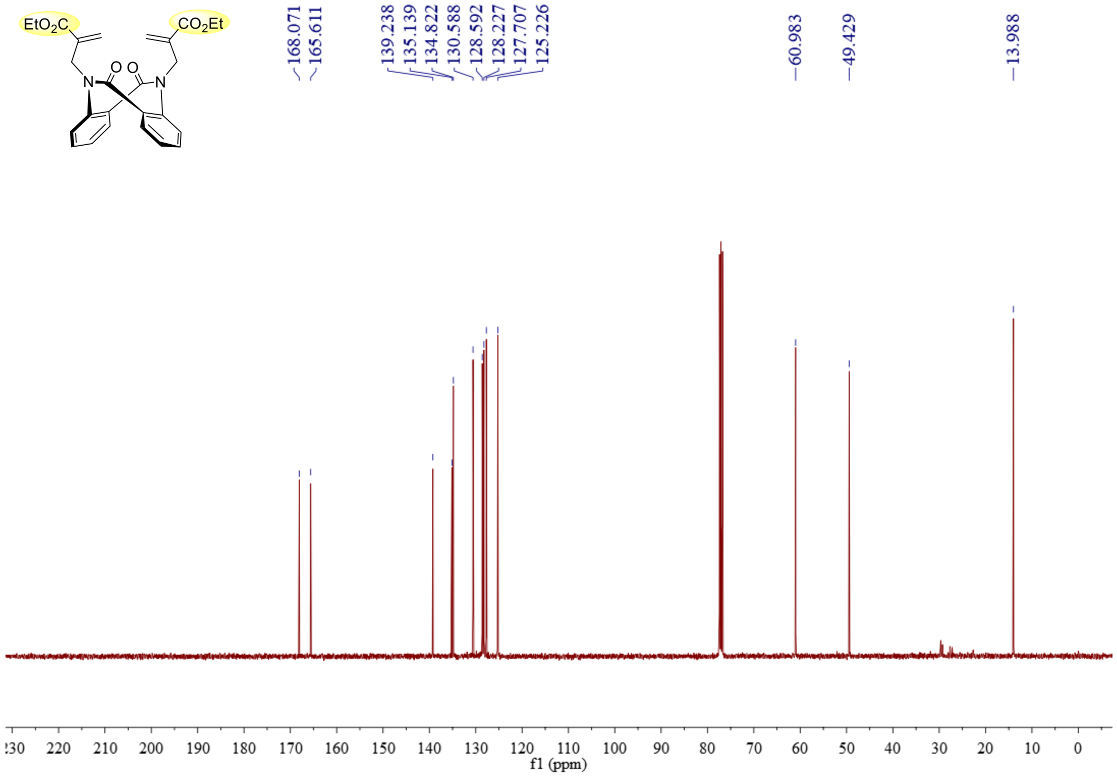

Supplementary Figure 27. ^{13}C NMR spectrum of compound **3c** ($CDCl_3$, 100 MHz)

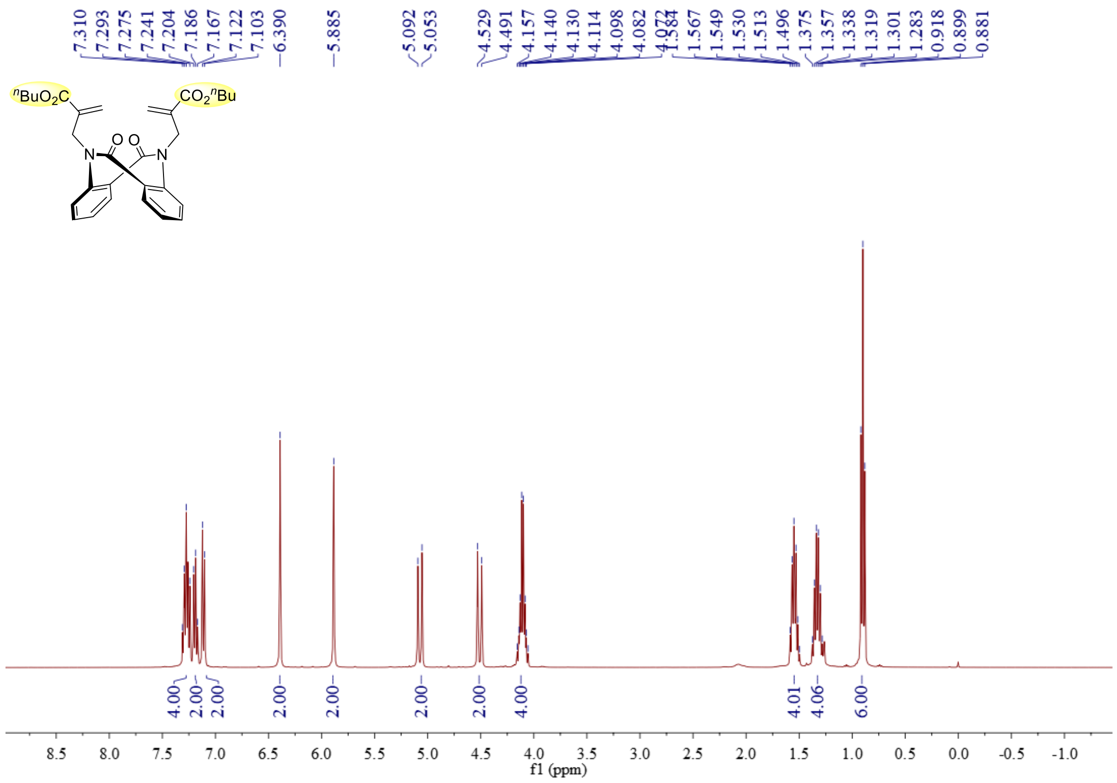

Supplementary Figure 28. ^{1}H NMR spectrum of compound **3d** ($CDCl_3$, 400 MHz)

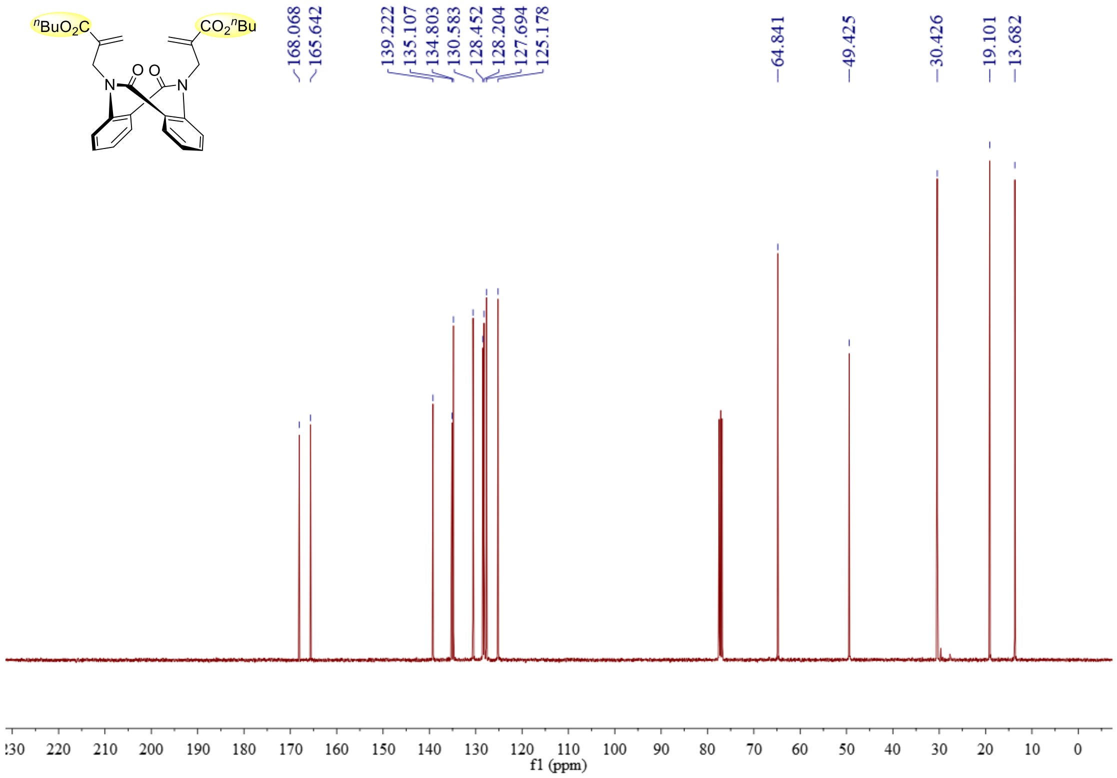

Supplementary Figure 29. ^{13}C NMR spectrum of compound **3d** ($CDCl_3$, 100 MHz)

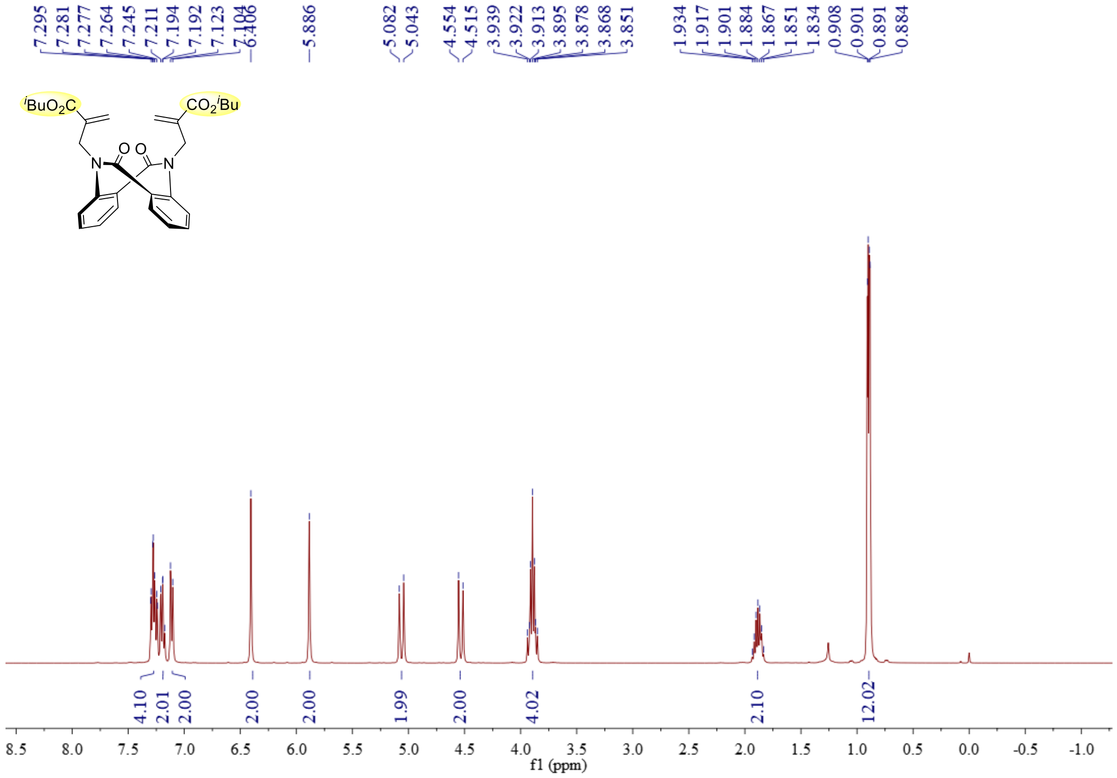

Supplementary Figure 30. ^{1}H NMR spectrum of compound **3e** ($CDCl_3$, 400 MHz)

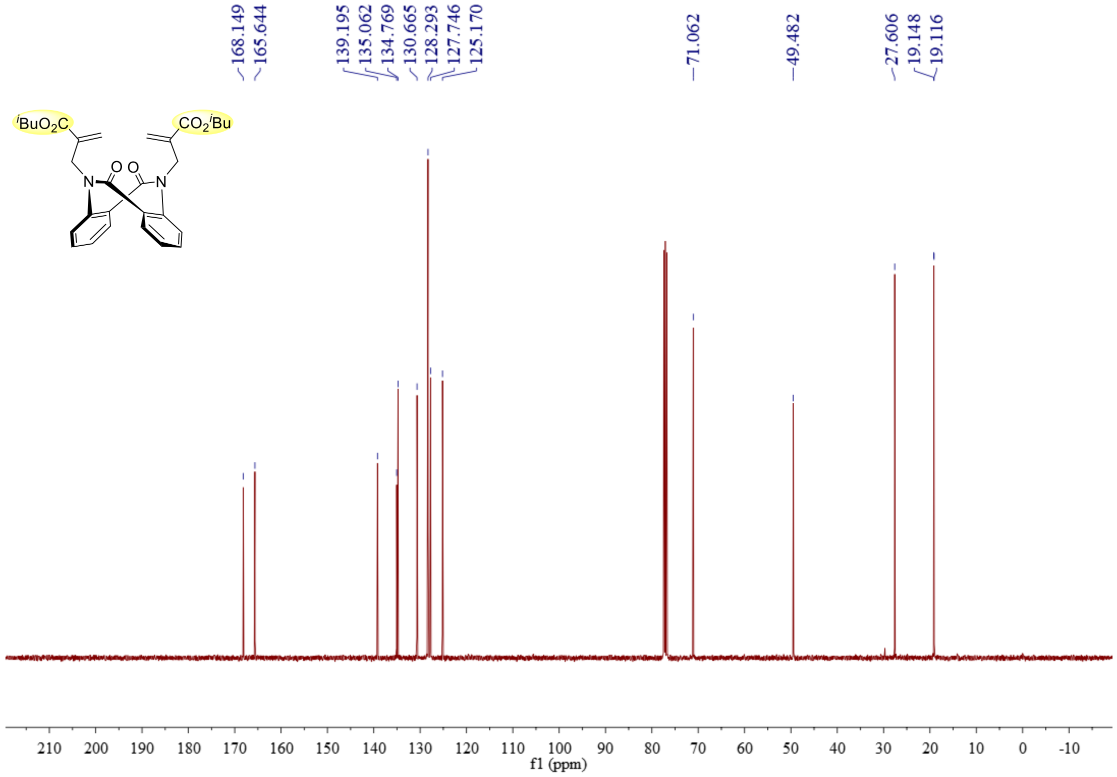

Supplementary Figure 31. ^{13}C NMR spectrum of compound **3e** ($CDCl_3$, 100 MHz)

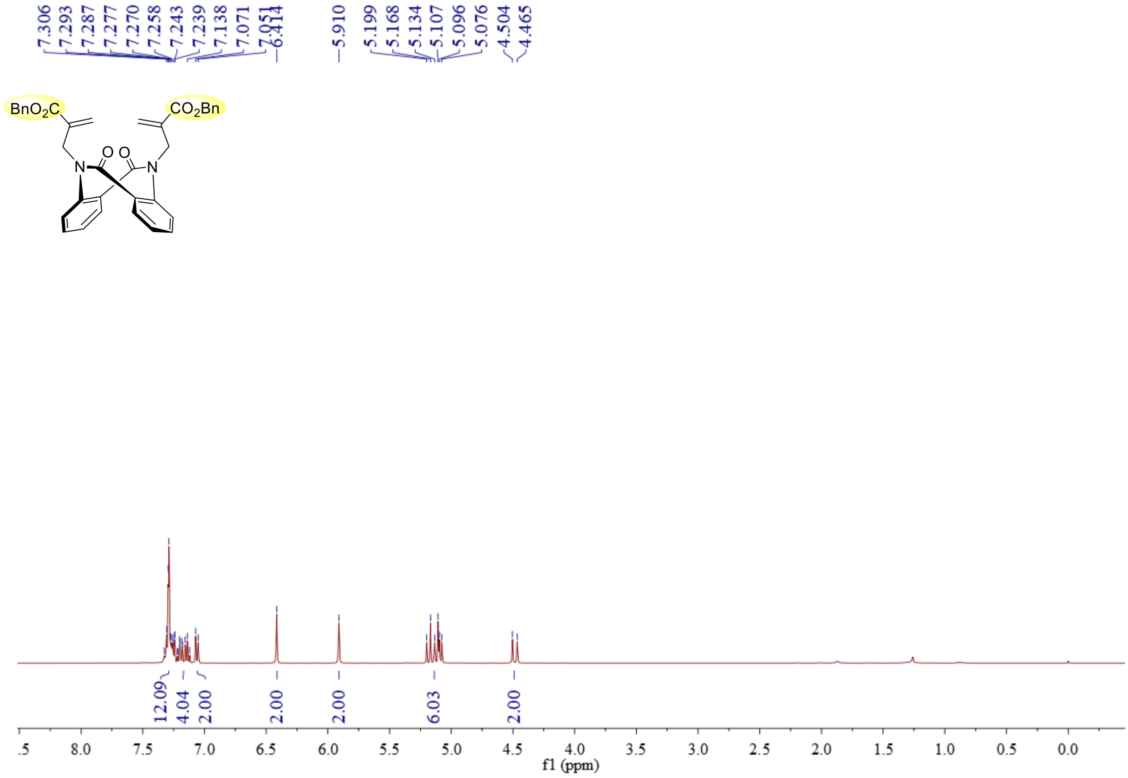

Supplementary Figure 32. ^{1}H NMR spectrum of compound **3f** ($CDCl_3$, 400 MHz)

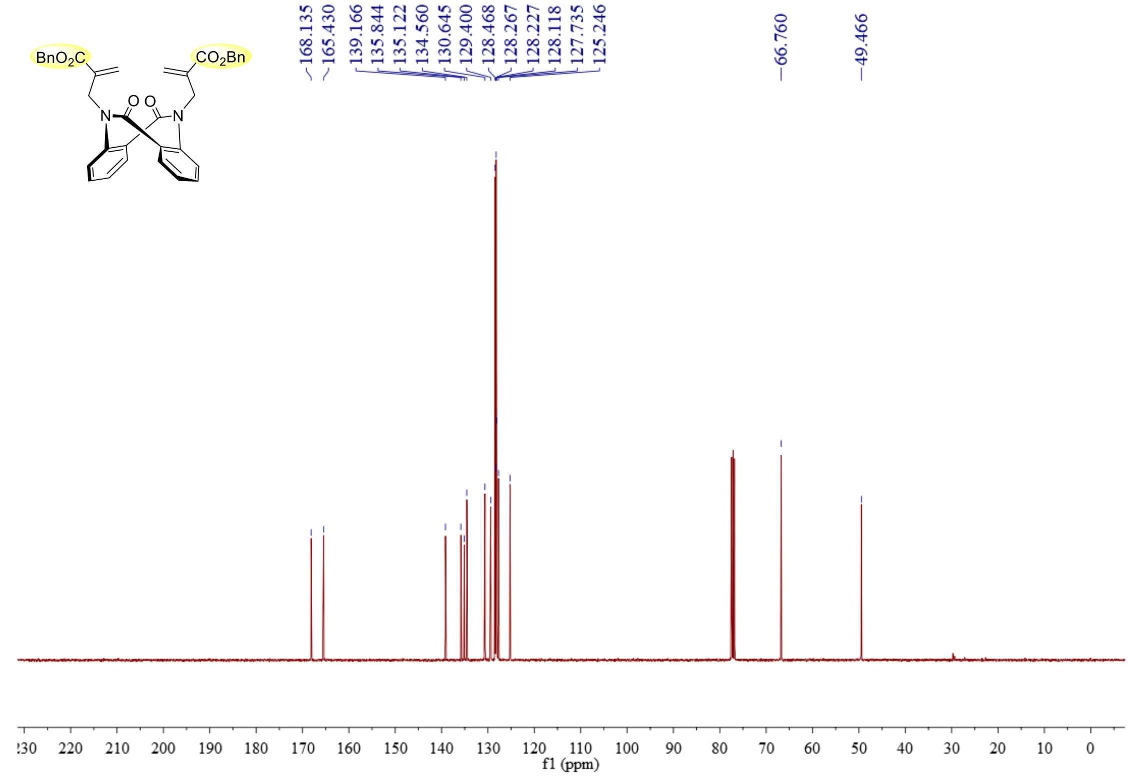

Supplementary Figure 33. ^{13}C NMR spectrum of compound **3f** ($CDCl_3$, 100 MHz)

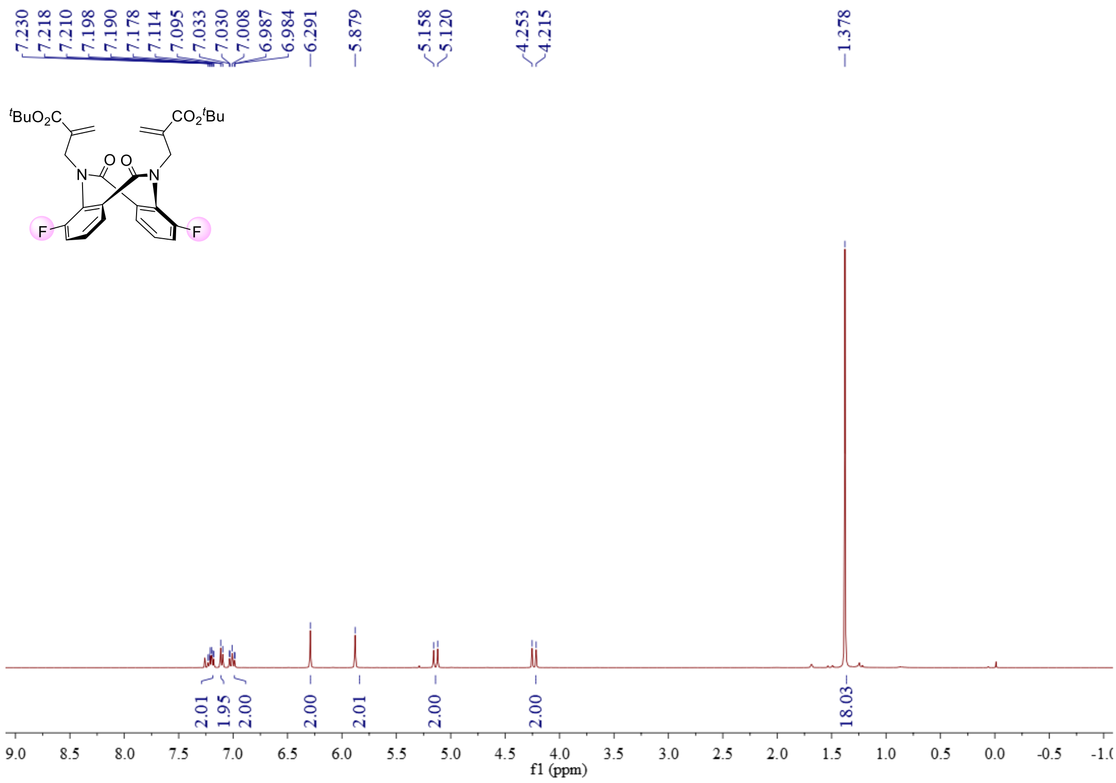

Supplementary Figure 34. ^{1}H NMR spectrum of compound **3g** ($CDCl_3$, 400 MHz)

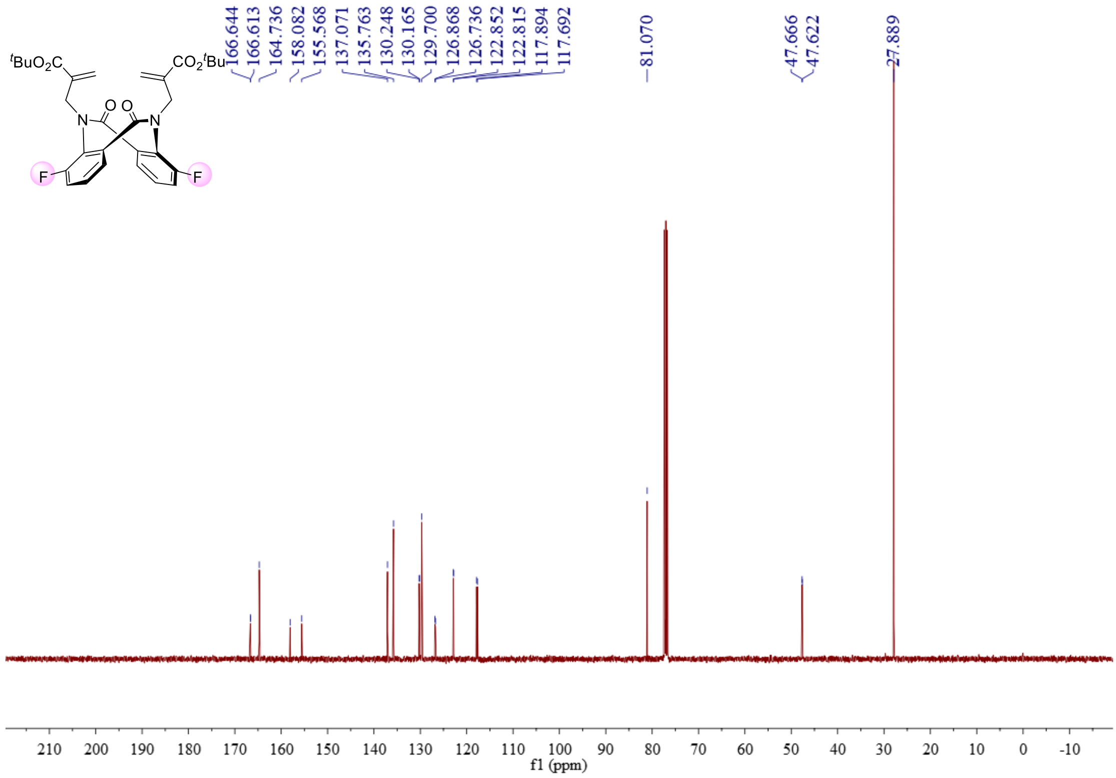

Supplementary Figure 35. ^{13}C NMR spectrum of compound **3g** ($CDCl_3$, 100 MHz)

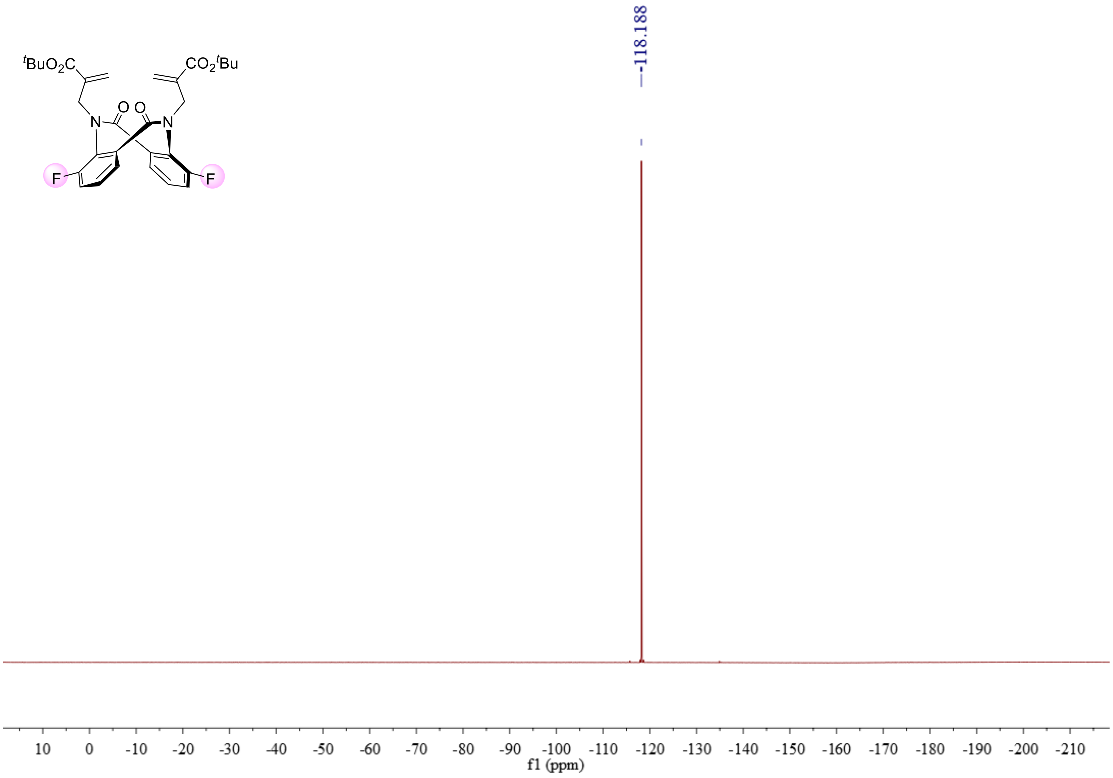

Supplementary Figure 36. ^{19}F NMR spectrum of compound **3g** ($CDCl_3$, 376 MHz)

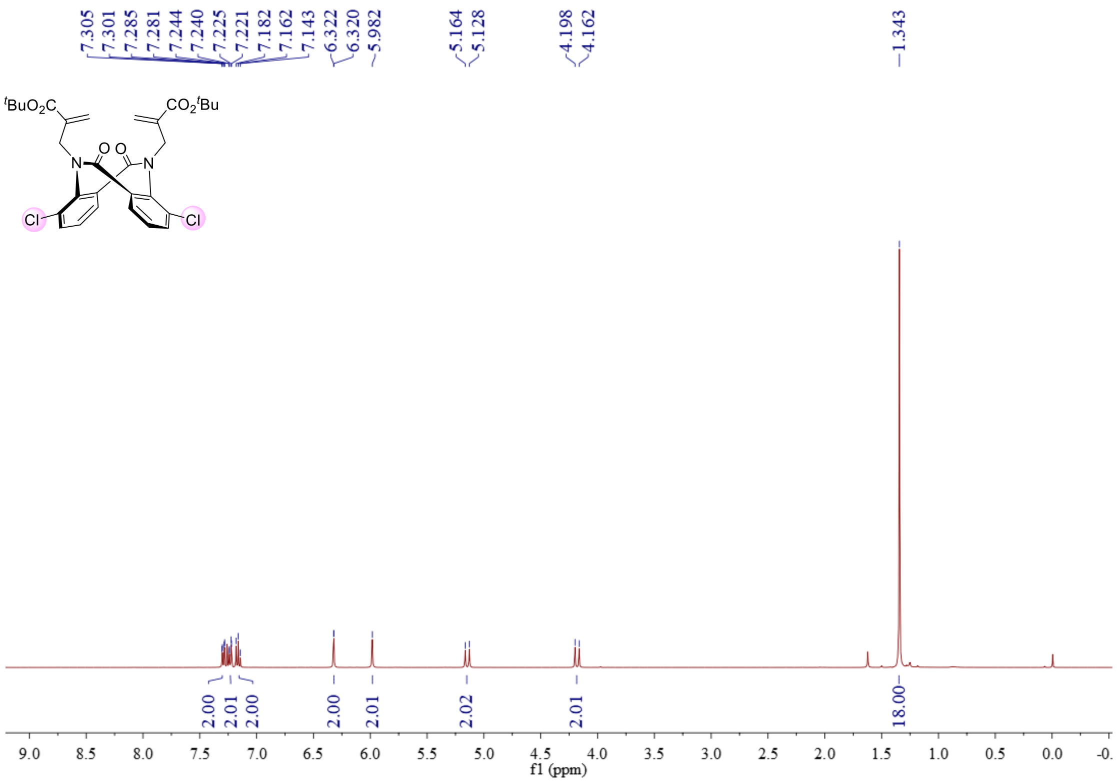

Supplementary Figure 37. ^{1}H NMR spectrum of compound **3h** ($CDCl_3$, 400 MHz)

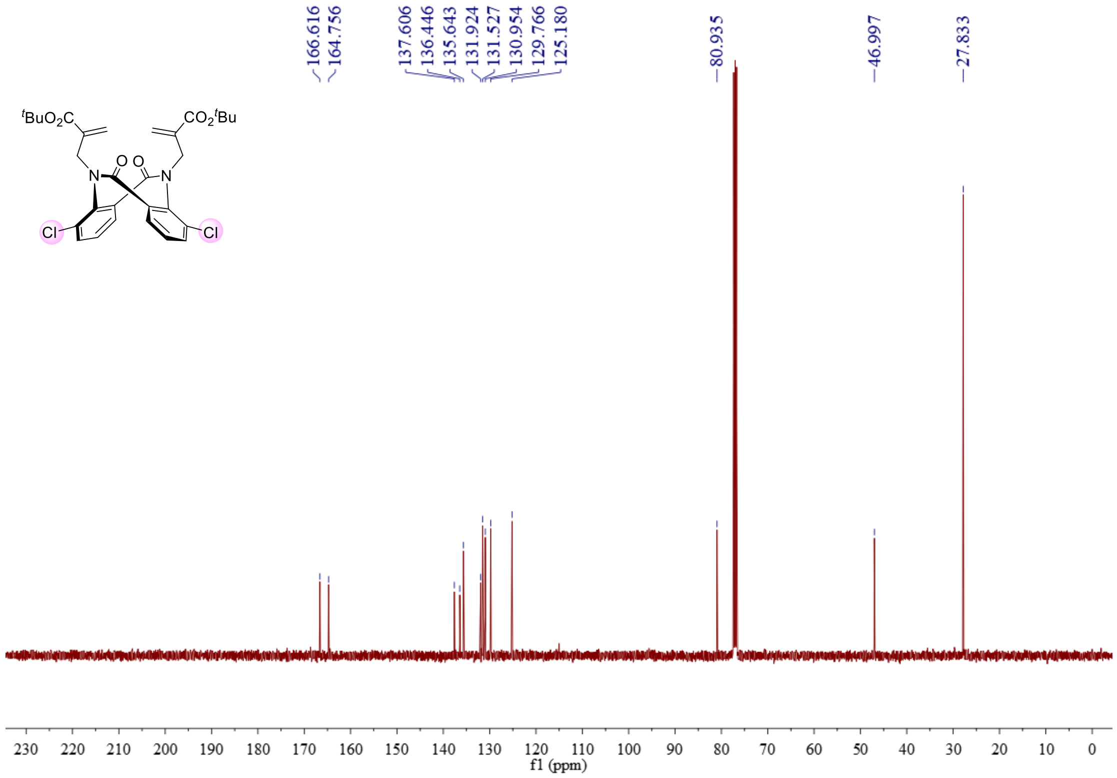

Supplementary Figure 38. ^{13}C NMR spectrum of compound **3h** ($CDCl_3$, 100 MHz)

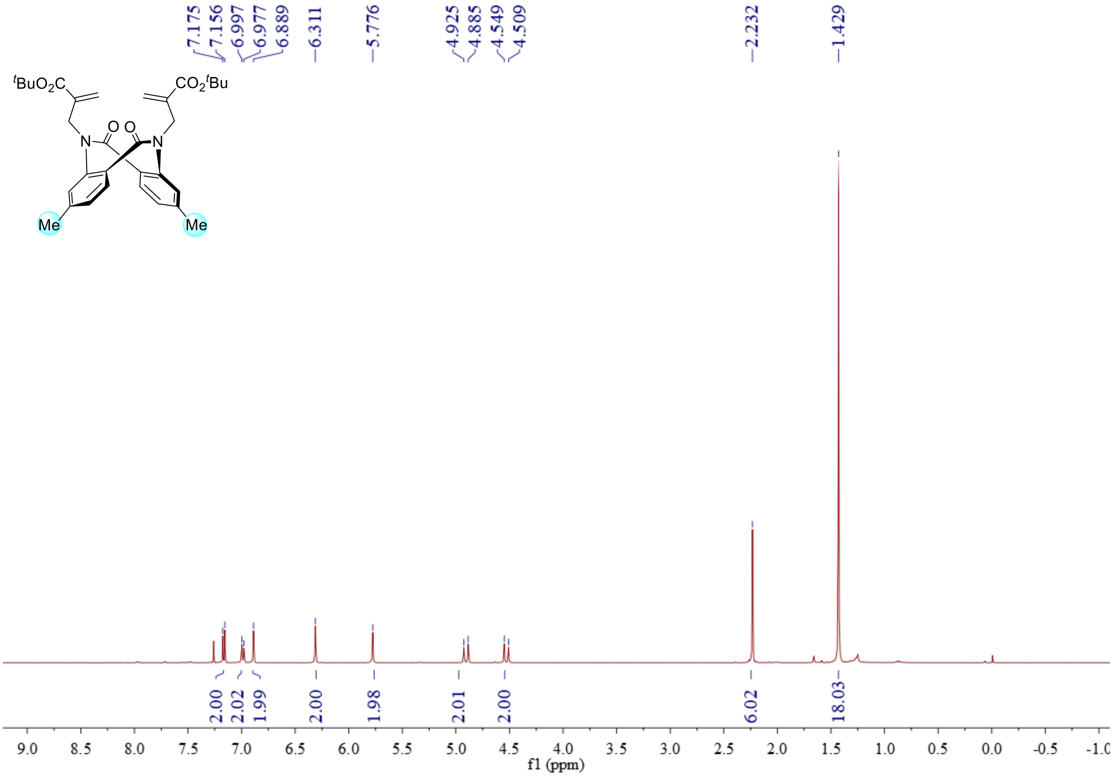

Supplementary Figure 39. ^{1}H NMR spectrum of compound **3i** ($CDCl_3$, 400 MHz)

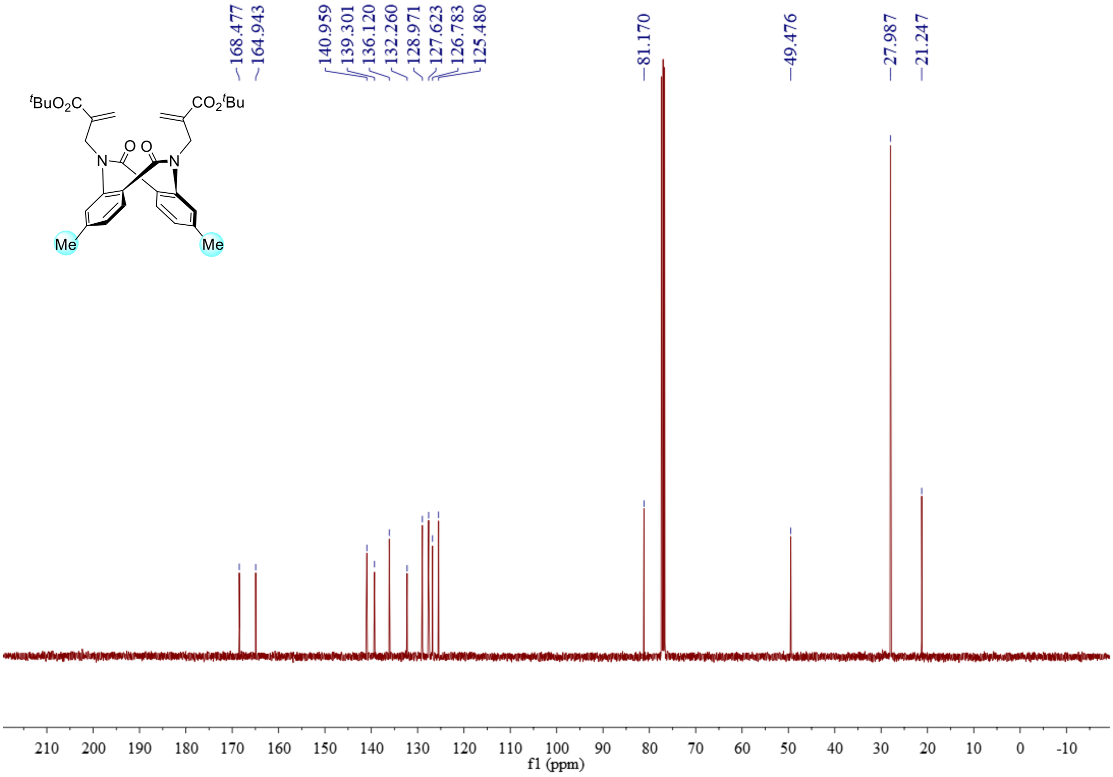

Supplementary Figure 40. ^{13}C NMR spectrum of compound **3i** ($CDCl_3$, 100 MHz)

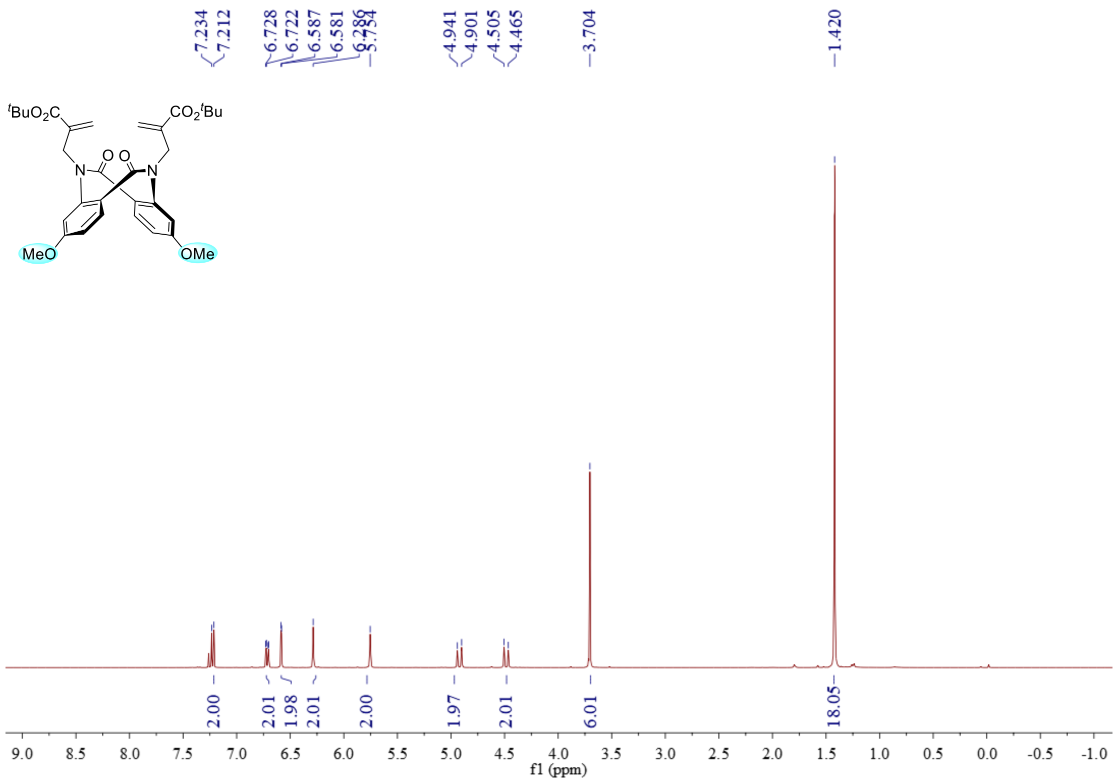

Supplementary Figure 41. ^{1}H NMR spectrum of compound **3j** ($CDCl_3$, 400 MHz)

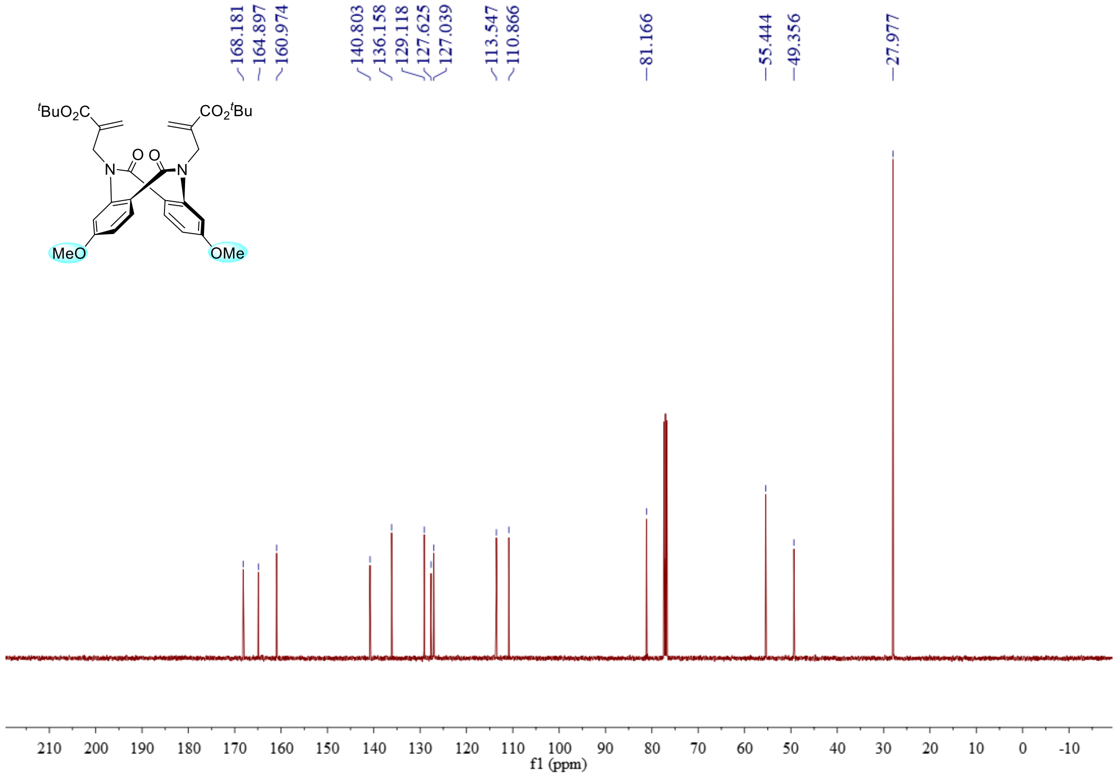

Supplementary Figure 42. ^{13}C NMR spectrum of compound **3j** ($CDCl_3$, 100 MHz)

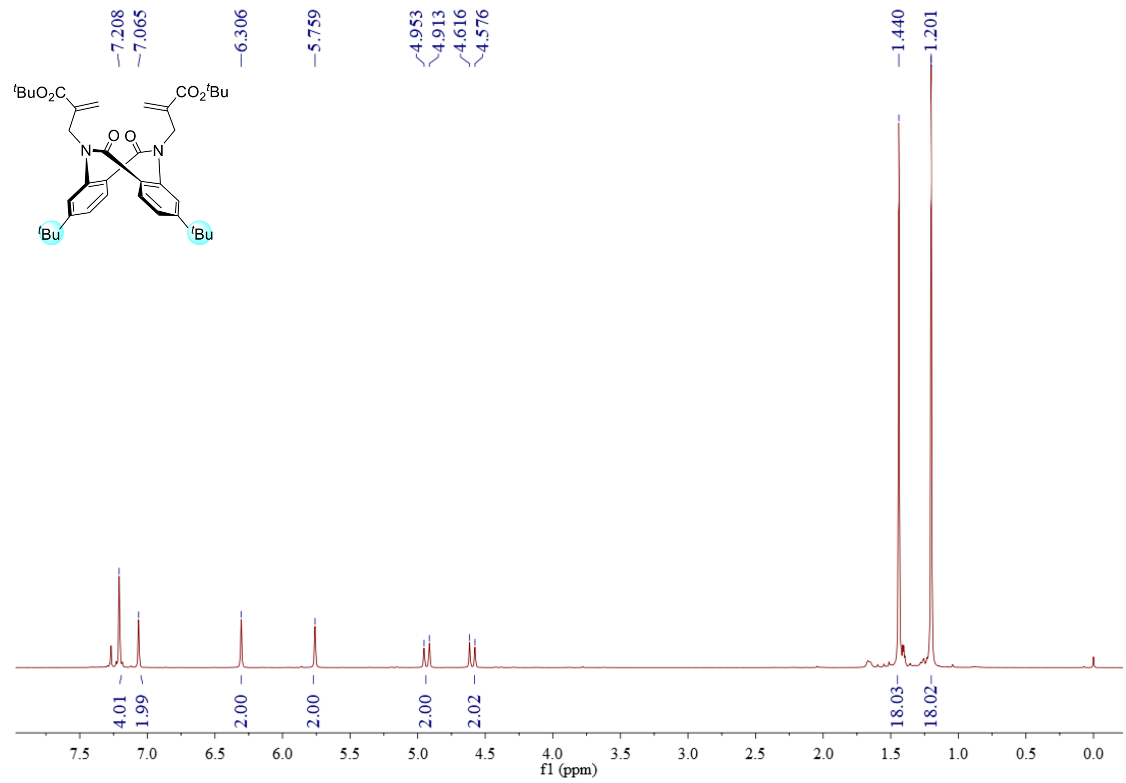

Supplementary Figure 43. ^{1}H NMR spectrum of compound **3k** ($CDCl_3$, 400 MHz)

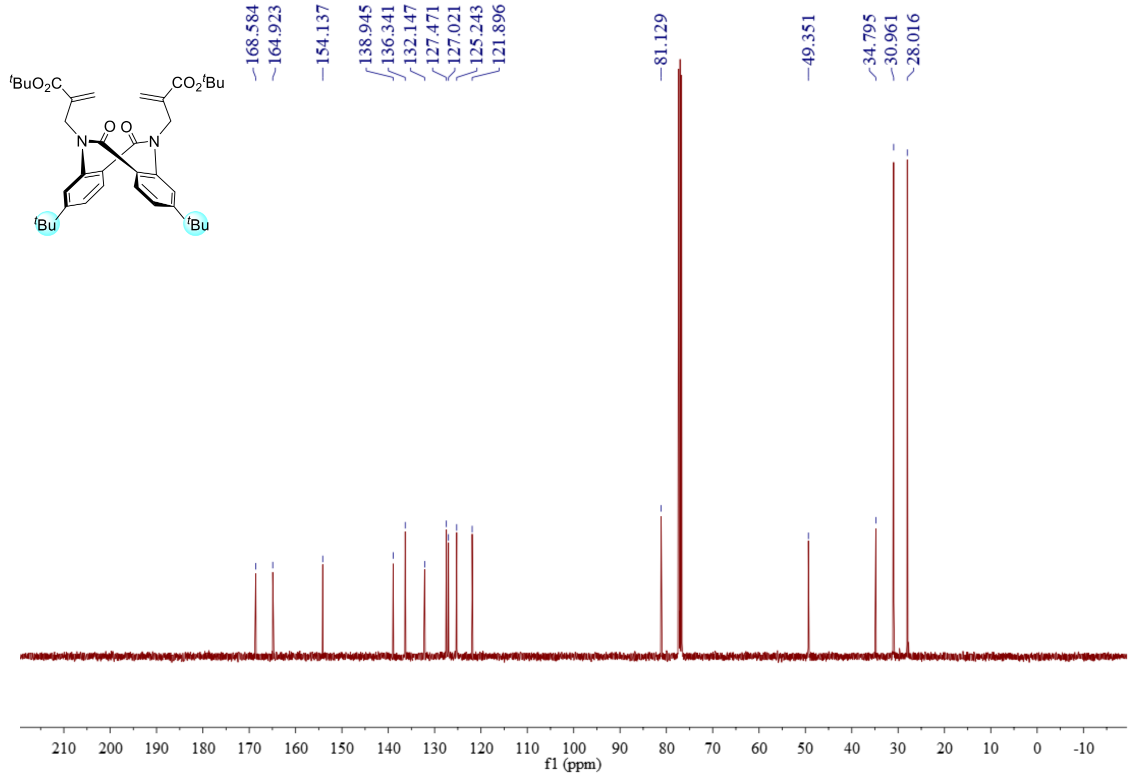

Supplementary Figure 44. ^{13}C NMR spectrum of compound **3k** ($CDCl_3$, 100 MHz)

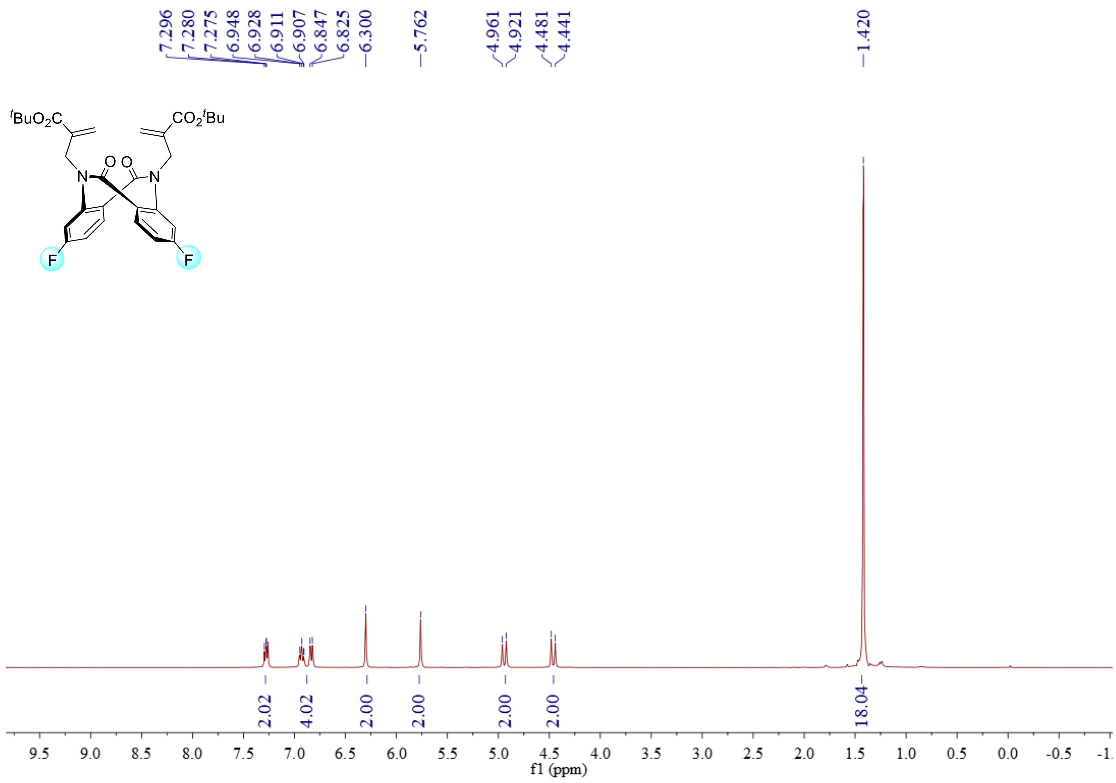

Supplementary Figure 45. ^{1}H NMR spectrum of compound **3l** ($CDCl_3$, 400 MHz)

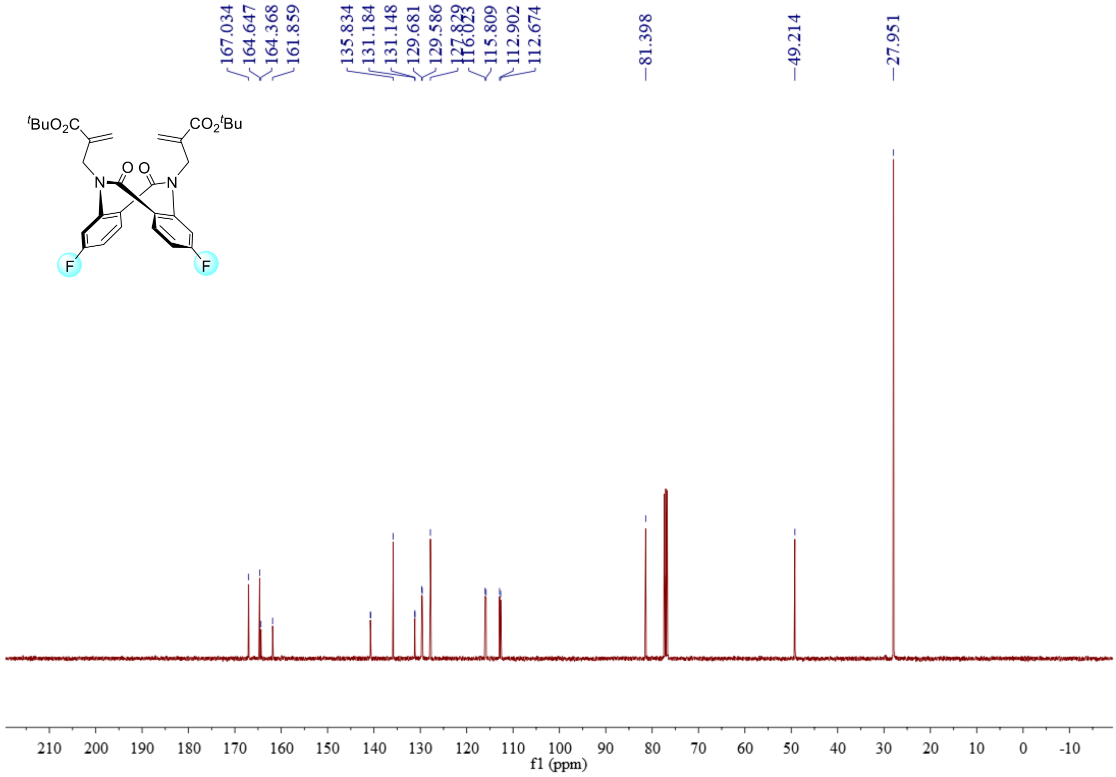

Supplementary Figure 46. ^{13}C NMR spectrum of compound **3l** ($CDCl_3$, 100 MHz)

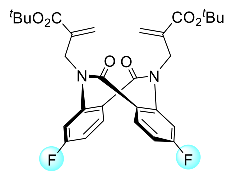

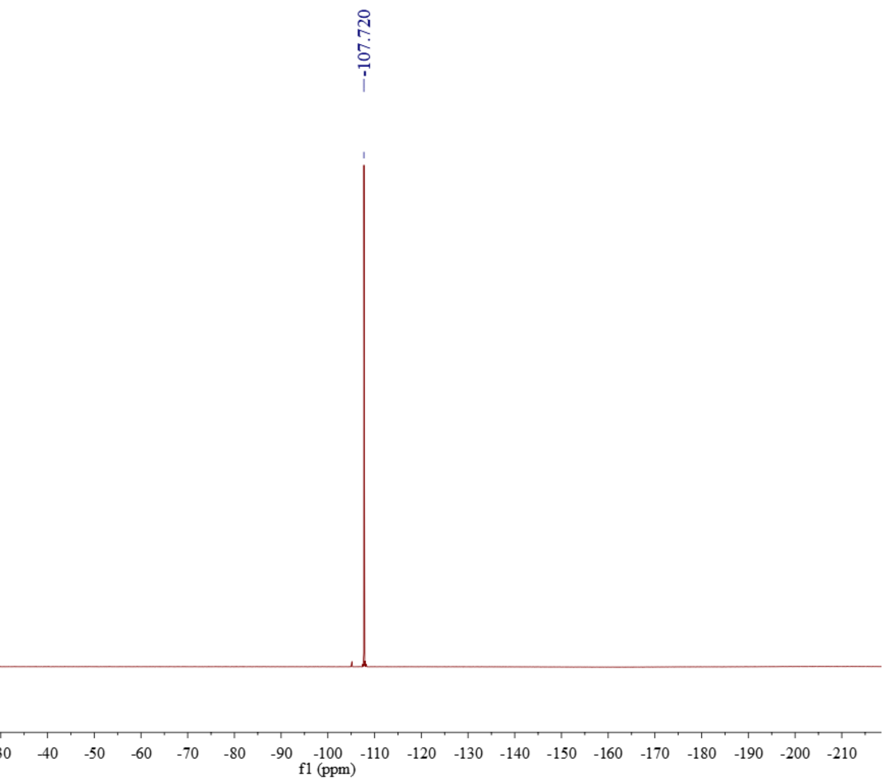

Supplementary Figure 47. ^{19}F NMR spectrum of compound **3l** ($CDCl_3$, 376 MHz)

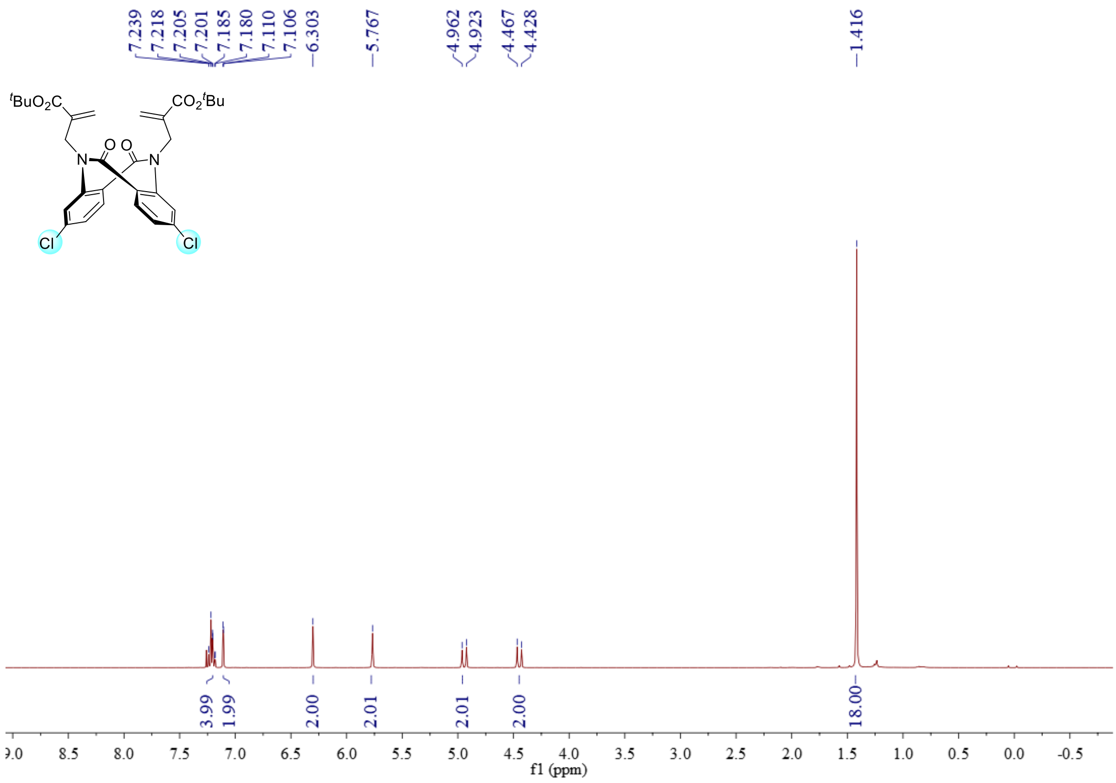

Supplementary Figure 48. ^{1}H NMR spectrum of compound **3m** ($CDCl_3$, 400 MHz)

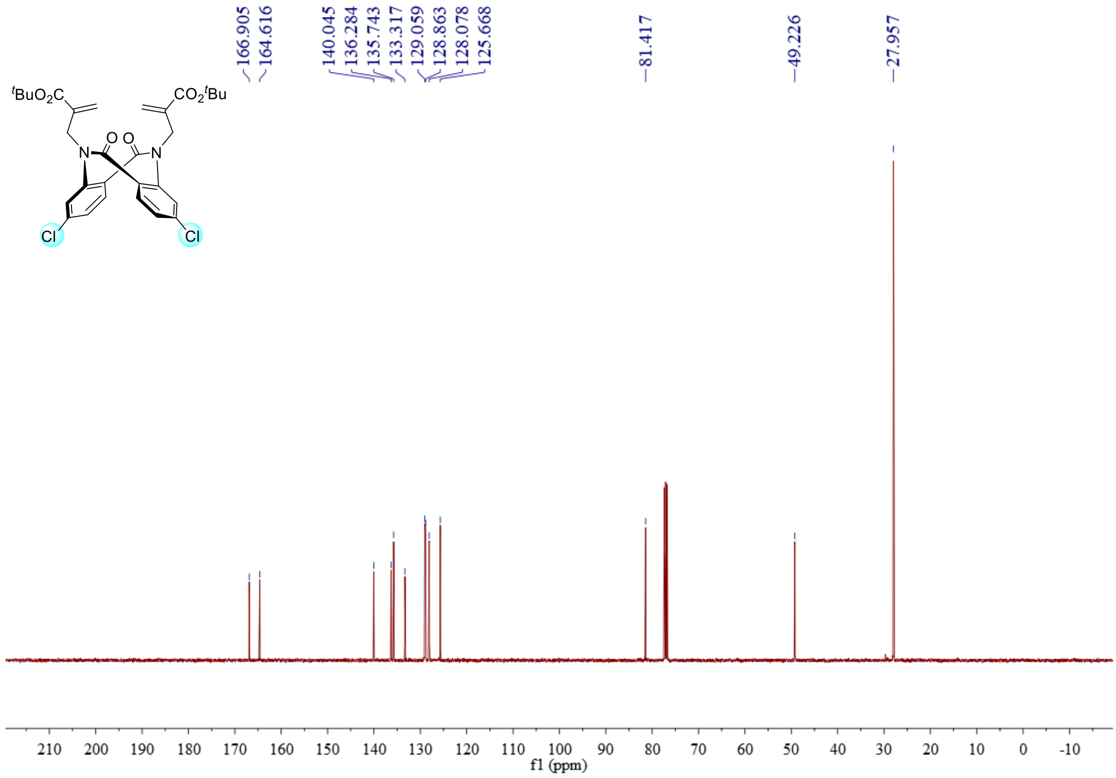

Supplementary Figure 49. ^{13}C NMR spectrum of compound **3m** ($CDCl_3$, 100 MHz)

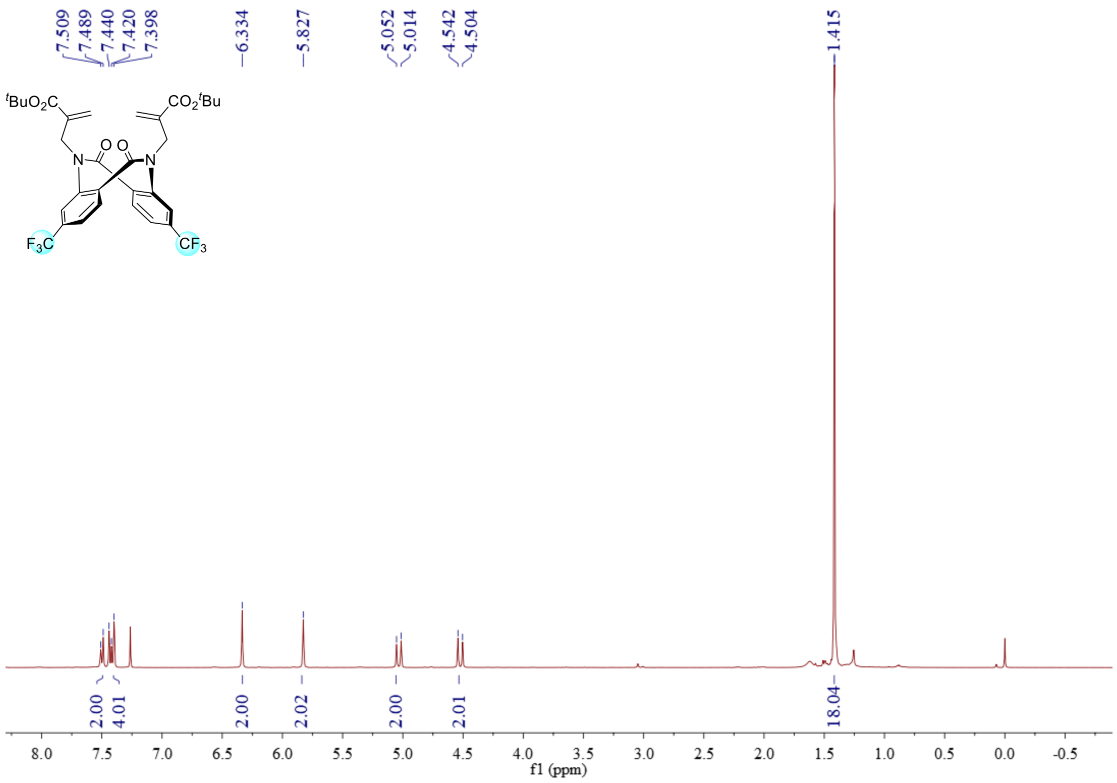

Supplementary Figure 50. ^1H NMR spectrum of compound **3n** ($CDCl_3$, 400 MHz)

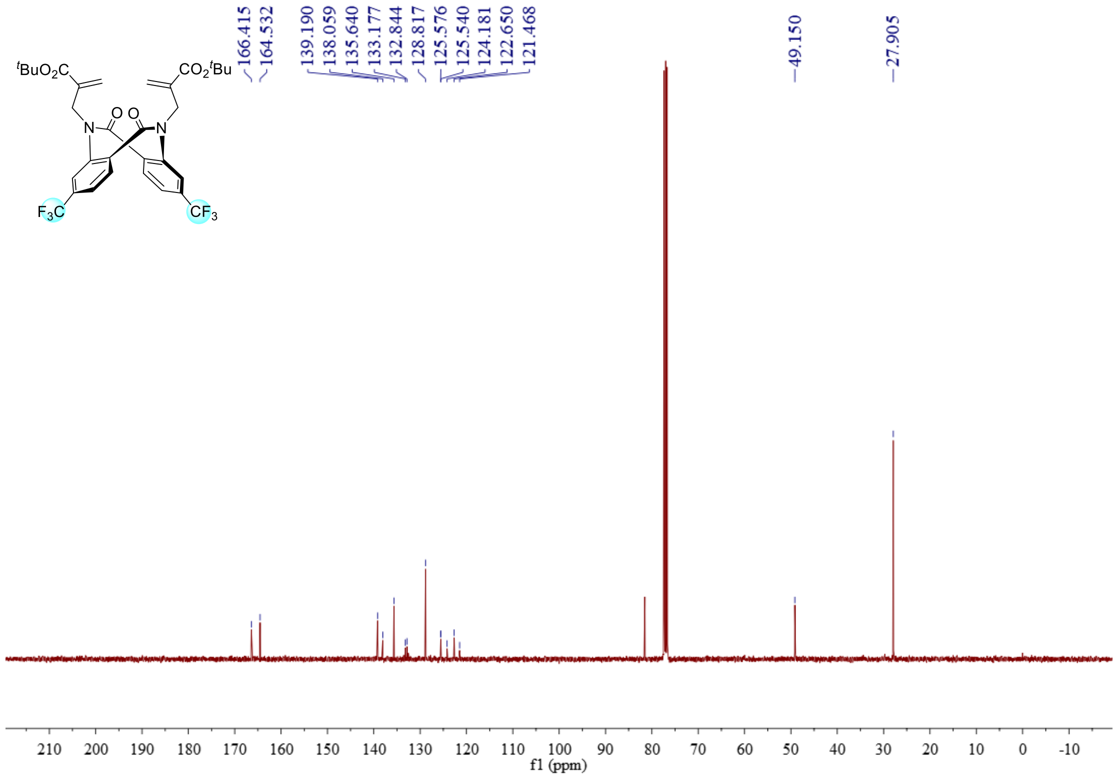

Supplementary Figure 51. ^{13}C NMR spectrum of compound **3n** ($CDCl_3$, 100 MHz)

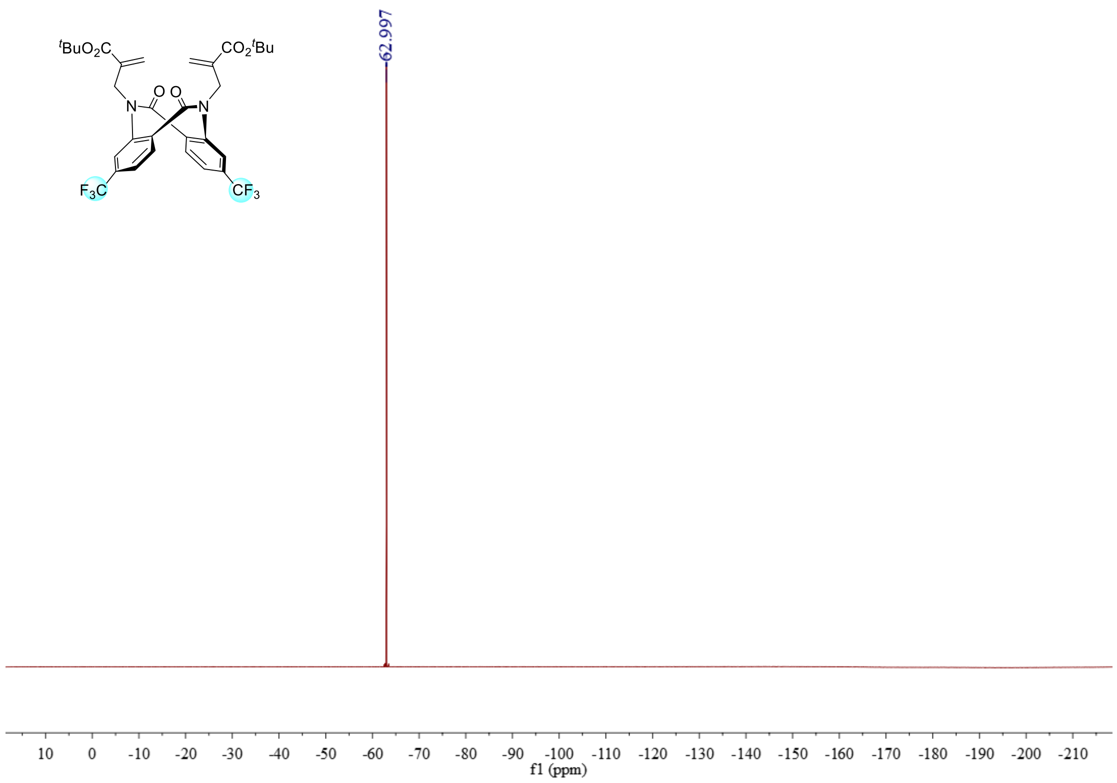

Supplementary Figure 52. ^{19}F NMR spectrum of compound **3n** ($CDCl_3$, 376 MHz)

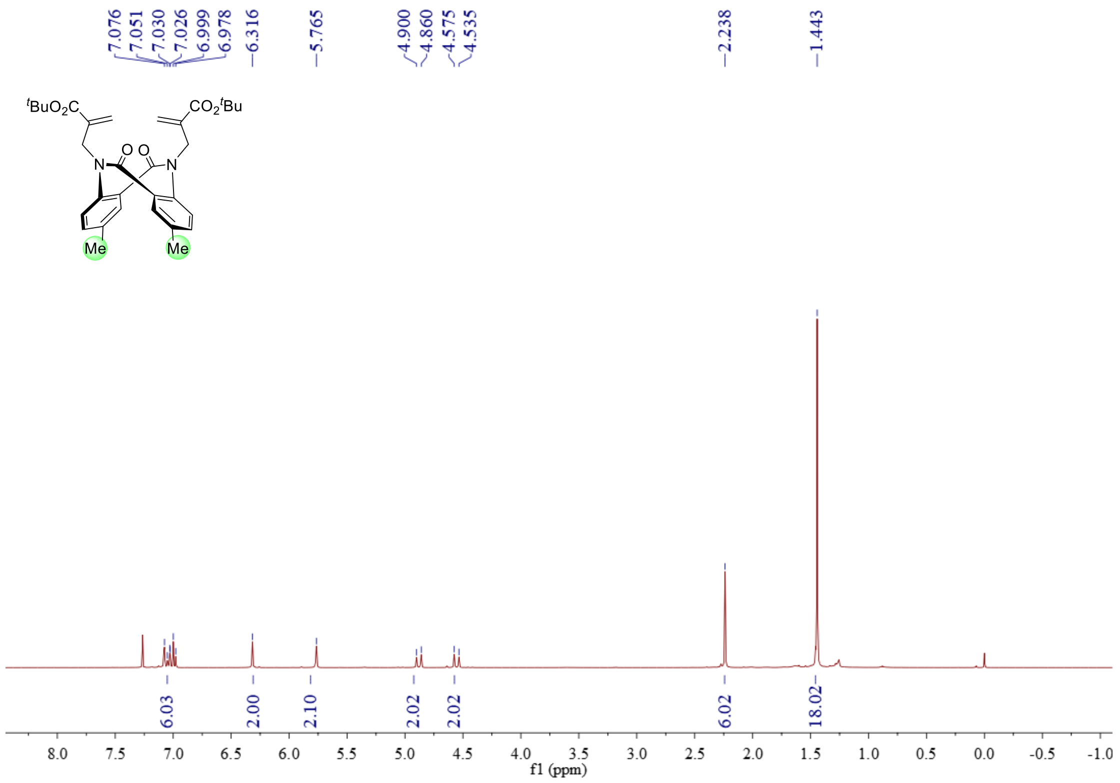

Supplementary Figure 53.^{1}H NMR spectrum of compound **3o** ($CDCl_3$, 400 MHz)

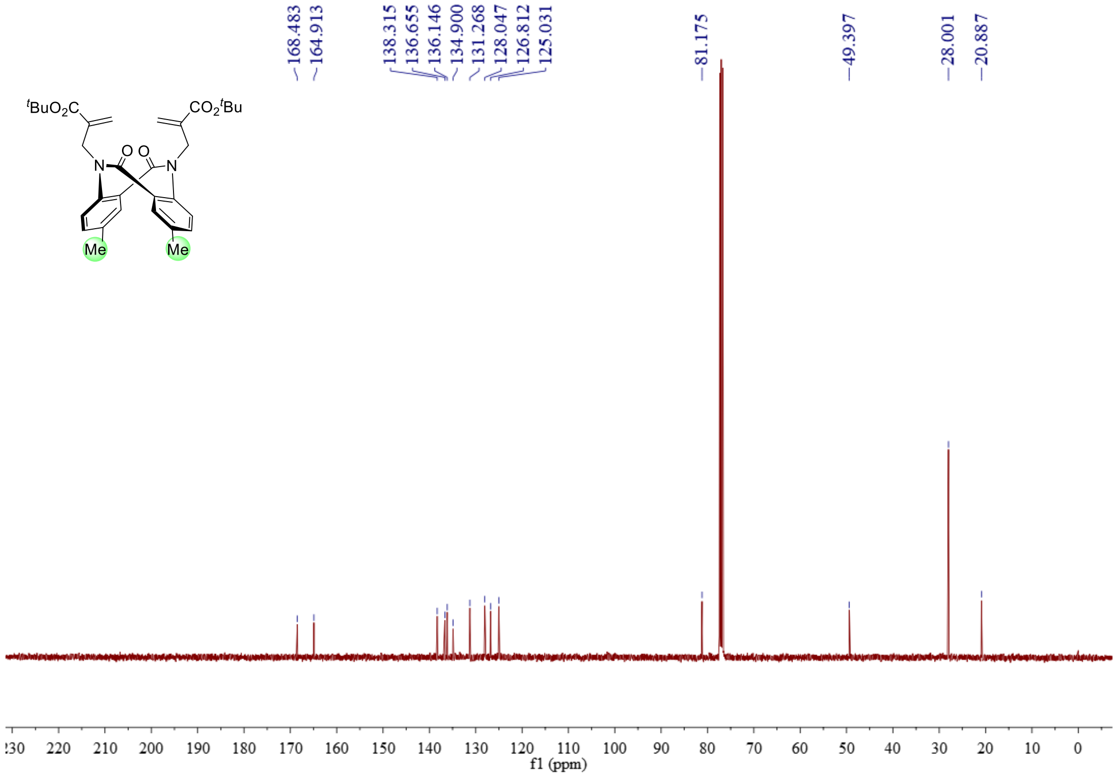

Supplementary Figure 54. ^{13}C NMR spectrum of compound **3o** ($CDCl_3$, 100 MHz)

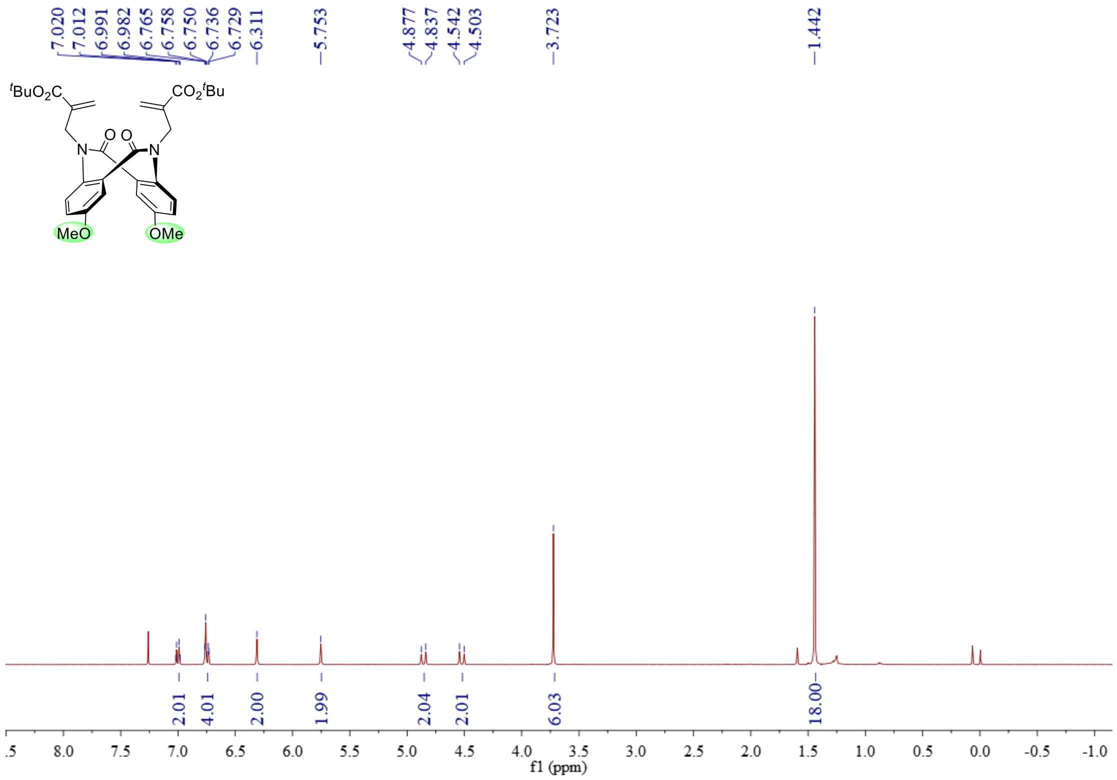

Supplementary Figure 55.^{1}H NMR spectrum of compound **3p** ($CDCl_3$, 400 MHz)

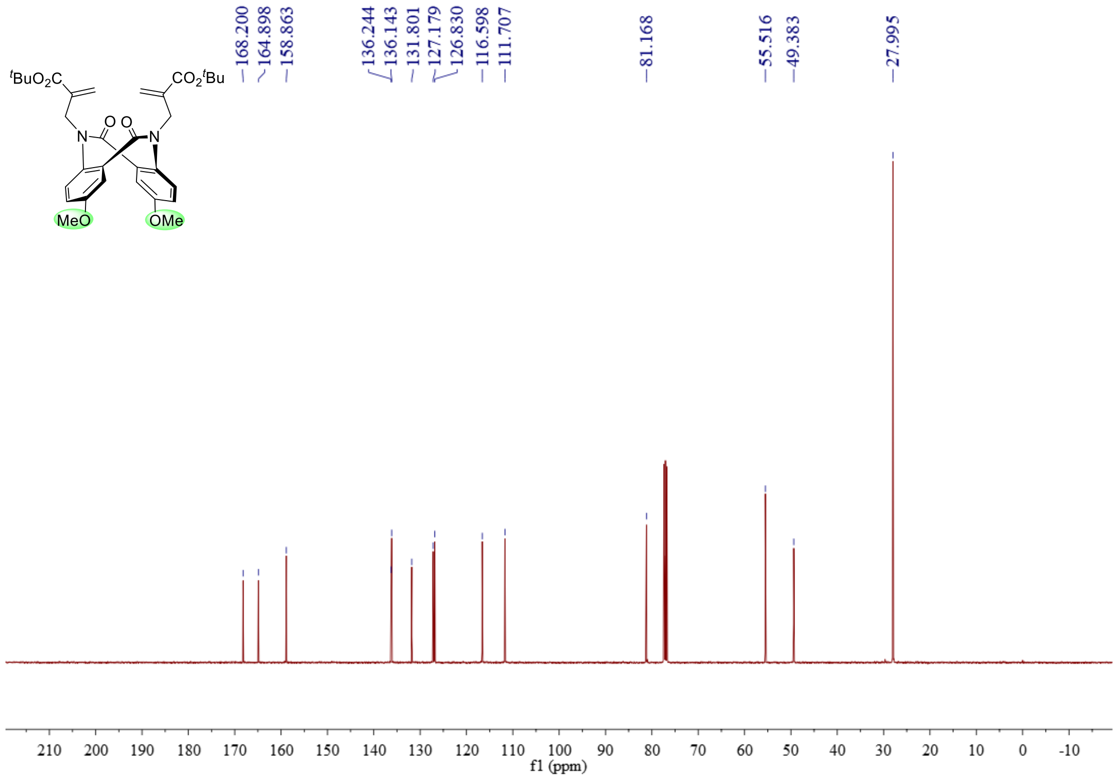

Supplementary Figure 56. ^{13}C NMR spectrum of compound **3p** ($CDCl_3$, 100 MHz)

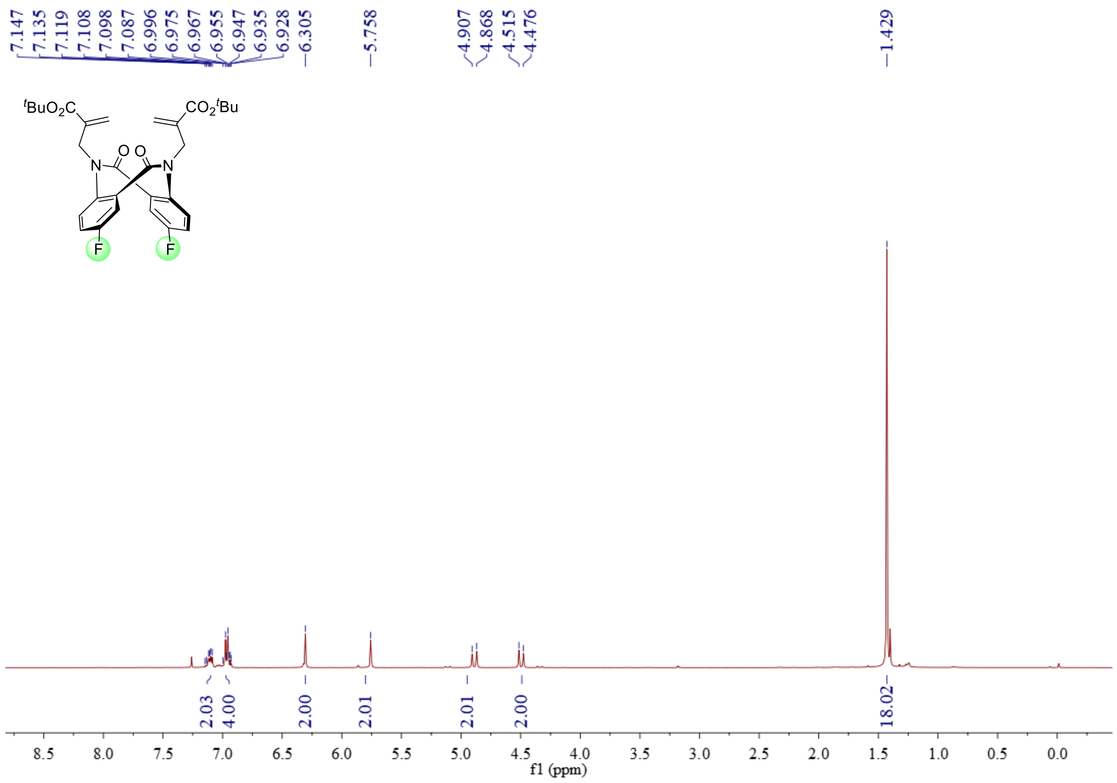

Supplementary Figure 57. ^{1}H NMR spectrum of compound **3q** ($CDCl_3$, 400 MHz)

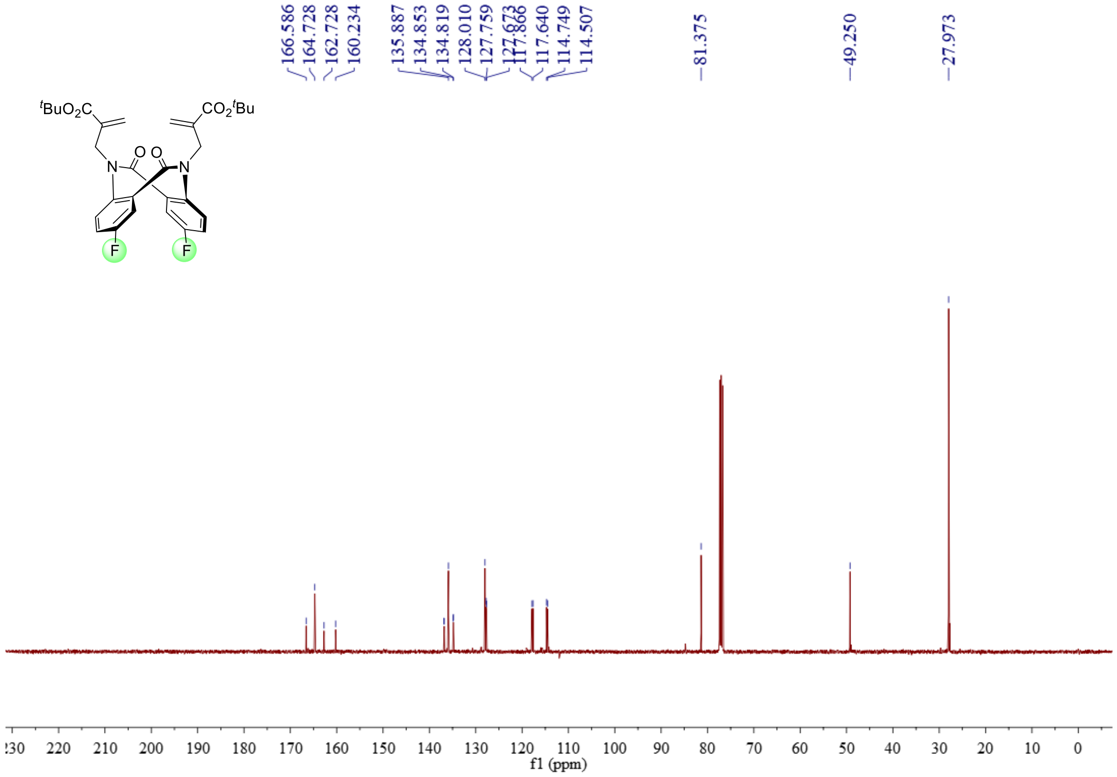

Supplementary Figure 58. ^{13}C NMR spectrum of compound **3q** ($CDCl_3$, 100 MHz)

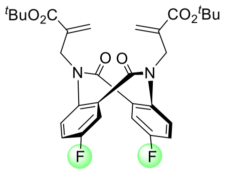

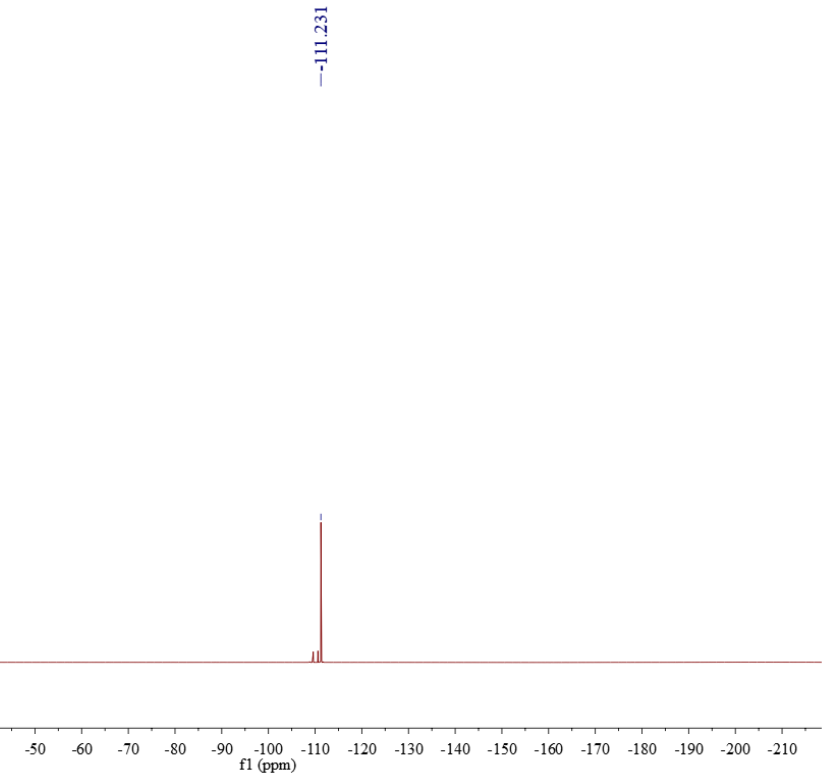

Supplementary Figure 59. ^{19}F NMR spectrum of compound **3q** ($CDCl_3$, 376 MHz)

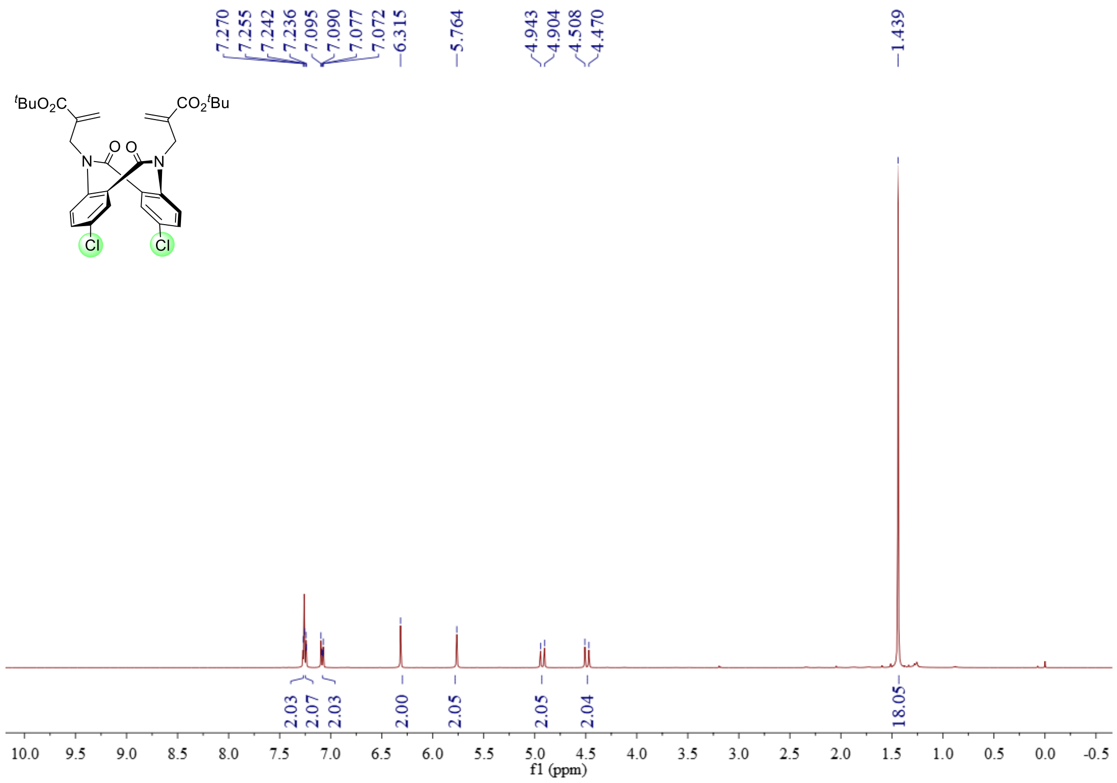

Supplementary Figure 60. ^{1}H NMR spectrum of compound **3r** ($CDCl_3$, 400 MHz)

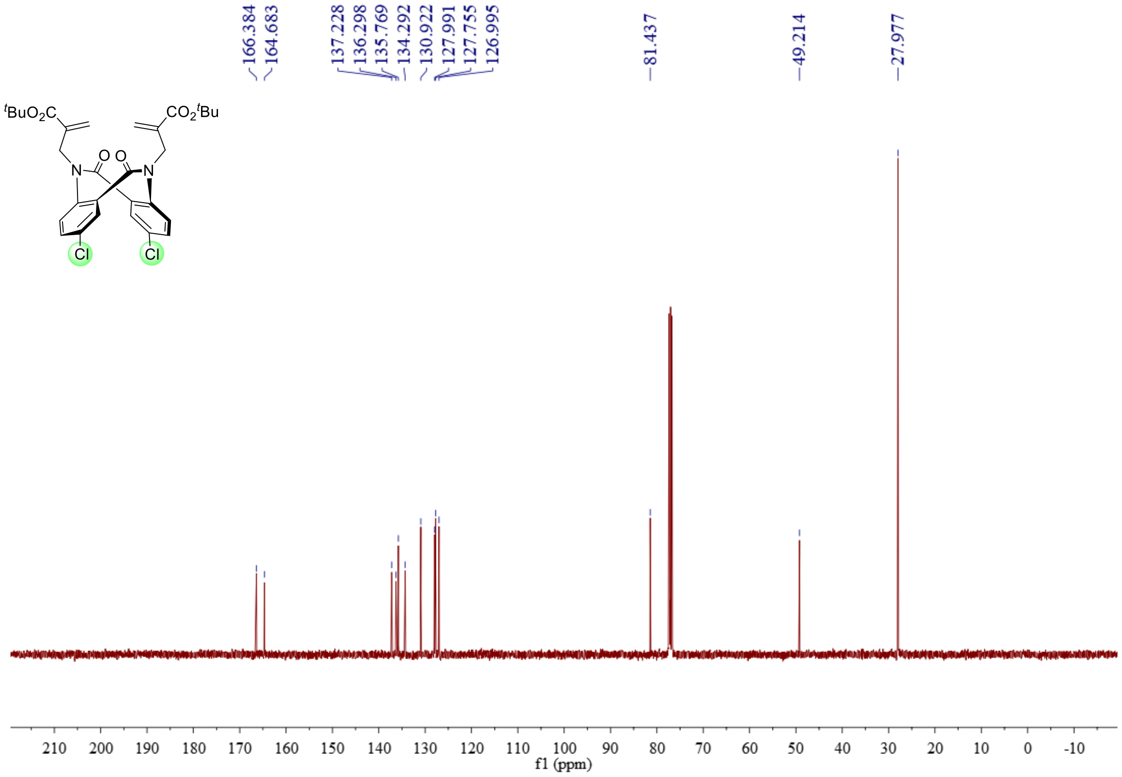

Supplementary Figure 61. ^{13}C NMR spectrum of compound **3r** ($CDCl_3$, 100 MHz)

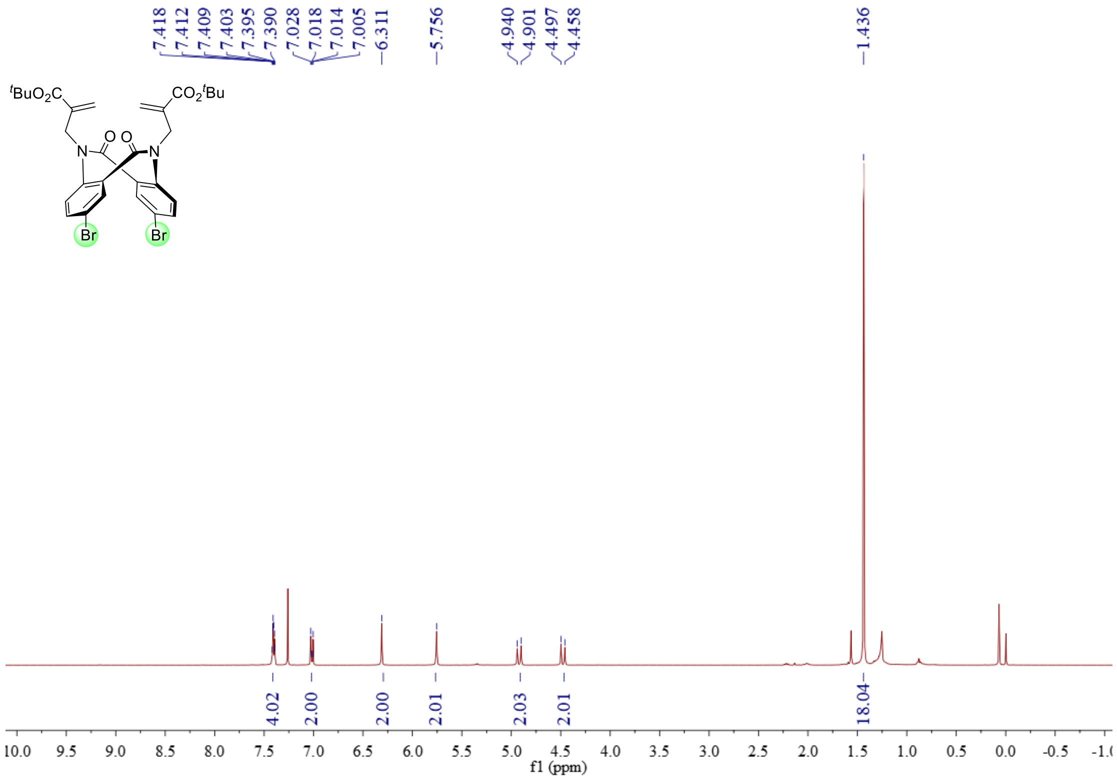

Supplementary Figure 62. ^1H NMR spectrum of compound **3s** ($CDCl_3$, 400 MHz)

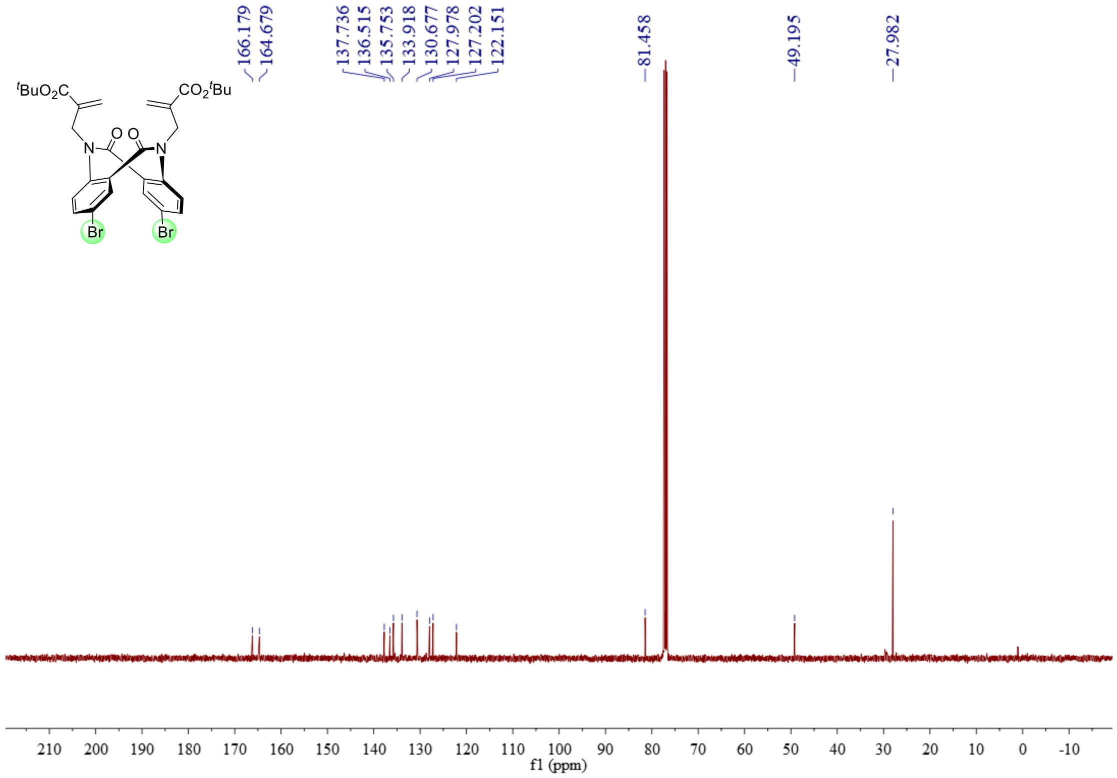

Supplementary Figure 63. ^{13}C NMR spectrum of compound **3s** ($CDCl_3$, 100 MHz)

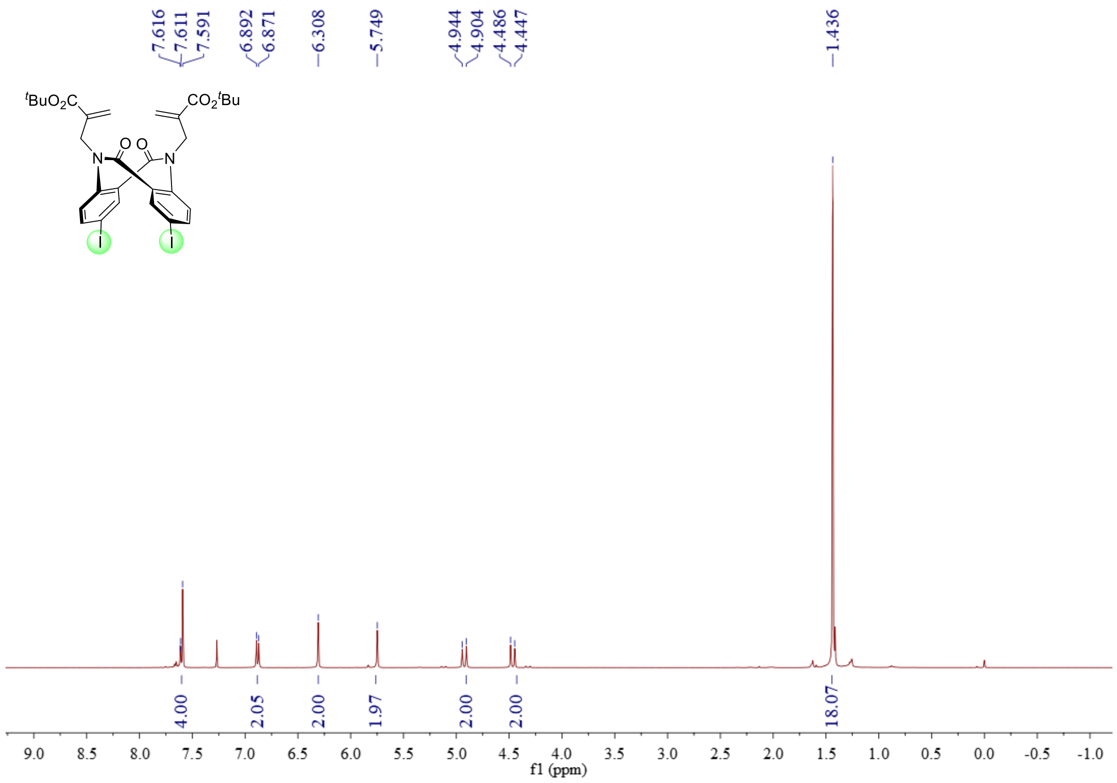

Supplementary Figure 64. ^{1}H NMR spectrum of compound **3t** ($CDCl_3$, 400 MHz)

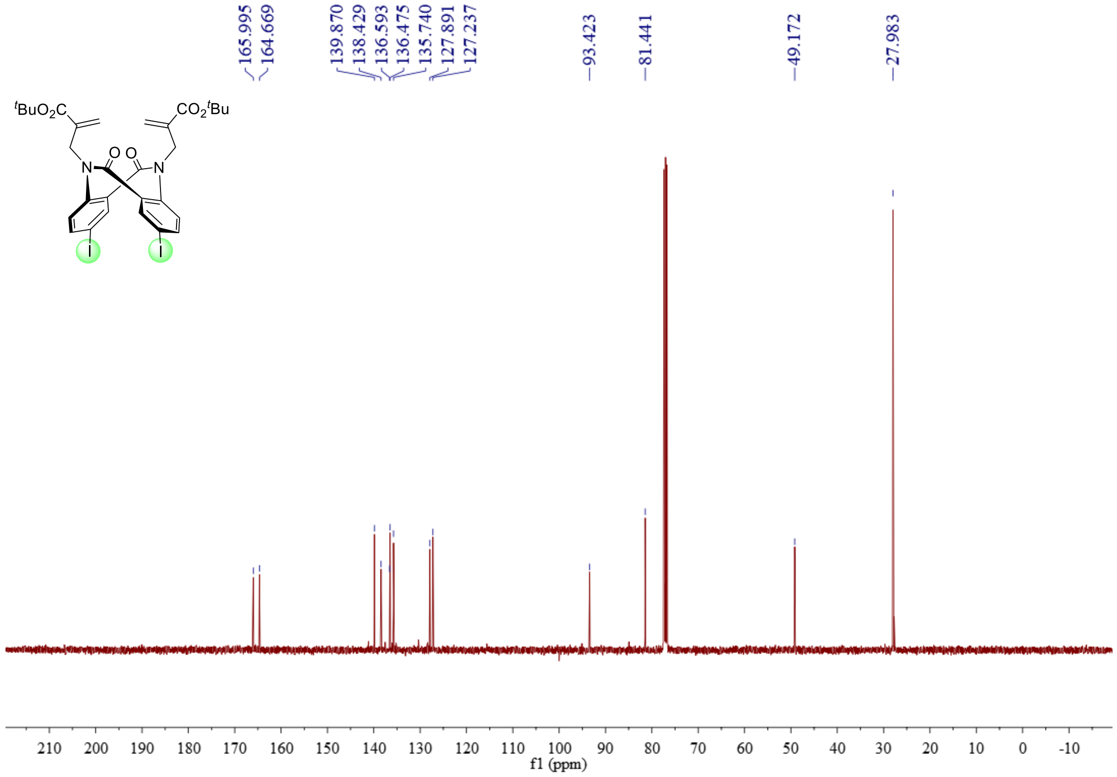

Supplementary Figure 65. ^{13}C NMR spectrum of compound **3t** ($CDCl_3$, 100 MHz)

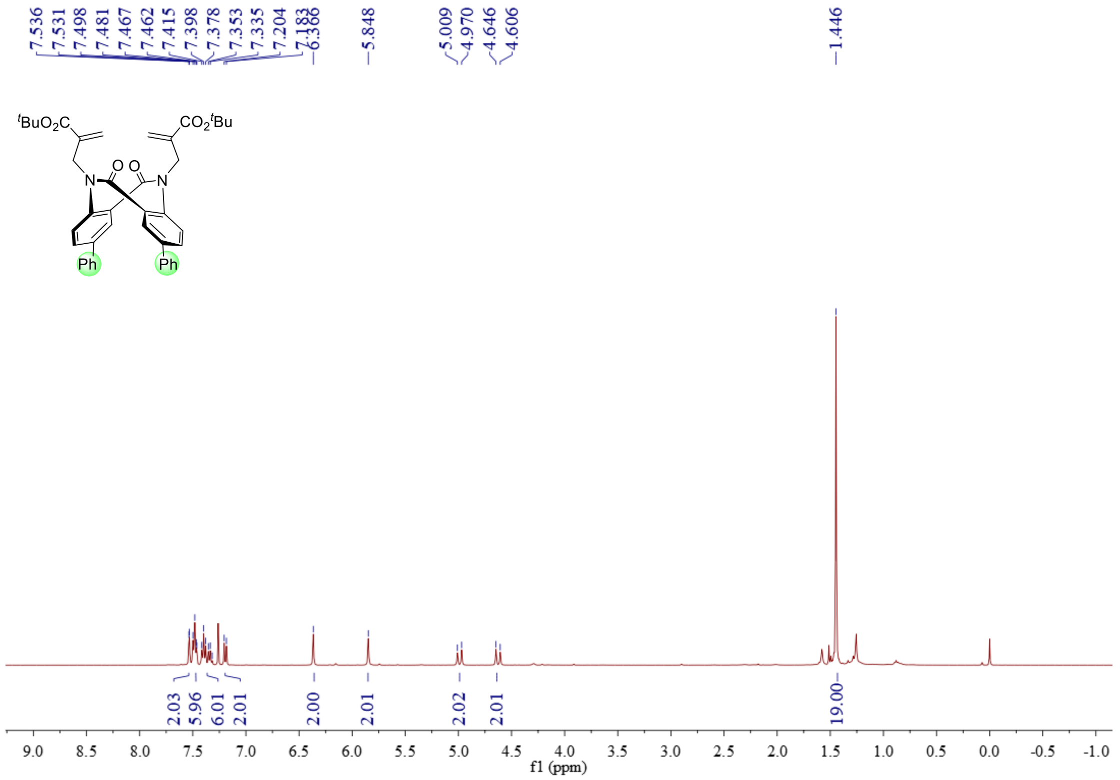

Supplementary Figure 66. ^{1}H NMR spectrum of compound **3u** ($CDCl_3$, 400 MHz)

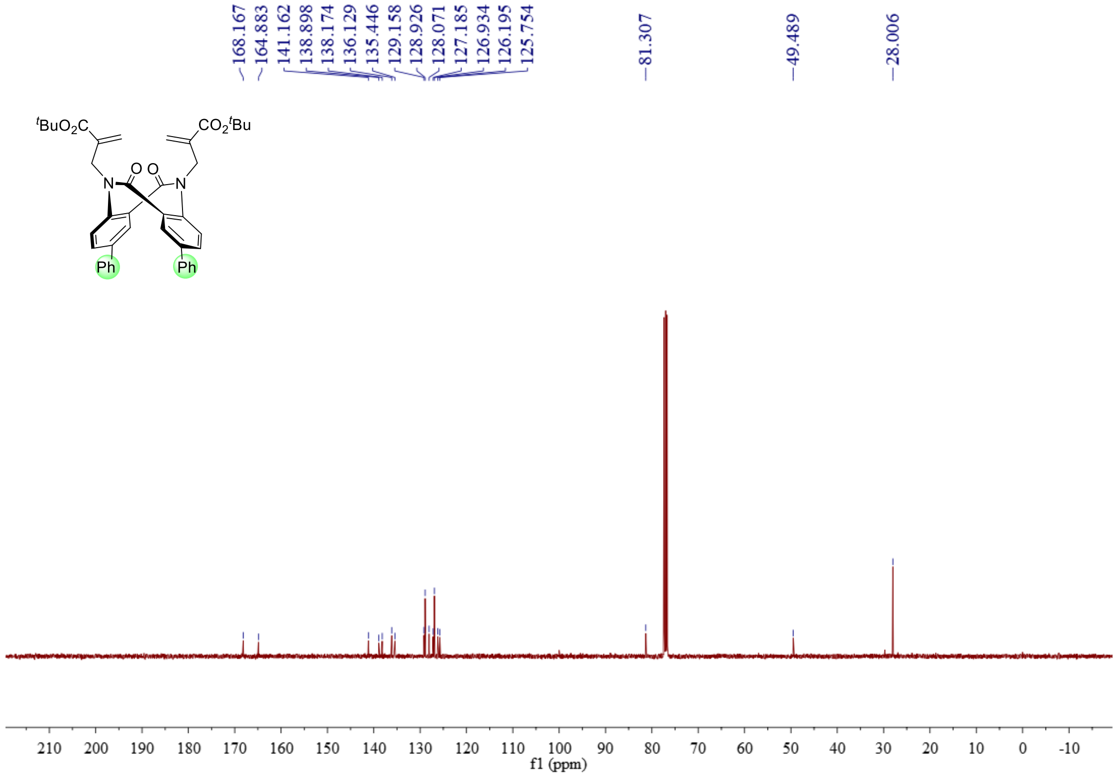

Supplementary Figure 67. ^{13}C NMR spectrum of compound **3u** ($CDCl_3$, 100 MHz)

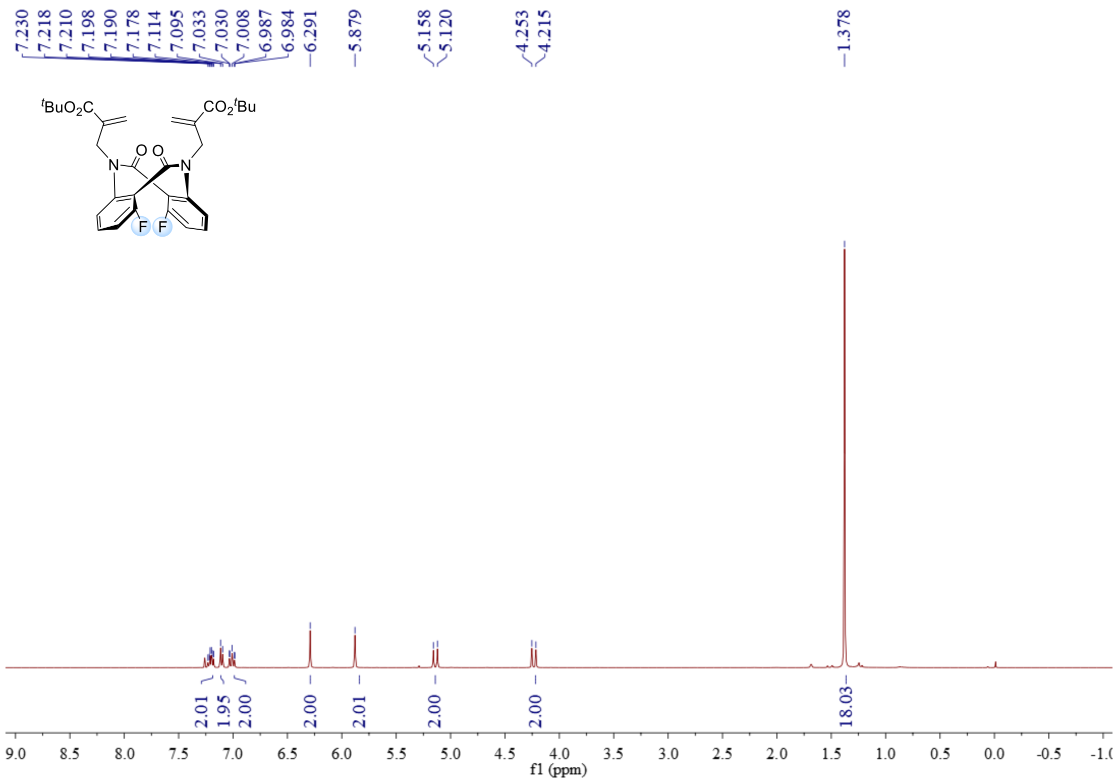

Supplementary Figure 68. ^1H NMR spectrum of compound **3v** ($CDCl_3$, 400 MHz)

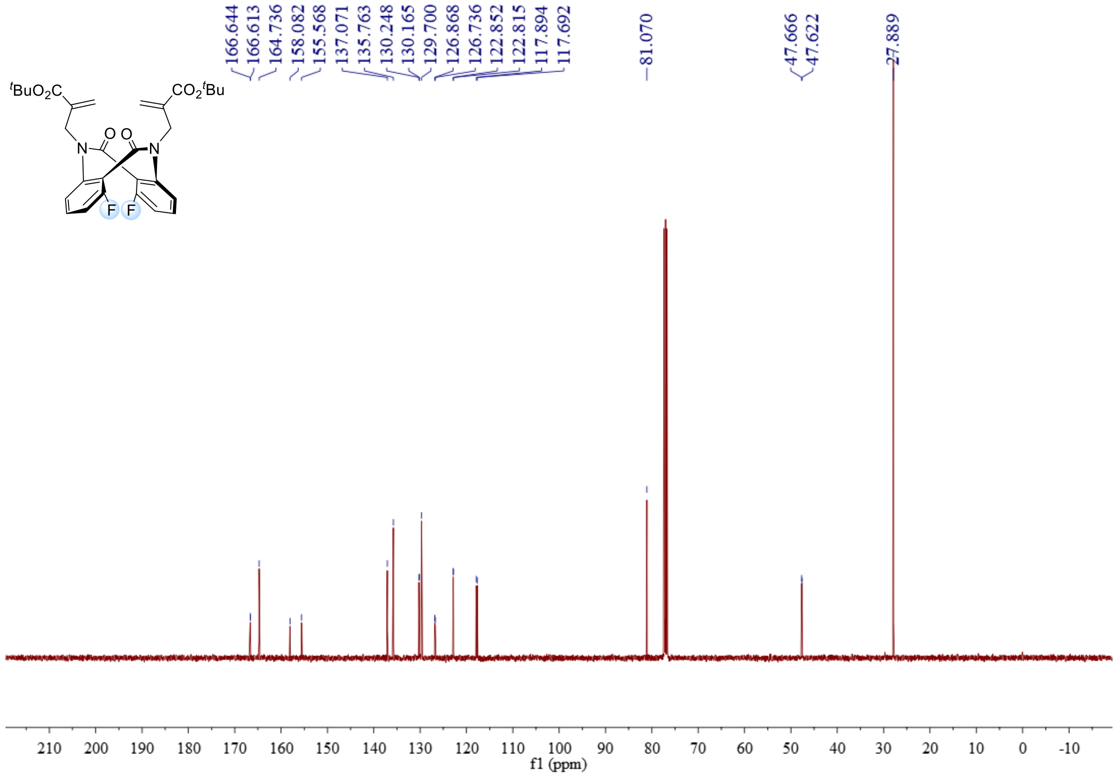

Supplementary Figure 69. ^{13}C NMR spectrum of compound **3v** ($CDCl_3$, 100 MHz)

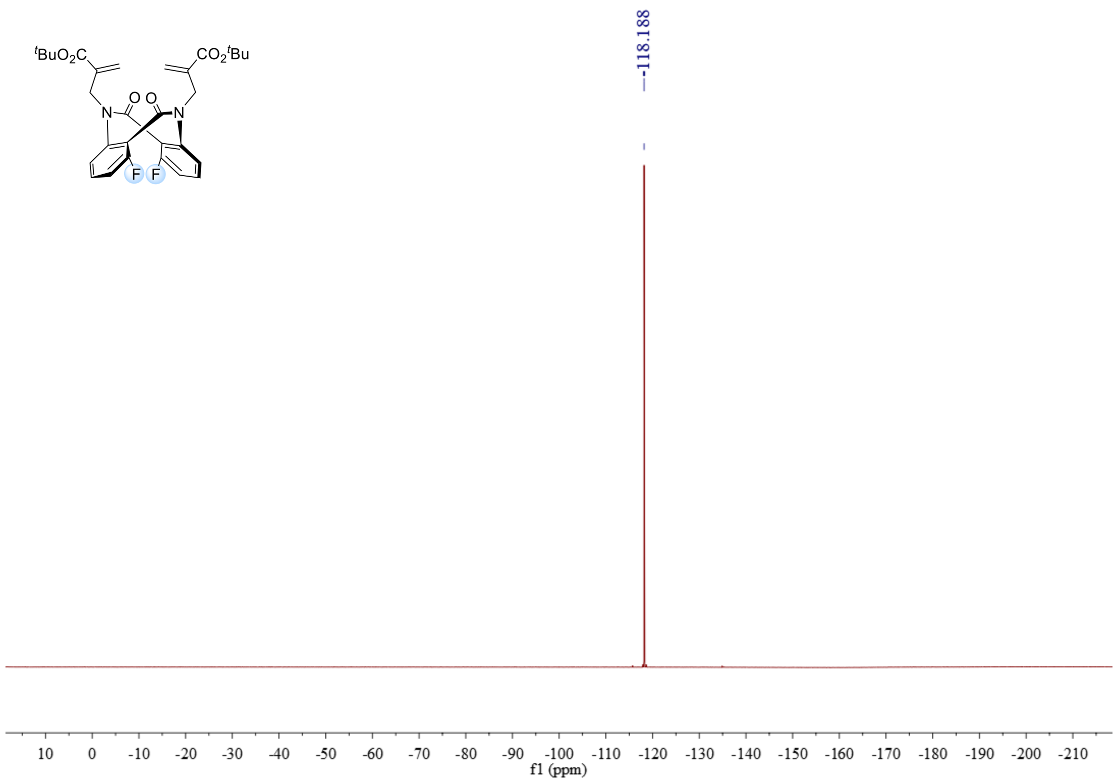

Supplementary Figure 70. ^{19}F NMR spectrum of compound **3v** ($CDCl_3$, 376 MHz)

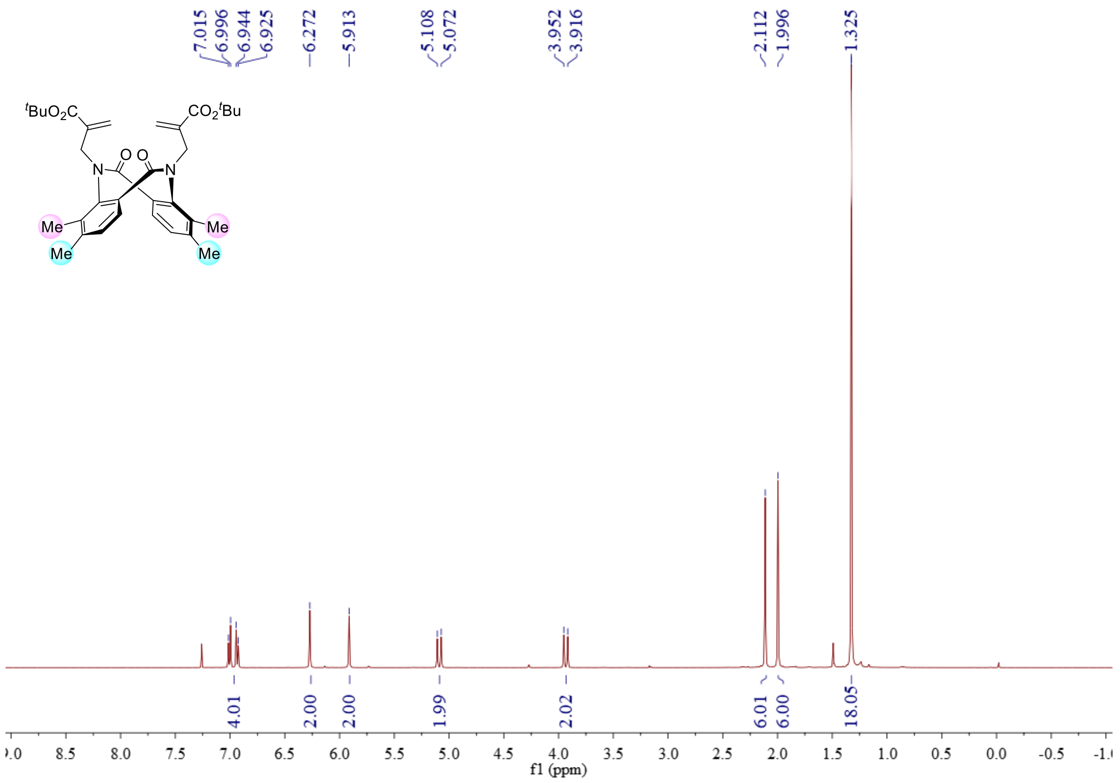

Supplementary Figure 71. ^{1}H NMR spectrum of compound **3w** ($CDCl_3$, 400 MHz)

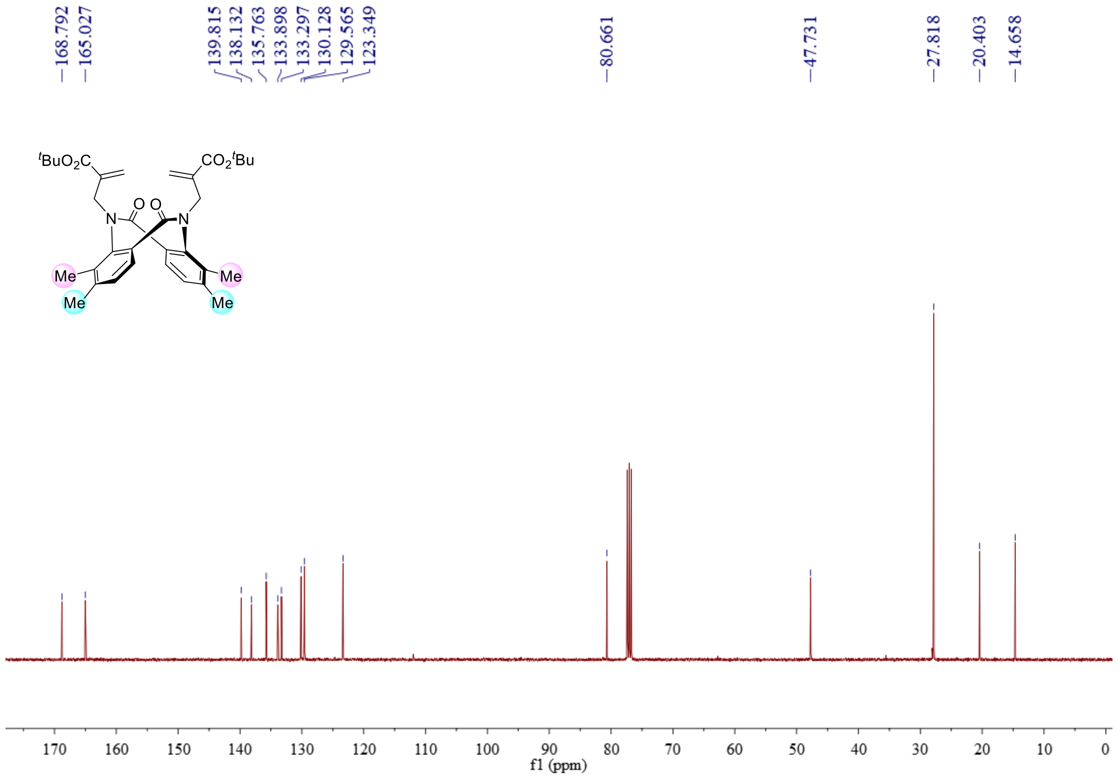

Supplementary Figure 72. ^{13}C NMR spectrum of compound **3w** ($CDCl_3$, 100 MHz)

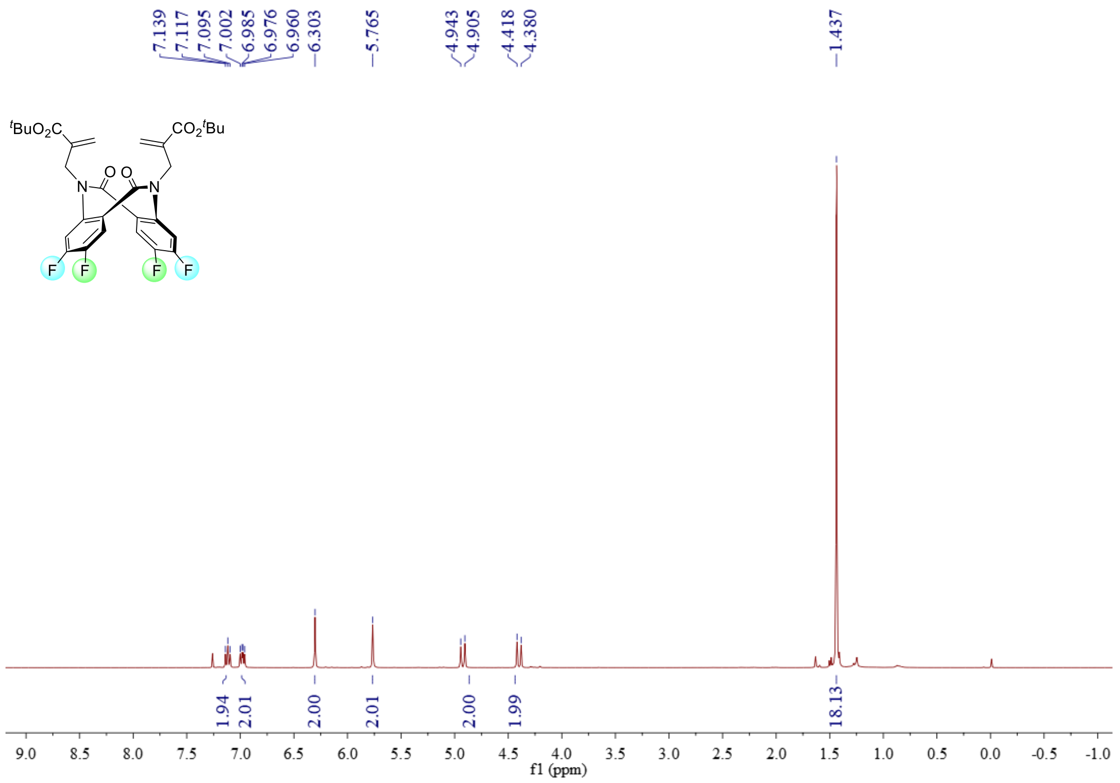

Supplementary Figure 73. ^{1}H NMR spectrum of compound **3x** ($CDCl_3$, 400 MHz)

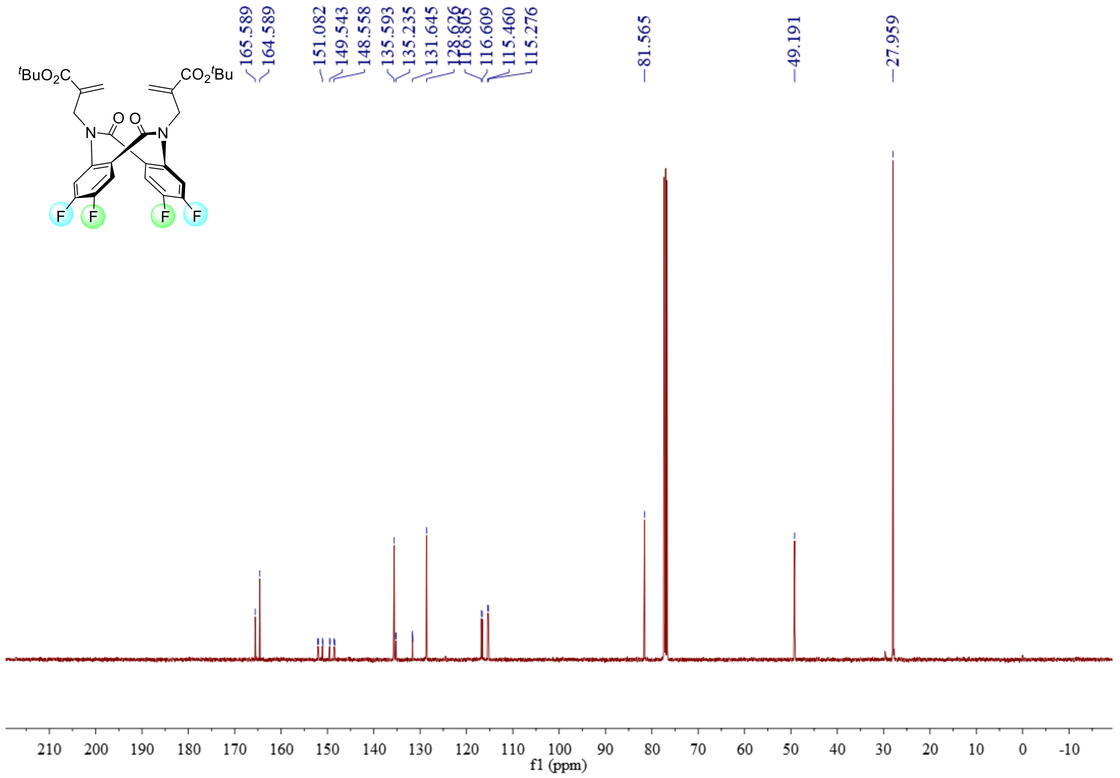

Supplementary Figure 74. ^{13}C NMR spectrum of compound **3x** ($CDCl_3$, 100 MHz)

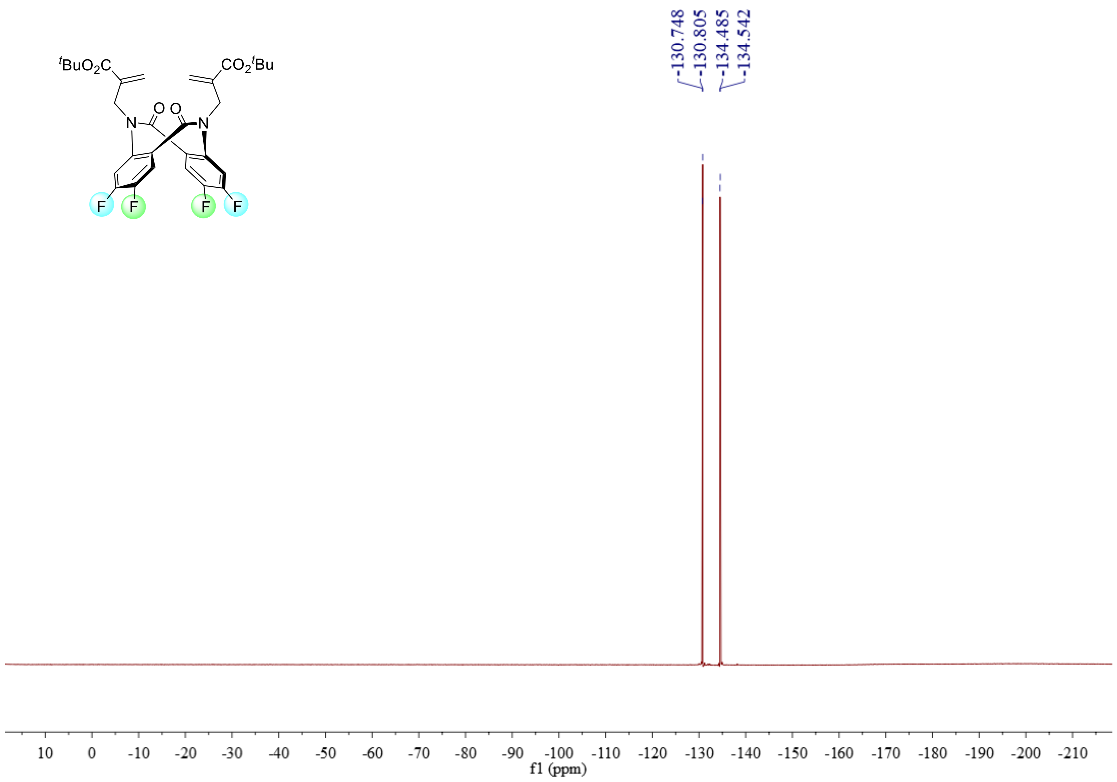

Supplementary Figure 75. ^{19}F NMR spectrum of compound **3x** ($CDCl_3$, 376 MHz)

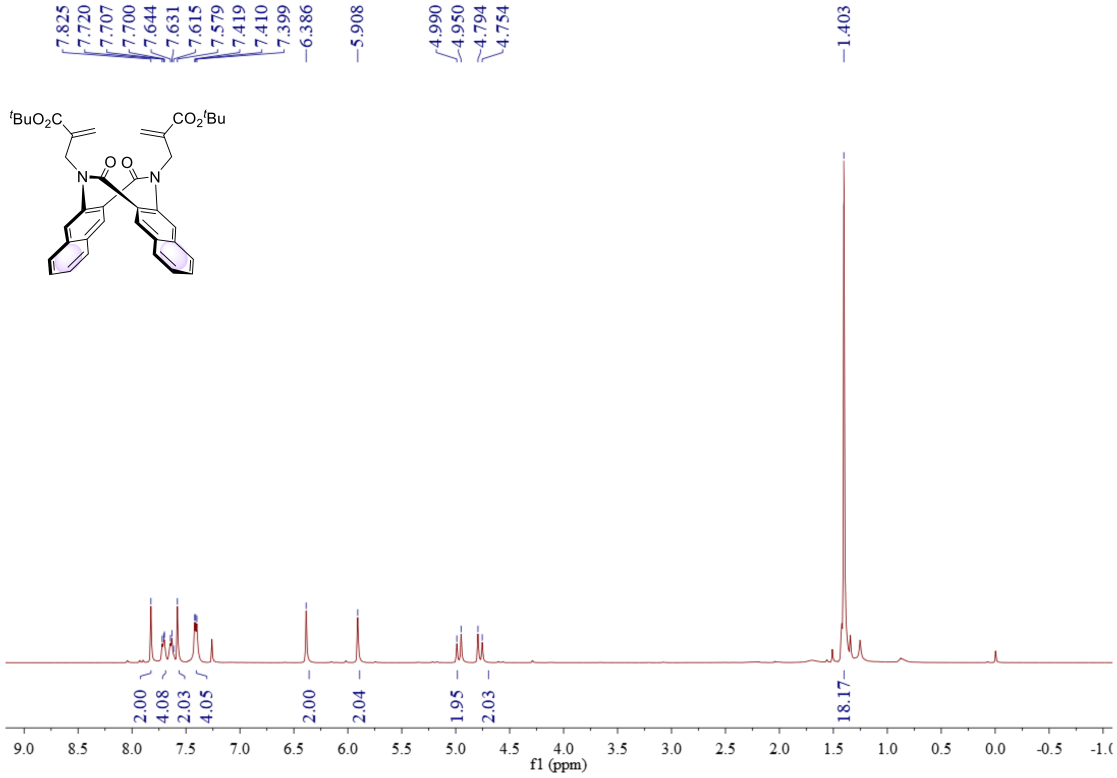

Supplementary Figure 76. ^{1}H NMR spectrum of compound **3y** ($CDCl_3$, 400 MHz)

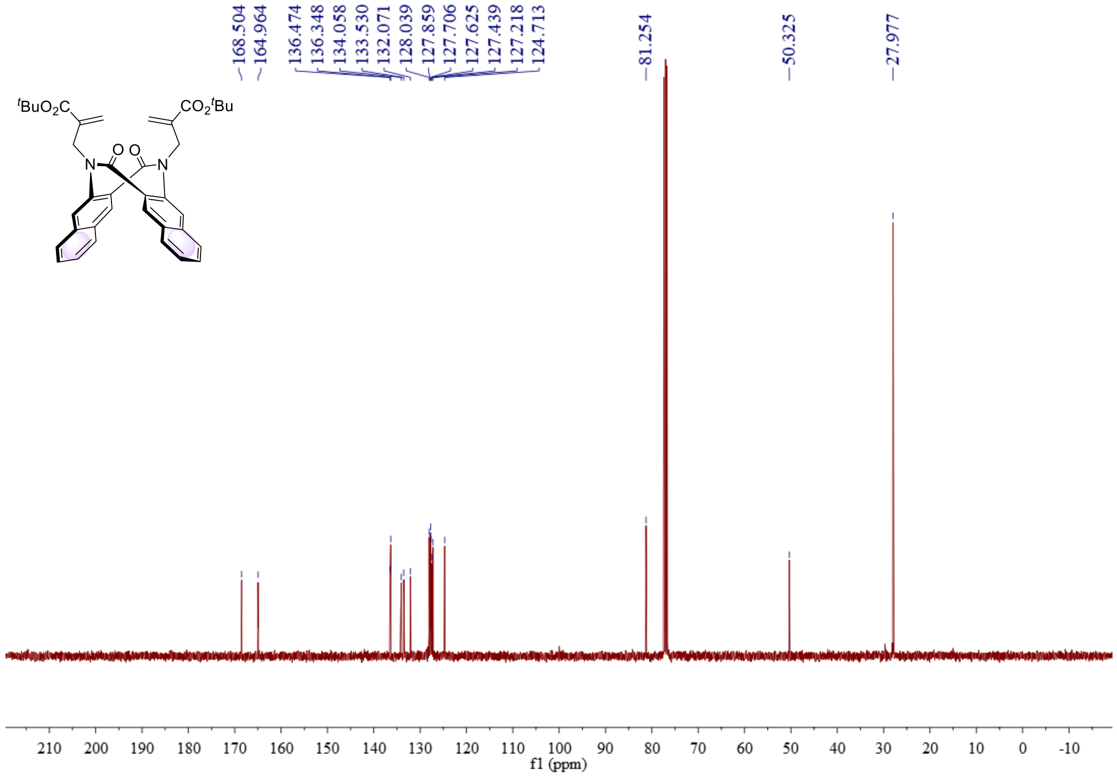

Supplementary Figure 77. ^{13}C NMR spectrum of compound **3y** ($CDCl_3$, 100 MHz)

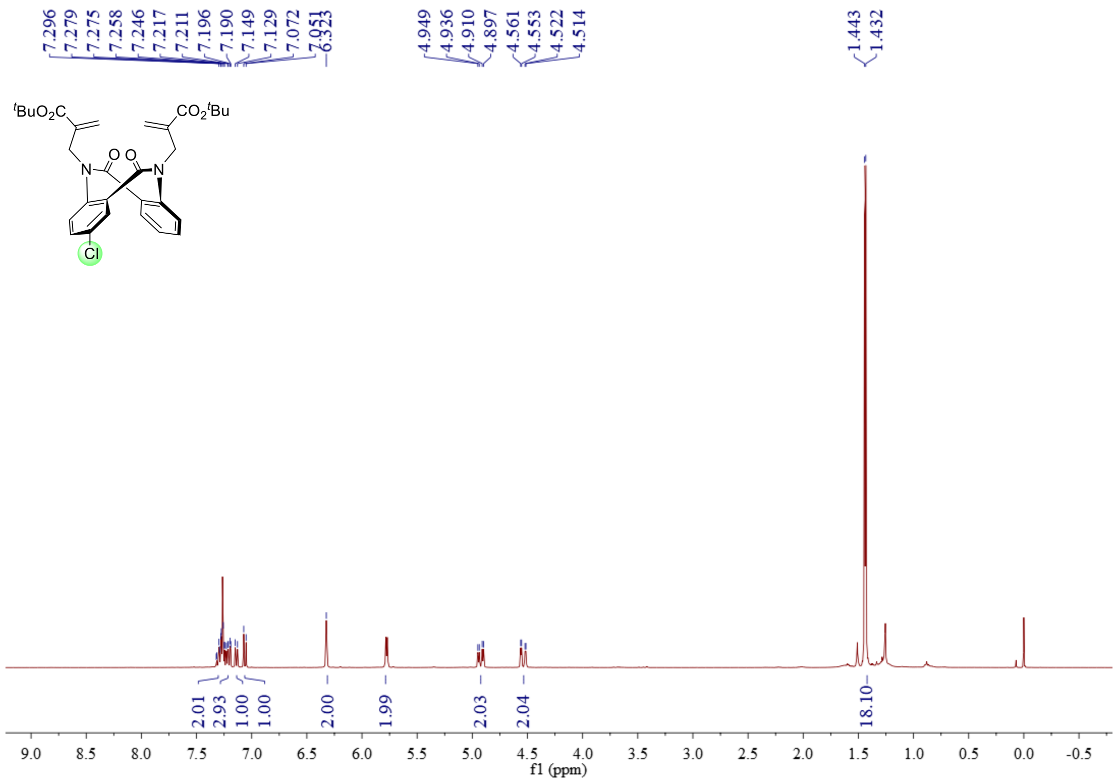

Supplementary Figure 78. ^{1}H NMR spectrum of compound **3z** ($CDCl_3$, 400 MHz)

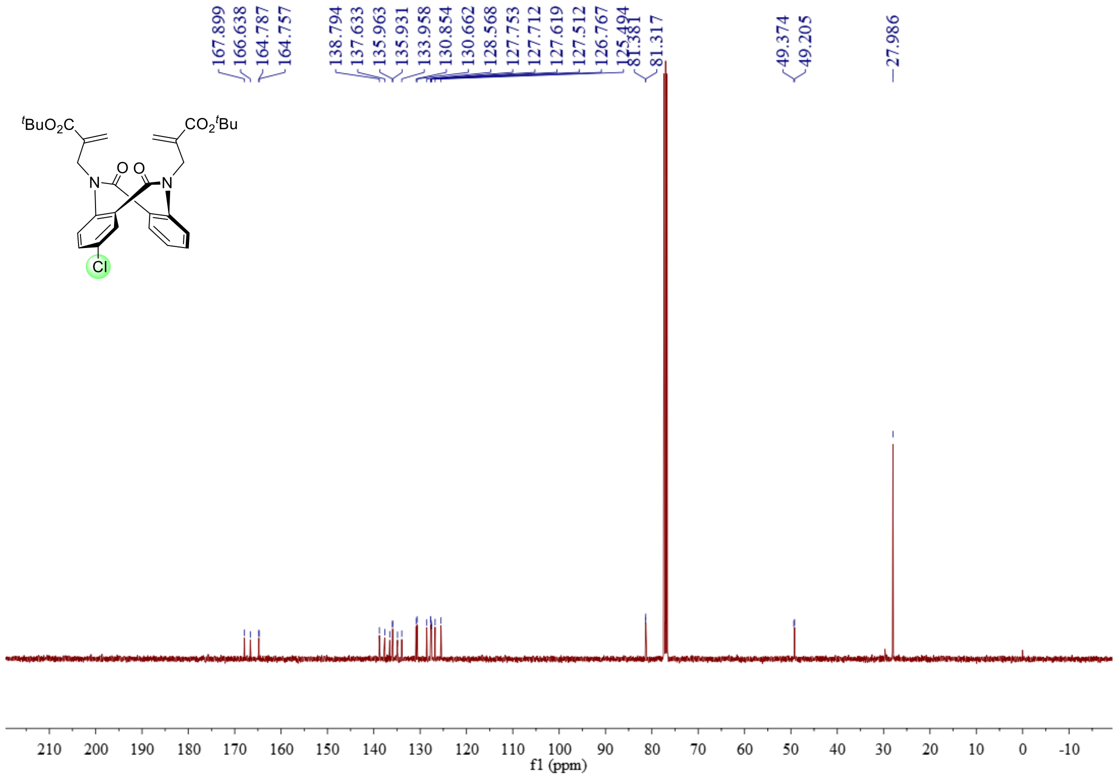

Supplementary Figure 79. ^{13}C NMR spectrum of compound **3z** ($CDCl_3$, 100 MHz)

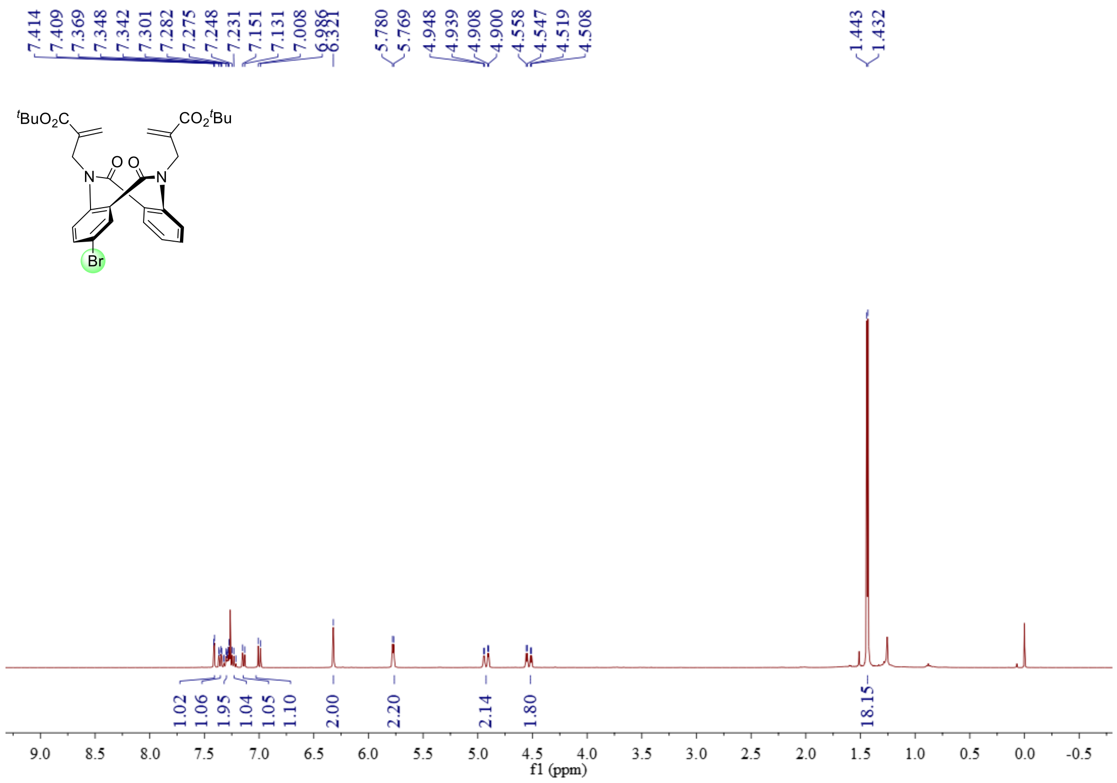

Supplementary Figure 80. ^{1}H NMR spectrum of compound **3aa** ($CDCl_3$, 400 MHz)

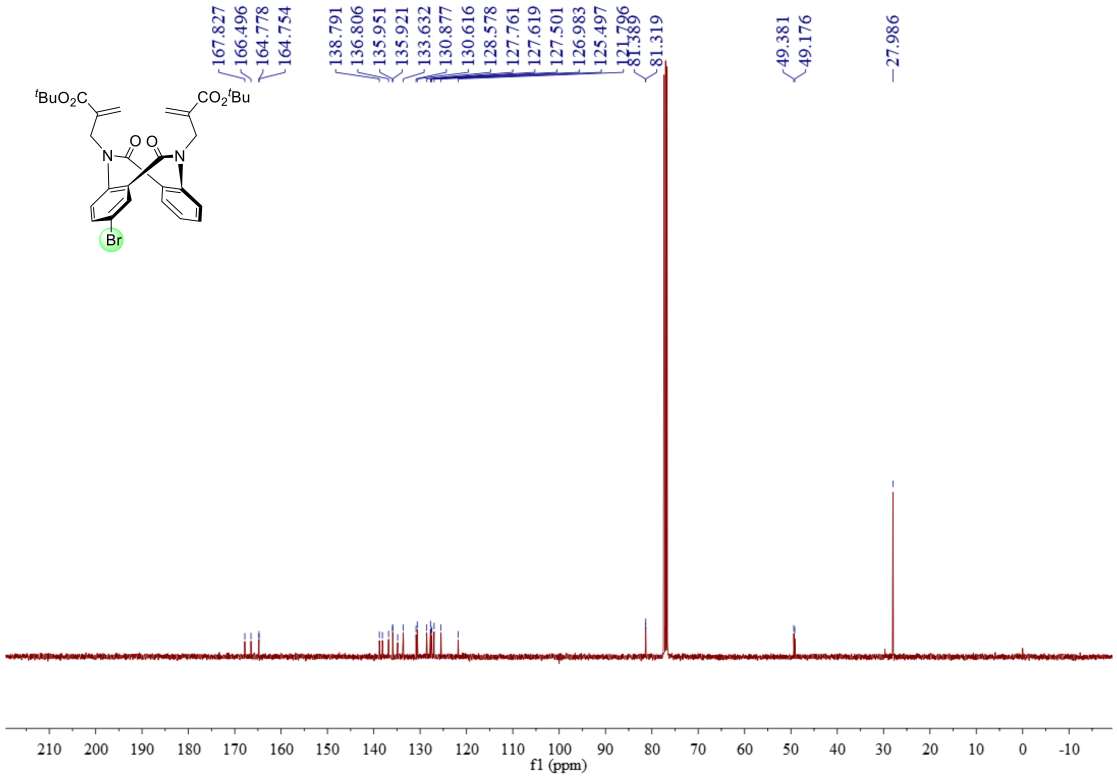

Supplementary Figure 81. ^{13}C NMR spectrum of compound **3aa** ($CDCl_3$, 100 MHz)

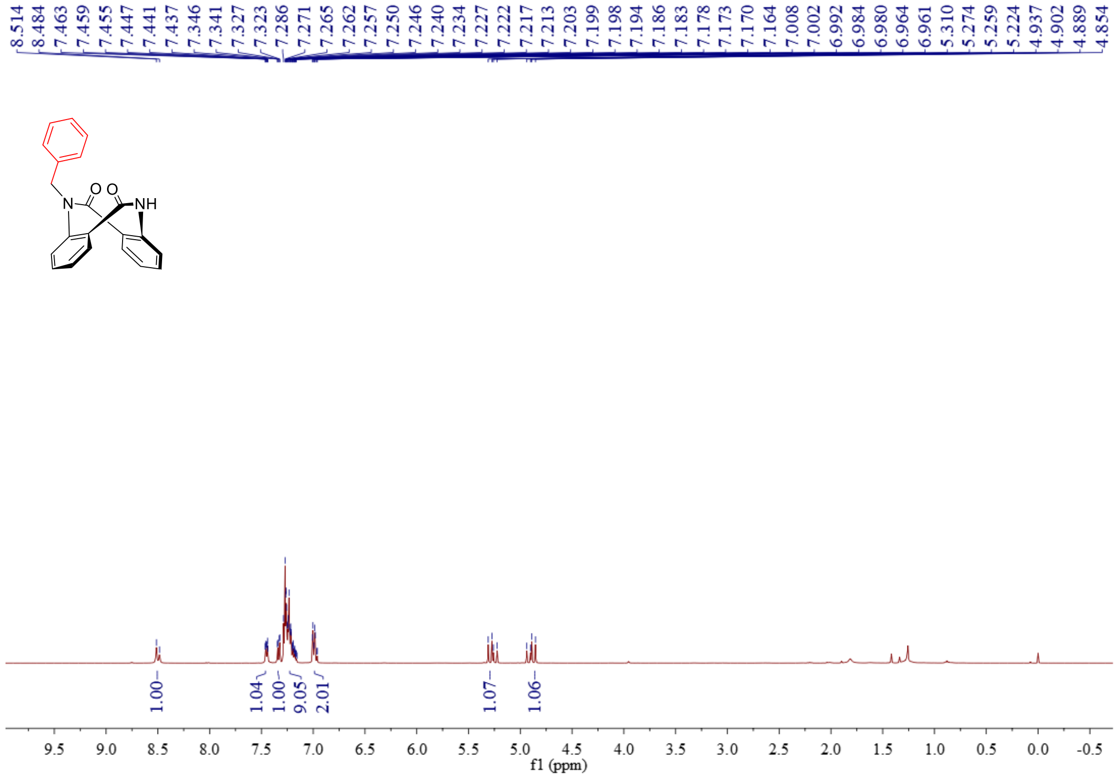

Supplementary Figure 82. ^{1}H NMR spectrum of compound **4a** ($CDCl_3$, 400 MHz)

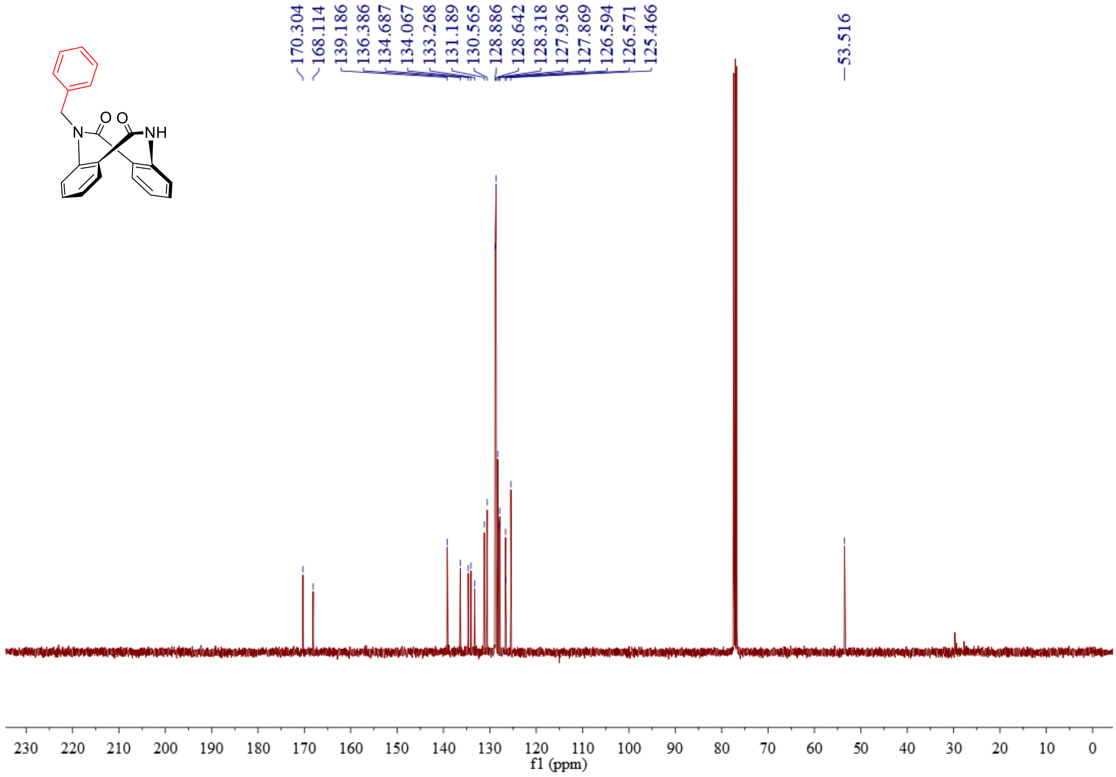

Supplementary Figure 83. ^{13}C NMR spectrum of compound **4a** ($CDCl_3$, 100 MHz)

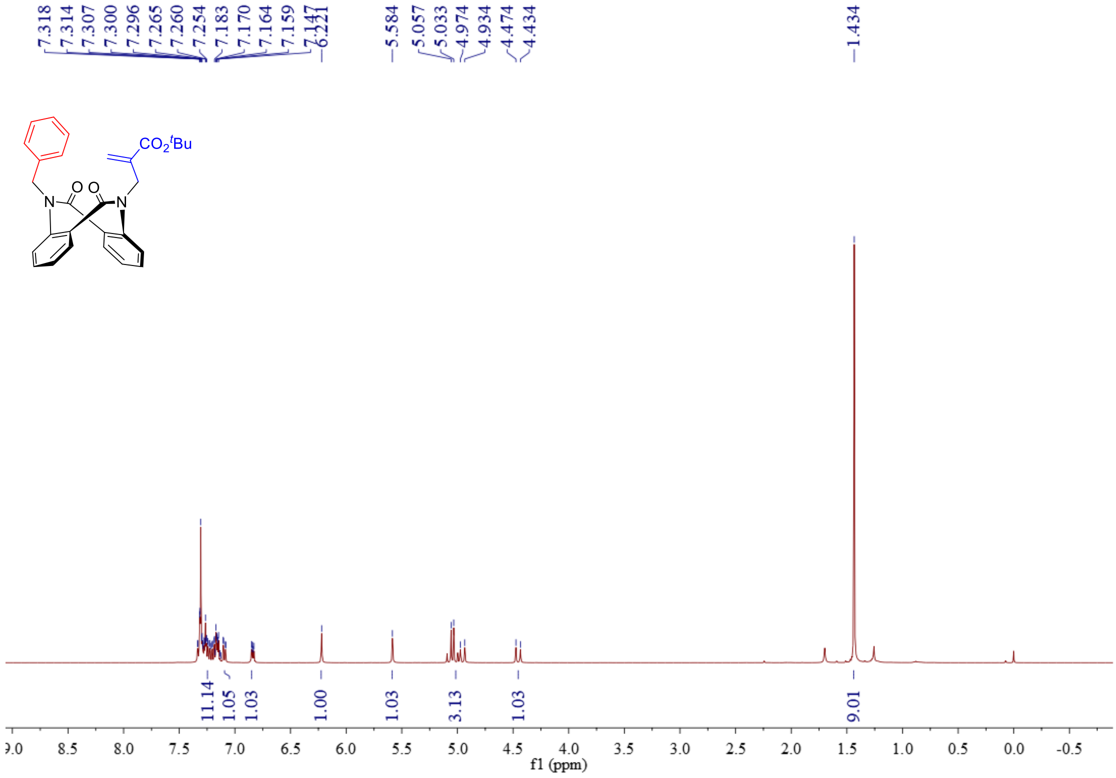

Supplementary Figure 84. ^{1}H NMR spectrum of compound **5a** ($CDCl_3$, 400 MHz)

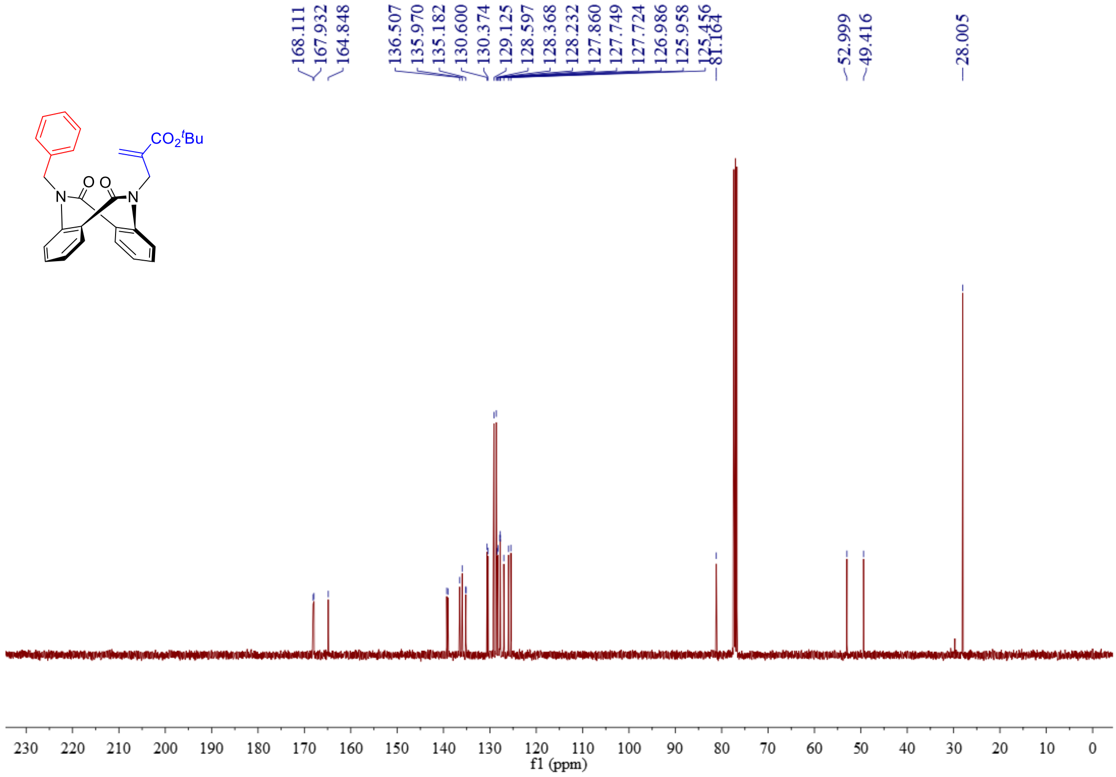

Supplementary Figure 85. ^{13}C NMR spectrum of compound **5a** ($CDCl_3$, 100 MHz)

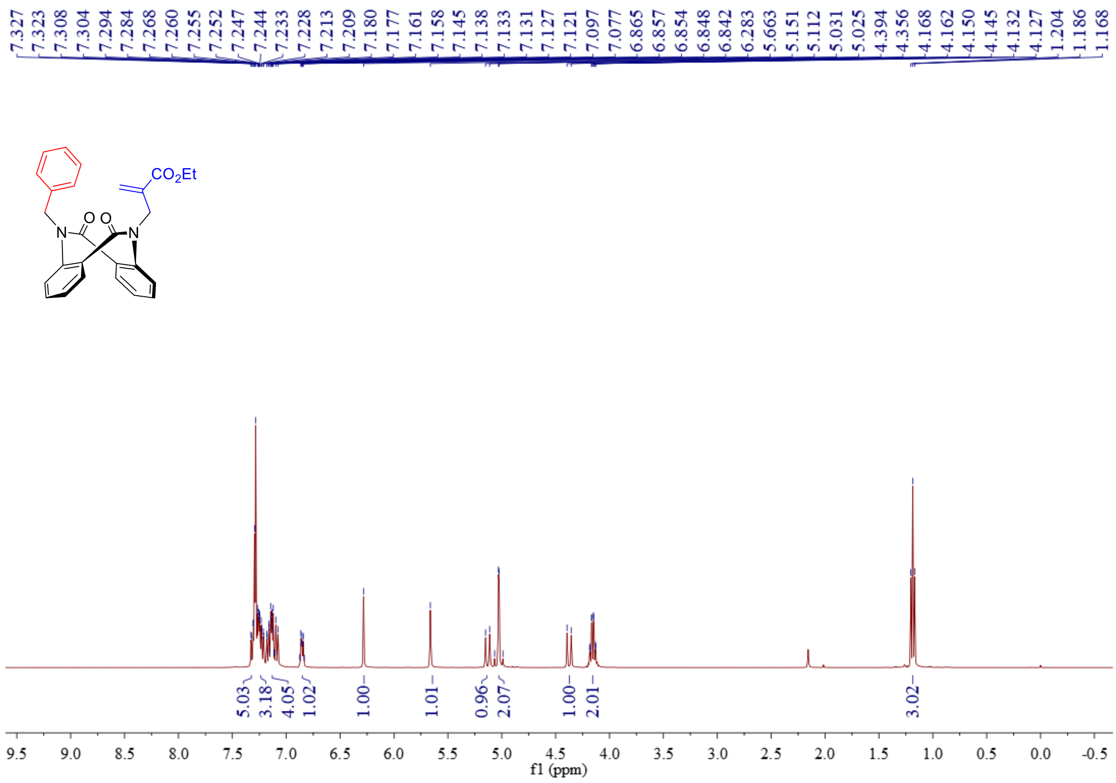

Supplementary Figure 86. ^{1}H NMR spectrum of compound **5b** ($CDCl_3$, 400 MHz)

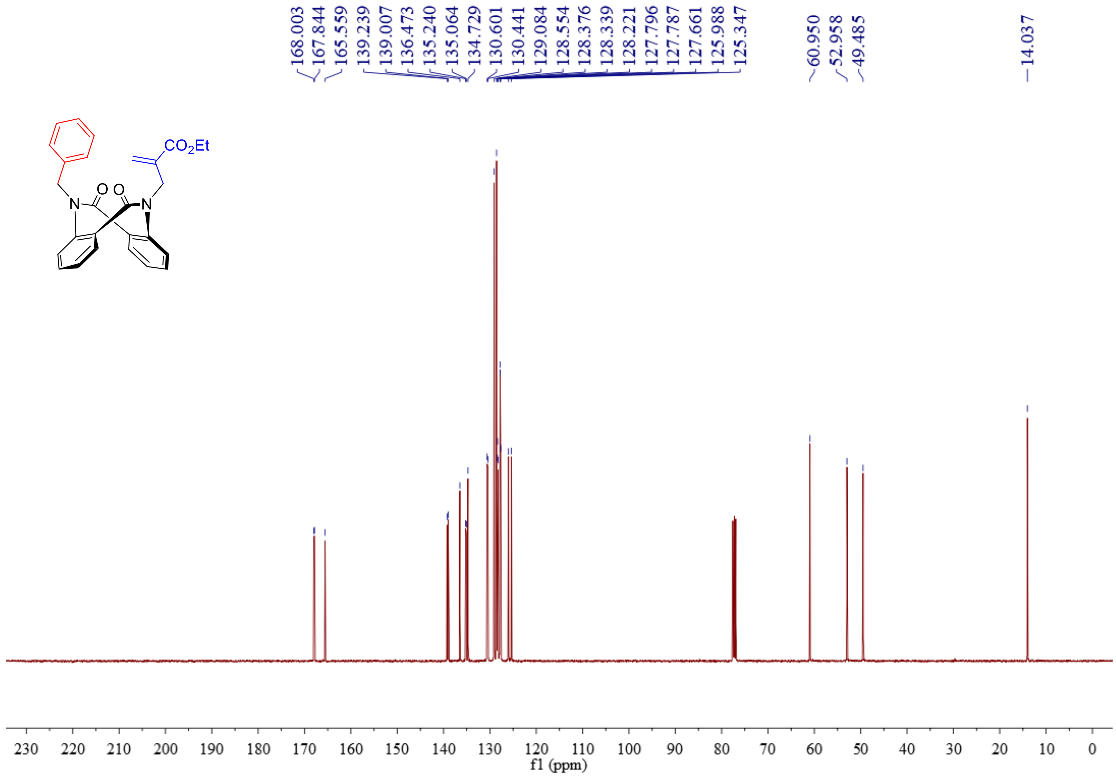

Supplementary Figure 87. ^{13}C NMR spectrum of compound **5b** ($CDCl_3$, 100 MHz)

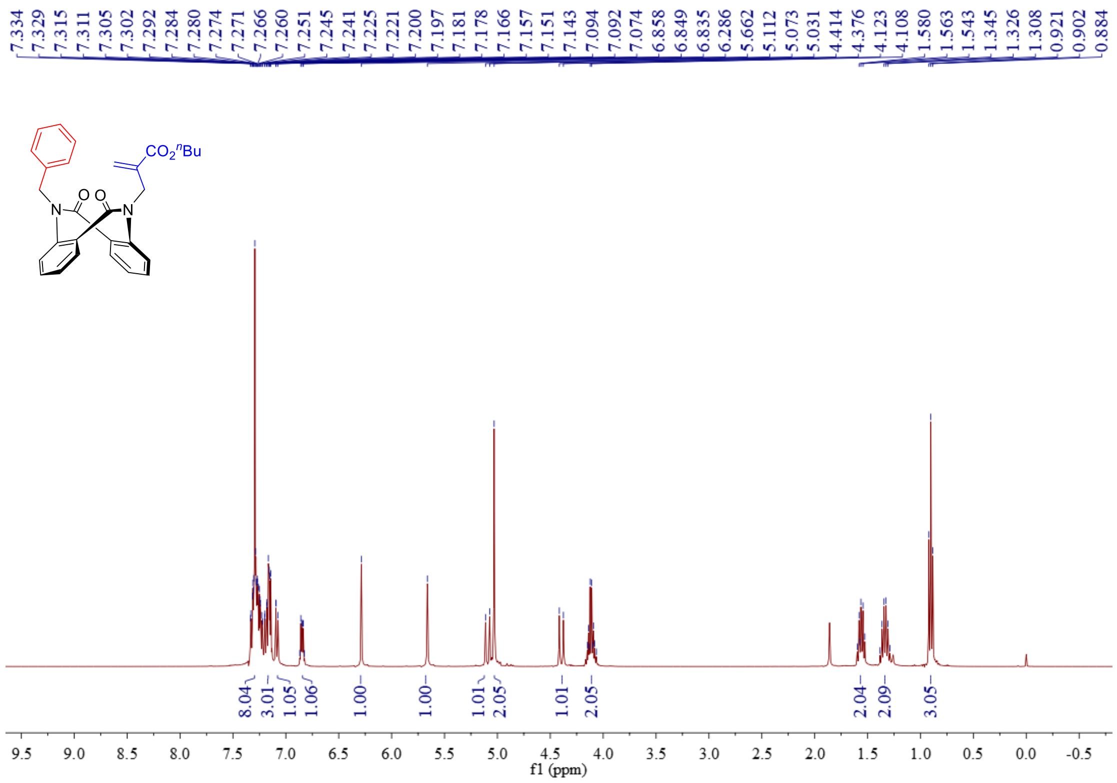

Supplementary Figure 88. ^{1}H NMR spectrum of compound **5c** ($CDCl_3$, 400 MHz)

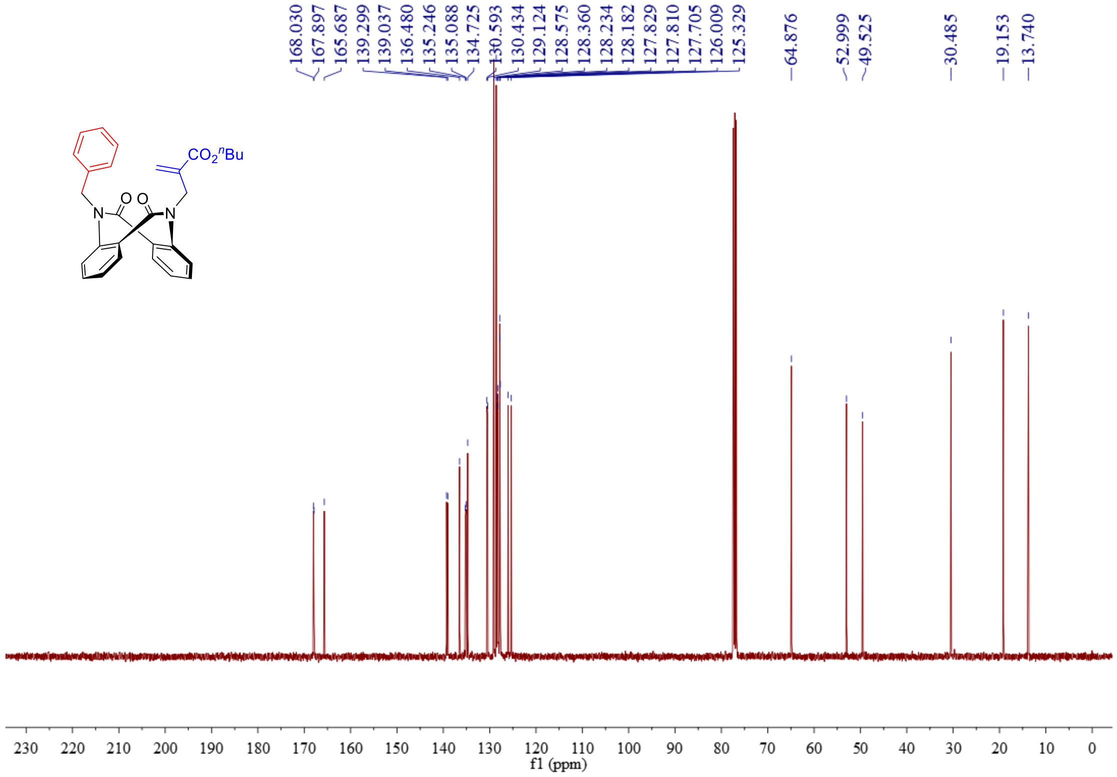

Supplementary Figure 89. ^{13}C NMR spectrum of compound **5c** ($CDCl_3$, 100 MHz)

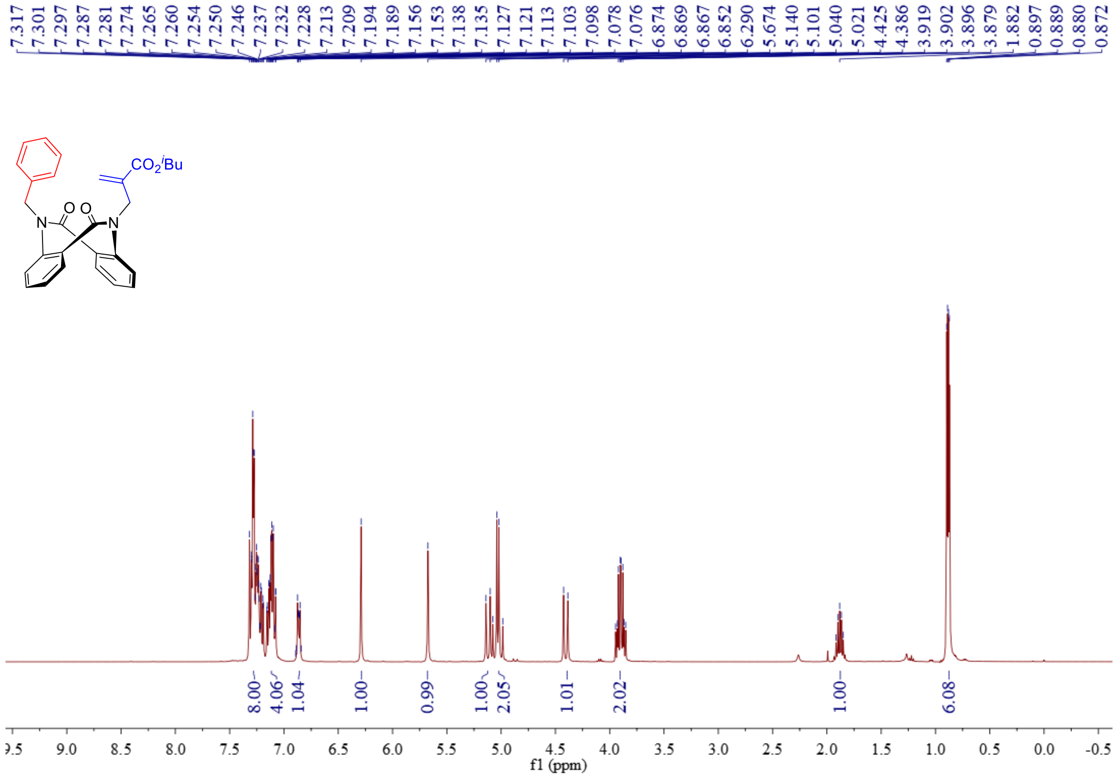

Supplementary Figure 90. ^{1}H NMR spectrum of compound **5d** ($CDCl_3$, 400 MHz)

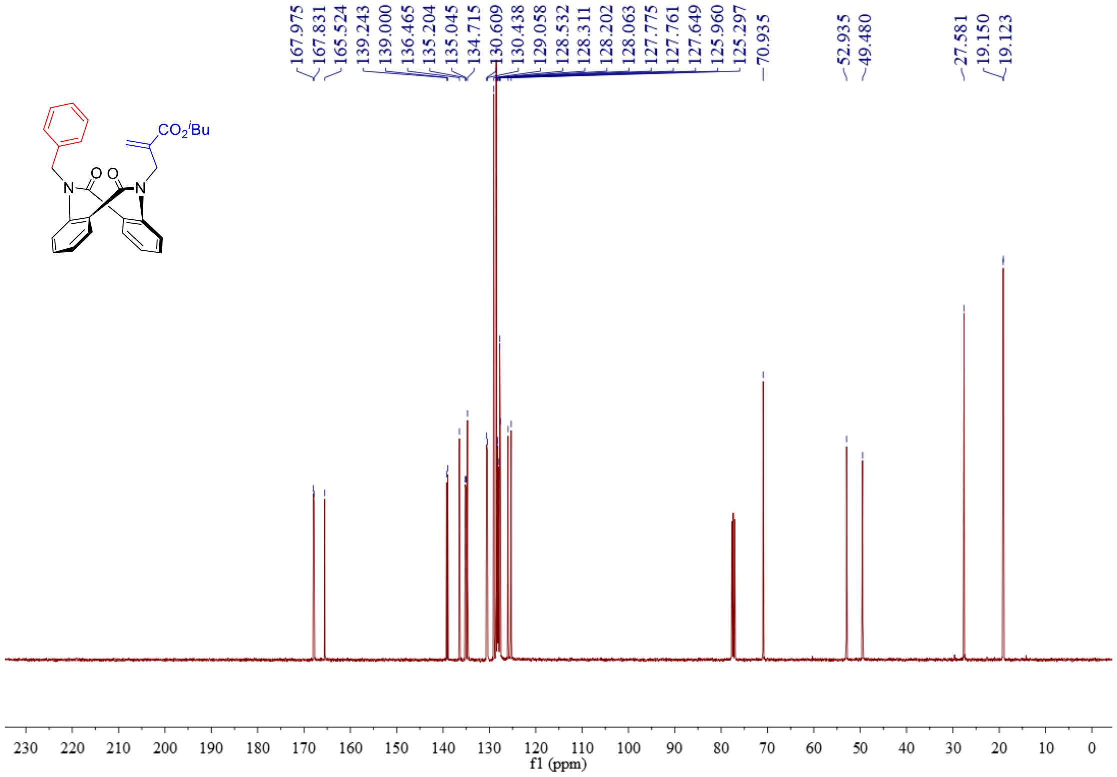

Supplementary Figure 91. ^{13}C NMR spectrum of compound **5d** ($CDCl_3$, 100 MHz)

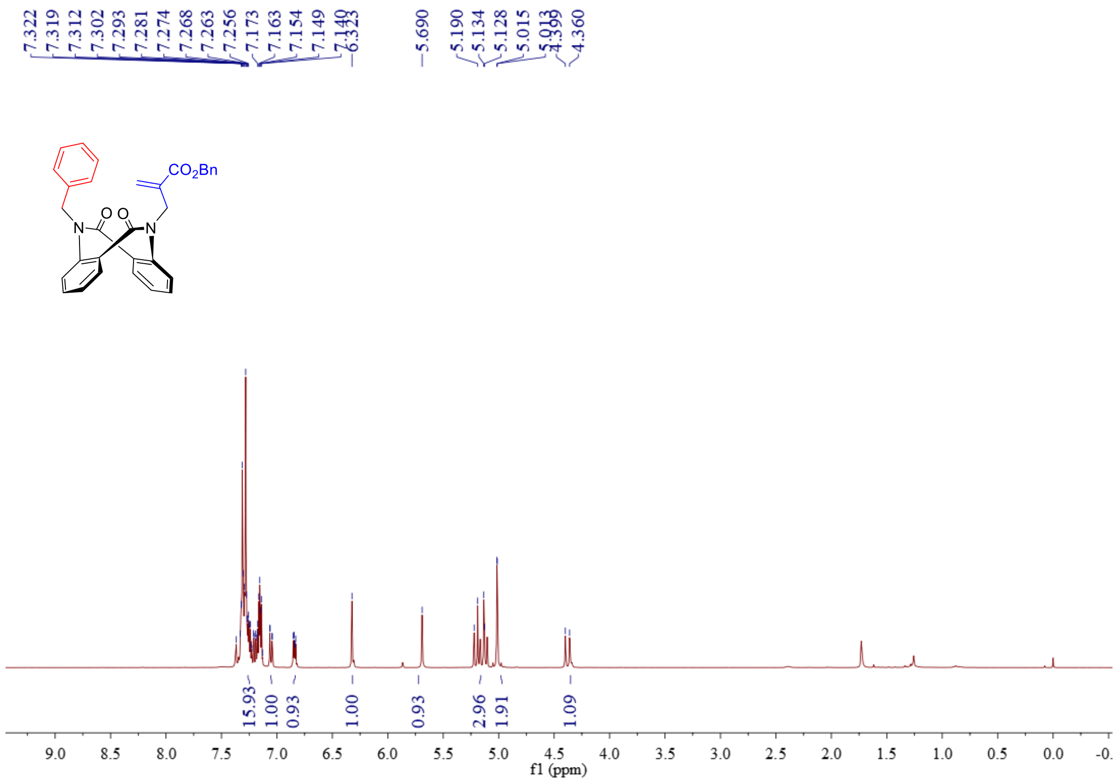

Supplementary Figure 92. ^{1}H NMR spectrum of compound **5e** ($CDCl_3$, 400 MHz)

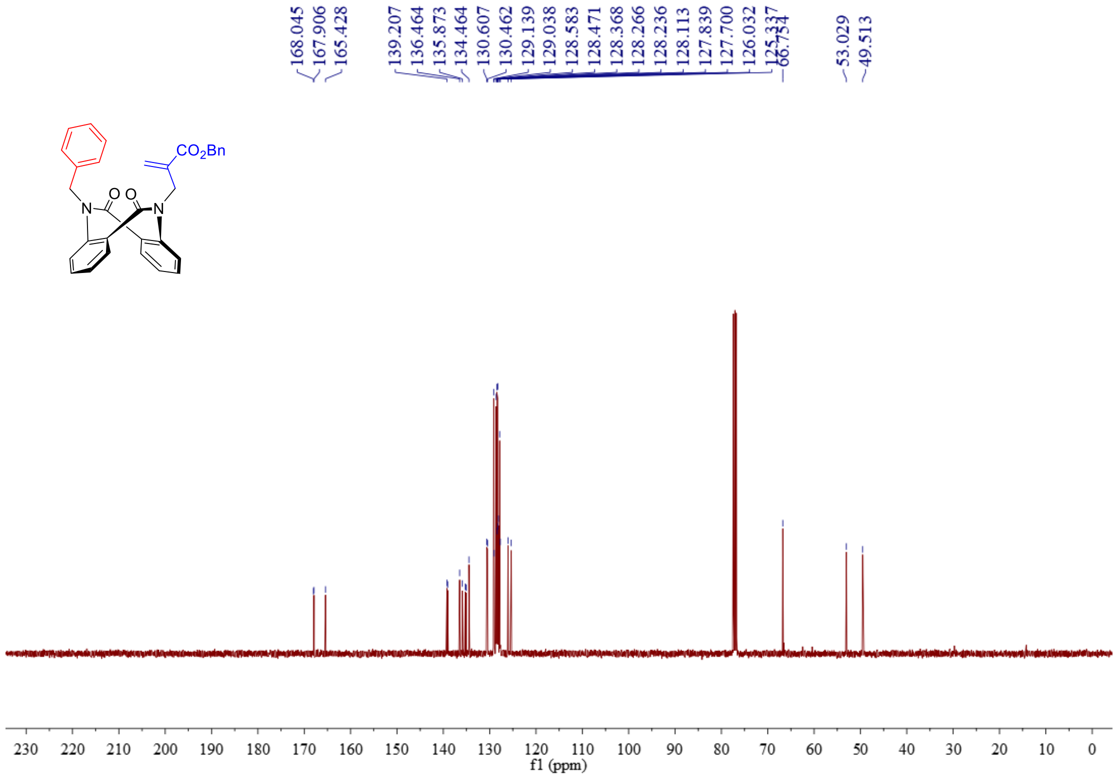

Supplementary Figure 93. ^{13}C NMR spectrum of compound **5e** ($CDCl_3$, 100 MHz)

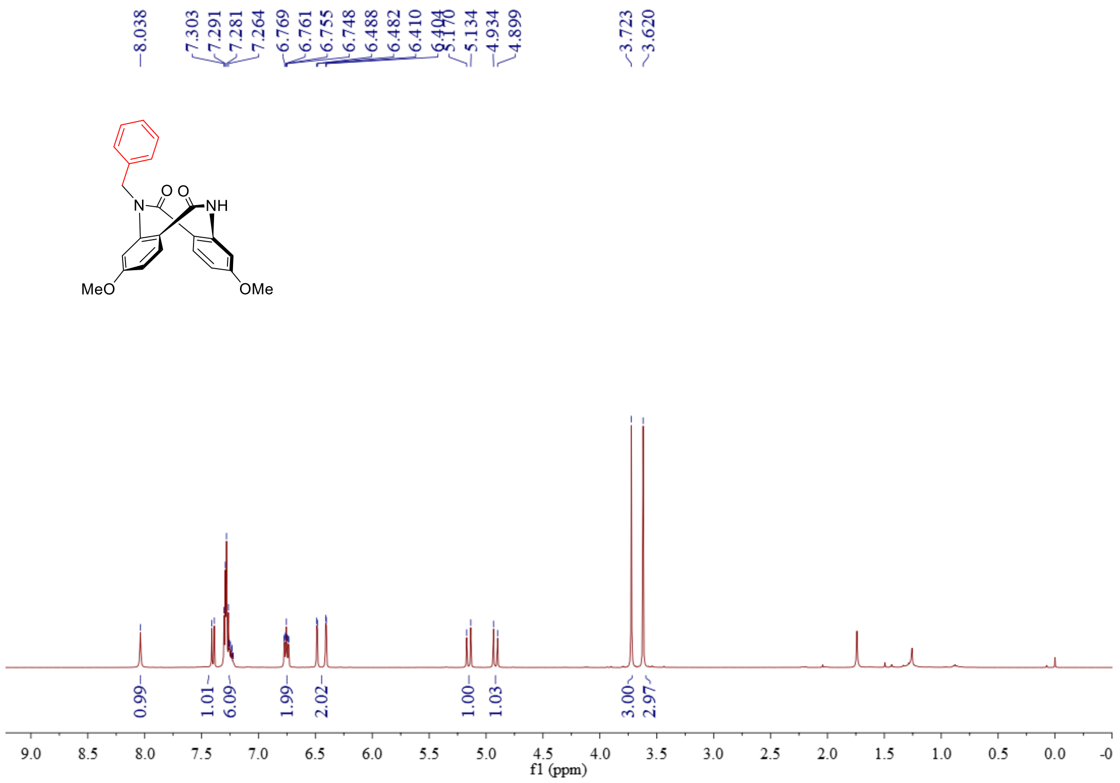

Supplementary Figure 94. ^{1}H NMR spectrum of compound **4f** ($CDCl_3$, 400 MHz)

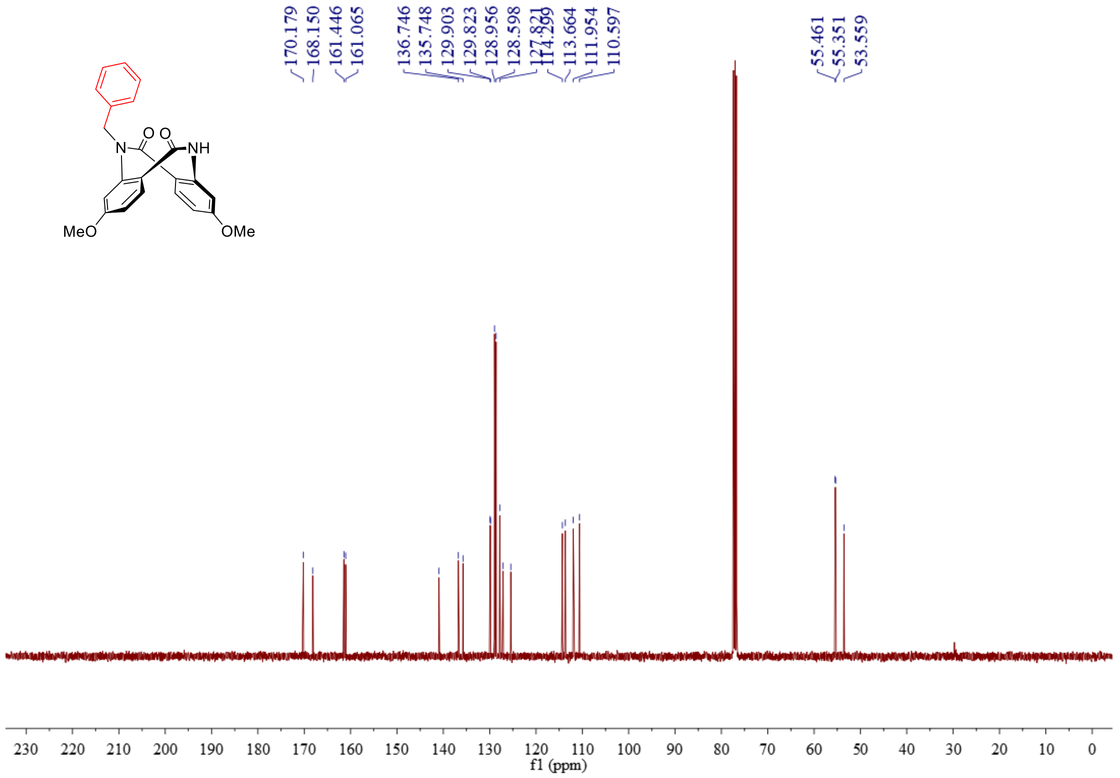

Supplementary Figure 95. ^{13}C NMR spectrum of compound **4f** ($CDCl_3$, 100 MHz)

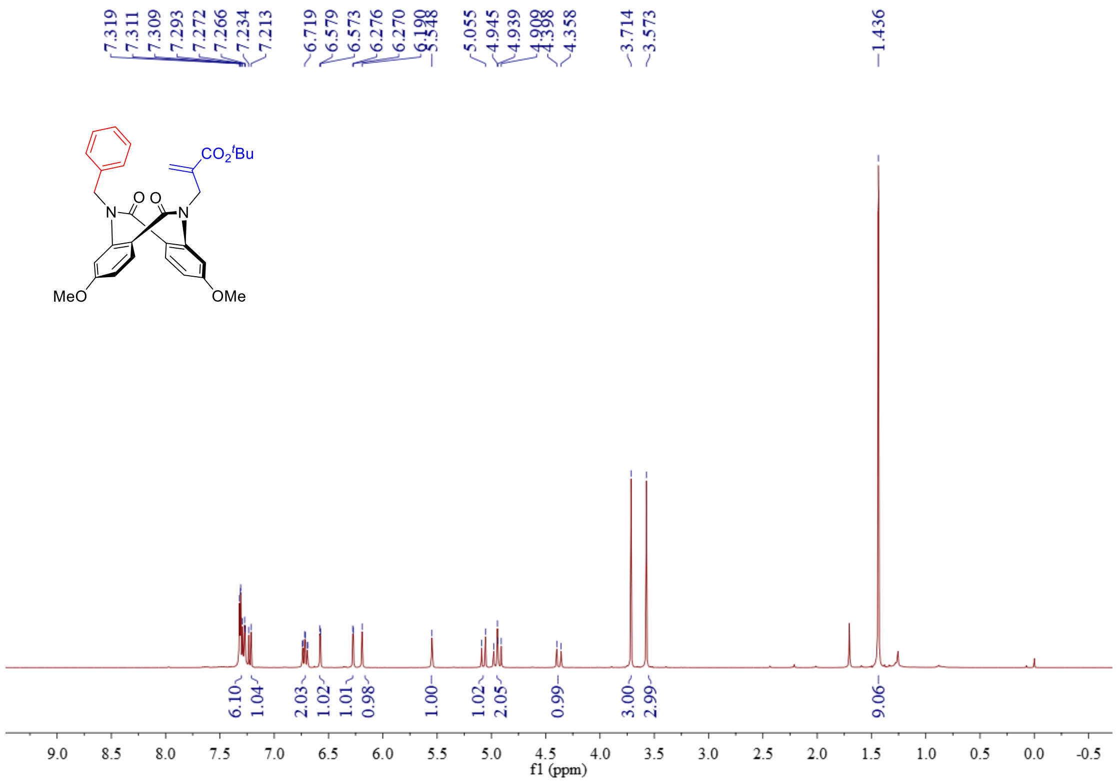

Supplementary Figure 96. ^{1}H NMR spectrum of compound **5f** ($CDCl_3$, 400 MHz)

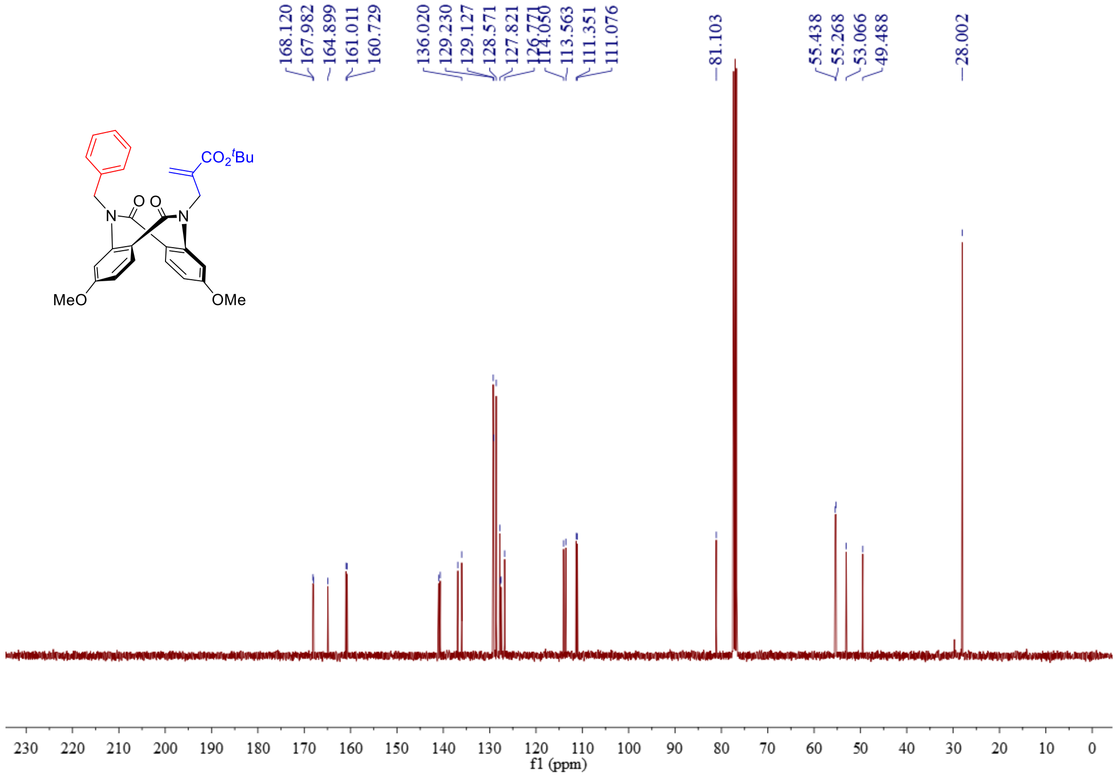

Supplementary Figure 97. ^{13}C NMR spectrum of compound **5f** ($CDCl_3$, 100 MHz)

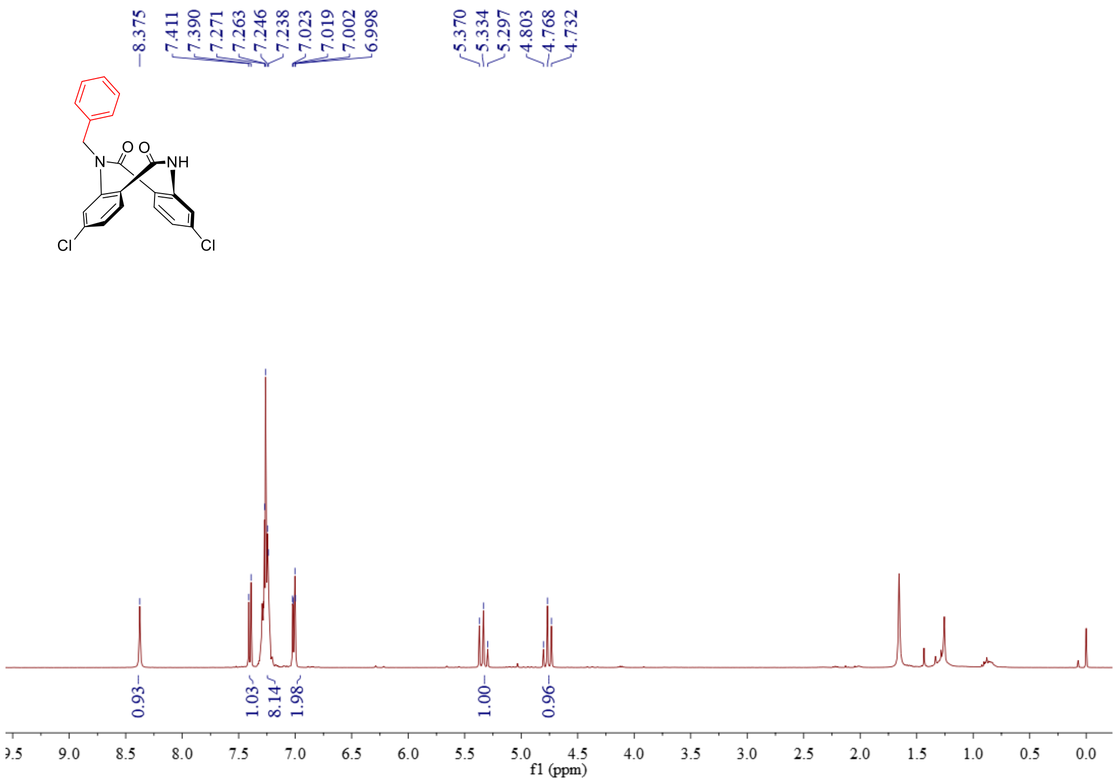

Supplementary Figure 98. ^{1}H NMR spectrum of compound **4g** ($CDCl_3$, 400 MHz)

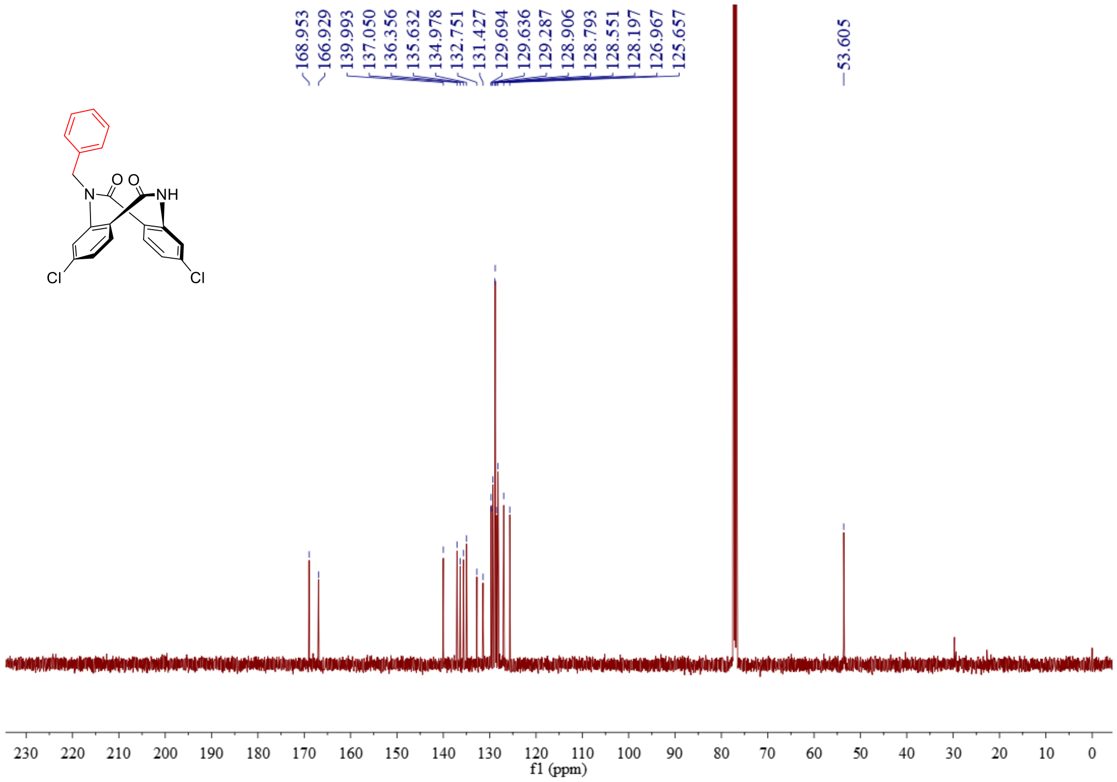

Supplementary Figure 99. ^{13}C NMR spectrum of compound **4g** ($CDCl_3$, 100 MHz)

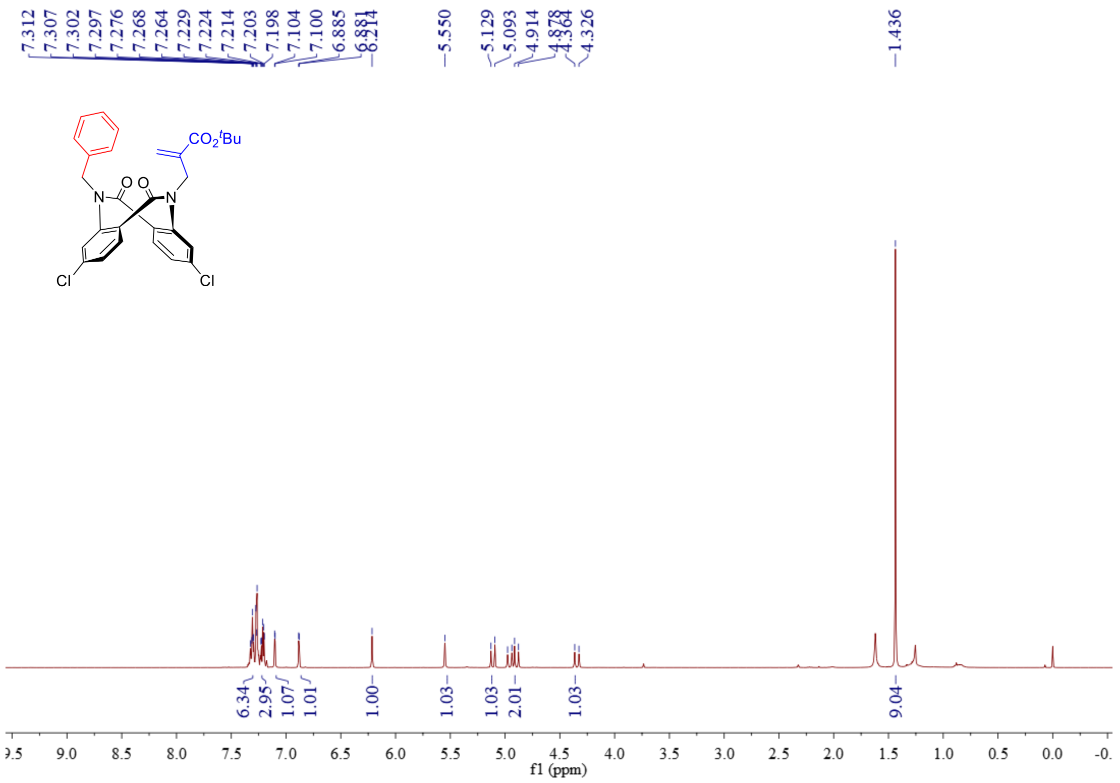

Supplementary Figure 100. ^{1}H NMR spectrum of compound **5g** ($CDCl_3$, 400 MHz)

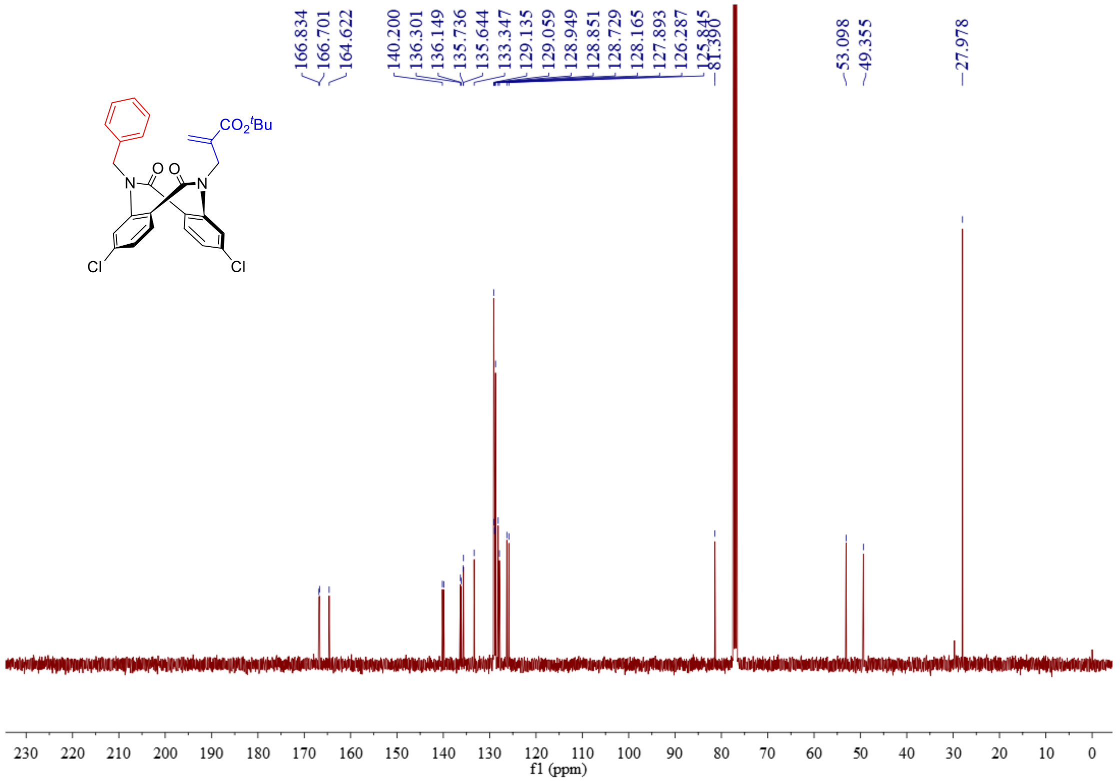

Supplementary Figure 101. ^{13}C NMR spectrum of compound **5g** ($CDCl_3$, 100 MHz)

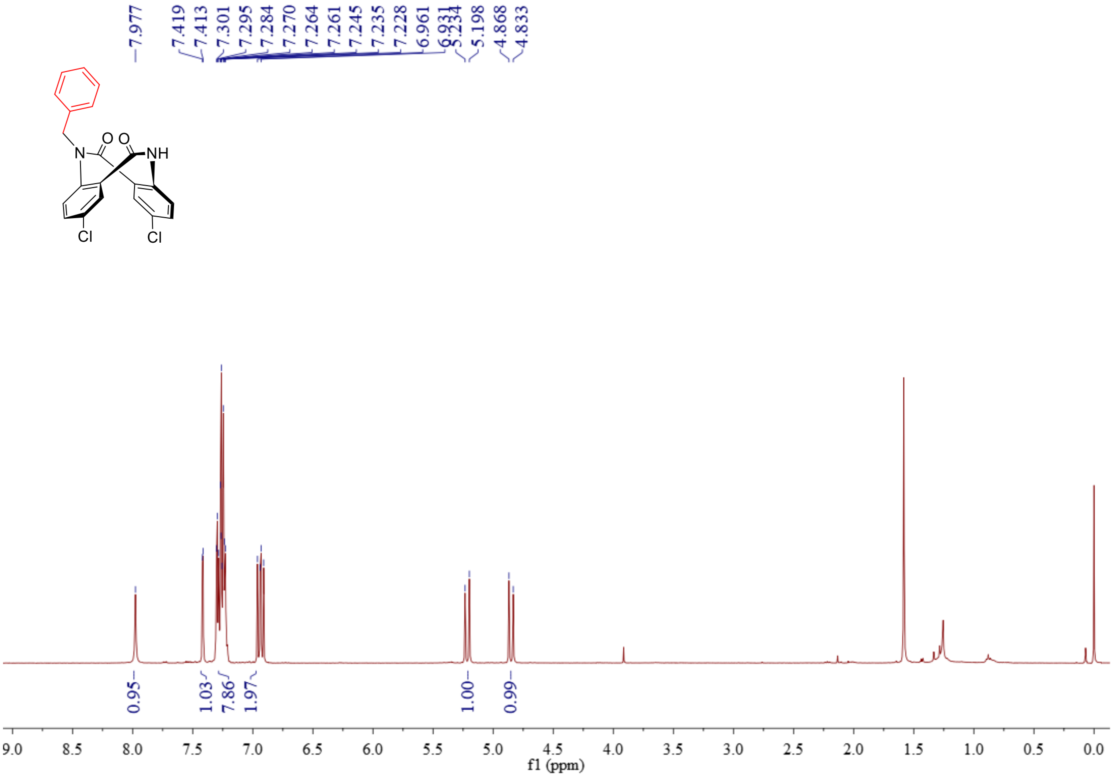

Supplementary Figure 102. ^{1}H NMR spectrum of compound **4h** ($CDCl_3$, 400 MHz)

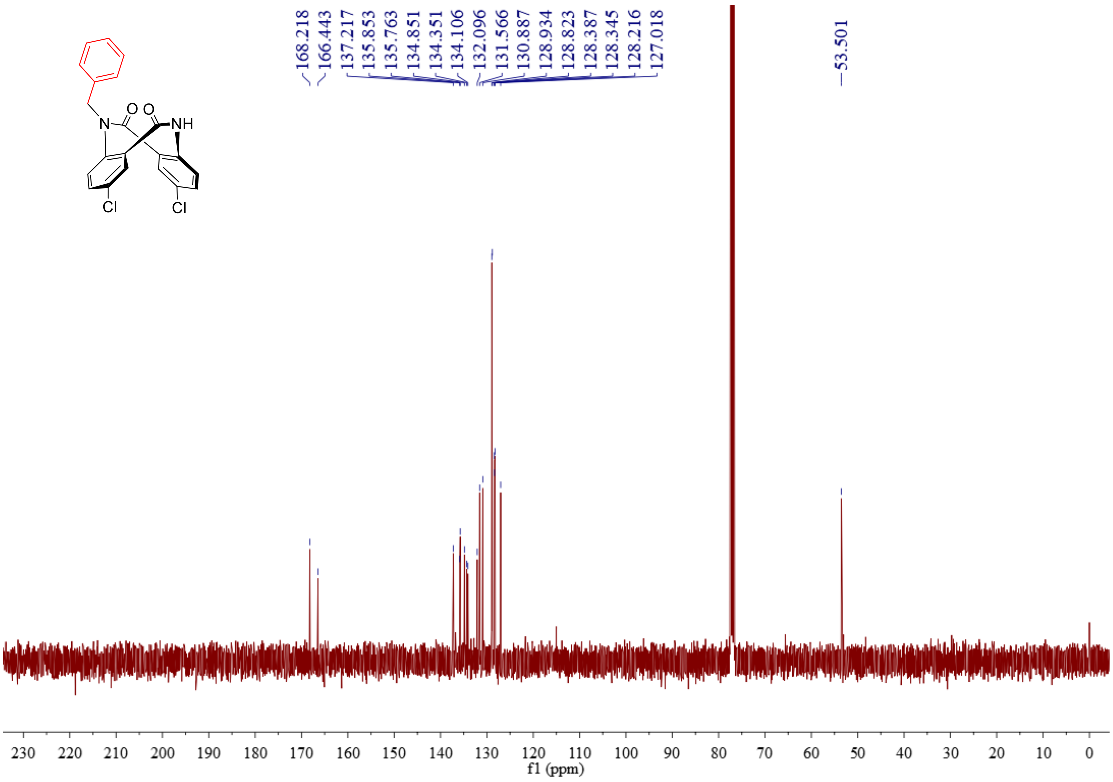

Supplementary Figure 103. ^{13}C NMR spectrum of compound **4h** ($CDCl_3$, 100 MHz)

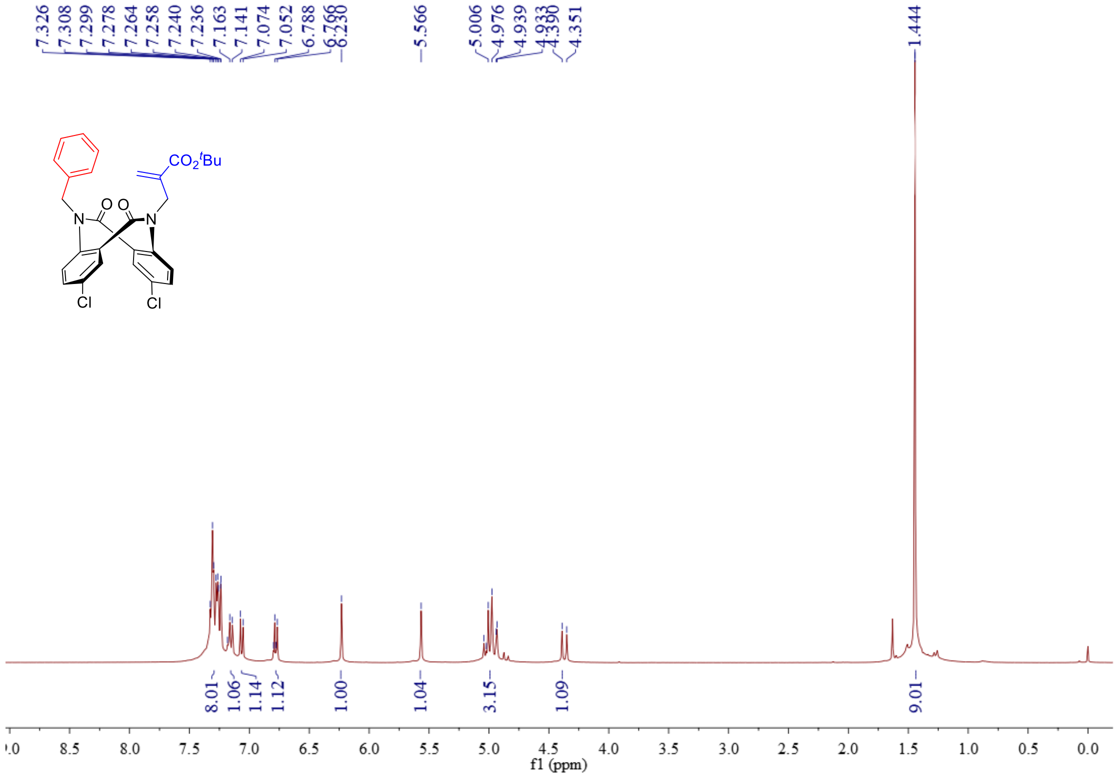

Supplementary Figure 104. ^{1}H NMR spectrum of compound **5h** ($CDCl_3$, 400 MHz)

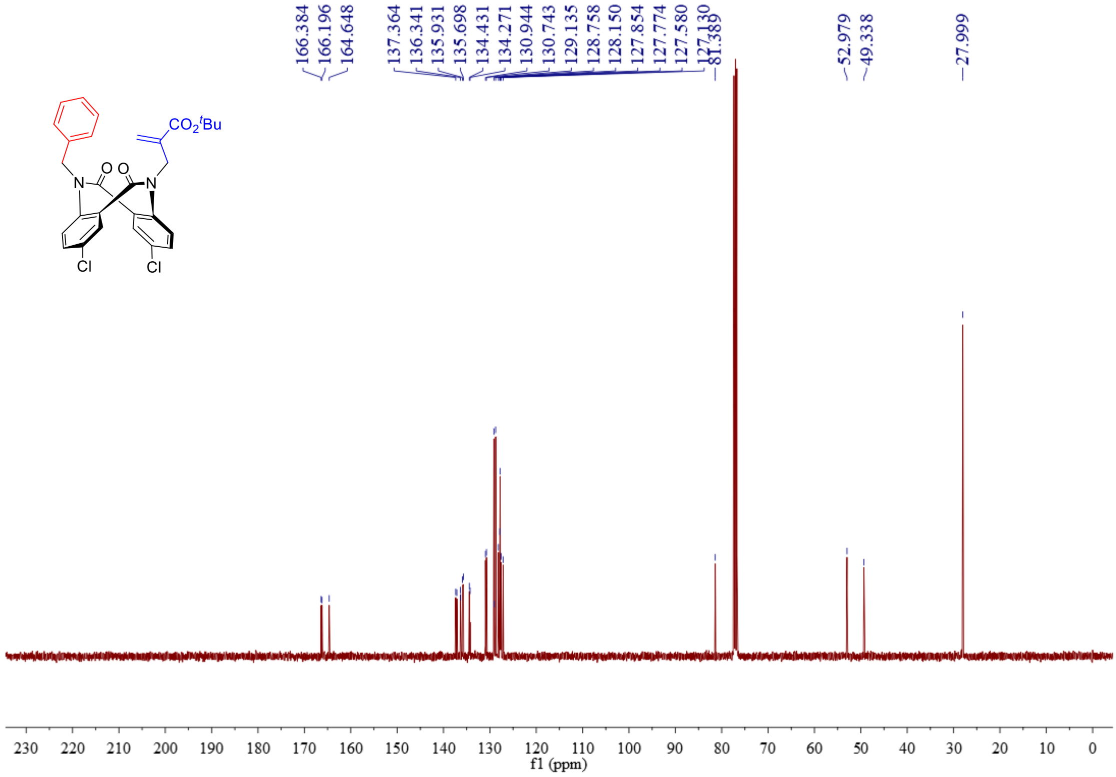

Supplementary Figure 105. ^{13}C NMR spectrum of compound **5h** ($CDCl_3$, 100 MHz)

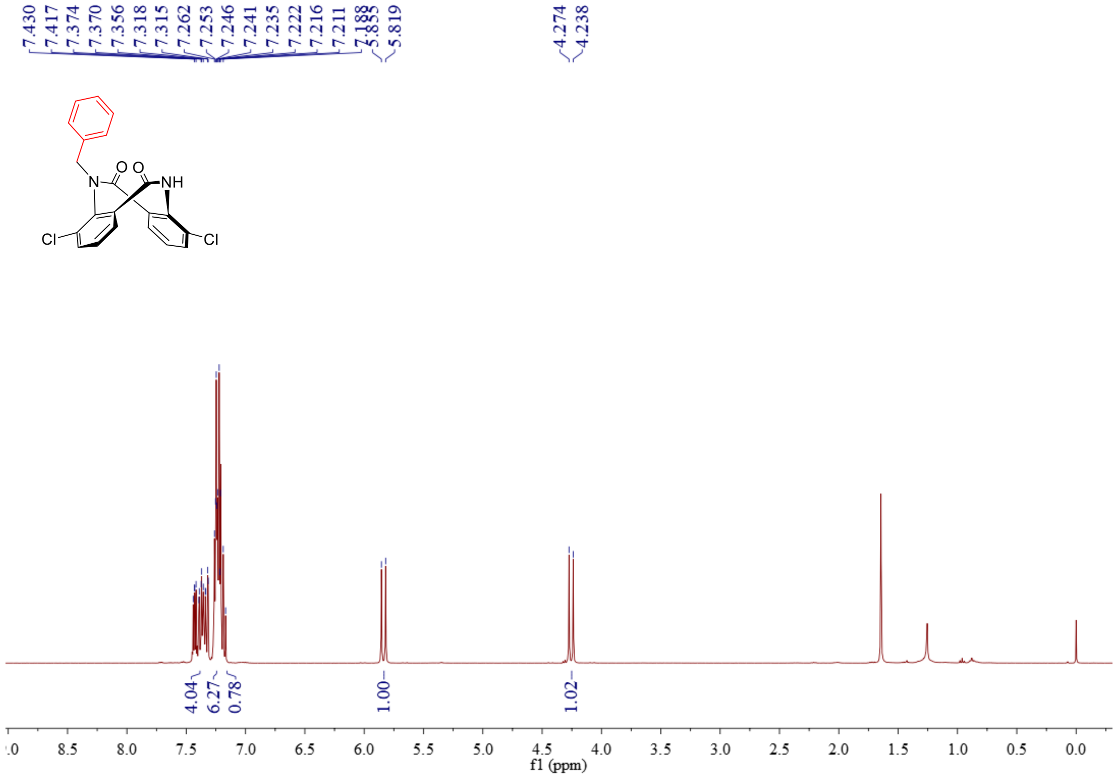

Supplementary Figure 106. ^{1}H NMR spectrum of compound **4i** ($CDCl_3$, 400 MHz)

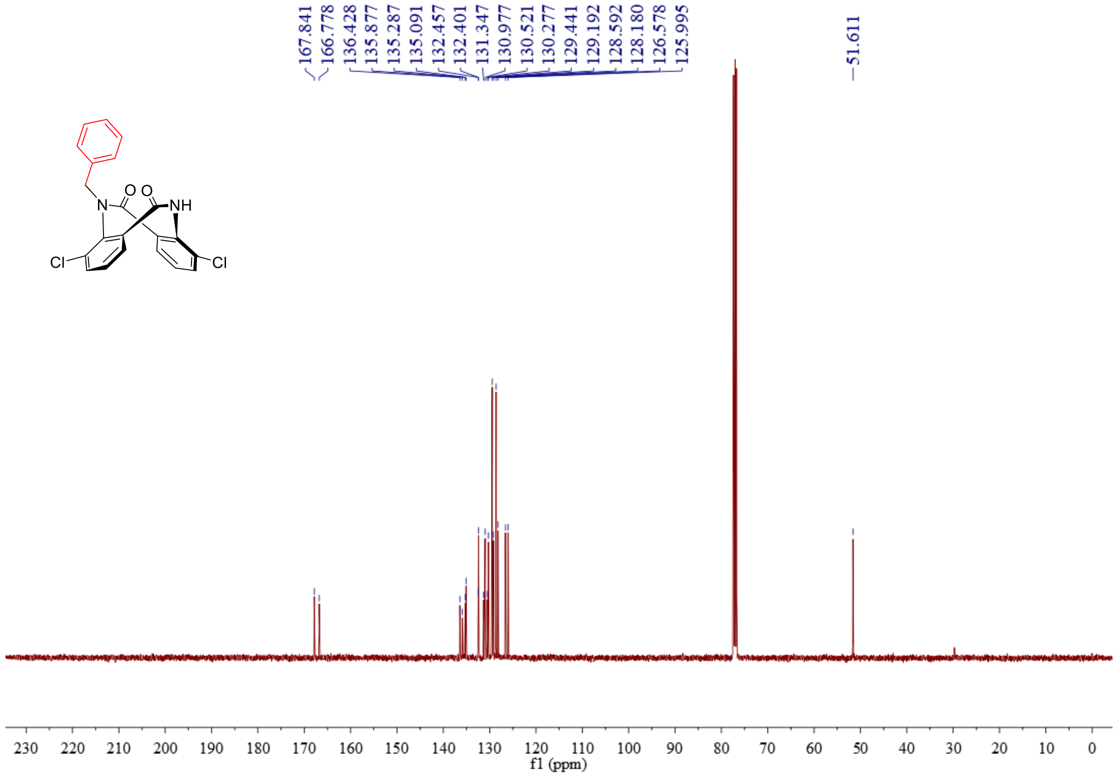

Supplementary Figure 107. ^{13}C NMR spectrum of compound **4i** ($CDCl_3$, 100 MHz)

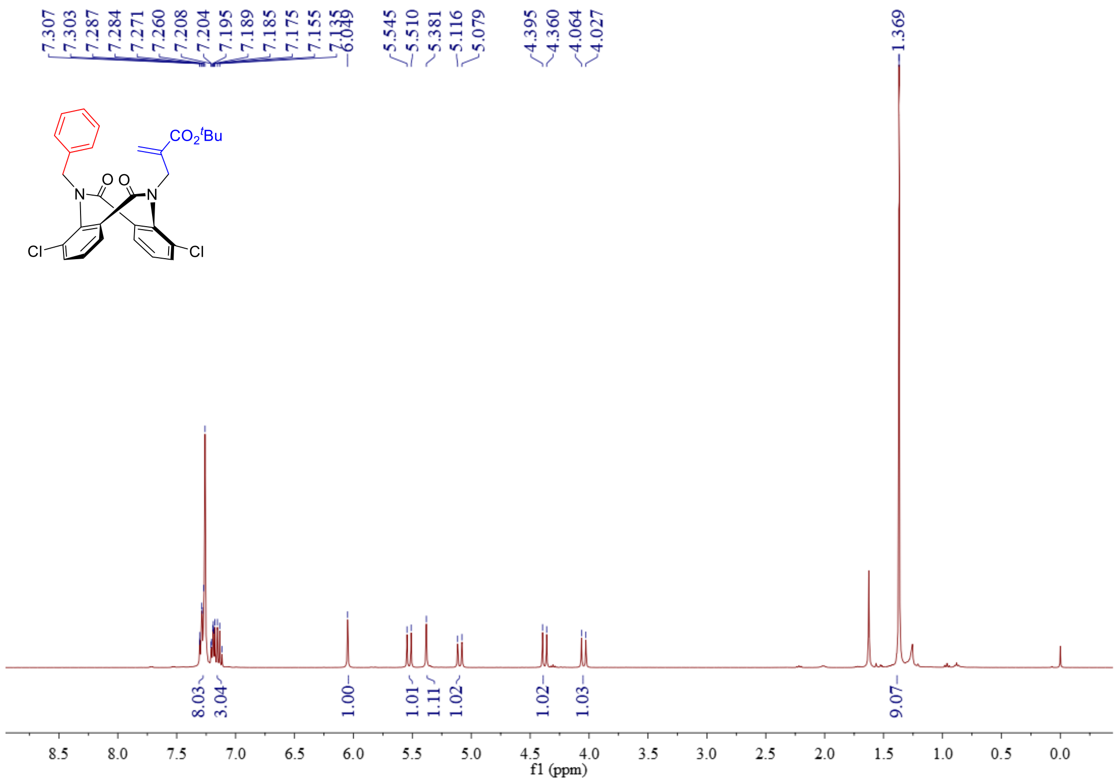

Supplementary Figure 108. ^{1}H NMR spectrum of compound **5i** ($CDCl_3$, 400 MHz)

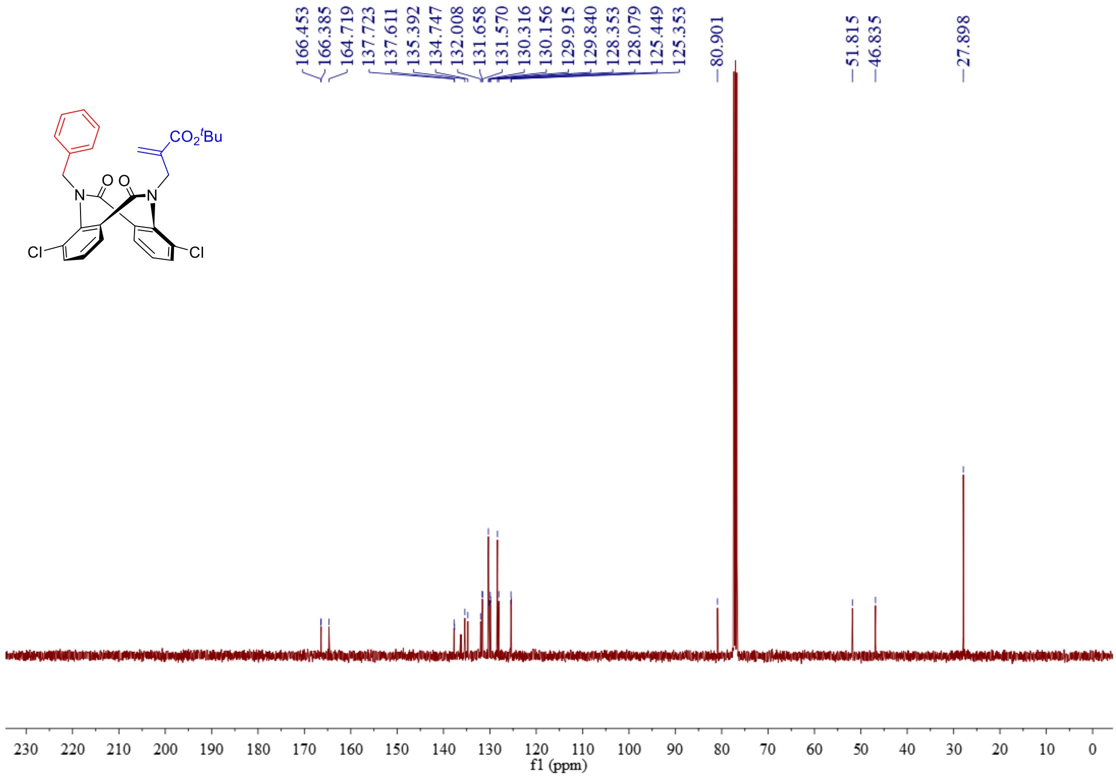

Supplementary Figure 109. ^{13}C NMR spectrum of compound **5i** ($CDCl_3$, 100 MHz)

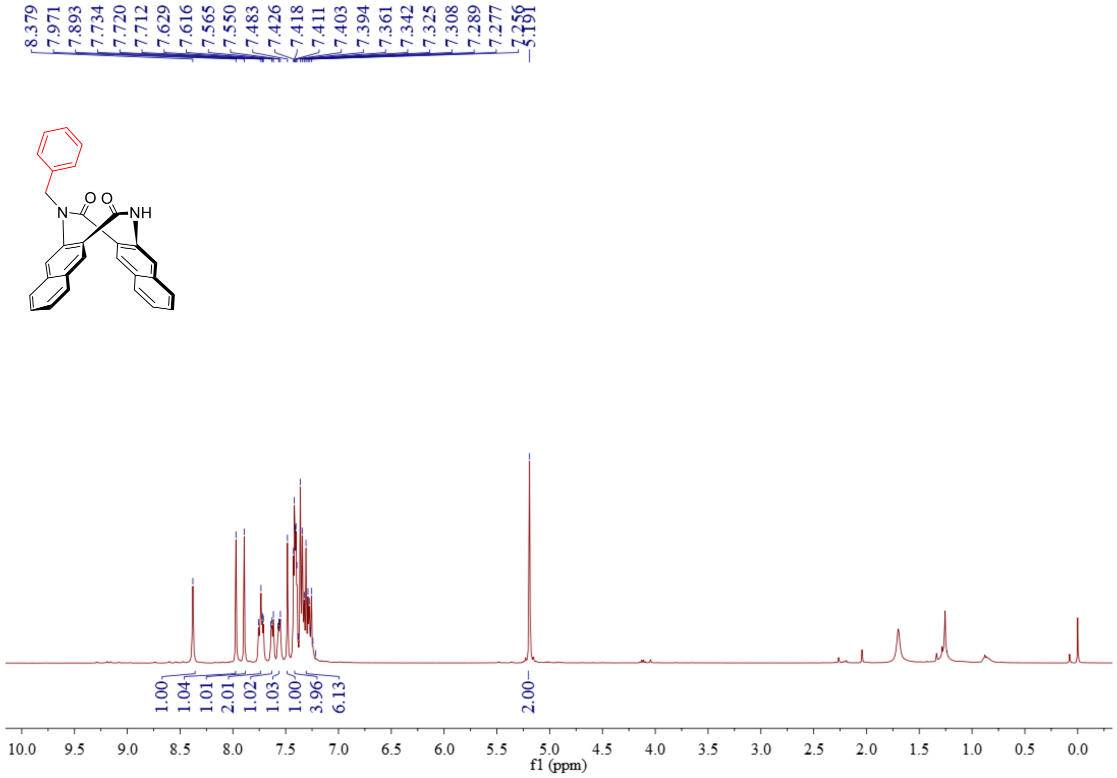

Supplementary Figure 110. ^{1}H NMR spectrum of compound **4j** ($CDCl_3$, 400 MHz)

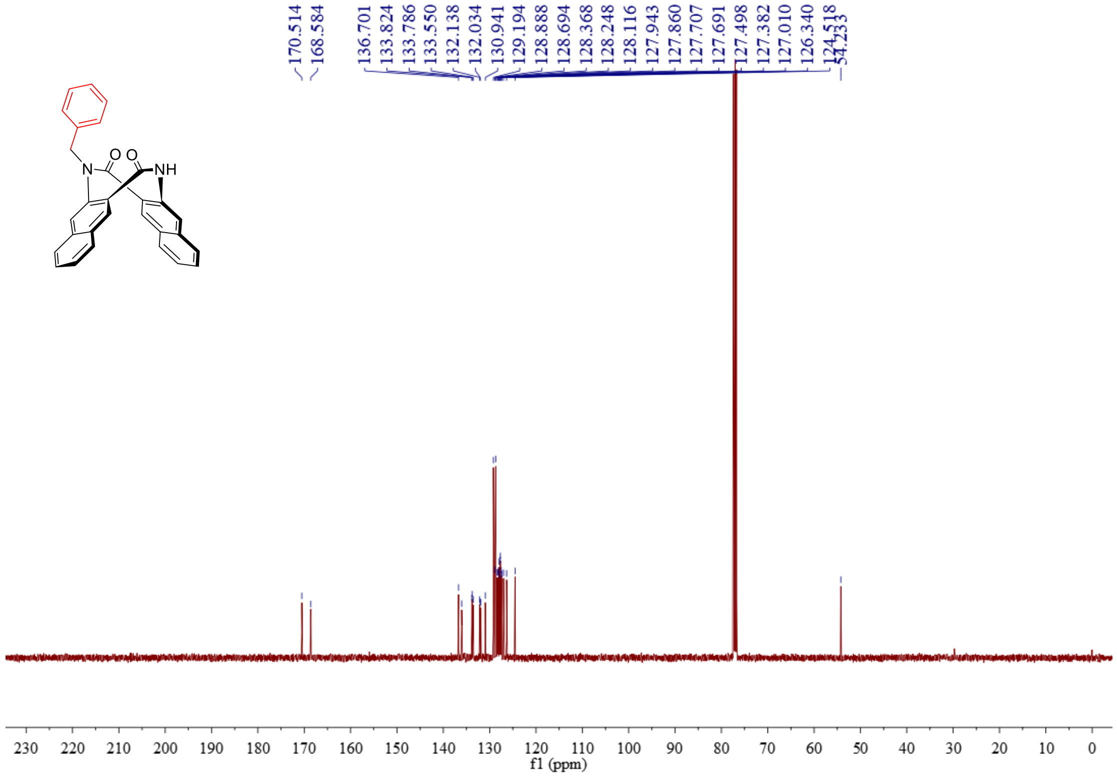

Supplementary Figure 111. ^{13}C NMR spectrum of compound **4j** ($CDCl_3$, 100 MHz)

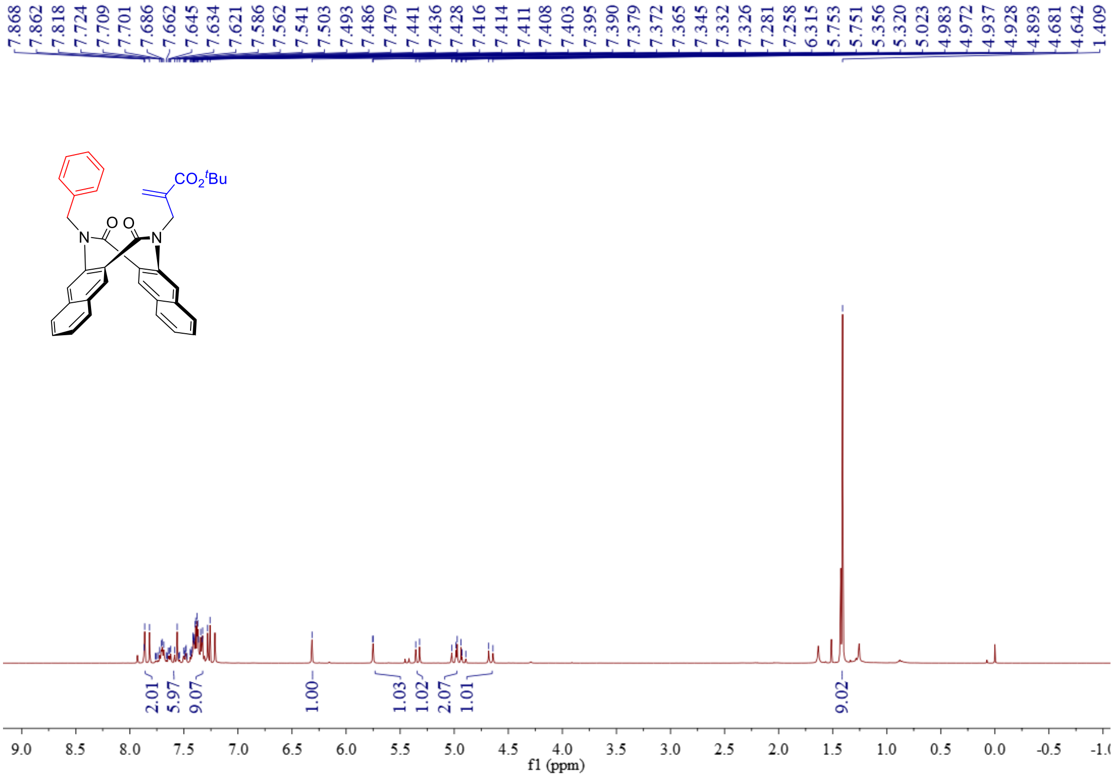

Supplementary Figure 112. ^{1}H NMR spectrum of compound **5j** ($CDCl_3$, 400 MHz)

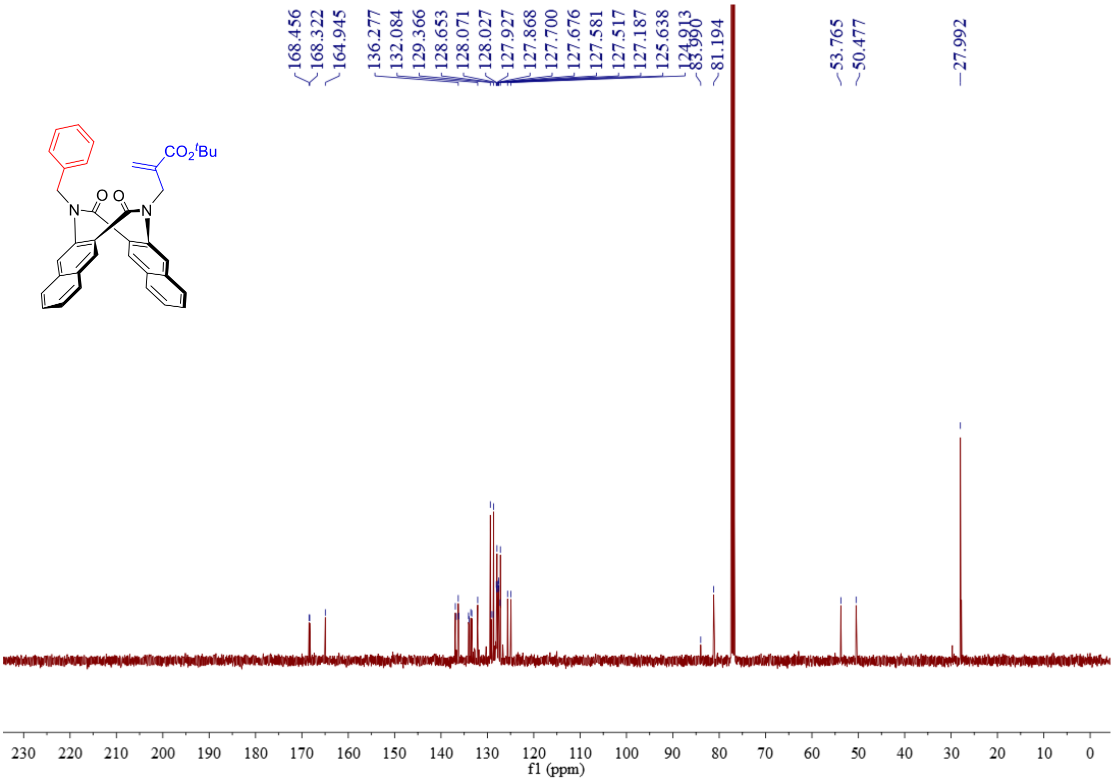

Supplementary Figure 113. ^{13}C NMR spectrum of compound **5j** ($CDCl_3$, 100 MHz)

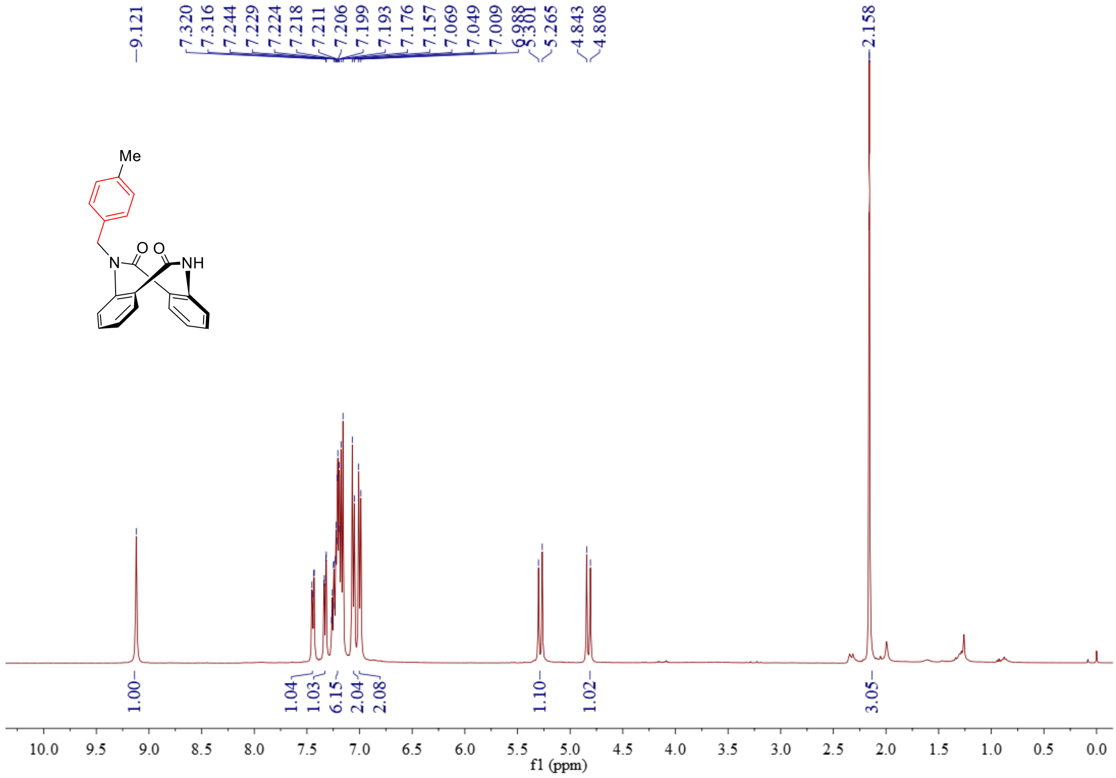

Supplementary Figure 114. ^{1}H NMR spectrum of compound **4k** ($CDCl_3$, 400 MHz)

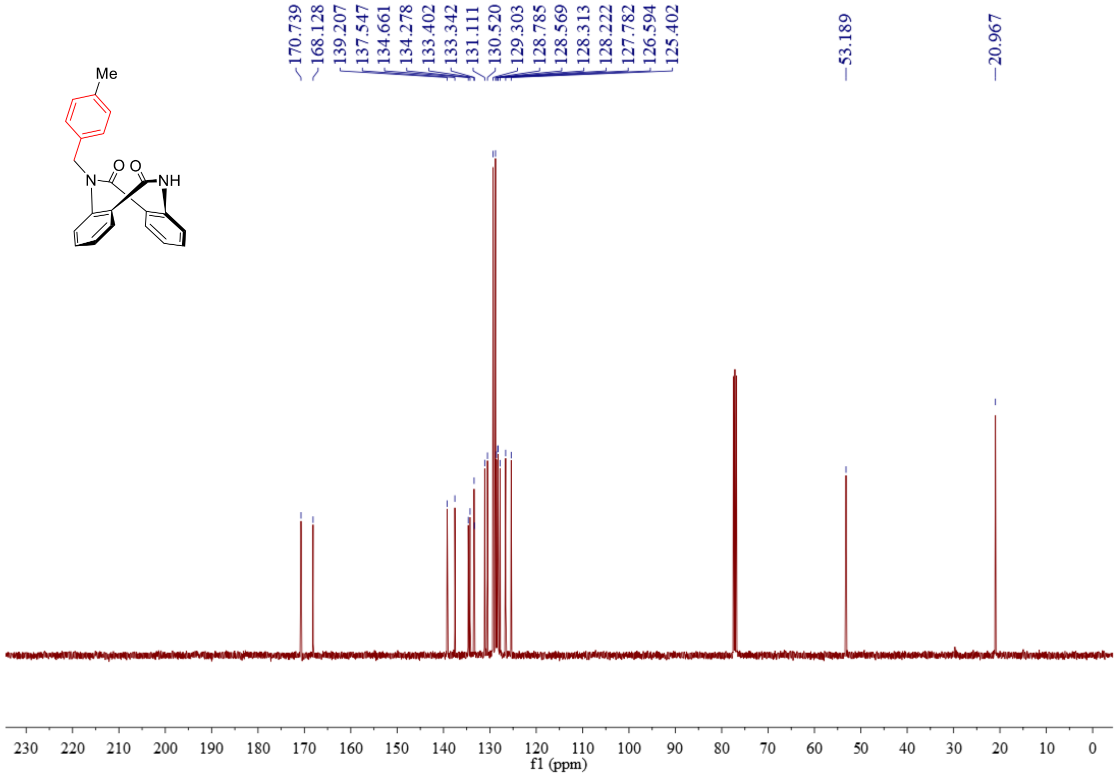

Supplementary Figure 115. ^{13}C NMR spectrum of compound **4k** ($CDCl_3$, 100 MHz)

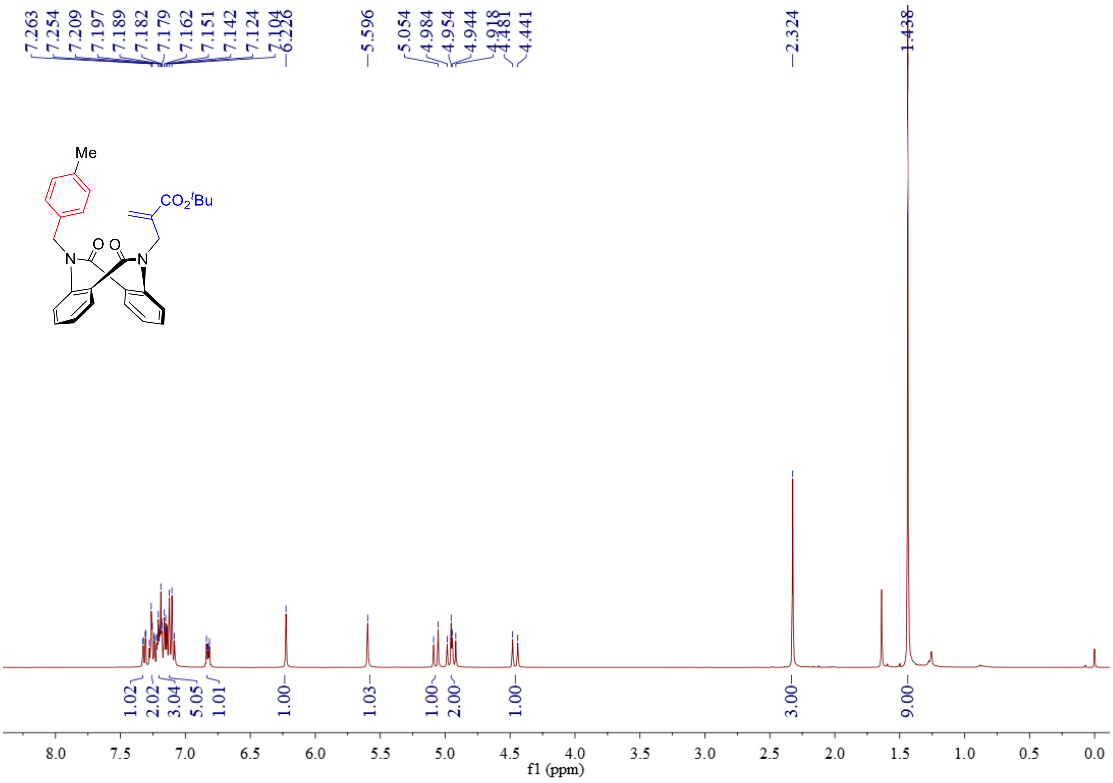

Supplementary Figure 116. ^{1}H NMR spectrum of compound **5k** ($CDCl_3$, 400 MHz)

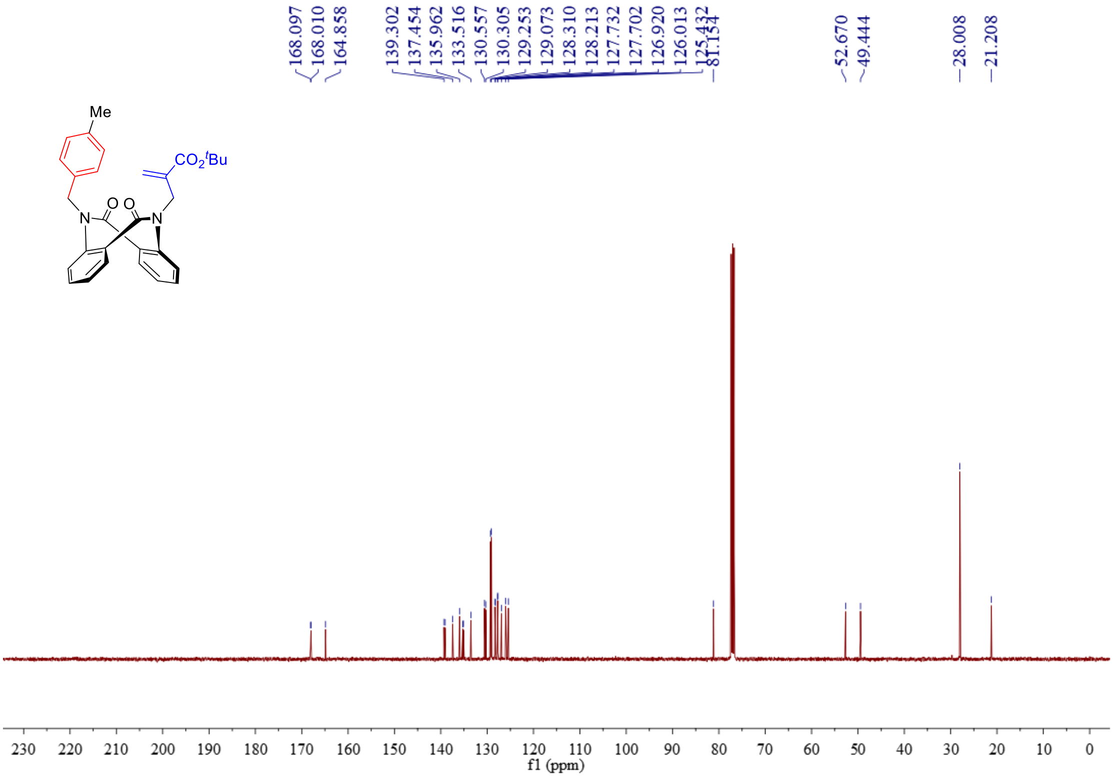

Supplementary Figure 117. ^{13}C NMR spectrum of compound **5k** ($CDCl_3$, 100 MHz)

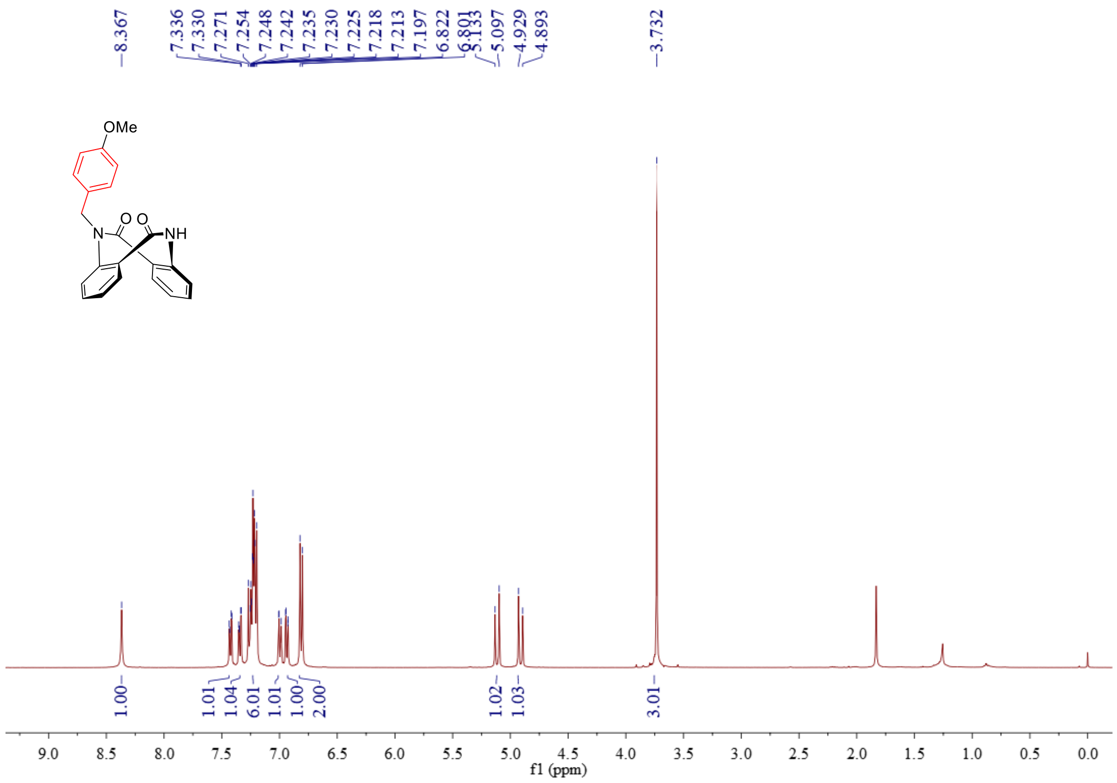

Supplementary Figure 118. ^{1}H NMR spectrum of compound **4l** ($CDCl_3$, 400 MHz)

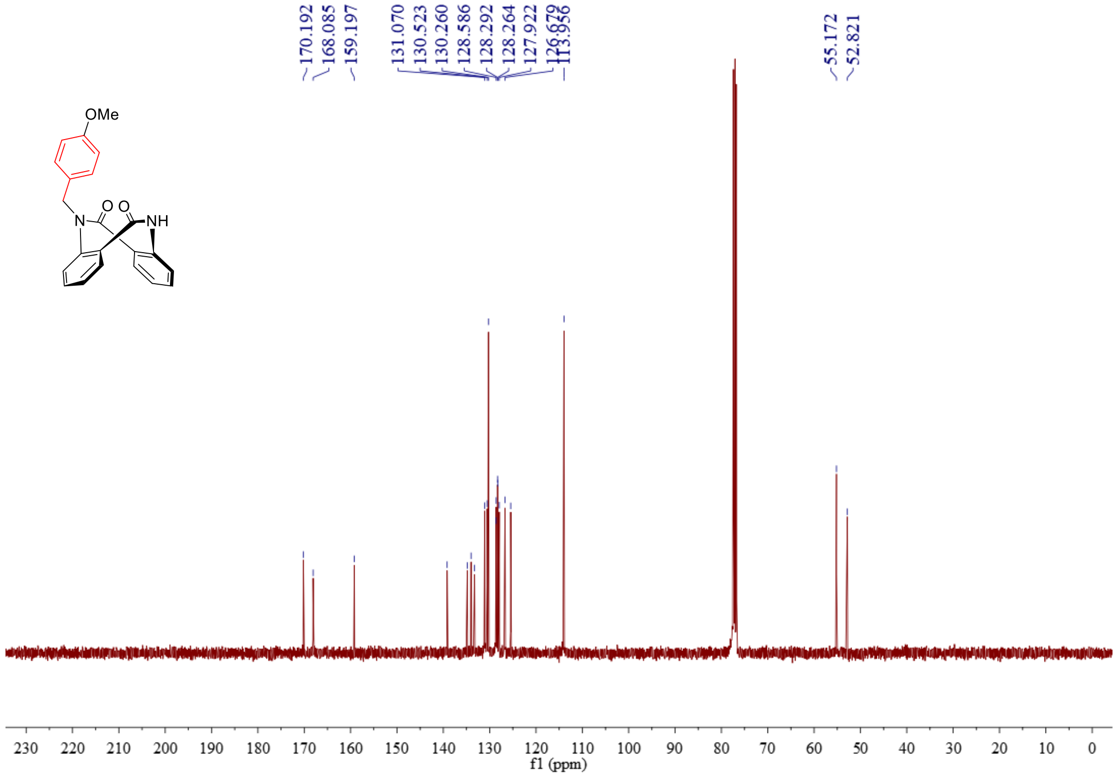

Supplementary Figure 119. ^{13}C NMR spectrum of compound **4l** ($CDCl_3$, 100 MHz)

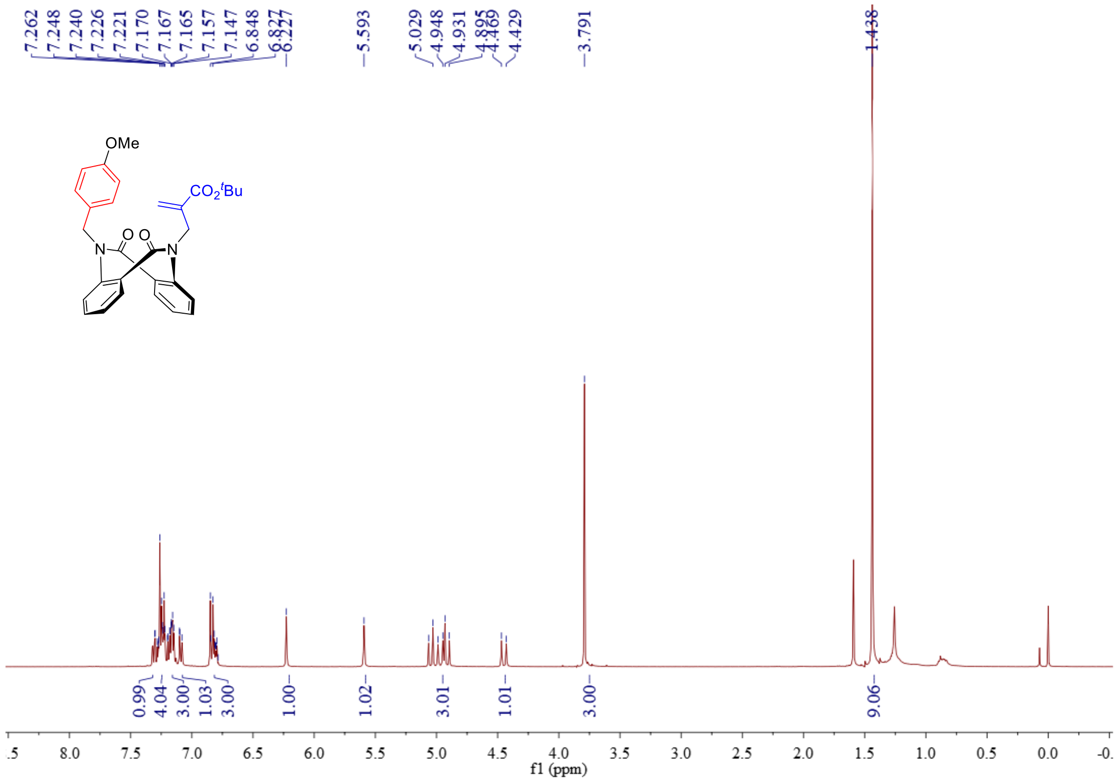

Supplementary Figure 120. ^{1}H NMR spectrum of compound **5l** ($CDCl_3$, 400 MHz)

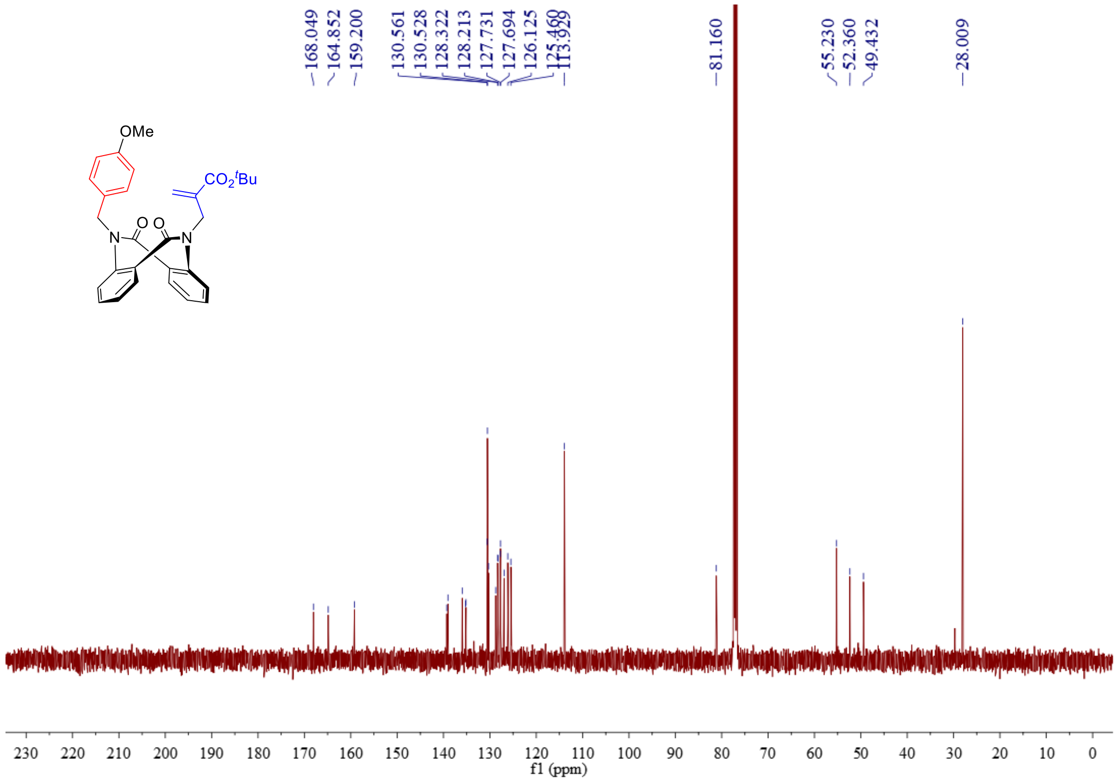

Supplementary Figure 121. ^{13}C NMR spectrum of compound **5l** ($CDCl_3$, 100 MHz)

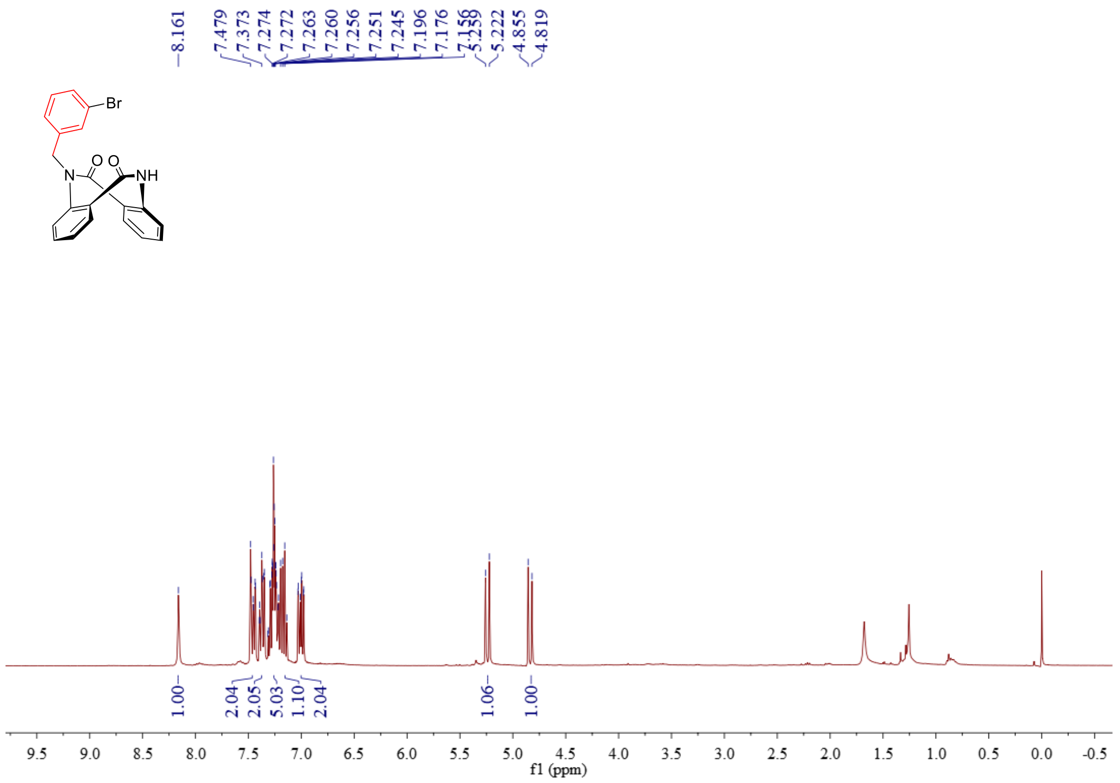

Supplementary Figure 122. ^{1}H NMR spectrum of compound **4m** ($CDCl_3$, 400 MHz)

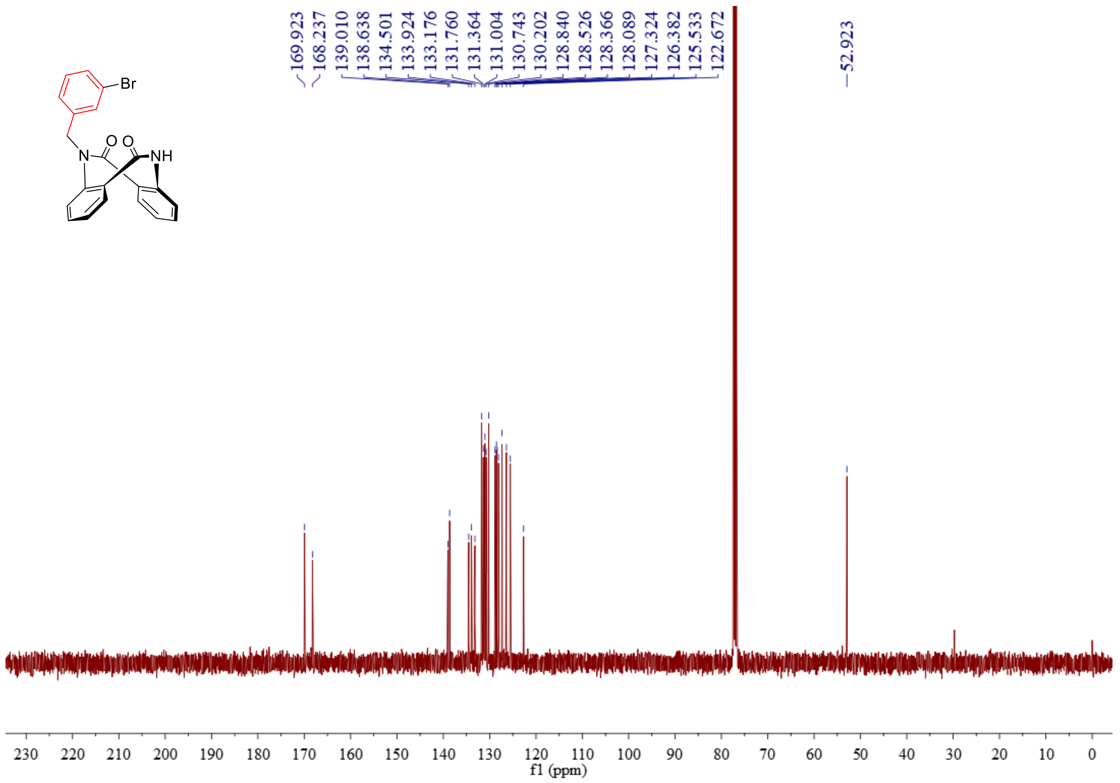

Supplementary Figure 123. ^{13}C NMR spectrum of compound **4m** ($CDCl_3$, 100 MHz)

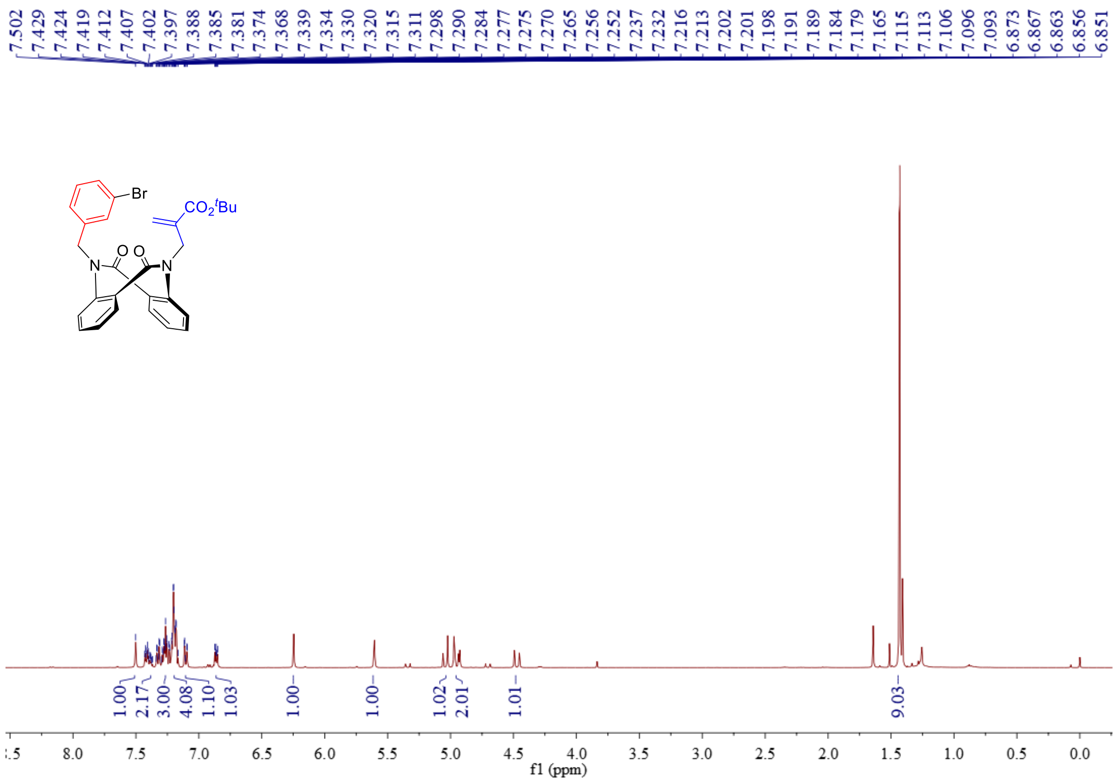

Supplementary Figure 124. ^{1}H NMR spectrum of compound **5m** ($CDCl_3$, 400 MHz)

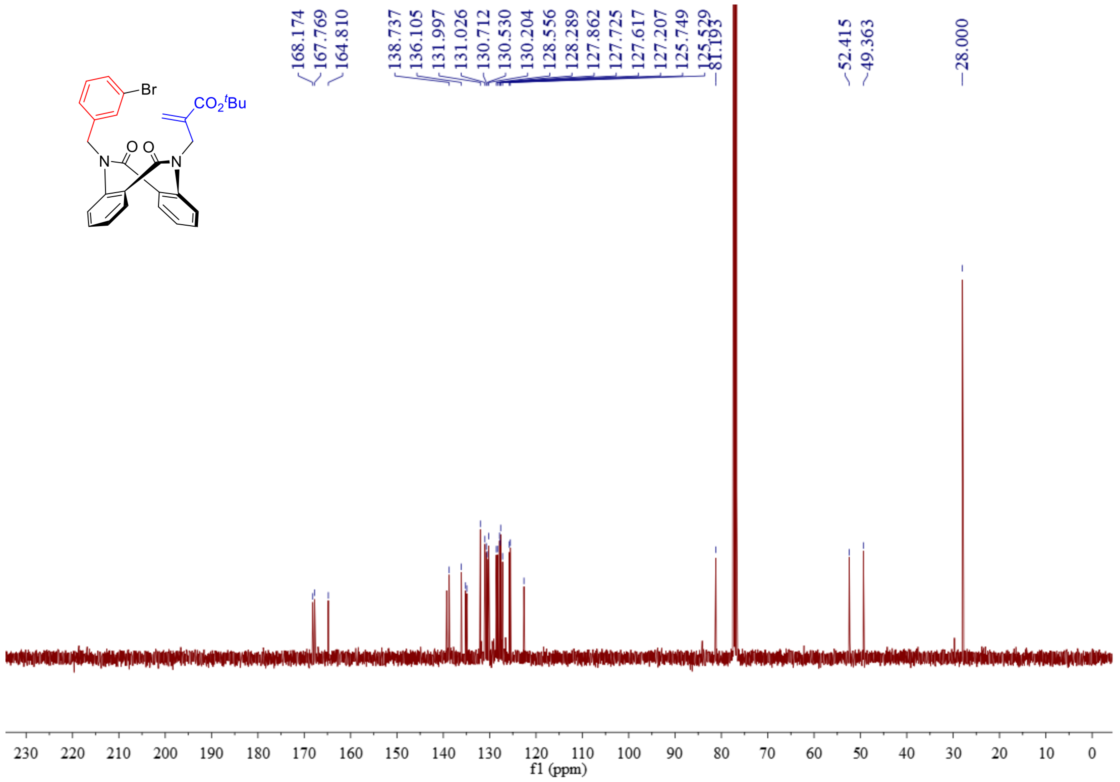

Supplementary Figure 125. ^{13}C NMR spectrum of compound **4m** ($CDCl_3$, 100 MHz)

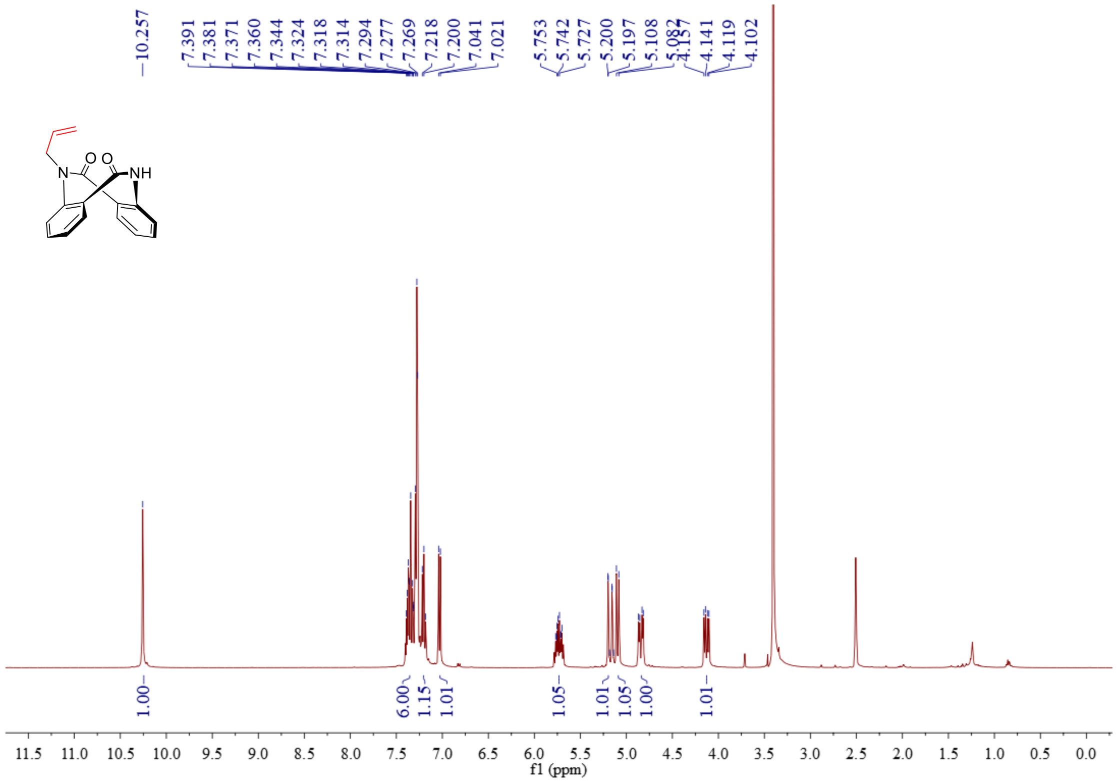

Supplementary Figure 126. ^{1}H NMR spectrum of compound **4n** ($(CD_3)_2SO$, 400 MHz)

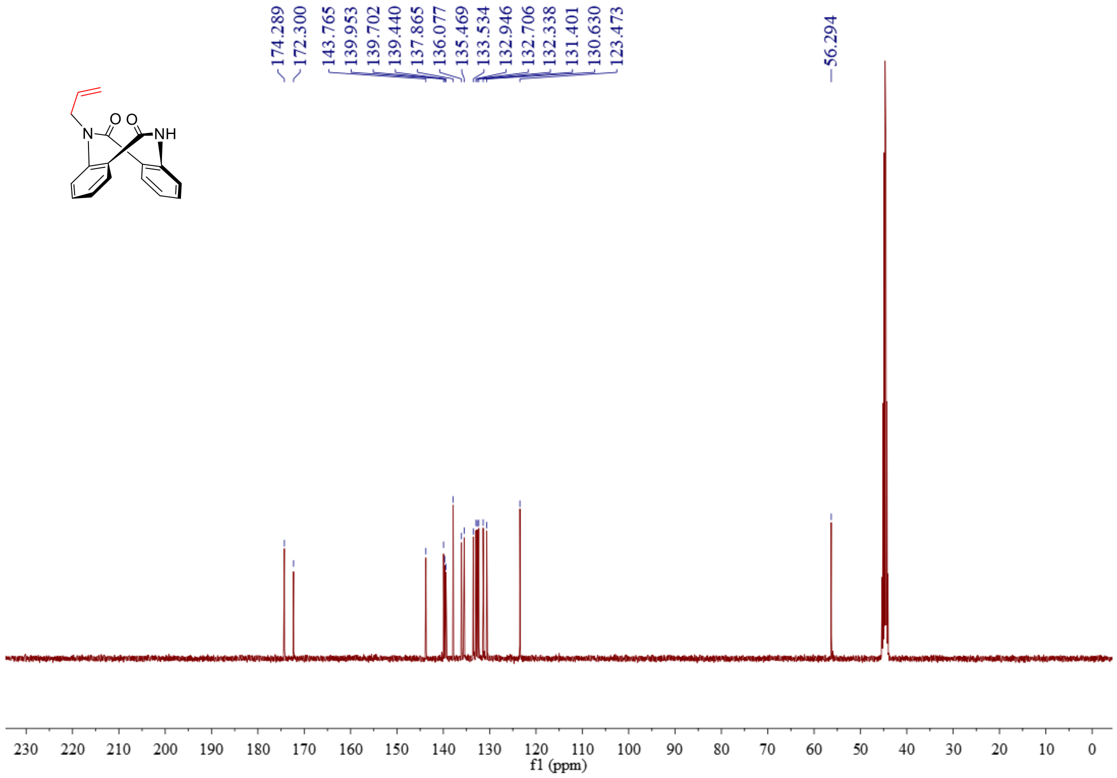

Supplementary Figure 127. ^{13}C NMR spectrum of compound **4n** ($(CD_3)_2SO$, 100 MHz)

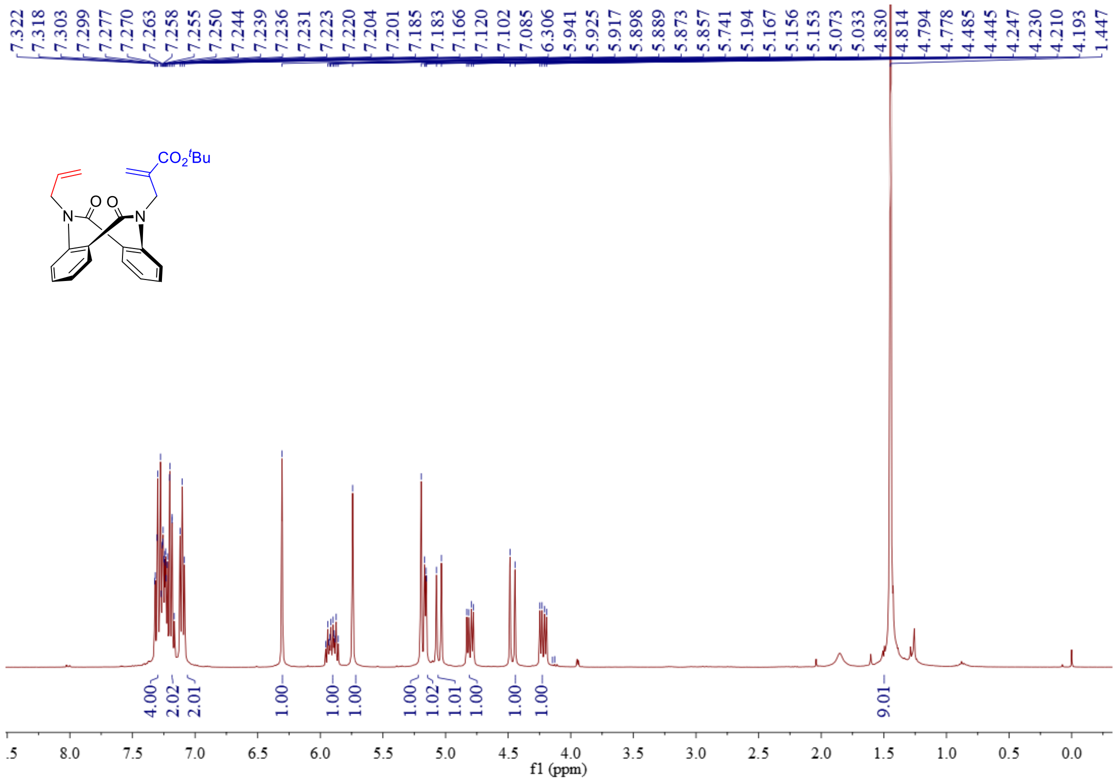

Supplementary Figure 128. ^{1}H NMR spectrum of compound **5n**(CDCl$_3$, 400 MHz)

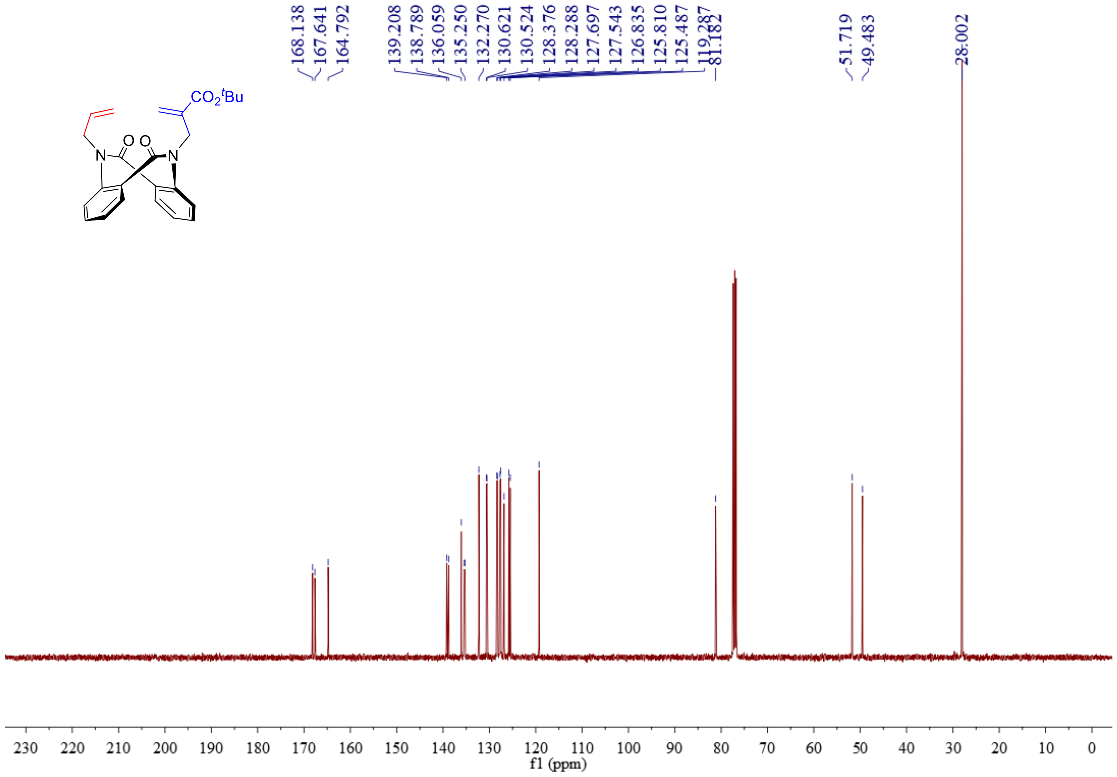

Supplementary Figure 129. ^{13}C NMR spectrum of compound **5n** (CDCl$_3$, 100 MHz)

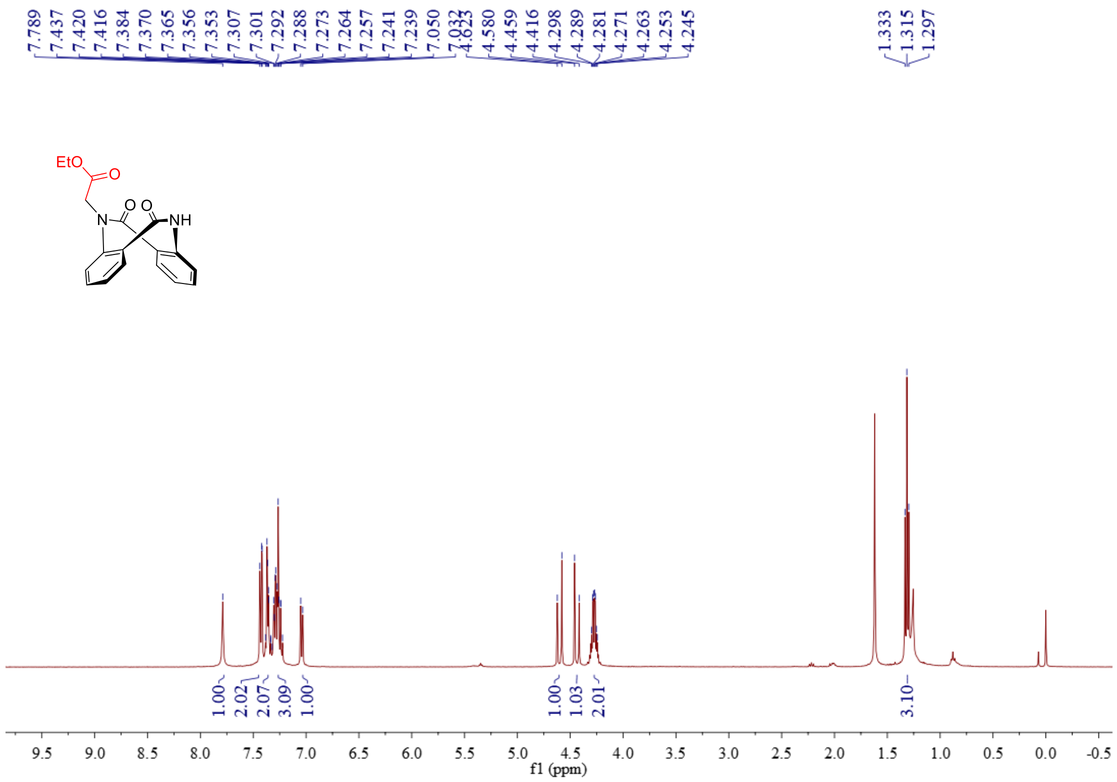

Supplementary Figure 130. ^{1}H NMR spectrum of compound **4o** ($CDCl_3$, 400 MHz)

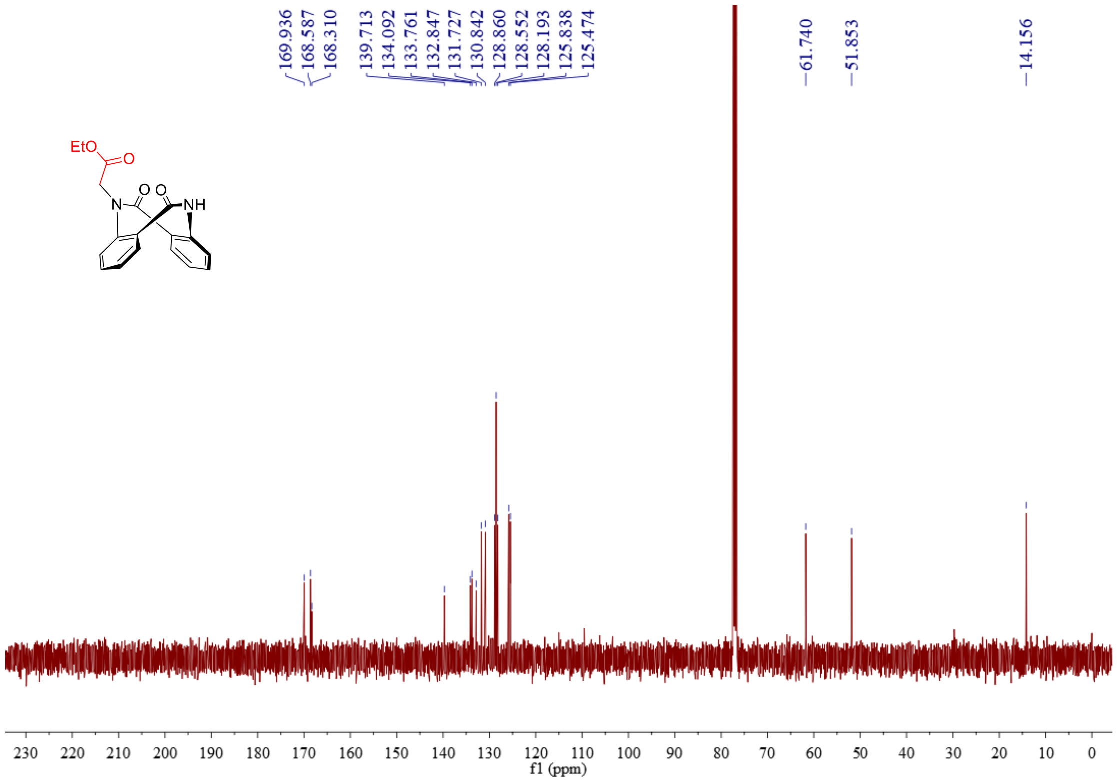

Supplementary Figure 131. ^{13}C NMR spectrum of compound **4o** ($CDCl_3$, 100 MHz)

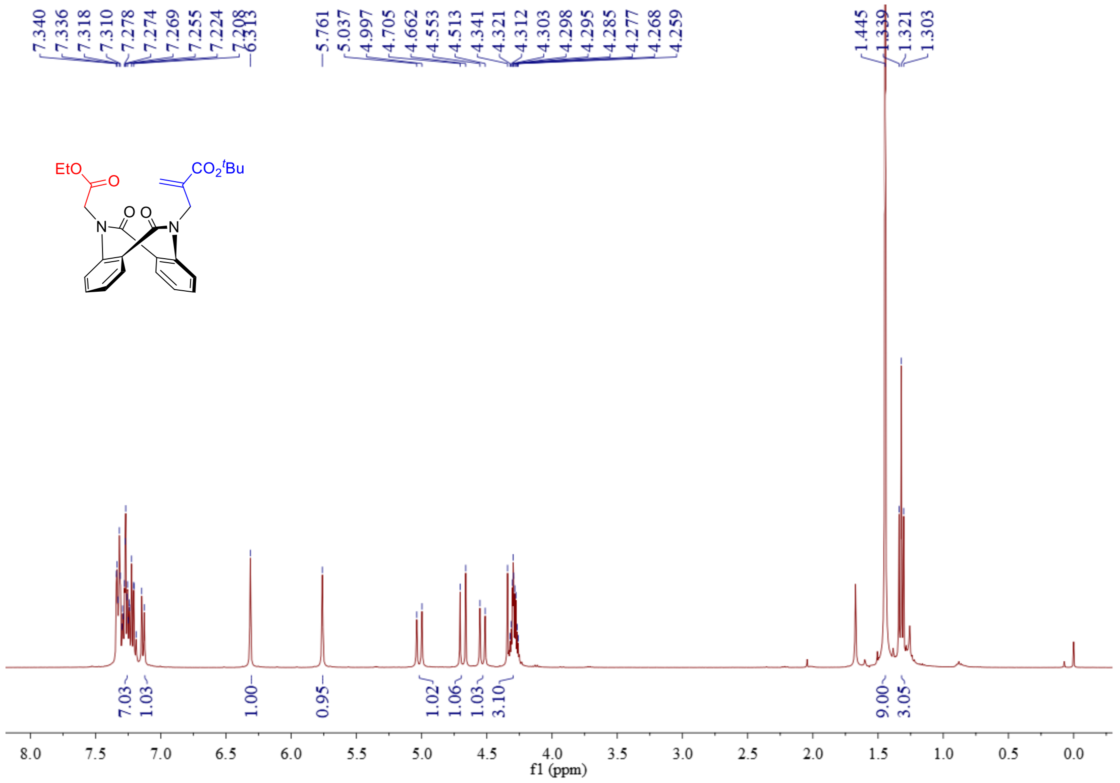

Supplementary Figure 132. ^{1}H NMR spectrum of compound **5o** ($CDCl_3$, 400 MHz)

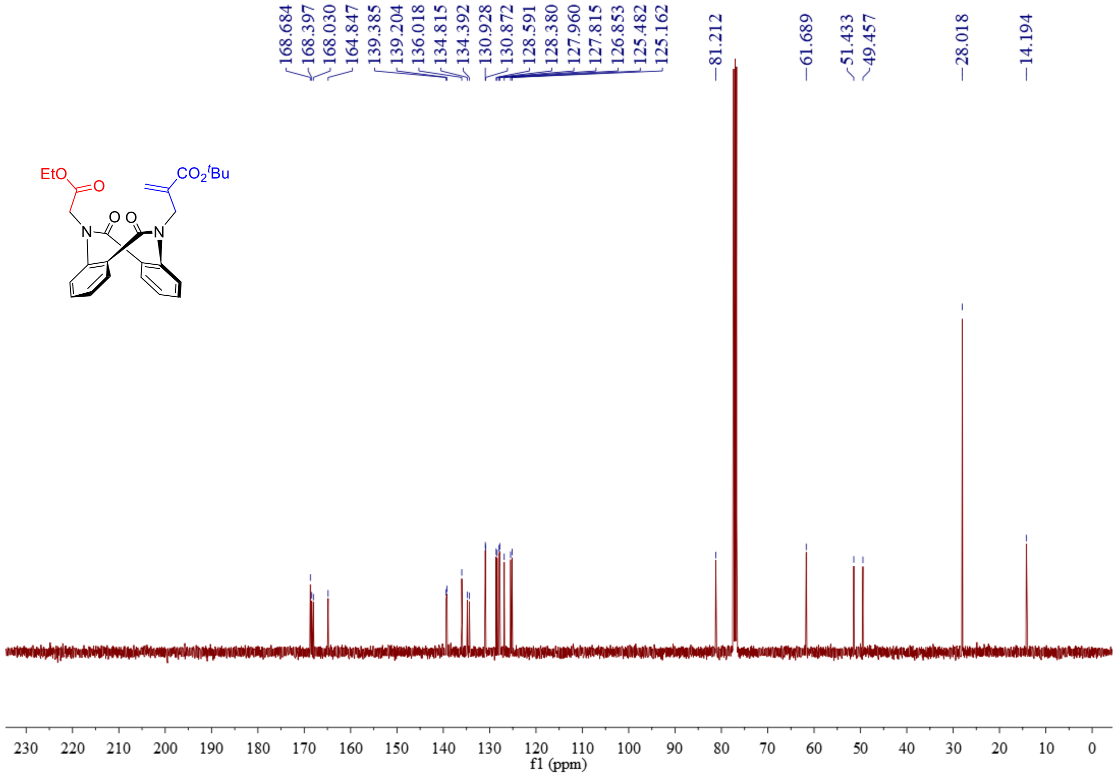

Supplementary Figure 133. ^{13}C NMR spectrum of compound **5o** ($CDCl_3$, 100 MHz)

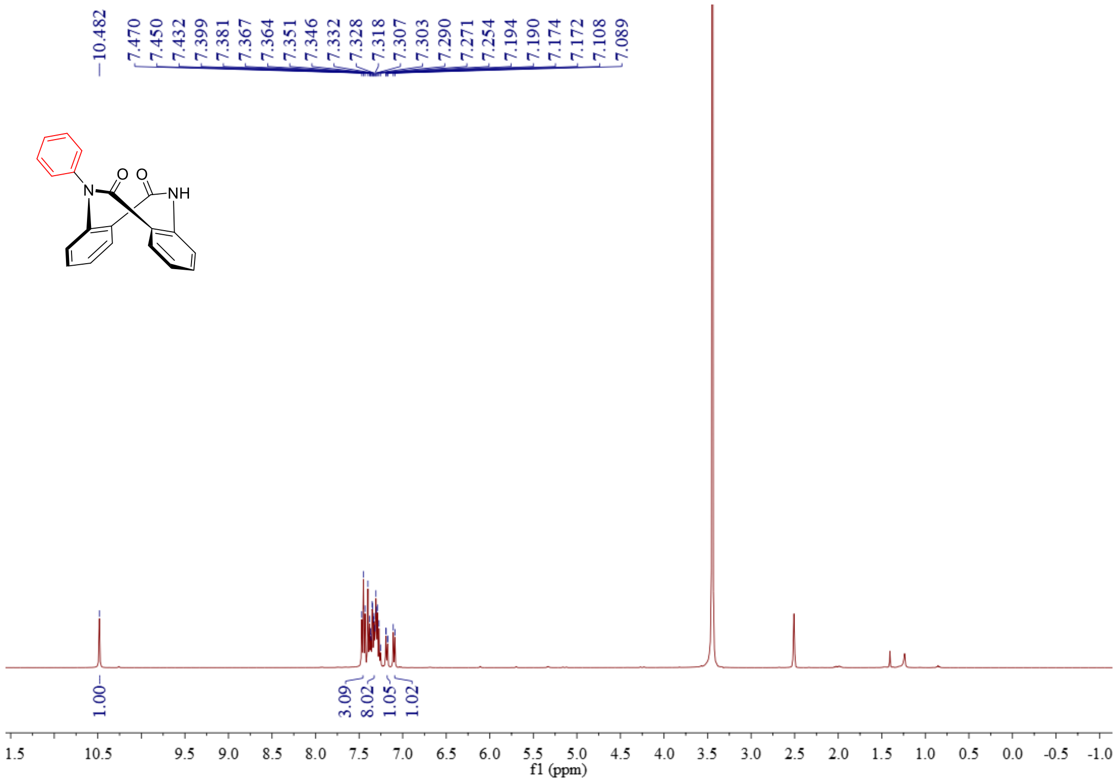

Supplementary Figure 134. ^{1}H NMR spectrum of compound **4p** (DMSO, 400 MHz)

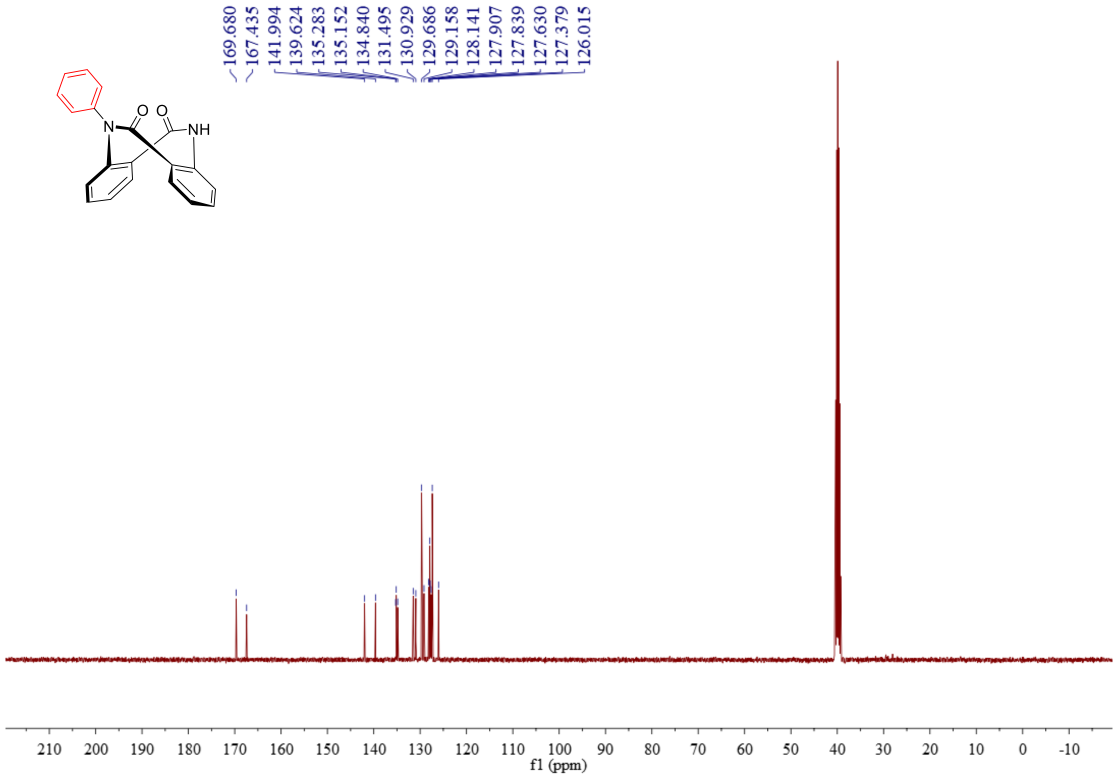

Supplementary Figure 135. ^{13}C NMR spectrum of compound **4p** (DMSO, 100 MHz)

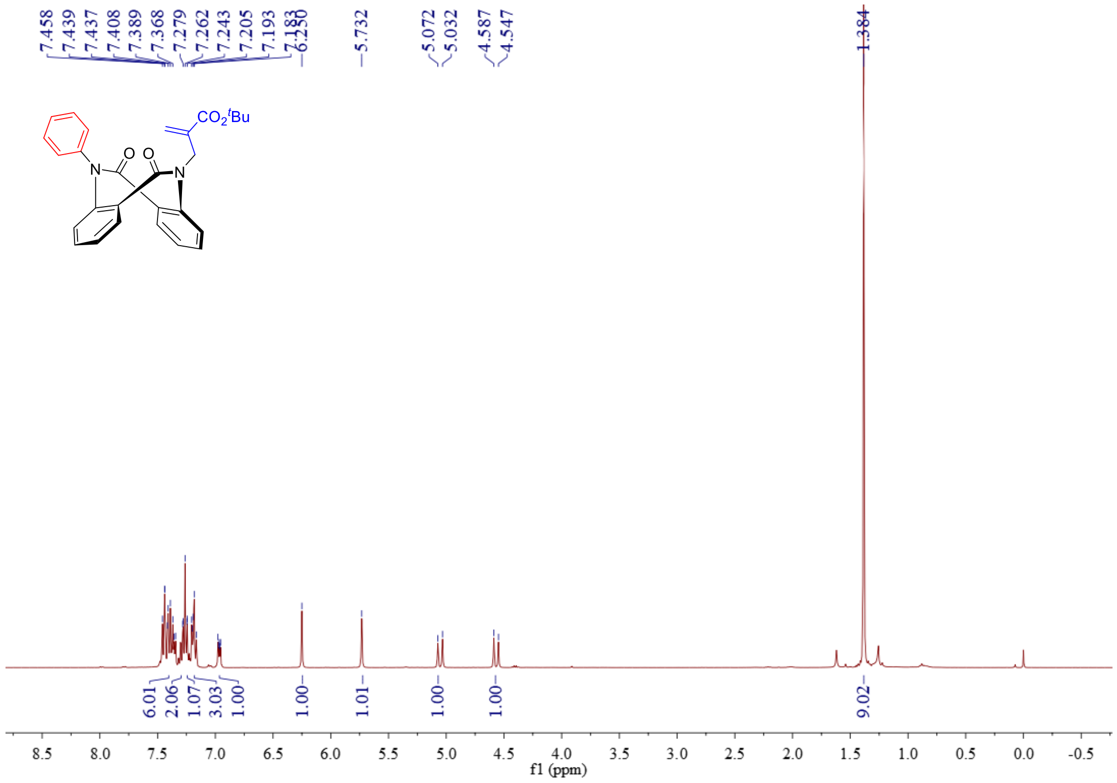

Supplementary Figure 136. ^{1}H NMR spectrum of compound **5p** ($CDCl_3$, 400 MHz)

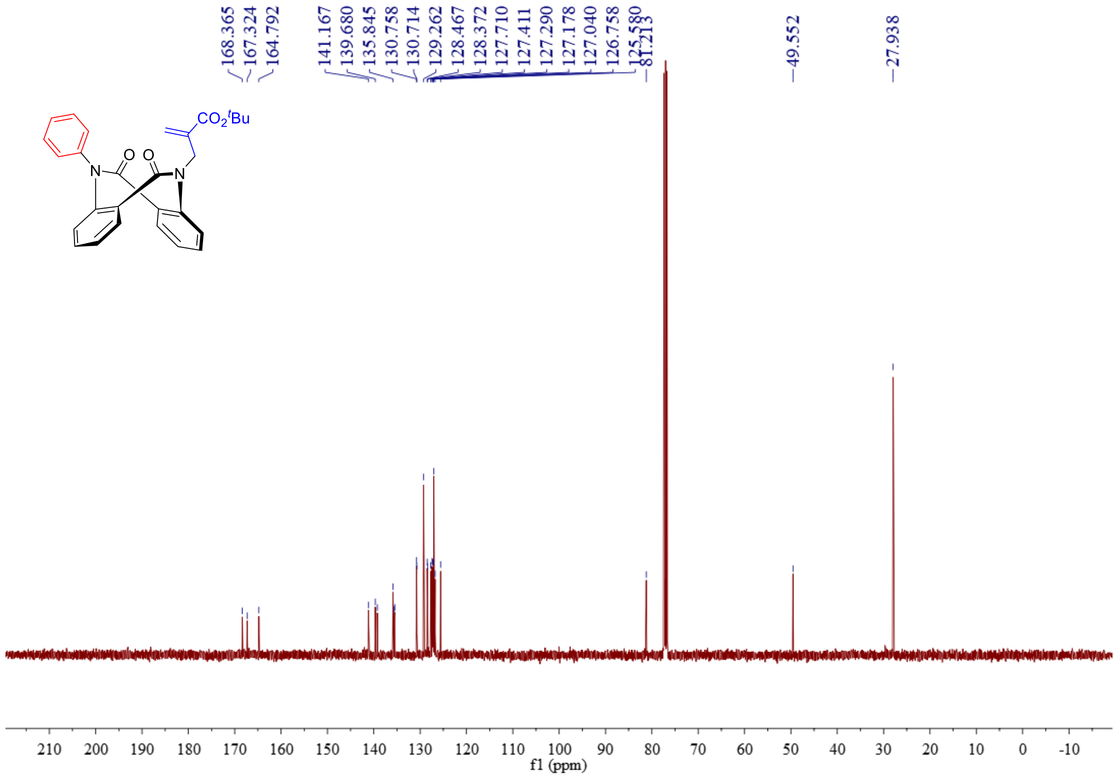

Supplementary Figure 137. ^{13}C NMR spectrum of compound **5p** ($CDCl_3$, 100 MHz)

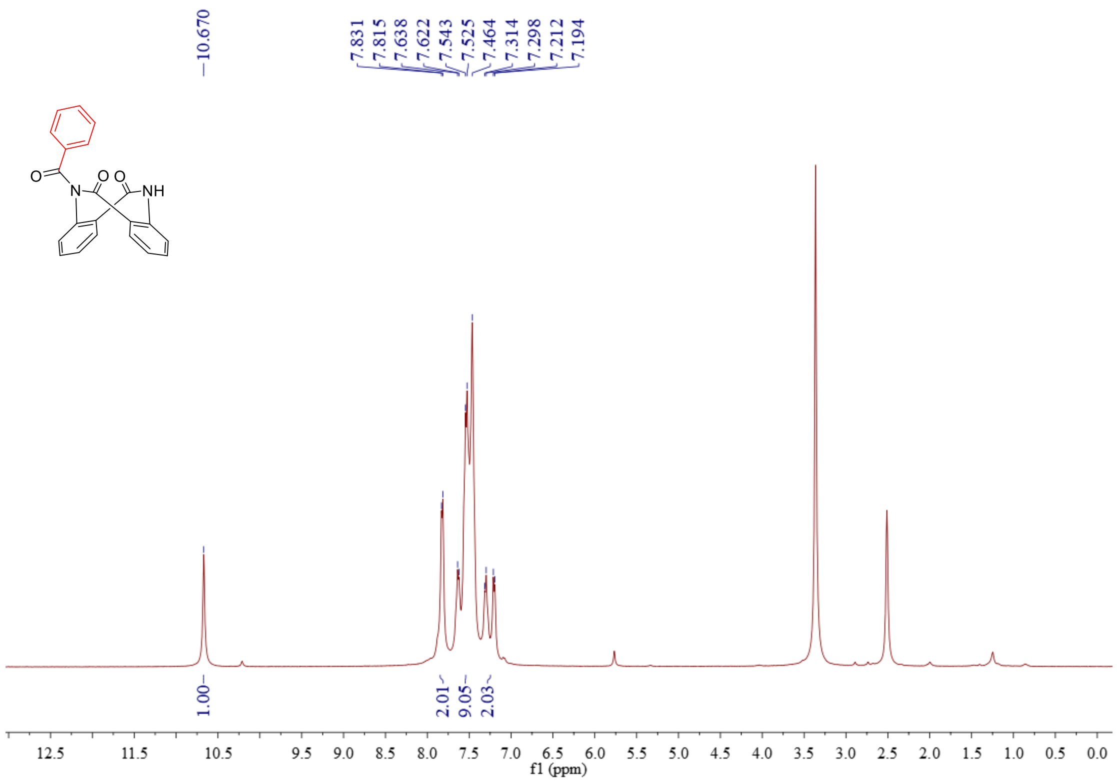

Supplementary Figure 138. ^{1}H NMR spectrum of compound **4q** (DMSO, 400 MHz)

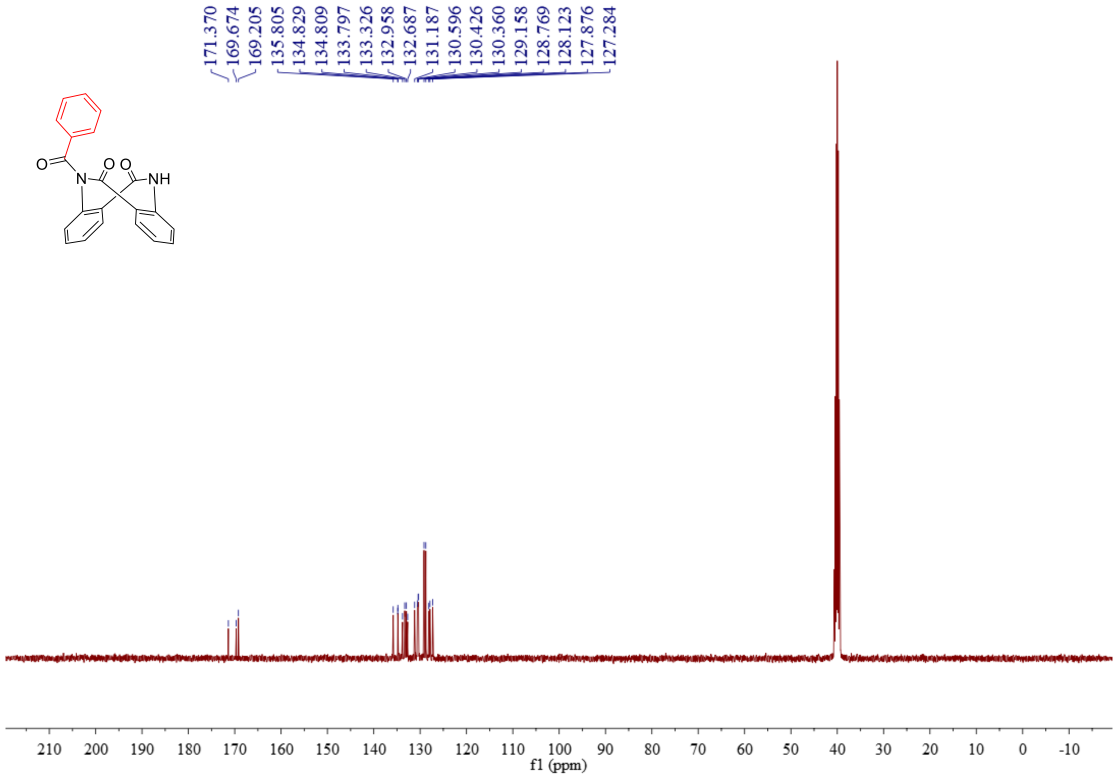

Supplementary Figure 139. ^{13}C NMR spectrum of compound **4q** (DMSO, 100 MHz)

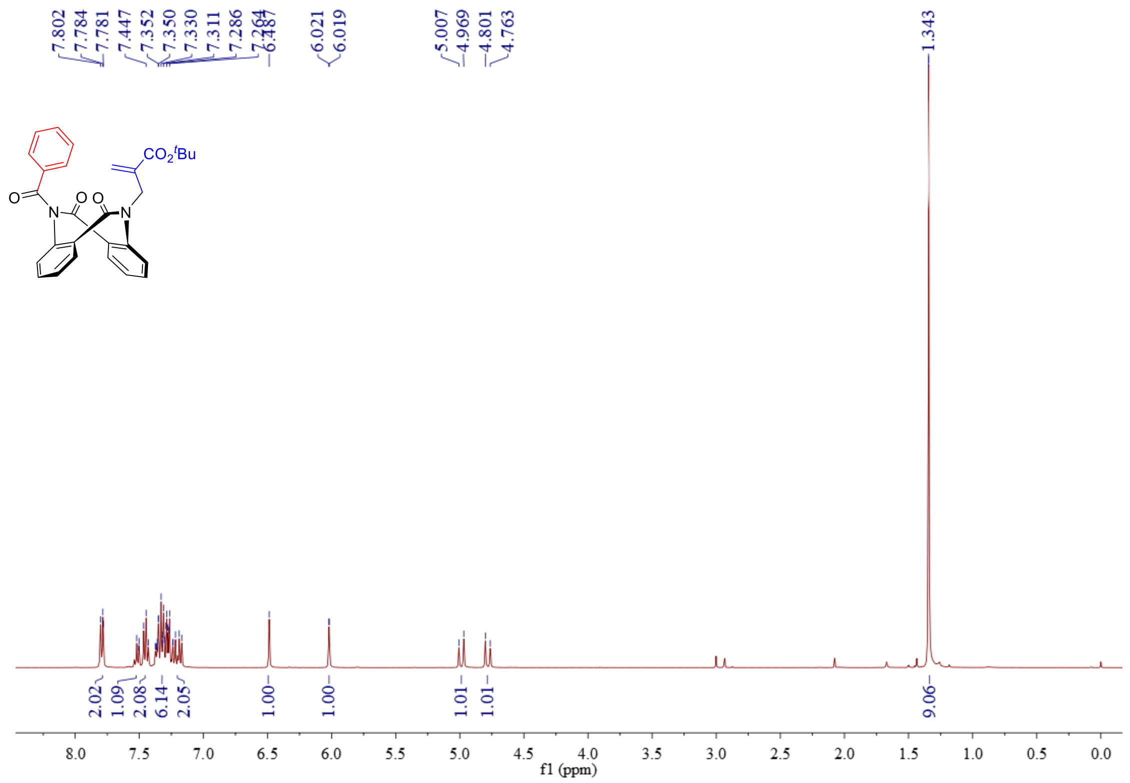

Supplementary Figure 140. ^{1}H NMR spectrum of compound **5q** ($CDCl_3$, 400 MHz)

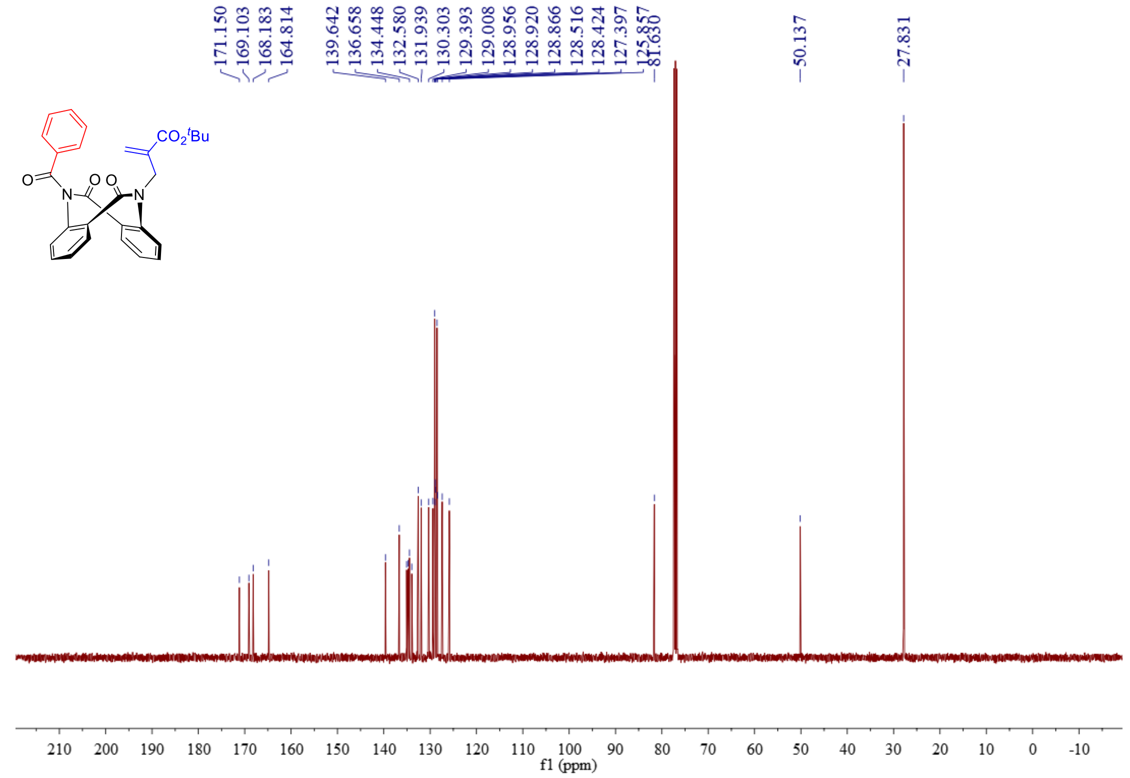

Supplementary Figure 141. ^{13}C NMR spectrum of compound **5q** ($CDCl_3$, 100 MHz)

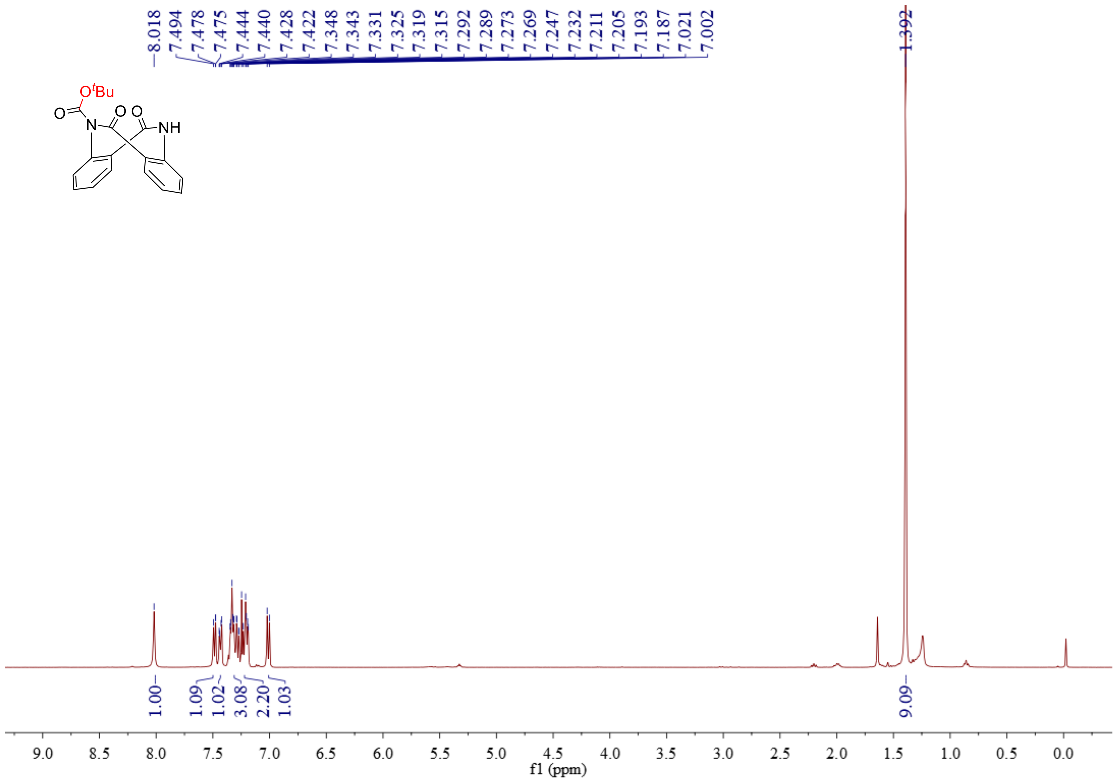

Supplementary Figure 142. ^{1}H NMR spectrum of compound **4r** ($CDCl_3$, 400 MHz)

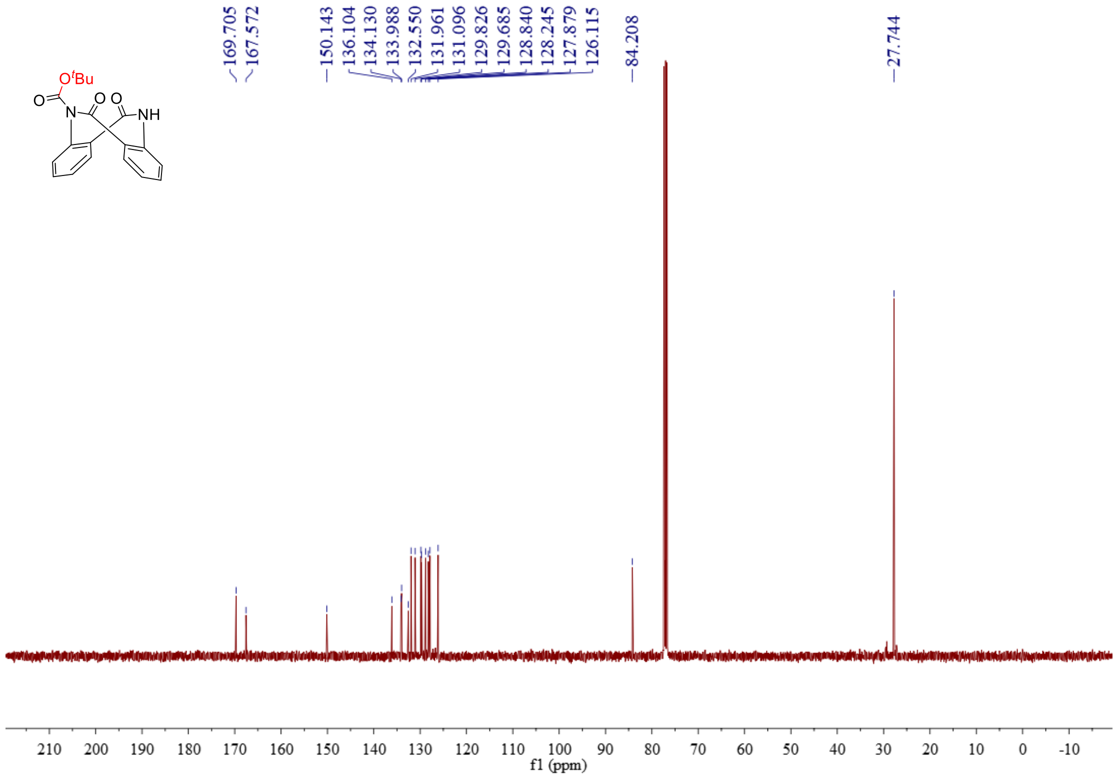

Supplementary Figure 143. ^{13}C NMR spectrum of compound **4r** ($CDCl_3$, 100 MHz)

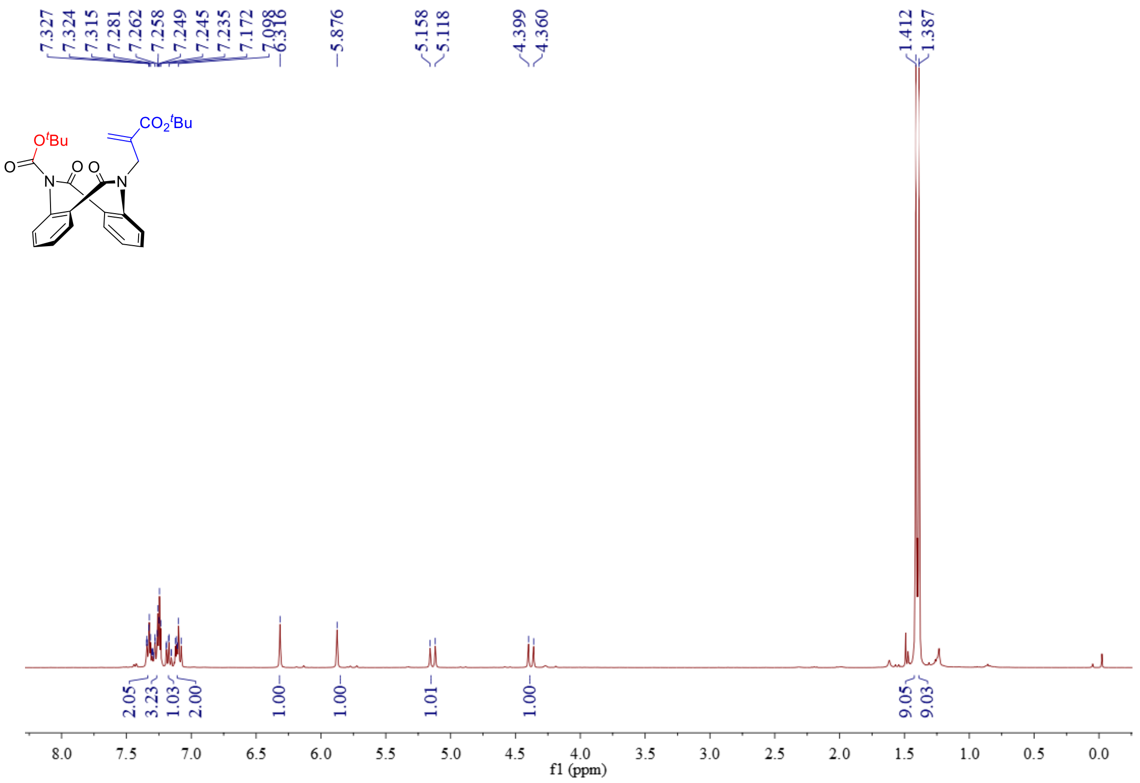

Supplementary Figure 144. ^{1}H NMR spectrum of compound **5r** ($CDCl_3$, 400 MHz)

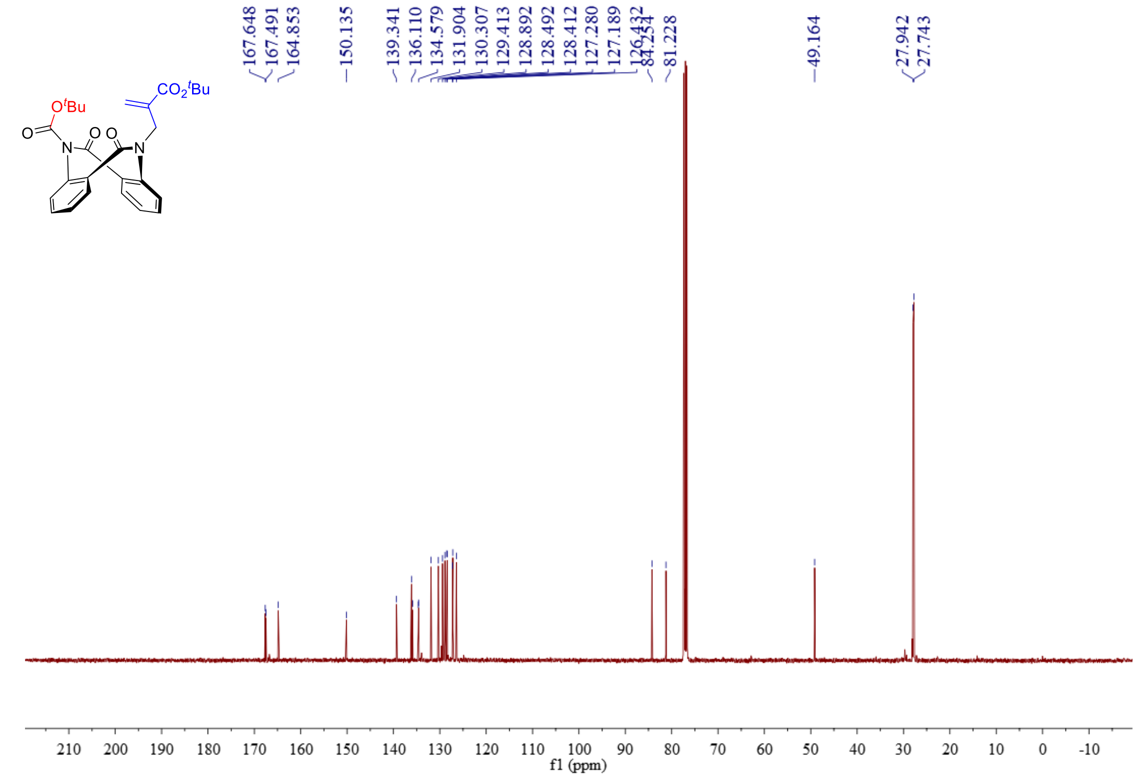

Supplementary Figure 145. ^{13}C NMR spectrum of compound **5r** ($CDCl_3$, 100 MHz)

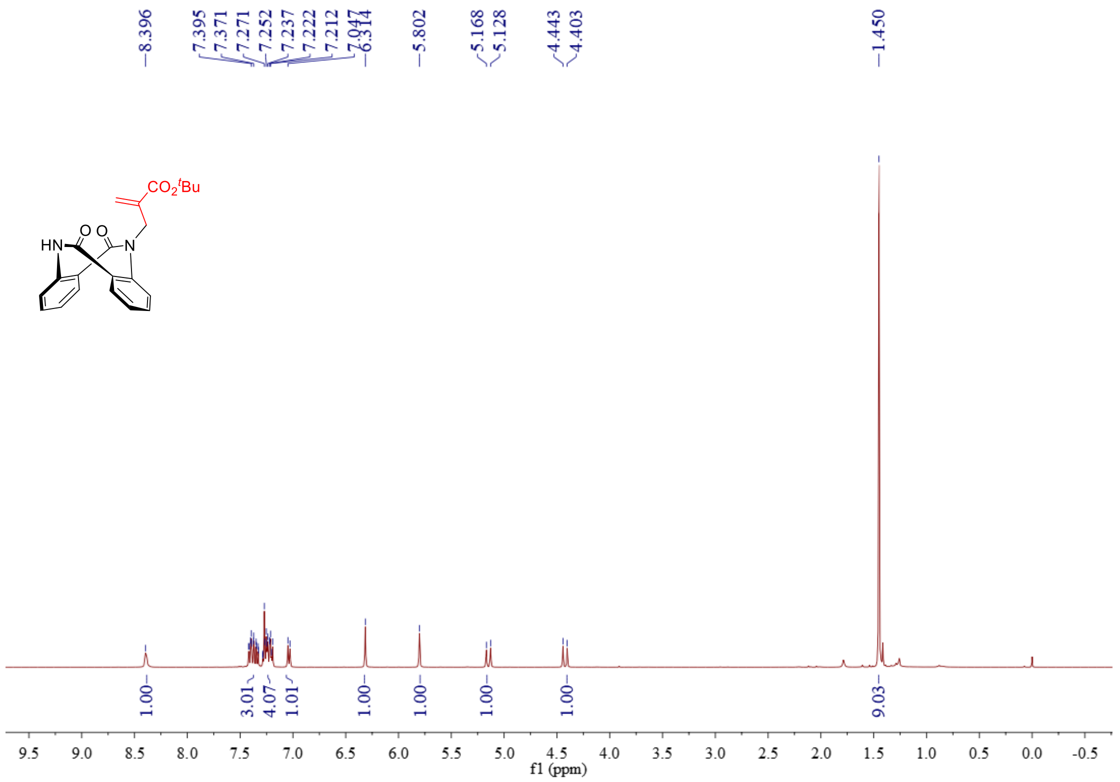

Supplementary Figure 146. ^{1}H NMR spectrum of compound **1a′** ($CDCl_3$, 400 MHz)

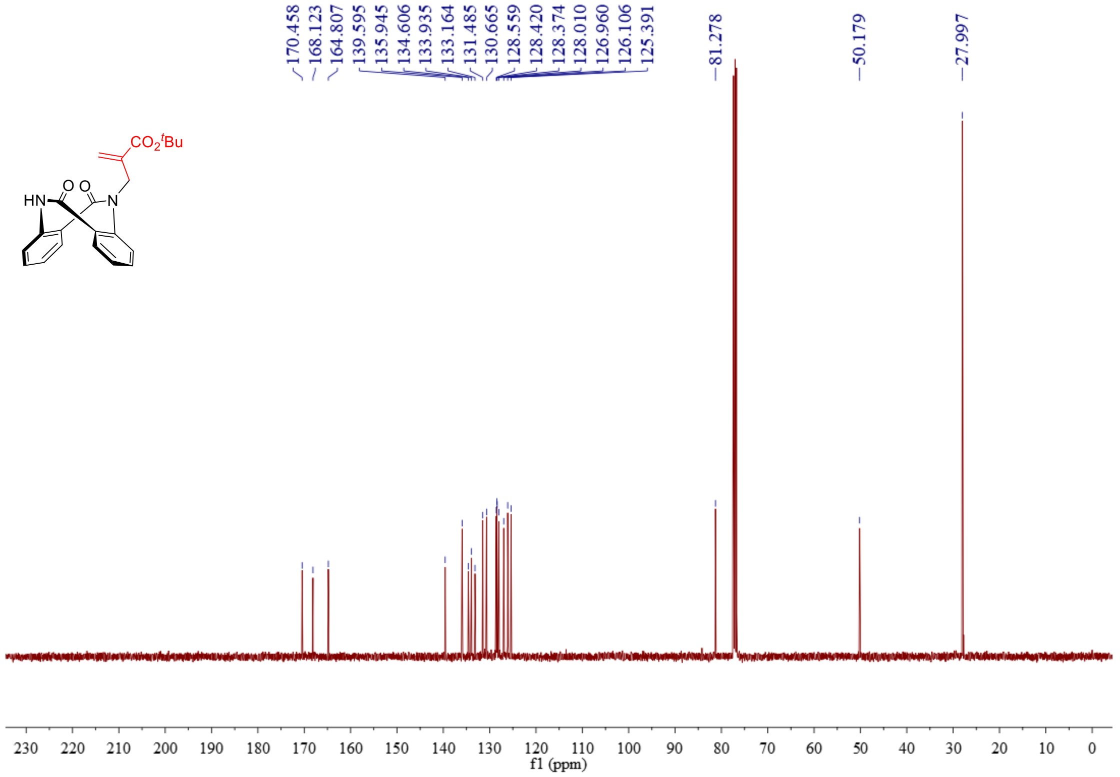

Supplementary Figure 147. ^{13}C NMR spectrum of compound **1a′** ($CDCl_3$, 100 MHz)

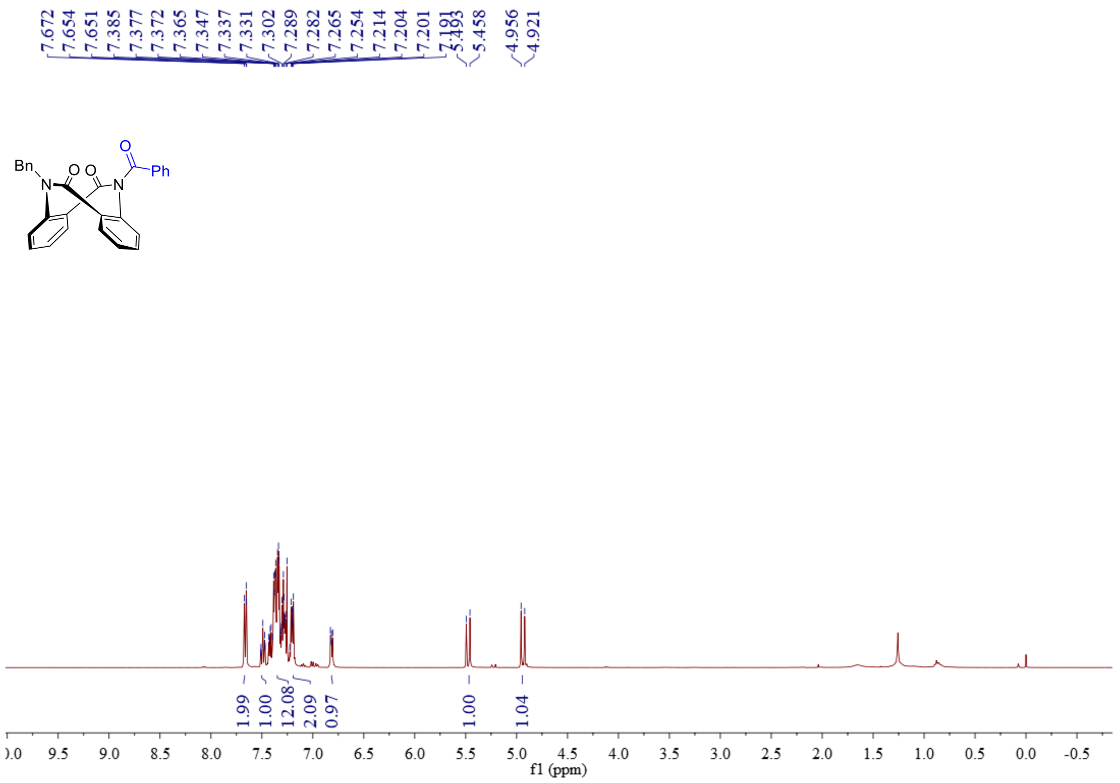

Supplementary Figure 148. ^{1}H NMR spectrum of compound **6a** ($CDCl_3$, 400 MHz)

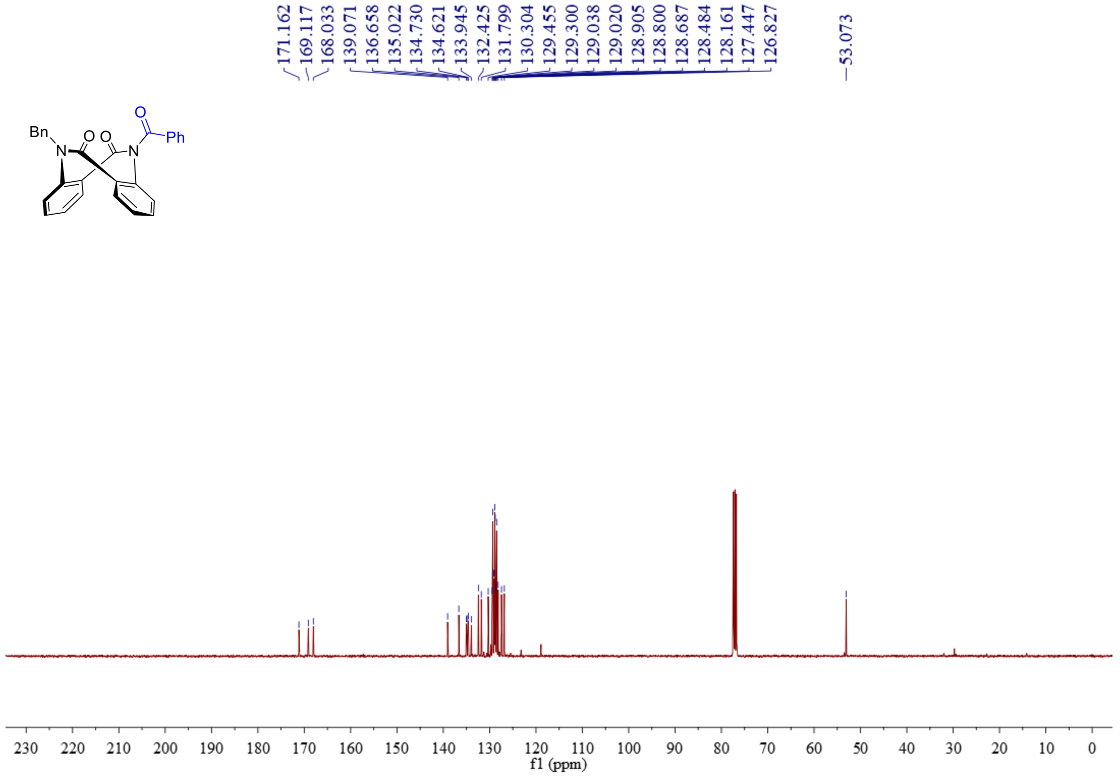

Supplementary Figure 149. ^{13}C NMR spectrum of compound **6a** ($CDCl_3$, 100 MHz)

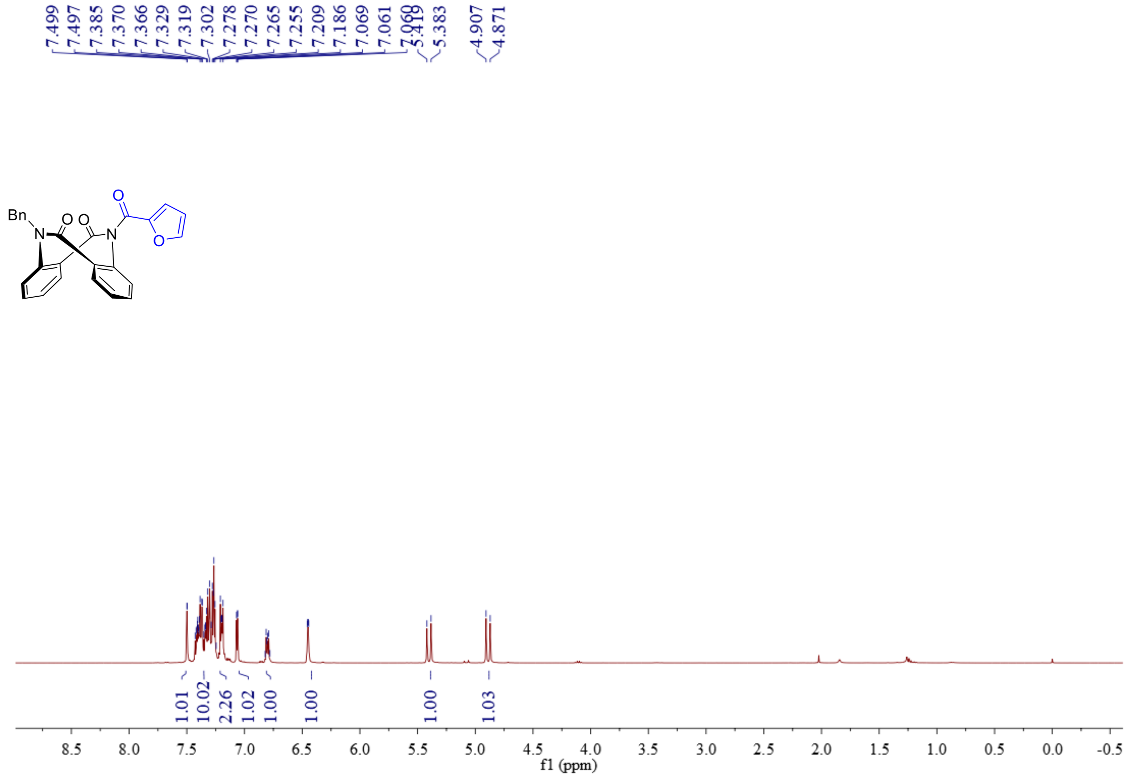

Supplementary Figure 150. ^{1}H NMR spectrum of compound **6b** ($CDCl_3$, 400 MHz)

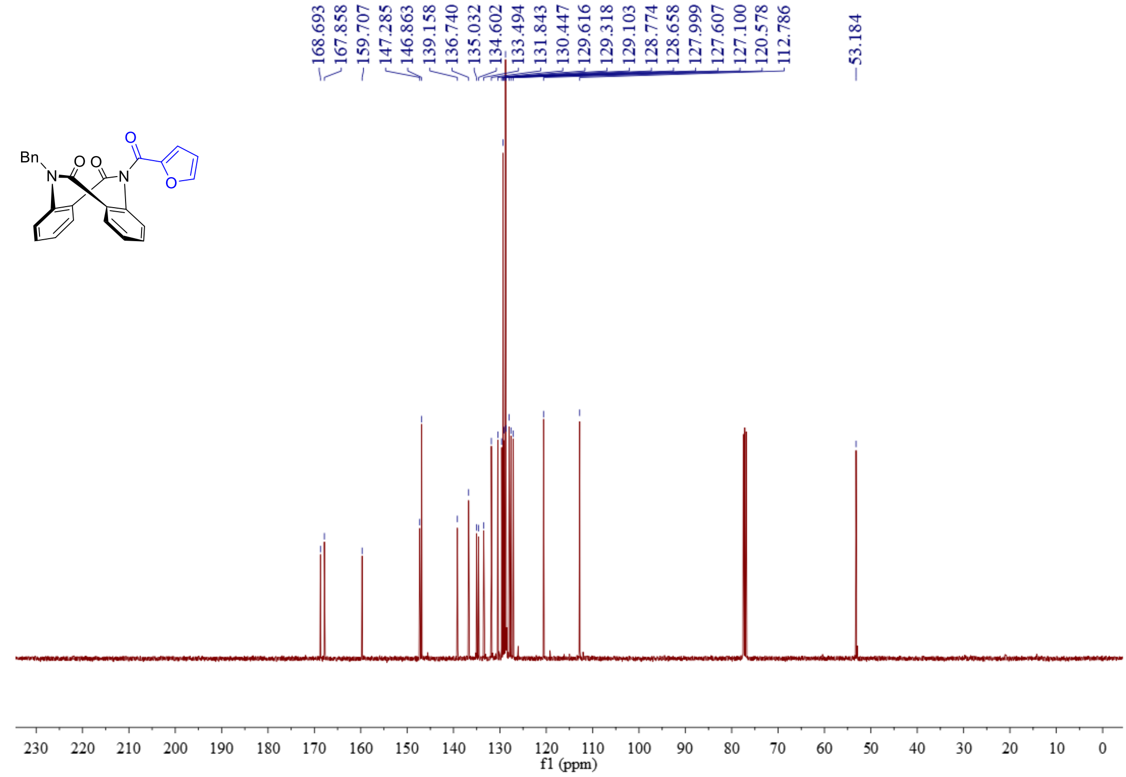

Supplementary Figure 151. ^{13}C NMR spectrum of compound **6b** ($CDCl_3$, 100 MHz)

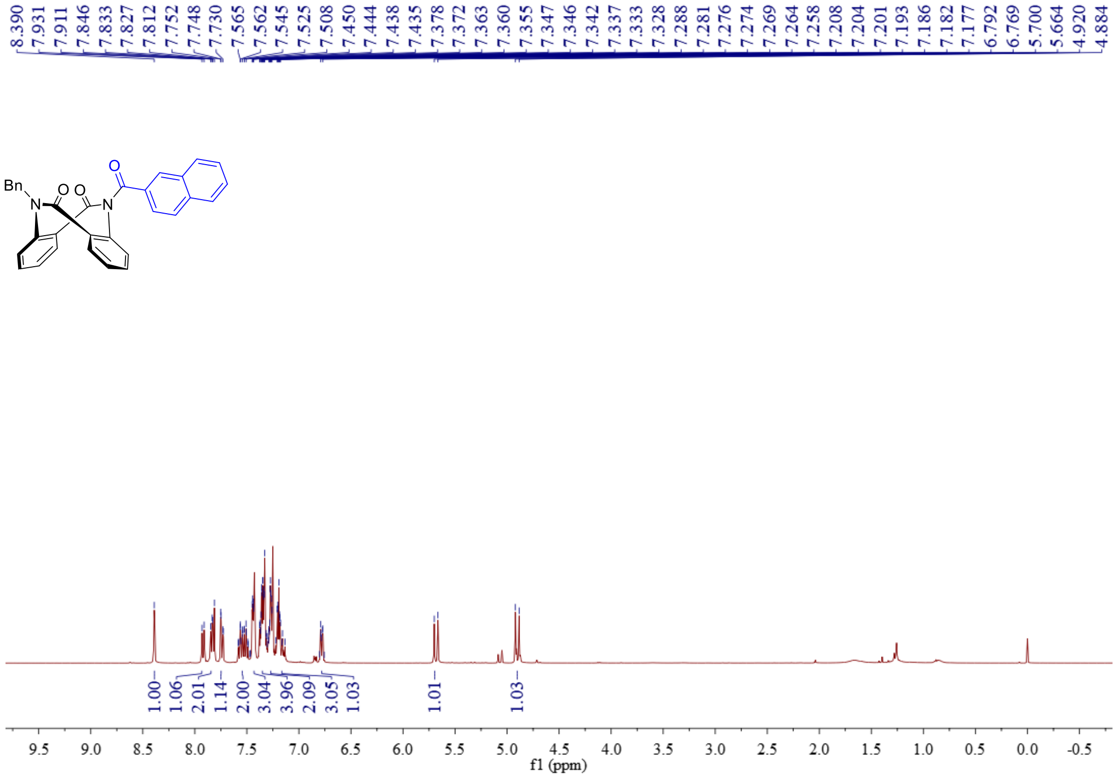

Supplementary Figure 152. ^{1}H NMR spectrum of compound **6c** ($CDCl_3$, 400 MHz)

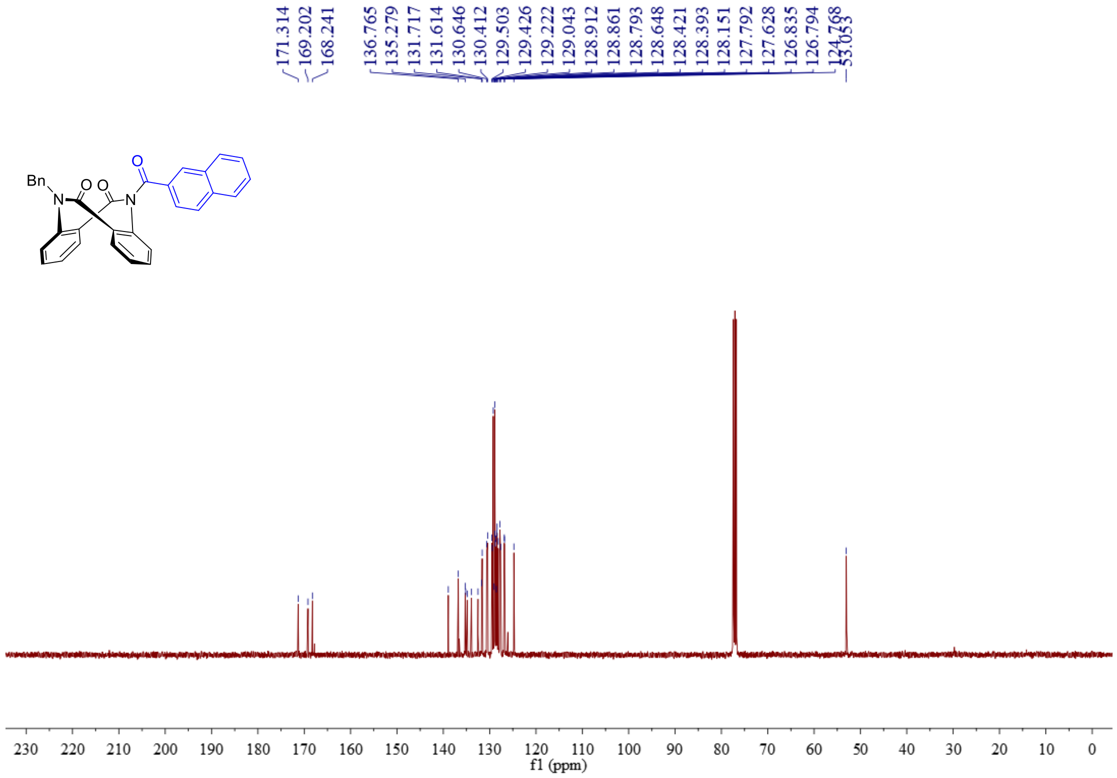

Supplementary Figure 153. ^{13}C NMR spectrum of compound **6c** ($CDCl_3$, 100 MHz)

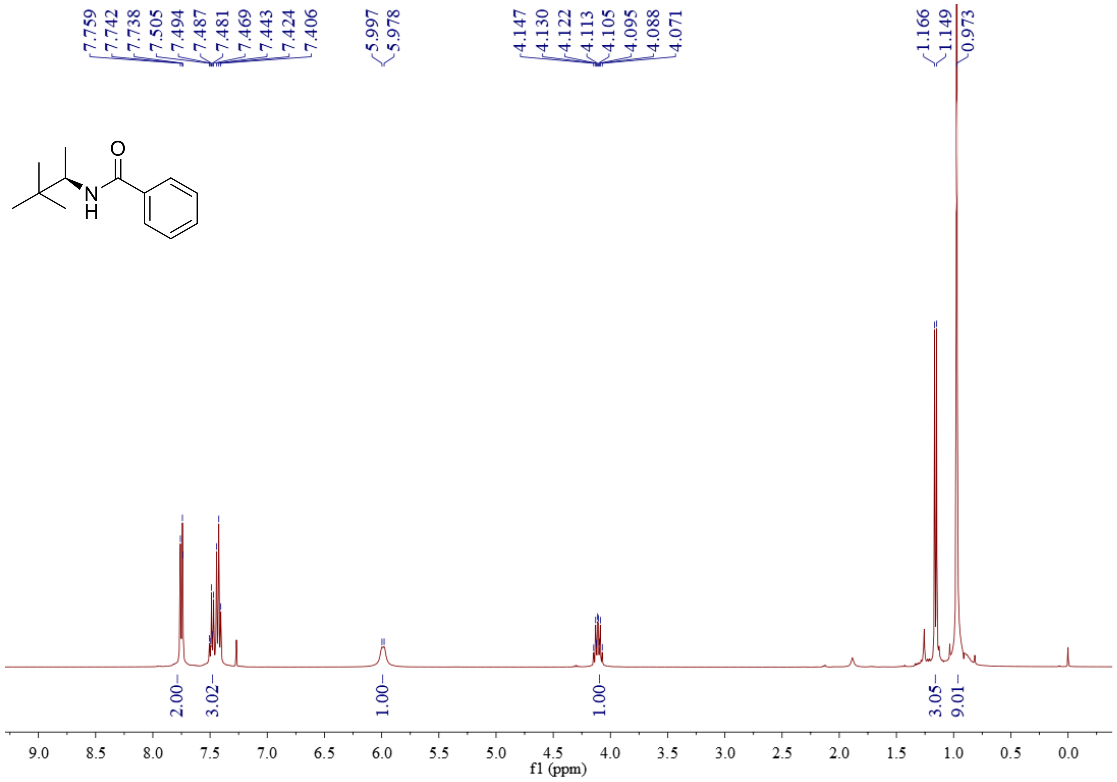

Supplementary Figure 154. ^{1}H NMR spectrum of compound **8a** ($CDCl_3$, 400 MHz)

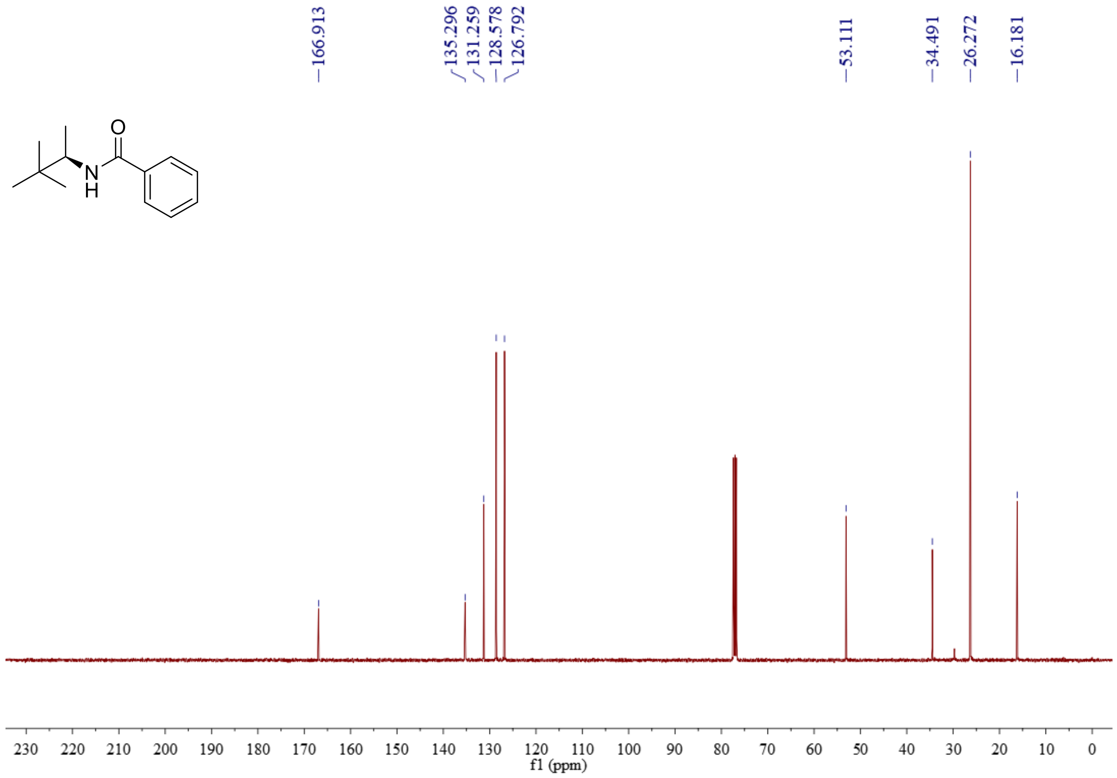

Supplementary Figure 155. ^{13}C NMR spectrum of compound **8a** ($CDCl_3$, 100 MHz)

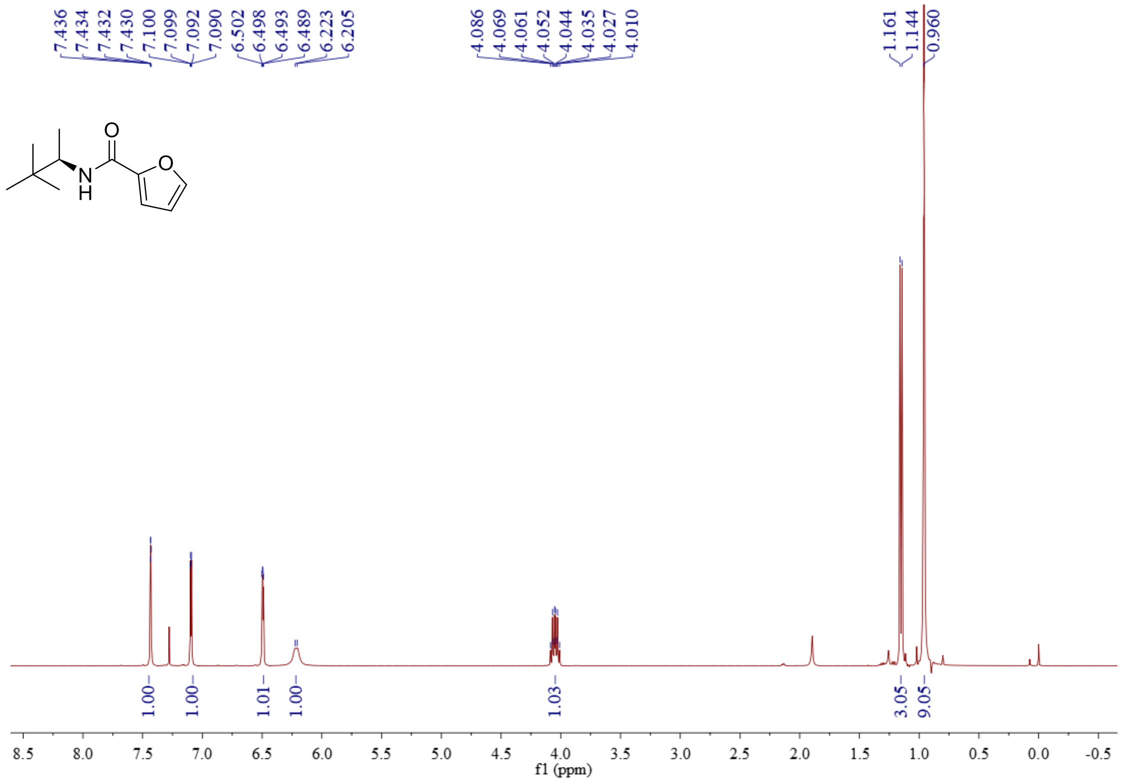

Supplementary Figure 156. ^{1}H NMR spectrum of compound **8b** ($CDCl_3$, 400 MHz)

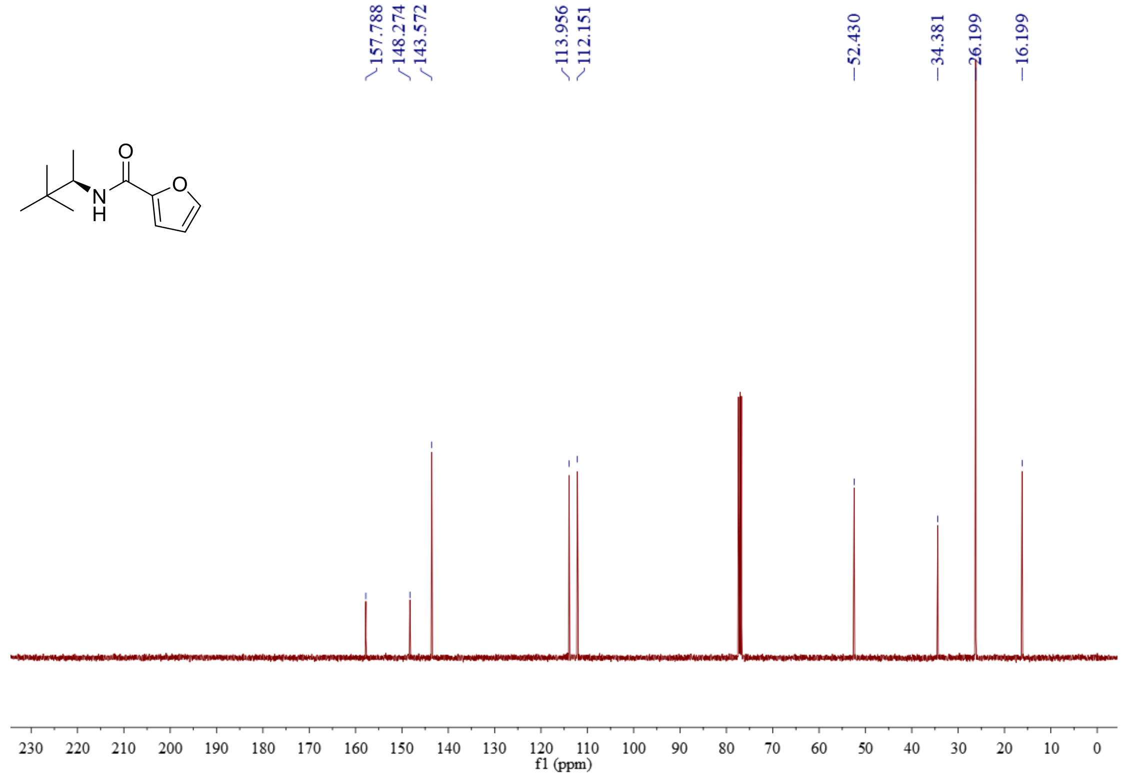

Supplementary Figure 157. ^{13}C NMR spectrum of compound **8b** ($CDCl_3$, 100 MHz)

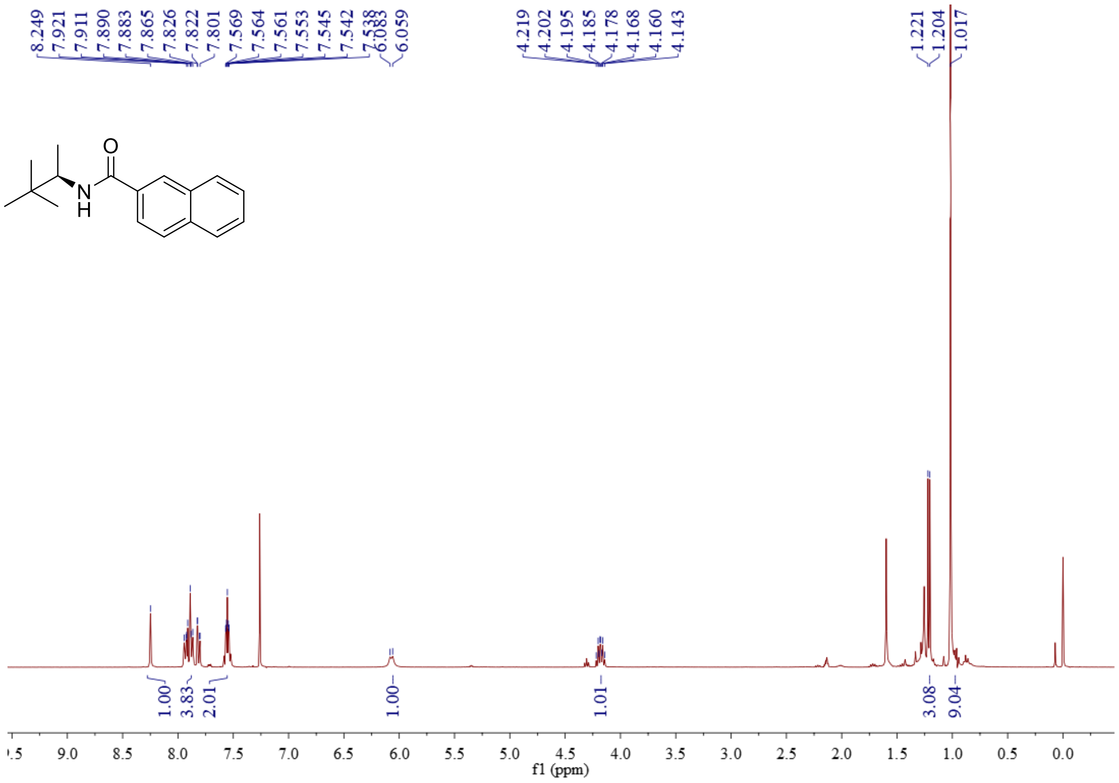

Supplementary Figure 158. ^{1}H NMR spectrum of compound **8c** ($CDCl_3$, 400 MHz)

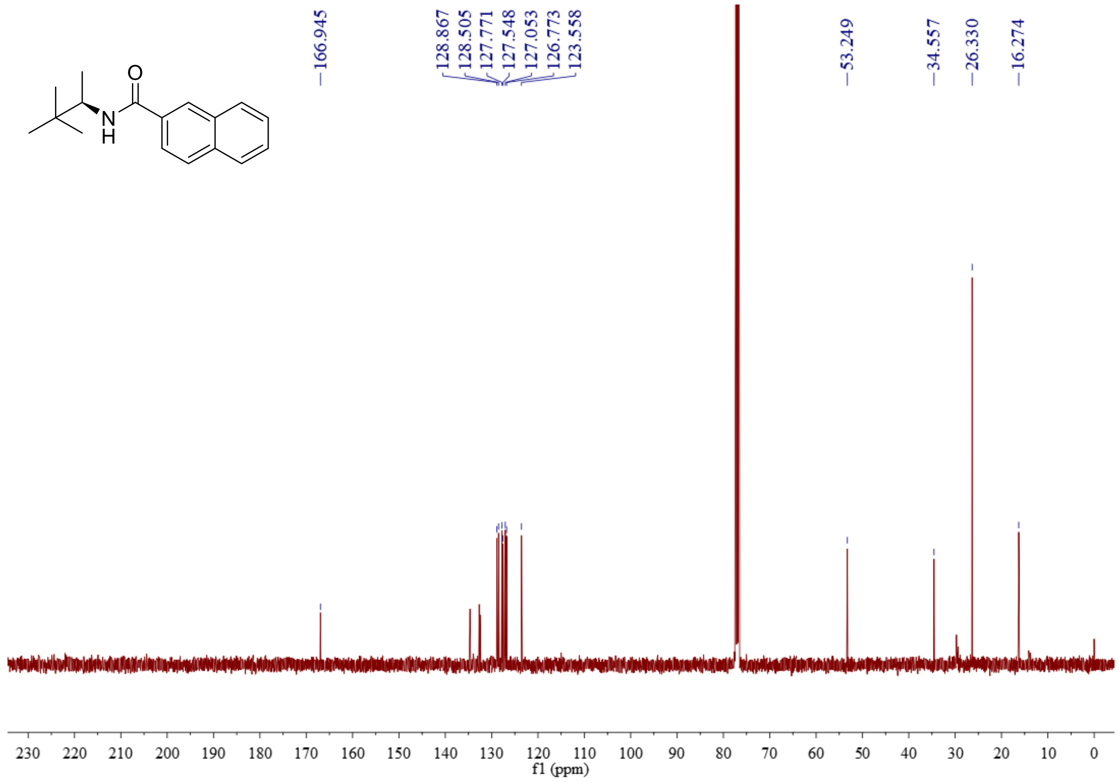

Supplementary Figure 159. ^{13}C NMR spectrum of compound **8c** ($CDCl_3$, 100 MHz)

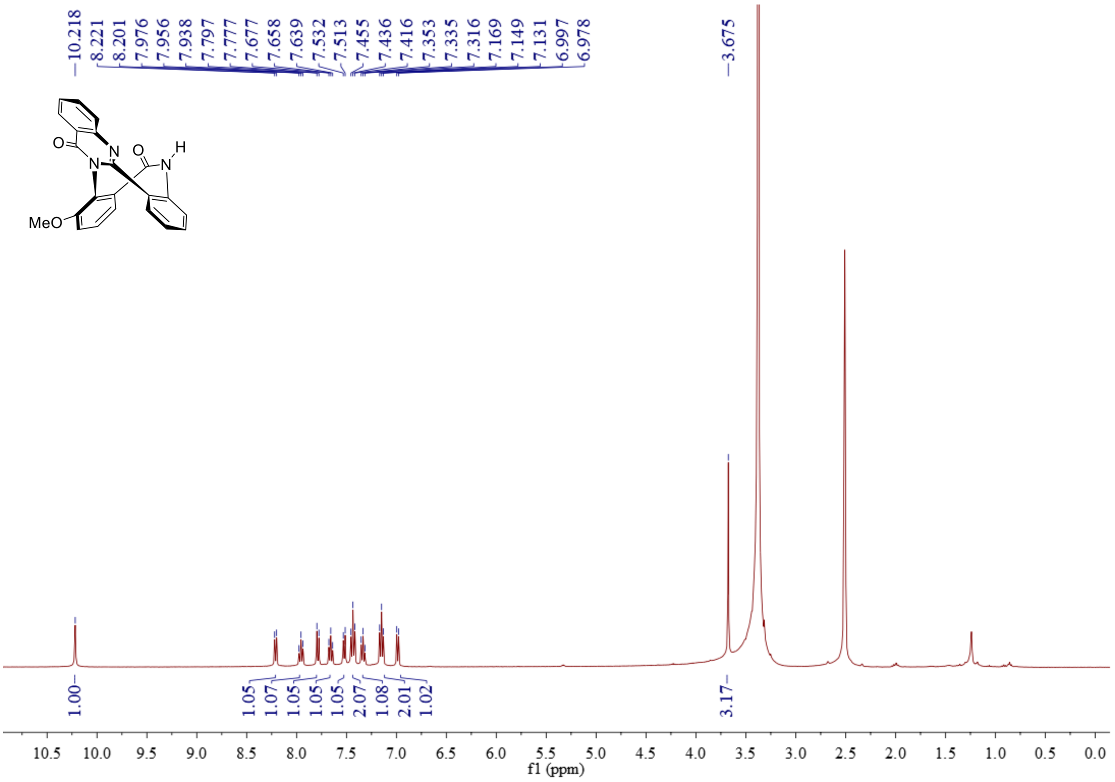

Supplementary Figure 160. ^{1}H NMR spectrum of compound **10** ($(CD_3)_2SO$, 400 MHz)

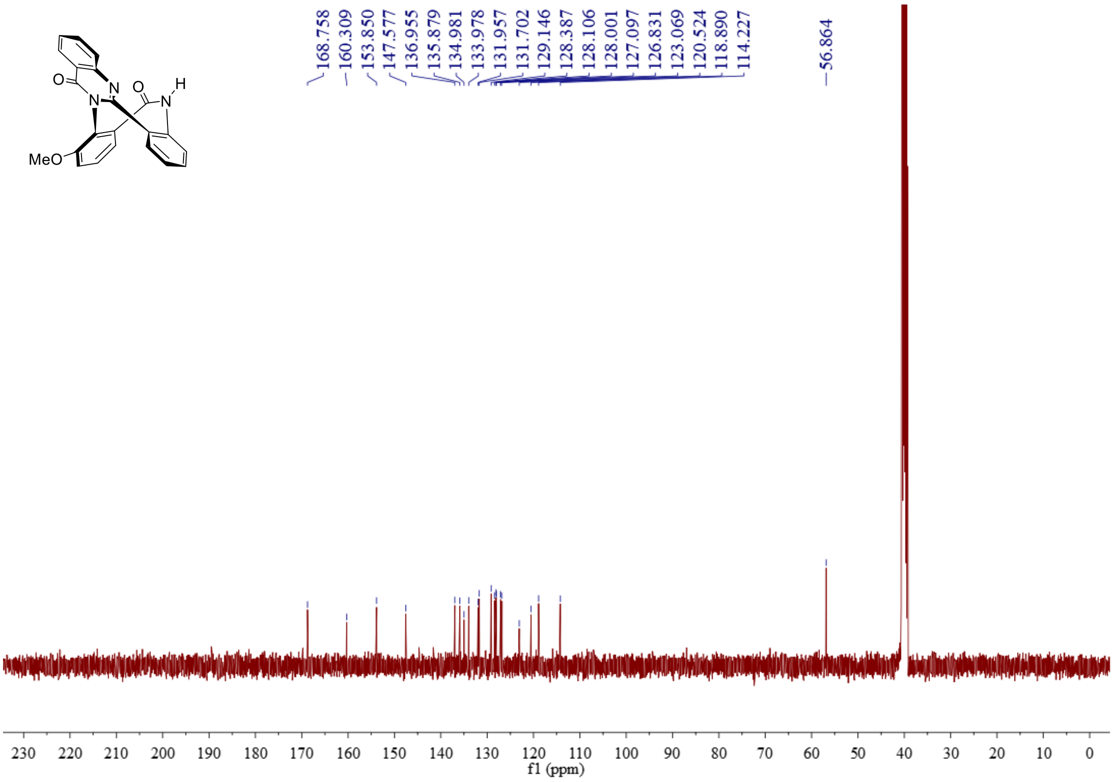

Supplementary Figure 161. ^{13}C NMR spectrum of compound **10** ($(CD_3)_2SO$, 100 MHz)

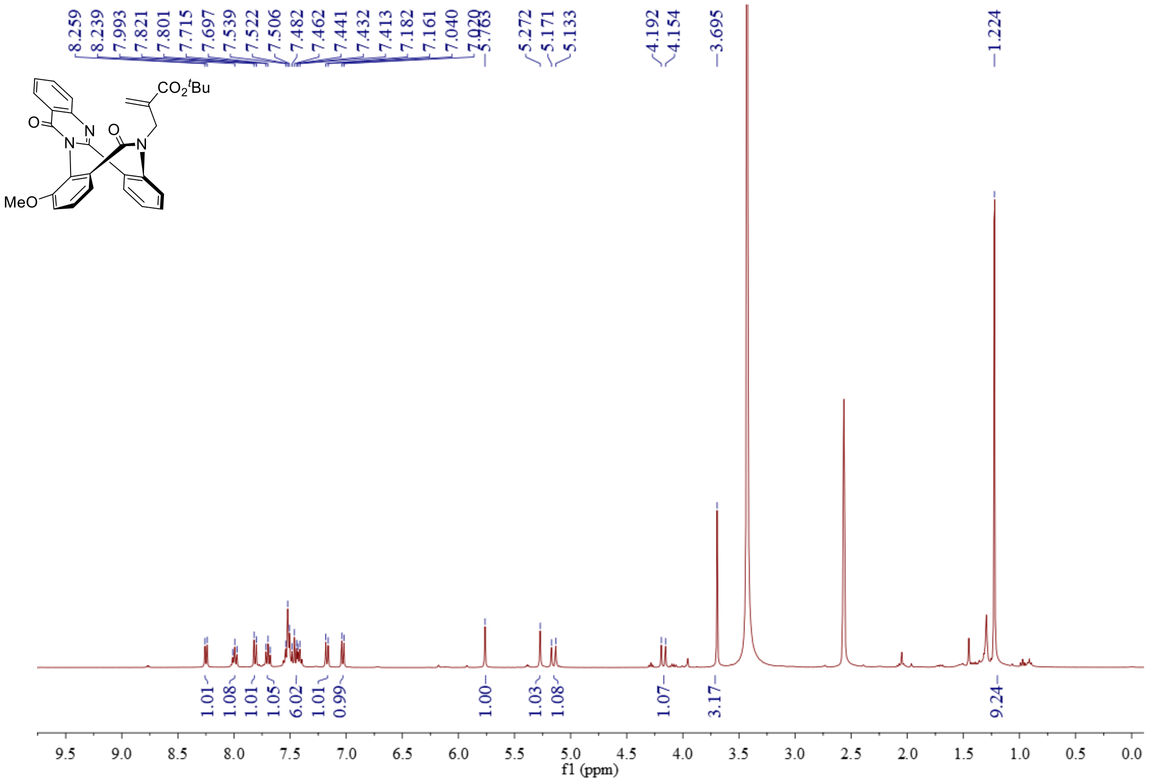

Supplementary Figure 162. ^{1}H NMR spectrum of compound **11** ($(CD_3)_2SO$, 400 MHz)

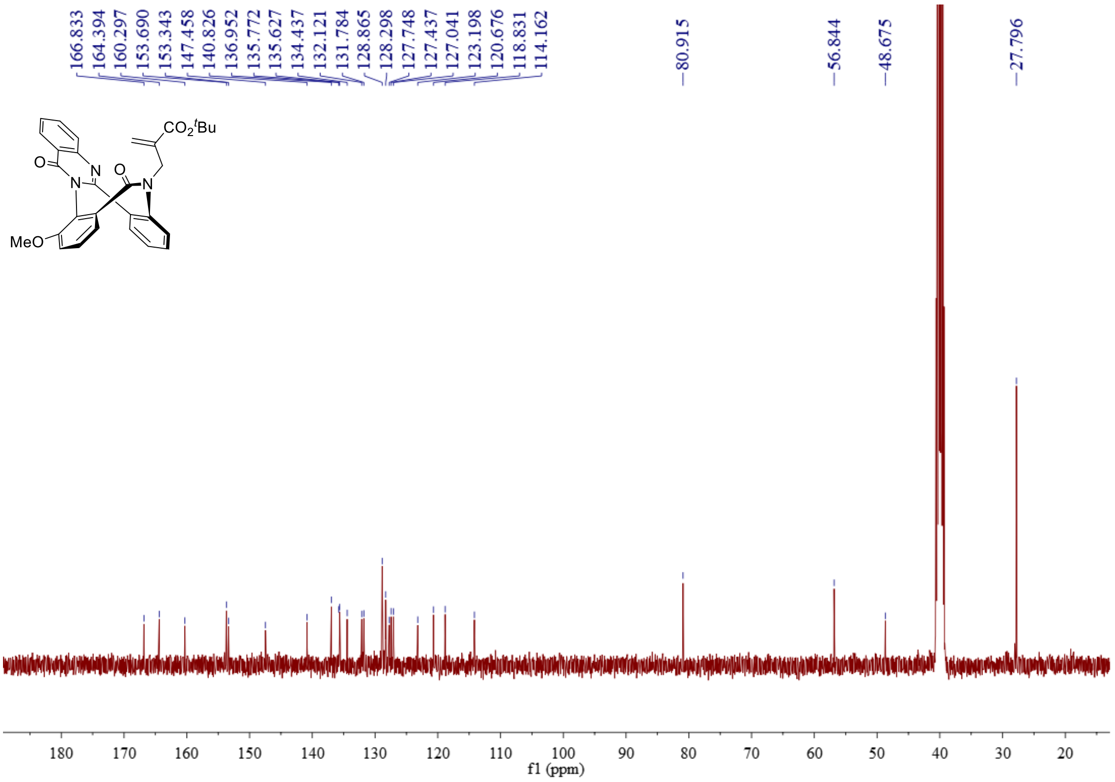

Supplementary Figure 163. ^{13}C NMR spectrum of compound **11** ($(CD_3)_2SO$, 100 MHz)

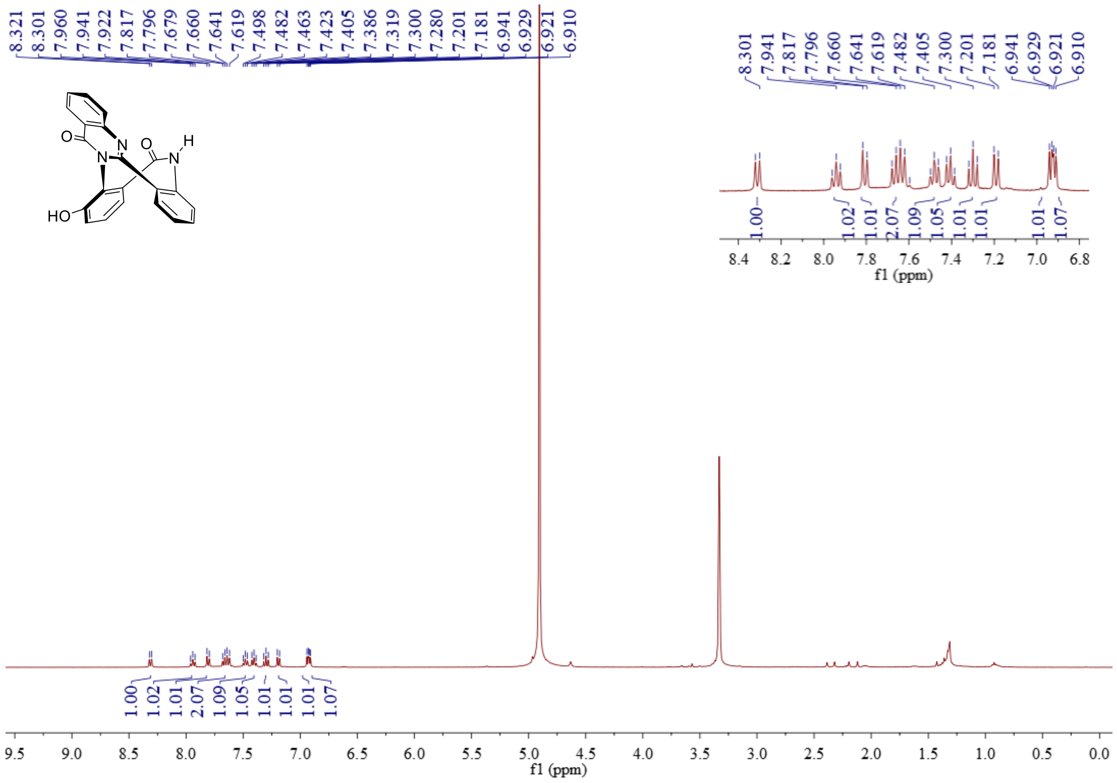

Supplementary Figure 164. ^{1}H NMR spectrum of compound **12** ((CD_3OD), 400 MHz)

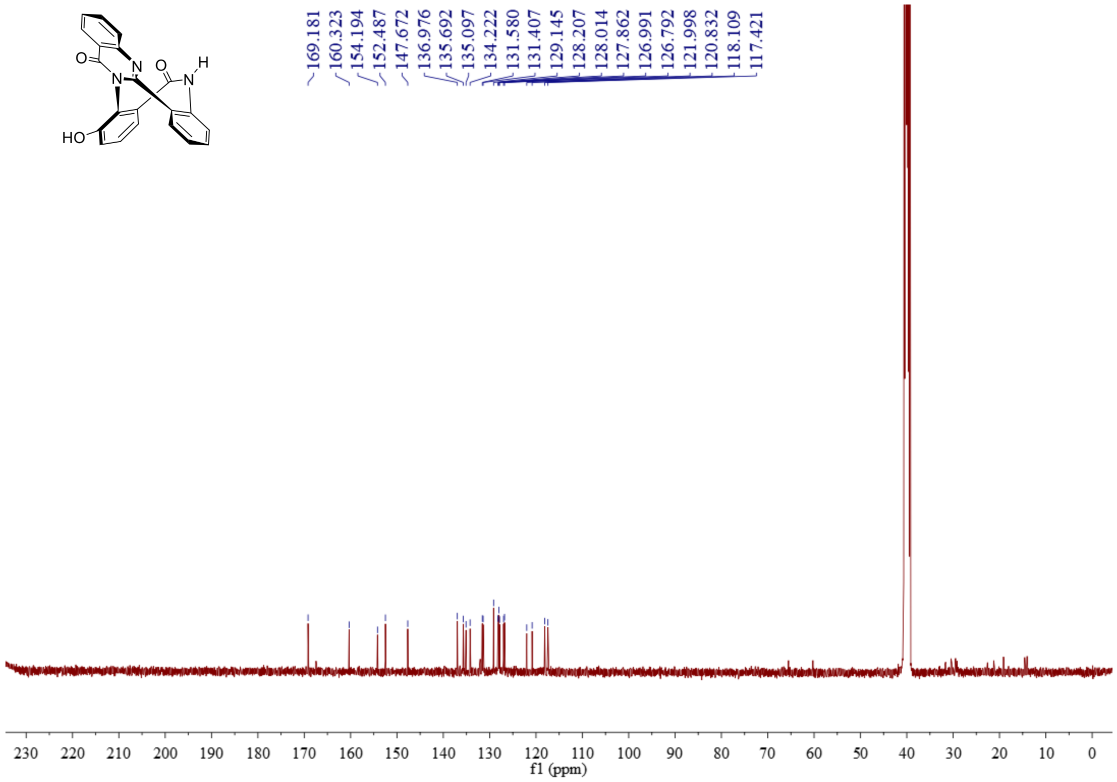

Supplementary Figure 165. ^{13}C NMR spectrum of compound **12** ($(CD_3)_2SO$, 100 MHz)

10. HPLC spectras

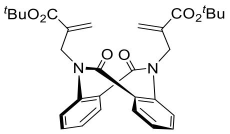

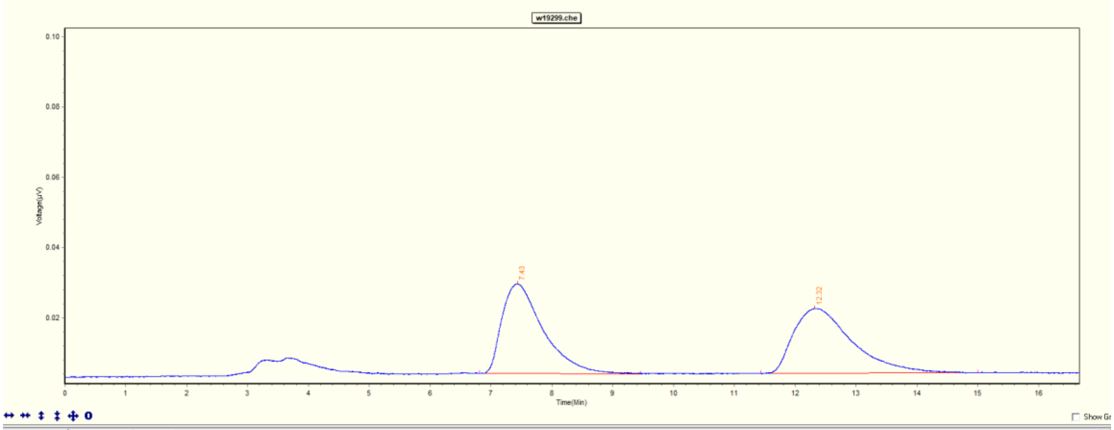

No.	Retention Time	Peak Area	Peak Height	Peak Area(%)	Peak Width	Peak Type
1	7.43	596007	12728	49.22%	2.646	BB
2	12.32	614892	9224	50.78%	3.564	BB
Total		1,210,899	21,952	100.00%		

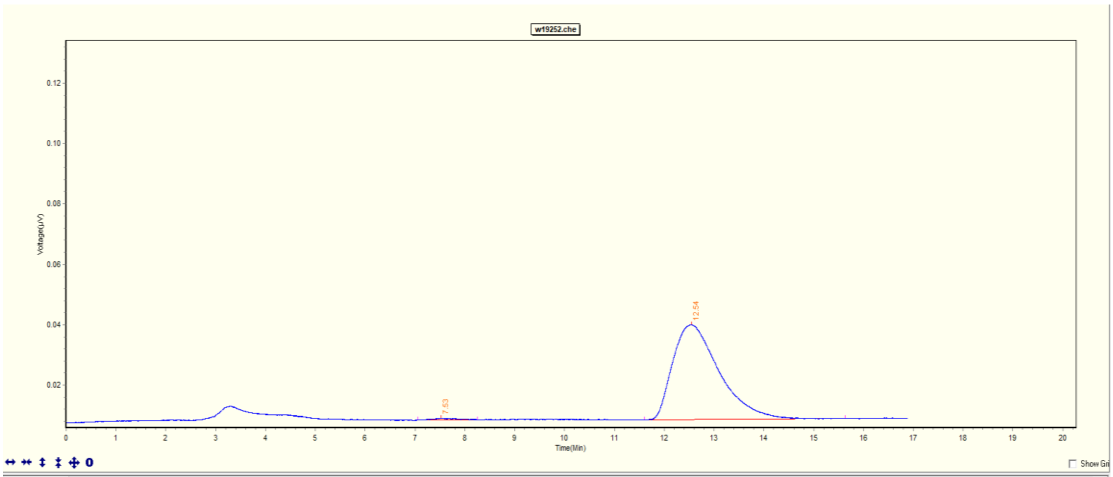

No.	Retention Time	Peak Area	Peak Height	Peak Area(%)	Peak Width	Peak Type
1	7.53	8942	305	0.88%	1.196	BB
2	12.54	1009892	15710	99.12%	4.028	BB
Total		1,018,834	16,015	100.00%		

Supplementary Figure 166. HPLC spectrum of compound **3a**

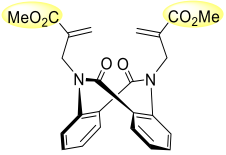

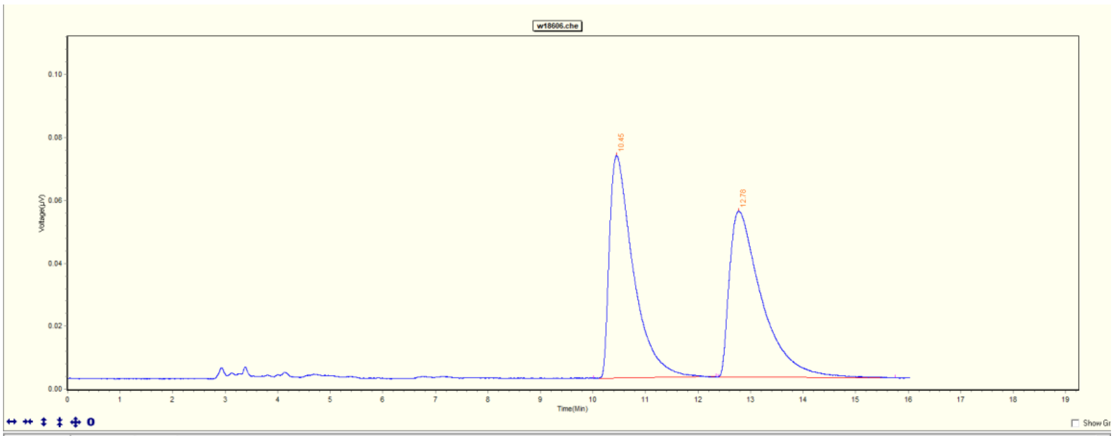

No.	Retention Time	Peak Area	Peak Height	Peak Area(%)	Peak Width	Peak Type
1	10.45	1117177	35357	49.92%	2.336	BB
2	12.78	1120821	26374	50.08%	3.37	BB
Total		2,237,998	61,731	100.00%		

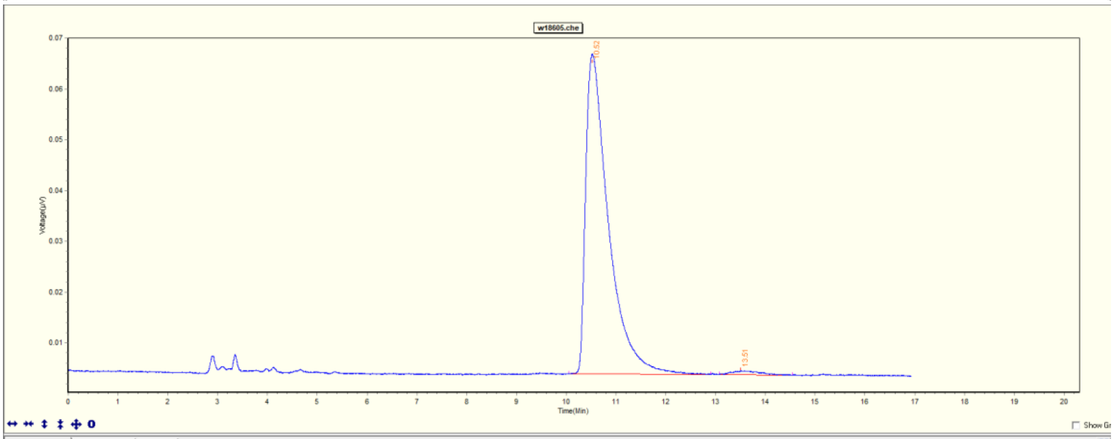

No.	Retention Time	Peak Area	Peak Height	Peak Area(%)	Peak Width	Peak Type
1	10.52	998862	31500	98.62%	2.85	BB
2	13.51	14028	375	1.38%	1.465	BB
Total		1,012,890	31,875	100.00%		

Supplementary Figure 167. HPLC spectrum of compound **3b**

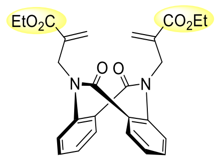

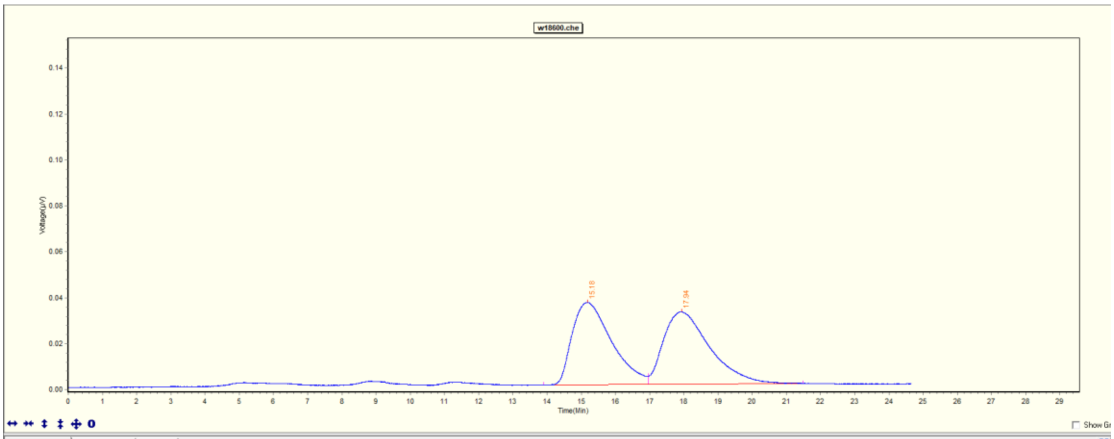

No.	Retention Time	Peak Area	Peak Height	Peak Area(%)	Peak Width	Peak Type
1	15.18	1436417	17897	49.20%	3.064	BV
2	17.94	1483261	15742	50.80%	4.518	VB
Total		2,919,678	33,639	100.00%		

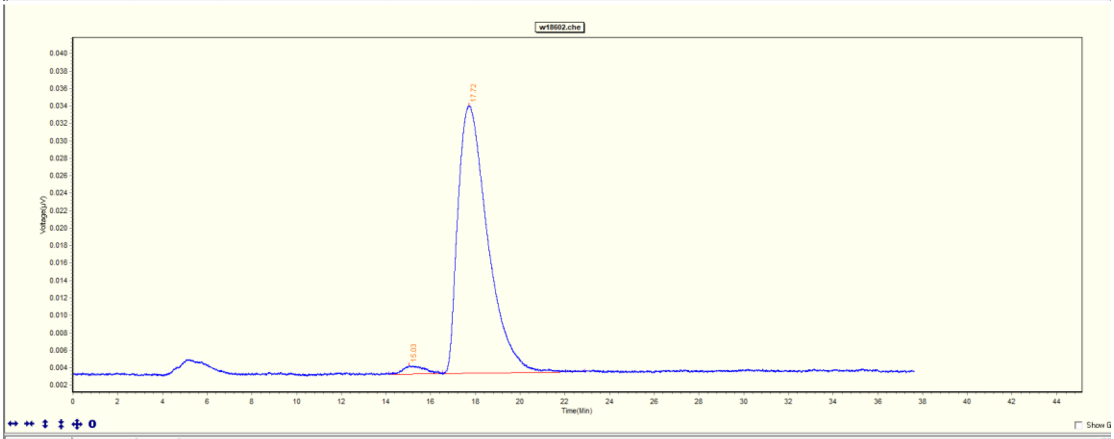

No.	Retention Time	Peak Area	Peak Height	Peak Area(%)	Peak Width	Peak Type
1	15.03	30612	474	2.12%	2.406	BV
2	17.72	1411656	15311	97.88%	6.359	VB
Total		1,442,268	15,785	100.00%		

Supplementary Figure 168. HPLC spectrum of compound **3c**

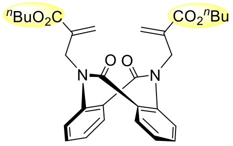

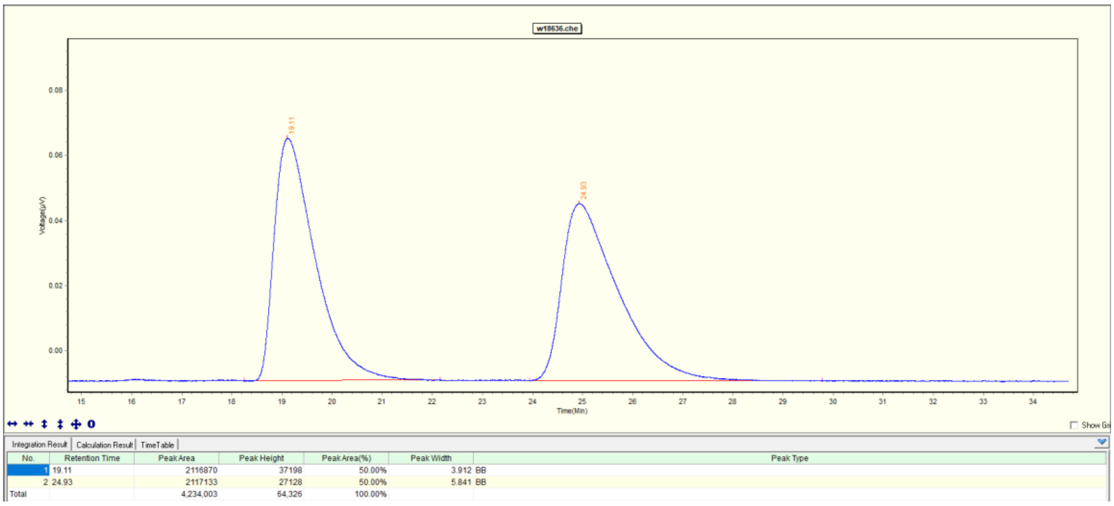

No.	Retention Time	Peak Area	Peak Height	Peak Area(%)	Peak Width	Peak Type
1	19.11	2116870	37198	50.00%	3.912	BB
2	24.93	2117133	27128	50.00%	5.841	BB
Total		4,234,003	64,326	100.00%		

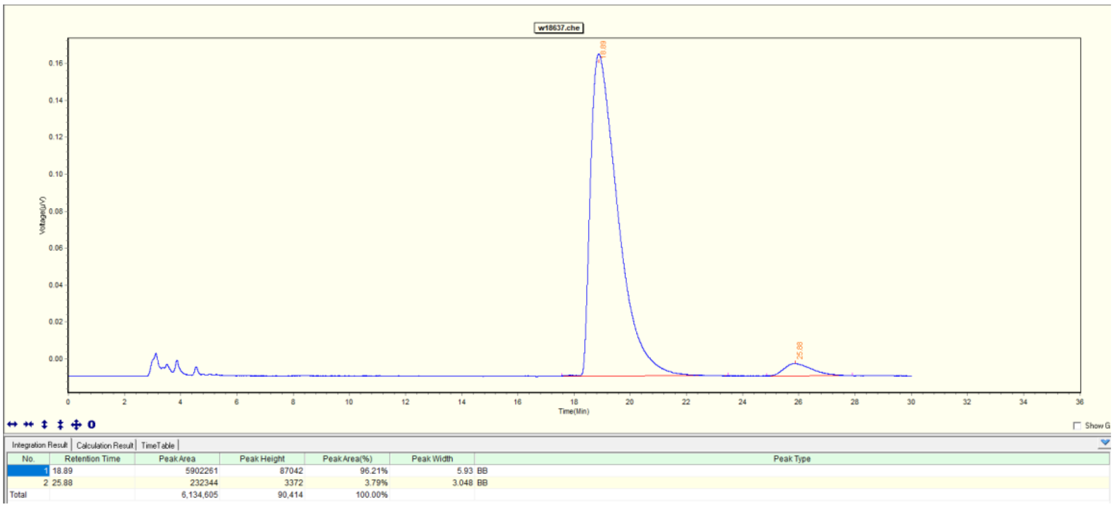

No.	Retention Time	Peak Area	Peak Height	Peak Area(%)	Peak Width	Peak Type
1	18.89	5902261	87042	96.21%	5.93	BB
2	25.88	232344	3372	3.79%	3.048	BB
Total		6,134,605	90,414	100.00%		

Supplementary Figure 169. HPLC spectrum of compound **3d**

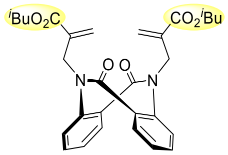

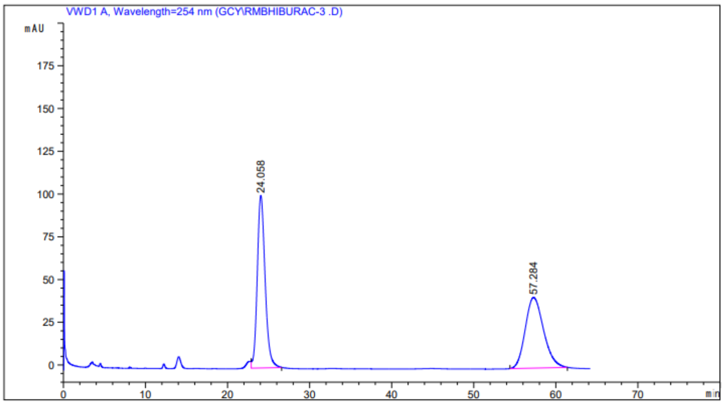

Area Percent Report

Sorted By : Signal
Multiplier : 1.0000
Dilution : 1.0000
Use Multiplier & Dilution Factor with ISTDs

Signal 1: VWD1 A, Wavelength=254 nm

Peak #	RetTime [min]	Type	Width [min]	Area mAU *s	Height [mAU]	Area %
1	24.058	VB	1.0194	6689.39209	100.92067	51.0392
2	57.284	BB	2.1944	6416.98389	41.32864	48.9608
Totals :				1.31064e4	142.24931	

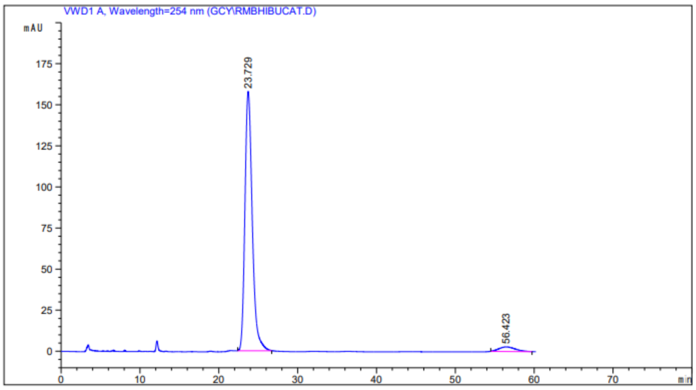

Area Percent Report

Sorted By : Signal
Multiplier : 1.0000
Dilution : 1.0000
Use Multiplier & Dilution Factor with ISTDs

Signal 1: VWD1 A, Wavelength=254 nm

Peak #	RetTime [min]	Type	Width [min]	Area mAU *s	Height [mAU]	Area %
1	23.729	BB	0.9908	1.01930e4	157.81914	96.1846
2	56.423	BB	1.7012	404.33618	2.79689	3.8154
Totals :				1.05974e4	160.61603	

Supplementary Figure 170. HPLC spectrum of compound **3e**

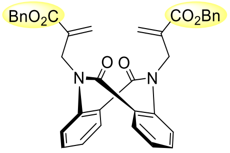

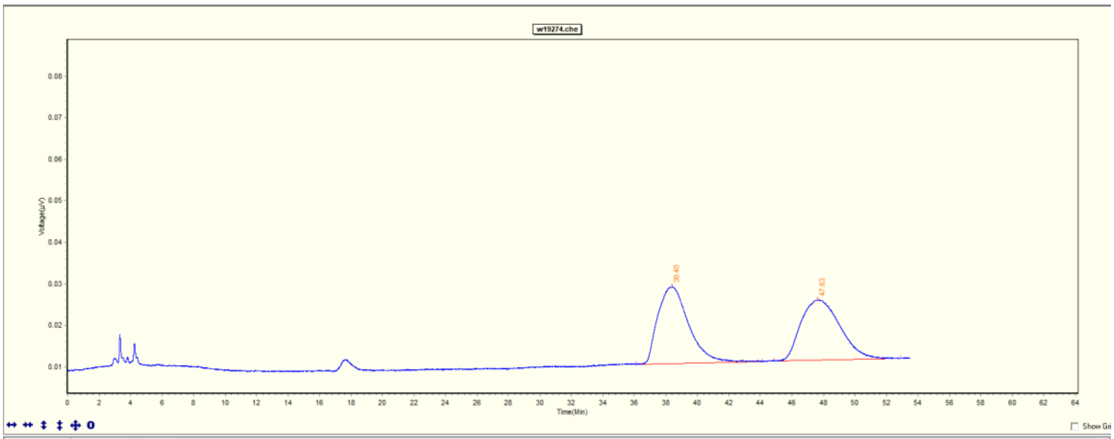

No.	Retention Time	Peak Area	Peak Height	Peak Area(%)	Peak Width	Peak Type
1	38.40	1254633	9221	50.20%	7.996	BB
2	47.63	1244624	7239	49.80%	8.038	BB
Total		2,499,257	16,460	100.00%		

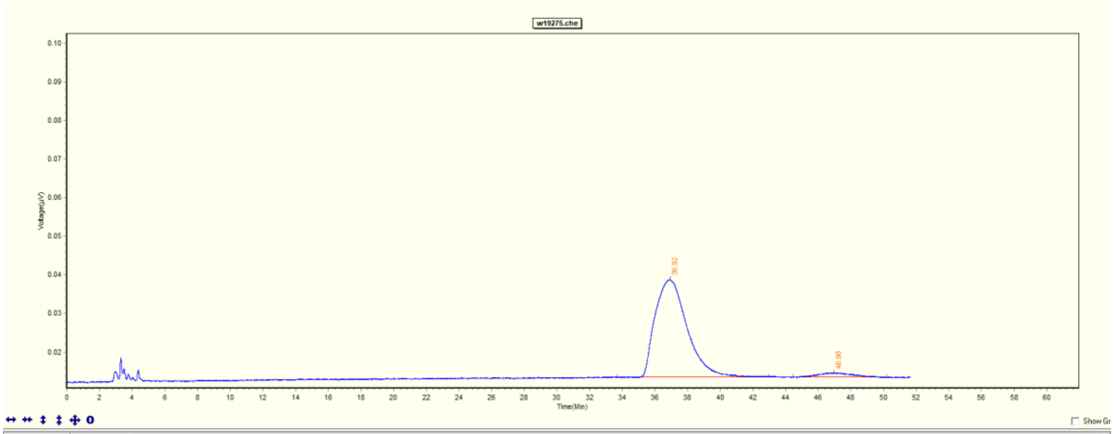

No.	Retention Time	Peak Area	Peak Height	Peak Area(%)	Peak Width	Peak Type
1	36.92	1692919	12597	95.48%	9.259	BB
2	46.98	80074	564	4.52%	5.769	BB
Total		1,772,993	13,161	100.00%		

Supplementary Figure 171. HPLC spectrum of compound **3f**

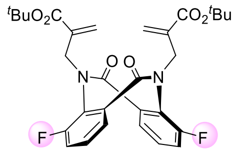

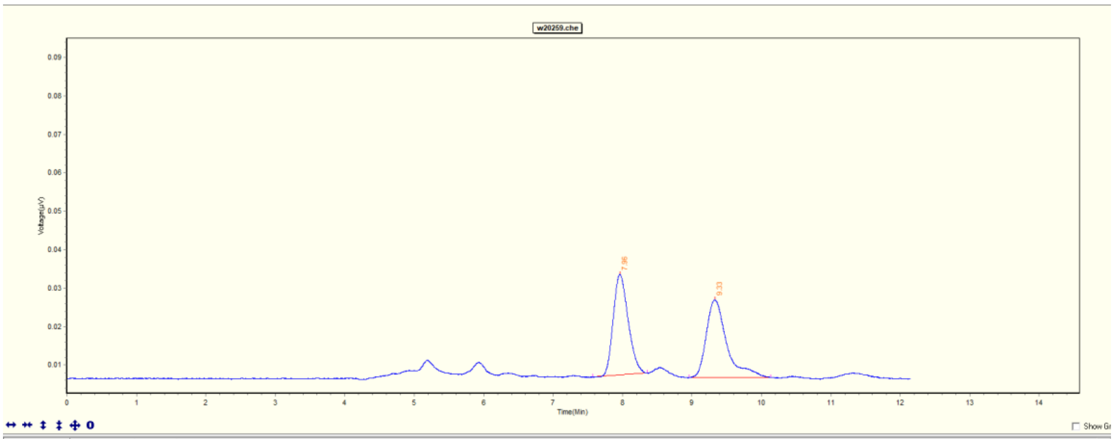

Integration Result | Calculation Result | TimeTable

No.	Retention Time	Peak Area	Peak Height	Peak Area(%)	Peak Width	Peak Type
1	7.96	195007	13064	48.67%	0.784	BB
2	9.33	205669	10080	51.33%	1.176	BB
Total		400,676	23,144	100.00%		

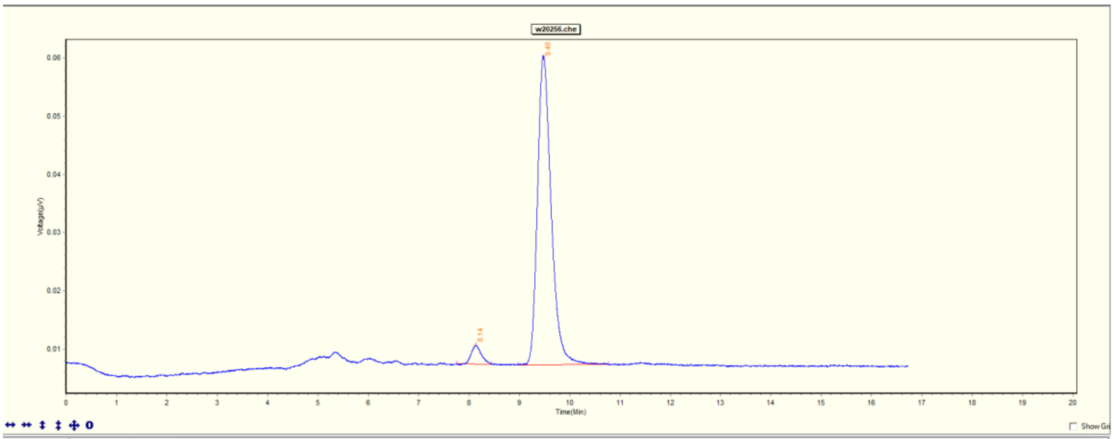

Integration Result | Calculation Result | TimeTable

No.	Retention Time	Peak Area	Peak Height	Peak Area(%)	Peak Width	Peak Type
1	8.14	21424	1561	4.10%	0.698	BB
2	9.48	501670	26537	95.90%	1.79	BB
Total		523,094	28,098	100.00%		

Supplementary Figure 172. HPLC spectrum of compound **3g**

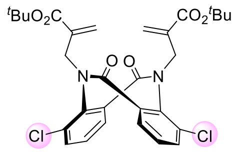

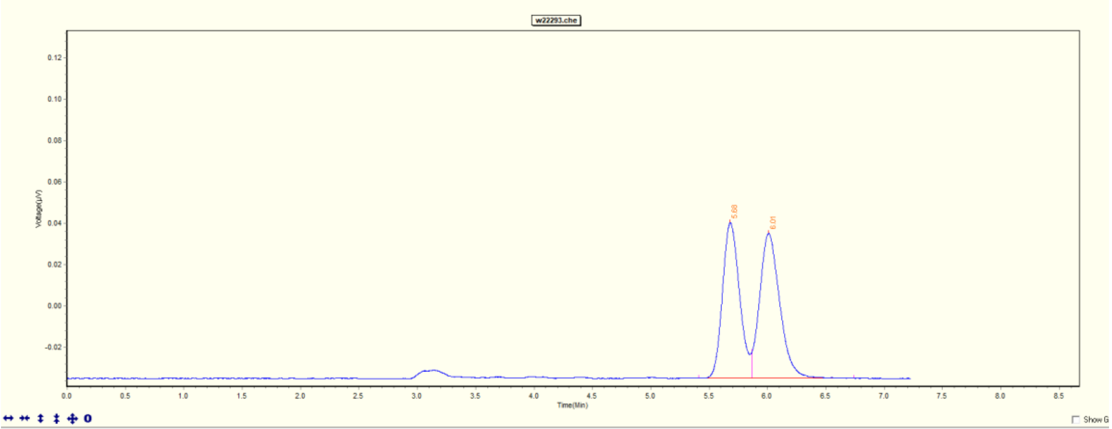

Integration Result | Calculation Result | TimeTable

No.	Retention Time	Peak Area	Peak Height	Peak Area(%)	Peak Width	Peak Type
1	5.68	382270	37543	47.95%	0.456	BV
2	6.01	414948	34921	52.05%	0.872	VB
Total		797,218	72,464	100.00%		

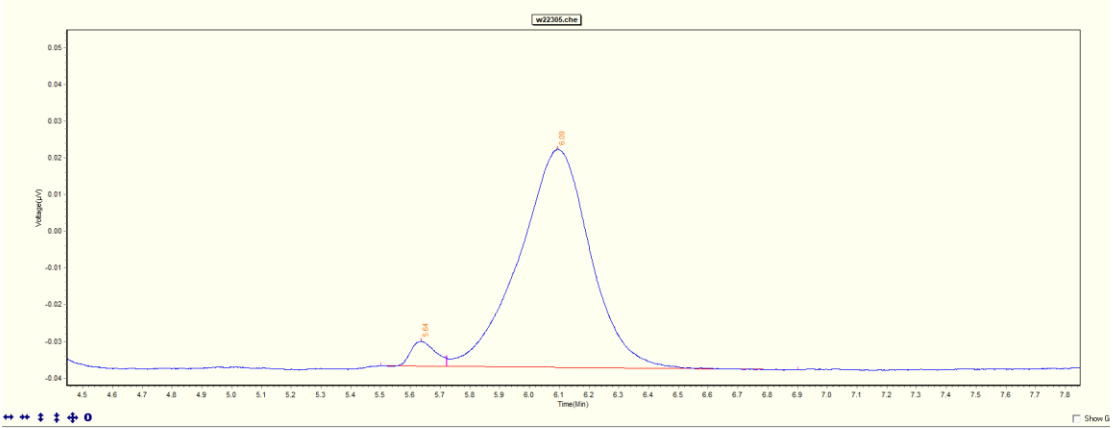

Integration Result | Calculation Result | TimeTable

No.	Retention Time	Peak Area	Peak Height	Peak Area(%)	Peak Width	Peak Type
1	5.64	18688	3353	3.68%	0.219	BV
2	6.09	489119	29682	96.32%	1.18	VB
Total		507,807	33,035	100.00%		

Supplementary Figure 173. HPLC spectrum of compound **3h**

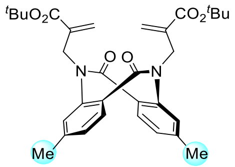

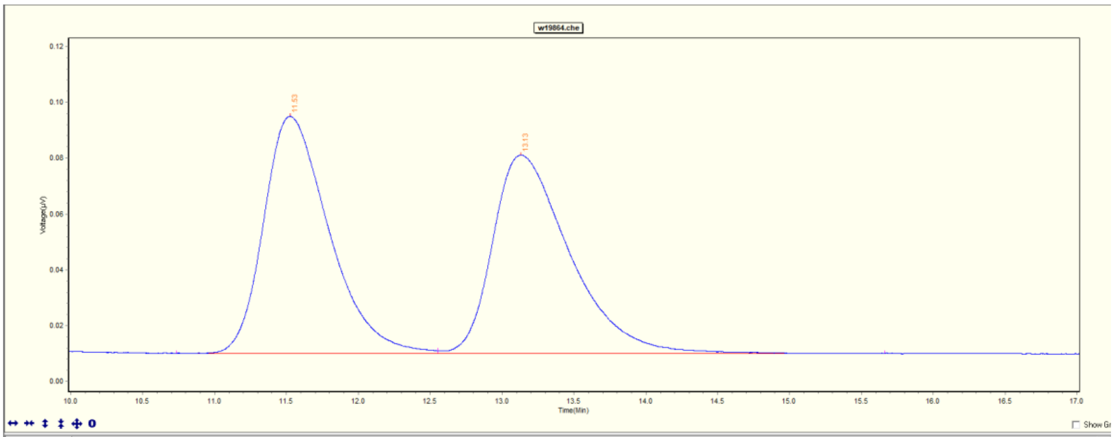

No.	Retention Time	Peak Area	Peak Height	Peak Area(%)	Peak Width	Peak Type
1	11.53	1340077	42492	50.32%	1.819	BV
2	13.13	1322862	35564	49.68%	3.107	VB
Total		2,662,939	78,056	100.00%		

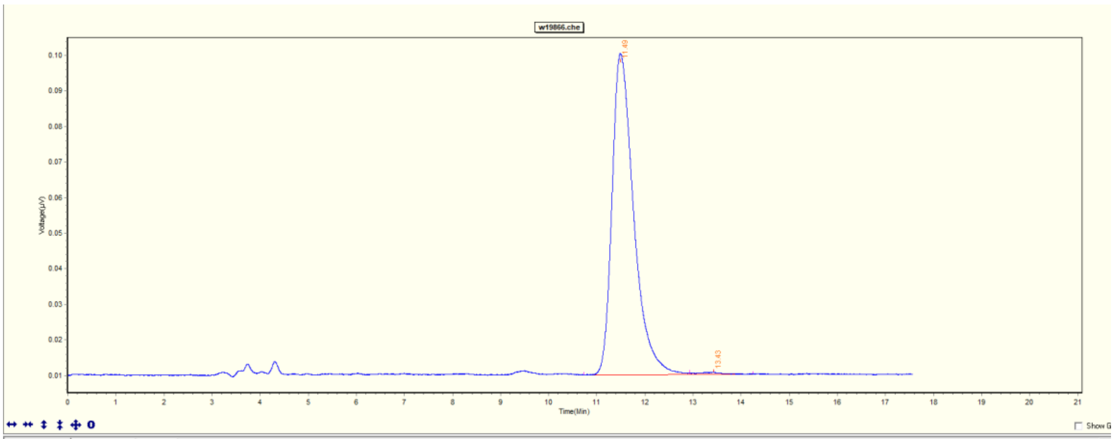

No.	Retention Time	Peak Area	Peak Height	Peak Area(%)	Peak Width	Peak Type
1	11.49	1413487	45089	99.36%	2.198	BV
2	13.43	9156	294	0.64%	1.313	VB
Total		1,422,643	45,383	100.00%		

Supplementary Figure 174. HPLC spectrum of compound **3i**

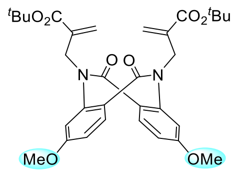

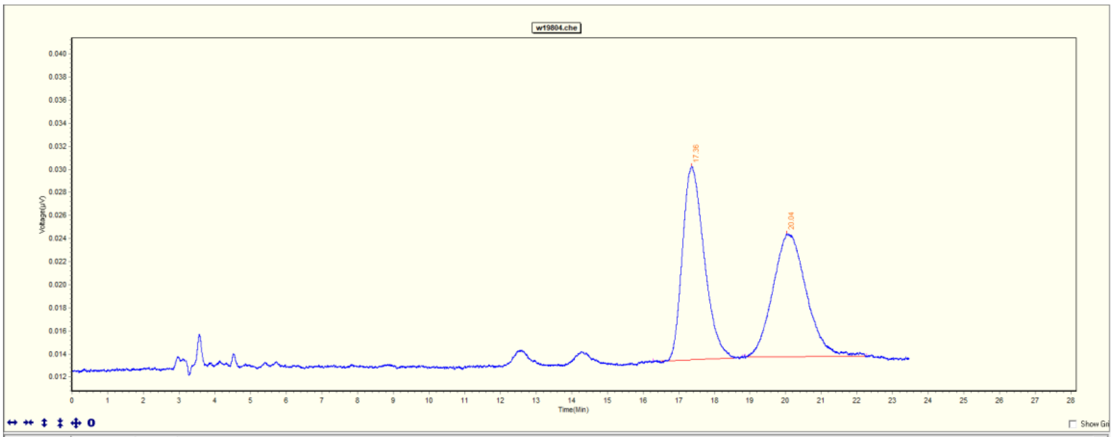

Integration Result | Calculation Result | TimeTable

No.	Retention Time	Peak Area	Peak Height	Peak Area(%)	Peak Width	Peak Type
1	17.36	355095	8404	50.71%	2.502	BB
2	20.04	345209	5314	49.29%	3.409	BB
Total		700,304	13,718	100.00%		

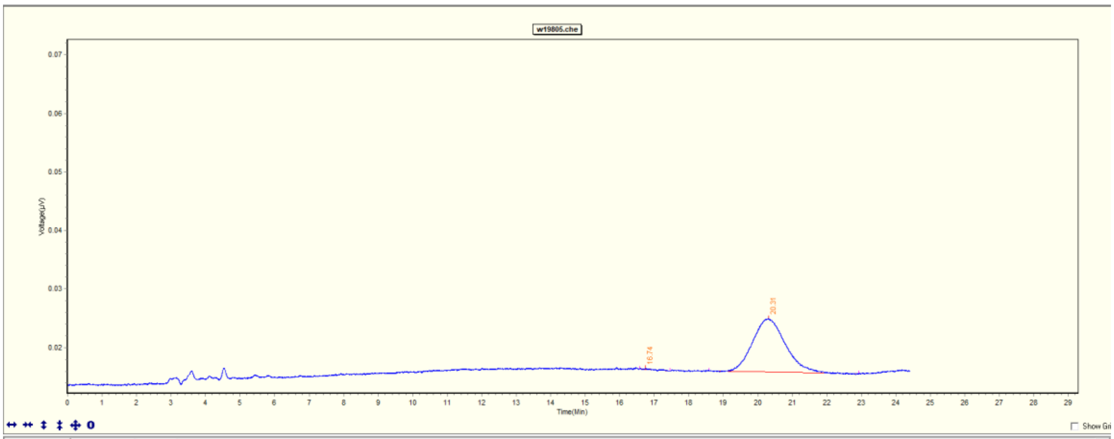

Integration Result | Calculation Result | TimeTable

No.	Retention Time	Peak Area	Peak Height	Peak Area(%)	Peak Width	Peak Type
1	16.74	571	77	0.19%	0.883	BB
2	20.31	297681	4573	99.81%	4.34	BB
Total		298,252	4,650	100.00%		

Supplementary Figure 175. HPLC spectrum of compound **3j**

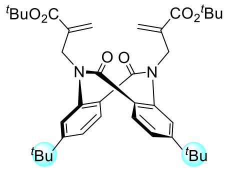

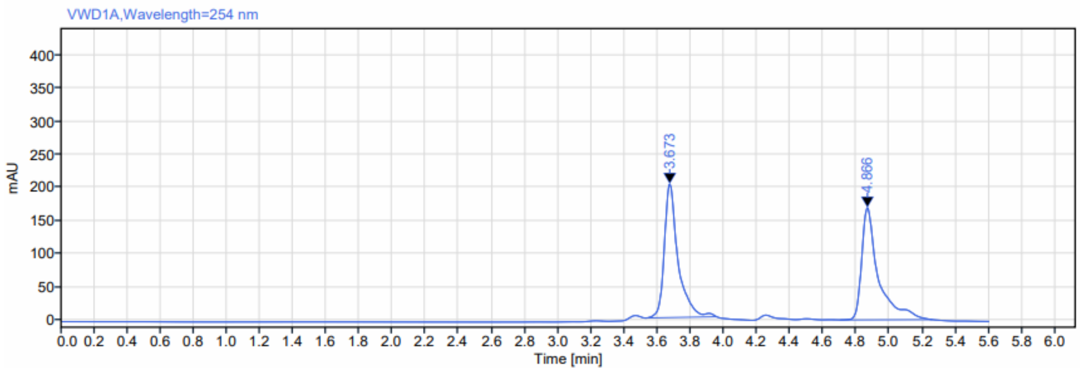

Signal: VWD1A,Wavelength=254 nm

RT [min]	Type	Width [min]	Area	Height	Area%	Name
3.673	MM m	0.43	1159.61	201.28	49.21	
4.866	VM m	0.56	1196.83	169.08	50.79	
		**Sum**	**2356.44**			

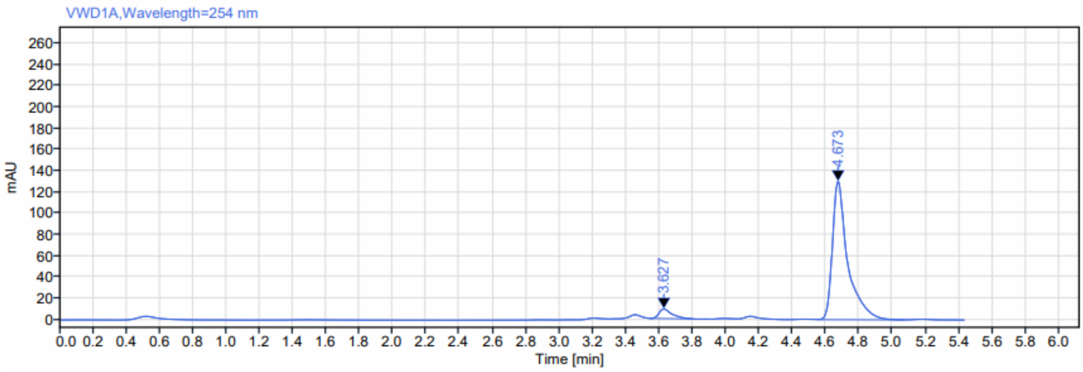

Signal: VWD1A,Wavelength=254 nm

RT [min]	Type	Width [min]	Area	Height	Area%	Name
3.627	MM m	0.26	48.93	9.04	5.55	
4.673	VV	0.52	832.82	130.04	94.45	
		**Sum**	**881.75**			

Supplementary Figure 176. HPLC spectrum of compound **3k**

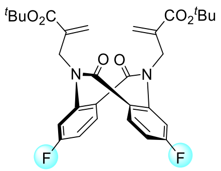

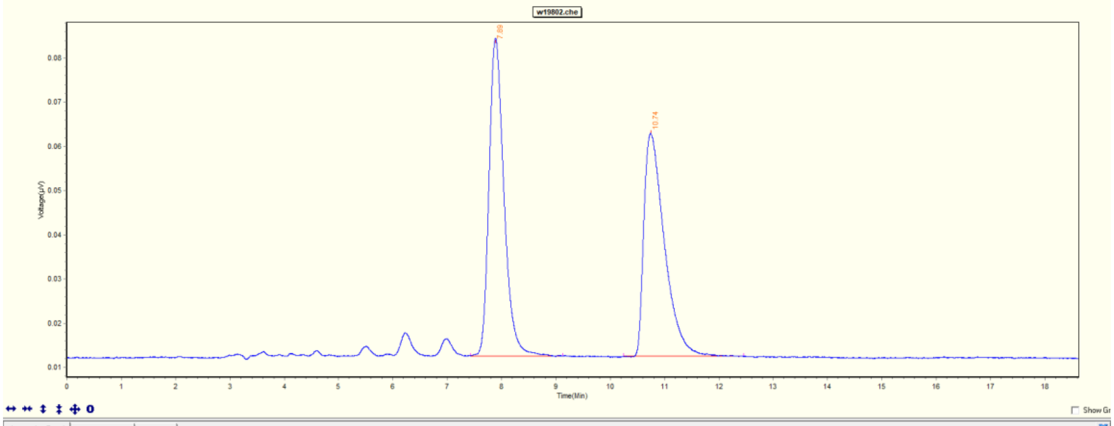

No.	Retention Time	Peak Area	Peak Height	Peak Area(%)	Peak Width	Peak Type
1	7.89	688089	35920	50.93%	1.698	BB
2	10.74	662980	25217	49.07%	2.211	BB
Total		1,351,069	61,137	100.00%		

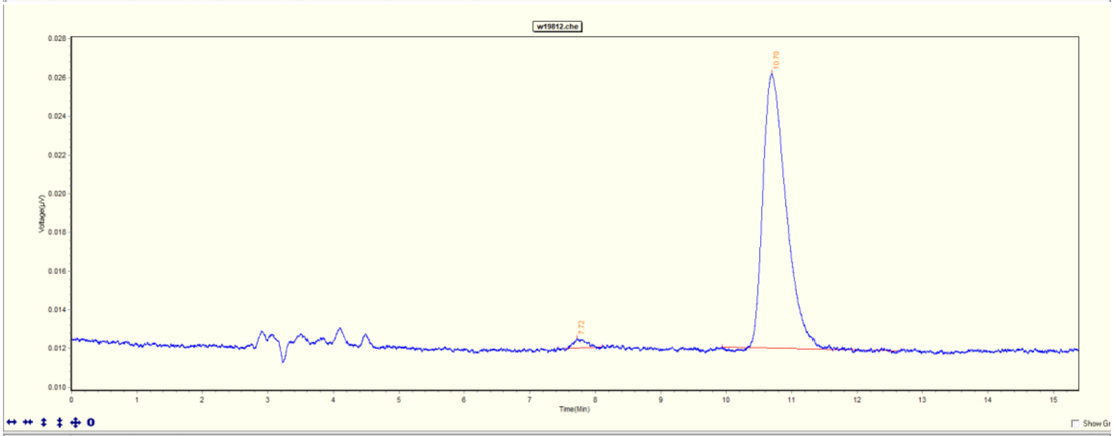

No.	Retention Time	Peak Area	Peak Height	Peak Area(%)	Peak Width	Peak Type
1	7.72	2867	242	1.63%	0.669	BB
2	10.70	172707	7099	98.37%	2.735	BB
Total		175,574	7,341	100.00%		

Supplementary Figure 177. HPLC spectrum of compound **3l**

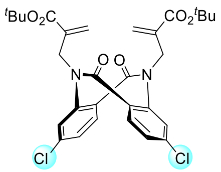

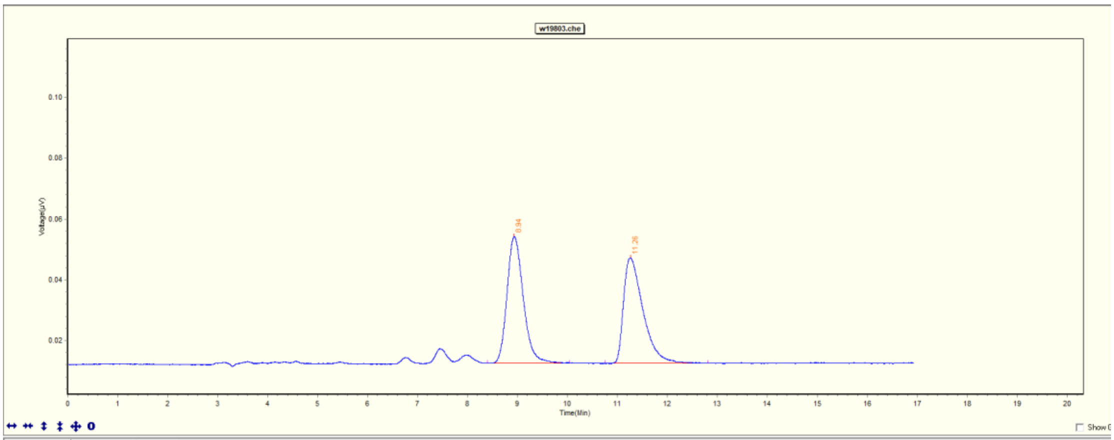

Integration Result | Calculation Result | TimeTable

No.	Retention Time	Peak Area	Peak Height	Peak Area(%)	Peak Width	Peak Type
1	8.94	470014	20850	50.09%	1.639	BB
2	11.26	468242	17381	49.91%	2.053	BB
Total		938,256	38,231	100.00%		

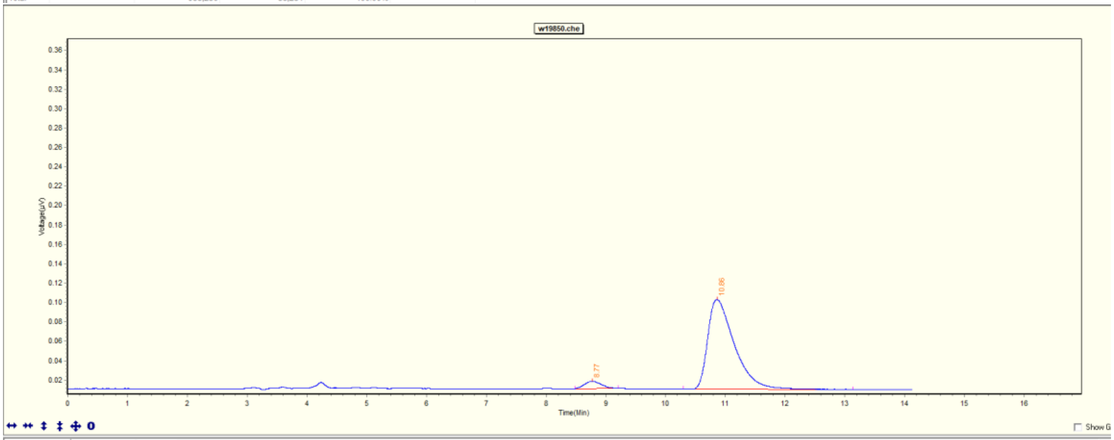

Integration Result | Calculation Result | TimeTable

No.	Retention Time	Peak Area	Peak Height	Peak Area(%)	Peak Width	Peak Type
1	8.77	71012	3693	4.74%	0.722	BB
2	10.86	1427352	46226	95.26%	2.835	BB
Total		1,498,364	49,919	100.00%		

Supplementary Figure 178. HPLC spectrum of compound **3m**

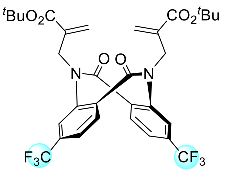

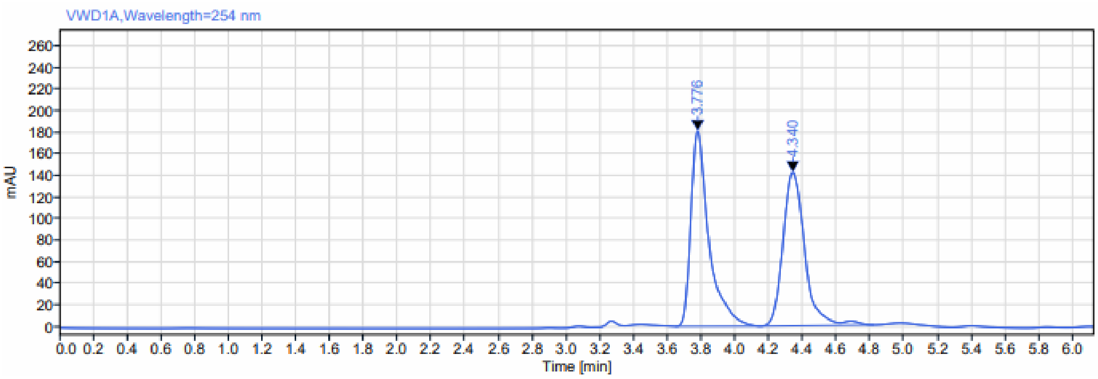

Signal: VWD1A,Wavelength=254 nm

RT [min]	Type	Width [min]	Area	Height	Area%	Name
3.776	BB	0.50	1339.09	180.81	51.17	
4.340	BM m	0.67	1277.71	142.13	48.83	
		**Sum**	**2616.81**			

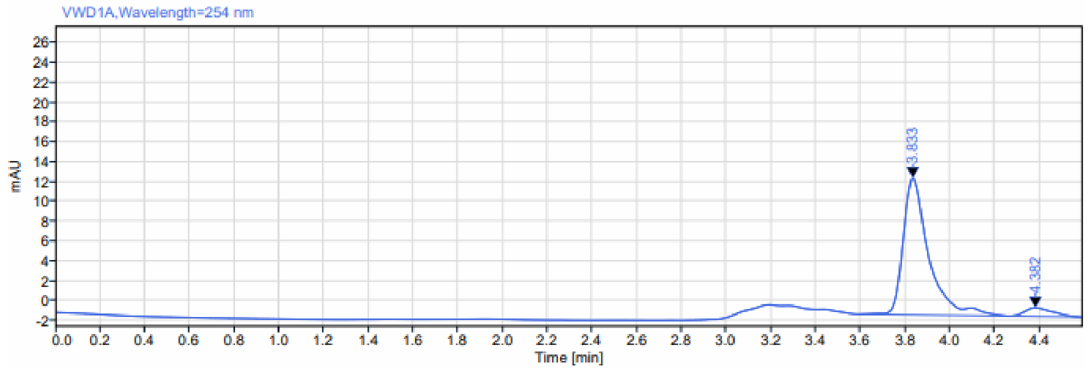

Signal: VWD1A,Wavelength=254 nm

RT [min]	Type	Width [min]	Area	Height	Area%	Name
3.833	BM m	0.68	109.76	13.78	93.78	
4.382	MM m	0.30	7.28	0.86	6.22	
		**Sum**	**117.04**			

Supplementary Figure 179. HPLC spectrum of compound **3n**

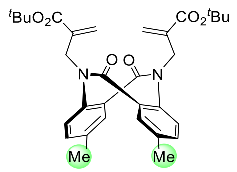

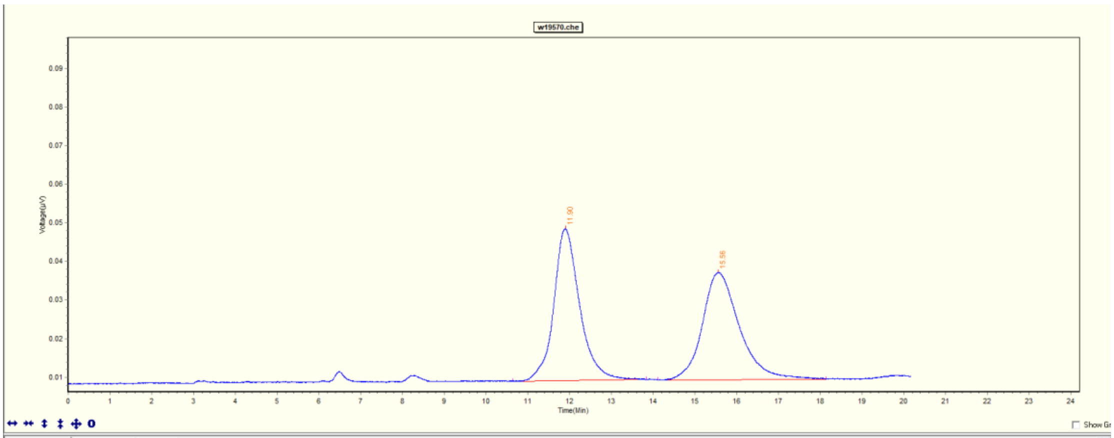

No.	Retention Time	Peak Area	Peak Height	Peak Area(%)	Peak Width	Peak Type
1	11.90	844761	19657	49.81%	3.205	BB
2	15.56	851073	13897	50.19%	4.031	BB
Total		1,695,834	33,554	100.00%		

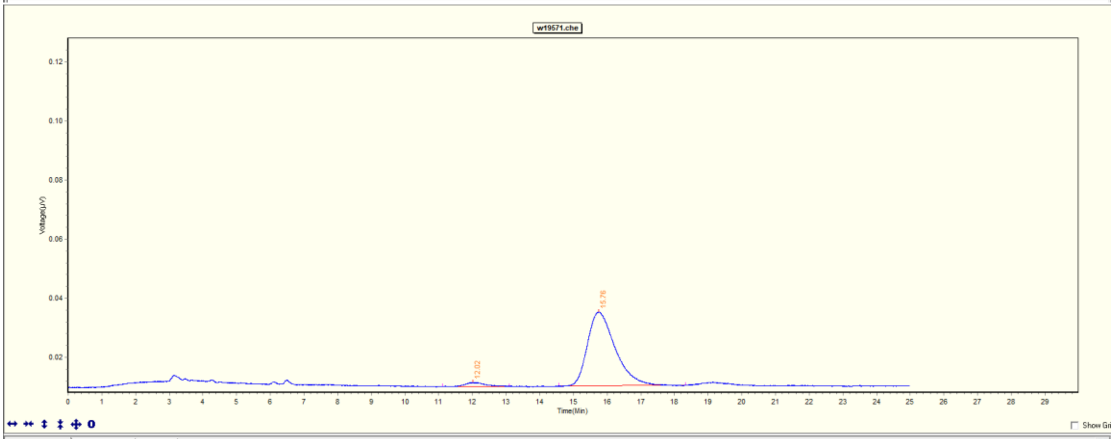

No.	Retention Time	Peak Area	Peak Height	Peak Area(%)	Peak Width	Peak Type
1	12.02	26543	665	3.57%	1.974	BB
2	15.76	717305	12516	96.43%	3.751	BB
Total		743,848	13,181	100.00%		

Supplementary Figure 180. HPLC spectrum of compound **3o**

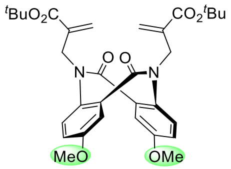

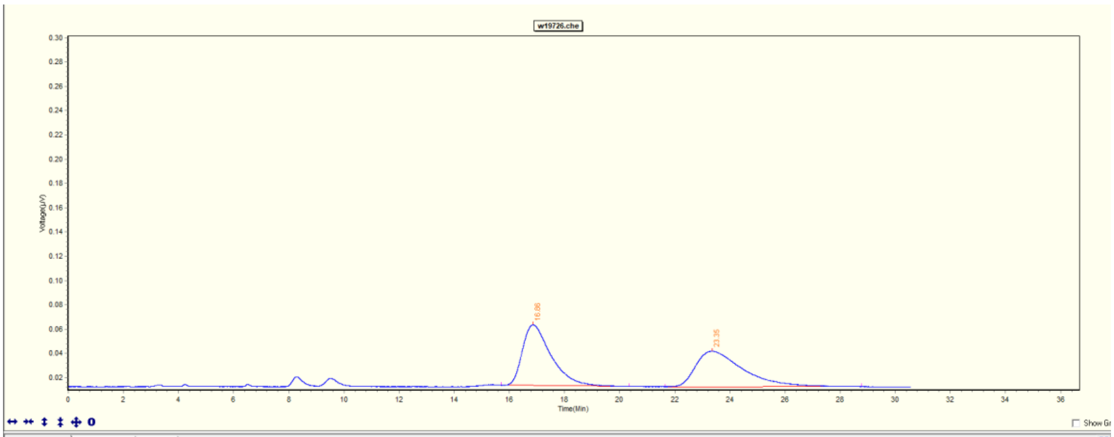

Integration Result | Calculation Result | TimeTable

No.	Retention Time	Peak Area	Peak Height	Peak Area(%)	Peak Width	Peak Type
1	16.86	1808661	25031	50.89%	4.643	BB
2	23.35	1745165	14712	49.11%	7.121	BB
Total		3,553,826	39,743	100.00%		

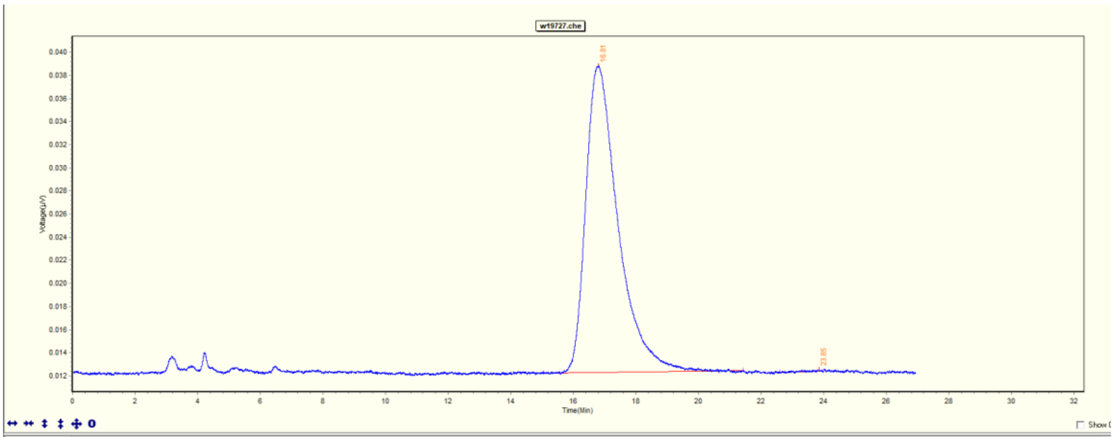

Integration Result | Calculation Result | TimeTable

No.	Retention Time	Peak Area	Peak Height	Peak Area(%)	Peak Width	Peak Type
1	16.81	950748	13255	99.91%	6.384	BB
2	23.85	869	70	0.09%	1.404	BB
Total		951,617	13,325	100.00%		

Supplementary Figure 181. HPLC spectrum of compound **3p**

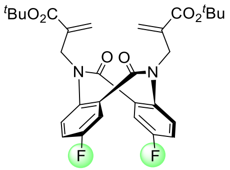

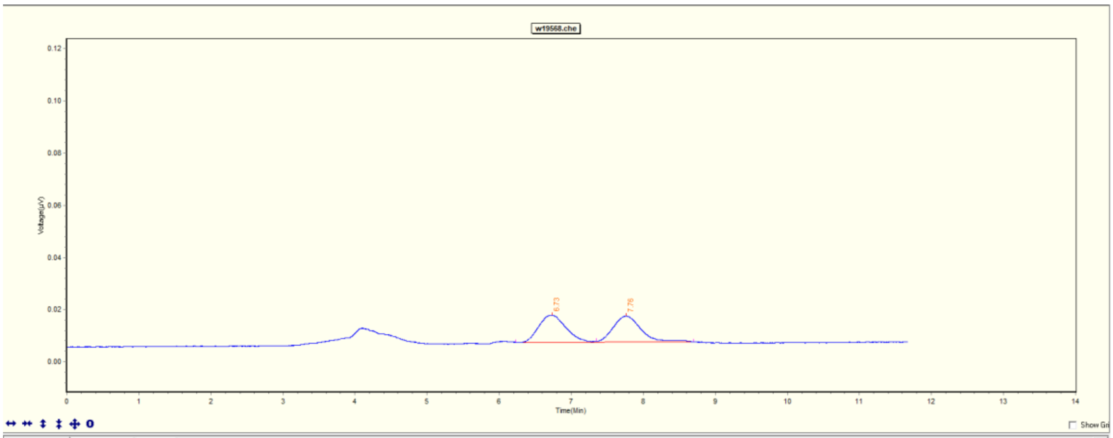

No.	Retention Time	Peak Area	Peak Height	Peak Area(%)	Peak Width	Peak Type
1	6.73	134968	5199	50.75%	1.121	BV
2	7.76	131001	4933	49.25%	1.35	VB
Total		265,969	10,132	100.00%		

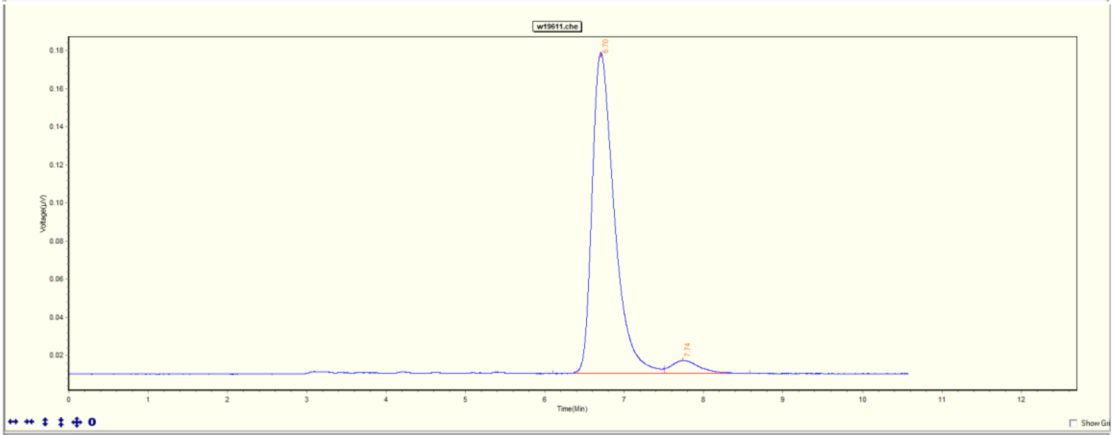

No.	Retention Time	Peak Area	Peak Height	Peak Area(%)	Peak Width	Peak Type
1	6.70	1634842	84136	95.28%	1.408	BV
2	7.74	81034	3363	4.72%	1.074	VB
Total		1,715,876	87,499	100.00%		

Supplementary Figure 182. HPLC spectrum of compound **3q**

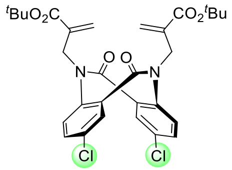

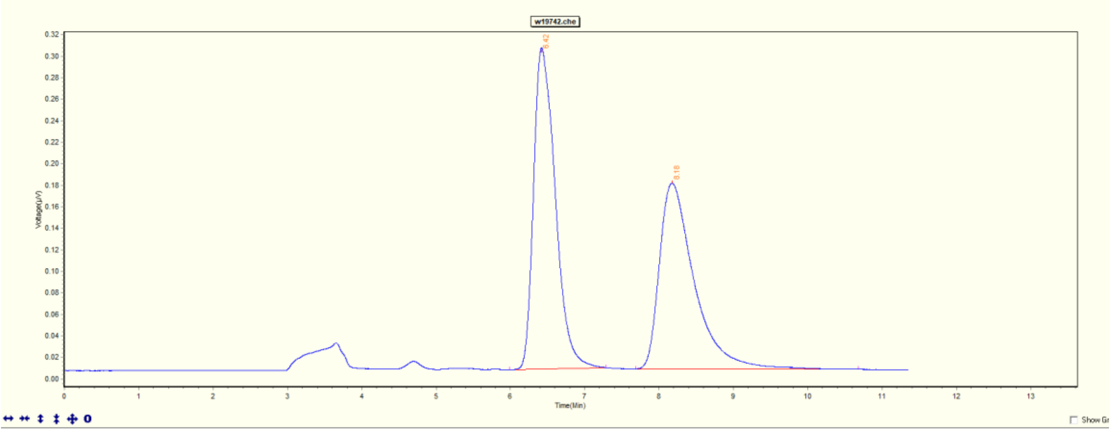

No.	Retention Time	Peak Area	Peak Height	Peak Area(%)	Peak Width	Peak Type
1	6.42	3027644	149223	51.78%	1.296	BB
2	8.18	2819030	86434	48.22%	2.993	BB
Total		5,846,674	235,657	100.00%		

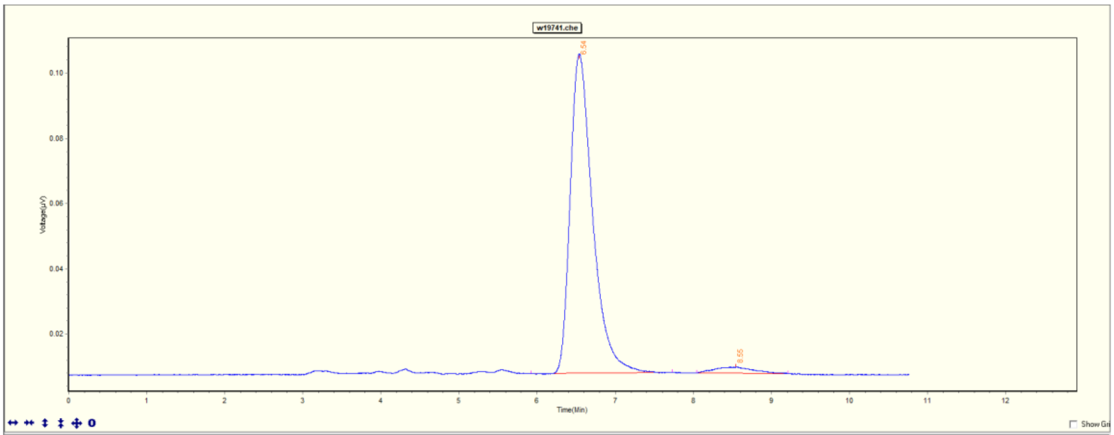

No.	Retention Time	Peak Area	Peak Height	Peak Area(%)	Peak Width	Peak Type
1	6.54	973211	48923	96.79%	1.812	BB
2	8.55	32314	931	3.21%	1.169	BB
Total		1,005,525	49,854	100.00%		

Supplementary Figure 183. HPLC spectrum of compound **3r**

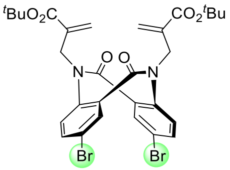

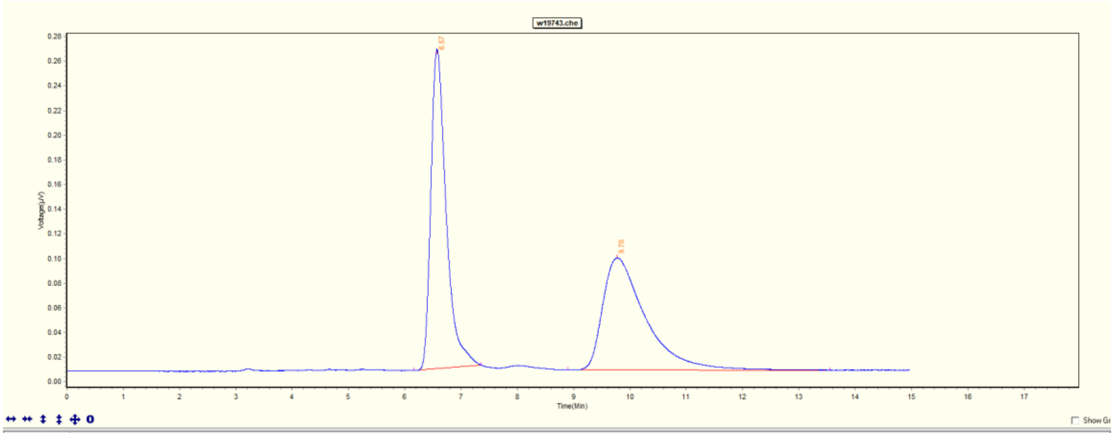

Integration Result | Calculation Result | TimeTable

No.	Retention Time	Peak Area	Peak Height	Peak Area(%)	Peak Width	Peak Type
1	6.57	2445702	129538	50.60%	1.191	BB
2	9.78	2387800	45433	49.40%	4.655	BB
Total		4,833,502	174,971	100.00%		

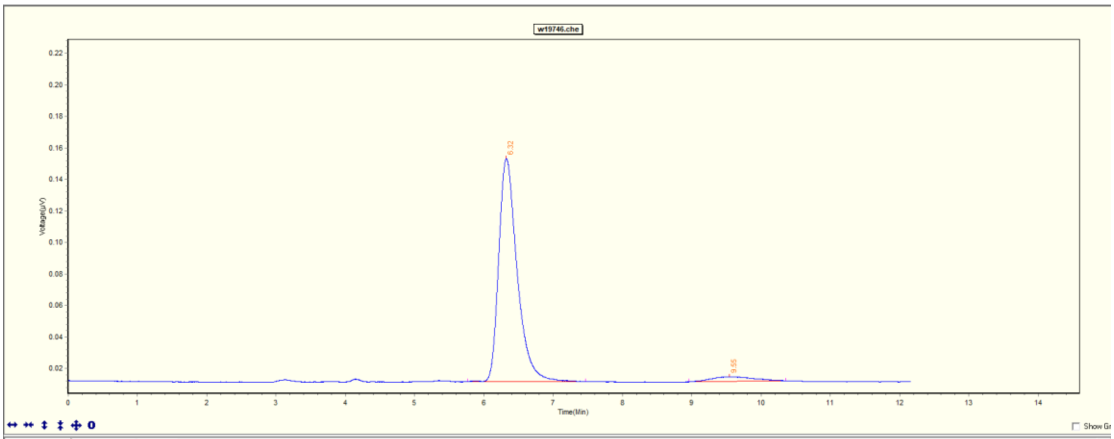

Integration Result | Calculation Result | TimeTable

No.	Retention Time	Peak Area	Peak Height	Peak Area(%)	Peak Width	Peak Type
1	6.32	1305447	70722	95.48%	1.704	BB
2	9.55	61796	1451	4.52%	1.398	BB
Total		1,367,243	72,173	100.00%		

Supplementary Figure 184. HPLC spectrum of compound **3s**

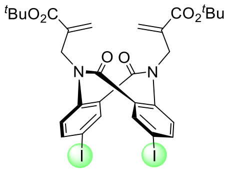

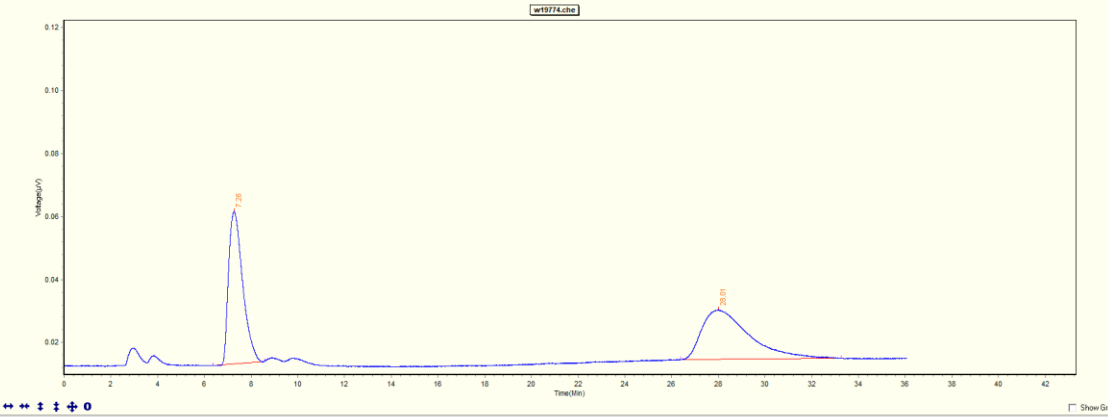

No.	Retention Time	Peak Area	Peak Height	Peak Area(%)	Peak Width	Peak Type
1	7.28	1008241	24155	47.80%	2.216	BB
2	28.01	1101131	7835	52.20%	6.874	BB
Total		2,109,372	31,990	100.00%		

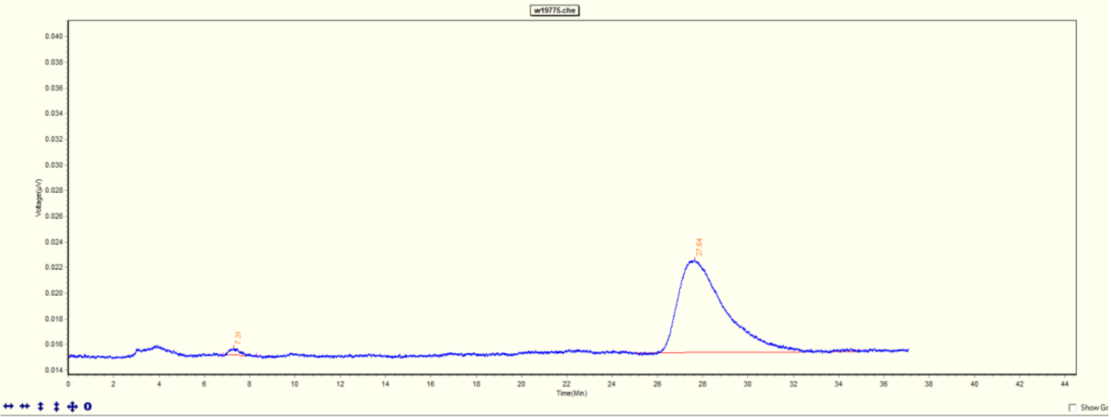

No.	Retention Time	Peak Area	Peak Height	Peak Area(%)	Peak Width	Peak Type
1	7.31	8510	267	1.62%	1.494	BB
2	27.64	515875	3618	98.38%	10.281	BB
Total		524,385	3,885	100.00%		

Supplementary Figure 185. HPLC spectrum of compound **3t**

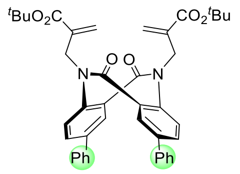

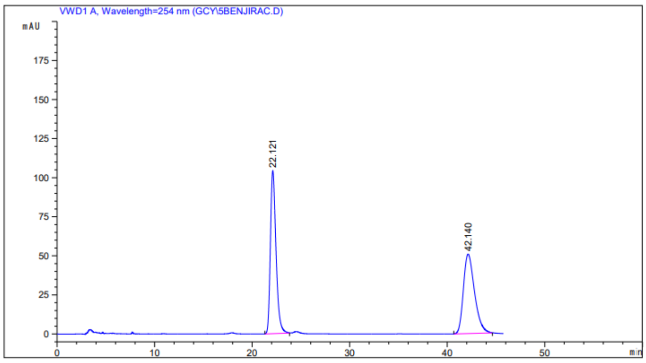

===

Area Percent Report

===

Sorted By : Signal
Multiplier : 1.0000
Dilution : 1.0000
Use Multiplier & Dilution Factor with ISTDs

Signal 1: VWD1 A, Wavelength=254 nm

Peak #	RetTime [min]	Type	Width [min]	Area mAU *s	Height [mAU]	Area %
1	22.121	BB	0.6076	4160.86475	104.40443	50.8182
2	42.140	BB	1.2197	4026.88086	50.83803	49.1818

Totals : 8187.74561 155.24247

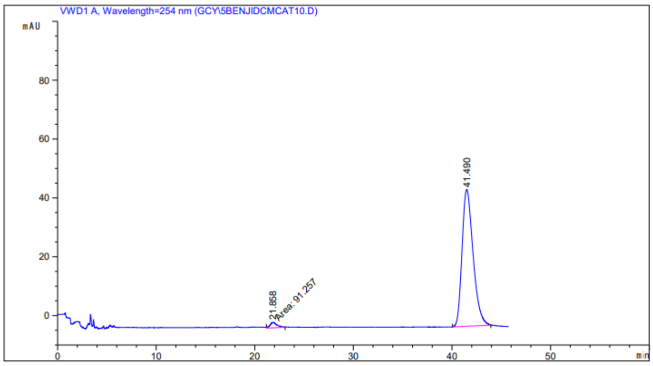

===

Area Percent Report

===

Sorted By : Signal
Multiplier : 1.0000
Dilution : 1.0000
Use Multiplier & Dilution Factor with ISTDs

Signal 1: VWD1 A, Wavelength=254 nm

Peak #	RetTime [min]	Type	Width [min]	Area mAU *s	Height [mAU]	Area %
1	21.858	MM	0.8336	91.25700	1.82451	2.4622
2	41.490	BB	1.1873	3615.01489	46.46017	97.5378

Totals : 3706.27189 48.28468

Supplementary Figure 186. HPLC spectrum of compound **3u**

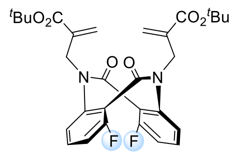

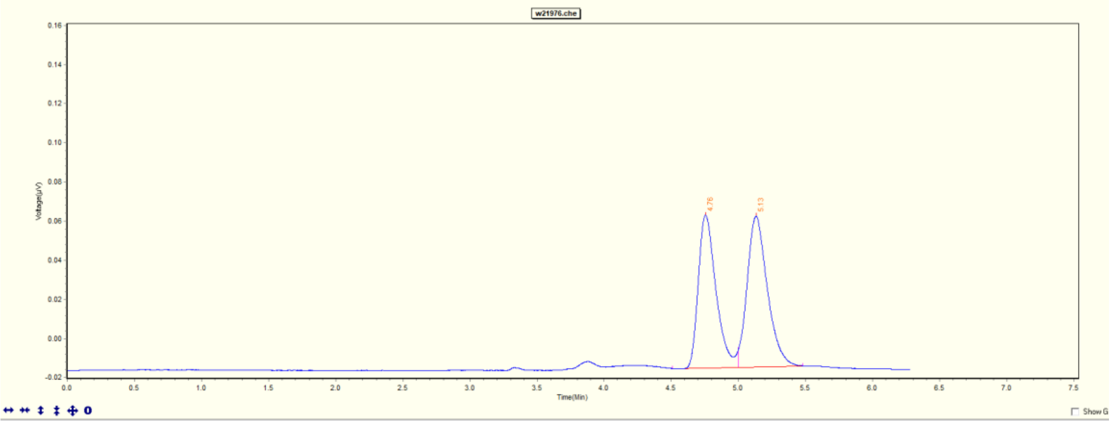

Integration Result | Calculation Result | TimeTable

No.	Retention Time	Peak Area	Peak Height	Peak Area(%)	Peak Width	Peak Type
1	4.76	357979	38987	47.13%	0.49	BV
2	5.13	401510	38495	52.87%	0.48	VB
Total		759,489	77,482	100.00%		

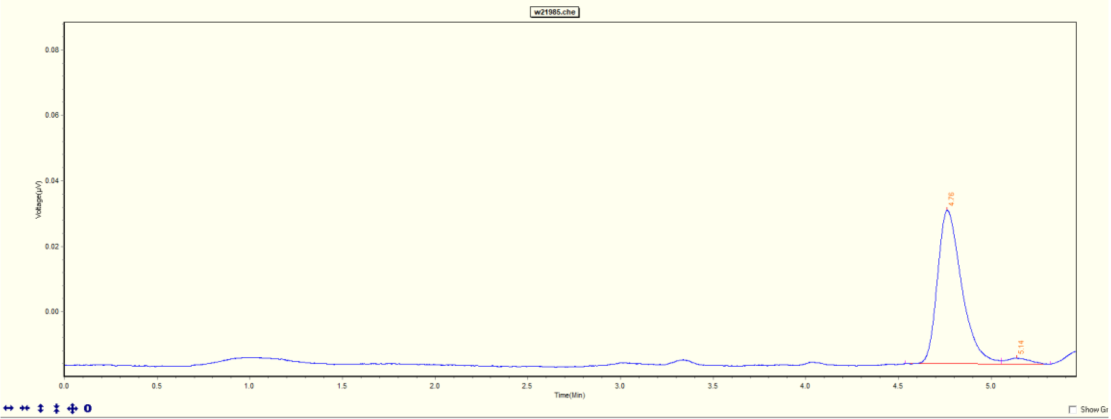

Integration Result | Calculation Result | TimeTable

No.	Retention Time	Peak Area	Peak Height	Peak Area(%)	Peak Width	Peak Type
1	4.76	213985	23458	96.38%	0.517	BV
2	5.14	8027	898	3.62%	0.265	VB
Total		222,012	24,356	100.00%		

Supplementary Figure 187. HPLC spectrum of compound **3v**

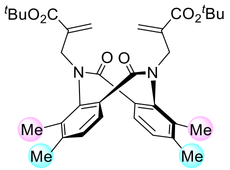

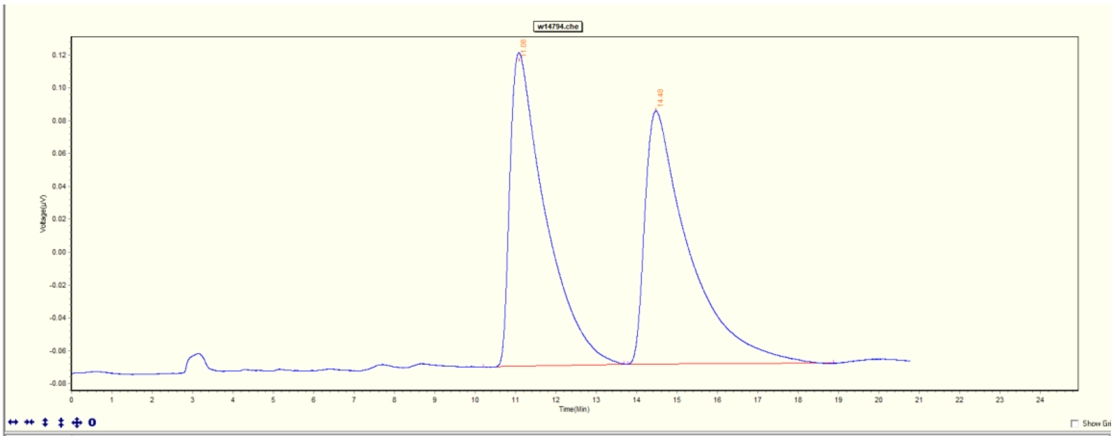

Integration Result | Calculation Result | TimeTable

No.	Retention Time	Peak Area	Peak Height	Peak Area(%)	Peak Width	Peak Type
1	11.08	5819275	95435	49.91%	3.493	BB
2	14.48	5841104	77045	50.09%	5.1	BB
Total		11,660,379	172,480	100.00%		

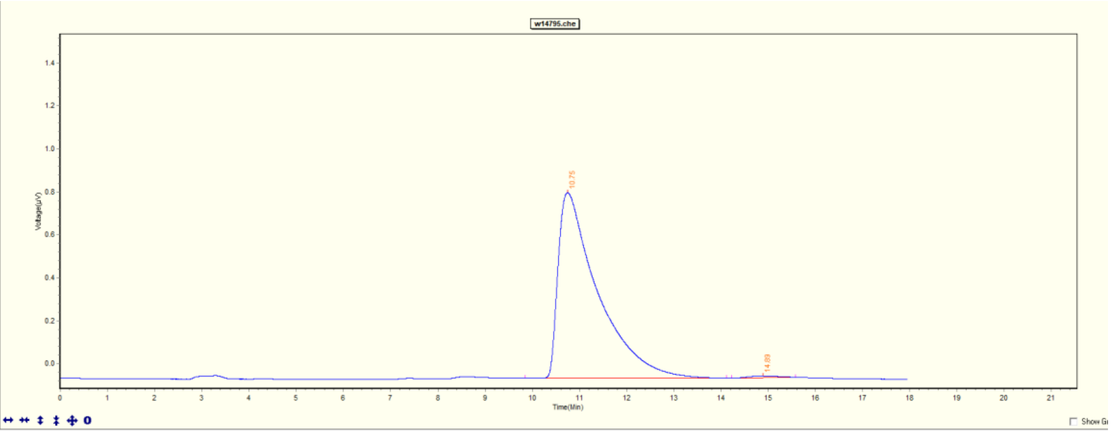

Integration Result | Calculation Result | TimeTable

No.	Retention Time	Peak Area	Peak Height	Peak Area(%)	Peak Width	Peak Type
1	10.75	25156353	431909	99.25%	4.264	BB
2	14.89	188835	4378	0.75%	1.351	BB
Total		25,345,188	436,287	100.00%		

Supplementary Figure 188. HPLC spectrum of compound **3w**

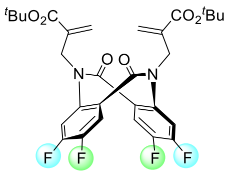

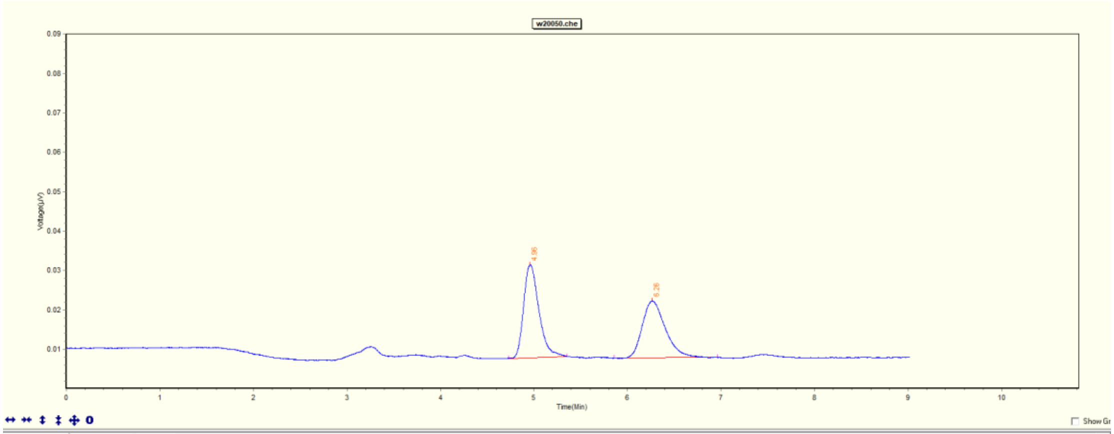

Integration Result | Calculation Result | TimeTable

No.	Retention Time	Peak Area	Peak Height	Peak Area(%)	Peak Width	Peak Type
1	4.96	133696	11803	51.91%	0.625	BB
2	6.26	123861	7217	48.09%	1.107	BB
Total		257,557	19,020	100.00%		

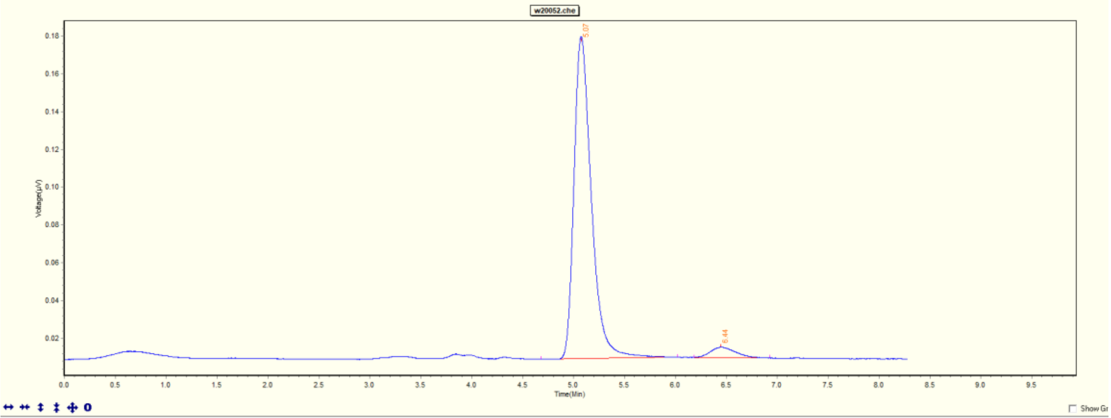

Integration Result | Calculation Result | TimeTable

No.	Retention Time	Peak Area	Peak Height	Peak Area(%)	Peak Width	Peak Type
1	5.07	1012460	85162	95.55%	1.343	BB
2	6.44	47100	2743	4.45%	0.743	BB
Total		1,059,560	87,905	100.00%		

Supplementary Figure 189. HPLC spectrum of compound **3x**

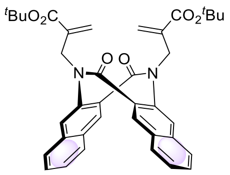

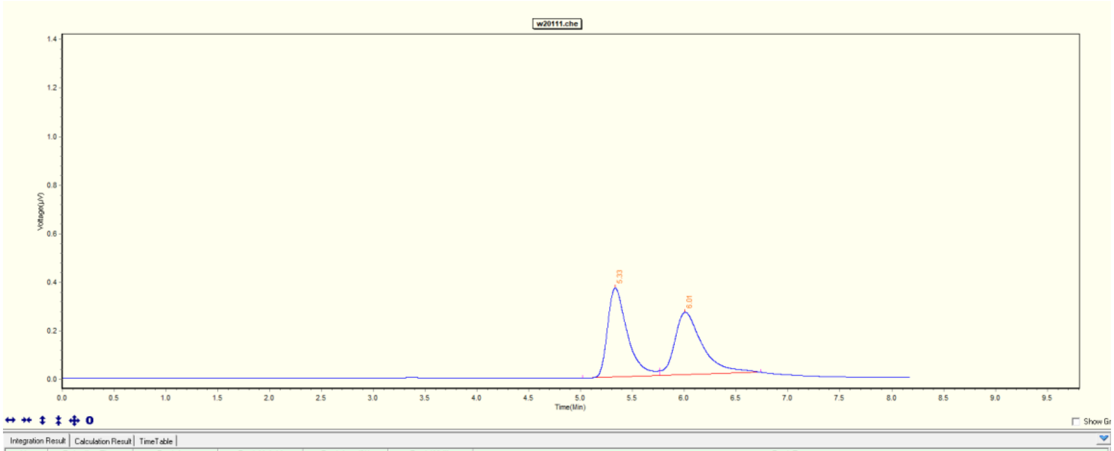

No.	Retention Time	Peak Area	Peak Height	Peak Area(%)	Peak Width	Peak Type
1	5.33	2421418	182793	50.41%	0.743	BV
2	6.01	2381773	128371	49.59%	0.973	VB
Total		4,803,191	311,164	100.00%		

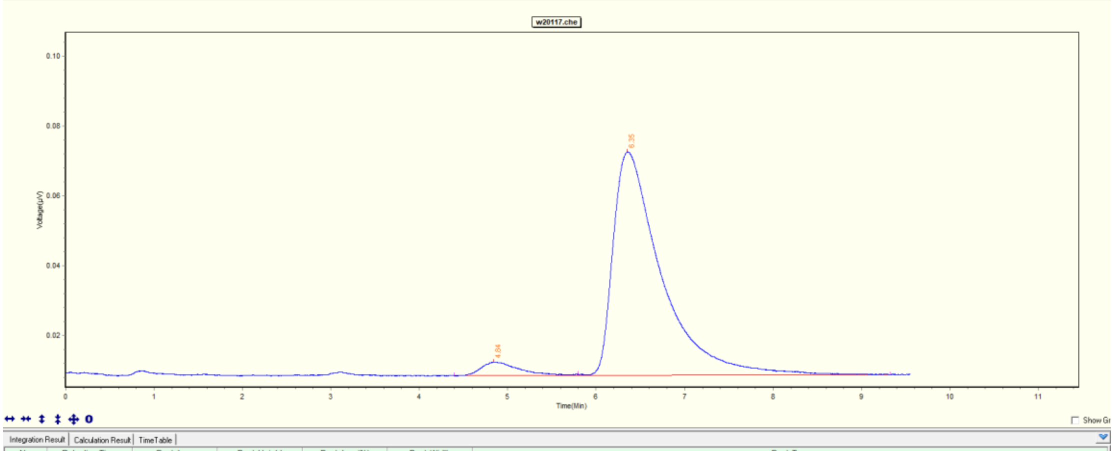

No.	Retention Time	Peak Area	Peak Height	Peak Area(%)	Peak Width	Peak Type
1	4.84	58267	1941	4.58%	1.399	BV
2	6.35	1214866	32014	95.42%	3.529	VB
Total		1,273,133	33,955	100.00%		

Supplementary Figure 190. HPLC spectrum of compound **3y**

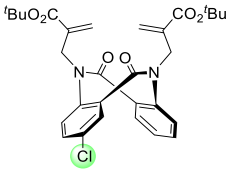

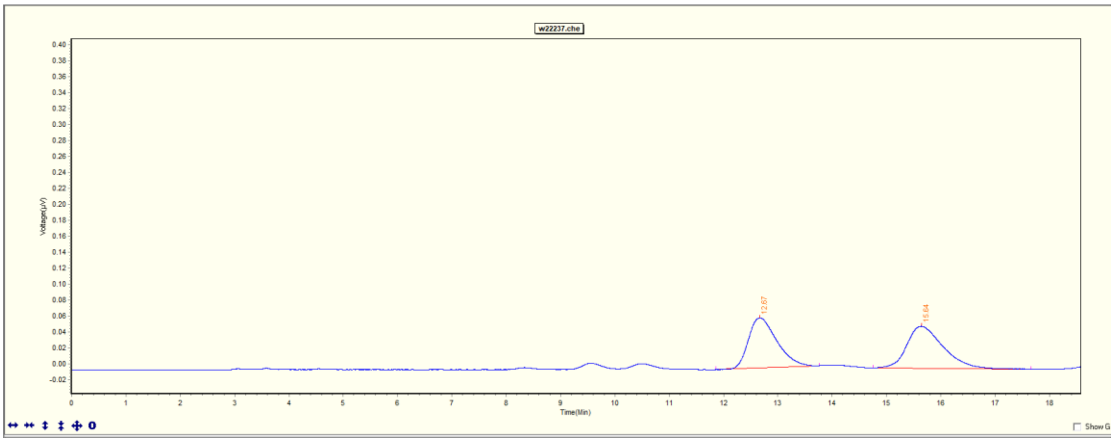

Integration Result | Calculation Result | TimeTable

No.	Retention Time	Peak Area	Peak Height	Peak Area(%)	Peak Width	Peak Type
1	12.67	1129844	31397	48.05%	1.901	BB
2	15.64	1221596	26299	51.95%	2.912	BB
Total		2,351,440	57,696	100.00%		

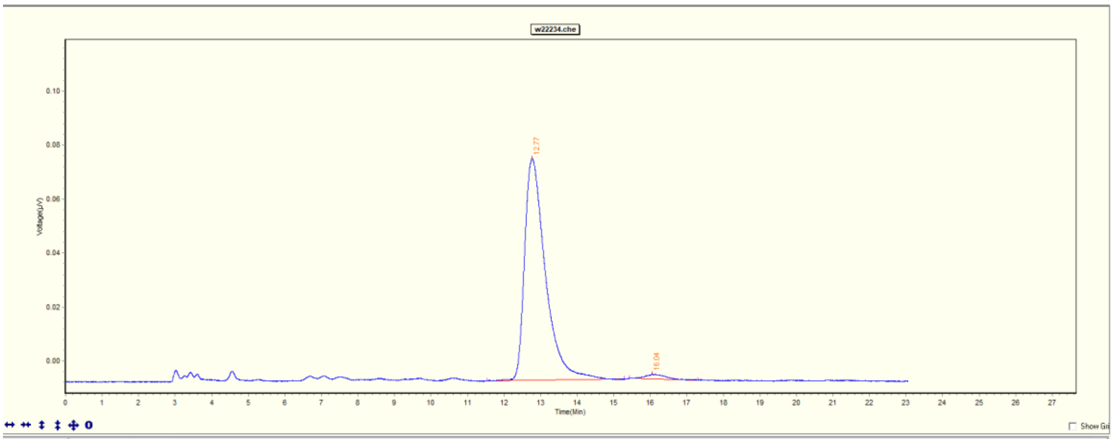

Integration Result | Calculation Result | TimeTable

No.	Retention Time	Peak Area	Peak Height	Peak Area(%)	Peak Width	Peak Type
1	12.77	1619943	40978	97.93%	3.764	BB
2	16.04	34230	864	2.07%	1.855	BB
Total		1,654,173	41,842	100.00%		

Supplementary Figure 191. HPLC spectrum of compound **3z**

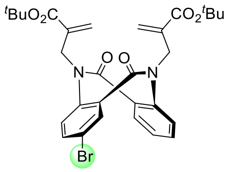

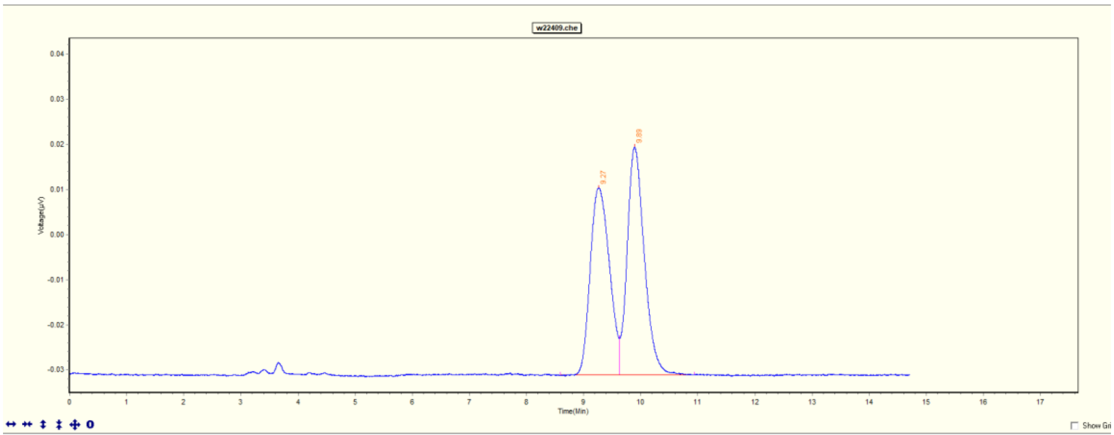

No.	Retention Time	Peak Area	Peak Height	Peak Area(%)	Peak Width	Peak Type
1	9.27	484858	20754	47.82%	1.033	BV
2	9.89	529162	25261	52.18%	1.313	VB
Total		1,014,020	46,015	100.00%		

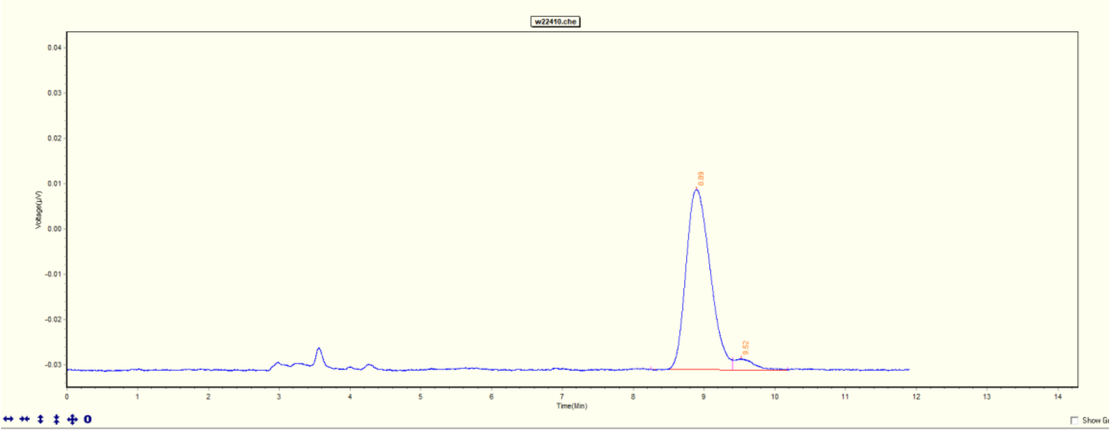

No.	Retention Time	Peak Area	Peak Height	Peak Area(%)	Peak Width	Peak Type
1	8.89	471962	19899	95.04%	1.155	BV
2	9.52	24640	1213	4.96%	0.779	VB
Total		496,602	21,112	100.00%		

Supplementary Figure 192. HPLC spectrum of compound **3aa**

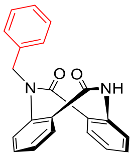

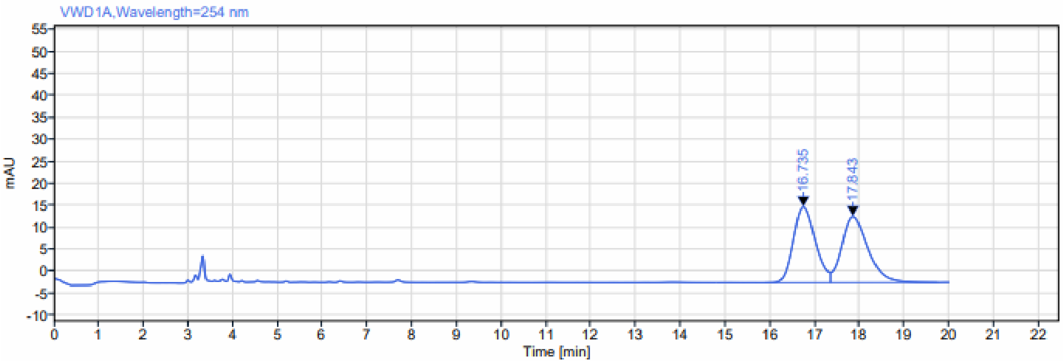

Signal: VWD1A,Wavelength=254 nm

RT [min]	Type	Width [min]	Area	Height	Area%	Name
16.735	BV	1.44	588.55	17.21	49.00	
17.843	VB	2.40	612.56	15.00	51.00	
		**Sum**	**1201.12**			

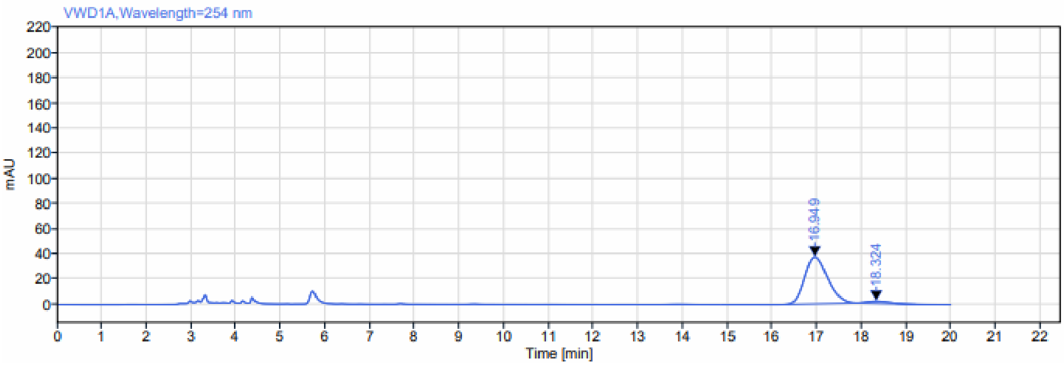

Signal: VWD1A,Wavelength=254 nm

RT [min]	Type	Width [min]	Area	Height	Area%	Name
16.949	BB	1.67	1340.13	37.24	95.62	
18.324	BBA	1.31	61.32	1.74	4.38	
		**Sum**	**1401.45**			

Supplementary Figure 193. HPLC spectrum of compound **4a**

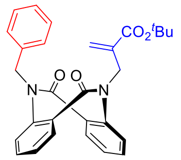

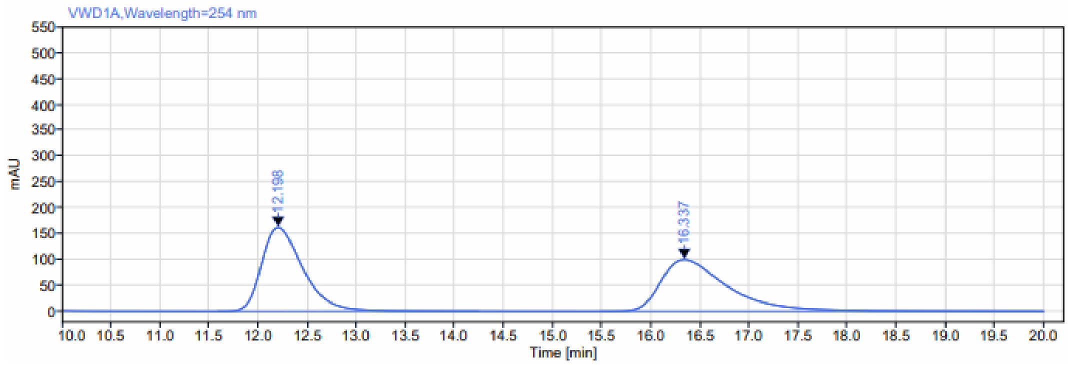

Signal: VWD1A,Wavelength=254 nm

RT [min]	Type	Width [min]	Area	Height	Area%	Name
12.198	BB	2.67	4762.50	162.72	50.00	
16.337	BBA	4.52	4762.71	100.24	50.00	
		**Sum**	**9525.21**			

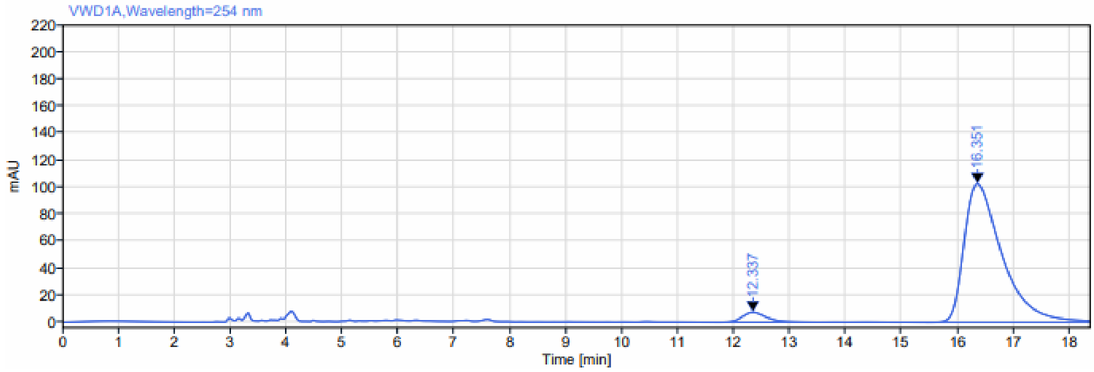

Signal: VWD1A,Wavelength=254 nm

RT [min]	Type	Width [min]	Area	Height	Area%	Name
12.337	BB	1.88	221.15	7.36	4.33	
16.351	BBA	4.56	4882.19	102.47	95.67	
		**Sum**	**5103.33**			

Supplementary Figure 194. HPLC spectrum of compound **5a**

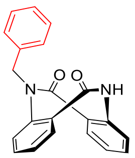

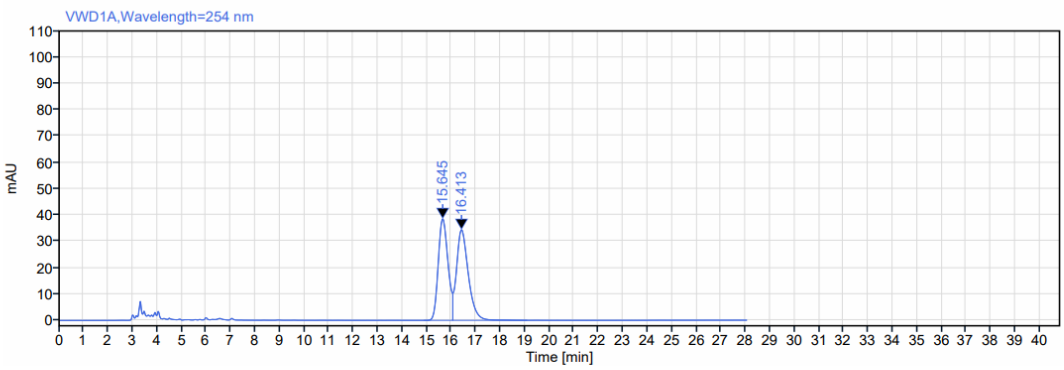

Signal: VWD1A,Wavelength=254 nm

RT [min]	Type	Width [min]	Area	Height	Area%	Name
15.645	BV	1.18	1067.34	38.53	48.04	
16.413	VB	3.03	1154.40	34.40	51.96	
		**Sum**	**2221.73**			

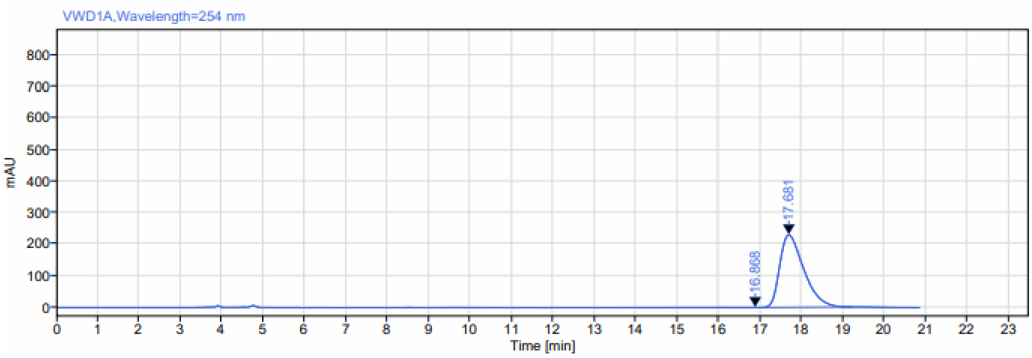

Signal: VWD1A,Wavelength=254 nm

RT [min]	Type	Width [min]	Area	Height	Area%	Name
16.868	MM n	1.03	1.47	0.04	0.02	
17.681	BB	2.26	9256.33	228.79	99.98	
		**Sum**	**9257.80**			

Supplementary Figure 195. HPLC spectrum of compound **4a**

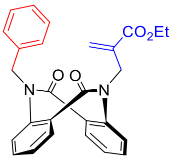

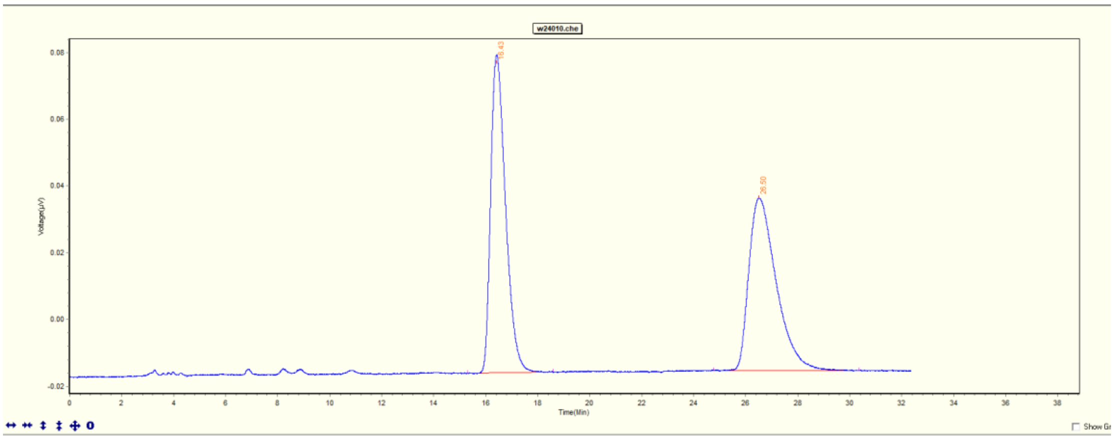

Integration Result | Calculation Result | TimeTable

No.	Retention Time	Peak Area	Peak Height	Peak Area(%)	Peak Width	Peak Type
1	16.43	1892466	47599	50.12%	3.294	BB
2	26.50	1883172	25800	49.88%	5.593	BB
Total		3,775,638	73,399	100.00%		

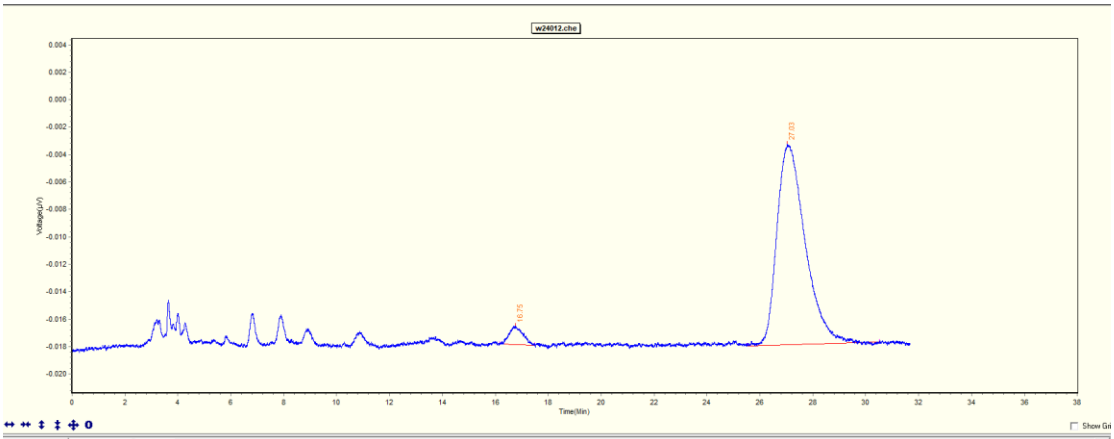

Integration Result | Calculation Result | TimeTable

No.	Retention Time	Peak Area	Peak Height	Peak Area(%)	Peak Width	Peak Type
1	16.75	23924	672	4.25%	1.33	BB
2	27.03	539290	7317	95.75%	4.994	BB
Total		563,214	7,989	100.00%		

Supplementary Figure 196. HPLC spectrum of compound **5b**

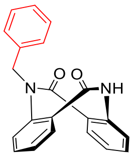

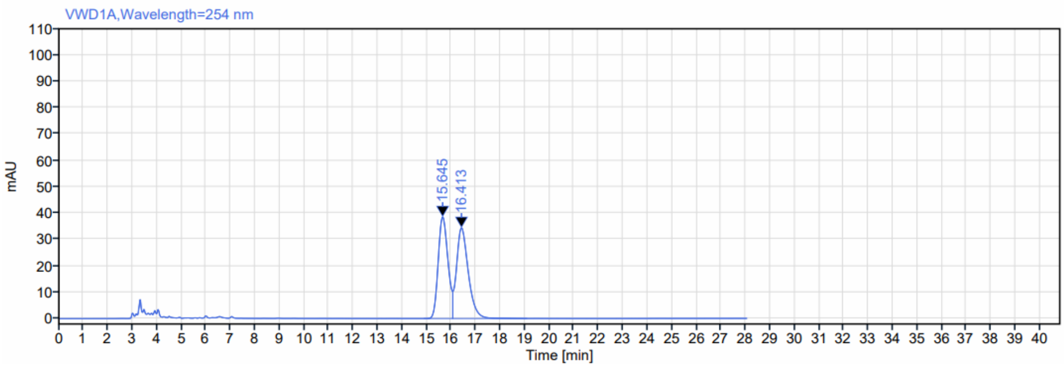

Signal: VWD1A,Wavelength=254 nm

RT [min]	Type	Width [min]	Area	Height	Area%	Name
15.645	BV	1.18	1067.34	38.53	48.04	
16.413	VB	3.03	1154.40	34.40	51.96	
		**Sum**	**2221.73**			

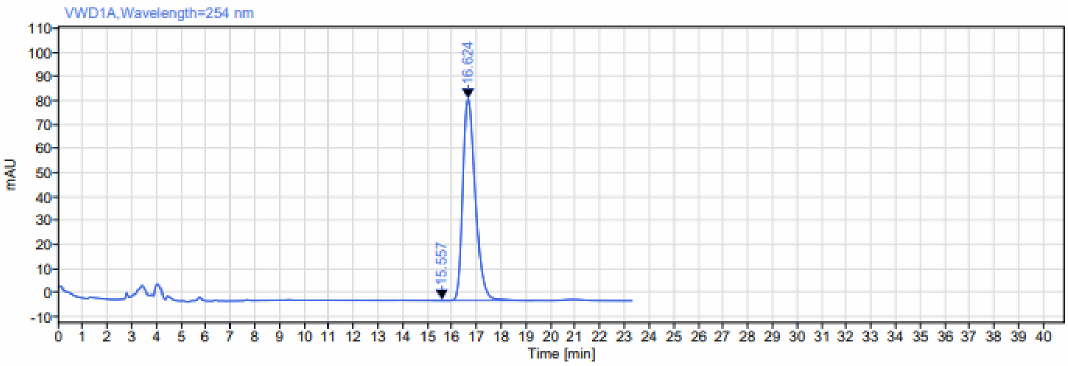

Signal: VWD1A,Wavelength=254 nm

RT [min]	Type	Width [min]	Area	Height	Area%	Name
15.557	MM n	0.61	0.15	0.01	0.00	
16.624	BB	3.51	2952.36	83.80	100.00	
		**Sum**	**2952.50**			

Supplementary Figure 197. HPLC spectrum of compound **4a**

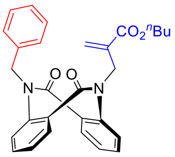

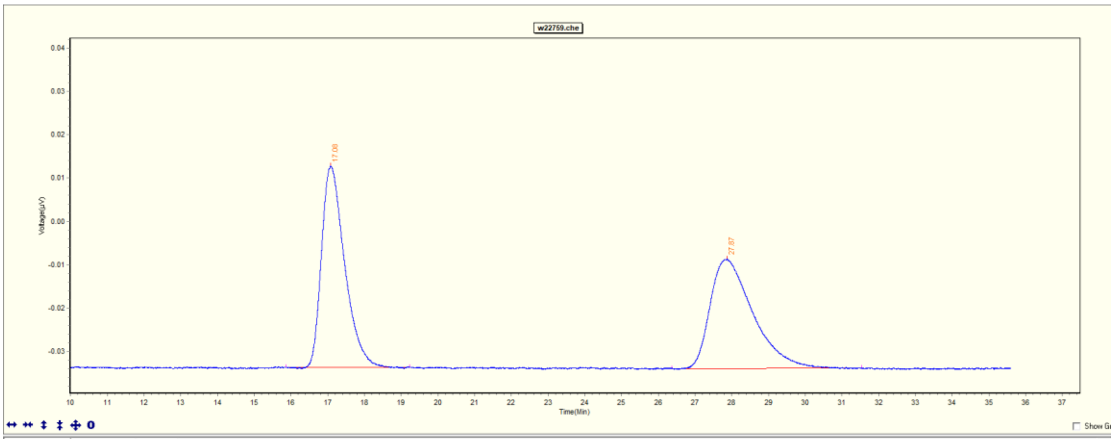

No.	Retention Time	Peak Area	Peak Height	Peak Area(%)	Peak Width	Peak Type
1	17.08	1012276	23182	49.87%	3.369	BB
2	27.87	1017492	12603	50.13%	5.166	BB
Total		2,029,768	35,785	100.00%		

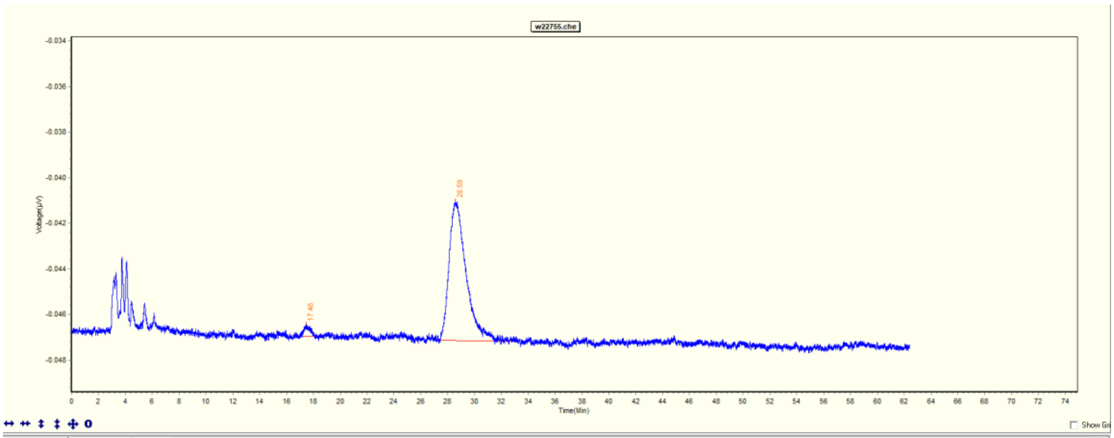

No.	Retention Time	Peak Area	Peak Height	Peak Area(%)	Peak Width	Peak Type
1	17.46	7653	252	2.73%	2.126	BB
2	28.59	272620	3040	97.27%	6.377	BB
Total		280,273	3,292	100.00%		

Supplementary Figure 198. HPLC spectrum of compound **5c**

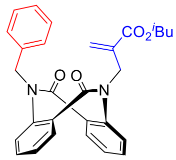

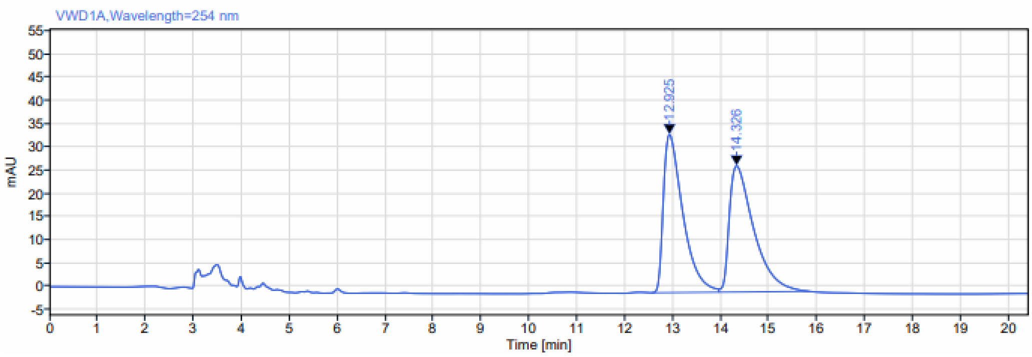

Signal: VWD1A,Wavelength=254 nm

RT [min]	Type	Width [min]	Area	Height	Area%	Name
12.925	BM m	1.40	967.78	34.01	49.93	
14.326	MM m	1.87	970.55	27.23	50.07	
		**Sum**	**1938.33**			

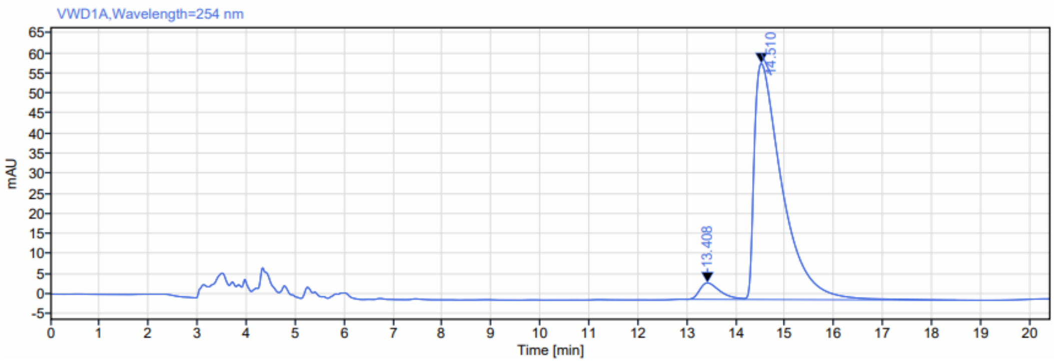

Signal: VWD1A,Wavelength=254 nm

RT [min]	Type	Width [min]	Area	Height	Area%	Name
13.408	BV	1.18	118.25	4.10	4.83	
14.510	VB	4.34	2330.95	58.89	95.17	
		**Sum**	**2449.20**			

Supplementary Figure 199. HPLC spectrum of compound **5d**

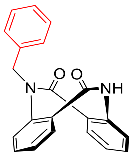

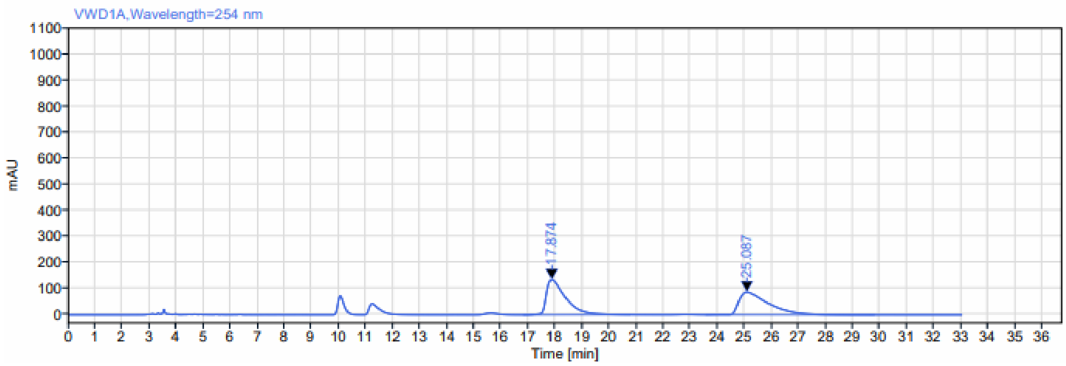

Signal: VWD1A,Wavelength=254 nm

RT [min]	Type	Width [min]	Area	Height	Area%	Name
17.874	BM m	3.97	6660.95	135.16	50.14	
25.087	BB	6.25	6624.94	87.09	49.86	
		**Sum**	**13285.89**			

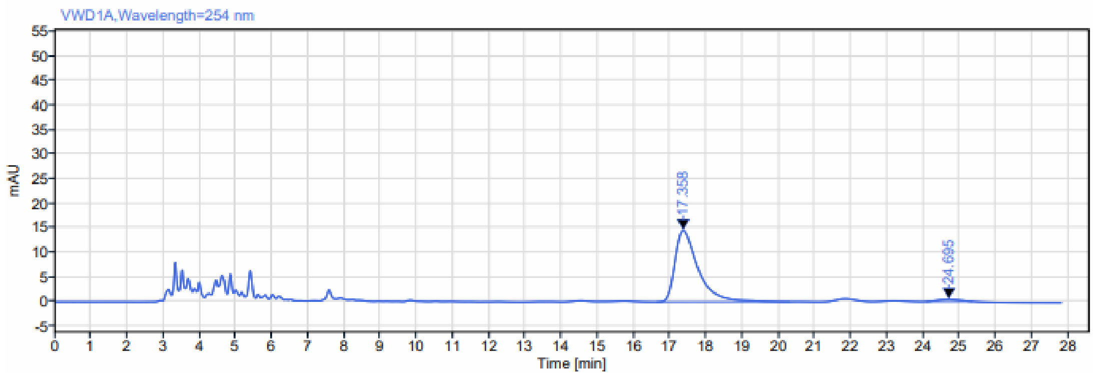

Signal: VWD1A,Wavelength=254 nm

RT [min]	Type	Width [min]	Area	Height	Area%	Name
17.358	BB	3.69	649.16	14.57	97.13	
24.695	MM m	1.29	19.16	0.45	2.87	
		**Sum**	**668.31**			

Supplementary Figure 200. HPLC spectrum of compound **4a**

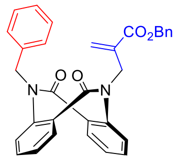

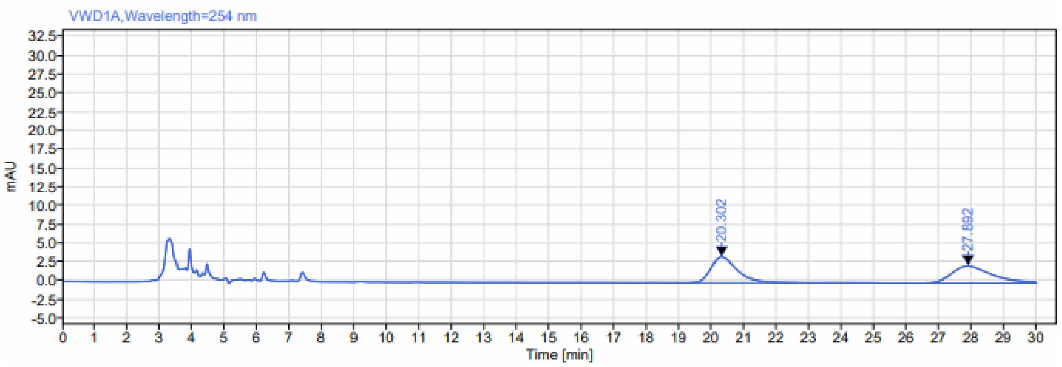

Signal: VWD1A,Wavelength=254 nm

RT [min]	Type	Width [min]	Area	Height	Area%	Name
20.302	BB	3.40	191.41	3.42	49.77	
27.892	BBA	3.56	193.21	2.26	50.23	
		**Sum**	**384.62**			

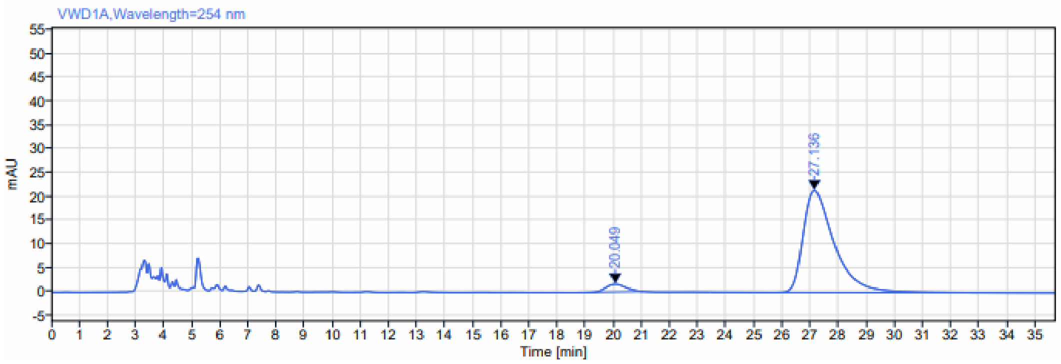

Signal: VWD1A,Wavelength=254 nm

RT [min]	Type	Width [min]	Area	Height	Area%	Name
20.049	BM m	2.09	81.07	1.64	4.68	
27.136	BB	7.13	1651.87	21.40	95.32	
		**Sum**	**1732.93**			

Supplementary Figure 201. HPLC spectrum of compound **5e**

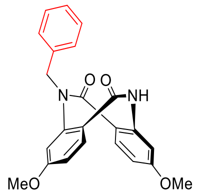

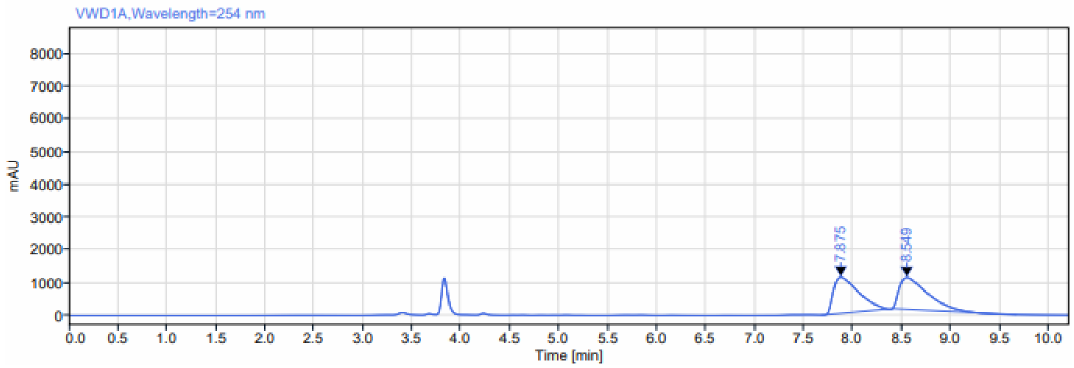

Signal: VWD1A,Wavelength=254 nm

RT [min]	Type	Width [min]	Area	Height	Area%	Name
7.875	MM m	0.73	19591.53	1107.53	49.91	
8.549	MM m	1.27	19664.05	971.07	50.09	
		**Sum**	**39255.57**			

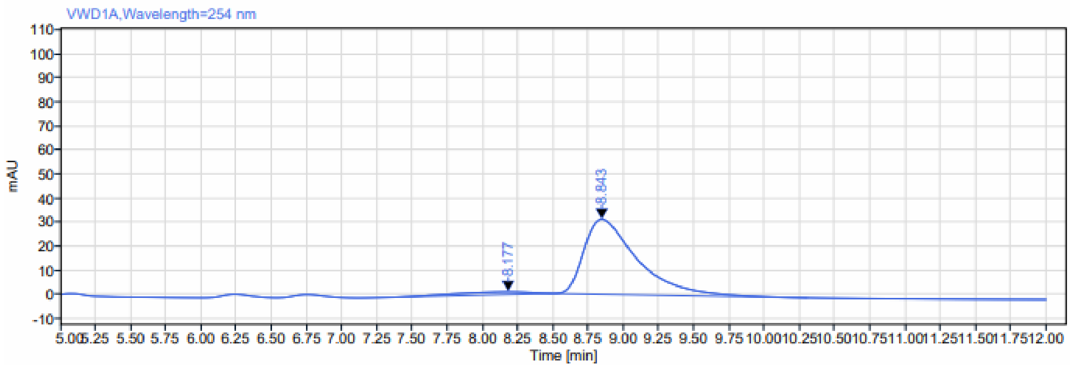

Signal: VWD1A,Wavelength=254 nm

RT [min]	Type	Width [min]	Area	Height	Area%	Name
8.177	MM m	1.13	45.86	1.13	5.28	
8.843	BBA	1.81	822.34	31.14	94.72	
		**Sum**	**868.20**			

Supplementary Figure 202. HPLC spectrum of compound **4f**

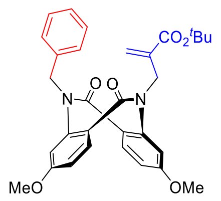

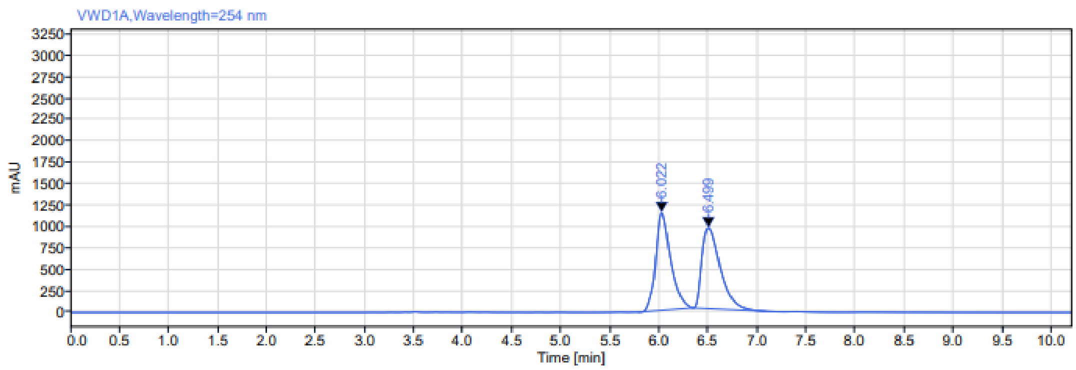

Signal: VWD1A,Wavelength=254 nm

RT [min]	Type	Width [min]	Area	Height	Area%	Name
6.022	MM m	0.56	12142.26	1138.73	49.52	
6.499	MM m	0.75	12375.40	940.81	50.48	
		**Sum**	**24517.66**			

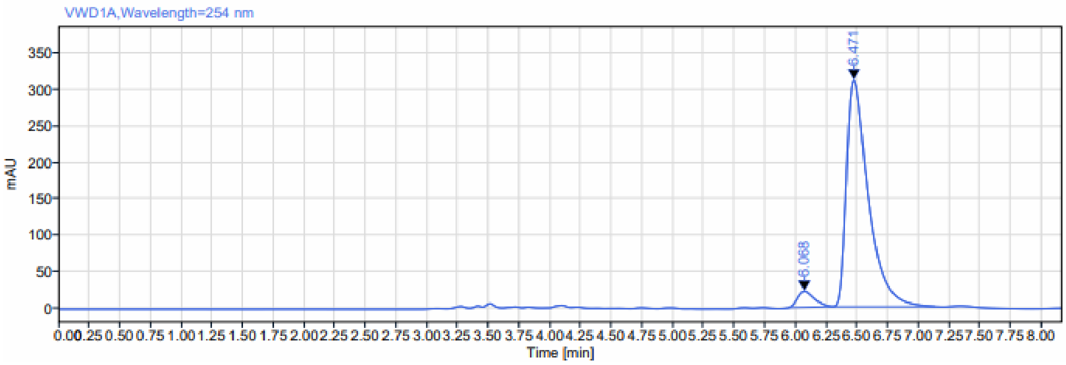

Signal: VWD1A,Wavelength=254 nm

RT [min]	Type	Width [min]	Area	Height	Area%	Name
6.068	MM m	0.36	208.73	21.92	5.17	
6.471	MM m	0.84	3826.22	309.50	94.83	
		**Sum**	**4034.95**			

Supplementary Figure 203. HPLC spectrum of compound **5f**

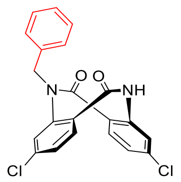

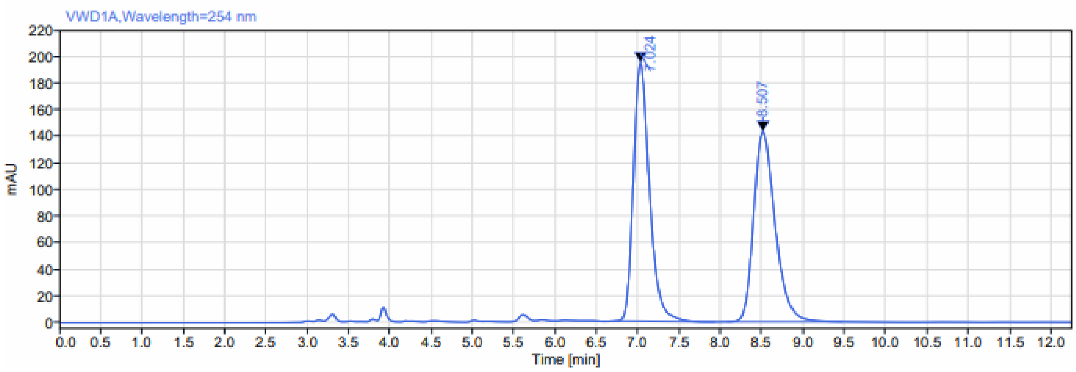

Signal: VWD1A,Wavelength=254 nm

RT [min]	Type	Width [min]	Area	Height	Area%	Name
7.024	MM m	1.20	2637.60	194.19	50.53	
8.507	MM m	1.39	2582.64	142.83	49.47	
		**Sum**	**5220.24**			

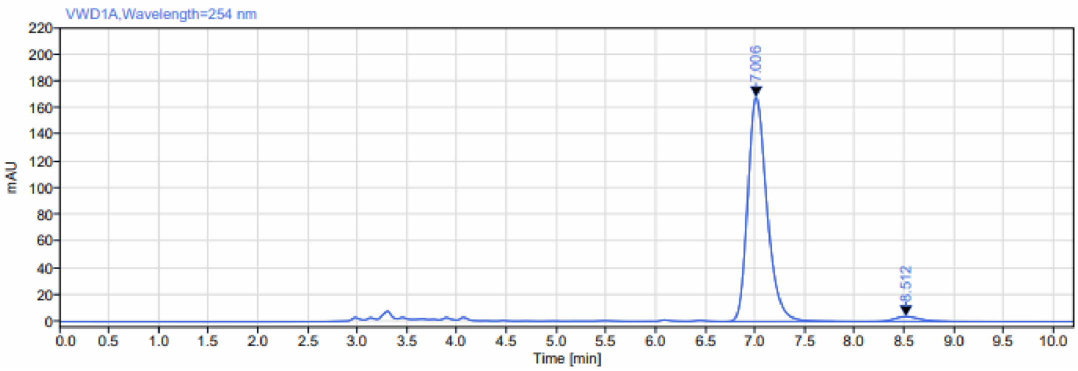

Signal: VWD1A,Wavelength=254 nm

RT [min]	Type	Width [min]	Area	Height	Area%	Name
7.006	MM m	1.11	2259.01	167.87	97.21	
8.512	MB m	0.81	64.78	3.61	2.79	
		**Sum**	**2323.80**			

Supplementary Figure 204. HPLC spectrum of compound **4g**

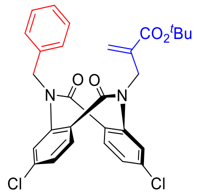

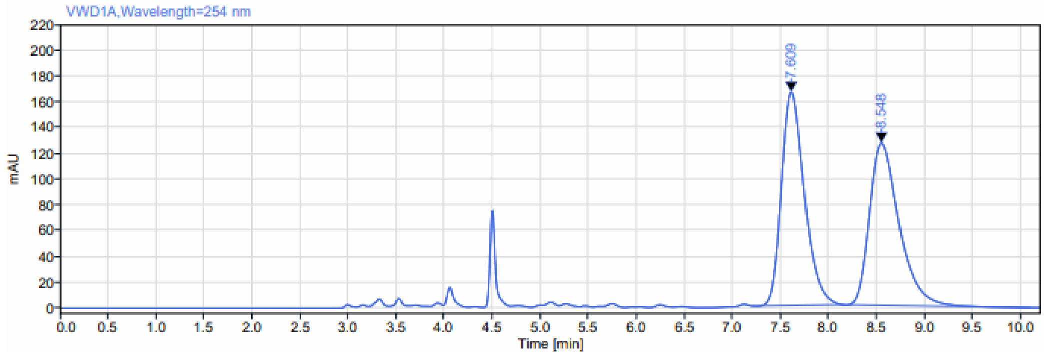

Signal: VWD1A,Wavelength=254 nm

RT [min]	Type	Width [min]	Area	Height	Area%	Name
7.609	MM m	0.93	2758.50	165.39	50.44	
8.548	MM m	1.97	2709.86	125.84	49.56	
		**Sum**	**5468.36**			

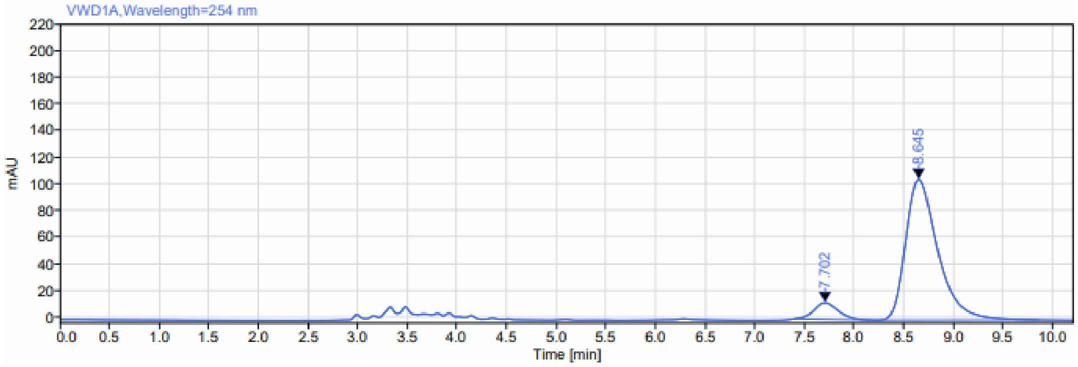

Signal: VWD1A,Wavelength=254 nm

RT [min]	Type	Width [min]	Area	Height	Area%	Name
7.702	MM m	0.79	214.98	12.49	8.26	
8.645	MB m	1.68	2386.27	105.47	91.74	
		**Sum**	**2601.25**			

Supplementary Figure 205. HPLC spectrum of compound **5g**

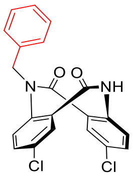

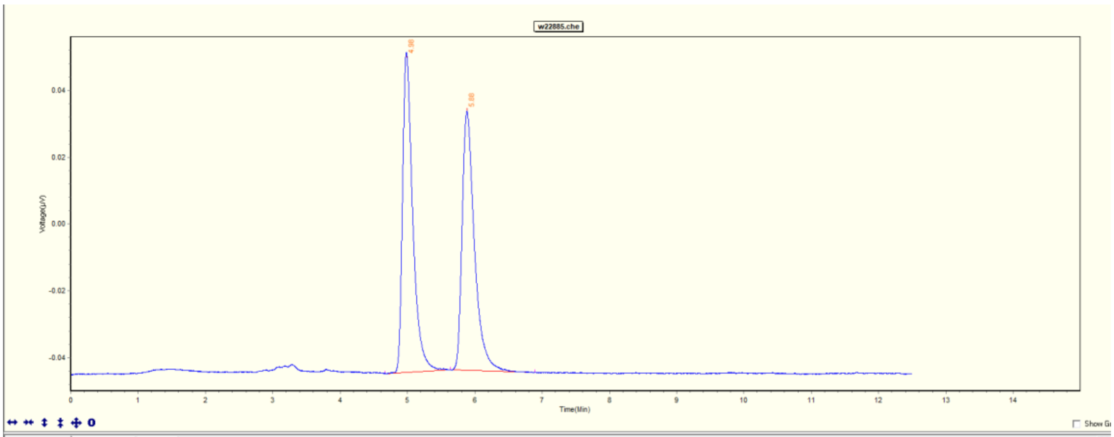

Integration Result | Calculation Result | TimeTable

No.	Retention Time	Peak Area	Peak Height	Peak Area(%)	Peak Width	Peak Type
1	5.88	497363	38887	49.61%	1.252	BB
2	4.98	505114	47878	50.39%	0.977	BB
Total		1,002,477	86,765	100.00%		

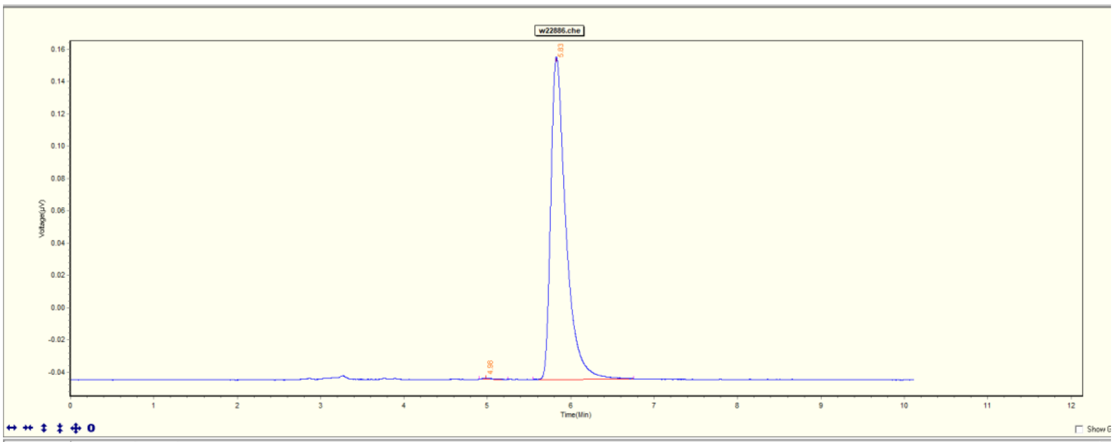

Integration Result | Calculation Result | TimeTable

No.	Retention Time	Peak Area	Peak Height	Peak Area(%)	Peak Width	Peak Type
1	4.98	2827	355	0.23%	0.343	BB
2	5.83	1250551	99820	99.77%	1.205	BB
Total		1,253,378	100,175	100.00%		

Supplementary Figure 206. HPLC spectrum of compound **4h**

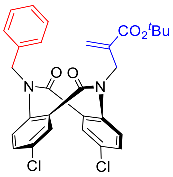

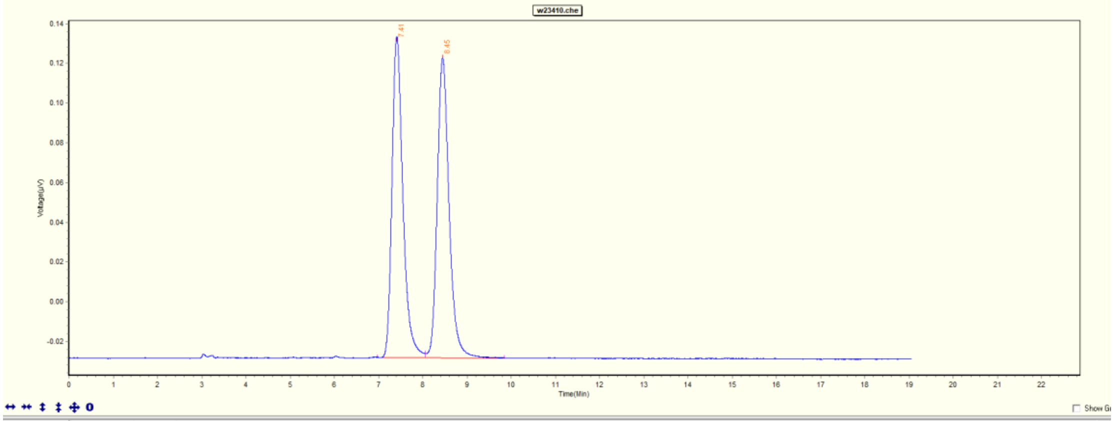

No.	Retention Time	Peak Area	Peak Height	Peak Area(%)	Peak Width	Peak Type
1	7.41	1385863	80695	49.56%	1.083	BV
2	8.45	1410213	75430	50.44%	1.788	VB
Total		2,796,076	156,125	100.00%		

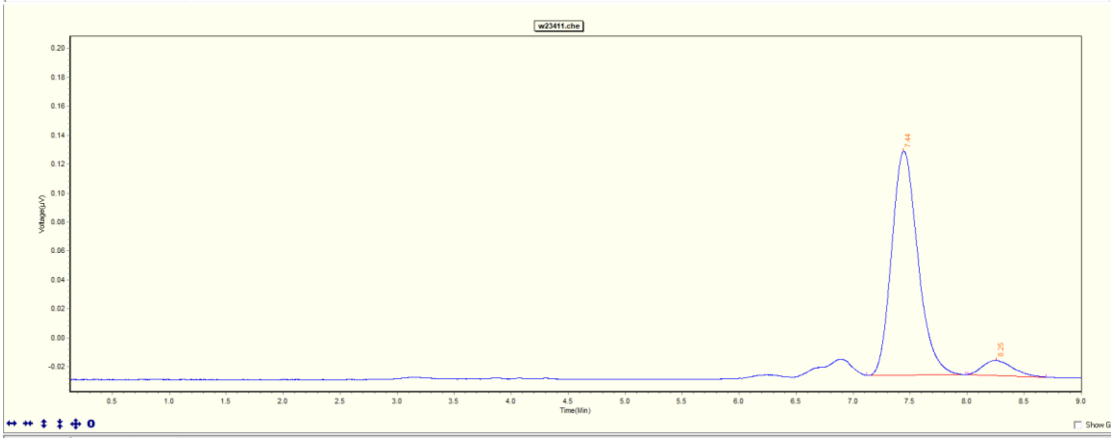

No.	Retention Time	Peak Area	Peak Height	Peak Area(%)	Peak Width	Peak Type
1	7.44	1233497	77343	93.05%	0.868	BB
2	8.25	92067	5101	6.95%	0.688	BB
Total		1,325,564	82,444	100.00%		

Supplementary Figure 207. HPLC spectrum of compound **5h**

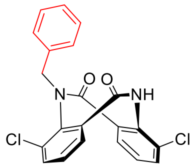

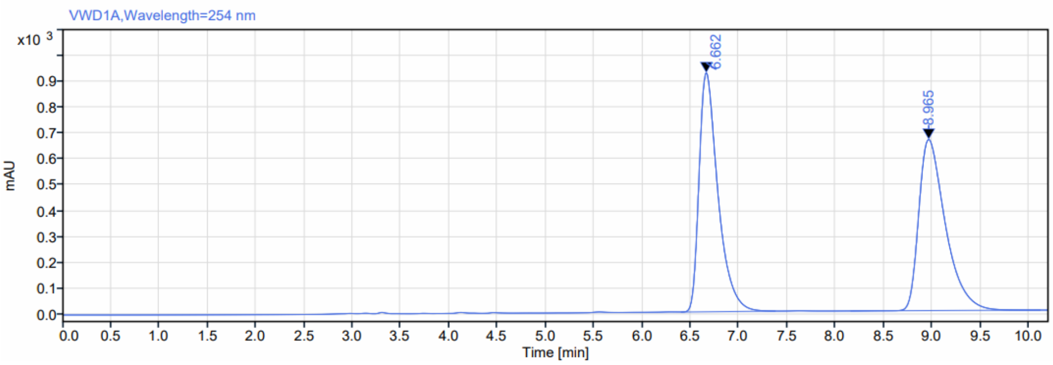

Signal: VWD1A,Wavelength=254 nm

RT [min]	Type	Width [min]	Area	Height	Area%	Name
6.662	VM m	0.98	12527.15	921.09	49.77	
8.965	BB	1.97	12641.70	659.58	50.23	
		**Sum**	**25168.85**			

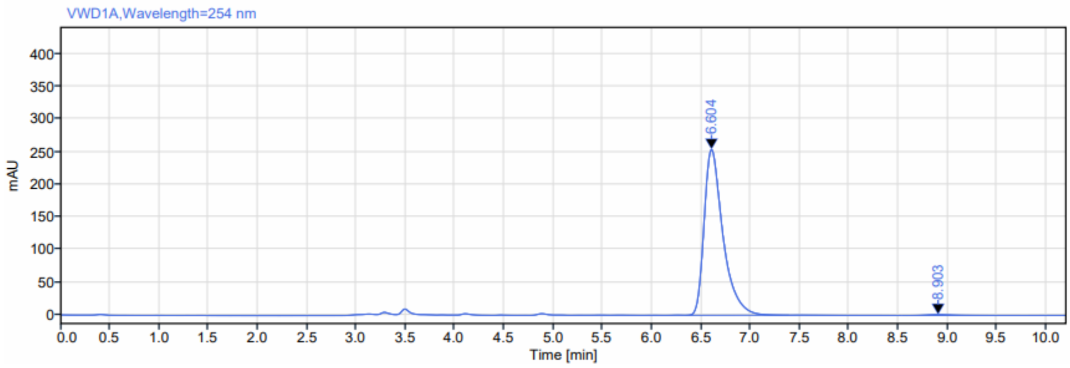

Signal: VWD1A,Wavelength=254 nm

RT [min]	Type	Width [min]	Area	Height	Area%	Name
6.604	VB	1.05	3381.50	253.92	99.31	
8.903	BB	1.19	23.40	1.22	0.69	
		**Sum**	**3404.90**			

Supplementary Figure 208. HPLC spectrum of compound **4i**

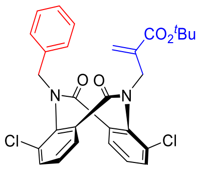

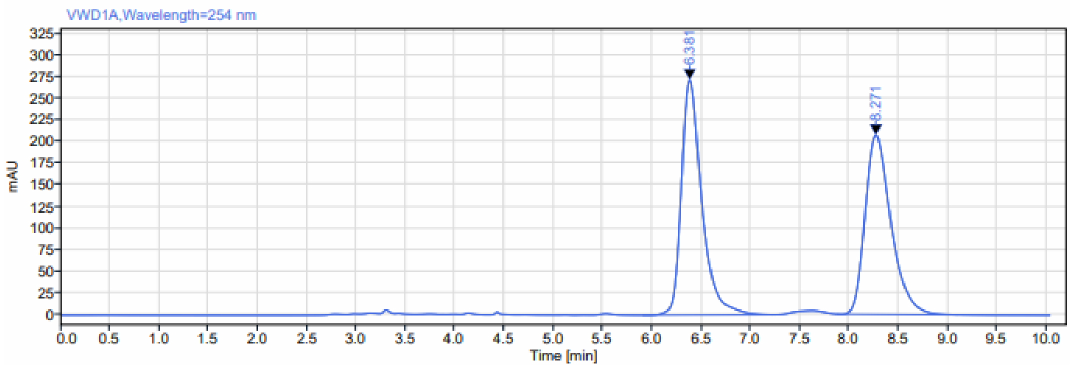

Signal: VWD1A,Wavelength=254 nm

RT [min]	Type	Width [min]	Area	Height	Area%	Name
6.381	BM m	1.33	3901.25	271.11	50.83	
8.271	MM m	1.24	3774.34	206.70	49.17	
		**Sum**	**7675.59**			

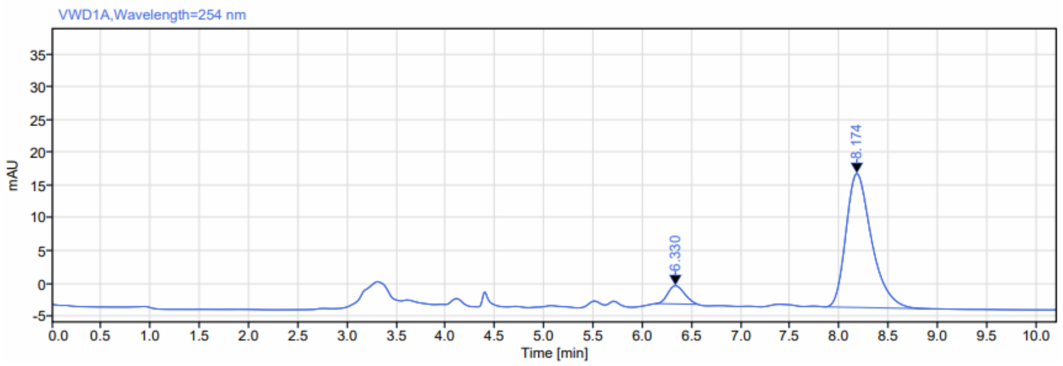

Signal: VWD1A,Wavelength=254 nm

RT [min]	Type	Width [min]	Area	Height	Area%	Name
6.330	MM m	0.43	32.73	2.77	8.12	
8.174	BB	1.08	370.27	20.44	91.88	
		**Sum**	**403.00**			

Supplementary Figure 209. HPLC spectrum of compound **5i**

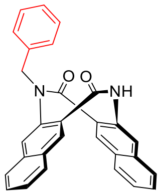

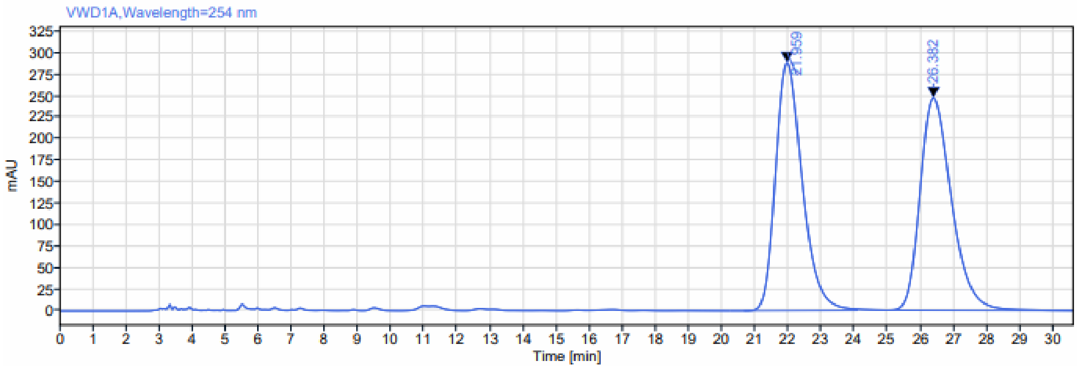

Signal: VWD1A,Wavelength=254 nm

RT [min]	Type	Width [min]	Area	Height	Area%	Name
21.959	BM m	3.42	16209.88	288.13	49.89	
26.382	BB	7.22	16280.83	246.78	50.11	
		**Sum**	**32490.71**			

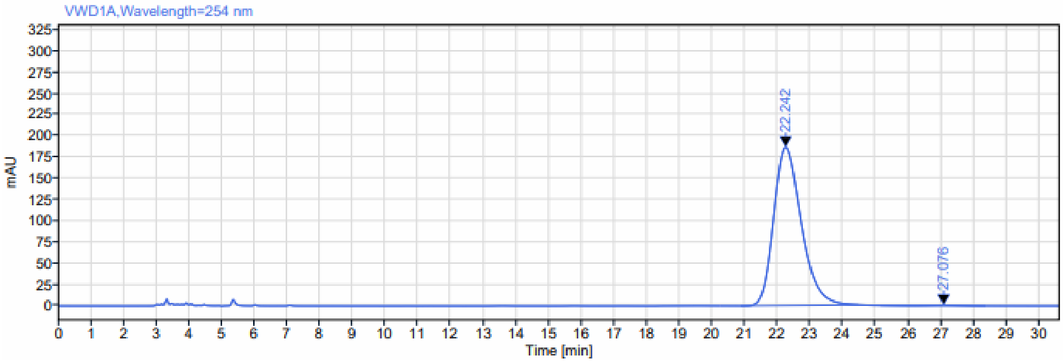

Signal: VWD1A,Wavelength=254 nm

RT [min]	Type	Width [min]	Area	Height	Area%	Name
22.242	BM m	3.60	10913.47	186.18	99.92	
27.076	MM m	3.22	8.72	0.35	0.08	
		**Sum**	**10922.19**			

Supplementary Figure 210. HPLC spectrum of compound **4j**

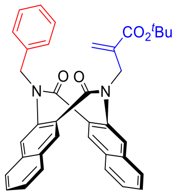

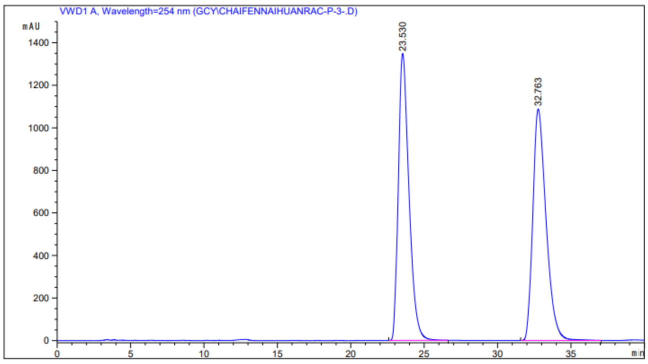


```
=====================================================================
                      Area Percent Report
=====================================================================

Sorted By             :      Signal
Multiplier            :      1.0000
Dilution              :      1.0000
Use Multiplier & Dilution Factor with ISTDs


Signal 1: VWD1 A, Wavelength=254 nm

Peak RetTime Type  Width     Area      Height     Area
  #   [min]        [min]  mAU    *s   [mAU   ]     %
----|-------|----|-------|----------|----------|--------|
   1  23.530 BB    0.7544 6.76942e4 1348.86633  49.7922
   2  32.763 BB    0.9413 6.82593e4 1088.43420  50.2078

Totals :                  1.35953e5  2437.30054
```

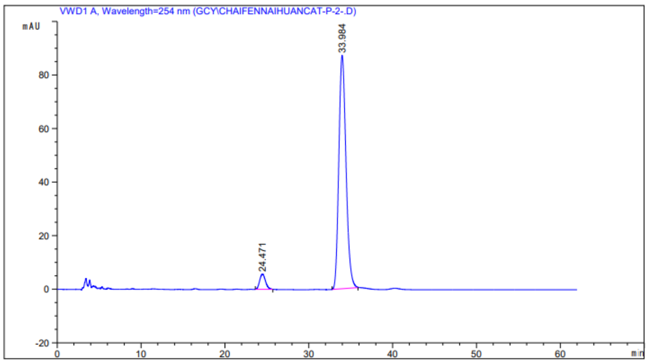


```
=====================================================================
                      Area Percent Report
=====================================================================

Sorted By             :      Signal
Multiplier            :      1.0000
Dilution              :      1.0000
Use Multiplier & Dilution Factor with ISTDs


Signal 1: VWD1 A, Wavelength=254 nm

Peak RetTime Type  Width     Area      Height     Area
  #   [min]        [min]  mAU    *s   [mAU   ]     %
----|-------|----|-------|----------|----------|--------|
   1  24.471 BB    0.7353  275.29550    5.71415   5.0432
   2  33.984 BB    0.9149 5183.43799   87.19252  94.9568

Totals :                  5458.73349   92.90667
```

Supplementary Figure 211. HPLC spectrum of compound **5j**

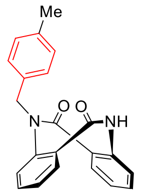

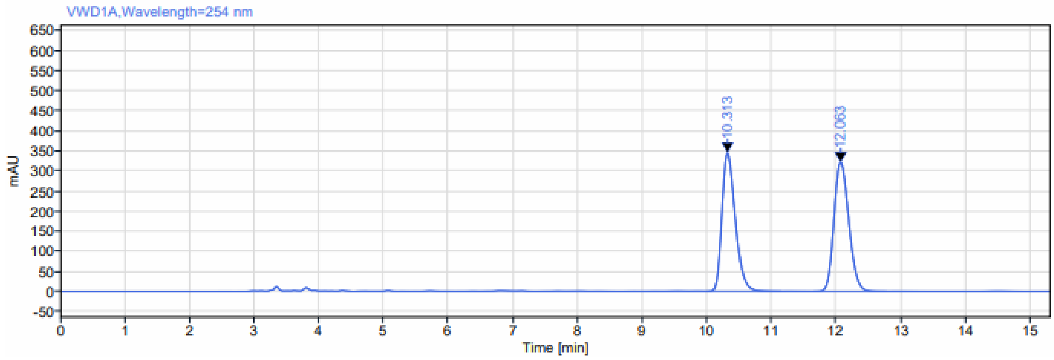

Signal: VWD1A,Wavelength=254 nm

RT [min]	Type	Width [min]	Area	Height	Area%	Name
10.313	BB	1.15	5009.95	345.03	49.43	
12.063	BB	1.13	5124.50	322.02	50.57	
		**Sum**	**10134.45**			

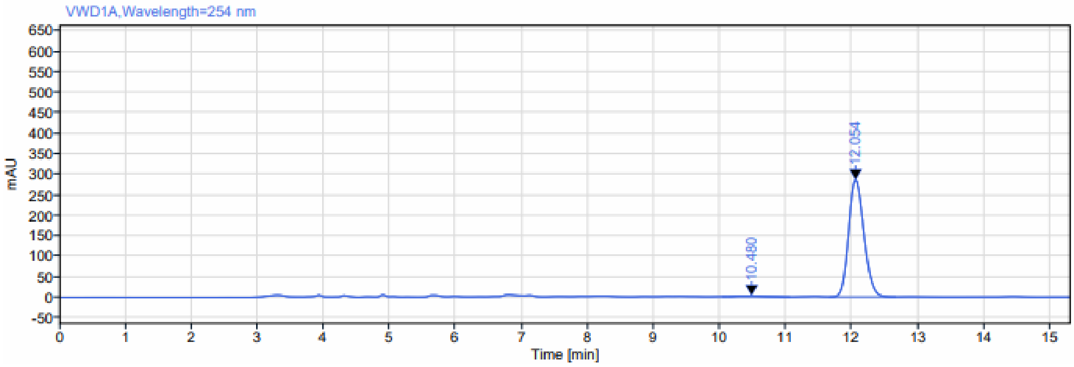

Signal: VWD1A,Wavelength=254 nm

RT [min]	Type	Width [min]	Area	Height	Area%	Name
10.480	MB m	1.04	55.70	1.79	1.19	
12.054	VB	1.01	4611.35	285.36	98.81	
		**Sum**	**4667.05**			

Supplementary Figure 212. HPLC spectrum of compound **4k**

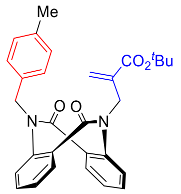

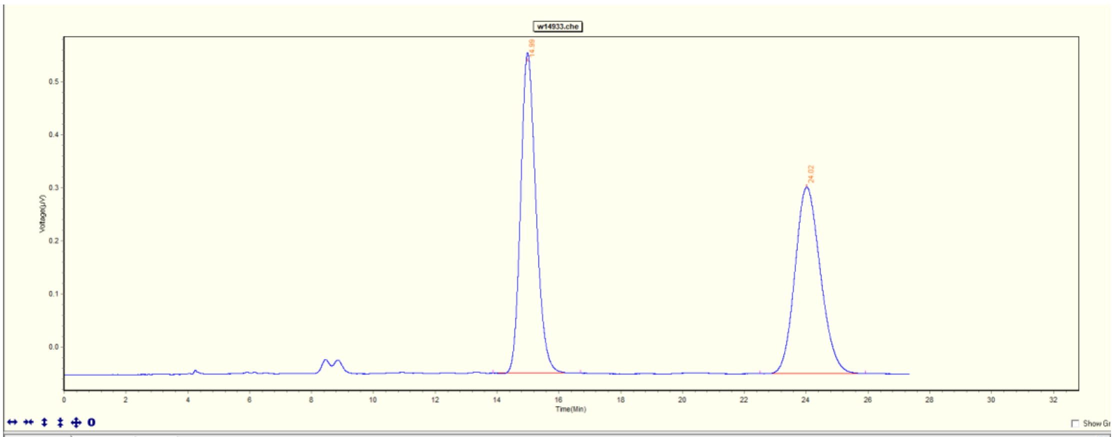

Integration Result | Calculation Result | TimeTable

No.	Retention Time	Peak Area	Peak Height	Peak Area(%)	Peak Width	Peak Type
1	14.99	10376837	301927	50.78%	2.83	BB
2	24.02	10057501	175786	49.22%	3.408	BB
Total		20,434,338	477,713	100.00%		

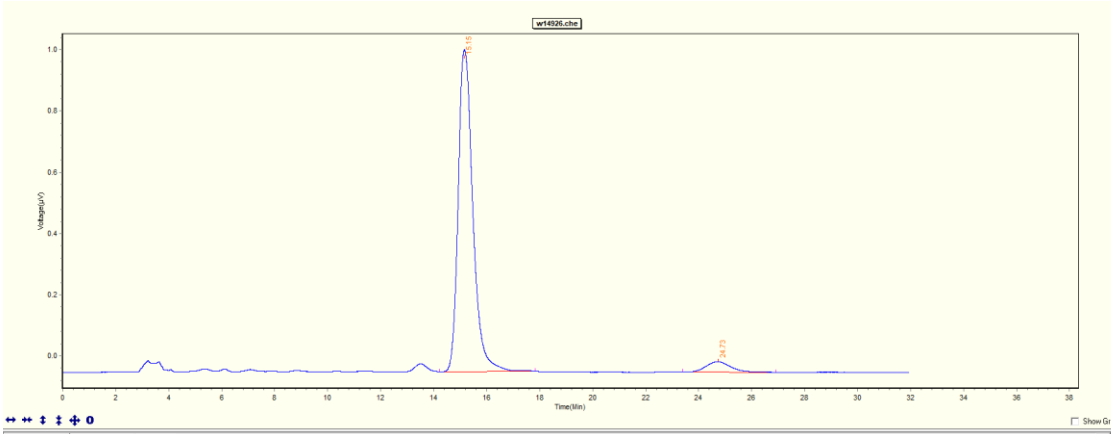

Integration Result | Calculation Result | TimeTable

No.	Retention Time	Peak Area	Peak Height	Peak Area(%)	Peak Width	Peak Type
1	15.15	20176767	525126	94.93%	3.607	BB
2	24.73	1077592	16962	5.07%	3.531	BB
Total		21,254,359	542,088	100.00%		

Supplementary Figure 213. HPLC spectrum of compound **5k**

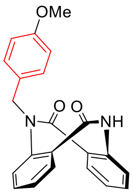

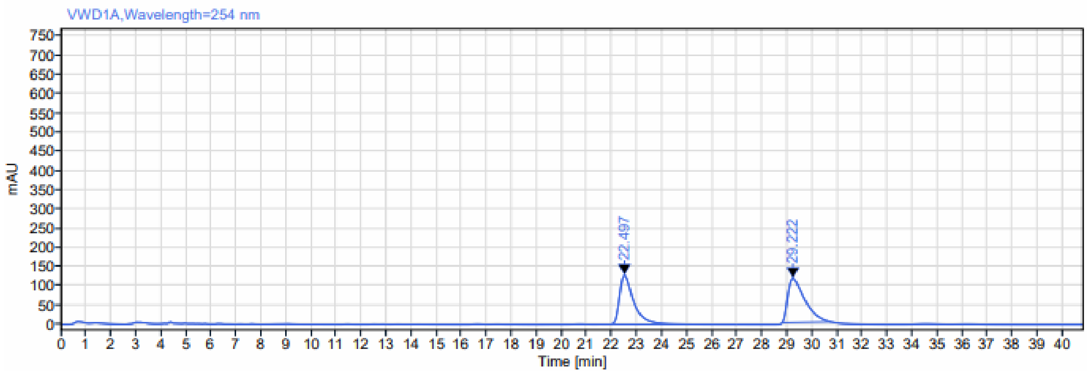

Signal: VWD1A,Wavelength=254 nm

RT [min]	Type	Width [min]	Area	Height	Area%	Name
22.497	MM m	3.59	5049.98	127.77	48.21	
29.222	MM m	1.94	5426.03	114.49	51.79	
		**Sum**	**10476.01**			

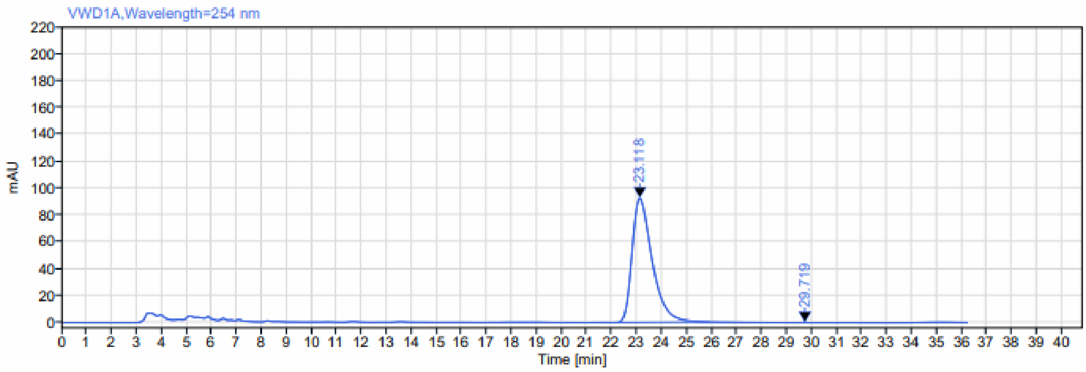

Signal: VWD1A,Wavelength=254 nm

RT [min]	Type	Width [min]	Area	Height	Area%	Name
23.118	BM m	3.84	5169.85	92.37	100.00	
29.719	MM n	0.77	0.05	0.00	0.00	
		**Sum**	**5169.90**			

Supplementary Figure 214. HPLC spectrum of compound **4l**

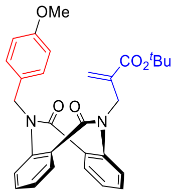

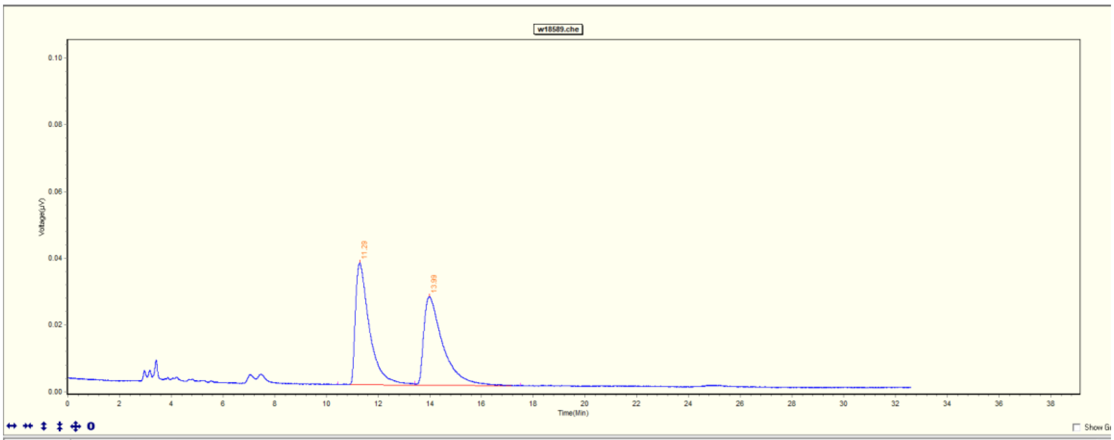

No.	Retention Time	Peak Area	Peak Height	Peak Area(%)	Peak Width	Peak Type
1	11.29	687248	18235	50.15%	2.988	BV
2	13.99	683223	13257	49.85%	4.07	VB
Total		1,370,471	31,492	100.00%		

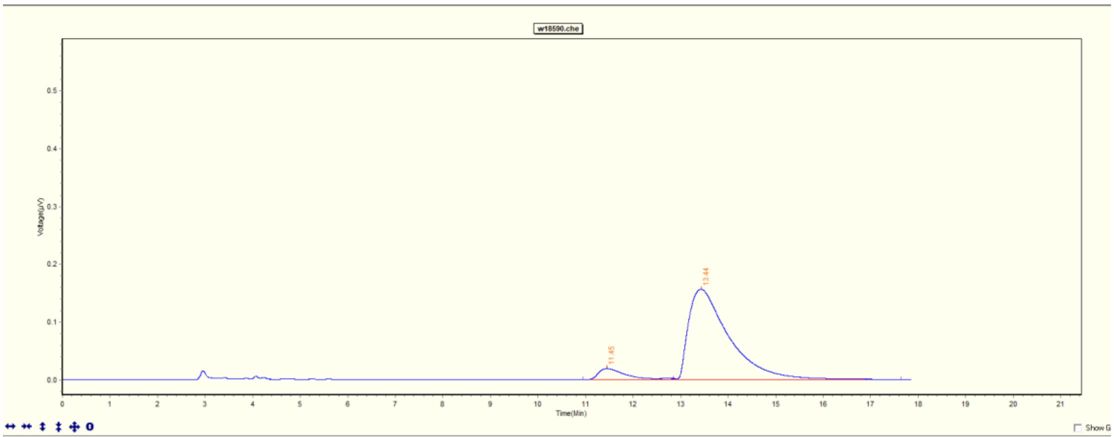

No.	Retention Time	Peak Area	Peak Height	Peak Area(%)	Peak Width	Peak Type
1	11.45	369625	9520	7.45%	1.903	BV
2	13.44	4594201	77808	92.55%	4.788	VB
Total		4,963,826	87,328	100.00%		

Supplementary Figure 215. HPLC spectrum of compound **5l**

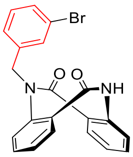

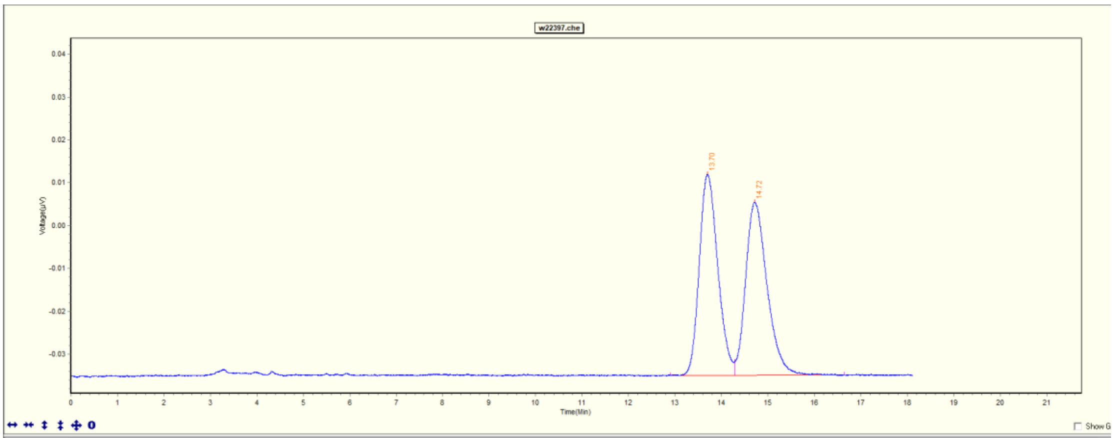

Integration Result | Calculation Result | TimeTable

No.	Retention Time	Peak Area	Peak Height	Peak Area(%)	Peak Width	Peak Type
1	13.70	650545	23455	50.12%	1.387	BV
2	14.72	647484	20164	49.88%	2.358	VB
Total		1,298,029	43,619	100.00%		

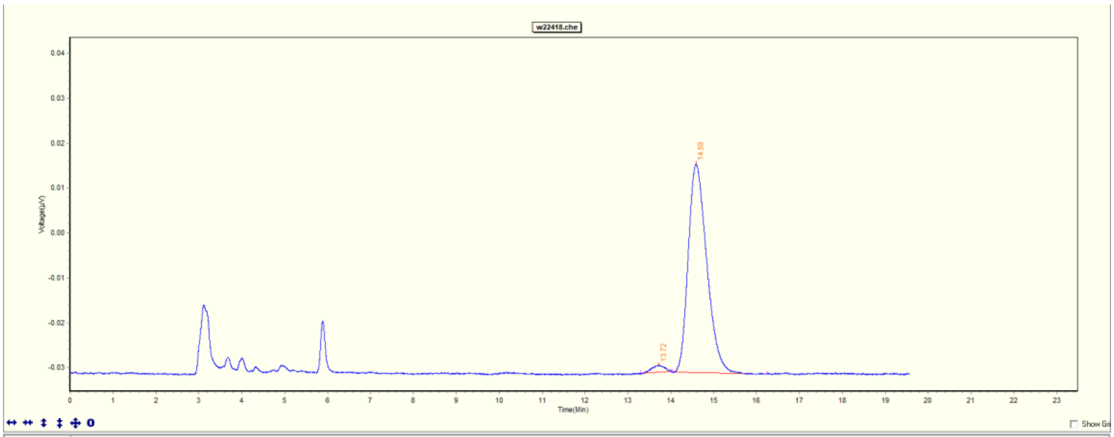

Integration Result | Calculation Result | TimeTable

No.	Retention Time	Peak Area	Peak Height	Peak Area(%)	Peak Width	Peak Type
1	13.72	16068	786	2.26%	0.71	BB
2	14.59	696075	23236	97.74%	2.208	BB
Total		712,143	24,022	100.00%		

Supplementary Figure 216. HPLC spectrum of compound **4m**

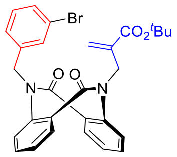

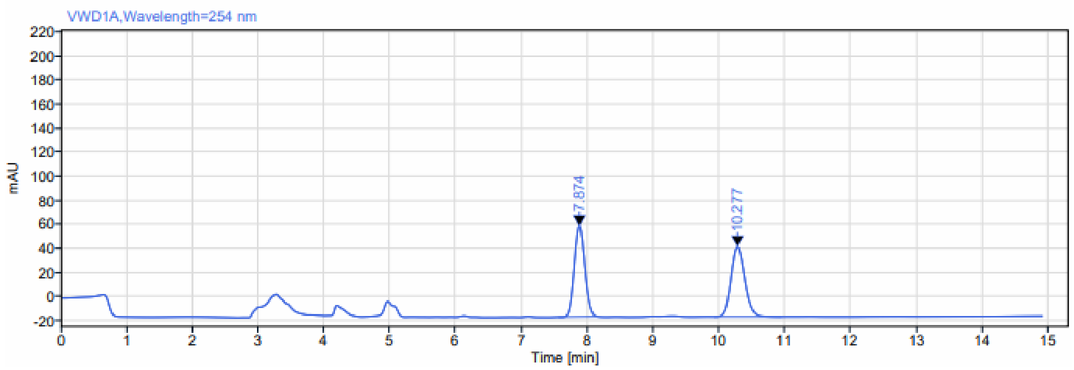

Signal: VWD1A,Wavelength=254 nm

RT [min]	Type	Width [min]	Area	Height	Area%	Name
7.874	MM m	0.60	836.91	75.33	49.63	
10.277	BB	1.03	849.24	58.23	50.37	
		**Sum**	**1686.15**			

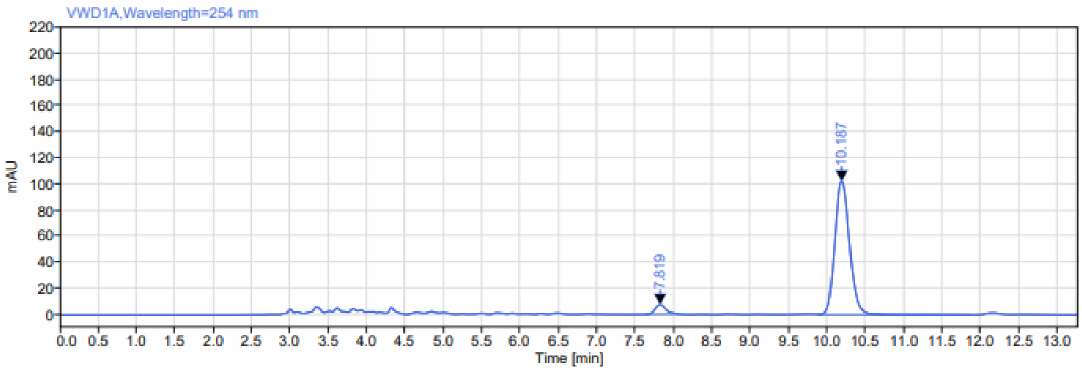

Signal: VWD1A,Wavelength=254 nm

RT [min]	Type	Width [min]	Area	Height	Area%	Name
7.819	MM m	0.44	70.95	7.30	4.97	
10.187	VB	1.98	1358.06	102.06	95.03	
		**Sum**	**1429.02**			

Supplementary Figure 217. HPLC spectrum of compound **5m**

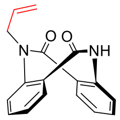

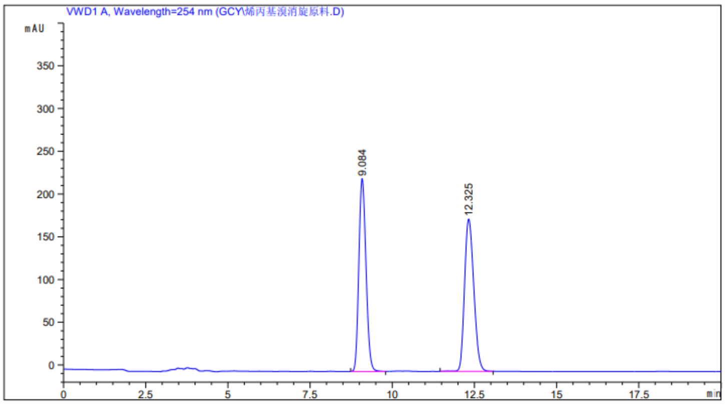


```
=====================================================================
                         Area Percent Report
=====================================================================

Sorted By             :      Signal
Multiplier            :      1.0000
Dilution              :      1.0000
Use Multiplier & Dilution Factor with ISTDs


Signal 1: VWD1 A, Wavelength=254 nm

Peak RetTime Type  Width     Area      Height     Area
  #   [min]        [min]  mAU    *s   [mAU   ]      %
----|-------|----|-------|----------|----------|--------|
   1   9.084 BB    0.2406 3444.19702  225.69106  49.7358
   2  12.325 BB    0.3065 3480.78784  178.20488  50.2642

Totals :                  6924.98486  403.89594
```

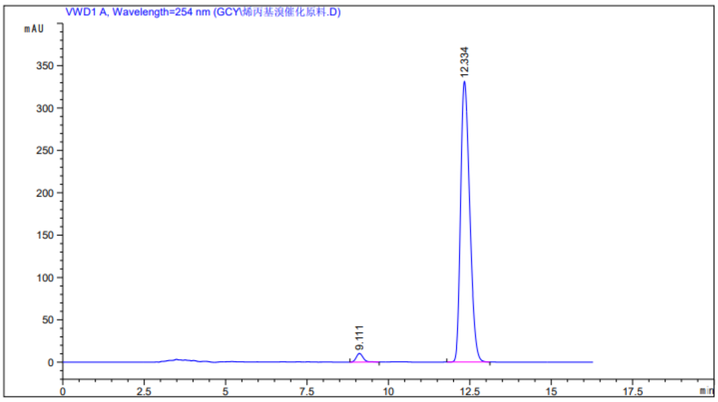


```
=====================================================================
                         Area Percent Report
=====================================================================

Sorted By             :      Signal
Multiplier            :      1.0000
Dilution              :      1.0000
Use Multiplier & Dilution Factor with ISTDs


Signal 1: VWD1 A, Wavelength=254 nm

Peak RetTime Type  Width     Area      Height     Area
  #   [min]        [min]  mAU    *s   [mAU   ]      %
----|-------|----|-------|----------|----------|--------|
   1   9.111 BB    0.2340  152.15306   10.18437   2.3113
   2  12.334 BB    0.3036 6430.96973  331.28662  97.6887

Totals :                  6583.12279  341.47099
```

Supplementary Figure 218. HPLC spectrum of compound **4n**

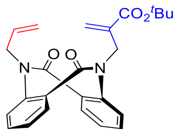

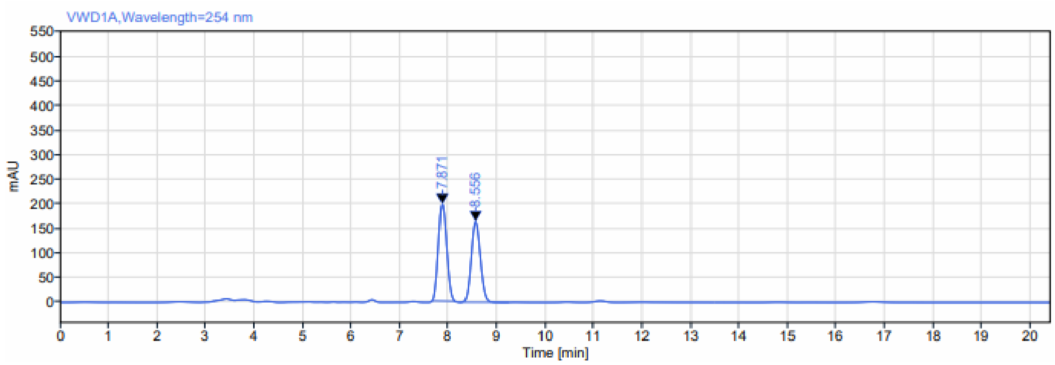

Signal: VWD1A,Wavelength=254 nm

RT [min]	Type	Width [min]	Area	Height	Area%	Name
7.871	MM m	0.47	2339.90	196.71	51.84	
8.556	MM m	1.03	2173.56	163.71	48.16	
		**Sum**	**4513.46**			

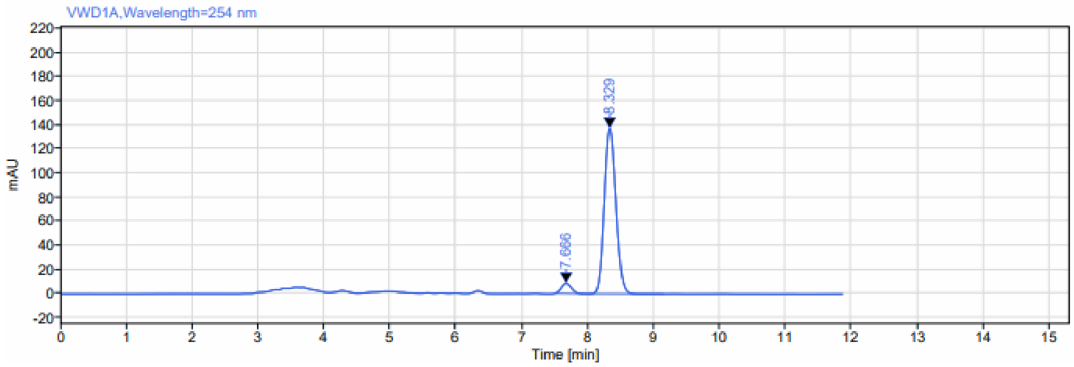

Signal: VWD1A,Wavelength=254 nm

RT [min]	Type	Width [min]	Area	Height	Area%	Name
7.666	MM m	0.44	88.71	8.29	5.02	
8.329	MM m	1.20	1677.71	137.36	94.98	
		**Sum**	**1766.42**			

Supplementary Figure 219. HPLC spectrum of compound **5n**

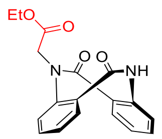

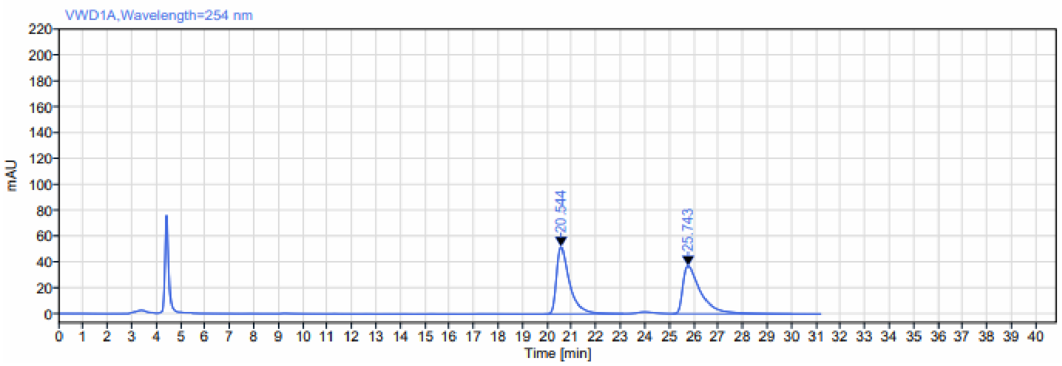

Signal:	VWD1A,Wavelength=254 nm					
RT [min]	Type	Width [min]	Area	Height	Area%	Name
20.544	BB	3.23	1917.00	51.57	50.64	
25.743	BB	4.92	1868.21	36.64	49.36	
		**Sum**	**3785.21**			

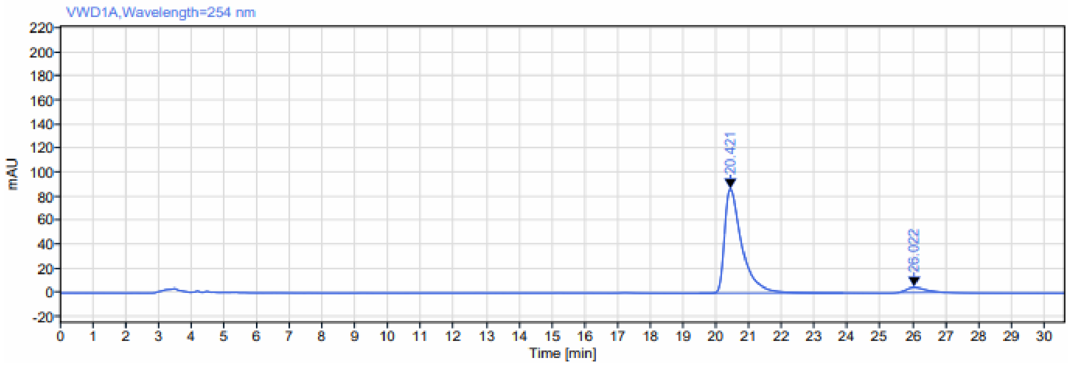

Signal:	VWD1A,Wavelength=254 nm					
RT [min]	Type	Width [min]	Area	Height	Area%	Name
20.421	MM m	4.38	3168.30	86.35	94.90	
26.022	MM m	1.45	170.39	4.17	5.10	
		**Sum**	**3338.68**			

Supplementary Figure 220. HPLC spectrum of compound **4o**

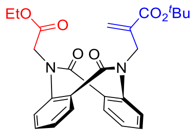

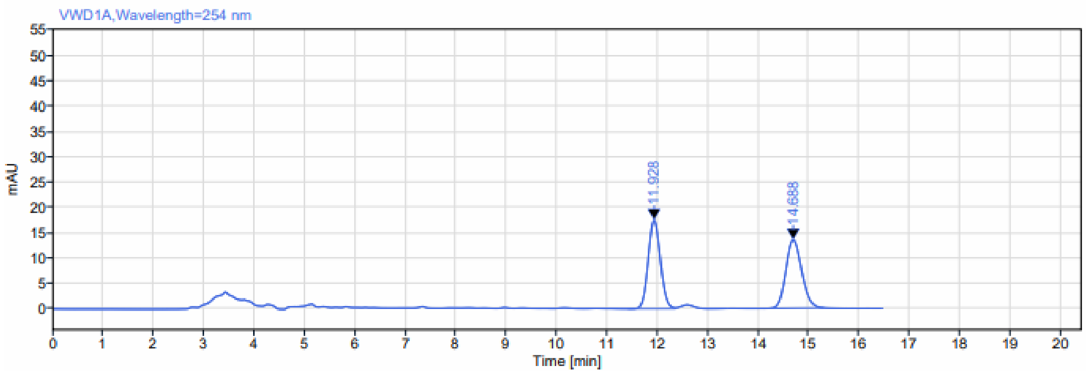

Signal: VWD1A,Wavelength=254 nm

RT [min]	Type	Width [min]	Area	Height	Area%	Name
11.928	BV	0.94	294.77	17.49	49.92	
14.688	BM m	1.20	295.75	13.50	50.08	
		**Sum**	**590.52**			

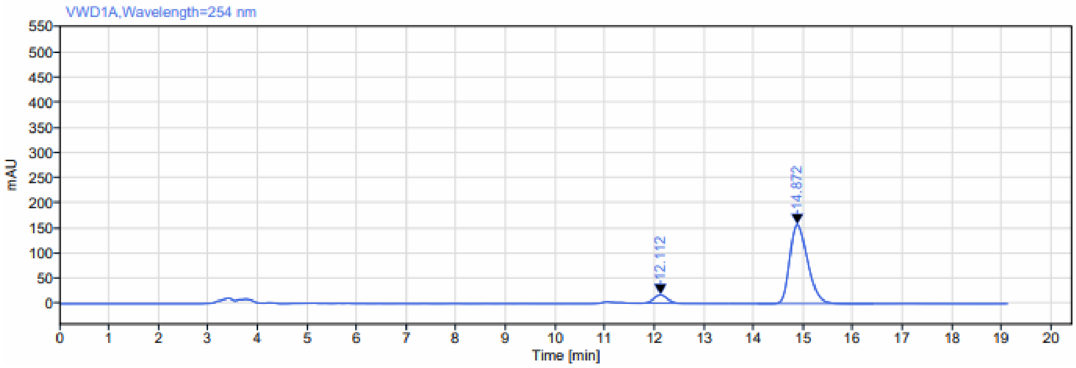

Signal: VWD1A,Wavelength=254 nm

RT [min]	Type	Width [min]	Area	Height	Area%	Name
12.112	MM m	0.70	297.63	16.05	7.05	
14.872	MM m	2.33	3927.00	156.63	92.95	
		**Sum**	**4224.62**			

Supplementary Figure 221. HPLC spectrum of compound **5o**

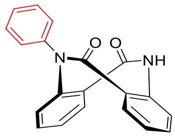

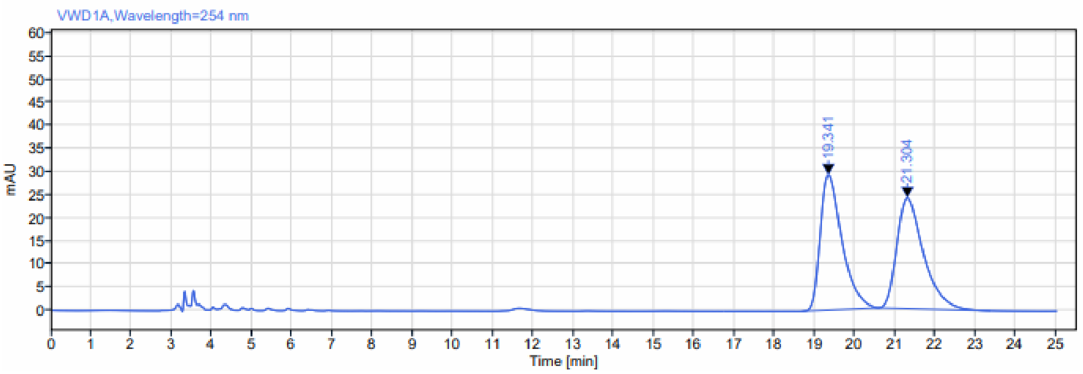

Signal: VWD1A,Wavelength=254 nm

RT [min]	Type	Width [min]	Area	Height	Area%	Name
19.341	BM m	1.90	1100.18	29.24	49.83	
21.304	BB	2.78	1107.68	23.90	50.17	
		**Sum**	**2207.86**			

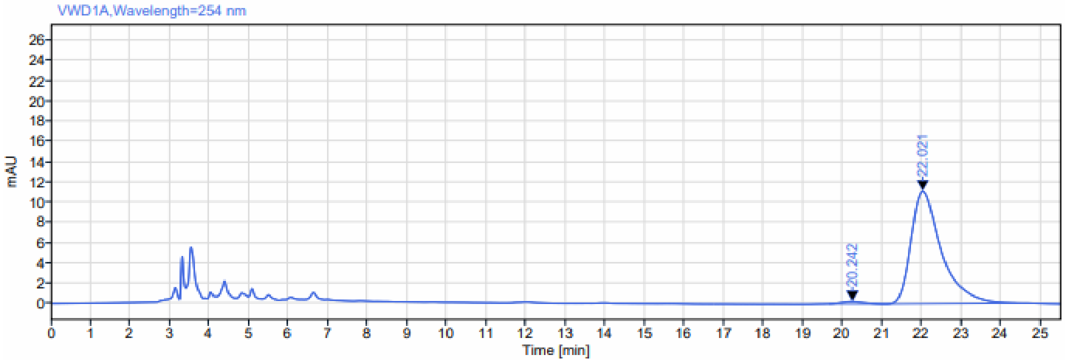

Signal: VWD1A,Wavelength=254 nm

RT [min]	Type	Width [min]	Area	Height	Area%	Name
20.242	MM m	1.45	9.09	0.24	1.52	
22.021	BB	3.31	590.76	11.10	98.48	
		**Sum**	**599.85**			

Supplementary Figure 222. HPLC spectrum of compound **4p**

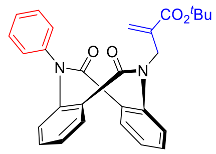

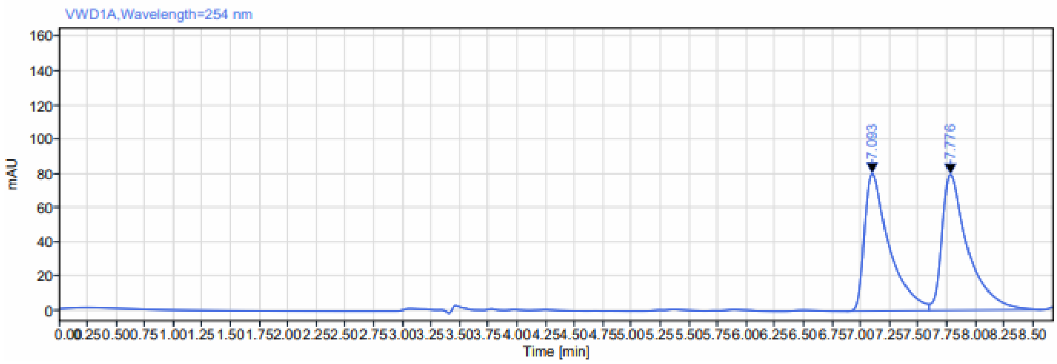

Signal: VWD1A,Wavelength=254 nm

RT [min]	Type	Width [min]	Area	Height	Area%	Name
7.093	BV	0.72	1206.62	80.35	49.14	
7.776	VB	0.98	1248.78	79.53	50.86	
		**Sum**	**2455.40**			

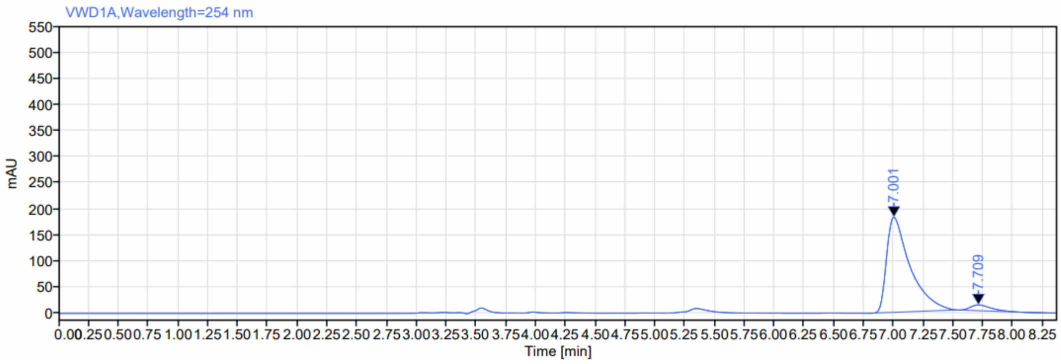

Signal: VWD1A,Wavelength=254 nm

RT [min]	Type	Width [min]	Area	Height	Area%	Name
7.001	BM m	0.74	2498.69	182.28	95.06	
7.709	MM m	0.75	129.90	11.10	4.94	
		**Sum**	**2628.59**			

Supplementary Figure 223. HPLC spectrum of compound **5p**

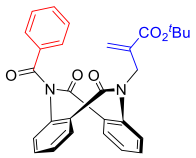

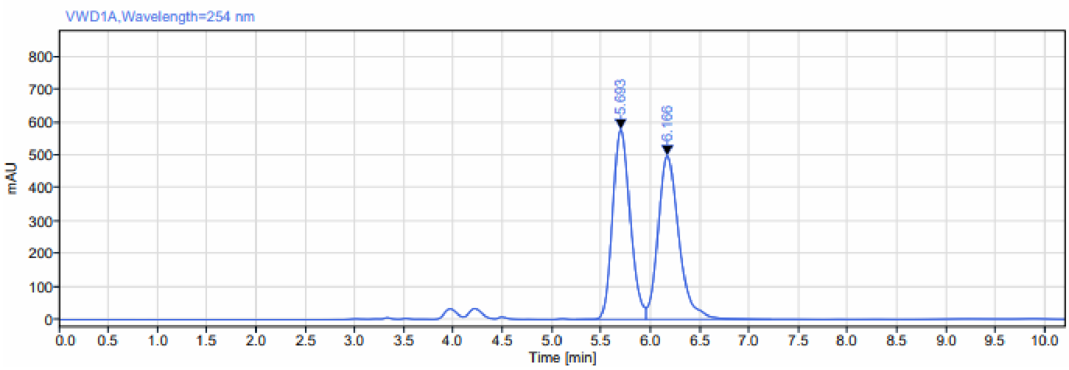

Signal: VWD1A,Wavelength=254 nm

RT [min]	Type	Width [min]	Area	Height	Area%	Name
5.693	BV	0.71	7146.50	576.75	49.36	
6.166	VB	1.27	7331.19	497.00	50.64	
		**Sum**	**14477.68**			

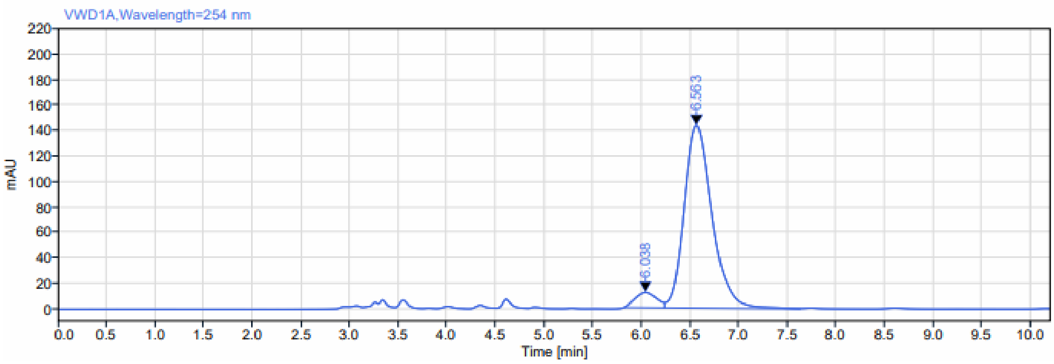

Signal: VWD1A,Wavelength=254 nm

RT [min]	Type	Width [min]	Area	Height	Area%	Name
6.038	MM m	0.42	183.72	11.92	6.03	
6.563	MV m	1.39	2863.41	143.36	93.97	
		**Sum**	**3047.13**			

Supplementary Figure 224. HPLC spectrum of compound **5q**

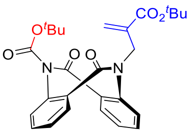

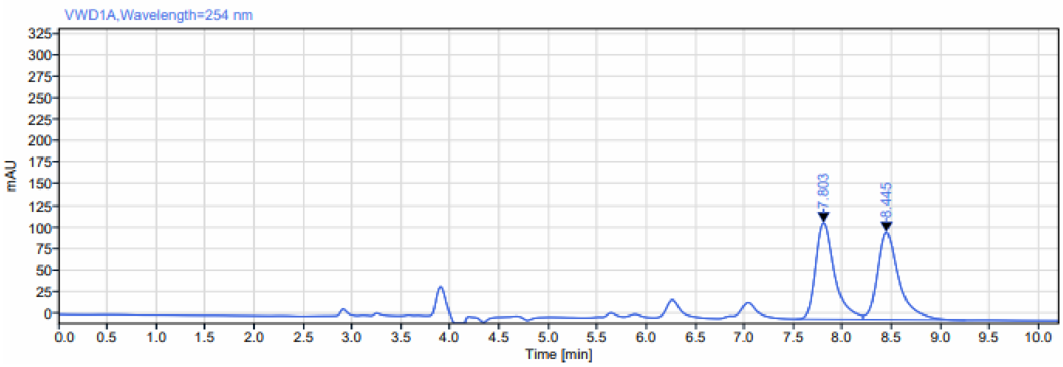

Signal: VWD1A,Wavelength=254 nm

RT [min]	Type	Width [min]	Area	Height	Area%	Name
7.803	BV	0.69	1510.23	111.40	49.23	
8.445	VB	1.05	1557.51	100.98	50.77	
		**Sum**	**3067.74**			

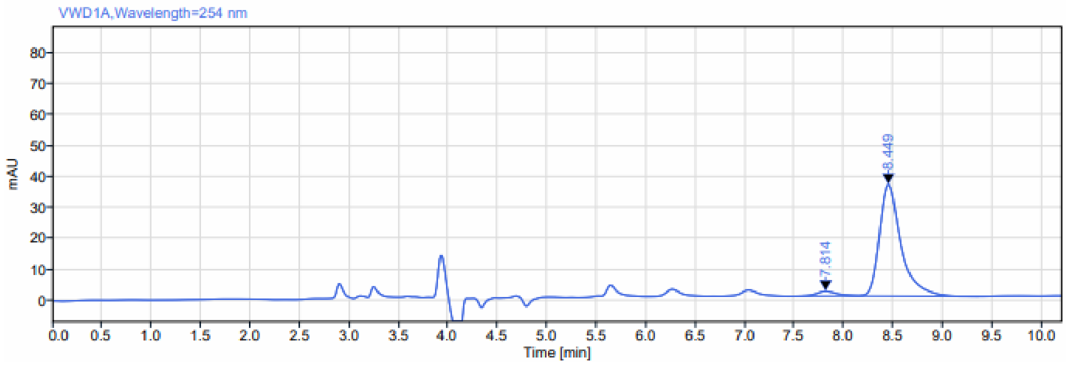

Signal: VWD1A,Wavelength=254 nm

RT [min]	Type	Width [min]	Area	Height	Area%	Name
7.814	MM m	0.57	20.23	1.49	3.60	
8.449	MB m	1.22	542.19	35.92	96.40	
		**Sum**	**562.42**			

Supplementary Figure 225. HPLC spectrum of compound **5r**

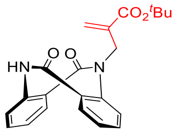

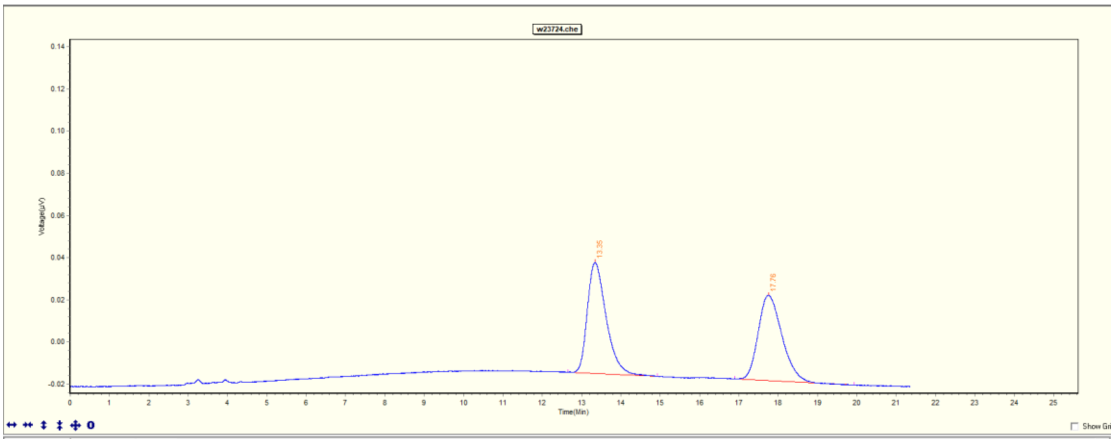

Integration Result | Calculation Result | TimeTable

No.	Retention Time	Peak Area	Peak Height	Peak Area(%)	Peak Width	Peak Type
1	13.35	867915	26280	50.83%	2.274	BB
2	17.76	839451	20174	49.17%	3.033	BB
Total		1,707,366	46,454	100.00%		

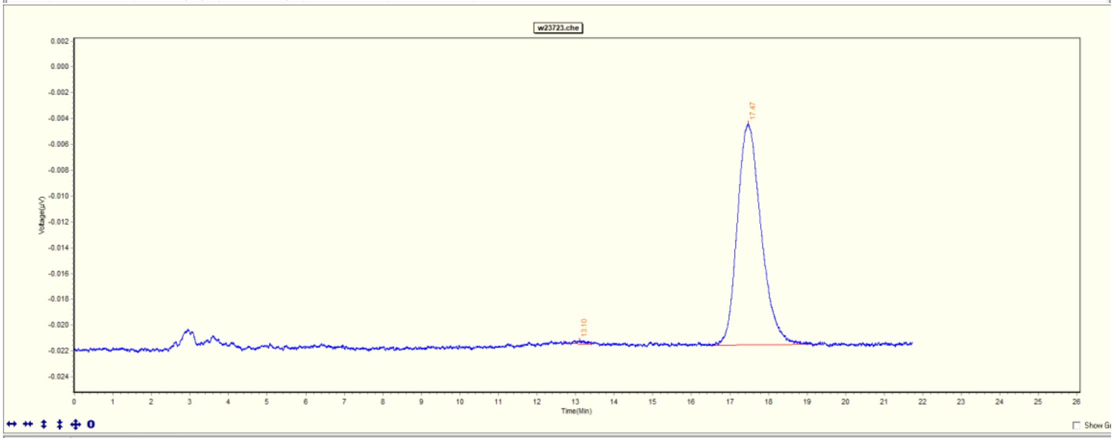

Integration Result | Calculation Result | TimeTable

No.	Retention Time	Peak Area	Peak Height	Peak Area(%)	Peak Width	Peak Type
1	13.10	2836	138	0.77%	0.72	BB
2	17.47	367067	8568	99.23%	2.881	BB
Total		369,903	8,706	100.00%		

Supplementary Figure 226. HPLC spectrum of compound **1a′**

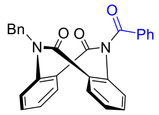

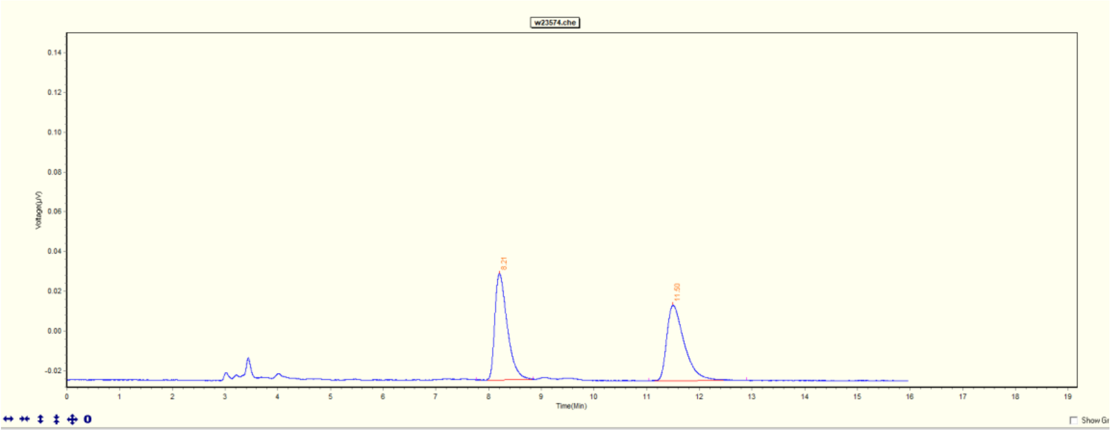

No.	Retention Time	Peak Area	Peak Height	Peak Area(%)	Peak Width	Peak Type
1	8.21	437698	26687	49.89%	1.083	BB
2	11.50	439552	18984	50.11%	1.853	BB
Total		877,250	45,671	100.00%		

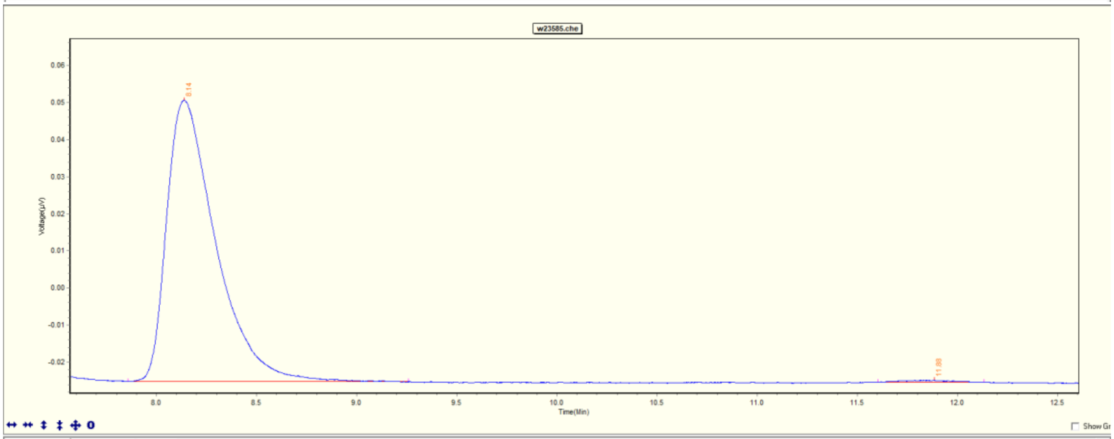

No.	Retention Time	Peak Area	Peak Height	Peak Area(%)	Peak Width	Peak Type
1	8.14	645227	37919	99.32%	1.402	BB
2	11.88	4394	285	0.68%	0.53	BB
Total		649,621	38,204	100.00%		

Supplementary Figure 227. HPLC spectrum of compound **6a**

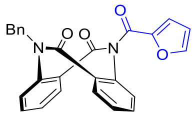

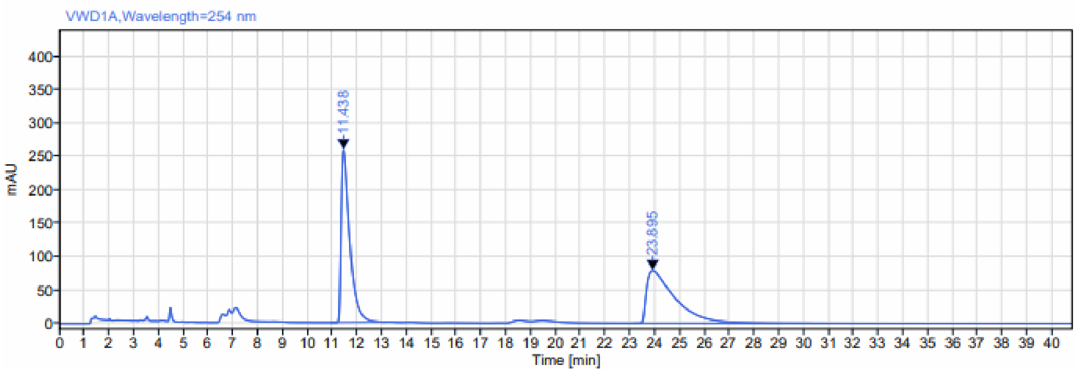

Signal: VWD1A,Wavelength=254 nm

RT [min]	Type	Width [min]	Area	Height	Area%	Name
11.438	BB	2.24	6283.08	258.75	49.84	
23.895	BB	7.16	6324.33	78.83	50.16	
		**Sum**	**12607.42**			

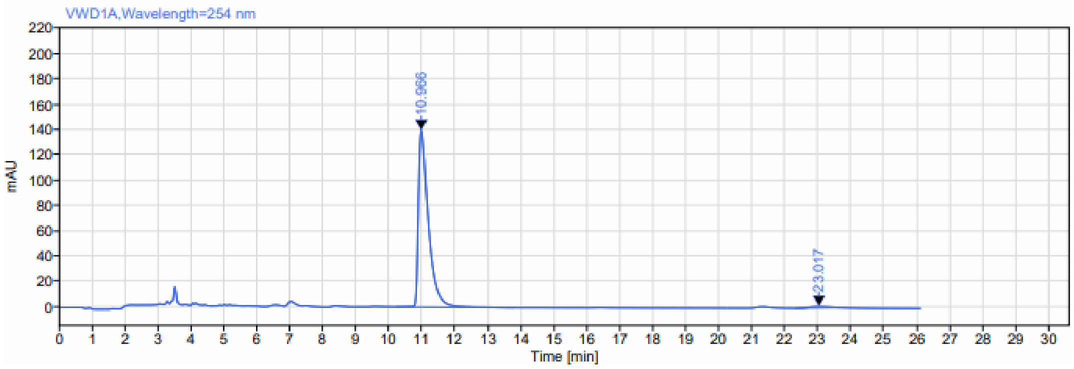

Signal: VWD1A,Wavelength=254 nm

RT [min]	Type	Width [min]	Area	Height	Area%	Name
10.966	BB	3.54	2964.85	139.38	98.70	
23.017	BM m	1.35	39.19	0.98	1.30	
		**Sum**	**3004.04**			

Supplementary Figure 228. HPLC spectrum of compound **6b**

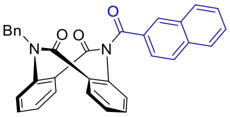

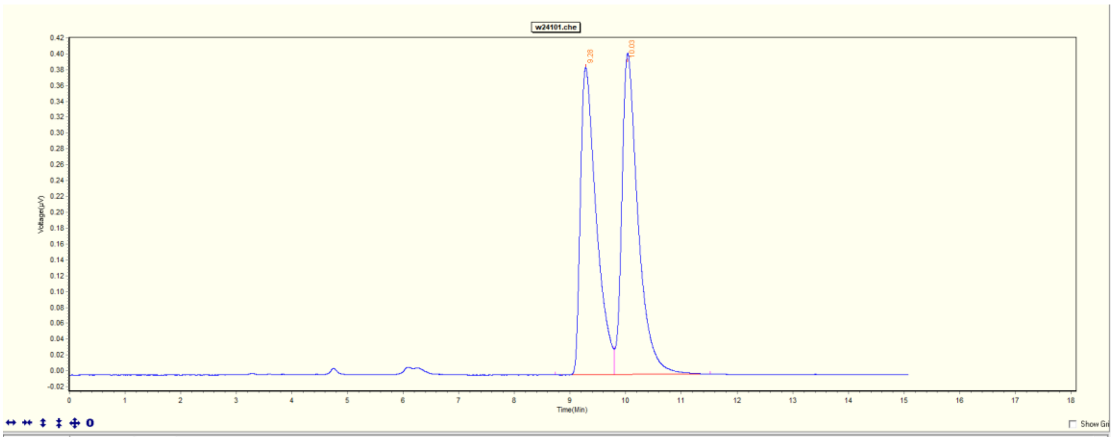

Integration Result | Calculation Result | TimeTable

No.	Retention Time	Peak Area	Peak Height	Peak Area(%)	Peak Width	Peak Type
1	9.28	3845704	193762	48.27%	1.059	BV
2	10.03	4120925	202476	51.73%	1.725	VB
Total		7,966,629	396,238	100.00%		

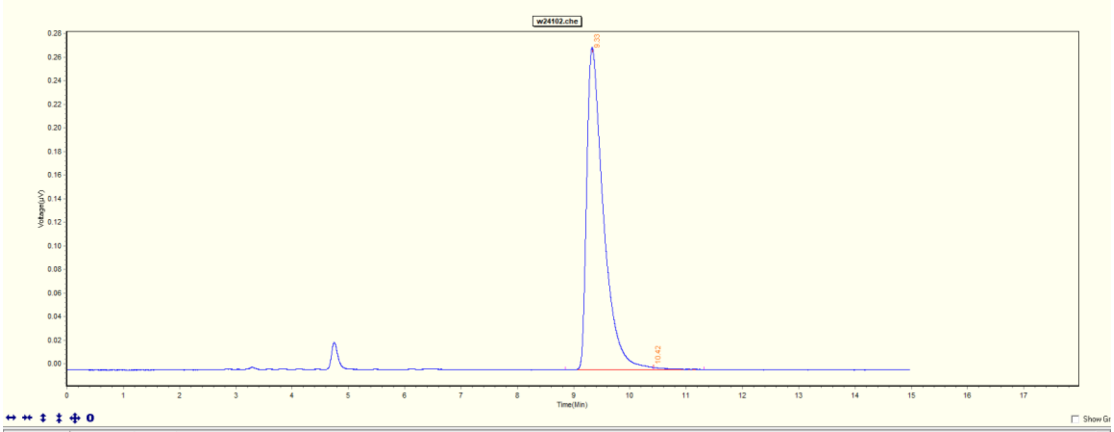

Integration Result | Calculation Result | TimeTable

No.	Retention Time	Peak Area	Peak Height	Peak Area(%)	Peak Width	Peak Type
1	9.33	2810214	136545	99.52%	1.568	BV
2	10.42	13482	809	0.48%	0.894	VB
Total		2,823,696	137,354	100.00%		

Supplementary Figure 229. HPLC spectrum of compound **6c**

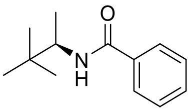

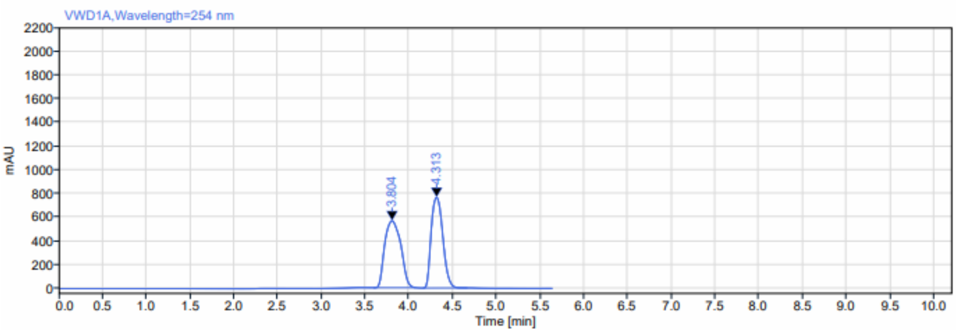

Signal: VWD1A,Wavelength=254 nm

RT [min]	Type	Width [min]	Area	Height	Area%	Name
3.804	MM m	0.48	7188.50	564.54	50.39	
4.313	MM m	0.51	7078.24	763.67	49.61	
		**Sum**	**14266.75**			

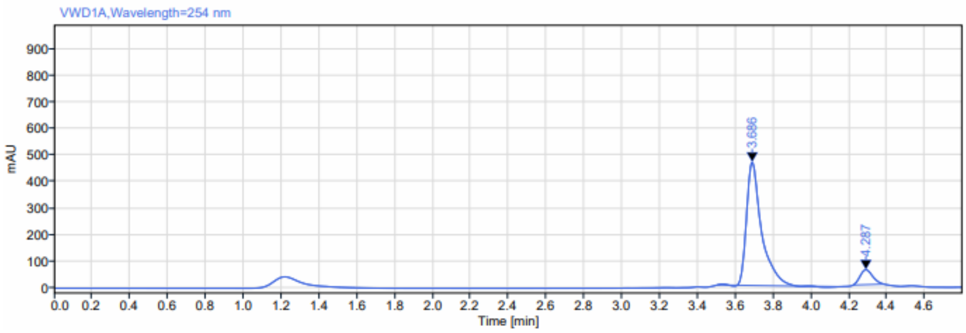

Signal: VWD1A,Wavelength=254 nm

RT [min]	Type	Width [min]	Area	Height	Area%	Name
3.686	MM m	0.65	2551.85	464.16	90.81	
4.287	MM m	0.16	258.27	55.90	9.19	
		**Sum**	**2810.12**			

Supplementary Figure 230. HPLC spectrum of compound **8a**

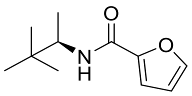

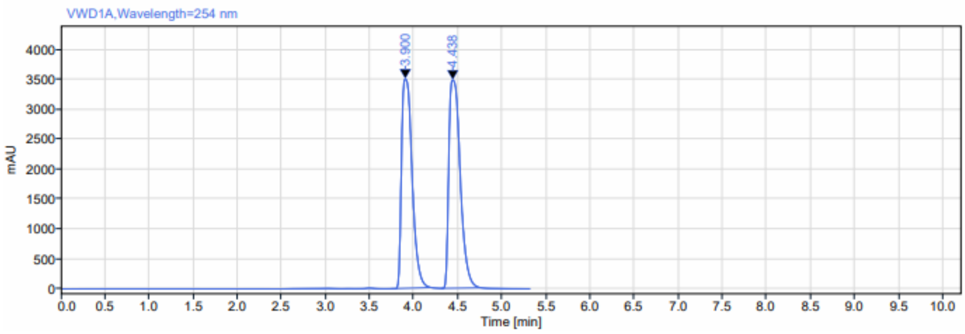

Signal:	VWD1A,Wavelength=254 nm					
**RT [min]**	**Type**	**Width [min]**	**Area**	**Height**	**Area%**	**Name**
3.900	MM m	0.45	29233.46	3503.30	48.28	
4.438	MM m	0.45	31320.78	3484.85	51.72	
		**Sum**	**60554.23**			

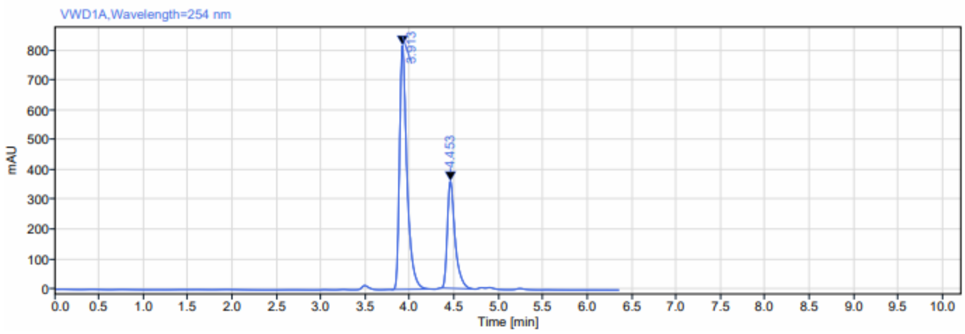

Signal:	VWD1A,Wavelength=254 nm					
**RT [min]**	**Type**	**Width [min]**	**Area**	**Height**	**Area%**	**Name**
3.913	MM m	0.44	4758.13	818.45	68.76	
4.453	MM m	0.36	2161.78	358.67	31.24	
		**Sum**	**6919.91**			

Supplementary Figure 231. HPLC spectrum of compound **8b**

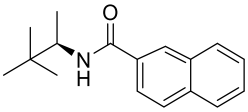

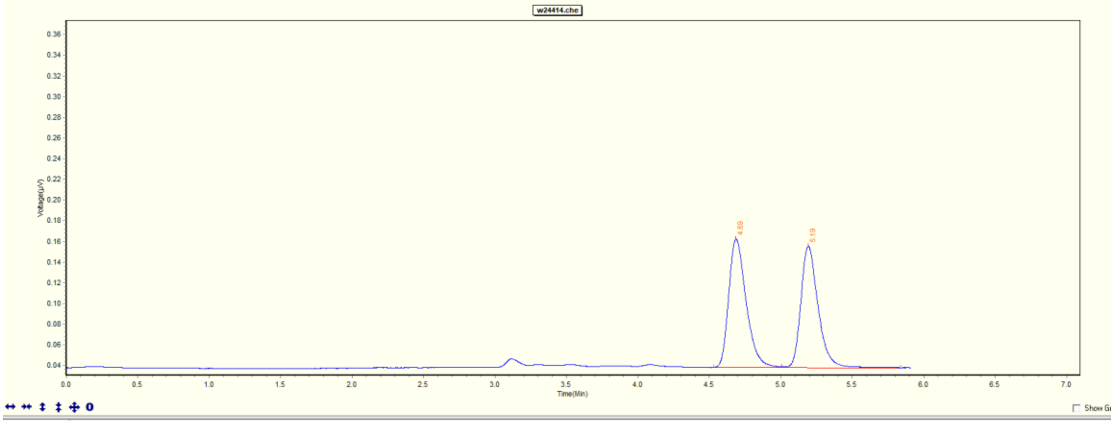

Integration Result | Calculation Result | TimeTable

No.	Retention Time	Peak Area	Peak Height	Peak Area(%)	Peak Width	Peak Type
1	4.69	525315	61980	51.76%	0.474	BV
2	5.19	489616	58542	48.24%	0.833	VB
Total		1,014,931	120,522	100.00%		

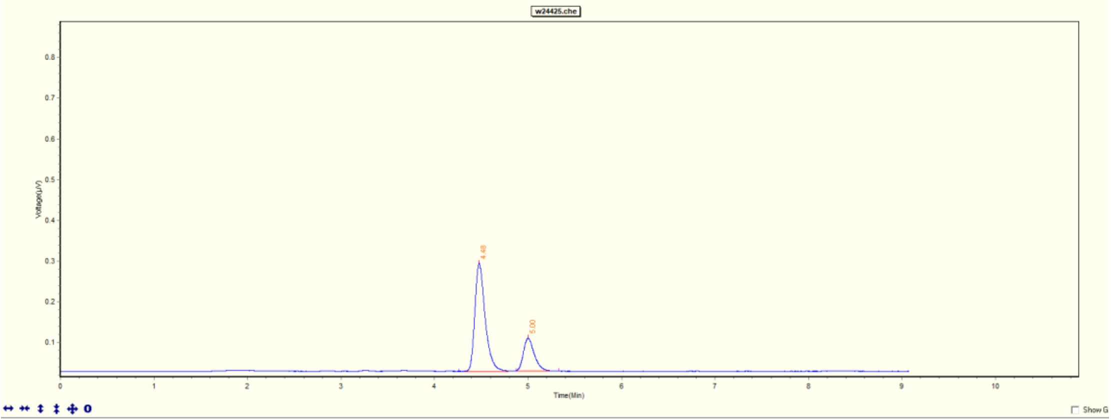

Integration Result | Calculation Result | TimeTable

No.	Retention Time	Peak Area	Peak Height	Peak Area(%)	Peak Width	Peak Type
1	4.48	1009902	132561	75.19%	0.607	BV
2	5.00	333210	40825	24.81%	0.457	VB
Total		1,343,112	173,386	100.00%		

Supplementary Figure 232. HPLC spectrum of compound **8c**

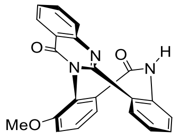

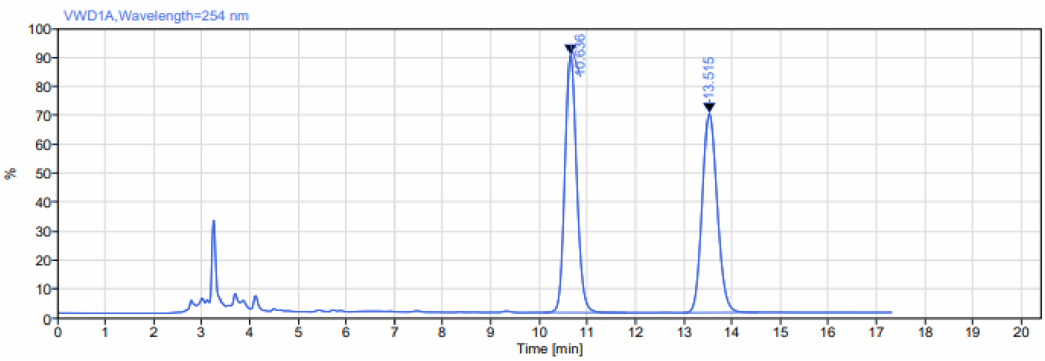

Signal: VWD1A,Wavelength=254 nm

RT [min]	Type	Width [min]	Area	Height	Area%	Name
10.636	BB	1.72	704.70	42.13	50.16	
13.515	BB	1.57	700.07	32.53	49.84	
		**Sum**	**1404.77**			

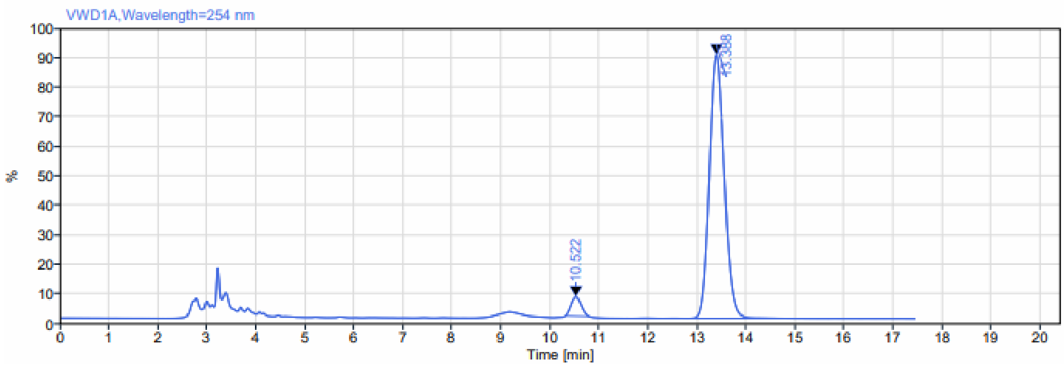

Signal: VWD1A,Wavelength=254 nm

RT [min]	Type	Width [min]	Area	Height	Area%	Name
10.522	MM m	0.49	39.99	2.78	4.67	
13.388	BB	1.82	815.86	38.31	95.33	
		**Sum**	**855.84**			

Supplementary Figure 233. HPLC spectrum of compound **(*S*)-10**

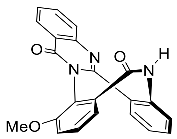

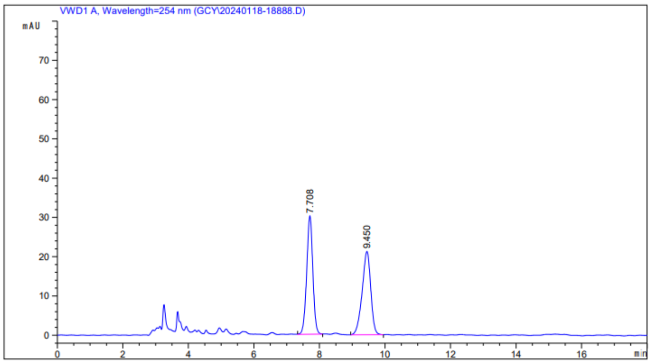

Area Percent Report

Sorted By	:	Signal
Multiplier	:	1.0000
Dilution	:	1.0000
Sample Amount	:	1.00000 [ng/ul] (not used in calc.)

Use Multiplier & Dilution Factor with ISTDs

Signal 1: VWD1 A, Wavelength=254 nm

Peak #	RetTime [min]	Type	Width [min]	Area mAU *s	Height [mAU]	Area %
1	7.708	BB	0.2005	393.97272	30.15417	50.4584
2	9.450	BB	0.2768	386.81381	21.20975	49.5416
Totals :				780.78653	51.36392	

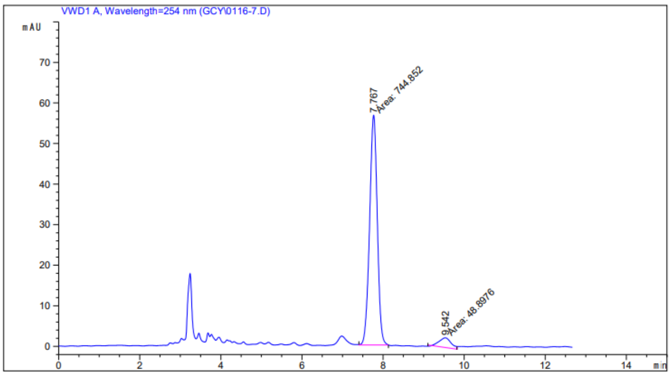

Area Percent Report

Sorted By	:	Signal
Multiplier	:	1.0000
Dilution	:	1.0000
Sample Amount	:	1.00000 [ng/ul] (not used in calc.)

Use Multiplier & Dilution Factor with ISTDs

Signal 1: VWD1 A, Wavelength=254 nm

Peak #	RetTime [min]	Type	Width [min]	Area mAU *s	Height [mAU]	Area %
1	7.767	MM	0.2191	744.85168	56.66010	93.8397
2	9.542	MM	0.3394	48.89759	2.40140	6.1603

Supplementary Figure 234. HPLC spectrum of compound ***(R)*-10**

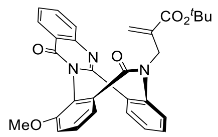

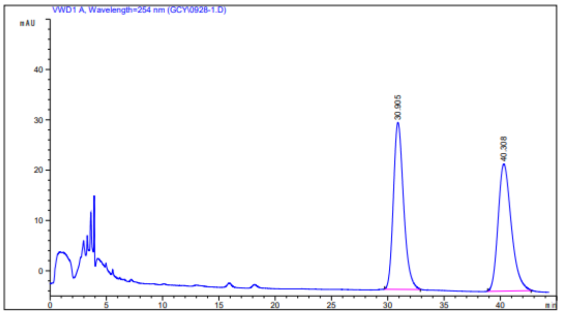


```
=====================================================================
                        Area Percent Report
=====================================================================

Sorted By             :      Signal
Multiplier            :      1.0000
Dilution              :      1.0000
Sample Amount         :      1.00000  [ng/ul]   (not used in calc.)
Use Multiplier & Dilution Factor with ISTDs


Signal 1: VWD1 A, Wavelength=254 nm

Peak RetTime Type  Width     Area      Height     Area
  #   [min]        [min]  mAU    *s   [mAU   ]      %
----|-------|----|-------|----------|----------|--------|
   1  30.905 BB    0.9581 2087.69214   33.18713  50.2226
   2  40.308 BB    1.2316 2069.18311   25.27773  49.7774

Totals :                  4156.87524   58.46486
```

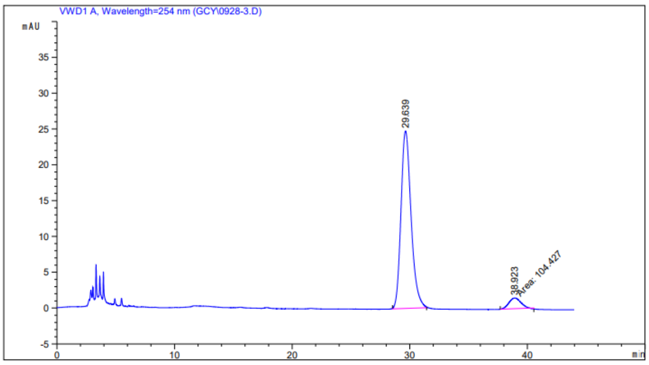


```
=====================================================================
                        Area Percent Report
=====================================================================

Sorted By             :      Signal
Multiplier            :      1.0000
Dilution              :      1.0000
Sample Amount         :      1.00000  [ng/ul]   (not used in calc.)
Use Multiplier & Dilution Factor with ISTDs


Signal 1: VWD1 A, Wavelength=254 nm

Peak RetTime Type  Width     Area      Height     Area
  #   [min]        [min]  mAU    *s   [mAU   ]      %
----|-------|----|-------|----------|----------|--------|
   1  29.639 BB    0.9105 1478.40662   24.76352  93.4025
   2  38.923 MM    1.1687  104.42670    1.48921   6.5975

Totals :                  1582.83331   26.25273
```

Supplementary Figure 235. HPLC spectrum of compound **11**

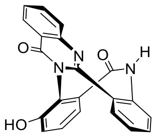

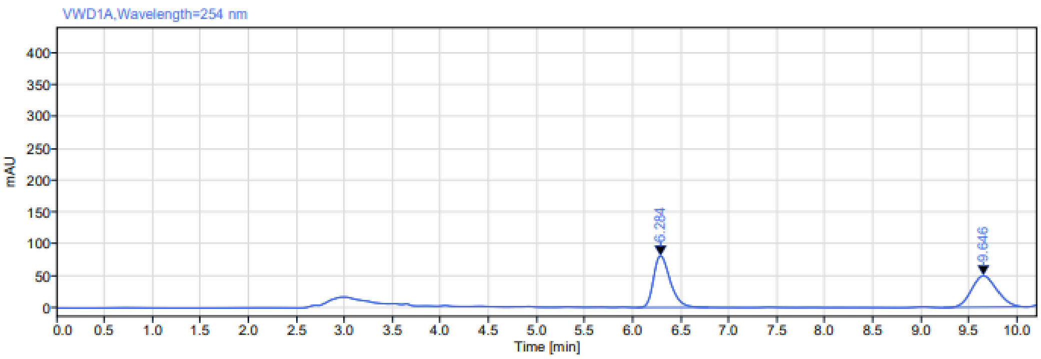

Signal: VWD1A,Wavelength=254 nm

RT [min]	Type	Width [min]	Area	Height	Area%	Name
6.284	BM m	0.75	964.26	80.84	51.64	
9.646	MM m	0.89	903.00	48.75	48.36	
		**Sum**	**1867.26**			

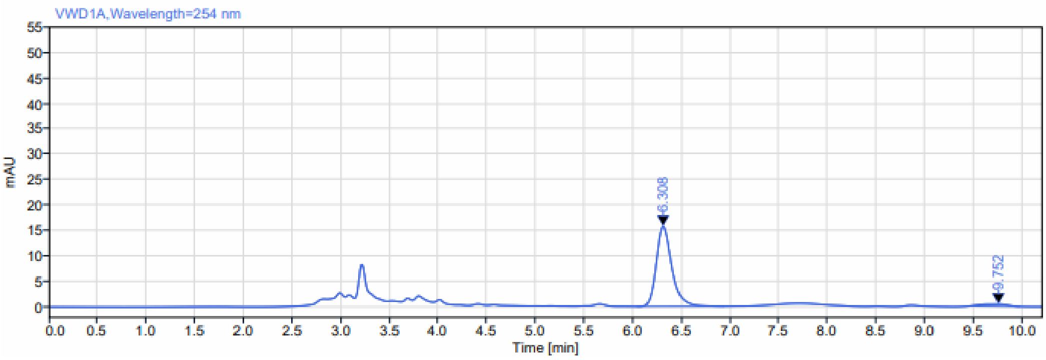

Signal: VWD1A,Wavelength=254 nm

RT [min]	Type	Width [min]	Area	Height	Area%	Name
6.308	BB	0.90	175.80	15.75	95.70	
9.752	MM m	0.51	7.91	0.42	4.30	
		**Sum**	**183.71**			

Supplementary Figure 236. HPLC spectrum of compound ***(S)*-12**

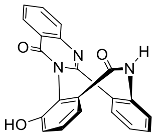

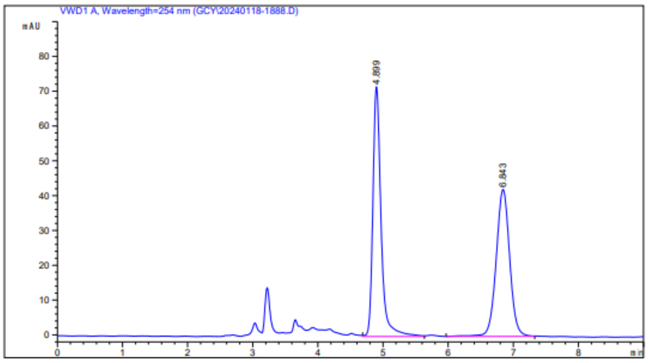

===

Area Percent Report

===

```
Sorted By             :      Signal
Multiplier            :      1.0000
Dilution              :      1.0000
Sample Amount         :      1.00000  [ng/ul]   (not used in calc.)
Use Multiplier & Dilution Factor with ISTDs
```

Signal 1: VWD1 A, Wavelength=254 nm

Peak #	RetTime [min]	Type	Width [min]	Area mAU *s	Height [mAU]	Area %
1	4.899	VB	0.1310	626.72217	71.74048	50.7966
2	6.843	VB	0.2218	607.06439	42.18090	49.2034
Totals :				1233.78656	113.92138	

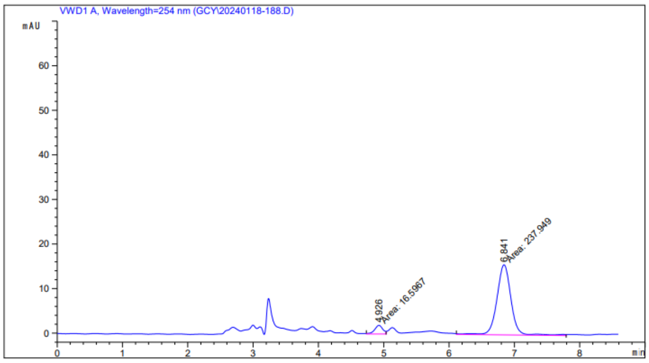

===

Area Percent Report

===

```
Sorted By             :      Signal
Multiplier            :      1.0000
Dilution              :      1.0000
Sample Amount         :      1.00000  [ng/ul]   (not used in calc.)
Use Multiplier & Dilution Factor with ISTDs
```

Signal 1: VWD1 A, Wavelength=254 nm

Peak #	RetTime [min]	Type	Width [min]	Area mAU *s	Height [mAU]	Area %
1	4.926	MM	0.1439	16.59669	1.92208	6.5201
2	6.841	MM	0.2520	237.94855	15.73705	93.4799
Totals :				254.54524	17.65913	

Supplementary Figure 237. HPLC spectrum of compound ***(R)*-12**

11. General procedure for the in vitro antitumor activity study

Cell viability was measured by CellTiter-Glo® Reagent

For cell viability, cells were seeded in 96-well plates per well. After 12 hours, serially diluted compounds were added and cells were cultured for another 72 hours. Then add 40 μL of CellTiter-Glo® Reagent (Promega, G7571) into each well and mix contents for 2 minutes on an orbital shaker to induce cell lysis. Incubate at room temperature for 60 minutes to stabilize luminescent signal and record luminescence on Multilabel Reader. The IC_{50} value of the compound was calculated using GraphPad Prism 5.

These representative products **3a**, **3f**, **3g**, **3h**, **3j**, **3m**, **3p**, **3q, 5a**, **5e**, **5f**, **5g**, **5h**, **5n**, **5o** on cell viability was evaluated *via* the CTG assay in A2780 (ovarian cancer cells), Hela (cervical carcinoma cells), HT-29 (colon cancer cells), LoVo (colon cancer cells), MV-4-11 (acute monocytic leukemia cells), and U87-MG (astrocytoma cells) human cancer cell lines, and the in vitro anti-tumor activity results have been listed in **Supplementary Table 16.**

Most of these Octa-amide derivatives showed significant anti-cancer activity, compounds **3a**, **3q**, and **5g** were were selected for further evaluation. The results show that the KR product (compound **5g**) show the highest anticancer potency against ovarian cancer cells (A2780 cells, IC50=10.58 μM). The results were presented as percentages.

Supplementary Table 16. Anti-tumor activities of compounds **3a**, **3f**, **3g**, **3h**, **3j**, **3m**, **3p**, **3q, 5a**, **5e**, **5f**, **5g**, **5h**, **5n** and **5o** (Inhibition rate at 50 μM)

Compound	Cell Types					
	A2780 (%)	HeLa (%)	HT-29 (%)	LoVo (%)	MV-4-11 (%)	U87-MG (%)
**3a**	91.45	50.58	92.16	85.52	98.95	81.66
**3f**	99.59	97.59	97.65	94.68	99.87	69.42
**3g**	93.03	74.31	97.31	92.97	99.93	87.14
**3h**	97.59	92.01	98.25	95.70	99.94	18.74
**3j**	85.32	60.68	81.04	70.18	97.54	46.81
**3m**	99.65	97.52	98.99	98.73	99.84	96.62
**3p**	82.08	68.87	82.23	82.20	98.15	30.66
**3q**	78.32	17.00	74.57	64.01	96.28	61.10
**5a**	69.21	11.36	64.54	59.16	81.91	36.03
**5e**	99.72	99.25	98.01	98.99	99.54	98.08
**5f**	70.73	44.45	25.75	69.26	4.51	62.85
**5g**	99.49	85.55	96.06	94.17	99.68	56.56
**5h**	56.86	11.65	46.91	38.59	64.83	16.17
**5n**	33.74	17.24	60.13	52.19	54.72	22.61
**5o**	99.68	91.96	99.23	99.09	99.92	89.56

Supplementary Table 17. Anti-tumor activities of compounds **3a**, **3q** and **5g** (IC_{50}, μM)

Compound	Cell Types					
	A2780 (%)	HeLa (%)	HT-29 (%)	LoVo (%)	MV-4-11 (%)	U87-MG (%)
**3a**	13.89±0.9	37.28±0.2	28.41±1.6	36.25±0.55	21.00±0.83	47.68±0.80
**3q**	16.24±0.4	61.51±1.8	54.07±5.2	53.60±1.05	38.79±3.16	109.33±2.60
**5g**	10.58±1.25	16.80±0.4	15.06±0.4	14.75±0.46	14.27±0.17	25.43±0.43

IC_{50} is the half maximal inhibitory concentration.

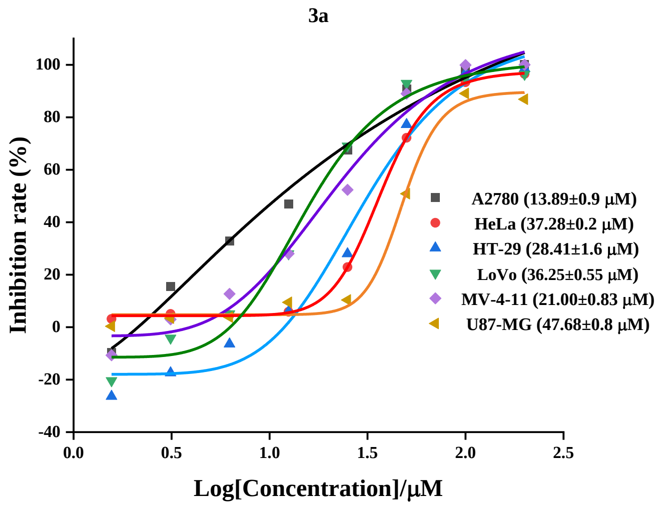

Supplementary Figure 238. Compound **3a** on the inhibition of A2780, HeLa, HT-29, LoVo, MV-4-11 and U87-MG cells.

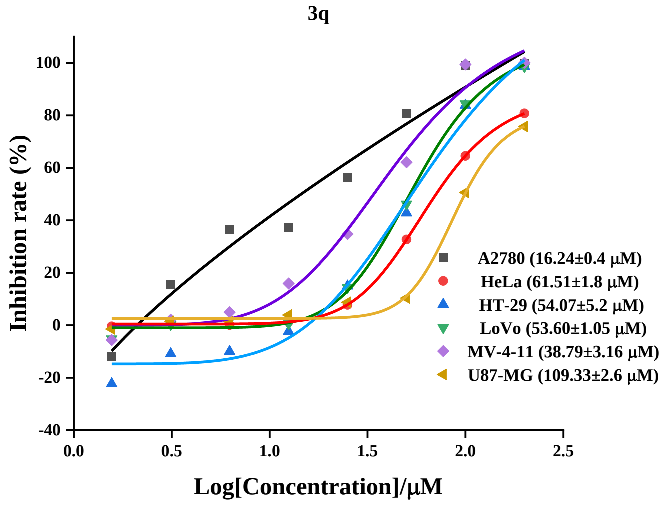

Supplementary Figure 239. Compound **3q** on the inhibition of A2780, HeLa, HT-29, LoVo, MV-4-11 and U87-MG cells.

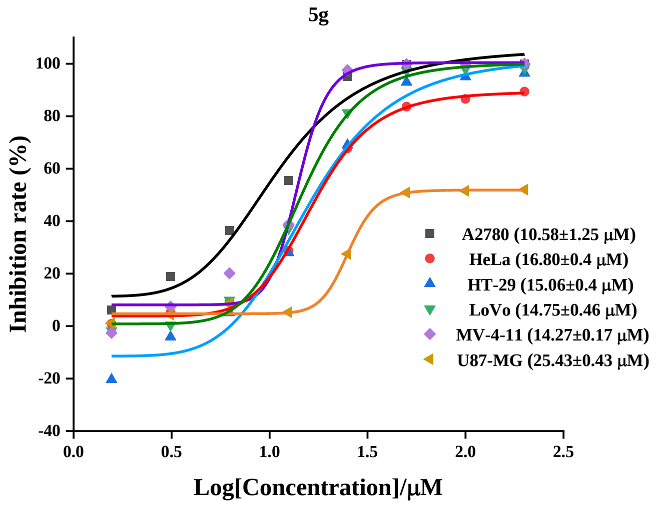

Supplementary Figure 240. Compound **5g** on the inhibition of A2780, HeLa, HT-29, LoVo, MV-4-11 and U87-MG cells.

12. DFT Computational studies

All computations were performed with the Gaussian16[5] program by using density functional theory (DFT). Then, the geometry optimization of compounds was carried out with the M06-2X[6,7] functional using 6-31G(d,p) basis set. Additionally, single-point energy calculations were performed at the M06-2X/6-311++G(2d,p) level. The solvation effect was considered in acetonitrile solvent by employing Solvation Model Density (SMD) [8]. In addition, we used Multiwfn[9], and VMD[10] to draw the pictures of the three-dimensional structures of transition states, as well as the AIM[11] and NCI analysis, we used CYL view[12] to draw the pictures of the three-dimensional structures of transition states.

12.1 Proposed reaction pathway of DKR and KR

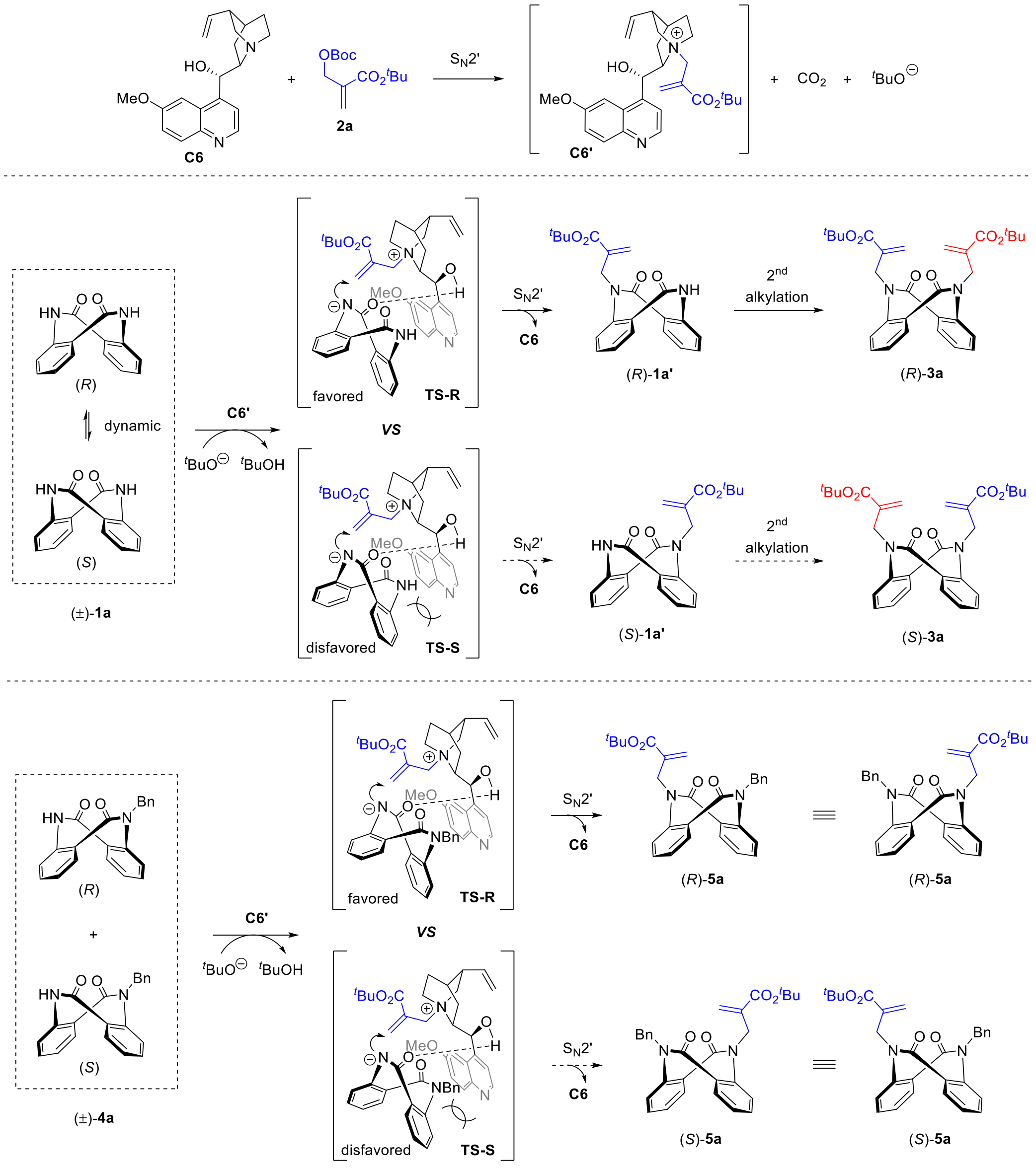

Supplementary Figure 241. Proposed reaction pathway of DKR and KR

12.2 The Gibbs free energy profiles of the stereoselective pathways

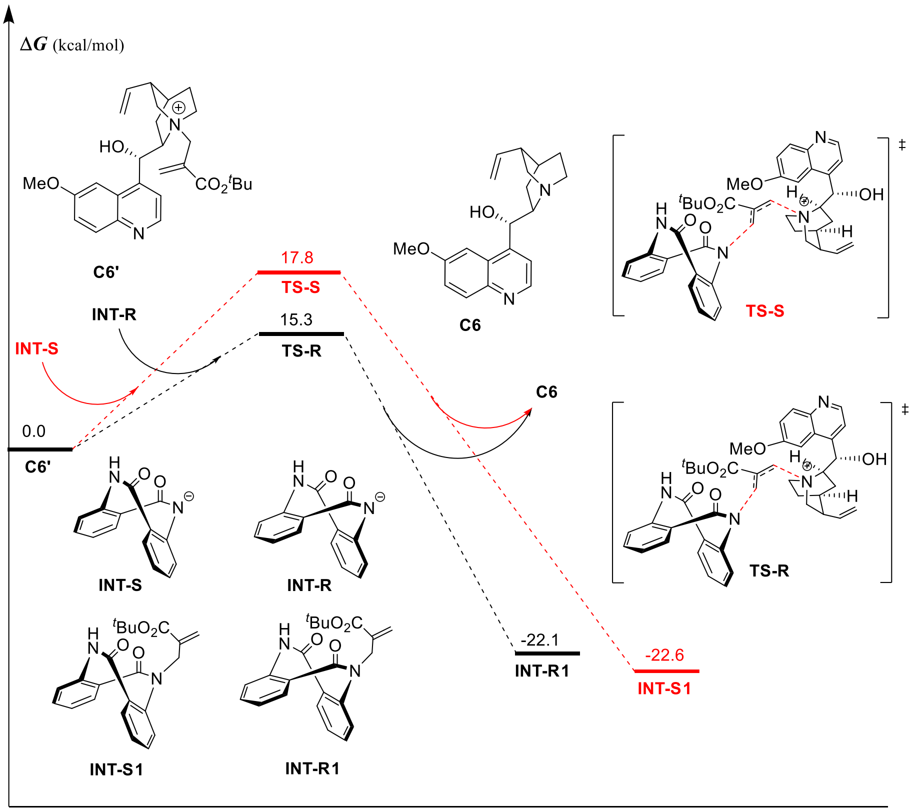

Supplementary Figure 242.: The Gibbs free energy profiles of the stereoselective pathways

As shown in **Supplementary Figure 242**, the double bond of **C6'** is attacked by the nitrogen ions of **INT-S** and **INT-R** via transaction states **TS-R** ($\Delta G^{\ddagger}$= 15.3 kcal/mol) and **TS-S** ($\Delta G^{\ddagger}$= 17.8 kcal/mol) respectively. Subsequently, the breaking of another C–N bond results in the formation of another double bond, yielding **C6** and generating **INT-S1** and **INT-R1** respectively. DFT study shows that the energy barrier of **TS-R** is 2.5 kcal/mol lower than that associated with **TS-S**, indicating that **TS-R** represents the preferred conformation. According to Boltzmann distribution, the calculated enantiomeric excess (*ee*) value is 97.1%, which is consistent with the experimental results.

Supplementary equation (4): $P_R = Q_{R\ (Relat)}/Q_{(Relat)} = Q_{R\ (Relat)}/[Q_{R(Relat)} + Q_{S(Relat)}] = e^{-\Delta E_R/RT}/(e^{-\Delta E_R/RT} + e^{-\Delta E_S/RT}) = 98.59\%$, $P_S = 1.41\%$.

Supplementary equation (5): $ee = P_R - P_S = 97.18\%$.

(ΔE_R =15.3 kcal/mol, ΔE_S =17.8 kcal/mol, T=298.15 K, R = 8.31451 $J{\cdot}K^{-1}{\cdot}mol^{-1}$)

12.3 The NCI and AIM analyses for the Transition states

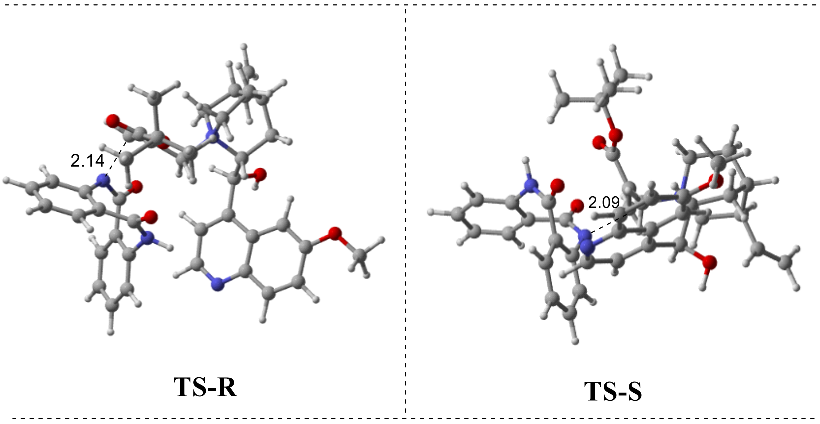

Supplementary Figure 243. The 3D structures of the optimized transition states **TS-R** and **TS-S** (distance in angstrom)

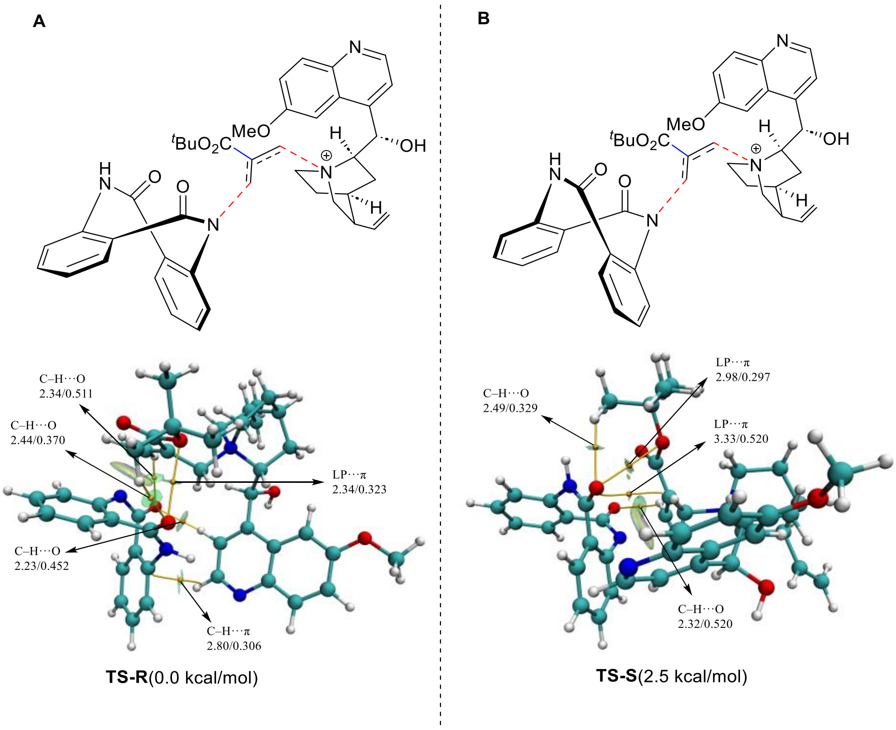

Supplementary Figure 244. NCI and AIM analyses for the transition states **TS-R** and **TS-S**, and the summary of Laplacian electron density (∇2ρ in 10−1 a.u.)

As showed in the **Supplementary Figure 243**, in the three-dimensional structures, oxygen, nitrogen, carbon, phosphorus, and hydrogen atoms are depicted as red, blue, green, brown, and white balls, respectively. Bond critical points (BCPs) along the bond paths were visualized as small orange balls connected by yellow lines.

To delve deeper into the origin and determining factors of the stereoselectivity, the

further qualitative and quantitative analyses of non-covalent interactions in stereoselectivity-determining transition states were depicted in **Supplementary Figure 244**, and the color scheme of blue, green, and red represents strong, weak interactions, and steric hindrance, respectively. Notably, **TS-R** exhibits C–H···O and LP···π as well as C–H···π weak interactions, with its C–H···O interaction being stronger than that in **TS-S**, indicating that hydrogen bond interactions should be key in controlling stereoselectivity.

13. Supplementary references

[1] Qiu, L., Wang, X., Zhao, N. *et al*. Reductive ring closure methodology toward heteroacenes bearing a dihydropyrrolo [3,2-*B*] pyrrole core: Scope and limitation. *J. Org. Chem.* **79**, 11339-11348, (2014).

[2] Yang, X. H., Li, J. P., Wang, D. C. *et al*. Enantioselective dearomative [3+2] cycloaddition of 2-nitrobenzofurans with aldehyde-derived Morita–Baylis–Hillman carbonates. *Chem. Commun.* **55**, 9144-9147, (2019).

[3] Bieszczad, B., Pawlędzio, S., Polak, K. *et al*. Influence of halogen size on the supramolecular and energy landscape of the THF solvates of the halogen derivatives of dianthranilide. *CrystEngComm.* **22**, 5389-5399, (2020).

[4] Bieszczad, B., Garbicz, D., Trzybiński, D. *et al*. Unsymmetrically substituted dibenzo [*b*,*f*] [1,5]-diazocine-6, 12 (5*H*, 11*H*) dione—A convenient scaffold for bioactive molecule design. *Molecules.* **25**, 906, (2020).

[5] Frisch, M. J., Trucks, G. W., Schlegel, H. B., Scuseria, G. E., Robb, M. A., Cheeseman, J. R., Scalmani, G., Barone, V., Petersson, G. A., Nakatsuji, H., Li, X., Caricato, M., Marenich, V., Bloino, J., Janesko, B. G., Gomperts, R., Mennucci, B., Hratchian, H. P., Ortiz, J. V., Izmaylov, A. F., Sonnenberg, J. L., Williams-Young, D., Ding, F., Lipparini, F., Egidi, F., Goings, J., Peng, B., Petrone, A., Henderson, T., Ranasinghe, D., Zakrzewski, V. G., Gao, J., Rega, N., Zheng, G., Liang, W., Hada, M., Ehara, M., Toyota, K., Fukuda, R., Hasegawa, J., Ishida, M., Nakajima, T., Honda, Y., Kitao, O., Nakai, H., Vreven, T., Throssell, K., Montgomery, J. A., Jr., Peralta, J. E., Ogliaro, F., Bearpark, M., Heyd, J. J., Brothers, E., Kudin, K. N., Staroverov, V. N., Keith, T. A., Kobayashi, R., Normand, J., Raghavachari, K., Rendell, A. P., Burant, J. C., Iyengar, S. S., Tomasi, J., Cossi, M., Millam, J. M., Klene, M., Adamo, C., Cammi, R., Ochterski, J. W., Martin, R. L., Morokuma, K., Farkas, O., Foresman J. B., Fox, D. J., Gaussian, Inc., Wallingford CT, 2016.

[6] Zhao, Y. & Truhlar, D, G. The M06 suite of density functionals for main group thermochemistry, thermochemical kinetics, noncovalent interactions, excited states, and transition elements: two new functionals and systematic testing of four M06-class functionals and 12 other functionals. *Theor. Chem. Acc.* **120**, 215-241, (2008).

[7] Zhao, Y. & Truhlar, D. G. Exploring the limit of accuracy of the global hybrid meta density functional for main-group thermochemistry, kinetics, and noncovalent interactions. *J. Chem. Theory Comput.* **4**, 1849-1868, (2008).

[8] Mennucci, B. & Tomasi, J. Continuum solvation models: A new approach to the problem of solute's charge distribution and cavity boundaries. *J. Chem. Phys.* **106**, 5151-5158, (1997).

[9] Lu, T. & Chen, F. Multiwfn: A multifunctional wavefunction analyzer. *J. Comput. Chem.* **33**, 580-592, (2012).

[10] Mankowska-Cyl, A., Krintus, M., Rajewski, P., *et al*. Gamma-glutamyltransferase activity as a surrogate biomarker of metabolic health status in young nondiabetic obese women. *Biomarkers Med.* **11**, 449-457, (2017).

[11] Bader, R. F. W. A quantum theory of molecular structure and its applications. *Chem. Rev.* **91**, 893-928, (1991).

[12] Legault, C. Y. CYL view, 1.0b; Université de Sherbrooke, 2009.
