# Supplementary material for: Catalytic asymmetric synthesis of planar-chiral dianthranilides via (Dynamic) kinetic resolution
Source: Nat Commun. 2024 May 29;15:4580. doi: 10.1038/s41467-024-48947-1 (PMC11136957; doi:10.1038/s41467-024-48947-1)
Supplement: Supplementary file 3 — Reporting Summary [file 41467_2024_48947_MOESM3_ESM.pdf]

nature portfolio

Corresponding author(s): Guang-Jian Mei

Last updated by author(s): May 20, 2024

Reporting Summary

Nature Portfolio wishes to improve the reproducibility of the work that we publish. This form provides structure for consistency and transparency in reporting. For further information on Nature Portfolio policies, see our Editorial Policies and the Editorial Policy Checklist.

Statistics

For all statistical analyses, confirm that the following items are present in the figure legend, table legend, main text, or Methods section.

n/a	Confirmed	
☐	☒	The exact sample size (n) for each experimental group/condition, given as a discrete number and unit of measurement
☐	☒	A statement on whether measurements were taken from distinct samples or whether the same sample was measured repeatedly
☒	☐	The statistical test(s) used AND whether they are one- or two-sided<br>*Only common tests should be described solely by name; describe more complex techniques in the Methods section.*
☐	☒	A description of all covariates tested
☒	☐	A description of any assumptions or corrections, such as tests of normality and adjustment for multiple comparisons
☒	☐	A full description of the statistical parameters including central tendency (e.g. means) or other basic estimates (e.g. regression coefficient) AND variation (e.g. standard deviation) or associated estimates of uncertainty (e.g. confidence intervals)
☒	☐	For null hypothesis testing, the test statistic (e.g. F, t, r) with confidence intervals, effect sizes, degrees of freedom and P value noted<br>*Give P values as exact values whenever suitable.*
☒	☐	For Bayesian analysis, information on the choice of priors and Markov chain Monte Carlo settings
☒	☐	For hierarchical and complex designs, identification of the appropriate level for tests and full reporting of outcomes
☒	☐	Estimates of effect sizes (e.g. Cohen's d, Pearson's r), indicating how they were calculated

Our web collection on statistics for biologists contains articles on many of the points above.

Software and code

Policy information about availability of computer code

Data collection	Data was collected using the softwares provided by the respective instrument vendors which were specified in the Methods section.
Data analysis	1. The NMR data recorded on Brucker DPX-400 MHz spectrometers, and were processed by MestRenova 6.1.0<br>2.The crystallography data was analyzed by Bruker D8 VENTURE Metaljet PHOTON II single crystal X-ray diffractometer.<br>3. The computational study was conducted by Gaussian 16 and Gaussian view 6 software, and the results were analyzed by Multiwfn and VMD software.<br>4. The IC50 value of the compound was calculated using GraphPad Prism 5, The IC50 chart data was processed by Origin 2022.<br>5. The HPLC specturms were obtained by Agilent 1100 and Agilent 1260.<br>6.The melting point was measured by the KeRui XR6.<br>7.High resolution data was obtained through the Agilent 1290.

For manuscripts utilizing custom algorithms or software that are central to the research but not yet described in published literature, software must be made available to editors and reviewers. We strongly encourage code deposition in a community repository (e.g. GitHub). See the Nature Portfolio guidelines for submitting code & software for further information.

Data

Policy information about availability of data

All manuscripts must include a data availability statement. This statement should provide the following information, where applicable:

- Accession codes, unique identifiers, or web links for publicly available datasets
- A description of any restrictions on data availability
- For clinical datasets or third party data, please ensure that the statement adheres to our policy

The data relating to the characterization of products, experimental protocols and the computational studies are available within the article and its Supplementary Information. Data for the crystal structure 3m reported in this paper are deposited at the Cambridge Crystallographic Data Centre (CCDC) under the deposition number CCDC 2271258. Copies of the data can be obtained free of charge via www.ccdc.cam.ac.uk/data_request/cif. Further data supporting the findings of this study are available from the corresponding author upon request. Source data are provided with this paper.

Research involving human participants, their data, or biological material

Policy information about studies with human participants or human data. See also policy information about sex, gender (identity/presentation), and sexual orientation and race, ethnicity and racism.

Reporting on sex and gender	N/R
Reporting on race, ethnicity, or other socially relevant groupings	N/R
Population characteristics	N/R
Recruitment	N/R
Ethics oversight	N/R

Note that full information on the approval of the study protocol must also be provided in the manuscript.

Field-specific reporting

Please select the one below that is the best fit for your research. If you are not sure, read the appropriate sections before making your selection.

☒ Life sciences ☐ Behavioural & social sciences ☐ Ecological, evolutionary & environmental sciences

For a reference copy of the document with all sections, see nature.com/documents/nr-reporting-summary-flat.pdf

Life sciences study design

All studies must disclose on these points even when the disclosure is negative.

Sample size	The IC50 value of each cell was tested in at least three parallel groups and eight concentrations were measured in each group of cells. In case that a fold difference in the IC50 value bigger than 10-fold was obtained between replicates, an additional replicate was performed. In addition,the complete dose-effect curve can be accurately fitted with 5~8 concentration points. In general the obtained IC50 values from three parallel experiments met that criteria and hence we consider that sample size accurate enough to report the result.
Data exclusions	No data was excluded
Replication	To ensure robust reproducibility: All bioassay data presented in this manuscript were repeated twice times at least. And all attempts to repeat the experiment were successful.
Randomization	Cells were randomly divided into experimental groups. All other samples collected were used for this study without any discrimination.
Blinding	The evaluation of bioactivity of dianthranilides derivatives were conducted in blinding. The compounds structure and resources information was unknown to the investigator who conducted bioactivity test, to ensure the objective data collection and analysis.

Reporting for specific materials, systems and methods

We require information from authors about some types of materials, experimental systems and methods used in many studies. Here, indicate whether each material, system or method listed is relevant to your study. If you are not sure if a list item applies to your research, read the appropriate section before selecting a response.

Materials & experimental systems

n/a	Involved in the study
x	Antibodies
	x Eukaryotic cell lines
x	Palaeontology and archaeology
x	Animals and other organisms
x	Clinical data
x	Dual use research of concern
x	Plants

Methods

n/a	Involved in the study
x	ChIP-seq
x	Flow cytometry
x	MRI-based neuroimaging

Eukaryotic cell lines

Policy information about cell lines and Sex and Gender in Research

Cell line source(s)	The A2780 cell lines were originally purchased from the cobioer biosciences co., LTD; The Hela, HT-29, LoVo, MV-4-11 and U87-MG cell lines were originally purchased from the American Type Culture Collection (ATCC). The Hela, the A2780 cell and the HT-29 were from female, Lovo, MV-4-11 AND U87-MG were from male.
Authentication	Cell lines were authenticated using STR testing by Genetic Testing Biotechnology
Mycoplasma contamination	All cell lines were negative for mycoplasma contamination.
Commonly misidentified lines (See ICLAC register)	No commonly misidentified cell lines were used

Plants

Seed stocks	N/R
Novel plant genotypes	N/R
Authentication	N/R
